# A supramolecular lanthanide separation approach based on multivalent cooperative enhancement of metal ion selectivity

**DOI:** 10.1038/s41467-018-02940-7

**Published:** 2018-02-07

**Authors:** Xiao-Zhen Li, Li-Peng Zhou, Liang-Liang Yan, Ya-Min Dong, Zhuan-Ling Bai, Xiao-Qi Sun, Juan Diwu, Shuao Wang, Jean-Claude Bünzli, Qing-Fu Sun

**Affiliations:** 1 State Key Laboratory of Structural Chemistry, Fujian Institute of Research on the Structure of Matter, Chinese Academy of Sciences, Fuzhou, 350002 People’s Republic of China; 2 University of Chinese Academy of Sciences, Beijing, 100049 People’s Republic of China; 3 Xiamen Institute of Rare Earth Materials, Haixi Institute, Chinese Academy of Sciences, Xiamen, 361021 People’s Republic of China; 4 School for Radiological and Interdisciplinary Sciences (RAD-X) and Collaborative Innovation Center of Radiation Medicine of Jiangsu Higher Education Institutions, Soochow University, Suzhou, 215123 People’s Republic of China; 5 Institute of Chemical Sciences and Engineering, Swiss Federal Institute of Technology, 1015 Lausanne, Switzerland

## Abstract

Multivalent cooperativity plays an important role in the supramolecular self-assembly process. Herein, we report a remarkable cooperative enhancement of both structural integrity and metal ion selectivity on metal-organic M_4_L_4_ tetrahedral cages self-assembled from a tris-tridentate ligand (L^1^) with a variety of metal ions spanning across the periodic table, including alkaline earth (Ca^II^), transition (Cd^II^), and all the lanthanide (Ln^III^) metal ions. All these M_4_L^1^_4_ cages are stable to excess metal ions and ligands, which is in sharp contrast with the tridentate (L^2^) ligand and bis-tridentate (L^3^) ligand bearing the same coordination motif as L^1^. Moreover, high-precision metal ion self-sorting is observed during the mixed-metal self-assembly of tetrahedral M_4_L_4_ cages, but not on the M_2_L_3_ counterparts. Based on the strong cooperative metal ion self-recognition behavior of M_4_L_4_ cages, a supramolecular approach to lanthanide separation is demonstrated, offering a new design principle of next-generation extractants for highly efficient lanthanide separation.

## Abstract (web summary)

Lanthanide ions possess similar chemical properties, making their separation from one another challenging. Here the authors show that a tris-tridentate ligand causes high-precision metal ion self-sorting, leading to the selective assembly of tetrahedral M_4_L_4_ cages across the lanthanide series.

## Introduction

Lanthanides, owing to their peculiar electronic structure, are used in a wealth of important applications, including batteries, display devices, contrast agents, magnetic or superconducting materials, and catalytic converters^1^. Because they tend to be distributed in relatively small concentrations and highly insoluble in their pure forms, solvent extraction has been the primary means to separate, purify and recycle lanthanides. Especially, separation from calcium, one of the primary associated elements in lanthanide minerals, such as cerite and loparite-Ce, with an ionic radius similar to those in the middle of the lanthanide series, is crucial in the refining of high-grade lanthanide concentrates^2^. Moreover, nuclear reactors generate a wide variety of waste products including radioactive mixtures, lanthanides, and transition metals, which also require effective conversion and separation for long-term storage and recycling^3^. Owing to the similar ionic radii and coordination numbers/geometries of the rare earth elements, as well as calcium and cadmium metal ions, traditional extraction processes must be conducted in a cascade for complete separation and purification of the desired element^4–6^. New separation techniques that eliminate processing steps and waste are, therefore, of great importance with respect to economic and environmental concerns^7–10^.

Selective binding of metal ions has been a classical research topic in supramolecular chemistry since the very beginning of the field^11–14^. Covalent crown- or cryptand-based receptors possessing a complementary binding pocket are known to have specific ion-recognition properties^15–17^. Meanwhile, designed metal ion hosts that utilize the preorganization effect of the supramolecular scaffold have also been widely studied^18,19^. Moreover, a newly proposed solvent-free extraction/separation process is making use of resins derivatized with macrocyclic ligands^20^. However, almost all of these traditional extractants rely on the selective formation of mononuclear complex from metal ion mixtures. Metal selectivity in multinuclear complexes, especially for lanthanides, has been overlooked for a long time, possibly as a result of the limited number of stable multinuclear lanthanide complexes that exist in solution^21–24^. To the best of our knowledge, there are only a few examples of the control of lanthanide metal selectivity using self-assembly in the literature, and they are mainly based on the formation of dinuclear lanthanide helicate architectures^25–27^.

Multivalency plays an essential role, both in the mediation of biological processes as well as in the construction of supramolecular structures^28–31^. An important example of a multivalent interaction in nature is the interaction between a virus and its host cell, which leads to a stable initial adhesion. Remarkable enhancement in stability has also been proven for the self-assembly of three-dimensional architectures and capsules through the cooperative effect of a vast amount of noncovalent interactions^32–36^. This strong multivalent cooperativity is believed to be the main driving force for the self-sorting phenomena observed in these multi-component assemblies, which bias the complicated system toward the selective formation of one well-defined structure^37–42^. However, until now the term self-sorting has been predominantly used when referring to the organic components in a metal–organic assembly^43–47^. In clear contrast, metal ion self-sorting has scarcely been studied, especially in the cases where ionic radii discrepancy is the only variable^48–50^.

In our previous work, the first stereoselective self-assembly of chiral lanthanide tetrahedral cages was accomplished^51^ with intriguing ligand self-sorting behavior owing to the strong supramolecular cooperative mechanical-coupling effect^52,53^. Herein, we report the unprecedented self-assembly capacity of L^1^ with metal ions spanning across the periodic table, including alkaline earth (Ca^II^), transition (Cd^II^), and all the lanthanide (Ln^III^) metal ions (M), ascribed to the appropriate rigidity of the C_3_ symmetrical scaffolding, high assembly adaptability and adequate chelating affinity with lanthanide ions of the neutral coordination motif. More importantly, this versatile ligand displayed rare and rather high discrimination between metal ions with identical coordination geometries as well as extremely small ionic difference, arising from supramolecular multivalent cooperativity, resulting in absolute or highly efficient metal ion self-recognition during mixed-metal self-assembly process. During both one-pot mixed-metal self-assembly and post-synthetic metal-metathesis experiments, we have observed unprecedented discrimination in favor of including the smaller lanthanide ions in the tetranuclear complexes. For comparison purpose, monodentate (L^2^) and bis-tridentate (L^3^) ligands, bearing the same coordination motif as L^1^, have been synthesized; they turned out to be either unable to self-assemble, or form very fragile mononuclear and dinuclear complexes and no analogous high-precision metal ion self-recognition as that in the mixed-metal self-assembly of tetrahedral M_4_L_4_ cages was observed for the ML_3_ or M_2_L_3_ counterparts. Furthermore, lanthanide extraction separation experiments have also been conducted using alkyl-functionalized ligands, taking advantage of the strongly cooperative metal ion self-recognition behavior of the tetrahedral cages.

## Results

### Self-assembly of M_4_L_4_ cages from L^1^ with various metal ions

**Fig. 1:**
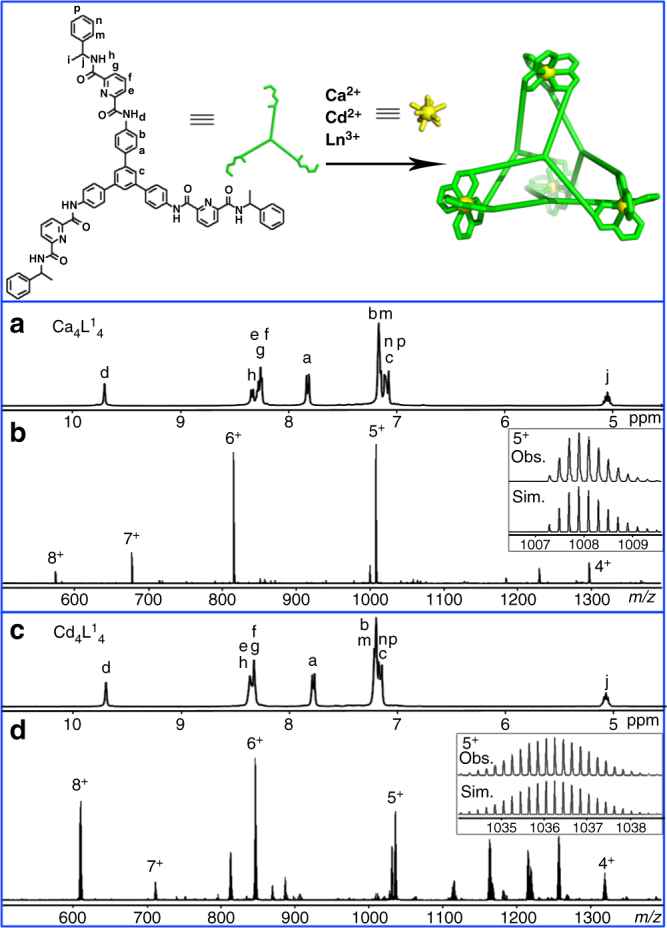
Schematic representation and characterization of the self-assembled M_4_(L^1^)_4_ complexes. ^1^H NMR spectra (400 MHz, CD_3_CN, 298 K) and ESI-TOF-MS spectra of **a**, **b** [Ca_4_(L^1^)_4_](CF_3_SO_3_)_8_, **c**, **d** [Cd_4_(L^1^)_4_](ClO_4_)_8_ with insets showing the observed (Obs.) and simulated (Sim.) isotope patterns of the 5+ peaks

When L^1^ (8 μmol) was treated with Ca(CF_3_SO_3_)_2_ (8 μmol) in CD_3_CN (500 μL) at 40 °C for 1 h, the quantitative formation of a single species was first confirmed by ^1^H NMR (nuclear magnetic resonance) spectroscopy, where a single set of signals was observed, pointing to the equivalence of the ligand strands in the complex (Fig. 1a). The high symmetry of the product is further suggested by the ^1^H-^1^H COSY spectrum, which shows the ligands experiencing identical magnetic environments (Supplementary Fig. 30). Furthermore, ^1^H diffusion ordered spectroscopy (DOSY) shows that all the protons of the tetrahedral cages have the same diffusion coefficient, with a dynamic radii calculated with Stokes-Einstein equation to be about 14.5 Å (Supplementary Fig. 63), which is in good agreement with the reported structure of the Eu_4_(L^1^)_4_ cage^51^. High-resolution electrospray ionization time-of-flight mass spectrometry (ESI-TOF-MS) analyses further confirmed the chemical formula of the isolated tetrahedral cage to be [Ca_4_(L^1^)_4_](CF_3_SO_3_)_8_, as shown in Fig. 1b. The spectrum features a series of peaks corresponding to multi-charged species with progressive loss of anions: for instance, peaks with *m*/*z* equal to 677.3736, 815.0914, and 1007.9010 could be assigned to the charged molecular {[Ca_4_(L^1^)_4_](CF_3_SO_3_)_*n*_}^(8−*n*)+^ complexes with *n* = 1 (7 + ), 2 (6 + ), and 3 (5 + ). The assignments were also verified by carefully comparing the simulated isotopic distributions of the peaks with high-resolution experimental data.

**Fig. 2:**
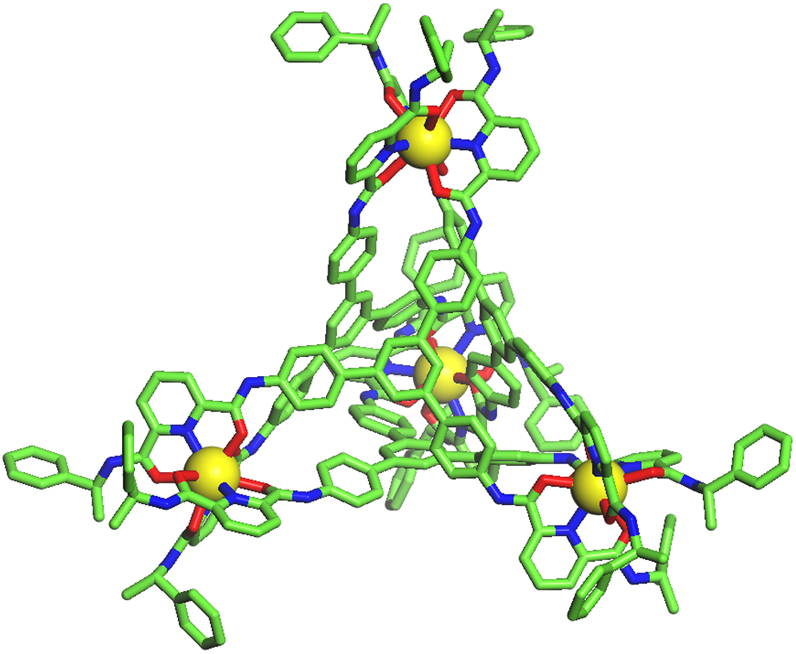
X-ray crystal structure of Cd_4_(L^1^)_4_. For clarity, only the tetrahedral cage framework is shown. Color code for Cd: Yellow, C: green, N: blue, O: red

Similarly, 1D and 2D NMR, ESI-TOF-MS confirmed the formation of a transition metal Cd_4_(L^1^)_4_ cage under the same reaction conditions by replacing the metal source with Cd(ClO_4_)_2_•6H_2_O (Fig. 1c, d, Supplementary Figs. 32, 64, and 210). The structure of this tetrahedral complex was unambiguously confirmed by single-crystal X-ray diffraction studies. Crystals of Cd_4_(L^1^)_4_(ClO_4_)_8_ were obtained by slow vapor diffusion of dichloromethane into an acetonitrile solution of the complex. The X-ray structure of Cd_4_(L^1^)_4_ shares most of the common features as that of the known Eu_4_L^1^_4_ tetrahedral complex, except for crystallizing in a different P6_3_22 space group (Fig. 2, Supplementary Data 1, Supplementary Figs. 1–3 and Supplementary Table 7). It is worth pointing out that in this case all Cd^II^ centers adopt nine-coordinating tricapped trigonal prismatic geometry. This is clearly different from the known Cd_4_L_6_-type cage assembled from another tris-tridentate ligand reported by Rizzuto et al.^54^ recently, where the Cd^II^ adopted a six-coordinating octahedral geometry. So the crystal structure of our Cd_4_(L^1^)_4_ cage represents the first example of discrete supramolecular framework using nine-coordinated Cd^II^ as vertices.

In similar reaction conditions as above, isostructural Ln_4_(L^1^)_4_ tetrahedral complexes were also obtained by reaction of L^1^ with different Ln^III^ metal salts, as confirmed by 1D and 2D NMR spectroscopy (for La^III^, Ce^III^, Pr^III^, Nd^III^, Sm^III^, Yb^III^, Lu^III^, and Y^III^) and ESI-TOF-MS. (Supplementary Figs. 35–72, 211–219). The self-assembled complexes of diamagnetic Y^III^, La^III^, Lu^III^ and weakly paramagnetic Sm^III^ show small chemical shifts compared to those of the ligands, while paramagnetic Ln^III^ ions (Ce^III^, Pr^III^, Nd^III^, Eu^III^, Yb^III^) shift the complexes resonances downfield and upfield obviously (Supplementary Table 1 and Fig. 62).

Moreover, single-crystal X-ray diffraction analysis of La_4_(L^1^)_4_ confirmed the tetrahedral molecular structure arrangement, consistent and isostructural with the reported Eu_4_(L^1^)_4_ cage (Supplementary Data 2, Supplementary Figs. 4–6 and Supplementary Table 8)^51^. Although the discrete M_4_(L^1^)_4_-type tetrahedral complexes are ‘‘isostructural’’ in nature, there are some distinct differences between the Cd^II^ complex and the La^III^ complex in their packing diagrams in the crystal states. Cd_4_(L^1^)_4_ tetrahedral cages are very loosely packed in the ab plane and there are infinite channels with diameters of ca. 2.24 nm along the c axis. As a result, ‘‘void’’ occupancy as much as 50.6% is calculated in the unit cell based on PLATON^55^. In clear contrast, La_4_(L^1^)_4_ tetrahedral cages are much densely packed and only 32.5% ‘‘void’’ occupancy is found by PLATON (Supplementary Fig. 7)^55^. As a result, crystals of Cd_4_(L^1^)_4_ diffract much more weakly than those of La_4_(L^1^)_4_.

The formation of M_4_L_4_ cages with various metal sources was inspiring, considering the facts that, until now, there was only one known discrete tetrahedral structure (Mg_4_L_4_) made from AE^II^ (alkaline earth metal ions), which was self-assembled from anionic 1,3-dicarbonyl ligands^56^; and transition metal Cd^II^ has been known to form only M_6_L_4_-type cages with tris-tridentate ligands^54^; and furthermore, in general, both AE^II^ and TM^II^ (transition metal ions) favor smaller coordination numbers instead of nine-coordinating tricapped trigonal prismatic geometry in their supramolecular coordination compounds^57–59^.

### High-precision metal ion selectivity in M_4_L_4_

Given the high self-assembly versatility of the tris-tridentate ligand (L^1^), metal ion selectivity was examined through mixed-metal self-assembly experiments (Ln_a_^III^/Ln_b_^III^/L^1^ = 1/1/1) and high-precision metal ion self-sorting behavior was observed due to the multivalent cooperative effect. When self-assembly of L^1^ (1.00 equiv) with an equimolar mixture of Ca(ClO_4_)_2_•4H_2_O (1.00 equiv) and La(ClO_4_)_3_•6H_2_O (1.00 equiv) was performed, absolute metal-selective self-organization, or in other words, narcissistic metal ion self-recognition, was observed, as the ^1^H NMR analysis showed only resonances corresponding to La_4_(L^1^)_4_ tetrahedral complexes (Supplementary Fig. 140). The exclusive formation of the homometallic La_4_(L^1^)_4_ complex was also confirmed by ESI-TOF-MS, in which only multiple charged species ascribed to [La_4_(L^1^)_4_(ClO_4_)_*m*_−*n*H]^(12−*m*−*n*)+^ were found (Supplementary Fig. 240).

Analogous to the above self-assembly process, reaction of L^1^ (1.00 equiv) with metal ion mixtures of Cd^II^/La^III^ (1.00 equiv of each) led to the formation of homometallic tetrahedra of La_4_(L^1^)_4_, as ascertained by ^1^H NMR and ESI-TOF-MS (Supplementary Figs. 141 and 241). This absolute self-organization behavior in the mixture of metal ions of identical coordination geometry properties is likely due to the strong supramolecular cooperative mechanical-coupling effect on the tetrahedral cages, facilitating the distinction between metal ions with different electron configurations, ionic charges, and ionic radii.

As for rare earth elements themselves, such absolute self-recognition is much more challenging and intriguing, considering their inherent similar physical and chemical properties, and thus an intricate mixture of heterometallic complexes is predicted in the mixed-lanthanide complexation process. To our delight, reaction of L^1^ (1.00 equiv) with an equimolar mixture of La^III^/Eu^III^ (1.00 equiv of each) turned out to be an absolute self-sorting process, with Eu_4_(L^1^)_4_ as the only product, as confirmed by ^1^H NMR and ESI-TOF-MS (Supplementary Figs. 148 and  247). This complete metal-selective binding phenomenon was totally beyond our expectation as the difference in the ionic radii of La^III^ and Eu^III^ is only 0.10 Å, and in general isostructural compounds will be formed from the same ligand^25^.

**Fig. 3:**
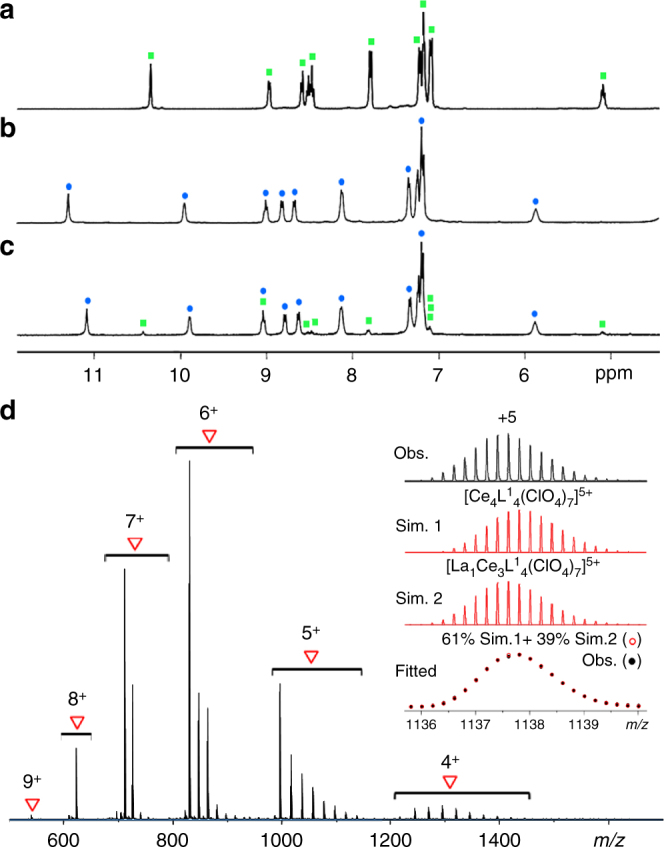
Characterization of mixed-metal self-assembled complexes. ^1^H NMR spectra (400 MHz, CD_3_CN, 298 K) of **a** [La_4_(L^1^)_4_](ClO_4_)_12_, **b** [Ce_4_(L^1^)_4_](CF_3_SO_3_)_12_, and **c** La^III^/Ce^III^ mixed-metal self-assembled complexes. ESI-TOF-MS spectrum **d** of the La^III^/Ce^III^ mixed-metal self-assembled complexes (ClO_4_^−^ salts) with insets showing the observed (Obs.) and fitted isotope patterns of the 5 +  peak from simulations (Sim.) of the component signals

More surprisingly, highly efficient metallic self-organization was also discovered in the case of lanthanide pairs with much smaller ionic radii difference. For example, reaction of ligand L^1^ (4 μmol) with an equimolar mixture of La^III^/Ce^III^ (4 μmol of each) resulted in Ln_4_(L^1^)_4_ tetrahedral coordination cages containing 10.7 % La^III^ and 89.3% Ce^III^, as determined by ^1^H NMR spectroscopy (Fig. 3). Two sets of signals were observed in the ^1^H NMR spectrum of the mixed-metal self-assembled complexes, corresponding to the La^III^- and Ce^III^-coordination environments^27^, as the chemical shifts of proton resonances on the ligand are mainly affected by the coordination environment. The proton signals are identified through comparison with homometallic complexes and further confirmed through post-synthetic metathesis experiments (Supplementary Figs. 142, 197, and 242). Based on the highly symmetrical ^1^H NMR spectrum and the ESI-TOF-MS data, we speculated that Ce_4_(L^1^)_4_ and trace amounts of hetero-metallic tetrahedral complex La_1_Ce_3_(L^1^)_4_ were formed. Nonlinear curve fitting of simulated isotopic patterns in the mass spectrum revealed a composition of 90% Ce^III^ and 10% La^III^, which is in accordance with the ^1^H NMR analysis (Fig. 3d, Supplementary Figs. 242 and 243). The formation of Ln_4_(L^1^)_4_ tetrahedral cages was also confirmed by DOSY spectra (Supplementary Fig. 144). The ^1^H NMR spectrum of the mixed-metal complexes appears to be insensitive to variations in reaction time and temperature (Supplementary Fig. 143), which indicates that the selectivity is thermodynamically favored.

Given the above observation of effective metal ion self-recognition properties, additional one-pot mixed-metal self-assembly experiments (28 combinations in total) were performed to further investigate the degree of metal ion self-recognition (Supplementary Figs. 145–173 and 244–272). In general, L^1^ demonstrates a clear and high preference for smaller sized metal ions in multi-component self-assembly process along the lanthanide series and the selectivity increases with the ionic radii difference, making it an ideal candidate for high-efficiency lanthanide separation and purification (Fig. 4, Supplementary Table 3). Moreover, one-pot tri-metallic self-assembly experiments were also conducted and selectivity results comparable to those of bimetallic systems were obtained, thus expanding the high self-recognition ability of L^1^ to multi-metal self-assembled systems (Supplementary Figs. 195, 196 and 273, 274). Mixed-metal self-assembly experiments with total metal ions to ligand ratios equal to 1:1 (Ln^a^/Ln^b^/L^1^ = 0.5/0.5/1) were also carried out, resulting in the biased formation of two homometallic cages instead of statistically-distributed mixtures of [Ln^a^_*n*_Ln^b^_4-*n*_(L^1^)_4_]^12+^ (*n* = 0–4) species, especially with lanthanide pair of La^III^/Lu^III^, implying the powerful metal ion selectivity of L^1^ (Supplementary Figs. 174–176 and 275–279).

**Fig. 4:**
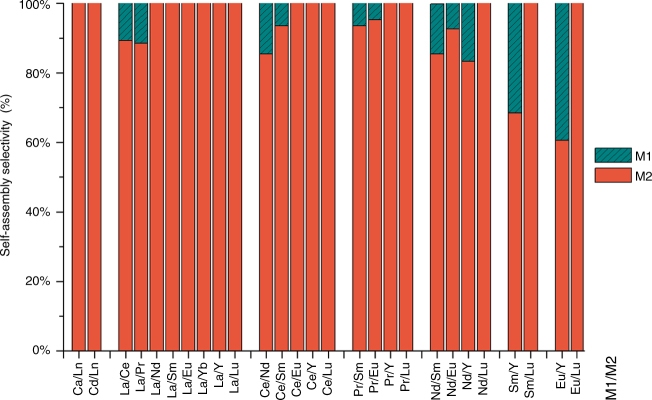
Selective self-assembly of L^1^ with M_2_ over M_1_ in the presence of excess equimolar mixture of M_1_/M_2_. Self-assembly selectivity was defined as [M_2_]/[M_1_] + [M_2_] in the assembled complexes and determined by ^1^H NMR spectroscopy with ± 5% exp. error

We envisioned that the tiny difference in lanthanide ions was amplified by strong cooperative mechanical-coupling effects during the multi-component self-assembly process, resulting in the observed high fidelity metal ion self-recognition behavior. A series of experiments were conducted to verify the vital function of supramolecular cooperativity in this surprising lanthanide separation and purification.

## Discussion

**Fig. 5:**
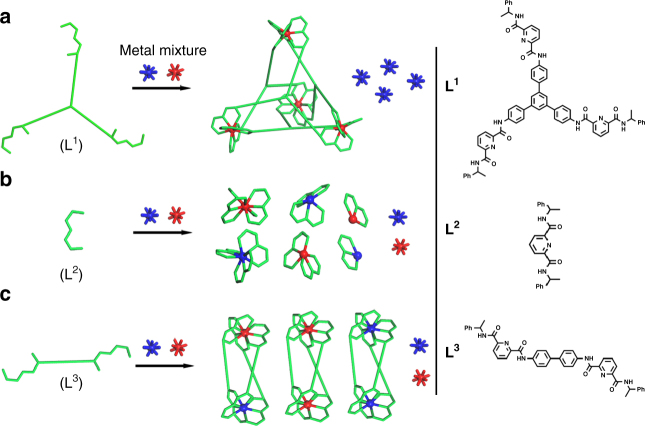
Cartoon representations for metal-ion self-recognition. Self-assembly of ligands **a** L^1^, **b** L^2^, and **c** L^3^ with a mixture of alkaline earth (AE^II^), transition (TM^II^) and lanthanide (Ln^III^) metal ions

To verify the extent of the multivalent cooperative effect in the unprecedented selective formation of M_4_L_4_ tetrahedral cages, ligands L^2^ and L^3^ (Fig. 5), which contain the same pyridine-2,6-dicarboxamide chelating moiety and are known to form mononuclear M(L^2^)_3_ and dinuclear M_2_(L^3^)_3_ complexes with lanthanides, were synthesized according to known procedures^22,60^. Titration experiments were performed in CD_3_CN/CDCl_3_ for L^1-3^ and Ca^II^ with increasing [M]/[L] ratios (*R*) for comparison of structure integrity. The stoichiometry of the tetrahedral assembly Ca_4_(L^1^)_4_ was confirmed by ^1^H NMR varying *R* from 0 to 2.0 (Supplementary Fig. 107). It is noteworthy that intermediate spectra (0.2≤*R*_Ca/L1_≤1.0) are simply additions of the ligand and tetrahedral assembly spectra. Moreover, the Ca_4_L^1^_4_ complexes maintained structural integrity even when the ratio of Ca^II^/L^1^ increased to 5.0. This high stability of M_4_(L^1^)_4_ toward excess metal ions and ligands serves as a prerequisite for the metal ion self-recognition experiments discussed above.

However, during a similar titration experiment with L^2^, ^1^H NMR, and ESI-TOF-MS showed a mixture of Ca(L^2^)_3_, the observed predominant Ca^II^-containing species, and abundant free ligands at *R* < 0.33 and a mixture of Ca(L^2^)_*n*_ (*n* = 1–3) at *R* > 0.33 (Supplementary Figs. 110 and 236). This indicates the low stability of Ca(L^2^)_3_ in comparison with Ca_4_(L^1^)_4_ tetrahedral cages. In a similar titration experiment with L^3^ (0.13≤*R*_Ca/L3_≤2.00, in CD_3_CN/CDCl_3_ = 1/3), CaL^3^_*n*_ complexes were speculated to form considering the ^1^H NMR spectra, nevertheless, large amounts of free ligands were observed in the ESI-TOF-MS, together with small signals from CaL^3^_*n*_ (*n* = 1–3), which can be ascribed to the high fragility of the complexes caused by the low association capacity of Ca^II^-containing complexes (Supplementary Figs. 113 and 238). Titration experiments of Cd^II^ and Eu^III^ with L^1-3^ gave similar variation tendency of stability as that of Ca^II^ (Supplementary Figs. 106–114 and 234–239). The enhanced structural stability from monometallic, dimetallic to tetrametallic self-assembly complexes followed the trend of increasing number of components and coordination interactions, which in turn confirmed the multivalent cooperative effect on the structural integrity of self-assembly systems.

As M(L^2^)_3_ is not applicable to the mixed-metal complexation process due to the rather low structural stability, metal ion selectivity during the formation of M_2_L_3_ and M_4_L_4_ was then compared using ligands L^3^ and L^1^ under similar experimental procedure and it was found that the ditopic ligand L^3^ has much poorer ion selectivity compared with L^1^ (Fig. 6, Supplementary Figs. 177–188 and 280–288).

**Fig. 6:**
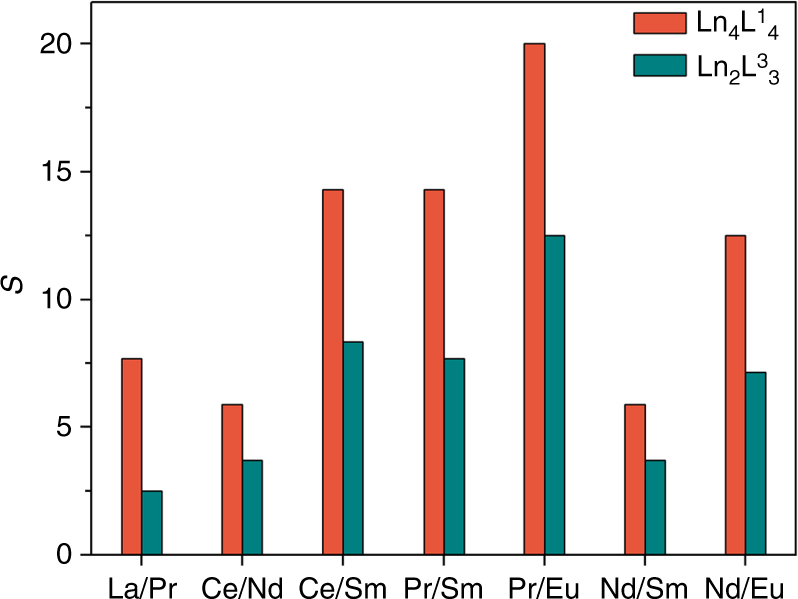
Comparison of metal ion selectivity *S* in Ln_2_L^3^_3_ and Ln_4_L^1^_4_ complexes. *S*, defined as [Ln^b^(III)]/[Ln^a^(III)] and determined by ^1^H NMR spectroscopy

The highly efficient recognition observed during the mixed-metal self-assembly process indicates a substantial difference in the binding affinity of L^1^ toward different lanthanide ions^61–63^. Post-synthetic metal-metathesis experiments were conducted to shed light on the mechanism of this highly controlled metal-selective self-assembly process. It is worth mentioning that the substitution rate and idealized relative formation constants for each metal combination depend on the difference in the ionic radii, and a larger difference results in faster substitution process and larger relative formation constants (Supplementary Figs. 197–203 and 295). Eu_4_(L^1^)_4_, for instance, has a formation constant of at least nine orders of magnitude higher than that of La_4_(L^1^)_4_ (Supplementary Table 4). Such a huge difference in formation constants serves as the primary driving force for the highly efficient metal ion recognition, and explains the complete Eu^III^ selectivity observed in the La^III^/Eu^III^ mixed-metal self-assembly discussed above.

Post-synthetic metal-metathesis experiments were also performed using L^3^ for comparing relative formation constants with L^1^ (Supplementary Figs. 204–208). Compared with the Ln_4_(L^1^)_4_ complexes, Ln_2_(L^3^)_3_ has much smaller relative formation constants for the same metal combinations (Supplementary Table 5). The increased thermodynamic stability and metal ion selectivity on going from dimetallic to tetrametallic complexes confirm that the cooperativity is dramatically enhanced with increased numbers of multitopic ligand chelation.

In addition to supramolecular multivalent cooperativity, structural stability of the supramolecular polyhedra and the rational choice of the multidentate coordination sites also contribute to the high-efficient metal ion selectivity. As a precondition for efficient metal ion selective self-assembly, the structural stability relies on both framework rigidity of the ligand and chelating affinity of the coordinating moieties for metal ions. Hamacek’s group has reported a pyridine-2,6-dicarboxamide (pcam) based tripodal ligand with flexible bridging units 1,1,1-tris(aminomethyl)ethane^64^. Tetrahedral complexes [Ln_4_L_4_]^12+^ (Ln = Eu, Tb, Lu) were formed in the self-assembly process. ^1^H NMR and ESI-MS titration show the appearance of other species when either ligands (LnL_3_, LnL_2_, Ln_2_L_3_, Ln_3_L_4_) or metal ions (Ln_4_L_3_, Ln_3_L_2_) are in excess. Moreover, the ^1^H NMR spectrum excluded the formation of a tetrahedral complex with La^III^. The rather low stability is speculated to derive from the flexibility of the ligand. Moreover, the Ln_4_L_4_ complexes assembled from the flexible ligand have rather small relative formation constants, with log*β*_La/Eu_ = 0.5 and log*β*_Tb/Lu_ = 1.4 (ref. ^61^). Reaction of lanthanide ions with a rigid tripodal ligand, three pcam coordination units connected with rigid triptycene moiety, generates low symmetry complexes in presence of excess ligand and a trinuclear sandwich complex [Eu_3_L_2_]^9+^ when the metal/ligand ratio [Eu]/[L] reaches 3:2 (ref. ^65^). For tris(tridentate) ligands with similar coordination moieties, both scaffold rigidity and geometry exert great influence on the structural stability. We hypothesize ligands with moderate rigidity operate as levers between the four metal centers on the tetrahedral vertices, in a way that a small distortion in coordination geometry on one metal center is transferred to the other three vertices, leading to an enlarged energy barrier in comparison with the perfectly symmetrical tetrahedral framework. However, changing one of the metal ions on a flexible tetrahedron does not have such an effect. Thus, we conclude that ligand rigidity enhances mechanical-coupling effects within the framework, and in turn contributes to cooperative enhancement of both stability and metal ion selectivity.

Higher chelating affinity between the coordinating moieties and metal ions would improve the structural stability but it has a complicated impact on metal ion selectivity. Ligands with ideal coordination sites for lanthanide separation in a supramolecular system are expected to coordinate with the entire lanthanide series while possessing distinct binding affinity toward different lanthanide ions. However, in addition to the efficient chelating ability of the ligand with all the lanthanide ions into specific structures, moderate binding affinity is necessary for practical usage. Overlarge binding affinity could result in kinetically trapped assemblies and hinder further transformation into the thermodynamically favored product (needing high temperature or prolonged reaction time). Hooley’s group has reported an acylhydrazone-phenolate based bis(tridentate) ligand with anionic coordination sites, which assembles with lanthanide ions into Ln_2_L_3_ complexes in DMSO and shows kinetic discrimination among lanthanide ions with a preference for smaller metals and a thermodynamic preference for larger metals^25^. However, in this system, the thermodynamic equilibrium is not reached even after 20 h. In comparison, the pcam-based Ln_4_(L^1^)_4_ complexes dissociate in DMSO, implying much weaker binding affinity between ligands and metal ions and the thermodynamic equilibrium is reached on a minute timescale. Supramolecular systems that can rapidly achieve thermodynamic equilibrium are required for efficient lanthanide separation for economic and practical consideration. Moreover, the pcam-based ditopic and tritopic ligands possess much higher lanthanide ion selectivity in the thermodynamically favored complexes owing to their moderate chelating affinity (Supplementary Figs. 186–188 and Table 2).

**Fig. 7:**
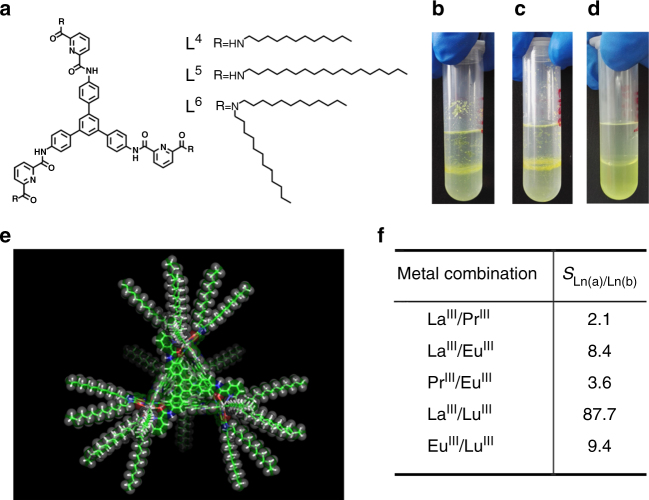
Demonstration of a lanthanide separation strategy using the multivalent cooperative effect of the tetranuclear cage complexes. **a** Modified structures of ligands L^4–6^ for solvent extraction experiments; Phase separation properties for La^III^/Lu^III^ mixed-metal self-assemblies using **b** L^4^, **c** L^5^, and **d** L^6^; **e** Simulated structure of Ln_4_L^6^_4_; **f** Separation factors (with ± 5% exp. error) obtained from lanthanide solvent extraction experiment

Cooperative enhancement of lanthanide selectivity in the formation of the tetrahedral cages indicated that tris(tridentate) ligands may serve as good extractants for lanthanide separation. As a proof-of-concept, L^4–6^, with hydrophobic alkyl groups introduced onto the periphery to afford better phase separation (Fig. 7), were synthesized and tested in lanthanide extraction experiments. The introduction of di-dodecanamine groups (L^6^) finally gave good dispersity of the complexes in CHCl_3_ for liquid–liquid extraction (Fig. 7b–d). In a typical procedure, L^6^ (12 μmol) was treated with an equimolar mixture of La(OTf)_3_ and Lu(OTf)_3_ (12 μmol of each) in 2 mL mixed solvent of CH_3_CN/CHCl_3_ (1:1 v/v, for better solubility) at room temperature. The turbid suspension of ligands became clear within 5 min with gentle shaking, and ^1^H NMR and ESI-TOF-MS confirmed the exclusive formation of Lu_4_(L^6^)_4_ complexes (Supplementary Figs. 192 and 292). After the reaction solvent was evaporated under reduced pressure, 2 mL CHCl_3_ was added to the self-assembled complex system, followed by the addition of the same volume of water to extract the unreacted La(OTf)_3_. The structural integrity of Lu_4_(L^6^)_4_ in the organic phase after extraction was ascertained by ^1^H NMR spectroscopy and the metal contents in the two separated phases were measured using inductively coupled plasma mass spectrometry (ICP-MS). The separation factor, defined as the ratio of the distribution coefficient of each lanthanide in the aqueous and organic phase (*S*_Ln(a)/Ln(b)_ = *D*_Ln(a)_/*D*_Ln(b)_, distribution coefficient *D*_Ln(a)_ = [Ln_(a)_]_aq_/[Ln_(a)_]_org_), was calculated to be ca. 87.7 without further optimization of the extraction process (with ± 5% exp. error). Further separation factors measured for some representative metal combinations are listed in Fig. 7f (Supplementary Figs. 189–194 and 289–294). Parallel extraction experiments were also conducted, suggesting good validity of the separation efficiency (Supplementary Table 6). In view of the poor water stability of the core cage compound, which in fact will fall apart when exposed to CD_3_CN/D_2_O (1:1 v/v) mixed solvent, we anticipate that separation factors can be further increased with this strategy by employing more stable tetrahedral frameworks. Furthermore, this supramolecular separation strategy is very promising in efficient actinides/actinides and actinides/lanthanides separation for the treatment of radioactive waste and the recycling of minor actinides, considering the similarities in oxidation states, chemical properties and ionic radii between actinides and lanthanides.

In summary, this supramolecular lanthanide extraction and separation approach has been established based on an exclusive metal ion self-sorting of tetrahedral cage complexes. Unprecedented separation abilities have been achieved by taking advantage of the multivalent supramolecular cooperativity of these complexes. As such, this study provides new insights into the design of next-generation lanthanide extractants. Further application of this strategy to lanthanide/actinide separation, or purification of other metals in general, is expected.

## Methods

### Materials

Deuterated solvents were purchased from Admas and J&K scientific. 1D and 2D NMR spectra were measured on a Bruker-BioSpin AVANCE III HD (400 MHz) spectrometer. ^1^H NMR chemical shifts were determined with respect to residual solvent signals of the deuterated solvents used. ^1^H NMR integrations were performed using TOPSPIN 2.1 software. ESI-TOF-MS were recorded on an Impact II UHR-TOF mass spectrometry from Bruker, with ESI-L low concentration tuning mix (from Agilent Technologies) as the internal standard (Accuracy < 3 ppm). Data analyses and simulations of ESI-TOF-MS were processed with the Bruker Data Analysis software (Version 4.3). ICP-MS analysis was performed on a Thermo Finnigan high-resolution magnetic sector Element 2 ICP-MS instrument. Unless otherwise stated, all chemicals and solvents were purchased from commercial companies and used without further purification.

Caution! Perchlorate salts are potentially explosive and should be handled carefully in small quantities.

### Synthesis and physical properties of [M_4_L^1^_4_]^8+^ and [Ln_4_L^1^_4_]^12+^

A solution of M(CF_3_SO_3_)_2_ (or M(ClO_4_)_2_•4H_2_O, M = Ca, Cd) or Ln(ClO_4_)_3_•6H_2_O (10.0 μmol, 1 equiv) (Ln = La, Pr, Nd, Sm, Eu, Yb, Y) in 0.50 mL CH_3_CN was added to a white suspension of L^1^ (either the *R* or *S* enantiomer form; 11.08 mg, 10.0 μmol, 1 equiv) in 1.00 mL CH_3_CN. After stirring at 40 °C for 1 h, the turbid suspension of ligands turned into homogeneous yellow solution. ^1^H NMR and ESI-TOF-MS showed the quantitative formation of M_4_(L^1^)_4_ complexes. The solvent is removed under reduced pressure to give a yellow powder product (Supplementary Figs. 29–72 and 209–219).

The above experimental procedure applies to the self-assembly of Ln(CF_3_SO_3_)_3_ (Ln = La, Ce, Pr, Nd, Sm, Eu, Yb, Lu, Y) with L^1^ as well. And it is worth mentioning that Ce(ClO_4_)_3_•6H_2_O and Lu(ClO_4_)_3_•6H_2_O are not used in the preparation of [Ln_4_(L^1^)_4_]^12+^ due to the poor solubility of their self-assembled complexes in CH_3_CN. However, in the existence of extra Ln(III) in mixed-metal one-pot self-assembly experiments, [Ce_4_(L^1^)_4_](ClO_4_)_12_ and [Lu_4_(L^1^)_4_](ClO_4_)_12_ have rather good solubility in CH_3_CN, which facilitates the manipulation and characterization of the metal-selective self-assembly process. No change in the NMR spectra was observed for the Ln_4_(L^1^)_4_ complexes with either ClO_4_^−^ or CF_3_SO_3_^−^ as the counter anions. No signals were observed in the negative range (−20–0 ppm) in the ^1^H NMR spectra.

[Ca_4_L^1^_4_](CF_3_SO_3_)_8_: ^1^H NMR (400 MHz, CD_3_CN) *δ* 9.90 (s, 3 H), 8.44 (d, *J* = 7.2 Hz, 3 H), 8.38–8.23 (m, 9 H), 7.88 (d, *J* = 8.5 Hz, 6 H), 7.31 – 7.03 (m, 24 H), 5.16–5.03 (m, 3 H), 1.71 (d, *J* = 7.1 Hz, 9 H). ^13^C NMR (101 MHz, CD_3_CN) *δ* 165.11, 164.55, 149.26, 148.93, 143.54, 141.26, 140.16, 137.01, 128.98, 127.82, 127.19, 126.65, 125.07, 124.73, 123.14, 122.91, 122.63, 122.04, 119.96, 51.41, 21.38. ESI-TOF-MS calcd. for [M-7(CF_3_SO_3_^−^)]^7+^ 677.3686, found 677.3736; calcd. for [M-6(CF_3_SO_3_^−^)]^6+^ 815.0887, found 815.0914; calcd. for [M-5(CF_3_SO_3_^−^)]^5+^ 1007.8970, found 1007.9010; calcd. for [M-4(CF_3_SO_3_^−^)]^4+^ 1297.1093, found 1297.3668.

[Cd_4_L^1^_4_](ClO_4_)_8_: ^1^H NMR (400 MHz, CD_3_CN) *δ* 9.80 (s, 3 H), 8.49–8.32 (m, 12 H), 7.83 (d, *J* = 8.6 Hz, 6 H), 7.35–7.12 (m, 24 H), 5.12 (p, *J* = 7.1 Hz, 3 H), 1.74 (d, *J* = 7.0 Hz, 9 H). ^13^C NMR (101 MHz, CD_3_CN) *δ* 163.61, 162.89, 146.76, 146.45, 143.65, 141.69, 140.23, 137.09, 136.96, 129.05, 127.88, 127.08, 126.71, 125.70, 125.46, 122.82, 122.67, 51.66, 21.51. ESI-TOF-MS calcd. for [M-8(ClO_4_^−^)]^8+^ 610.2990, found 610.6775; calcd. for [M-7(ClO_4_^−^)]^7+^ 711.7629, found 711.6234; calcd. for [M-6(ClO_4_^−^)]^6+^ 846.8814, found 846.8852; calcd. for [M-5(ClO_4_^−^)]^5+^ 1036.2475, found 1036.2516; calcd. for [M-4(ClO_4_^−^)]^4+^ 1320.0464, found 1320.3012.

[La_4_L^1^_4_](ClO_4_)_12_: ^1^H NMR (400 MHz, CD_3_CN) *δ* 10.35 (s, 3 H), 8.96 (d, *J* = 6.4 Hz, 3 H), 8.59 (d, *J* = 7.6 Hz, 3 H), 8.52 (d, *J* = 8.0 Hz, 3 H), 8.47 (t, *J* = 7.8 Hz, 3 H), 7.79 (d, *J* = 8.4 Hz, 6 H), 7.22 (d, *J* = 8.0 Hz, 6 H), 7.17 (s, 9 H), 7.09 (t, *J* = 5.2 Hz, 9 H), 5.09 (t, *J* = 6.8 Hz, 3 H), 1.70 (d, *J* = 8.0 Hz, 9 H). ^13^C NMR (101 MHz, CD_3_CN) *δ* 168.17, 167.35, 149.43, 149.09, 143.85, 142.34, 140.34, 138.56, 135.62, 129.41, 129.11, 128.18, 127.67, 127.27, 126.39, 123.91, 123.57, 117.98, 52.78, 29.91, 21.27. ESI-TOF-MS calcd. for [M-9(ClO_4_^−^)-3(HClO_4_)]^9+^ 553.9310, found 553.9323; calcd. for [M-8(ClO_4_^−^)-4(HClO_4_)]^8+^ 623.0465, found 623.0479; calcd. for [M-7(ClO_4_^−^)-3(HClO_4_)]^7+^ 740.6109, found 740.6123; calcd. for [M-6(ClO_4_^−^)-4(HClO_4_)]^6+^ 863.8781, found 863.8795; calcd. for [M-5(ClO_4_^−^)-4(HClO_4_)]^5+^ 1056.4434, found 1056.4447; calcd. for [M-4(ClO_4_^−^)-4(HClO_4_)]^4+^ 1345.5414, found 1345.5422.

[Ce_4_L^1^_4_](CF_3_SO_3_)_12_: ^1^H NMR (400 MHz, CD_3_CN) *δ* 11.38 (s, 3 H), 10.07 (d, *J* = 4.4 Hz, 3 H), 8.99 (t, *J* = 8.0 Hz, 3 H), 8.84 (d, *J* = 8.0 Hz, 3 H), 8.72 (d, *J* = 8.0 Hz, 3 H), 8.12 (d, *J* = 7.2 Hz, 6 H), 7.35 (d, *J* = 7.2 Hz, 6 H), 7.23 (t, *J* = 5.2 Hz, 6 H), 7.16 (t, *J* = 5.2 Hz, 12 H), 5.83 (s, 3 H), 1.94 (d, *J* = 7.2 Hz, 3 H). ^13^C NMR (101 MHz, CD_3_CN) *δ* 164.99, 164.03, 146.87, 146.53, 142.72, 142.54, 140.47, 138.65, 136.13, 131.63, 131.40, 129.14, 128.13, 127.88, 126.56, 123.96, 123.71, 123.04, 119.86, 53.65, 21.47. ESI-TOF-MS calcd. for [M-8(CF_3_SO_3_^−^)-4(HCF_3_SO_3_)]^8+^ 623.5460, found 623.5462; calcd. for [M-7(CF_3_SO_3_^−^)-5(HCF_3_SO_3_)]^7+^ 712.4800, found 712.4802; calcd. for [M-6(CF_3_SO_3_^−^)-6(HCF_3_SO_3_)]^6+^ 831.0588, found 831.2261; calcd. for [M-5(CF_3_SO_3_^−^)-7(HCF_3_SO_3_)]^5+^ 997.0692, found 997.2694; calcd. for [M-4(CF_3_SO_3_^−^)-6(HCF_3_SO_3_)]^4+^ 1321.3150, found 1321.3137.

[Eu_4_L^1^_4_](ClO_4_)_12_: ^1^H NMR (400 MHz, CD_3_CN) ^1^H NMR (400 MHz, CD_3_CN) *δ* 8.67 (s, 6 H), 7.94 (d, *J* = 7.3 Hz, 6 H), 7.73 (s, 3 H), 7.22 (d, *J* = 7.8 Hz, 3 H), 7.05 (s, 9 H), 6.94 (d, *J* = 19.8 Hz, 9 H), 6.45 (d, *J* = 7.2 Hz, 3 H), 6.38 (d, *J* = 7.8 Hz, 3 H), 5.95 (s, 3 H), 4.76 (s, 3 H), 1.93 (d, *J* = 5.7 Hz, 9 H). ^13^C NMR (101 MHz, CD_3_CN) *δ* 164.746, 159.907, 156.081, 143.316, 142.099, 140.586, 139.522, 135.987, 129.004, 127.951, 127.855, 125.991, 125.174, 124.671, 117.910, 92.685, 92.290, 52.082, 22.246. ESI-TOF-MS calcd. for [M-8(ClO_4_^−^)-4(HClO_4_)]^8+^ 629.5535, found 629.5544; calcd. for [M-7(ClO_4_^−^)-5(HClO_4_)]^7+^ 719.3459, found 719.3468; calcd. for [M-6(ClO_4_^−^)-6(HClO_4_)]^6+^ 839.0690, found 839.0700; calcd. for [M-5(ClO_4_^−^)-7(HClO_4_)]^5+^ 1006.6813, found 1006.6819; calcd. for [M-4(ClO_4_^−^)-6(HClO_4_)]^4+^ 1308.3277, found 1308.5779.

NMR and ESI-TOF-MS characterization of [Pr_4_L^1^_4_](ClO_4_)_12_, [Nd_4_L^1^_4_](ClO_4_)_12_, [Sm_4_L^1^_4_](ClO_4_)_12_, [Y_4_L^1^_4_](ClO_4_)_12_, [Yb_4_L^1^_4_](CF_3_SO_3_)_12_ and [Lu_4_L^1^_4_](CF_3_SO_3_)_12_ can be seen in Supplementary Methods.

### Synthesis and physical properties of [Ln_4_L^4-6^_4_]^12+^

A solution of Ln(CF_3_SO_3_)_3_ (10.0 μmol, 1 equiv) (Ln = La, Ce, Pr, Eu, Lu) in 0.50 mL CD_3_CN was added to a suspension of L^4-6^ (10.0 μmol, 1 equiv) in 1.00 mL CD_3_CN/CDCl_3_ (1/1 v/v). Homogeneous yellow solution was obtained after stirring at room temperature for 1 h. NMR and ESI-TOF-MS spectra showed the quantitative formation of [Ln_4_L^4-6^_4_](CF_3_SO_3_)_12_ (Supplementary Figs. 90–105 and 227–233).

[La_4_L^6^_4_](CF_3_SO_3_)_12_: ^1^H NMR (400 MHz, CD_3_CN) *δ* 11.05 (s, 3 H), 8.79 (d, *J* = 8.1 Hz, 3 H), 8.48 (t, *J* = 7.9 Hz, 3 H), 8.08 (d, *J* = 7.8 Hz, 3 H), 7.87 (d, *J* = 8.4 Hz, 6 H), 7.29 (d, *J* = 8.4 Hz, 6 H), 7.18 (s, 3 H), 3.63 (s, 3 H), 3.54 (d, *J* = 6.0 Hz, 3 H), 3.27 (s, 3 H), 3.16 (d, *J* = 17.8 Hz, 3 H), 1.80 (s, 6 H), 1.46 (s, 6 H), 1.25 (d, *J* = 16.2 Hz, 96 H), 1.10 (s, 12 H), 0.85 (dd, *J* = 9.6, 6.9 Hz, 18 H). ^13^C NMR (101 MHz, CD_3_CN) *δ* 169.29, 167.49, 150.09, 149.62, 143.26, 140.22, 138.63, 135.50, 128.38, 127.42, 123.29, 122.55, 119.37, 51.11, 49.53, 32.17, 32.07, 30.05, 30.01, 29.96, 29.81, 29.76, 29.65, 29.59, 29.51, 29.30, 28.75, 27.38, 26.86, 26.50, 22.89, 22.81, 14.00, 13.95. ESI-TOF-MS calcd. for [M-8(CF_3_SO_3_^−^)-4(HCF_3_SO_3_)]^8+^ 971.7668, found 971.7673; calcd. for [M-7(CF_3_SO_3_^−^)-5(HCF_3_SO_3_)]^7+^ 1110.4468, found 1110.4469; calcd. for [M-6(CF_3_SO_3_^−^)-6(HCF_3_SO_3_)]^6+^ 1295.3533, found 1295.3525; calcd. for [M-5(CF_3_SO_3_^−^)-7(HCF_3_SO_3_)]^5+^ 1554.2226, found 1554.2221.

[Pr_4_L^6^_4_](CF_3_SO_3_)_12_: ^1^H NMR (400 MHz, CD_3_CN) *δ* 12.56 (s, 3 H), 10.30 (s, 3 H), 10.06 (s, 3 H), 9.40 (s, 3 H), 6.10 (s, 9 H), 5.90 (s, 6 H), 3.68 (d, *J* = 24.7 Hz, 6 H), 2.96 (s, 3 H), 2.55 (s, 3 H), 2.07 (s, 3 H), 1.87 (s, 3 H), 1.24 (d, *J* = 11.3 Hz, 96 H), 0.91 (d, *J* = 5.8 Hz, 6 H), 0.90–0.83 (m, 18 H), 0.70 (s, 6 H), 0.54 (s, 6 H). ESI-TOF-MS calcd. for [M-8(CF_3_SO_3_^−^)-4(HCF_3_SO_3_)]^8+^ 972.7675, found 972.7676; calcd. for [M-7(CF_3_SO_3_^−^)-5(HCF_3_SO_3_)]^7+^ 1111.5904, found 1111.5908; calcd. for [M-6(CF_3_SO_3_^−^)-6(HCF_3_SO_3_)]^6+^ 1296.6875, found 1296.6874; calcd. for [M-5(CF_3_SO_3_^−^)-7(HCF_3_SO_3_)]^5+^ 1555.8236, found 1555.8223; calcd. for [M-4(CF_3_SO_3_^−^)-6(HCF_3_SO_3_)]^4+^ 2019.5074, found 2019.5039.

[Eu_4_L^6^_4_](CF_3_SO_3_)_12_: ^1^H NMR (400 MHz, CD_3_CN) *δ* 8.46 (s, 6 H), 7.84 (s, 6 H), 7.65 (s, 6 H), 7.29 (s, 3 H), 6.97 (s, 3 H), 6.23 (s, 3 H), 6.23 (s, 1 H), 3.51 (s, 6 H), 3.40 (s, 3 H), 3.13 (s, 3 H), 1.73 (s, 6 H), 1.21 (t, *J* = 47.2 Hz, 114 H), 1.02–0.64 (m, 18 H). ^13^C NMR (101 MHz, CD_3_CN) *δ* 154.77, 145.93, 140.23, 139.37, 135.52, 127.36, 124.68, 95.36, 94.25, 49.28, 47.79, 32.11, 32.00, 30.09, 30.04, 29.97, 29.93, 29.75, 29.70, 29.64, 29.60, 29.44, 29.27, 27.78, 27.53, 26.23, 22.82, 22.74, 13.98, 13.92. ESI-TOF-MS calcd. for [M-8(CF_3_SO_3_^−^)-4(HCF_3_SO_3_)]^8+^ 978.2739, found 978.2744; calcd. for [M-7(CF_3_SO_3_^−^)-5(HCF_3_SO_3_)]^7+^ 1117.8834, found 1117.8844; calcd. for [M-6(CF_3_SO_3_^−^)-6(HCF_3_SO_3_)]^6+^ 1304.0294, found 1304.1962; calcd. for [M-5(CF_3_SO_3_^−^)-7(HCF_3_SO_3_)]^5+^ 1564.6339, found 1564.6327; calcd. for [M-4(CF_3_SO_3_^−^)-8(HCF_3_SO_3_)]^4+^ 1955.5405, found 1955.5380.

[Lu_4_L^6^_4_](CF_3_SO_3_)_12_: ^1^H NMR (400 MHz, CD_3_CN) *δ* 11.05 (s, 3 H), 8.87 (d, *J* = 7.6 Hz, 3 H), 8.52 (t, *J* = 7.6 Hz, 3 H), 8.12 (d, *J* = 7.6 Hz, 3 H), 7.82 (d, *J* = 8.0 Hz, 6 H), 7.31 (d, *J* = 8.0 Hz, 6 H), 7.24 (s, 3 H), 3.72 (s, 3 H), 3.55 (dd, *J* = 16.9, 12.9 Hz, 3 H), 3.15 (s, 3 H), 1.85 (s, 6 H), 1.40–1.27 (m, 102 H), 1.13 (d, *J* = 6.9 Hz, 6 H), 1.03 (s, 6 H), 0.90–0.85 (m, 18 H). ESI-TOF-MS calcd. for [M-8(CF_3_SO_3_^−^)-4(HCF_3_SO_3_)]^8+^ 989.7840, found 989.7853; calcd. for [M-7(CF_3_SO_3_^−^)-5(HCF_3_SO_3_)]^7+^ 1131.0378, found 1131.0398; calcd. for [M-6(CF_3_SO_3_^−^)-6(HCF_3_SO_3_)]^6+^ 1319.3763, found 1319.3773; calcd. for [M-5(CF_3_SO_3_^−^)-7(HCF_3_SO_3_)]^5+^ 1583.0501, found 1583.0501; calcd. for [M-4(CF_3_SO_3_^−^)-6(HCF_3_SO_3_)]^4+^ 2053.5405, found 2503.7903.

[Eu_4_L^4^_4_](CF_3_SO_3_)_12_: ^1^H NMR (400 MHz, CD_3_CN) *δ* 8.68 (s, 6 H), 7.93 (d, *J* = 7.3 Hz, 6 H), 7.77 (s, 3 H), 7.49 (s, 3 H), 7.26 (t, *J* = 8.2 Hz, 3 H), 6.69 (d, *J* = 7.5 Hz, 3 H), 6.48 (d, *J* = 7.0 Hz, 3 H), 4.80 (s, 3 H), 3.80 (s, 6 H), 1.77 (s, 6 H), 1.61 (d, *J* = 11.9 Hz, 6 H), 1.47 (s, 6 H), 1.32 (dd, *J* = 18.7, 13.8 Hz, 42 H), 0.89 (dd, *J* = 8.9, 4.4 Hz, 9 H). ESI-TOF-MS calcd. for [M-9(CF_3_SO_3_^−^)-3(HCF_3_SO_3_)]^9+^ 645.3267, found 645.3273; calcd. for [M-8(CF_3_SO_3_^−^)-4(HCF_3_SO_3_)]^8+^ 725.8667, found 725.8678; calcd. for [M-7(CF_3_SO_3_^−^)-4(HCF_3_SO_3_)]^7+^ 850.8408, found 850.8414; calcd. for [M-6(CF_3_SO_3_^−^)-4(HCF_3_SO_3_)]^6+^ 1017.4730, found 1017.4729; calcd. for [M-5(CF_3_SO_3_^−^)-4(HCF_3_SO_3_)]^5+^ 1250.7580, found 1250.7570; calcd. for [M-4(CF_3_SO_3_^−^)-4(HCF_3_SO_3_)]^4+^ 1600.6856, found 1600.6835.

[Eu_4_L^5^_4_](CF_3_SO_3_)_12_: ^1^H NMR (400 MHz, CD_3_CN) *δ* 8.62 (s, 6 H), 7.90 (s, 6 H), 7.73 (s, 3 H), 7.59 (s, 3 H), 7.25 (s, 3 H), 6.78 (s, 3 H), 6.56 (s, 3 H), 4.93 (s, 3 H), 3.78 (s, 6 H), 1.27 (s, 96 H), 0.87 (s, 9 H). ESI-TOF-MS calcd. for [M-8(CF_3_SO_3_^−^)-4(HCF_3_SO_3_)]^8+^ 852.0077, found 852.0065; calcd. for [M-7(CF_3_SO_3_^−^)-4(HCF_3_SO_3_)]^7+^ 995.1451, found 995.2864; calcd. for [M-6(CF_3_SO_3_^−^)-4(HCF_3_SO_3_)]^6+^ 1185.8280, found 1185.8255; calcd. for [M-5(CF_3_SO_3_^−^)-6(HCF_3_SO_3_)]^5+^ 1392.8002, found 1392.7972; calcd. for [M-4(CF_3_SO_3_^−^)-6(HCF_3_SO_3_)]^4+^ 1778.2383, found 1778.2319.

Synthesis and characterization of L^4–6^ can be seen in Supplementary Methods and Supplementary Figs. 11–28.

Self-assembly of L^2^ and L^3^ with lanthanide ions is carried out according to literature ^26,66^.

NMR and ESI-TOF-MS characterization of [Eu_1_L^2^_3_](CF_3_SO_3_)_3_, [La_2_L^3^_3_](ClO_4_)_6_, [Ce_2_L^3^_3_](CF_3_SO_3_)_6_, [Pr_2_L^3^_3_](ClO_4_)_6_, [Nd_2_L^3^_3_](ClO_4_)_6_, [Sm_2_L^3^_3_](ClO_4_)_6_, [Eu_2_L^3^_3_](ClO_4_)_6_ and [Pr_4_L^6^_4_](CF_3_SO_3_)_12_, can be seen in Supplementary Methods and Supplementary Figs. 73–89, 109–114, 220–226.

### General procedure for mixed-metal one-pot self-assembly of L^1,3,6^

L^1^ (6.0 μmol) was treated with an equimolar mixture of Ln^a^(ClO_4_)_3_•6H_2_O and Ln^b^(ClO_4_)_3_•6H_2_O (6.0 μmol of each) in CD_3_CN (0.6 mL) at 40 °C for 1 h and the turbid suspension of ligands gradually turned clear. The resulting yellow solution was characterized by ^1^H NMR and ESI-TOF-MS to identify the selectivity in the mixed-metal one-pot self-assembly process. No change in the ^1^H NMR spectra was observed after elongated reaction time for even 2 weeks, suggesting that the self-assembly is fast and thermodynamically stable (Supplementary Figs. 116–176, 240–279).

^1^H NMR spectra of the mixed-metal self-assembled complexes with non-absolute self-recognition behavior were measured with d1 value set as 20 s to ensure the accuracy of the metal ion selectivity, which was calculated based on ^1^H NMR integration.

Metal combinations with rather small ionic difference were avoided, such as Sm^III^/Eu^III^, which leads to the formation of an intricate mixture of complexes with low symmetry as a result of poor metal-ion selectivity, making it difficult for the identification of selectivity through ^1^H NMR integration.

The selectivity for smaller sized Ln^III^ in incomplete self-sorting mixed-metal self-assembly process is determined by ^1^H NMR. The highly symmetrical ^1^H NMR patterns excluded the formation of a dynamic mixture of scrambled-metal cages. ESI-TOF-MS analyses further confirmed the formation of trace amounts of mono substituted (Ln_1_^a^Ln^b^_3_L^1^_4_) cage, along with homometallic cages. As the chemical shifts of the signals arising from the tetrahedral assemblies are manifestation of magnetic environments imposed by the coordinated paramagnetic Ln^III^, integration of two sets of NMR signals can be used for quantification of two kinds of Ln^III^ vertices (Supplementary Figs. 242–274).

Cd^II^/Ln^III^ or Ca^II^/Ln^III^ mixed-metal one-pot self-assembly with L^1^ was implemented in a similar procedure as above. As ligand L^1^ has much higher self-assembly preference to Ln^III^ ions with smaller ionic radii along the lanthanide series, mixed-metal self-assembly of Cd^II^/Ln^III^ and Ca^II^/Ln^III^ were only proceeded for La^III^, which has smaller association constant than other lanthanide ions and complete mixed-metal self-sorting assembly of Cd^II^/Ln^III^ and Ca^II^/Ln^III^ was thus speculated (Supplementary Figs. 140–141 and 240–241).

### Nonlinear curve fitting of simulated isotope patterns of La^III^-Ce^III^ mixed-metal self-assembly complexes with L^1^

Considering the tiny difference in the ESI­ response factors of the tetrahedral complexes of different lanthanide ions, nonlinear curve fitting using the model of *x*[Ce_4_L^1^_4_]^12+^ and (1-*x*)[La_1_Ce_3_L^1^_4_]^12+^ fits well with the observed mass spectrum, with the composition of [Ce_4_L^1^_4_]^12+^/[La_1_Ce_3_L^1^_4_]^12+^ in the La^III^-Ce^III^ mixed-metal self-assembly complexes as 0.61/0.39 and 0.62/0.38 for 5 +  and 6 +  peaks, respectively. This means about ten percent of La^III^ is incorporated in the mixed-metal complexes, which agrees well with ^1^H NMR analysis of 10.7 percent La^III^. Similar fitting using the model of *x*[Ce_4_L^1^_4_]^12+^ and (1-*x*)[La_4_L^1^_4_]^12+^ did not give consistent results with the observed isotope patterns of the 5 +  and 6 + peaks (Supplementary Figs. 242–243).

### General procedure for mixed-metal one-pot self-assembly of L^3^

L^3R^ or L^3S^ (4.5 μmol) was treated with an equimolar mixture of Ln^a^(ClO_4_)_3_•6H_2_O and Ln^b^(ClO_4_)_3_•6H_2_O (3.0 μmol of each) in CD_3_CN (0.6 mL) at 40 °C for 1 h and the turbid suspension of ligands gradually turned clear. The resulting yellow solution was characterized by ^1^H NMR and ESI-TOF-MS to identify the selectivity in the mixed-metal one-pot self-assembly process. No change in the ^1^H NMR spectra was observed after elongated reaction time for even 1 month, suggesting the self-assembly is fast and thermodynamically stable (Supplementary Figs. 177–188 and 280–288).

### General procedure for mixed-metal one-pot self-assembly of L^6^

L^6^ (6.0 μmol) was treated with an equimolar mixture of Ln^a^(CF_3_SO_3_)_3_ and Ln^b^(CF_3_SO_3_)_3_ (6.0 μmol of each) in CD_3_CN/CDCl_3_ (0.6 mL) at room temperature for 1 h. The resulting yellow solution was characterized by ^1^H NMR and ESI-TOF-MS to identify the selectivity in the mixed-metal one-pot self-assembly process. No change in the ^1^H NMR spectra was observed after elongated reaction time for even 1 month, suggesting the self-assembly is fast and thermodynamically stable (Supplementary Figs. 189–194 and 289–294).

### General procedure for one-pot tri-metallic mixed-metal self-assembly of L^1^

L^1R^ or L^1S^ (6.0 μmol) was treated with a mixture of La(ClO_4_)_3_•6H_2_O, Pr(ClO_4_)_3_•6H_2_O and Eu(ClO_4_)_3_•6H_2_O (6.0 μmol of each) in CD_3_CN (0.6 mL) at 40 °C for 1 h and the turbid suspension of ligands gradually turned clear. The resulting yellow solution was characterized by ^1^H NMR and ESI-TOF-MS, which suggests a comparable self-assembly selectivity to that of two-component mixed-metal one-pot self-assembly process (Supplementary Figs. 196 and 274).

Ca^II^/Cd^II^/Ln^III^ (ClO_4_^−^ as the counter ions) tri-metallic one-pot mixed-metal self-assembly of L^1^ was implemented in the similar procedure as above, and complete metal ion self-recognition was observed as ascertained by ^1^H NMR and ESI-TOF-MS (Supplementary Figs. 195 and 273).

### General procedure for post-synthetic metal-ion metathesis experiments

Self-assembled complexes [Ln^a^_4_L^1^_4_](ClO_4_)_12_ (1.5 μmol) in CD_3_CN were prepared in advance, followed by the addition of Ln^b^(ClO_4_)_3_•6H_2_O (6.0 μmol), resulting in a total volume of CD_3_CN of 0.6 mL. ^1^H NMR spectra were measured immediately after the addition of the second Ln^b^(III) at room temperature until the mixture reached the final thermodynamically stable state. The highly split ^1^H NMR signals and ESI-TOF-MS spectroscopy indicate the dynamic formation of multiple scrambled-metal cages [(Ln^a^_*n*_Ln^b^_4-*n*_)L^1^_4_]^12+^ (*n* = 0–4) during the post-synthetic metal-ion metathesis experiments, which further indicates the metal-metathesis on four metal vertices of tetrahedral cages [Ln^a^_4_L^1^_4_](ClO_4_)_12_ proceed stepwise. It is worth pointing out that the substitution rate depends on the difference of their ionic radius in each combination. With larger ionic radii difference, the substitution proceeds much faster and vice versa. As for La(III)/Ce(III) metal combination, the one-step substitution leading to the [La_1_Ce_3_L^1^_4_] species proceed so fast that no metathesis intermediates were observed (Supplementary Figs. 197–203 and 295).

Post-synthetic metal-ion metathesis experiments of L^3^ were performed in the same method as that for L^1^ (Supplementary Figs. 204–208).

### Single-crystal X-ray diffraction studies

The X-ray diffraction studies for complex Cd_4_L^1^_4_(ClO_4_)_8_ and La_4_L^1^_4_(ClO_4_)_12_ were carried out at the BL17B macromolecular crystallography beamline in Shanghai Synchrotron Radiation Facility. The collected diffraction data were processed with the HKL 3000 software program^66^. The structures were solved by direct methods and refined by full-matrix least-squares on *F*^*2*^ with anisotropic displacement using the SHELX software package^67^. The crystals diffract only very weakly due to large amounts of solvent molecules and anions. For the structure of Cd_4_L^1^_4_(ClO_4_)_8_, where counter ions and solvent molecules were so highly disordered that they could not be reasonably located, the residual intensities were removed by PLATON/SQUEEZE routine^55^. Still one A-alert and some B-alerts are found by the (IUCr) check CIF routine, all of which are due to the poor diffraction nature of the crystals. Details on crystal data collection and refinement are summarized in Supplementary Tables 7 and 8. Additional comments on the crystallographic works are also available in the Supplementary Methods.

### Investigation of thermodynamic stability of different lanthanide complexes

Post-synthetic metal-metathesis experiments of Ln^b^^III^ toward [Ln^a^_4_L^1^_4_]^12+^ complexes in CH_3_CN were identified to proceed in a stepwise mode, with only [Ln^a^_1_La^b^_3_L^1^_4_]^12+^ and [Ln^b^_4_L^1^_4_]^12+^ complexes observed in the thermodynamic equilibrium state (for some metal combinations), as confirmed by ^1^H NMR and ESI-TOF-MS. As for La^III^/Ce^III^ combination, the displacement process is shown as below:

1 $${\mathrm{Ce}} + {\mathrm{La}}_4{\mathrm{L}}_4^1 \rightleftharpoons {\mathrm{La}}_3{\mathrm{Ce}}_1{\mathrm{L}}_4^1 + {\mathrm{La}}\ \qquad {\mathrm \it {K }}_1 = \frac{{\left[ {{\mathrm{La}}_3{\mathrm{Ce}}_1{\mathrm{L}}_4^1} \right]\left[ {{\mathrm{La}}} \right]}}{{\left[ {{\mathrm{La}}_4{\mathrm{L}}_4^1} \right]\left[ {{\mathrm{Ce}}} \right]}}$$

2 $${\mathrm{Ce}} + {\mathrm{La}}_3{\mathrm{Ce}}_1{\mathrm{L}}_4^1 \ \rightleftharpoons {\mathrm{La}}_2{\mathrm{Ce}}_2{\mathrm{L}}_4^1 + {\mathrm{La}}\ \quad {\mathrm{\it K }}_2 = \frac{{\left[ {{\mathrm{La}}_2{\mathrm{Ce}}_2{\mathrm{L}}_4^1} \right]\left[ {{\mathrm{La}}} \right]}}{{\left[ {{\mathrm{La}}_3{\mathrm{Ce}}_1{\mathrm{L}}_4^1} \right]\left[ {{\mathrm{Ce}}} \right]}}$$

3 $${\mathrm{Ce}} + {\mathrm{La}}_2{\mathrm{Ce}}_2{\mathrm{L}}_4^1 \ \rightleftharpoons {\mathrm{La}}_1{\mathrm{Ce}}_3{\mathrm{L}}_4^1 + {\mathrm{La}}\ \quad {\mathrm{\it K }}_3 = \frac{{\left[ {{\mathrm{La}}_1{\mathrm{Ce}}_3{\mathrm{L}}_4^1} \right]\left[ {{\mathrm{La}}} \right]}}{{\left[ {{\mathrm{La}}_2{\mathrm{Ce}}_2{\mathrm{L}}_4^1} \right]\left[ {{\mathrm{Ce}}} \right]}}$$

4 $${\mathrm{Ce}} + {\mathrm{La}}_1{\mathrm{Ce}}_3{\mathrm{L}}_4^1 \ \rightleftharpoons {\mathrm{Ce}}_4{\mathrm{L}}_4^1 + {\mathrm{La}}\ \qquad {\mathrm{\it K }}_4 = \frac{{\left[ {{\mathrm{Ce}}_4{\mathrm{L}}_4^1} \right]\left[ {{\mathrm{La}}} \right]}}{{\left[ {{\mathrm{La}}_1{\mathrm{Ce}}_3{\mathrm{L}}_4^1} \right]\left[ {{\mathrm{Ce}}} \right]}}$$

5 $${\rm Cumulative}\ {\rm stability}\ {\rm constant}:{\mathrm{\beta }}_4 = {\mathrm{\it K }}_1{\mathrm{\it K }}_2{\mathrm{\it K }}_3{\mathrm{\it K }}_4 = \frac{{\left[ {{\mathrm{Ce}}_4{\mathrm{L}}_4^1} \right]\left[ {{\mathrm{La}}} \right]^4}}{{\left[ {{\mathrm{La}}_4{\mathrm{L}}_4^1} \right]\left[ {{\mathrm{Ce}}} \right]^4}}$$

$$\begin{array}{l}\left[ {{\mathrm{La}}_4{\mathrm{L}}_4^1} \right] \ll \left[ {{\mathrm{La}}_1{\mathrm{Ce}}_3{\mathrm{L}}_4^1} \right] \to {\mathrm{\beta }_4} = \frac{{\left[ {{\mathrm{Ce}}_4{\mathrm{L}}_4^1} \right]\left[ {{\mathrm{La}}} \right]^4}}{{\left[ {{\mathrm{La}}_4{\mathrm{L}}_4^1} \right]\left[ {{\mathrm{Ce}}} \right]^4}} \gg \frac{{\left[ {{\mathrm{Ce}}_4{\mathrm{L}}_4^1} \right]\left[ {{\mathrm{La}}} \right]^4}}{{\left[ {{\mathrm{La}}_1{\mathrm{Ce}}_3{\mathrm{L}}_4^1} \right]\left[ {{\mathrm{Ce}}} \right]^4}} = {\mathrm{\beta }}_{{\mathrm{La}}/{\mathrm{Ce}}}\end{array}$$

According to ^1^H NMR and ESI-TOF-MS analyses, the concentration of [La_4_L^1^_4_]^12+^ is much smaller than [La_1_Ce_3_L^1^_4_]^12+^. So the minimum of *β*_Ce/La_ was estimated by replacing [La_4_L^1^_4_] in Equation (5) with [La_1_Ce_3_L^1^_4_], which can be defined by NMR integrations, to simplify the cumulative stability constant.

Considering that the cumulative stability constant *β*_4_ in the metathesis experiments can be regarded as relative stability constant of [Ce_4_L^1^_4_]^12+^ toward [La_4_L^1^_4_]^12+^, *β*_Ce/La_ can be used for qualitative analysis and comprehensive comparison on stability of different [Ln_4_L^1^_4_]^12+^ complexes (Supplementary Tables  4 and 5).

### Data availability

X-ray crystal structures of compounds La_4_L^1^_4_ and Cd_4_L^1^_4_ reported in this paper have been deposited in the Cambridge Crystallographic Data Center under accession numbers CCDC: 1532086 and 1532087, respectively. These data can be obtained free of charge via http://www.ccdc.cam.ac.uk/data_request/cif). All other data supporting the findings of this study are available in the article and its Supplementary Information files and from the corresponding authors on request.

## Electronic supplementary material

Supplementary Information

Peer Review File

Description of Additional Supplementary Files

Supplementary Data 1

Supplementary Data 2
