# Supplementary material for: A supramolecular lanthanide separation approach based on multivalent cooperative enhancement of metal ion selectivity
Source: Nat Commun. 2018 Feb 7;9:547. doi: 10.1038/s41467-018-02940-7 (PMC5803205; doi:10.1038/s41467-018-02940-7)
Supplement: Supplementary file 1 — Supplementary Information [file 41467_2018_2940_MOESM1_ESM.pdf]

A Supramolecular Lanthanide Separation Approach Based on Multivalent Cooperative Enhancement of Metal Ion Selectivity

Li et al.

Supplementary Figures

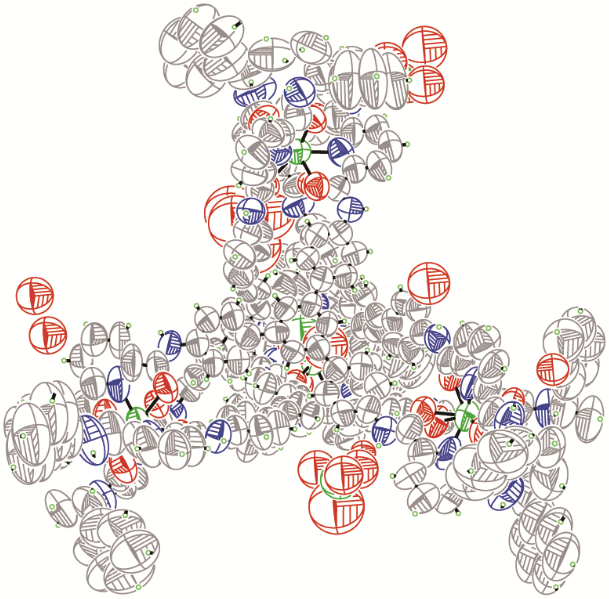

Supplementary Figure 1. Ortep drawing of the crystal structure of $\mathbf{Cd_4L^1_4}$ (ClO_4^- salt) at 50% probability leval.

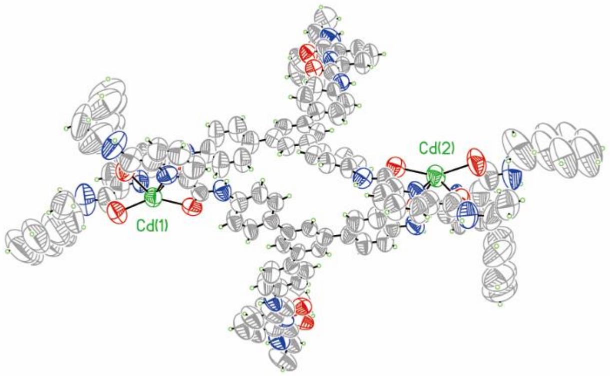

Supplementary Figure 2. ORTEP drawing of the individual metal centres and ligands in the asymmetrical unit of $\mathbf{Cd_4L^1_4}$ at 30% ellipsoids level.

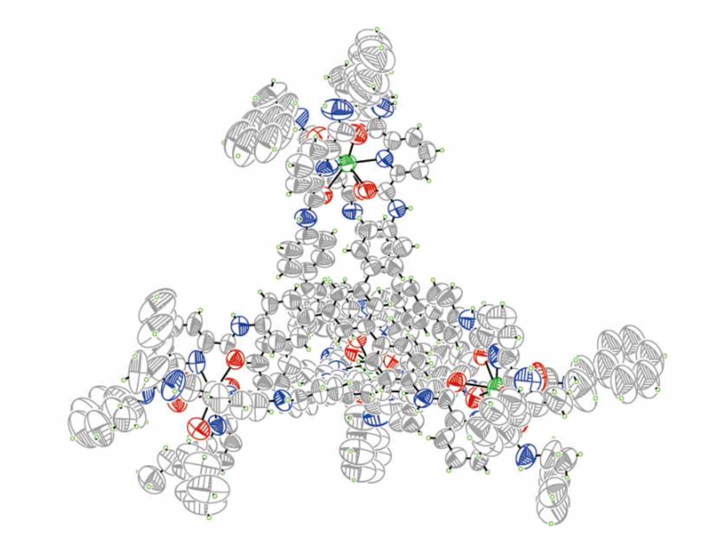

Supplementary Figure 3. ORTEP drawing of the $\mathbf{Cd_4L^1_4}$ tetrahedral complex at 30% ellipsoids level.

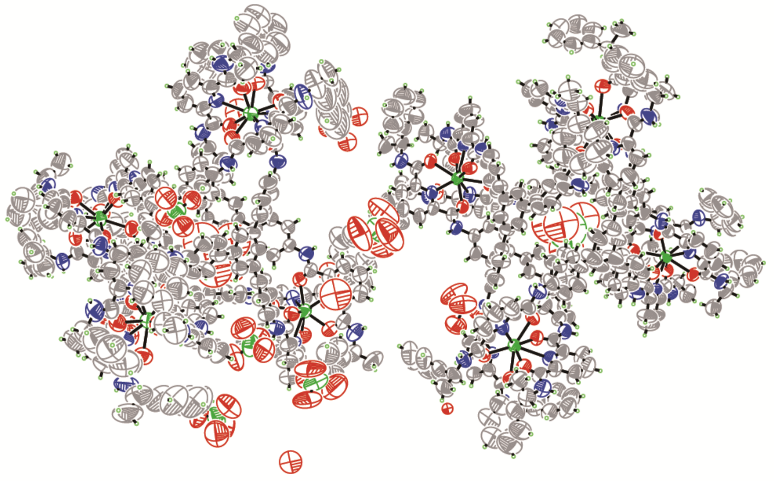

Supplementary Figure 4. Ortep drawing of the crystal structure of $\mathbf{La_4L^1_4}$ (ClO_4^- salt) at 50% probability leval.

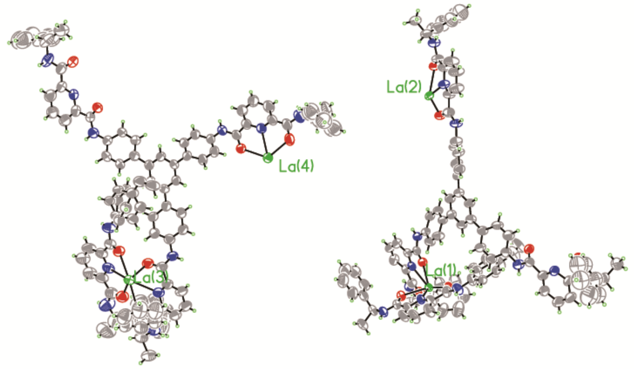

Supplementary Figure 5. ORTEP drawing of the individual metal centres and ligands in the asymmetrical unit of $\mathbf{La_4L^1_4}$ at 30% ellipsoids level.

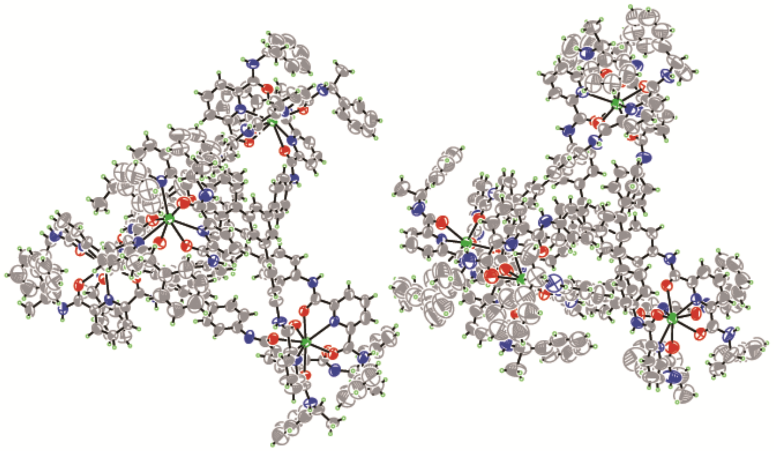

Supplementary Figure 6. ORTEP drawing of the $\mathbf{La_4L^1_4}$ tetrahedral complex at 30% ellipsoids level.

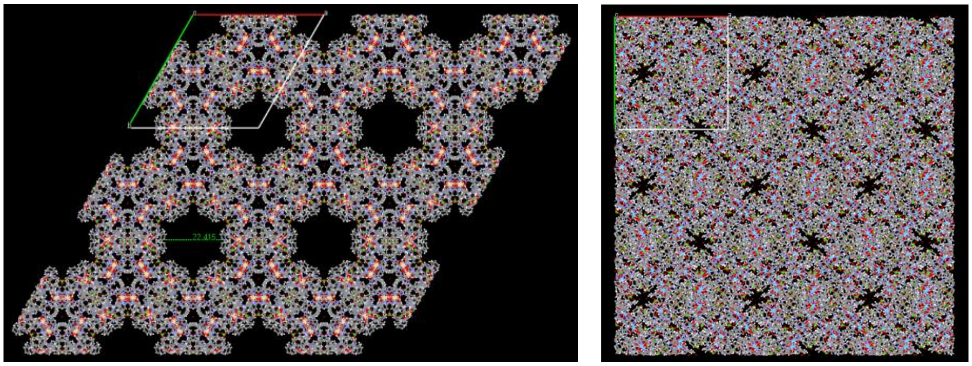

Supplementary Figure 7. Different crystal packing diagrams for the $\mathbf{Cd_4(L^1)_4}$ (left) and $\mathbf{La_4(L^1_4)}$ (right) tetrahedral cages viewing along the *c* axes.

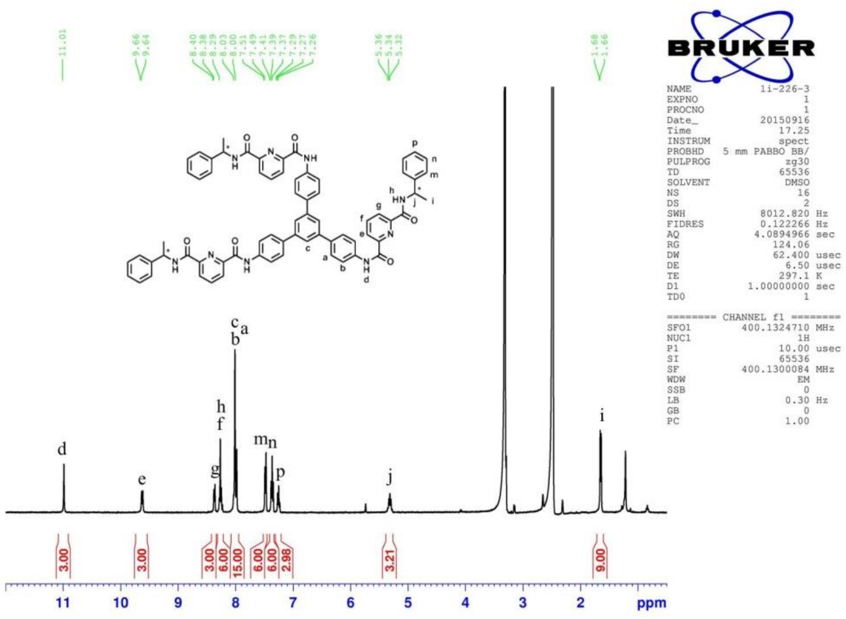

Supplementary Figure 8. ^{1}H NMR spectrum of $\mathbf{L^{1S}}$ (DMSO, 400 MHz, 298K).

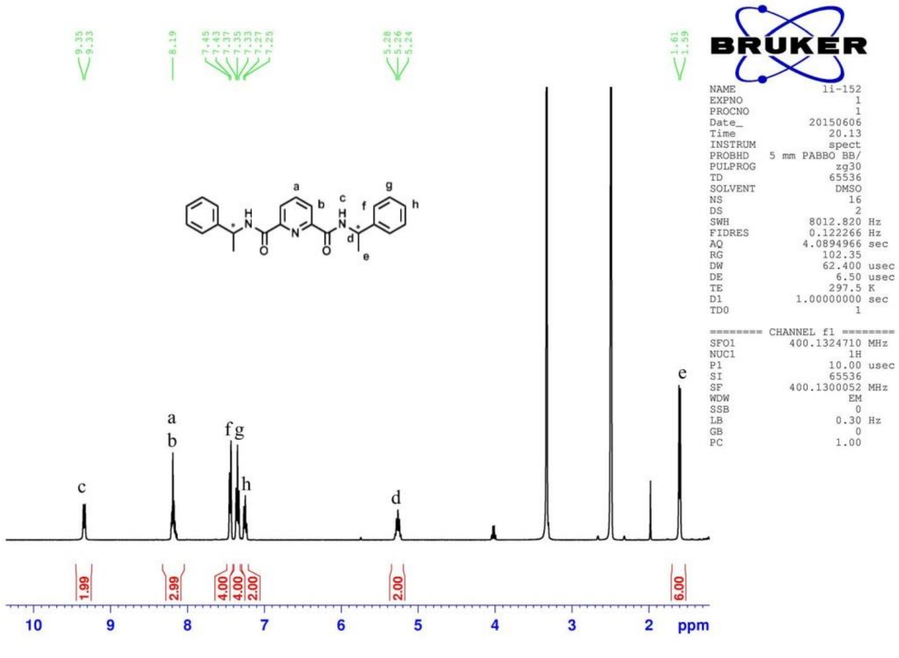

Supplementary Figure 9. ^{1}H NMR spectrum of $\mathbf{L^{2S}}$ (DMSO, 400 MHz, 298 K).

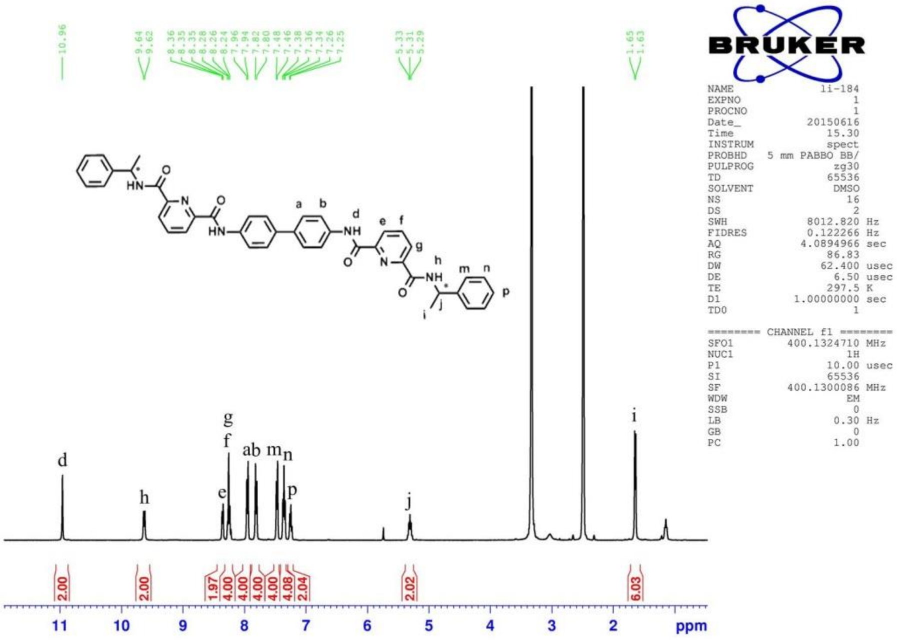

Supplementary Figure 10. ^{1}H NMR spectrum of $\mathbf{L^{3S}}$ (DMSO, 400 MHz, 298 K).

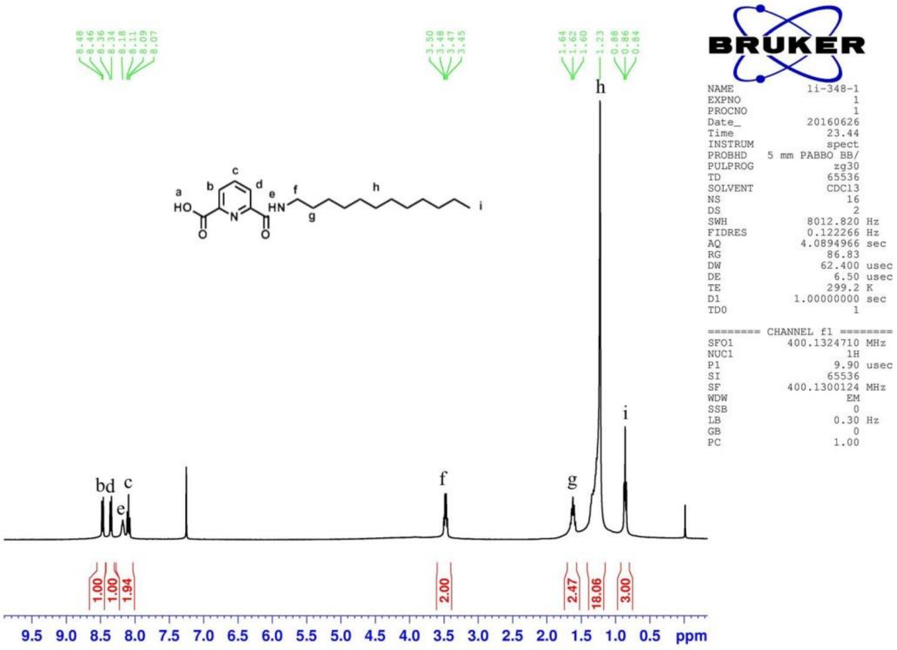

Supplementary Figure 11. ^{1}H NMR spectrum of **4** ($CDCl_3$, 400 MHz, 298 K).

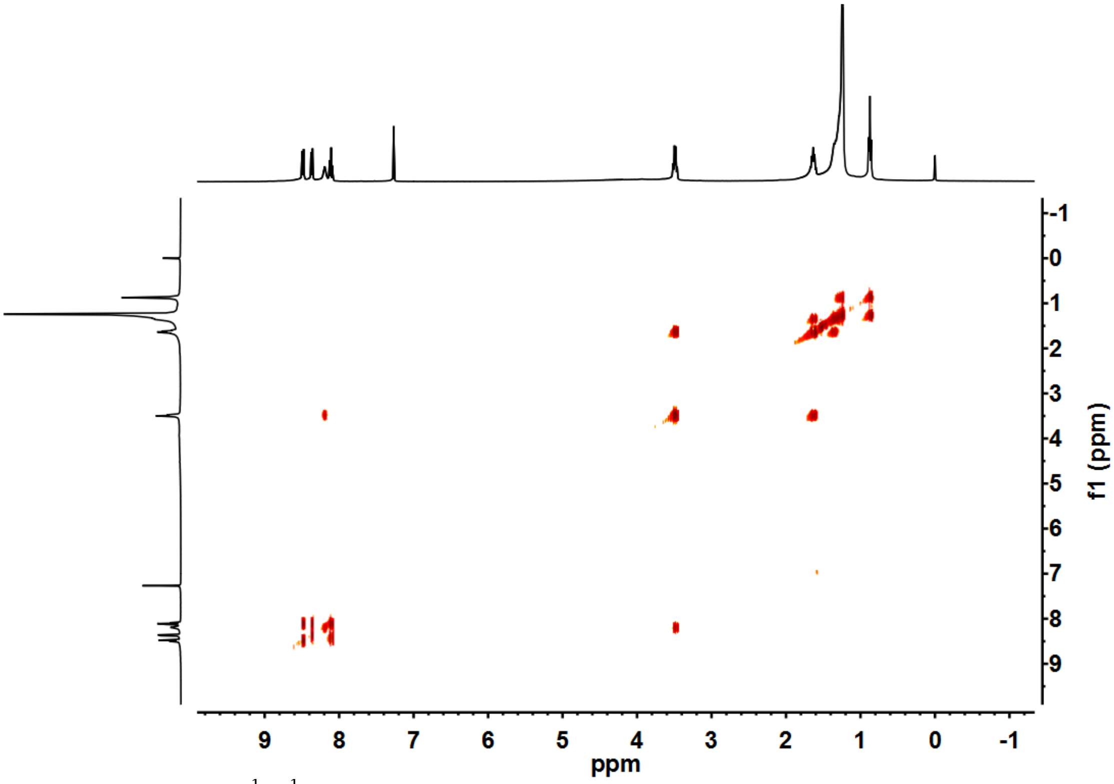

Supplementary Figure 12. ^{1}H-^{1}H COSY NMR spectrum of **4** ($CDCl_3$, 400 MHz, 298 K).

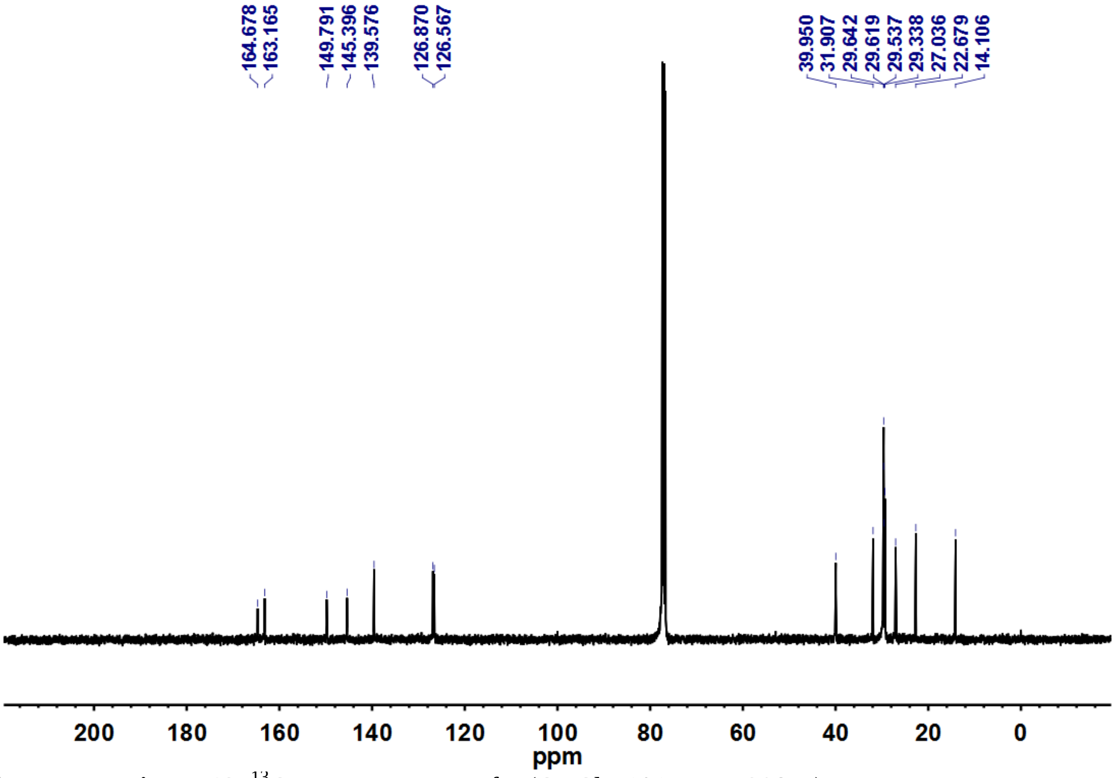

Supplementary Figure 13. ^{13}C NMR spectrum of **4** ($CDCl_3$, 101 MHz, 298 K).

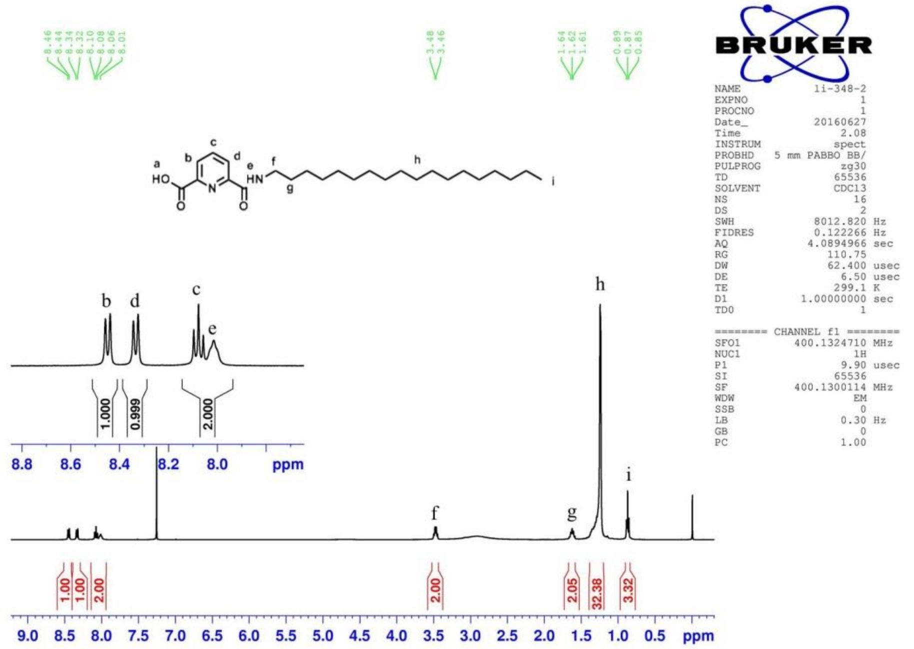

Supplementary Figure 14. ^{1}H NMR spectrum of **5** ($CDCl_3$, 400 MHz, 298 K).

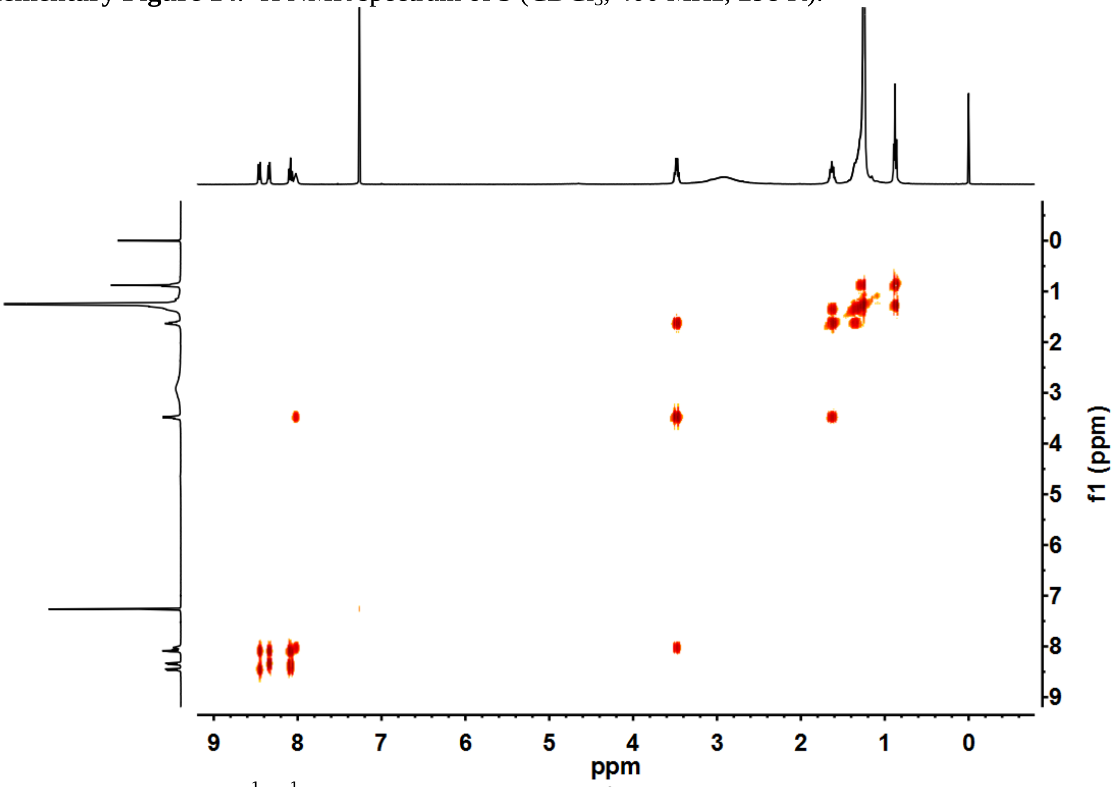

Supplementary Figure 15. ^{1}H-^{1}H COSY NMR spectrum of **5** ($CDCl_3$, 400 MHz, 298 K).

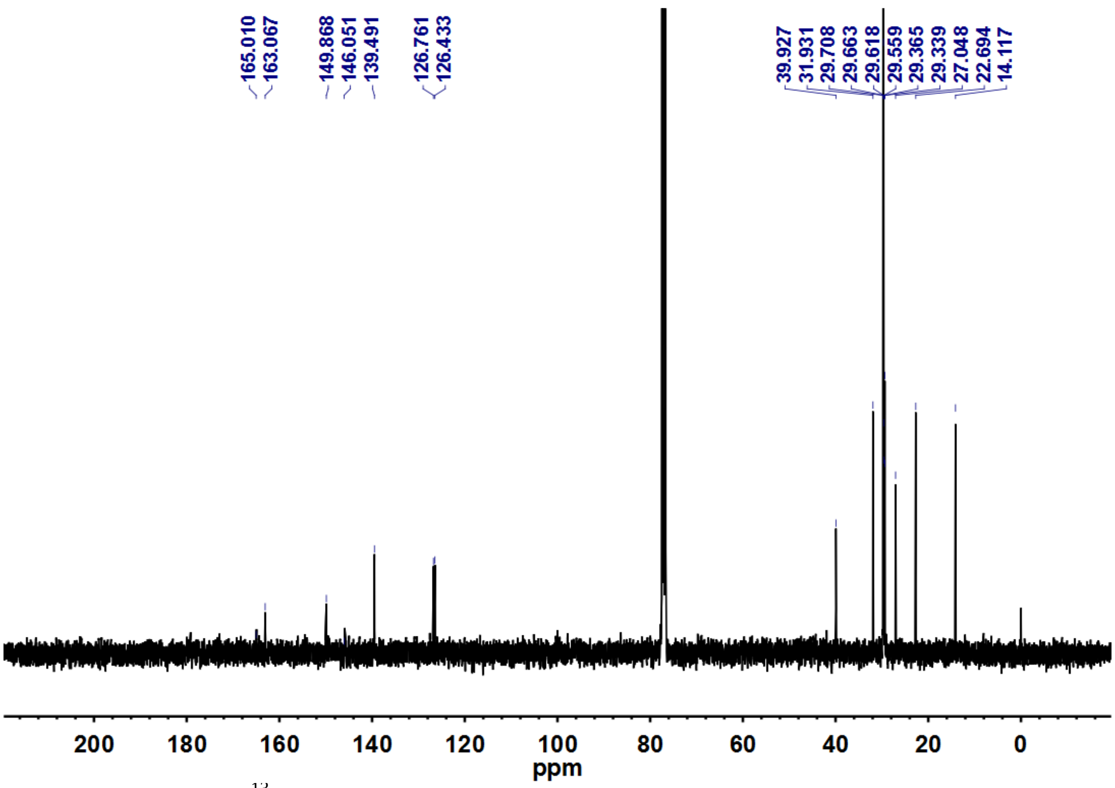

Supplementary Figure 16. ^{13}C NMR spectrum of **5** ($CDCl_3$, 101 MHz, 298 K).

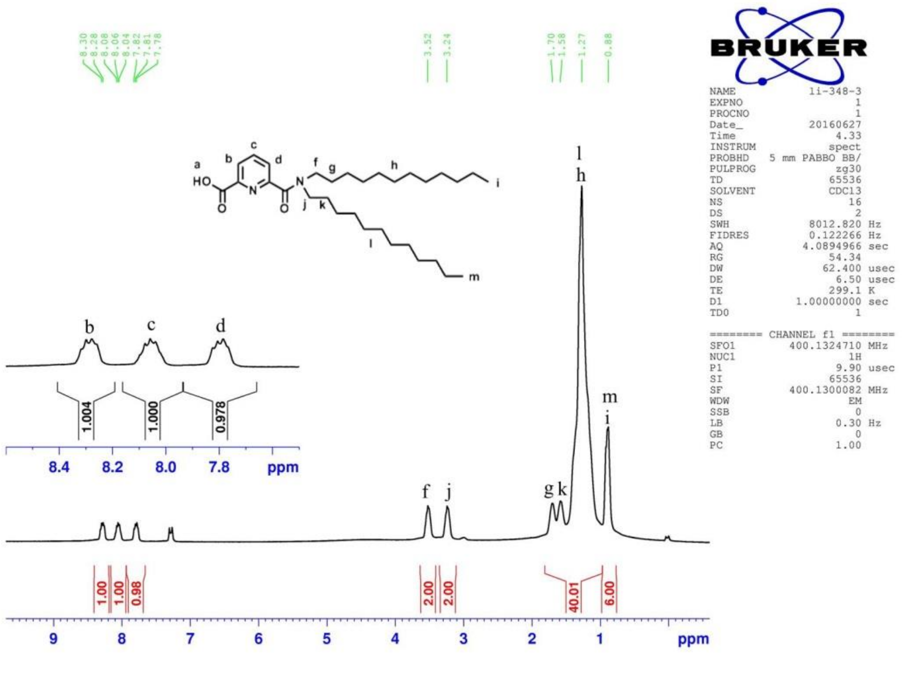

Supplementary Figure 17. ^{1}H NMR spectrum of **6** ($CDCl_3$, 400 MHz, 298 K).

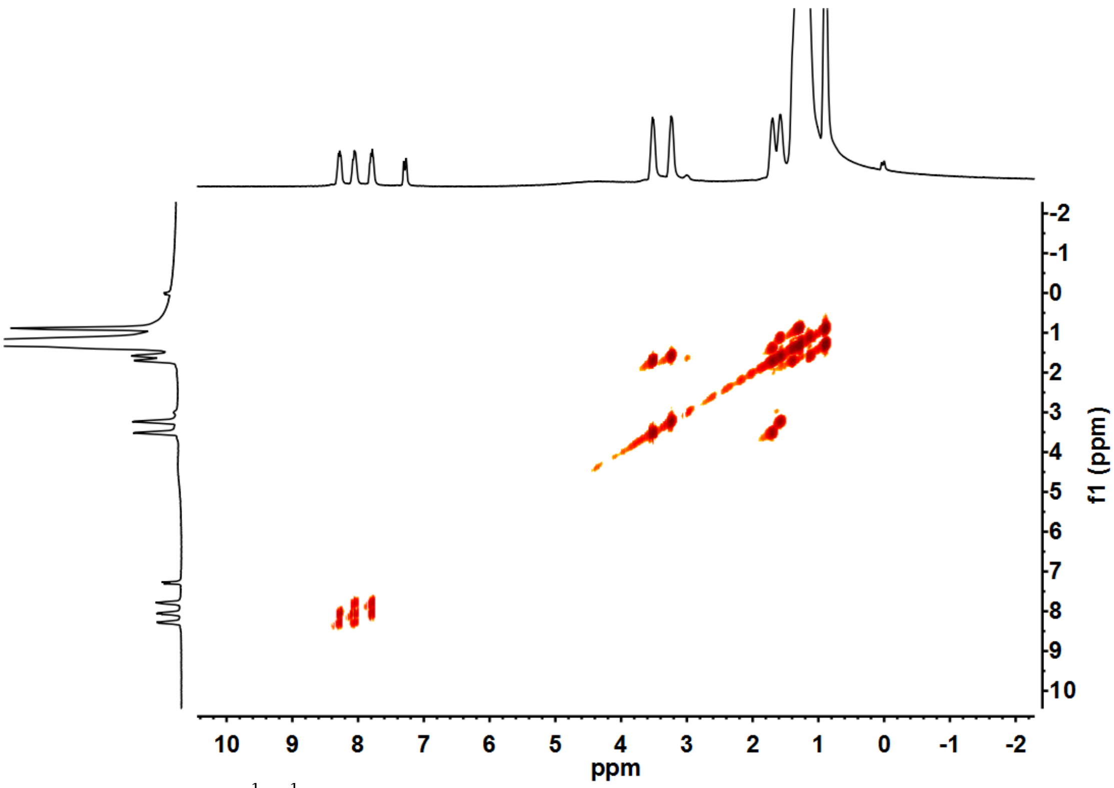

Supplementary Figure 18. ^{1}H-^{1}H COSY NMR spectrum of **6** ($CDCl_3$, 400 MHz, 298 K).

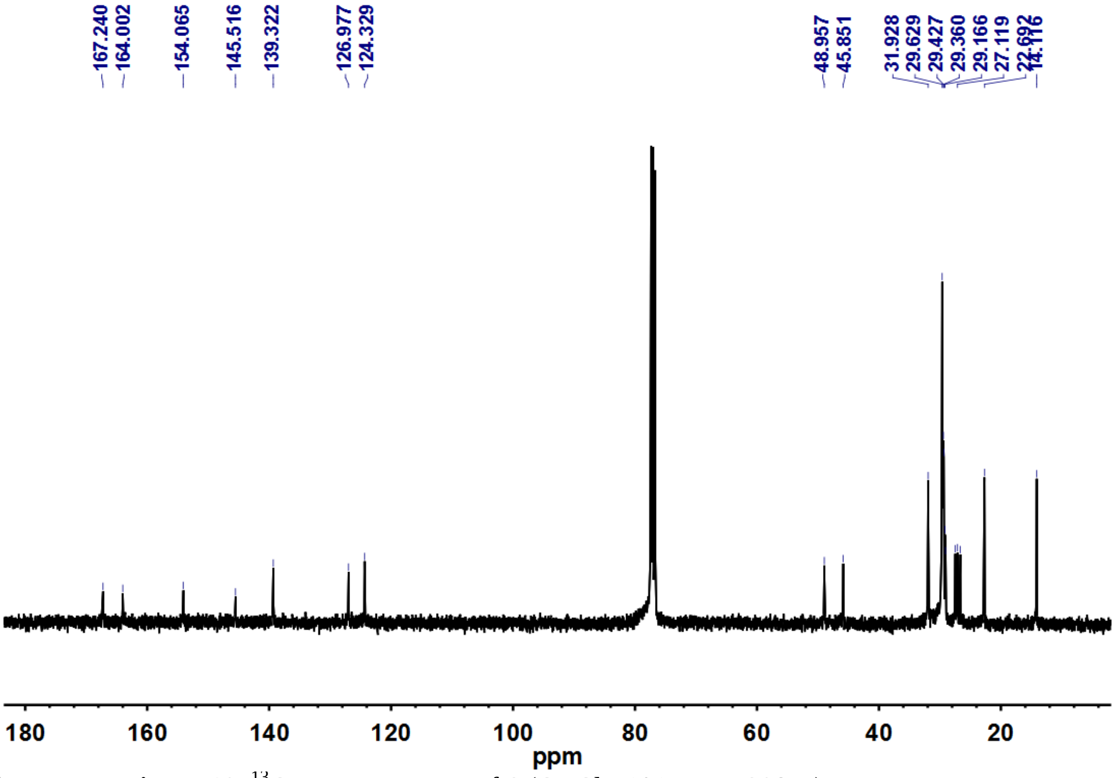

Supplementary Figure 19. ^{13}C NMR spectrum of **6** ($CDCl_3$, 101 MHz, 298 K).

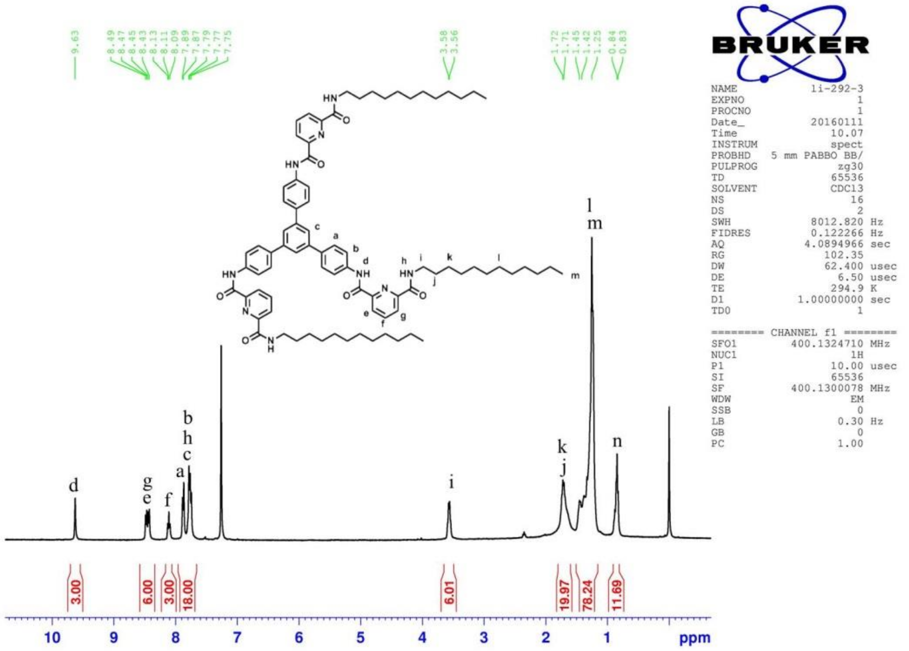

Supplementary Figure 20. ^{1}H NMR spectrum of $\mathbf{L^4}$ ($CDCl_3$, 400 MHz, 298 K).

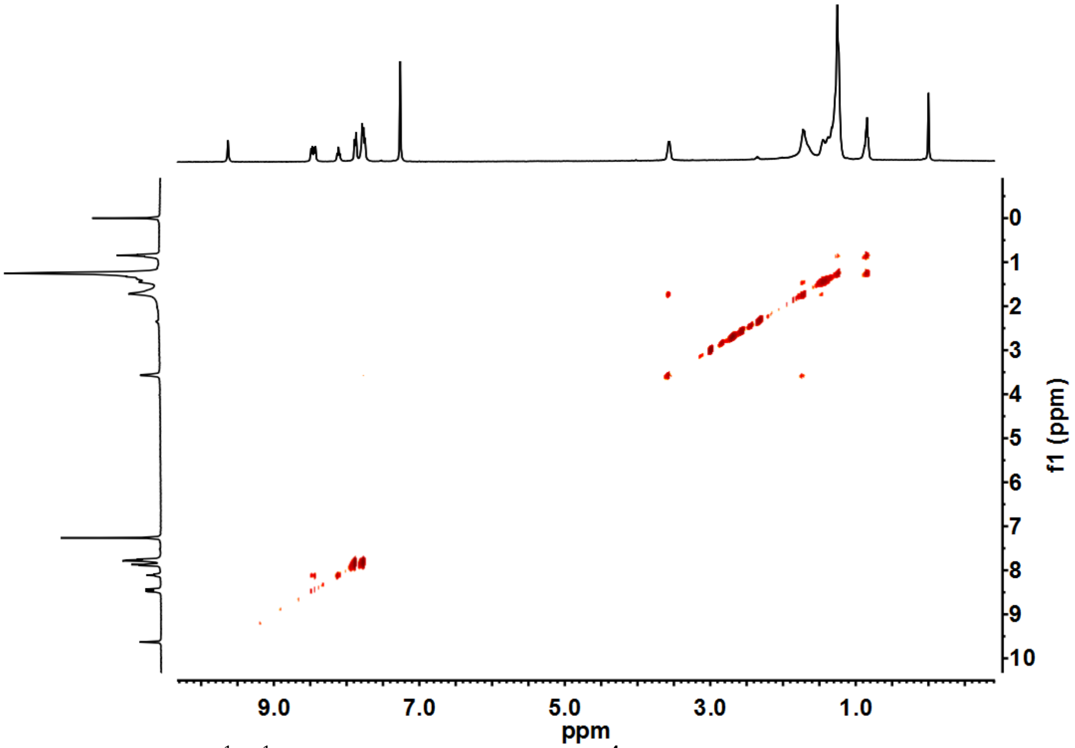

Supplementary Figure 21. ^{1}H-^{1}H COSY NMR spectrum of $\mathbf{L^4}$ ($CDCl_3$, 400 MHz, 298 K).

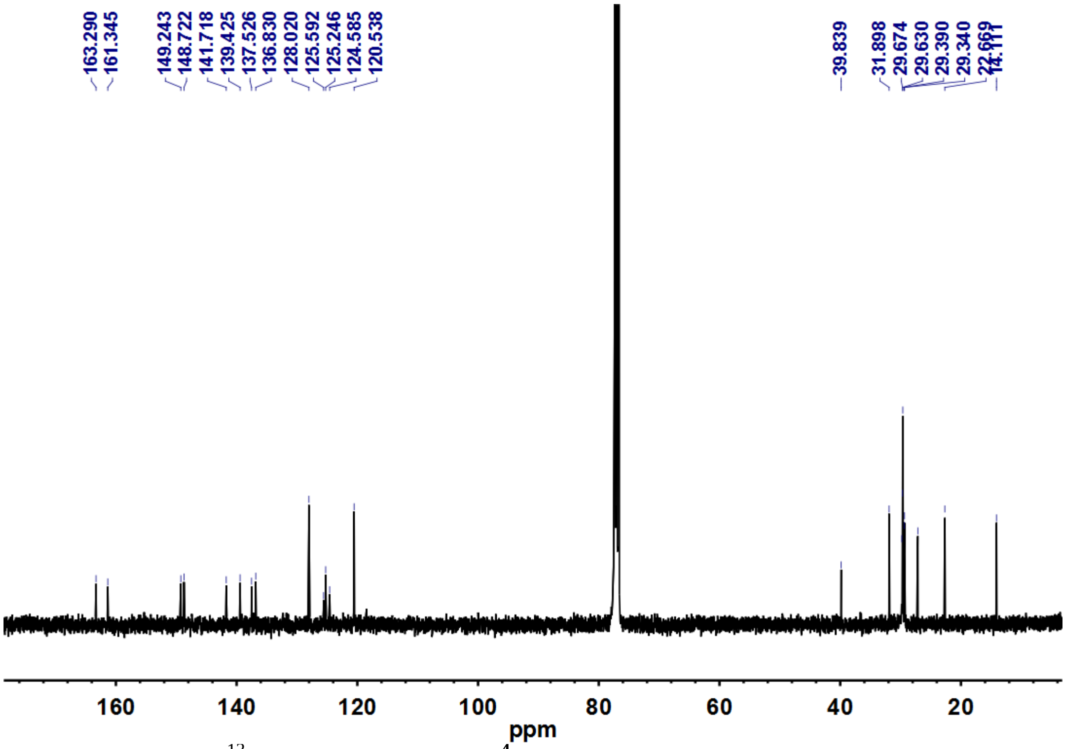

Supplementary Figure 22. ^{13}C NMR spectrum of $\mathbf{L^4}$ ($CDCl_3$, 101 MHz, 298 K).

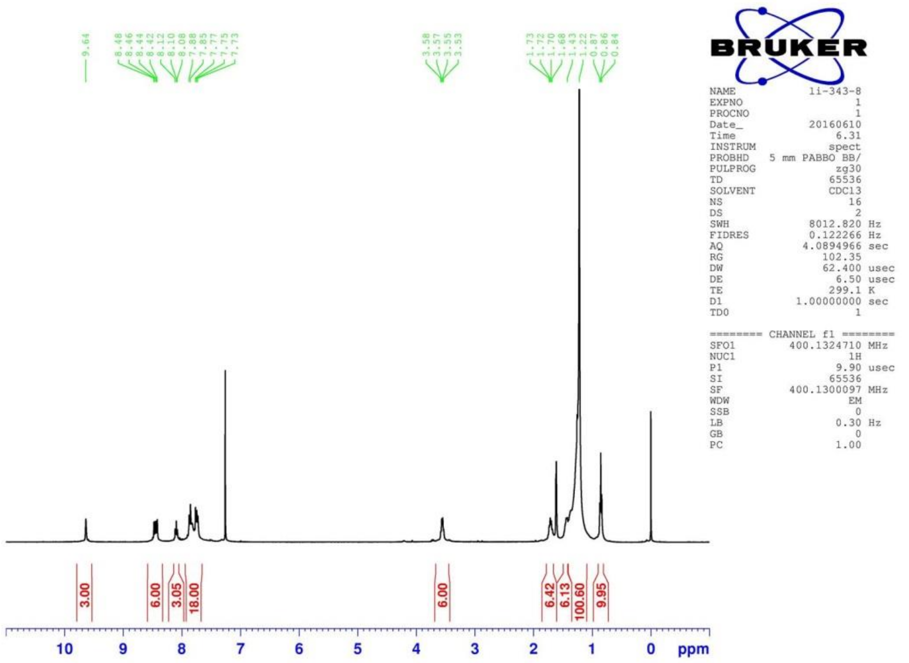

Supplementary Figure 23. ^{1}H NMR spectrum of $\mathbf{L^5}$ ($CDCl_3$, 400 MHz, 298 K).

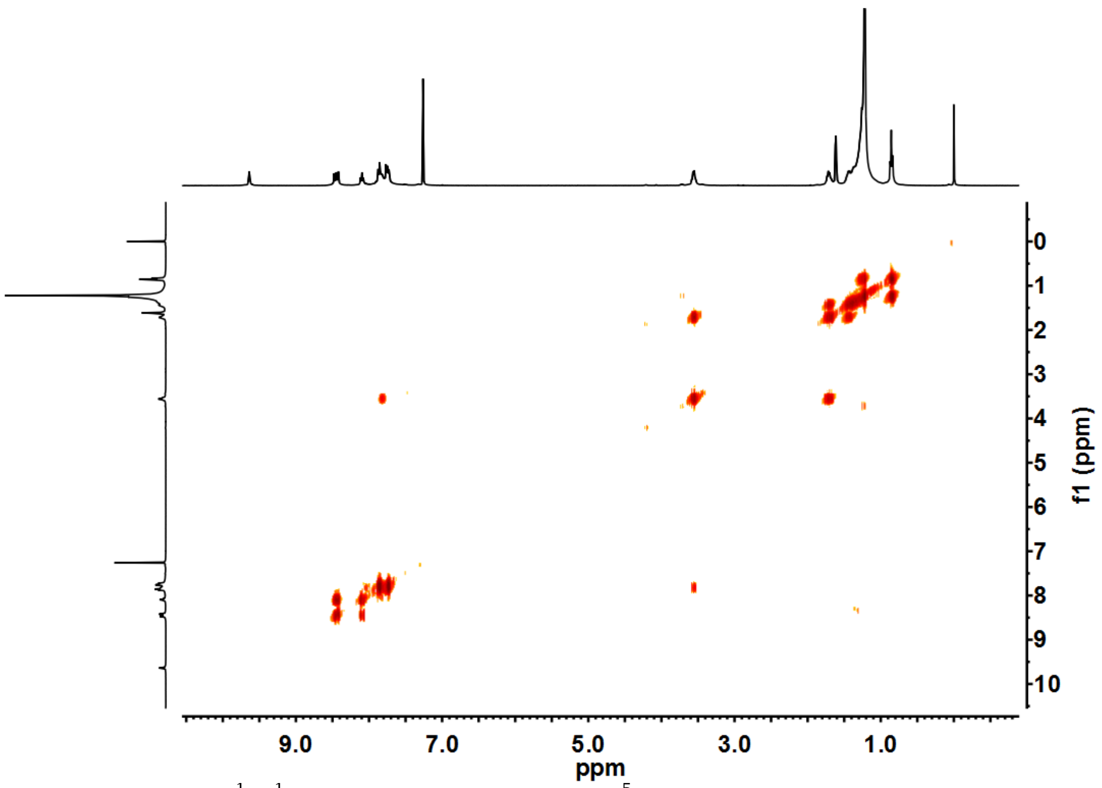

Supplementary Figure 24. ^{1}H-^{1}H COSY NMR spectrum of $\mathbf{L^5}$ ($CDCl_3$, 400 MHz, 298 K).

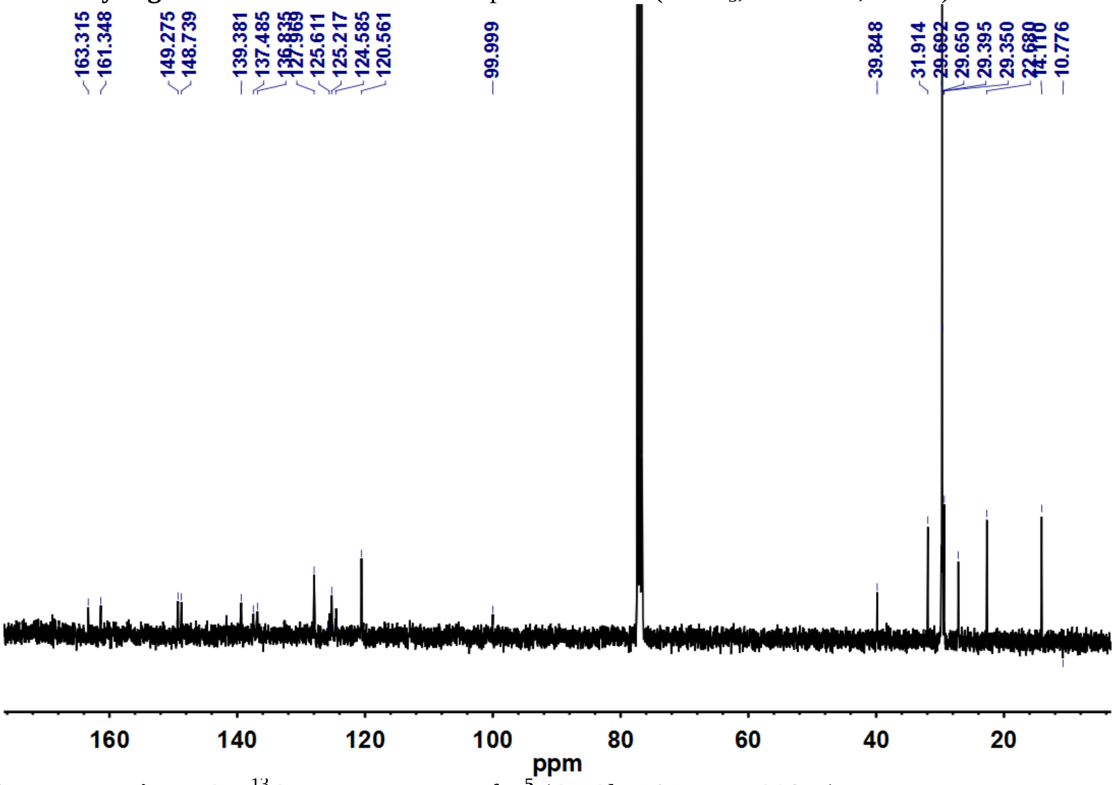

Supplementary Figure 25. ^{13}C NMR spectrum of $\mathbf{L^5}$ ($CDCl_3$, 101 MHz, 298 K).

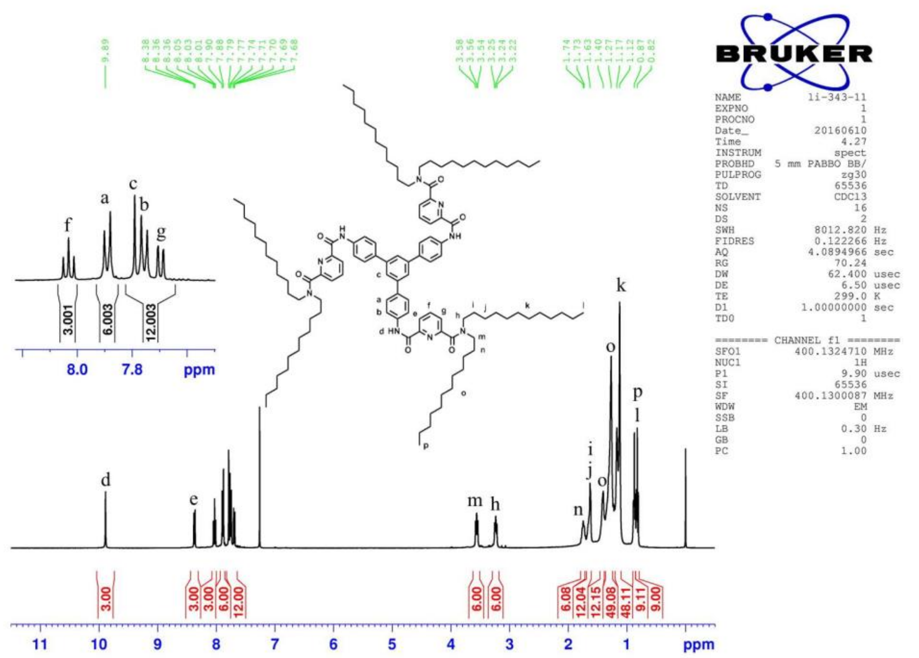

Supplementary Figure 26. ^{1}H NMR spectrum of $\mathbf{L^6}$ ($CDCl_3$, 400 MHz, 298 K).

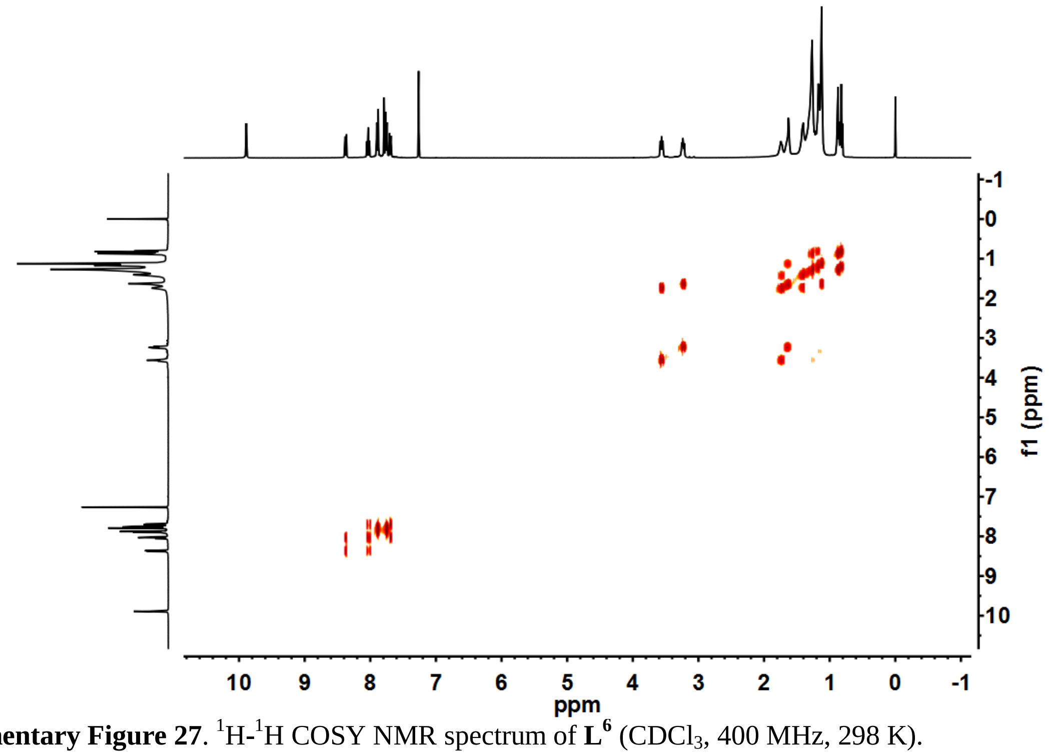

Supplementary Figure 27. ^{1}H-^{1}H COSY NMR spectrum of $\mathbf{L^6}$ ($CDCl_3$, 400 MHz, 298 K).

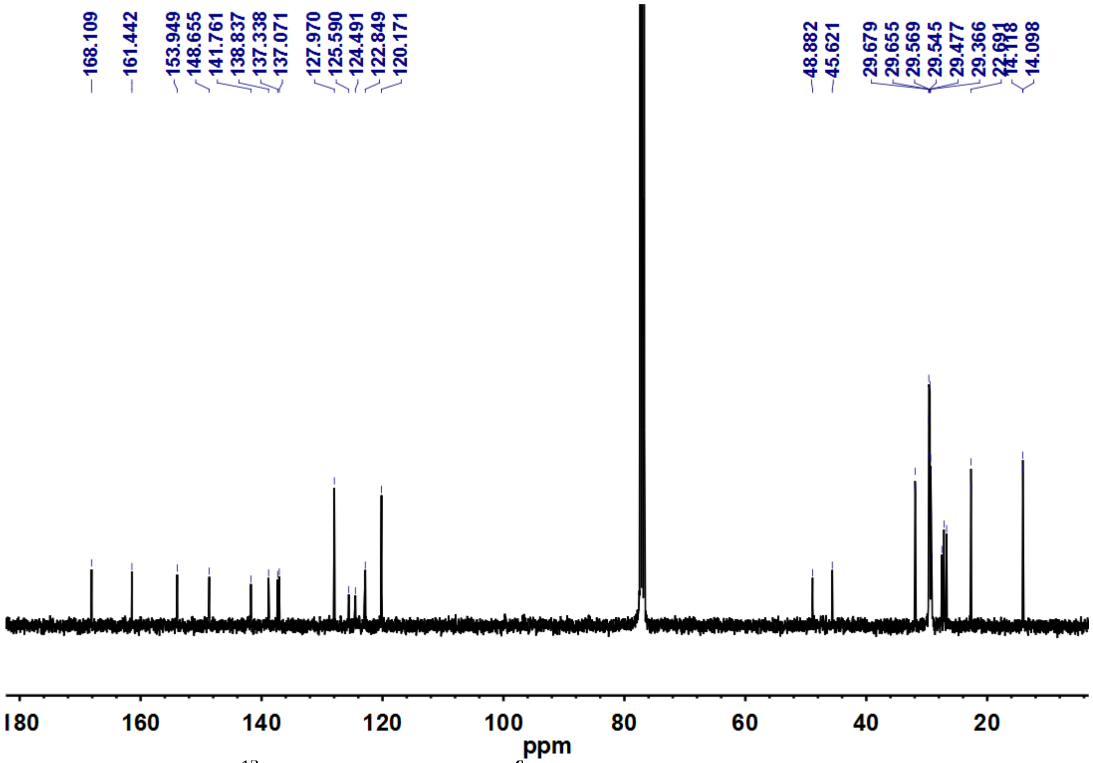

Supplementary Figure 28. ^{13}C NMR spectrum of $\mathbf{L^6}$ ($CDCl_3$, 101 MHz, 298 K).

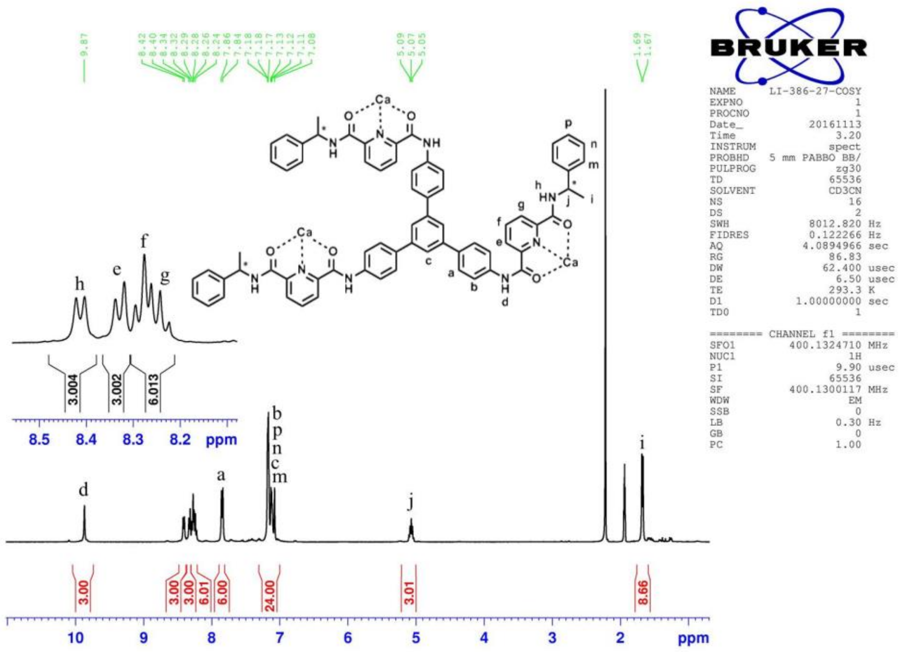

Supplementary Figure 29. ^{1}H NMR spectrum of $\mathbf{[Ca_4(L^{1S})_4](CF_3SO_3)_8}$ (CD_3CN, 400 MHz, 298 K).

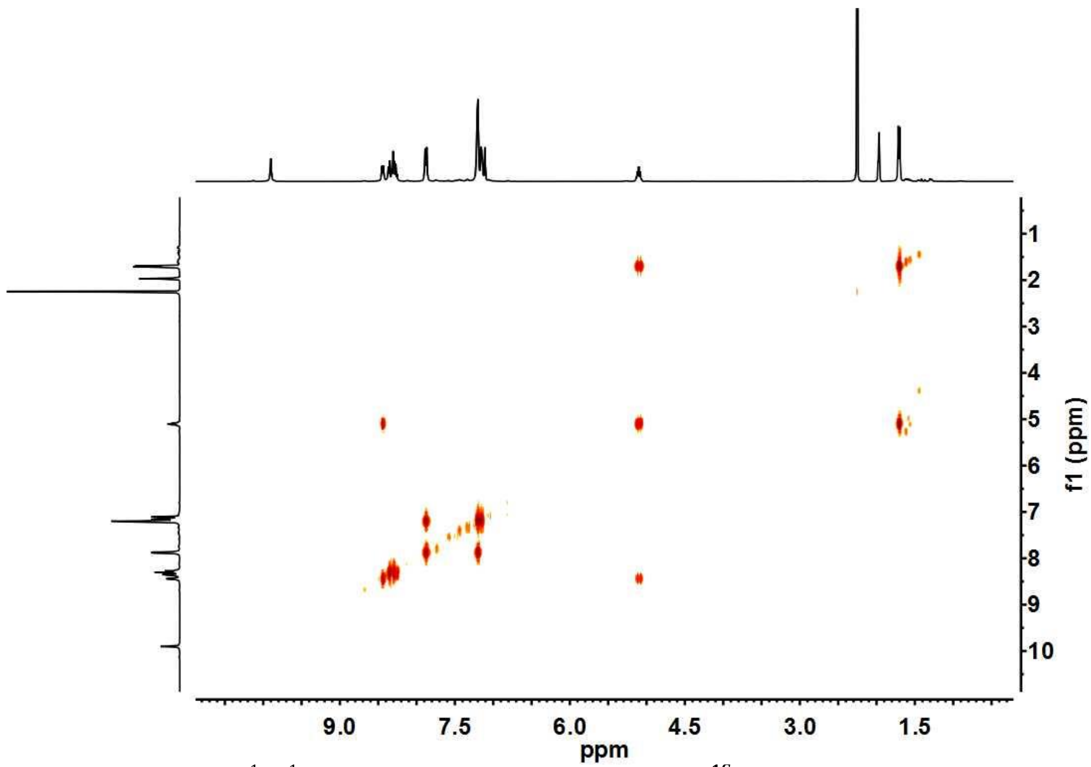

Supplementary Figure 30. ^{1}H-^{1}H COSY NMR spectrum of **[$Ca_4(L^{1S})_4$](CF_3SO_3)$_8$** (CD_3CN, 400 MHz, 298 K).

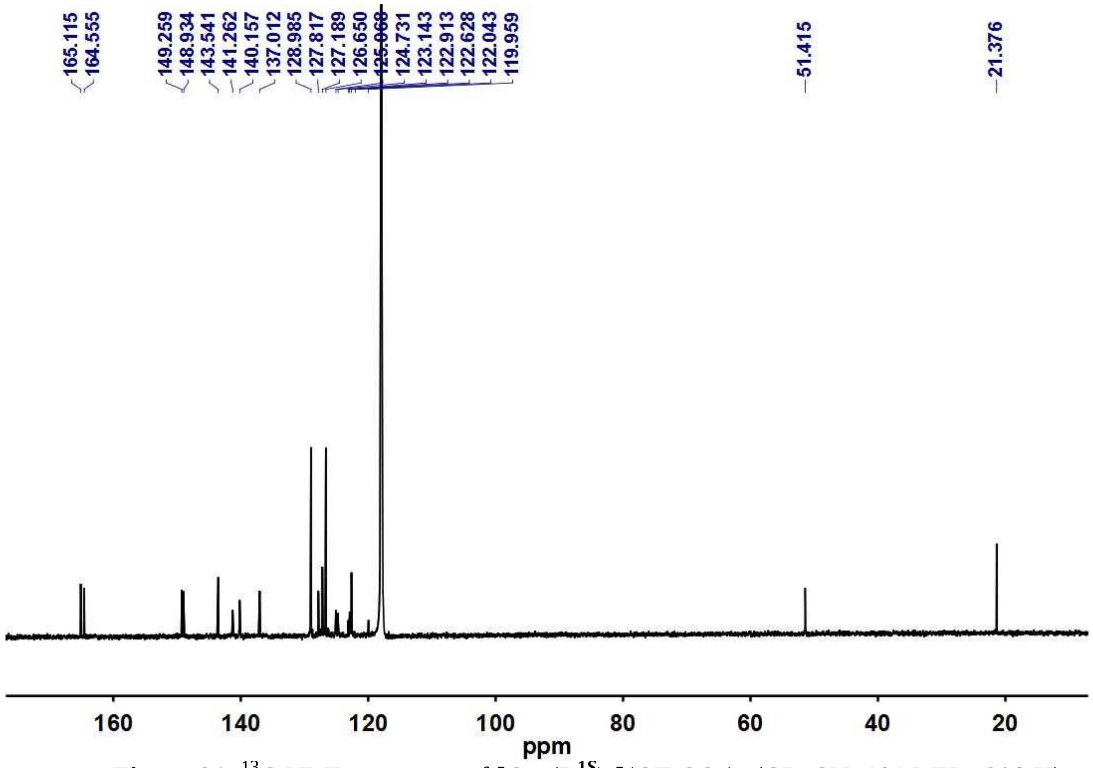

Supplementary Figure 31. ^{13}C NMR spectrum of **[$Ca_4(L^{1S})_4$](CF_3SO_3)$_8$** (CD_3CN, 101 MHz, 298 K).

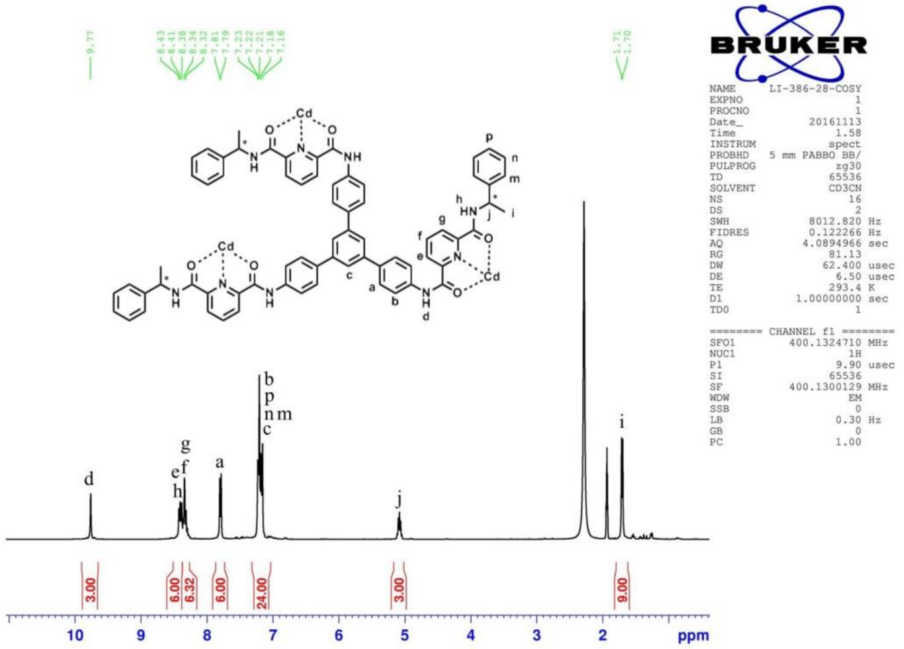

Supplementary Figure 32. ^{1}H NMR spectrum of **$[Cd_4(L^{1S})_4](ClO_4)_8$** ($CD_3CN$, 400 MHz, 298 K).

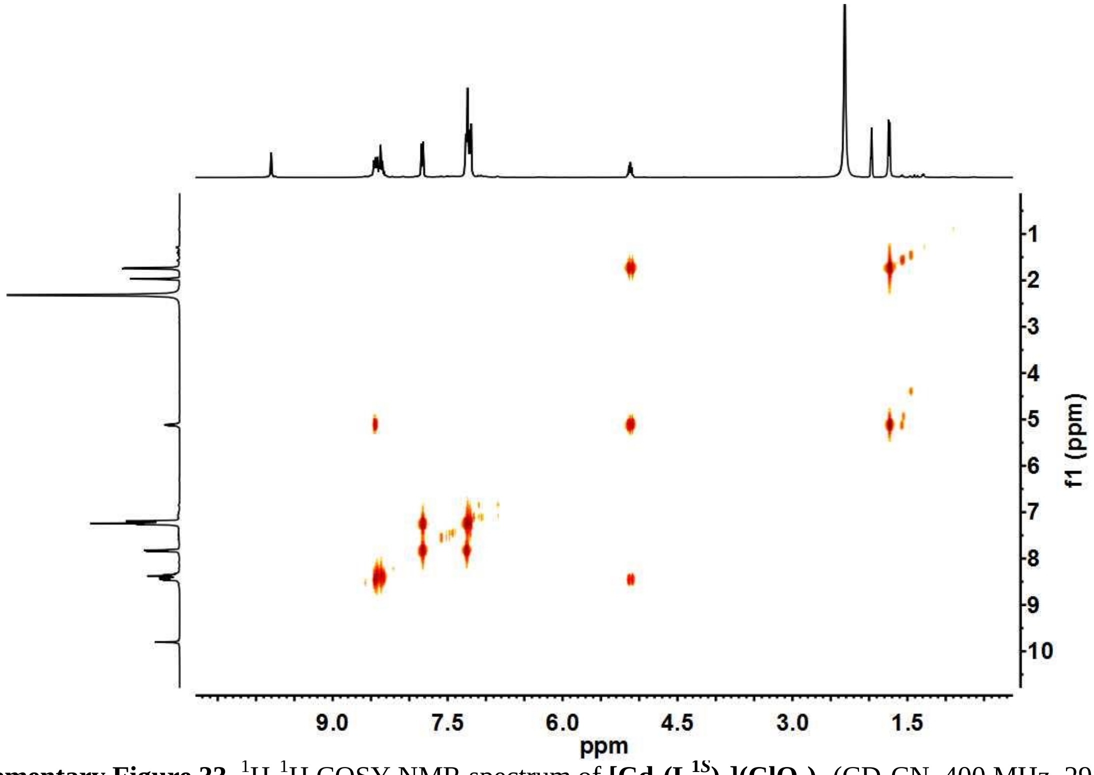

Supplementary Figure 33. ^{1}H-^{1}H COSY NMR spectrum of **$[Cd_4(L^{1S})_4](ClO_4)_8$** ($CD_3CN$, 400 MHz, 298 K).

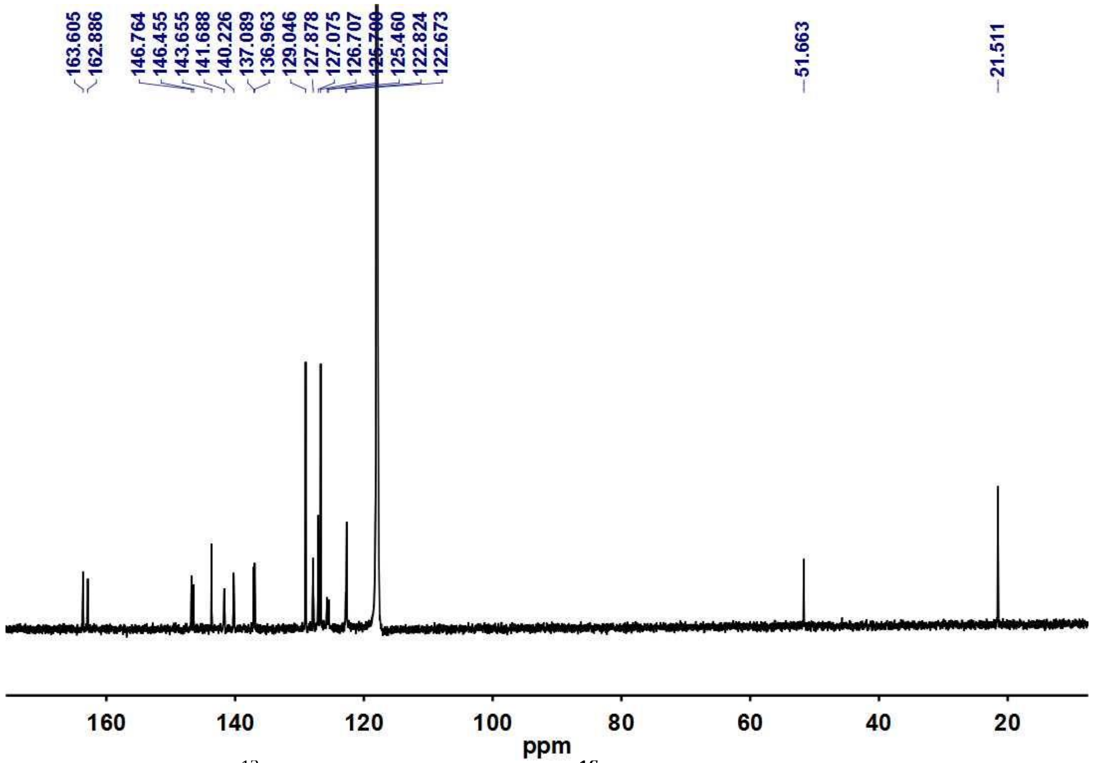

Supplementary Figure 34. ^{13}C NMR spectrum of **$[Cd_4(L^{1S})_4](ClO_4)_8$** ($CD_3CN$, 101 MHz, 298 K).

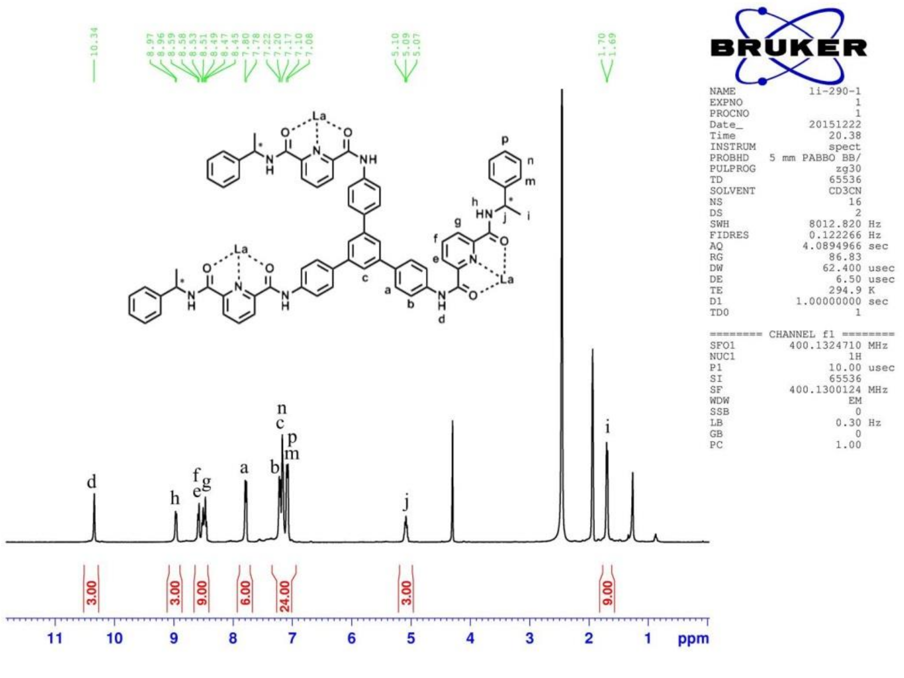

Supplementary Figure 35. ^{1}H NMR spectrum of **$[La_4(L^{1S})_4](ClO_4)_{12}$** ($CD_3CN$, 400 MHz, 298 K).

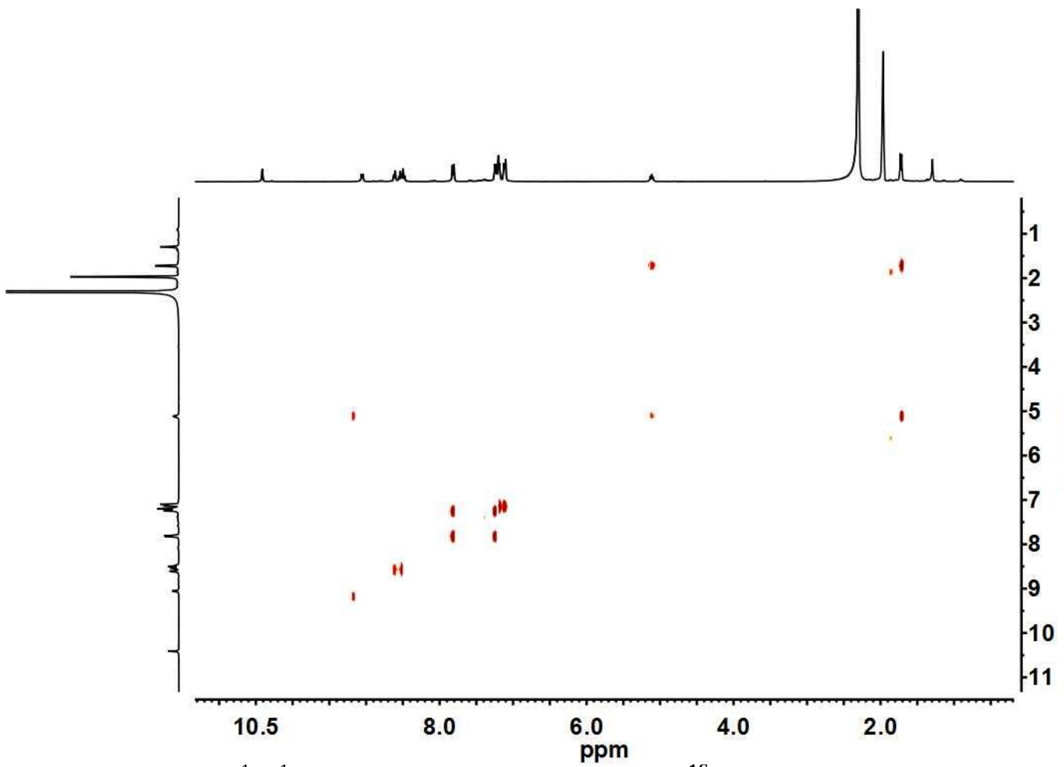

Supplementary Figure 36. ^{1}H-^{1}H COSY NMR spectrum of **$[La_4(L^{1S})_4](ClO_4)_{12}$** ($CD_3CN$, 400 MHz, 298 K).

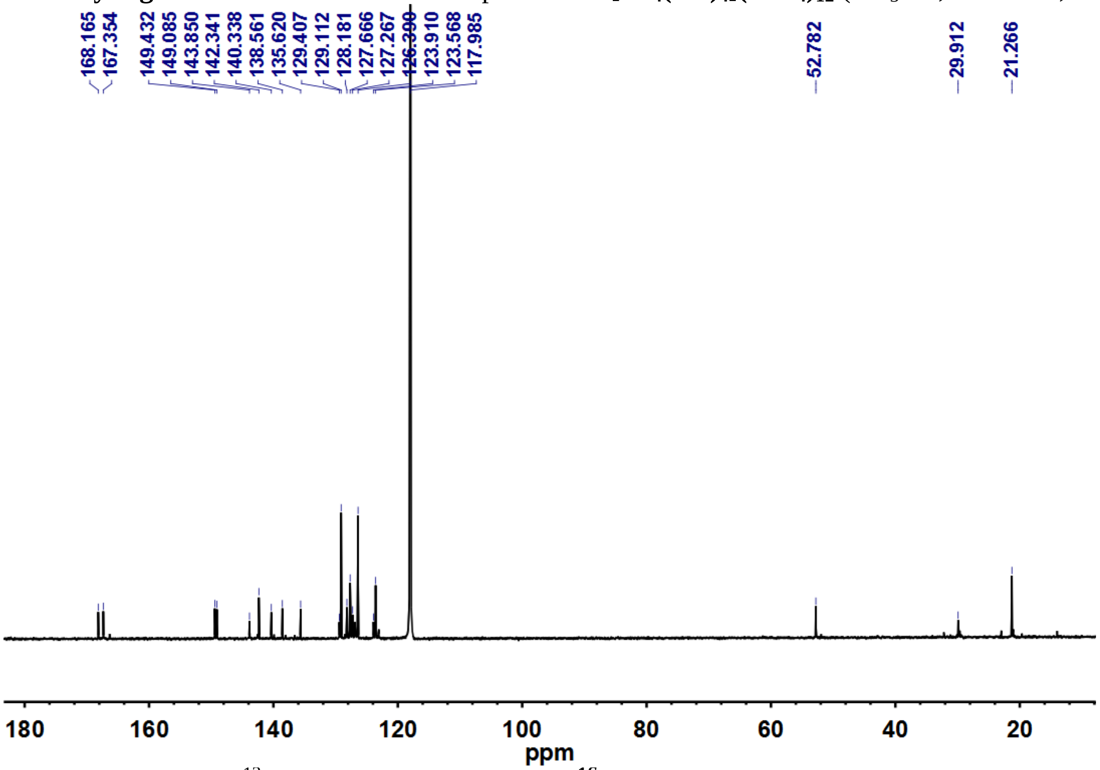

Supplementary Figure 37. ^{13}C NMR spectrum of **$[La_4(L^{1S})_4](ClO_4)_{12}$** ($CD_3CN$, 101 MHz, 298 K).

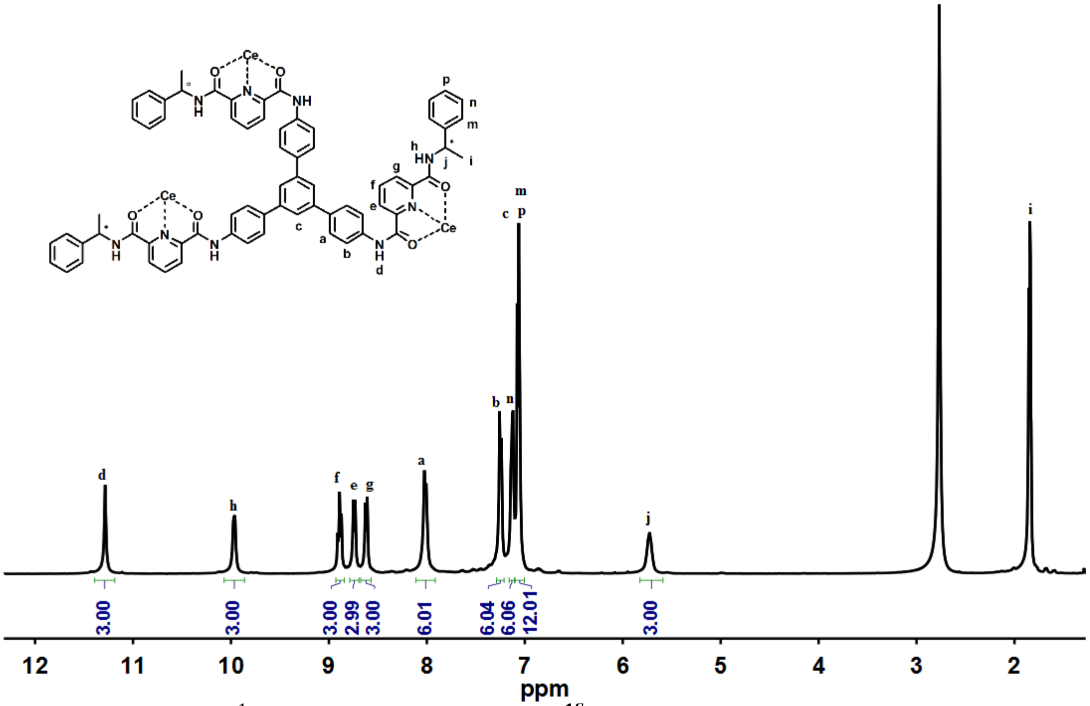

Supplementary Figure 38. ^{1}H NMR spectrum of **[Ce$_4$(L^{1S})$_4$](CF$_3$SO$_3$)$_{12}$** (CD$_3$CN, 400 MHz, 298 K).

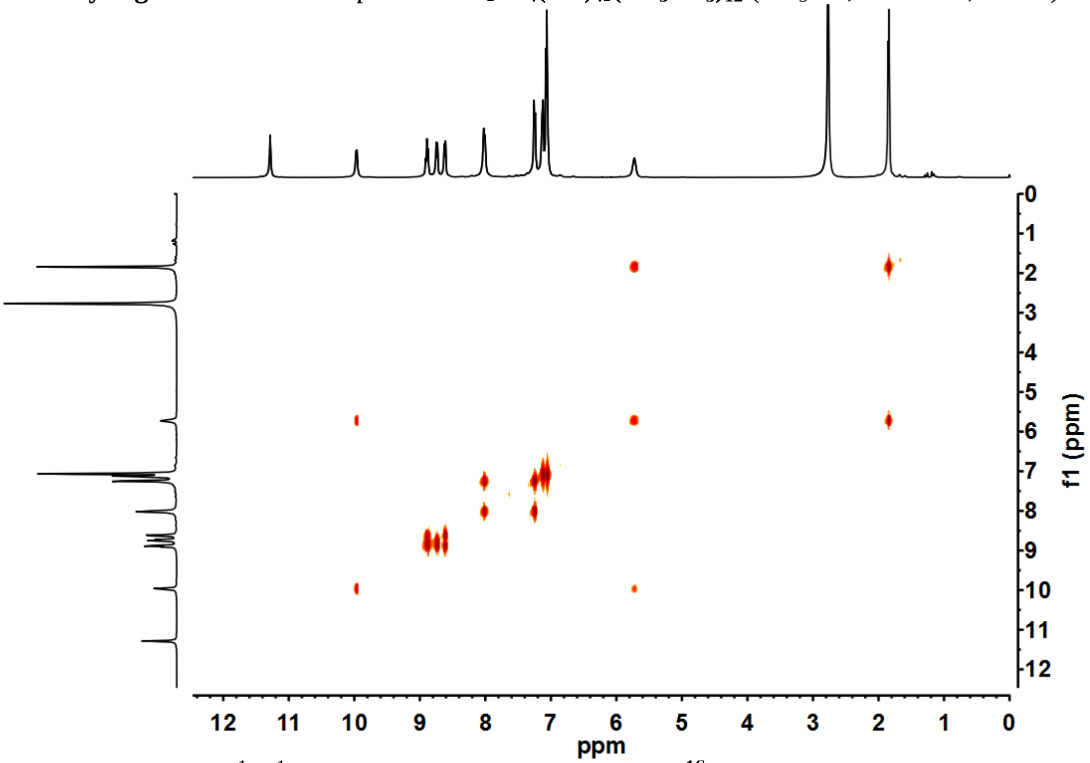

Supplementary Figure 39. ^{1}H-^{1}H COSY NMR spectrum of **[Ce$_4$(L^{1S})$_4$](CF$_3$SO$_3$)$_{12}$**(CD$_3$CN, 400 MHz, 298 K).

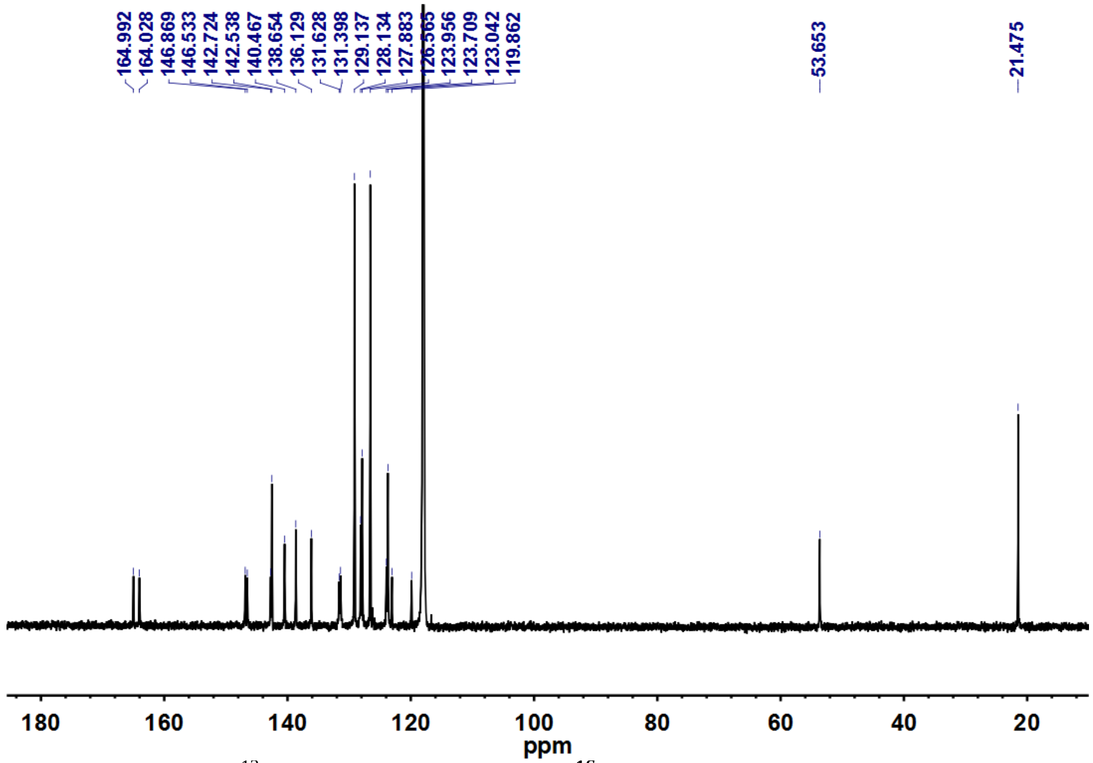

Supplementary Figure 40. ^{13}C NMR spectrum of **$[Ce_4(L^{1S})_4](CF_3SO_3)_{12}$** ($CD_3CN$, 101 MHz, 298 K).

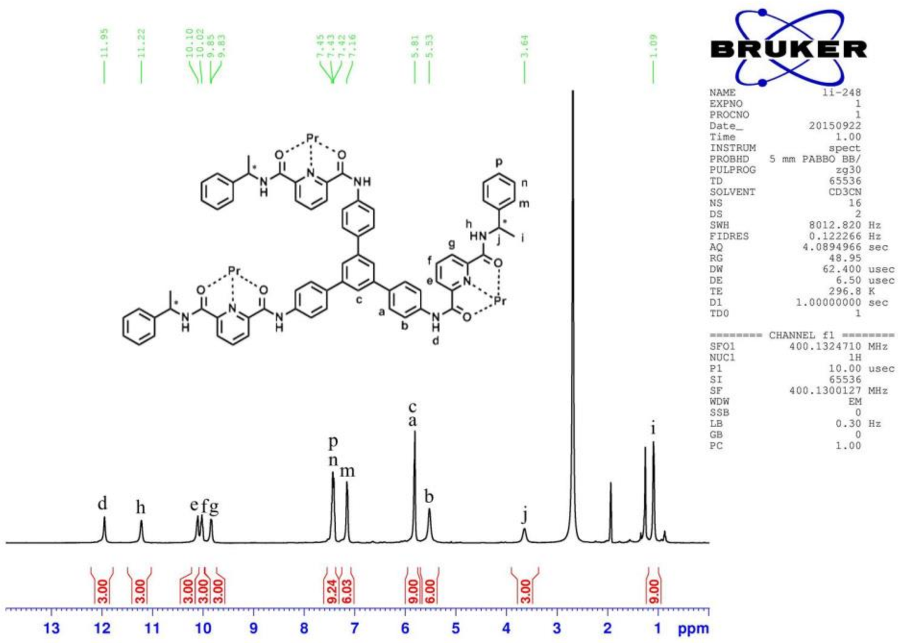

Supplementary Figure 41. ^{1}H NMR spectrum of **$[Pr_4(L^{1S})_4](ClO_4)_{12}$** ($CD_3CN$, 400 MHz, 298 K).

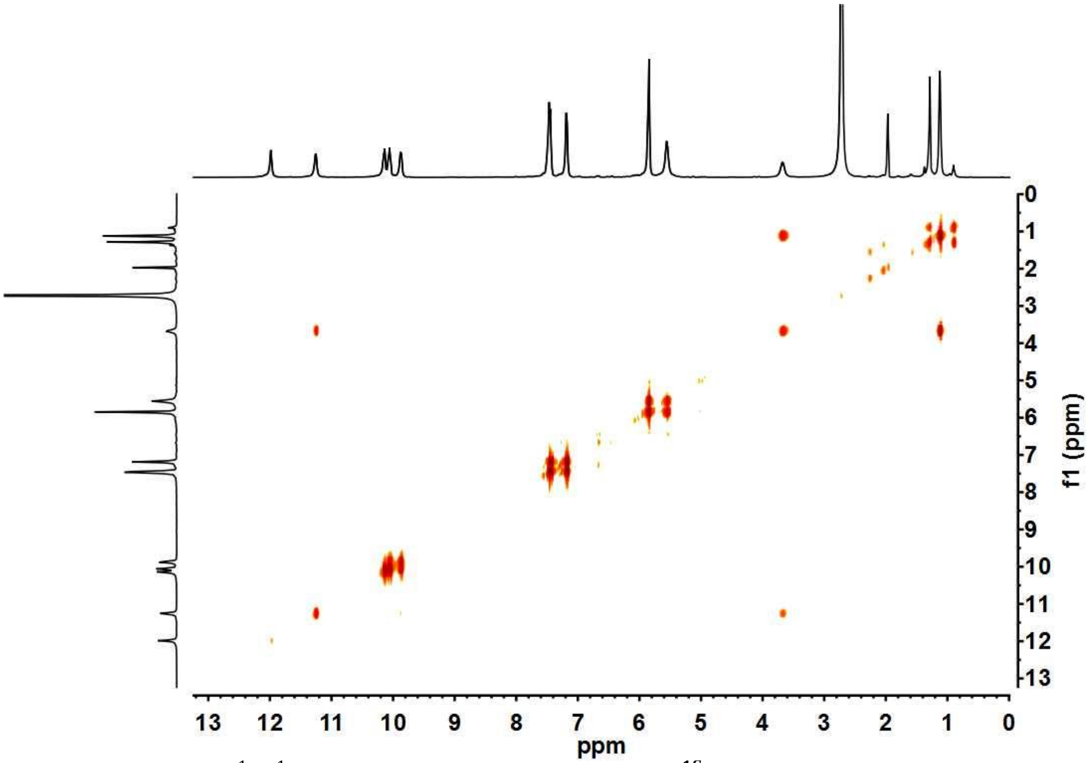

Supplementary Figure 42. ^{1}H-^{1}H COSY NMR spectrum of **[Pr$_4$(L^{1S})$_4$](ClO$_4$)$_{12}$** (CD$_3$CN, 400 MHz, 298 K).

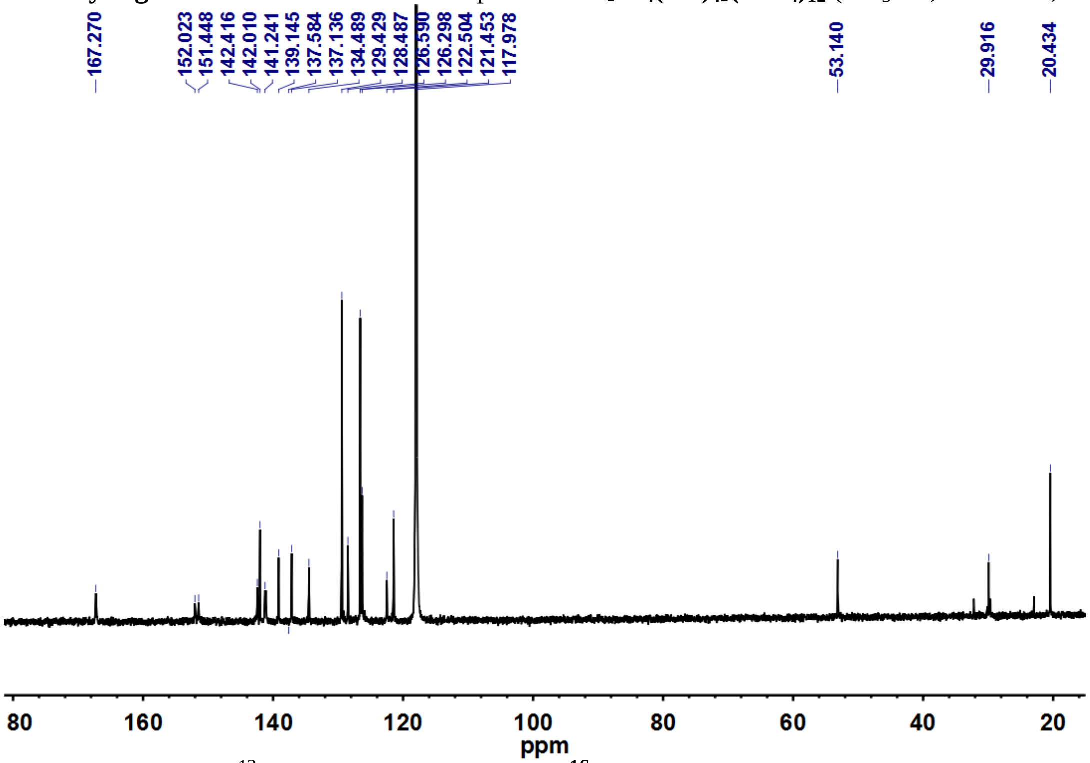

Supplementary Figure 43. ^{13}C NMR spectrum of **[Pr$_4$(L^{1S})$_4$](ClO$_4$)$_{12}$** (CD$_3$CN, 101 MHz, 298 K).

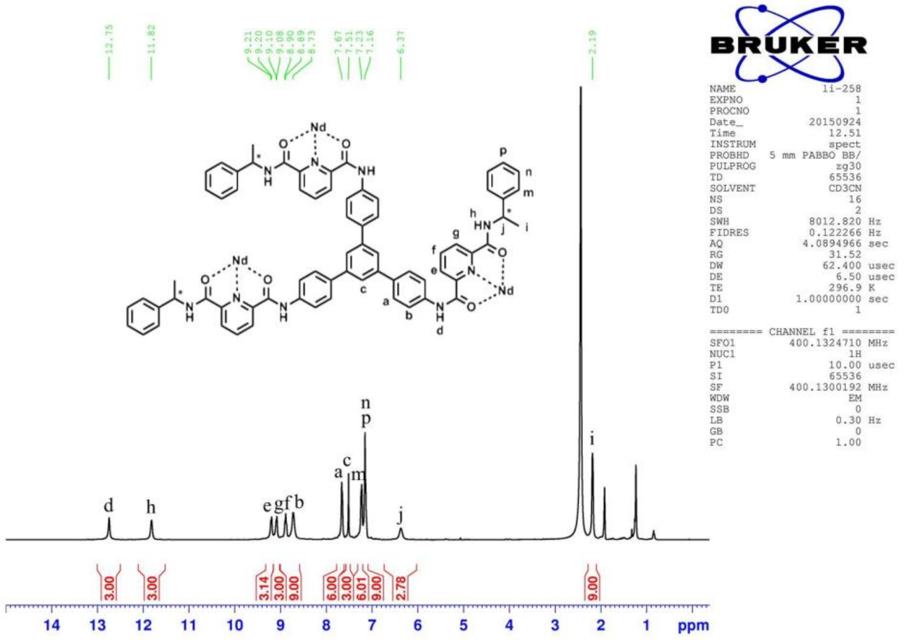

Supplementary Figure 44. ^{1}H NMR spectrum of **$[Nd_4(L^{1S})_4](ClO_4)_{12}$** ($CD_3CN$, 400 MHz, 298 K).

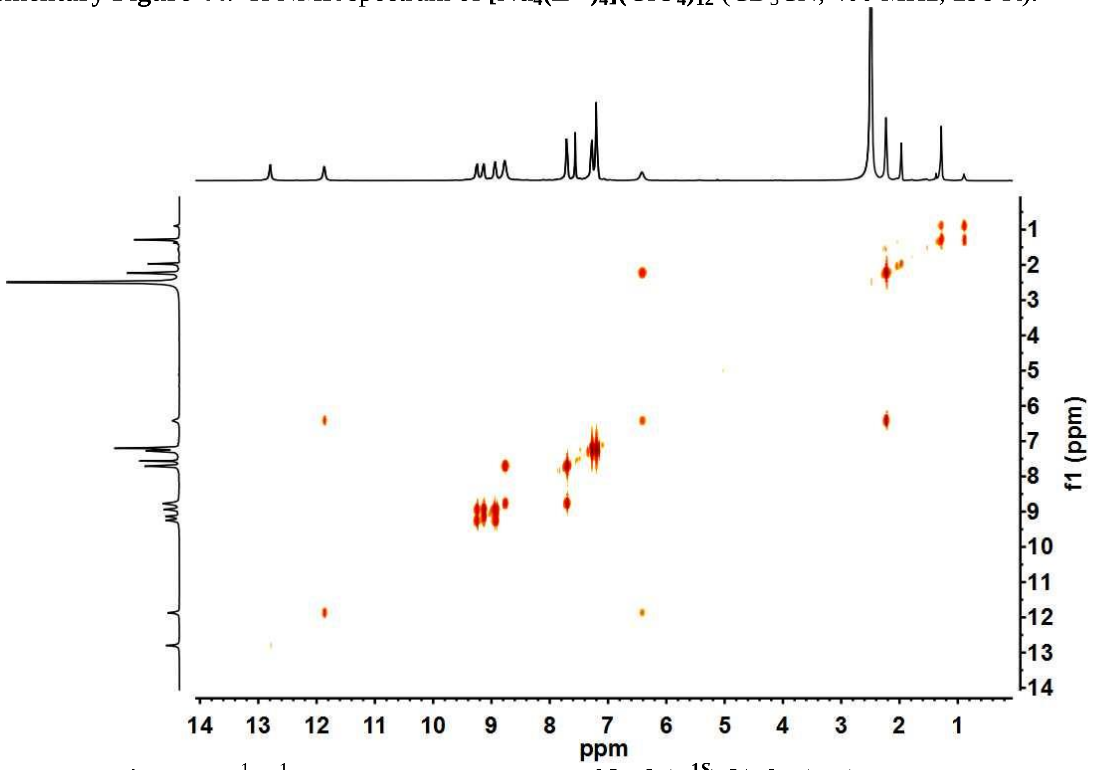

Supplementary Figure 45. ^{1}H-^{1}H COSY NMR spectrum of **$[Nd_4(L^{1S})_4](ClO_4)_{12}$** ($CD_3CN$, 400 MHz, 298 K).

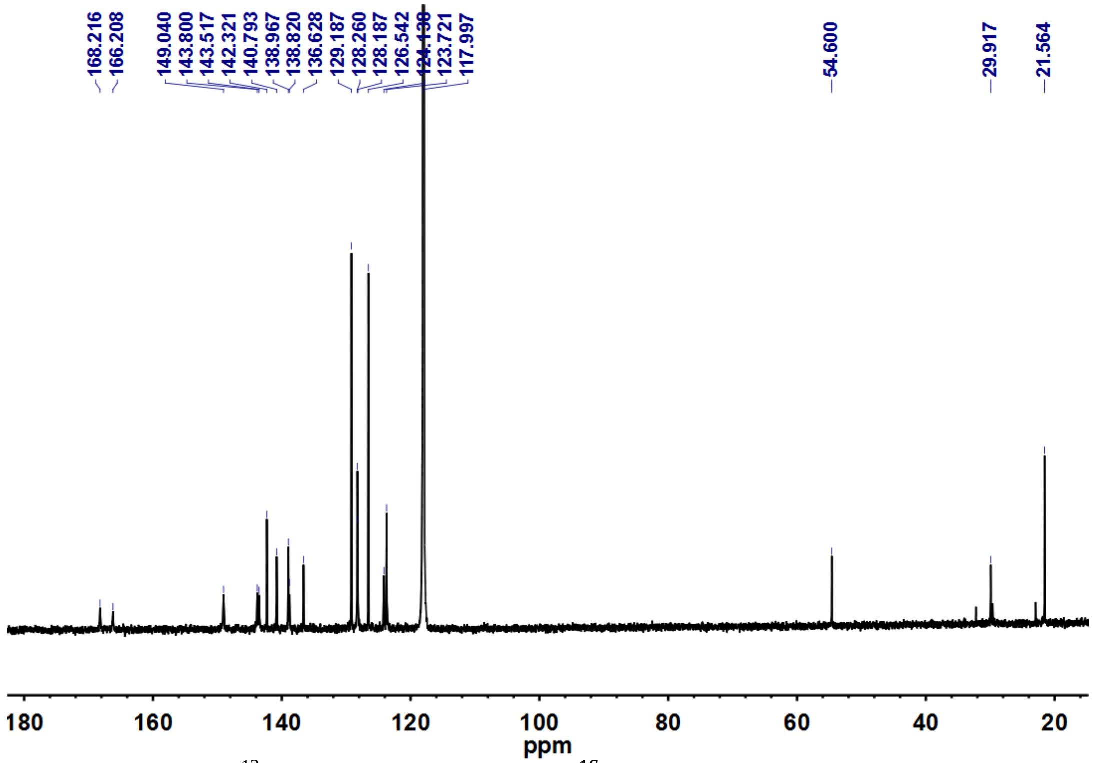

Supplementary Figure 46. ^{13}C NMR spectrum of **$[Nd_4(L^{1S})_4](ClO_4)_{12}$** ($CD_3CN$, 101 MHz, 298 K).

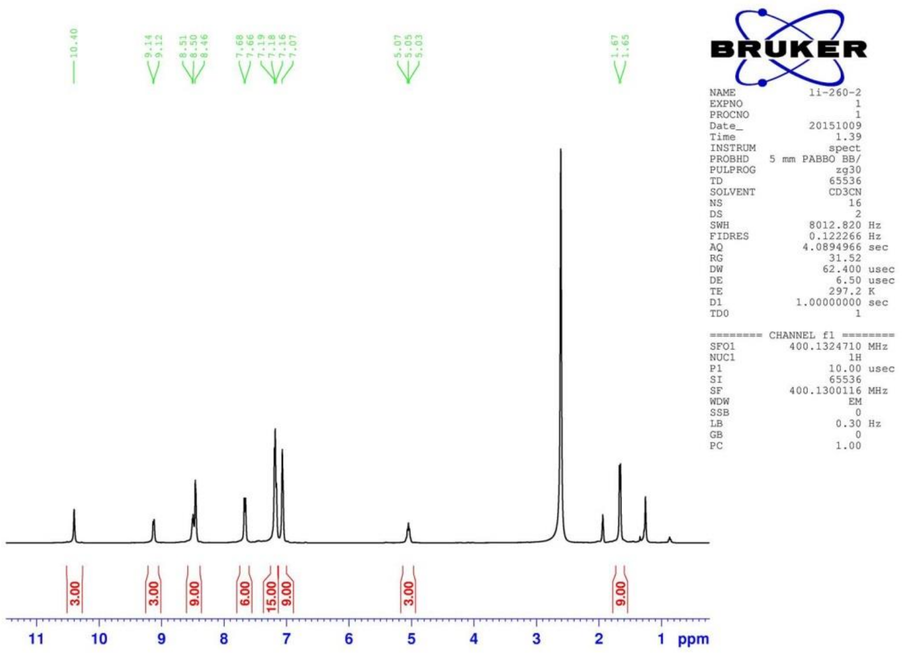

Supplementary Figure 47. ^{1}H NMR spectrum of **$[Sm_4(L^{1S})_4](ClO_4)_{12}$** ($CD_3CN$, 400 MHz, 298 K).

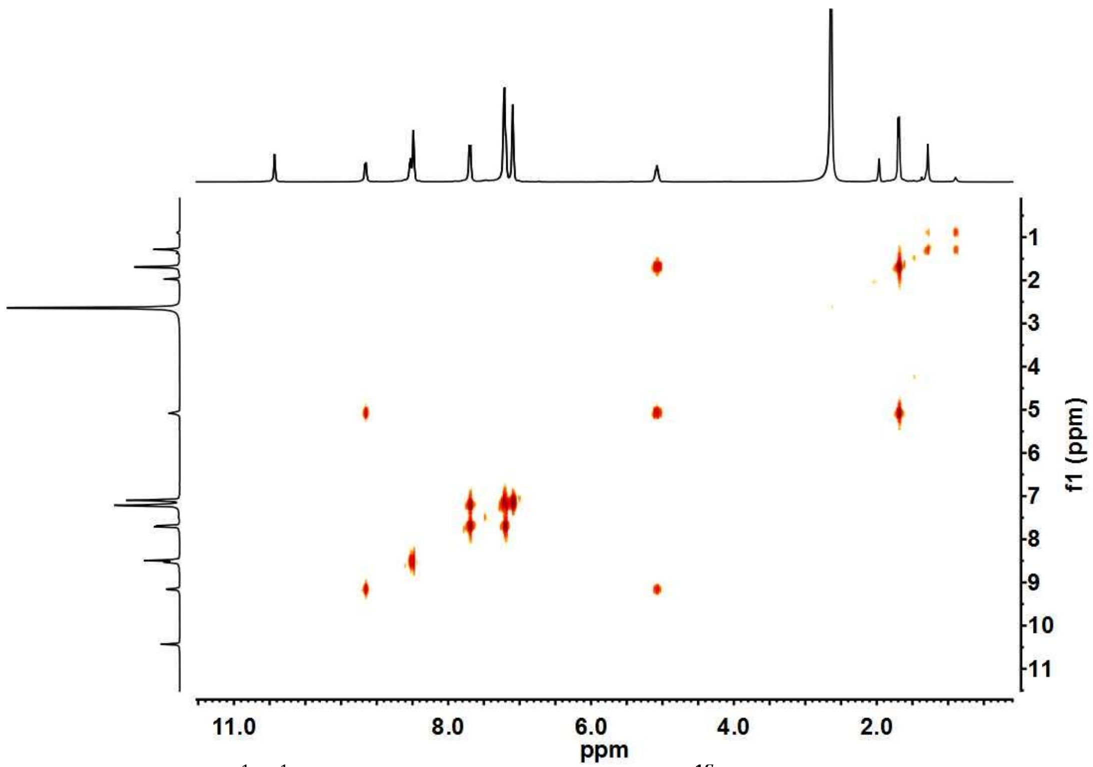

Supplementary Figure 48. ^1H-^1H COSY NMR spectrum of **$[Sm_4(L^{1S})_4](ClO_4)_{12}$** ($CD_3CN$, 400 MHz, 298 K).

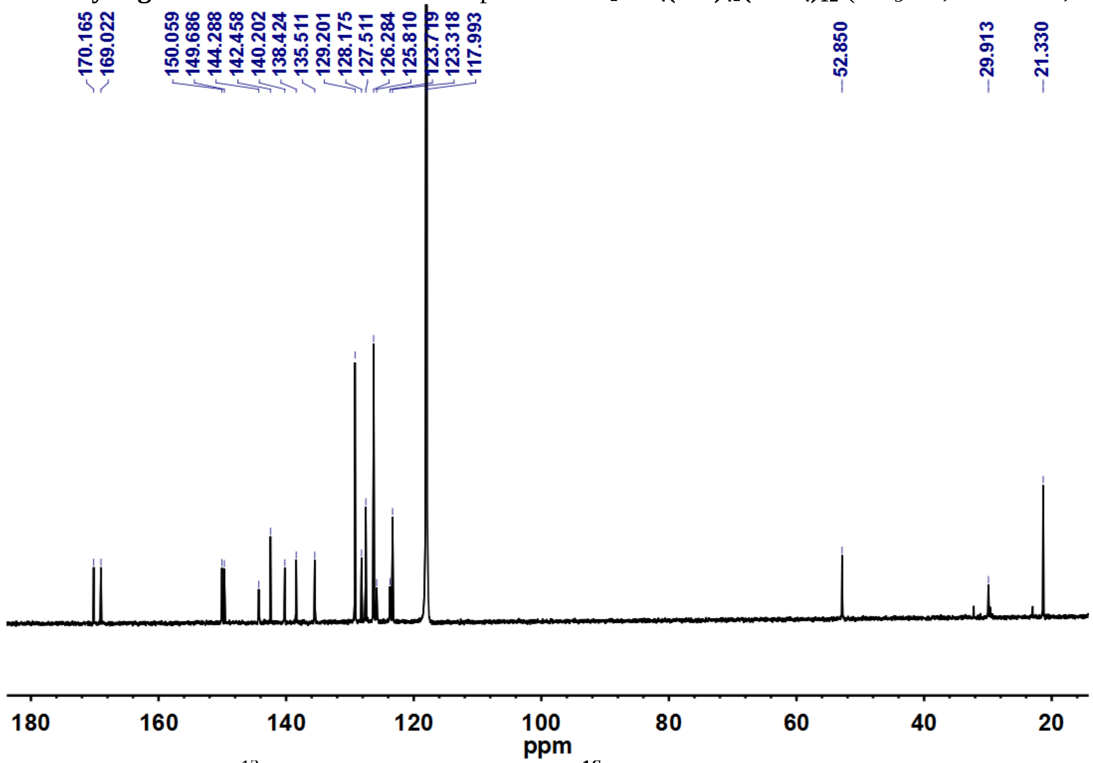

Supplementary Figure 49. ^{13}C NMR spectrum of **$[Sm_4(L^{1S})_4](ClO_4)_{12}$** ($CD_3CN$, 101 MHz, 298 K).

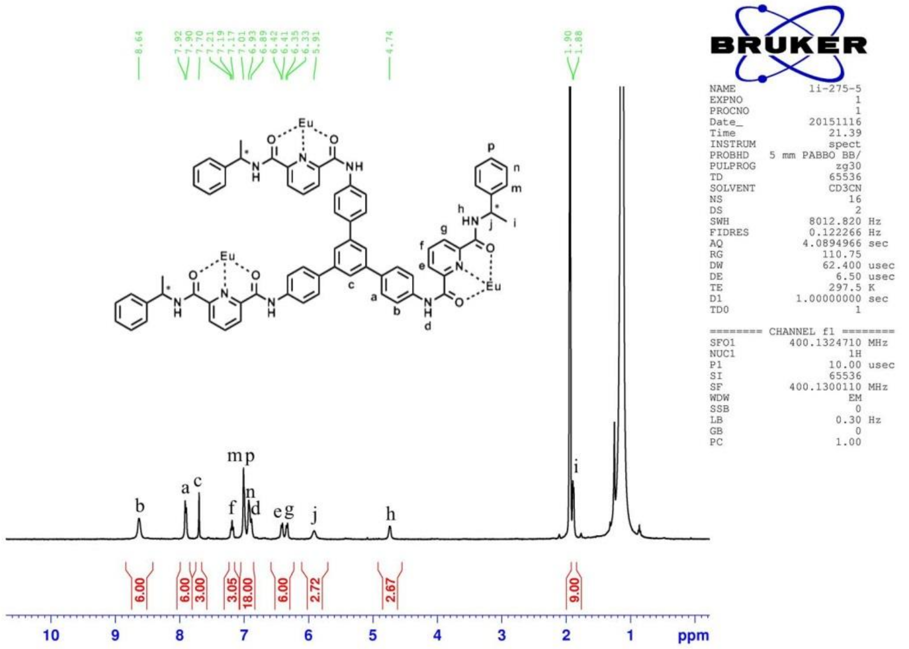

Supplementary Figure 50. ¹H NMR spectrum of **[$Eu_4(L^{1S})_4$](ClO_4)$_{12}$** (CD_3CN, 400 MHz, 298 K).

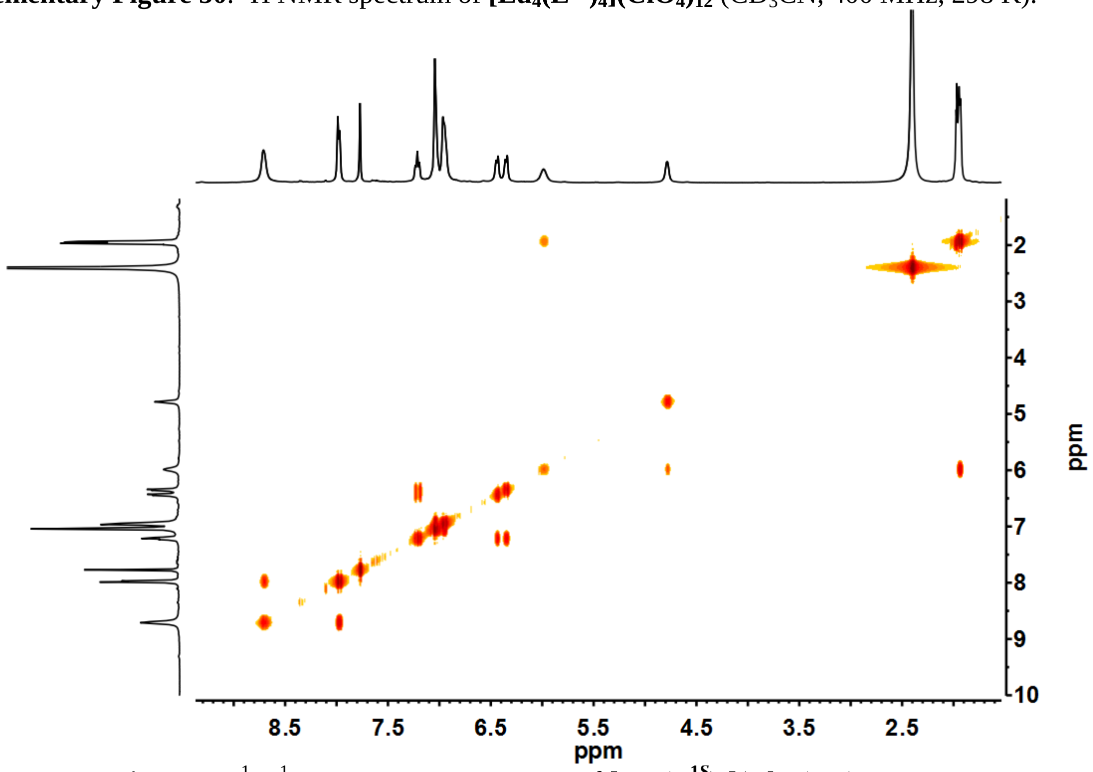

Supplementary Figure 51. ¹H-¹H COSY NMR spectrum of **[$Eu_4(L^{1S})_4$](ClO_4)$_{12}$** (CD_3CN, 400 MHz, 298 K).

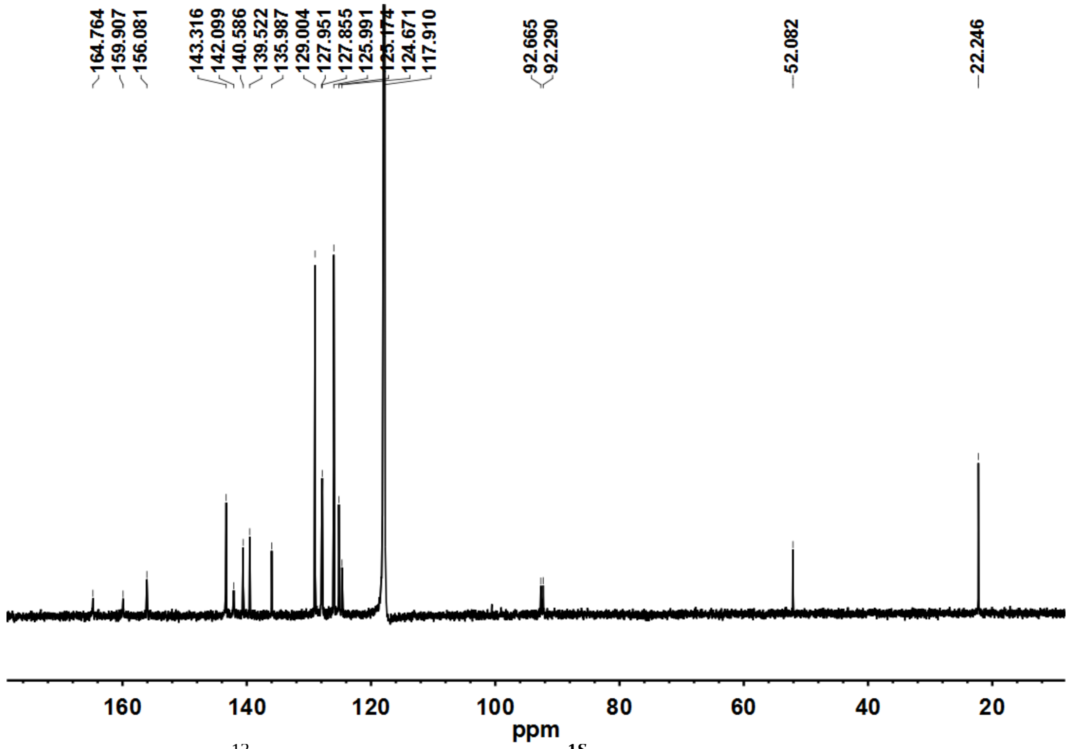

Supplementary Figure 52. ^{13}C NMR spectrum of **[Eu$_4$(L^{1S})$_4$](ClO$_4$)$_{12}$** (CD$_3$CN, 101 MHz, 298 K).

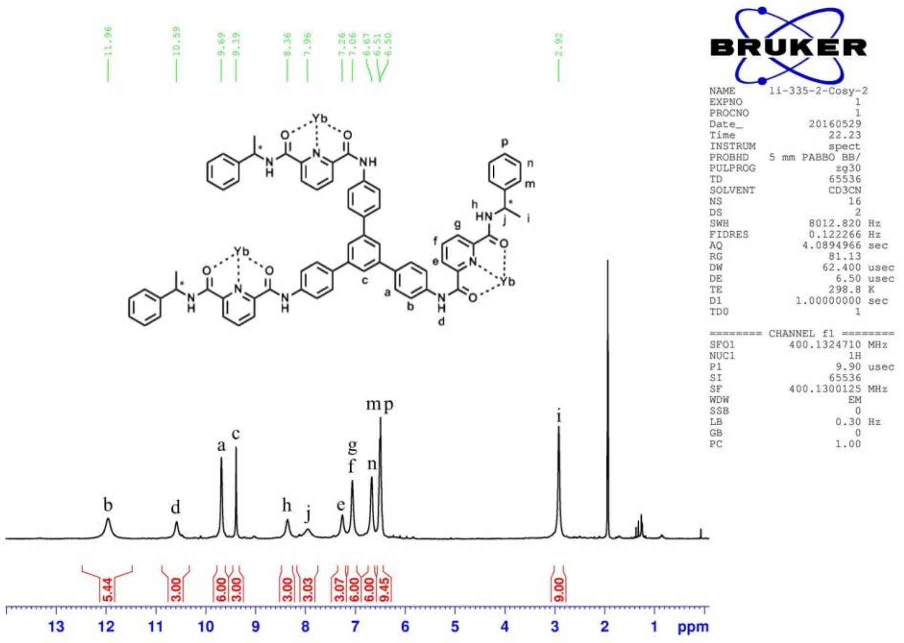

Supplementary Figure 53. ^{1}H NMR spectrum of **[Yb$_4$(L^{1S})$_4$](CF$_3$SO$_3$)$_{12}$** (CD$_3$CN, 400 MHz, 298 K).

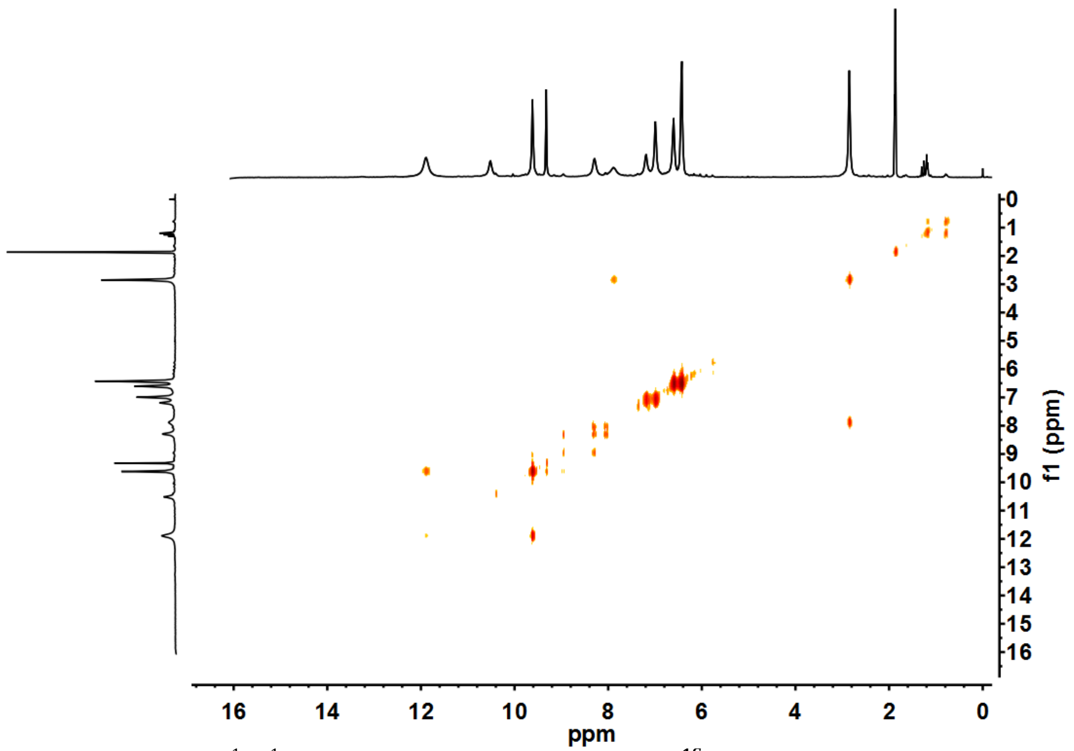

Supplementary Figure 54. ^{1}H-^{1}H COSY NMR spectrum of **$[Yb_4(L^{1S})_4](CF_3SO_3)_{12}$**($CD_3CN$, 400 MHz, 298) K).

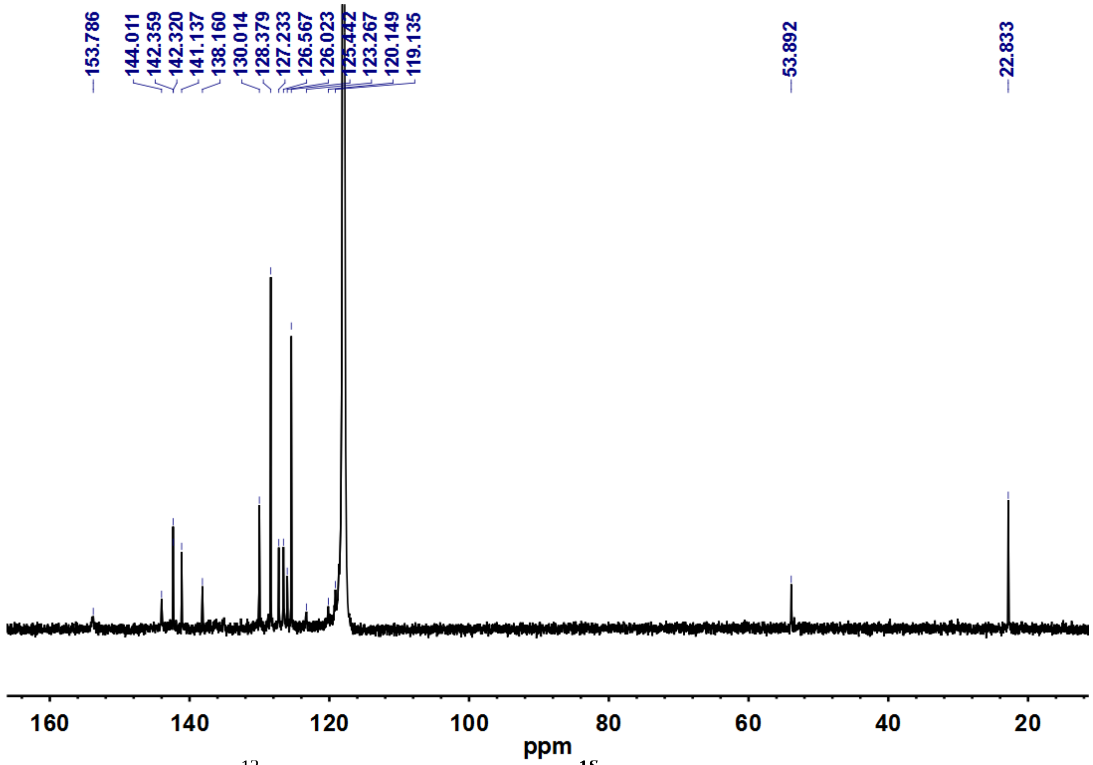

Supplementary Figure 55. ^{13}C NMR spectrum of **$[Yb_4(L^{1S})_4](CF_3SO_3)_{12}$** ($CD_3CN$, 101 MHz, 298 K).

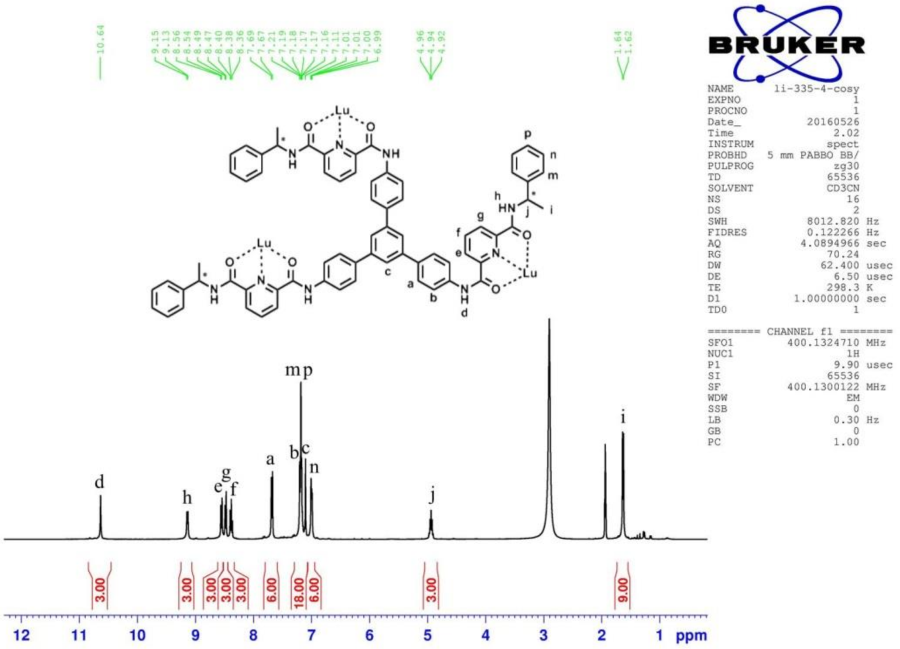

Supplementary Figure 56. ^{1}H NMR spectrum of **$[Lu_4(L^{1S})_4](CF_3SO_3)_{12}$** ($CD_3CN$, 400 MHz, 298 K).

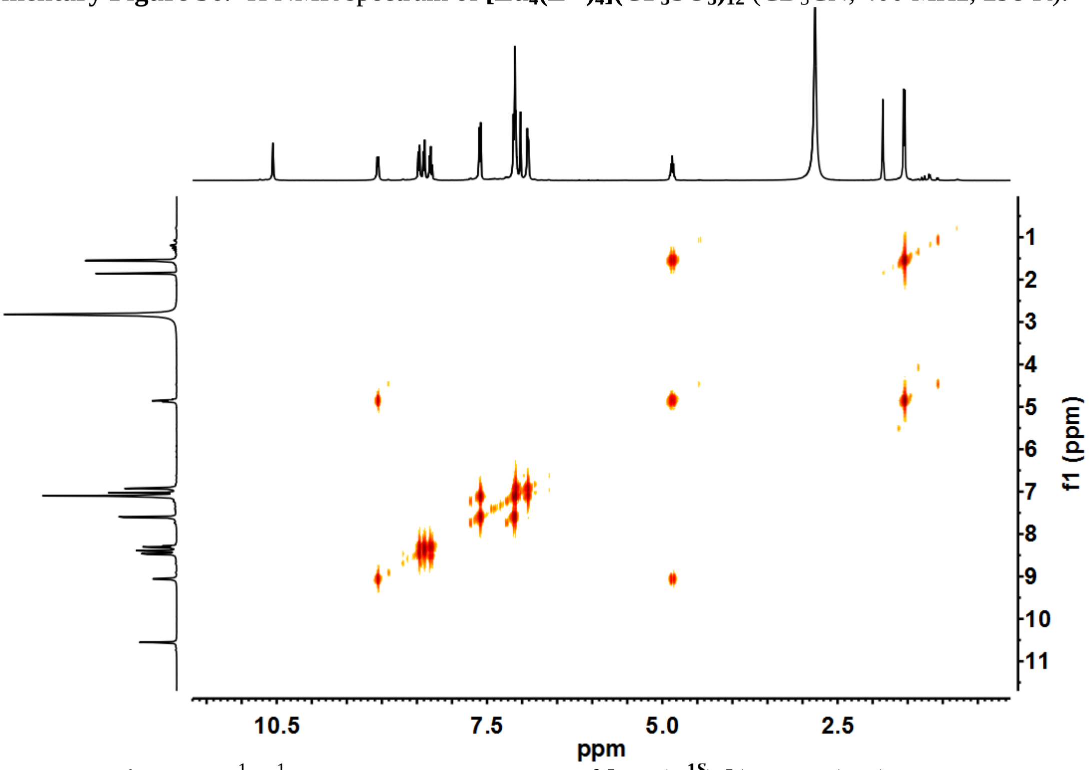

Supplementary Figure 57. ^{1}H-^{1}H COSY NMR spectrum of **$[Lu_4(L^{1S})_4](CF_3SO_3)_{12}$** ($CD_3CN$, 400 MHz, 298 K).

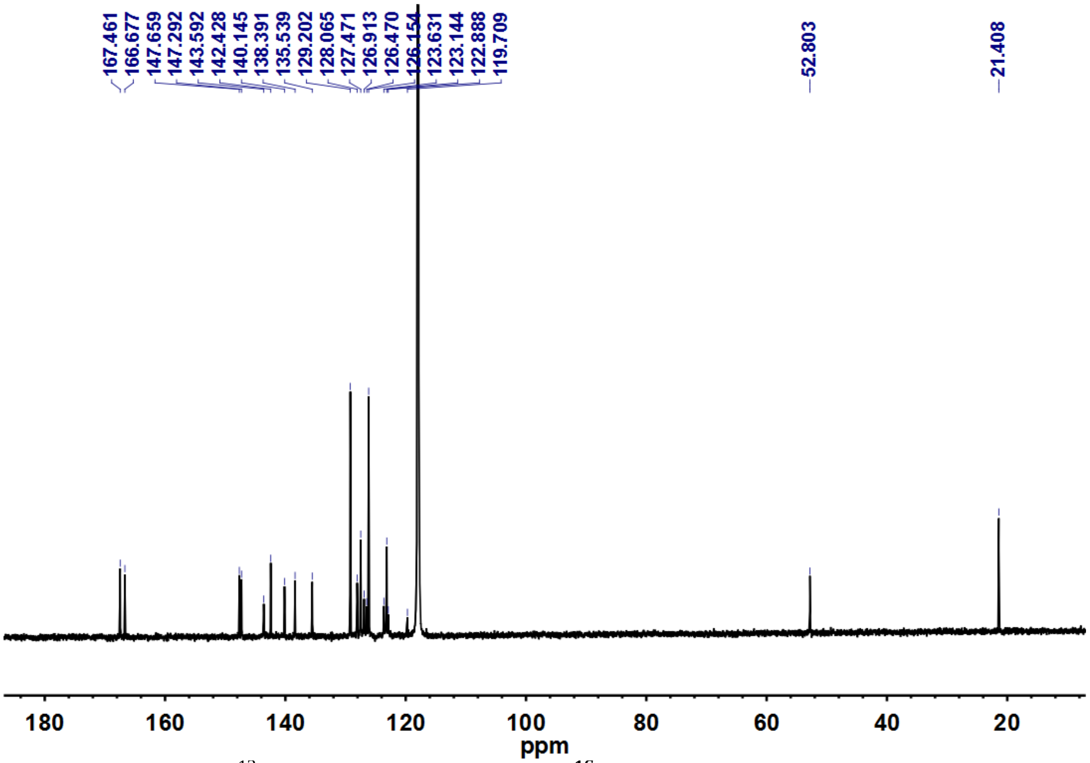

Supplementary Figure 58. ^{13}C NMR spectrum of **[Lu$_4$(L^{1S})$_4$](CF$_3$SO$_3$)$_{12}$** (CD$_3$CN, 101 MHz, 298 K).

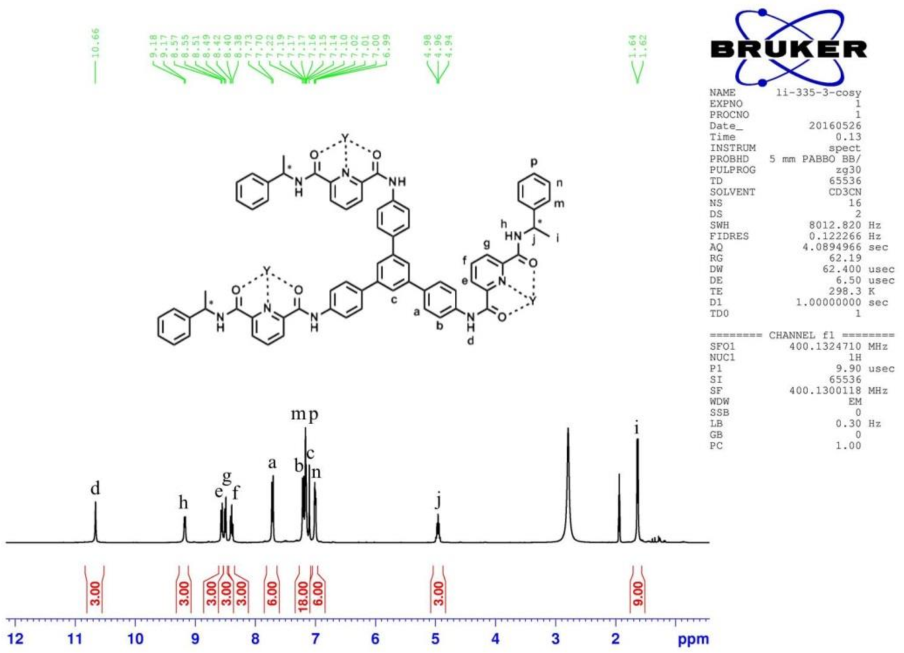

Supplementary Figure 59. ^{1}H NMR spectrum of **[Y$_4$(L^{1S})$_4$](ClO$_4$)$_{12}$** (CD$_3$CN, 400 MHz, 298 K).

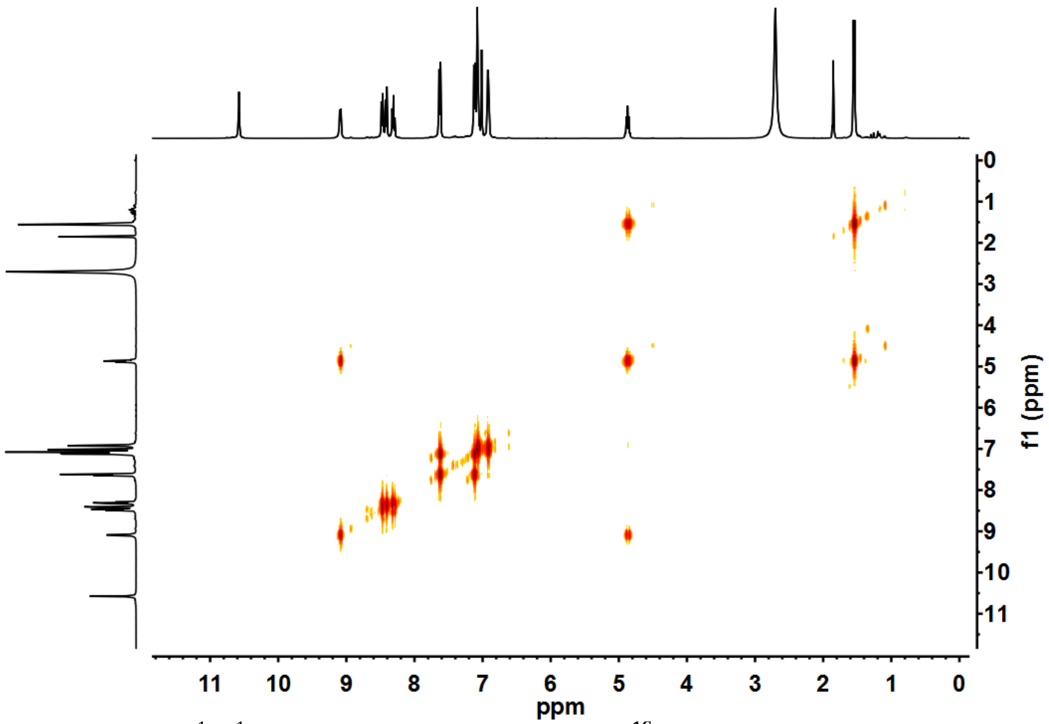

Supplementary Figure 60. ¹H-¹H COSY NMR spectrum of **[$Y_4(L^{1S})_4](ClO_4)_{12}$** ($CD_3CN$, 400 MHz, 298 K).

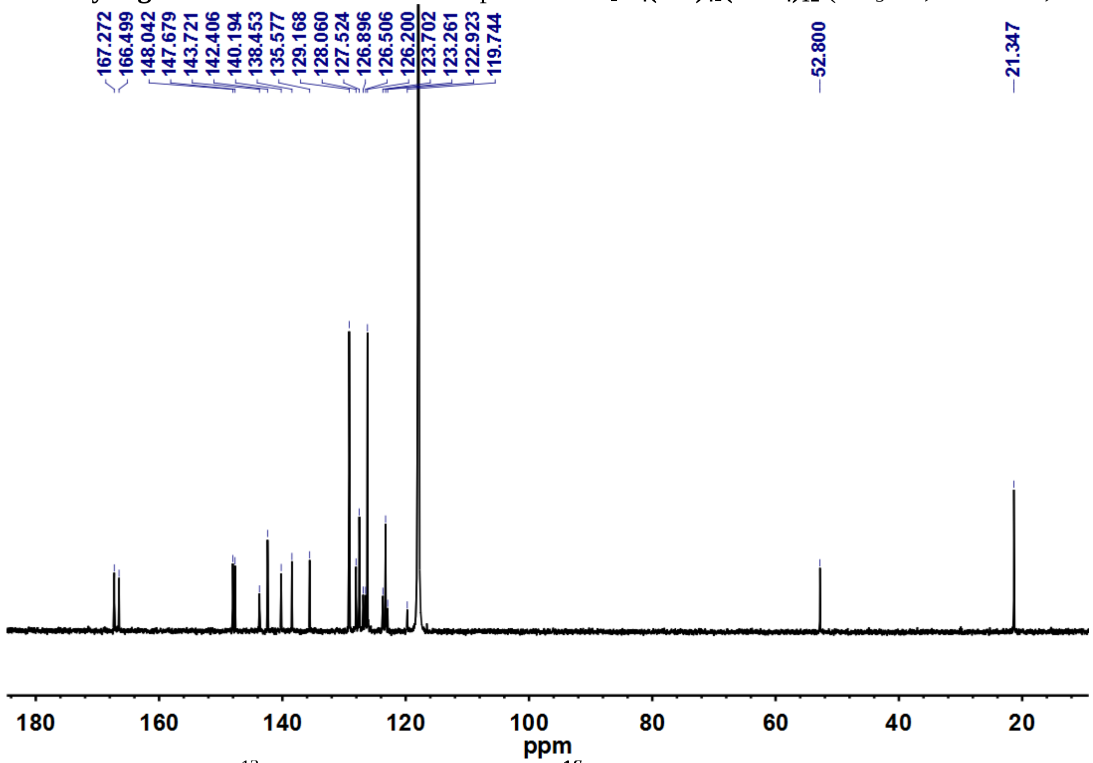

Supplementary Figure 61. ¹³C NMR spectrum of **[$Y_4(L^{1S})_4](ClO_4)_{12}$** ($CD_3CN$, 101 MHz, 298 K).

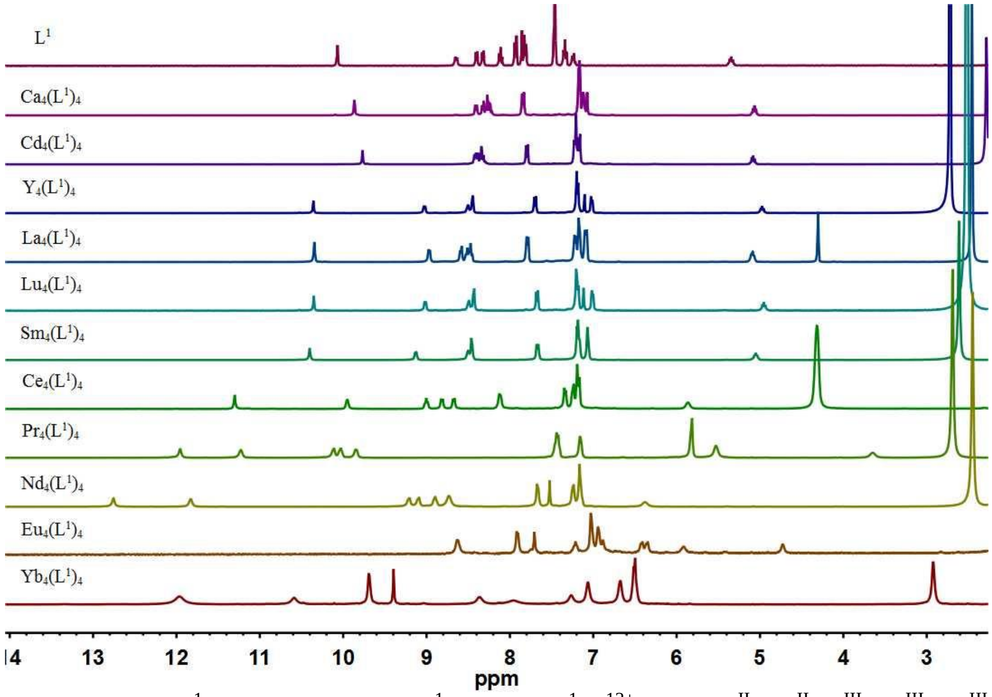

Supplementary Figure 62. ^{1}H NMR spectra of L^1 and $[M_4(L^1)_4]^{12+}$ (M = Ca^{II}, Cd^{II}, Y^{III}, La^{III}, Lu^{III}, Sm^{III}, Ce^{III}, Pr^{III}, Nd^{III}, Eu^{III}, Yb^{III}) (400 MHz, CD_3CN, 298K).

DOSY spectra

Stokes-Einstein equation

$$D = \frac{K_B T}{6\pi\eta r}$$

was applied to estimate the dynamic radius of $[M_4L^1{}_4]^{8+}$ (M = Ca^{II} or Cd^{II}) $[Ln_4L^1{}_4]^{12+}$ complexes. D is diffusion coefficient obtained from DOSY spectrum, K_B is Boltzmann constant, T is the absolute temperature, viscosity tested to be 0.343 mPa ·S, and r is the estimated dynamic radius.

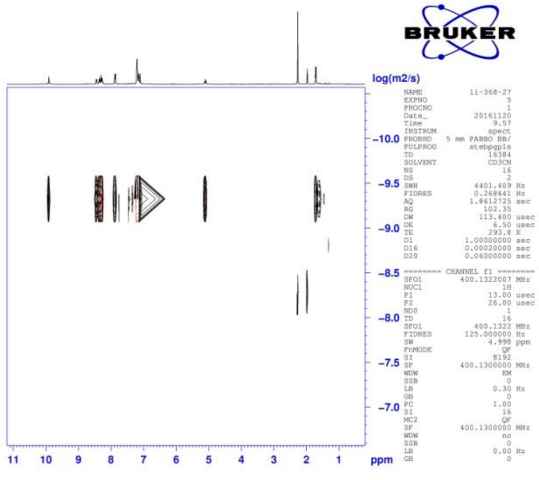

Diffusion Constant = 4.198E-10m^2/S   d = 2.9 nm

Supplementary Figure 63. ^{1}H DOSY spectrum of $\mathbf{[Ca_4(L^{1S})_4](CF_3SO_3)_8}$ (CD_3CN, 400 MHz, 298 K).

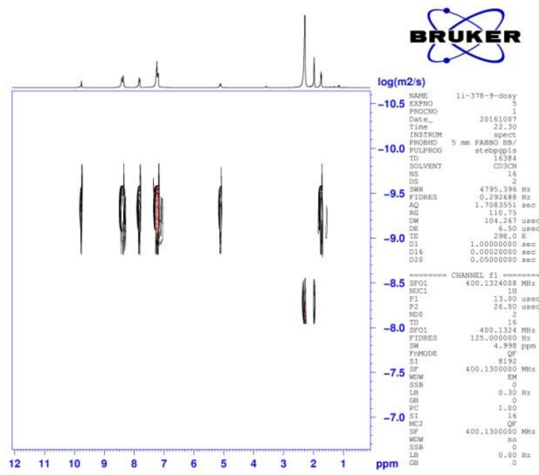

Diffusion Constant = 4.276E-10m^2/S d = 2.8 nm

Supplementary Figure 64. ^{1}H DOSY spectrum of **$[Cd_4(L^{1S})_4](ClO_4)_8$** ($CD_3CN$, 400 MHz, 298 K).

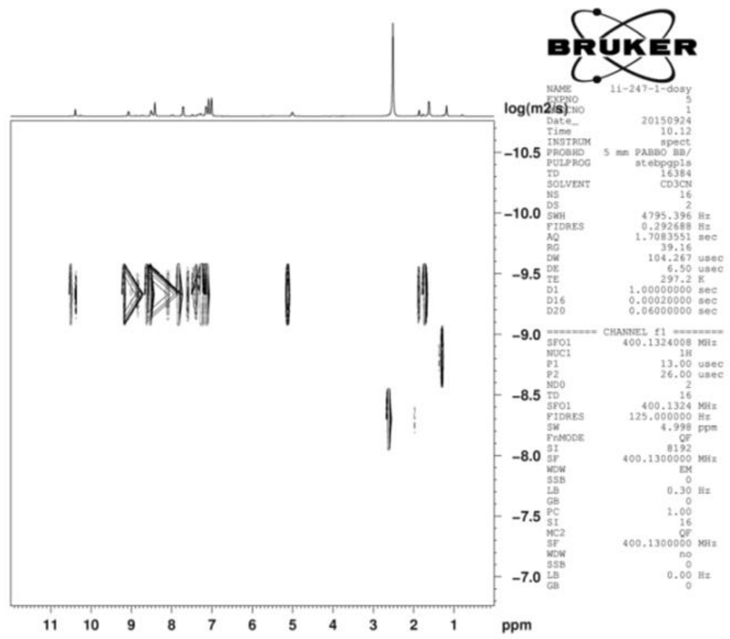

Diffusion Constant = 4.198E-10m^2/S d = 2.9 nm

Supplementary Figure 65. ^{1}H DOSY spectrum of **$[La_4(L^{1S})_4](ClO_4)_{12}$** ($CD_3CN$, 400 MHz, 298 K).

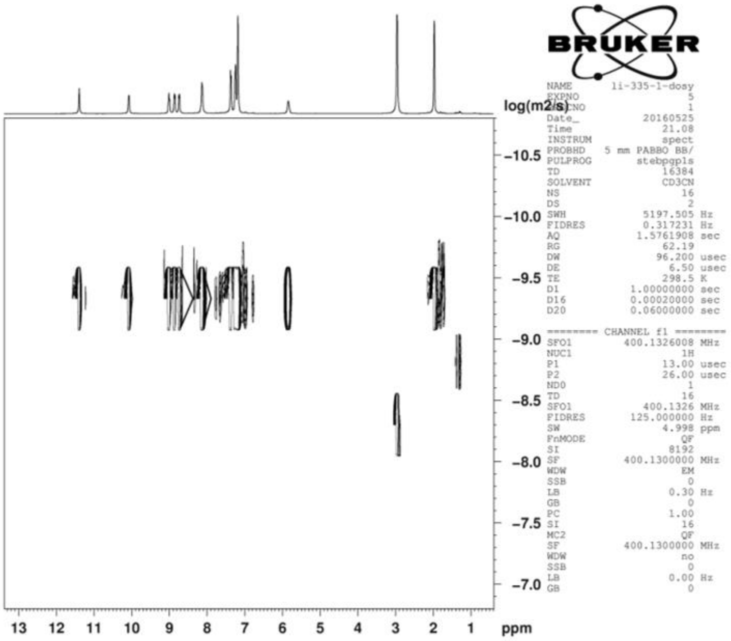

Diffusion Constant = 4.121E-10m^2/S   d = 3.0 nm

Supplementary Figure 66. ^{1}H DOSY spectrum of **$[Ce_4(L^{1S})_4](CF_3SO_3)_{12}$** ($CD_3CN$, 400 MHz, 298 K).

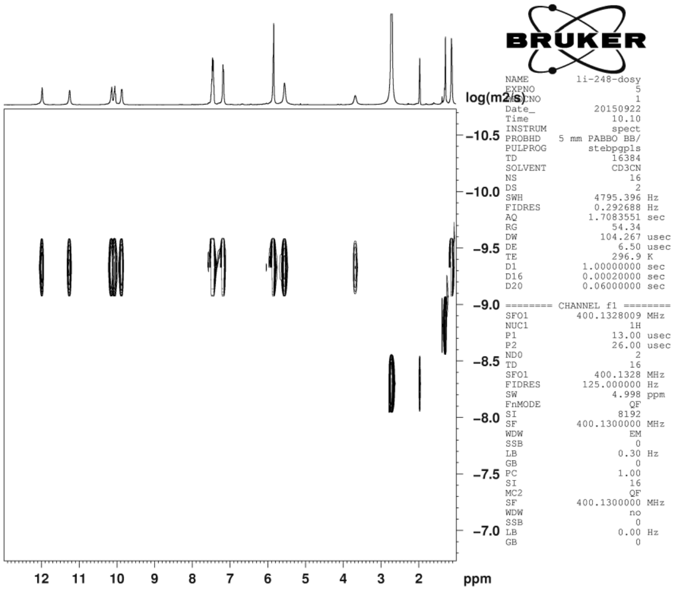

Diffusion Constant = 4.198E-10m^2/S   d = 2.9 nm

Supplementary Figure 67. ^{1}H DOSY spectrum of **$[Pr_4(L^{1S})_4](ClO_4)_{12}$** ($CD_3CN$, 400 MHz, 298 K).

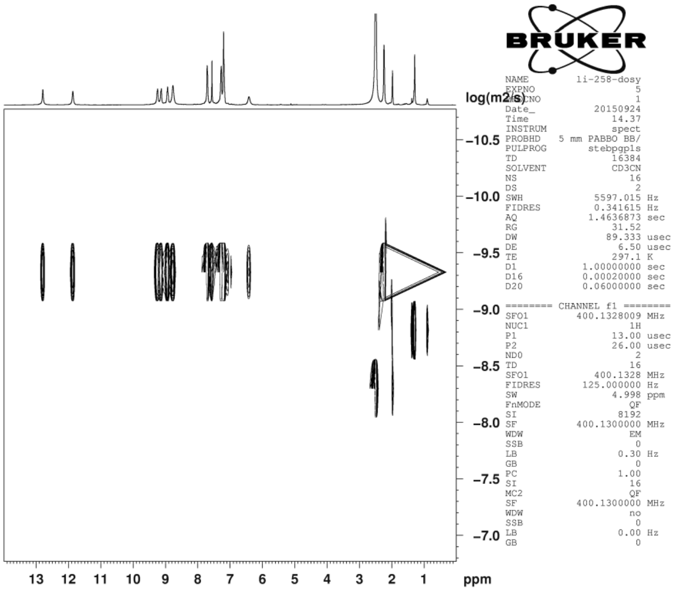

Diffusion Constant = 4.198E-10m^2/S   d = 2.9 nm

Supplementary Figure 68. ^{1}H DOSY spectrum of **[Nd$_4$(L^{1S})$_4$](ClO$_4$)$_{12}$** (CD$_3$CN, 400 MHz, 298 K).

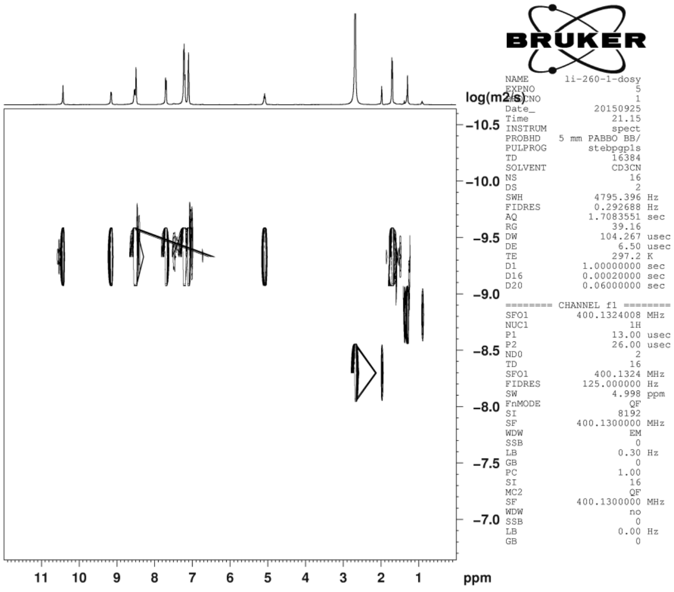

Diffusion Constant = 4.198E-10m^2/S   d = 2.9 nm

Supplementary Figure 69. ^{1}H DOSY spectrum of [**Sm$_4$(L^{1S})$_4$](ClO$_4$)$_{12}$** (CD$_3$CN, 400 MHz, 298 K).

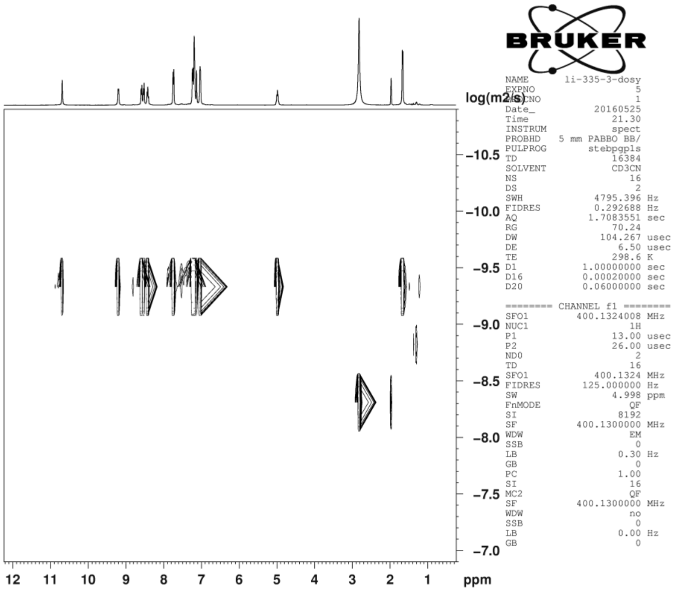

Diffusion Constant = 4.198E-10m^2/S   d = 2.9 nm

Supplementary Figure 70. ^{1}H DOSY spectrum of **[Y$_4$(L^{1S})$_4$](ClO$_4$)$_{12}$** (CD$_3$CN, 400 MHz, 298 K).

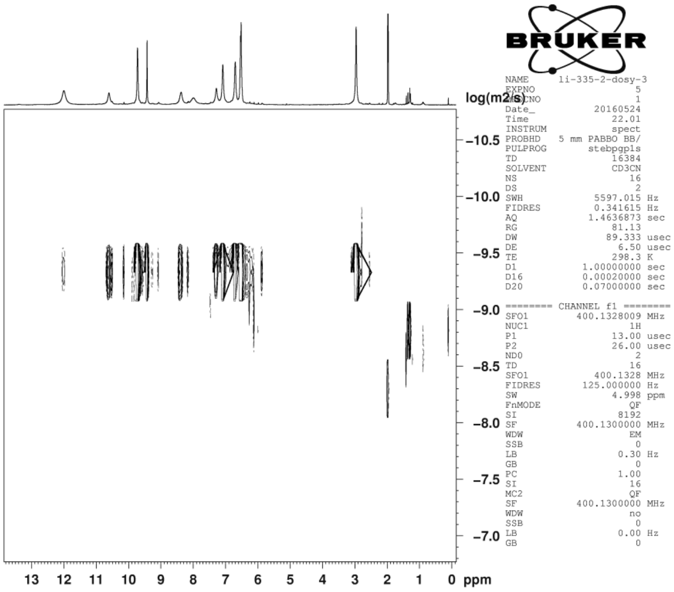

Diffusion Constant = 4.121E-10m^2/S   d = 3.0 nm

Supplementary Figure 71. ^{1}H DOSY spectrum of **[Yb$_4$(L^{1S})$_4$](CF$_3$SO$_3$)$_{12}$** (CD$_3$CN, 400 MHz, 298 K).

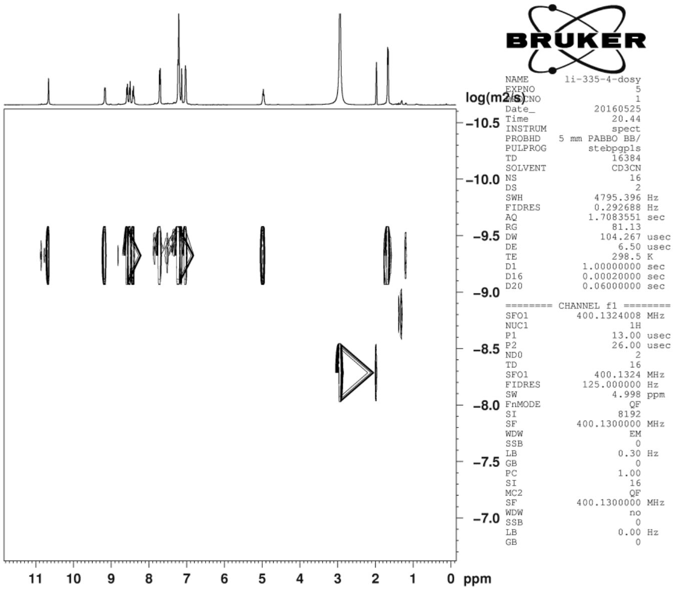

Diffusion Constant = 4.198E-10m^2/S d = 2.9 nm

Supplementary Figure 72. ^{1}H DOSY spectrum of **[Lu$_4$(L^{1S})$_4$](CF$_3$SO$_3$)$_{12}$** (CD$_3$CN, 400 MHz, 298 K).

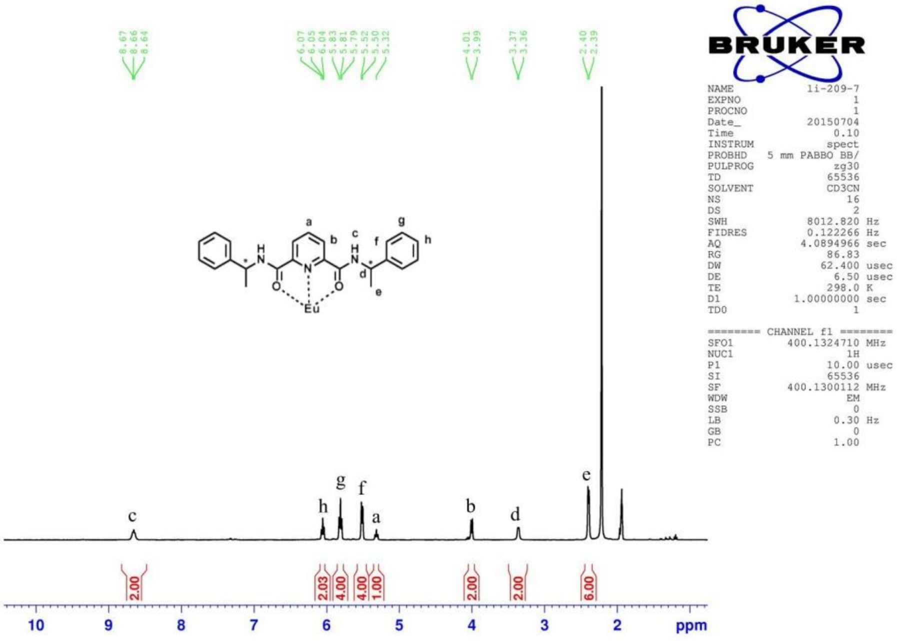

Supplementary Figure 73. ^{1}H NMR spectrum of **[Eu$_1$(L^{2S})$_3$](CF$_3$SO$_3$)$_3$** (CD$_3$CN, 400 MHz, 298 K).

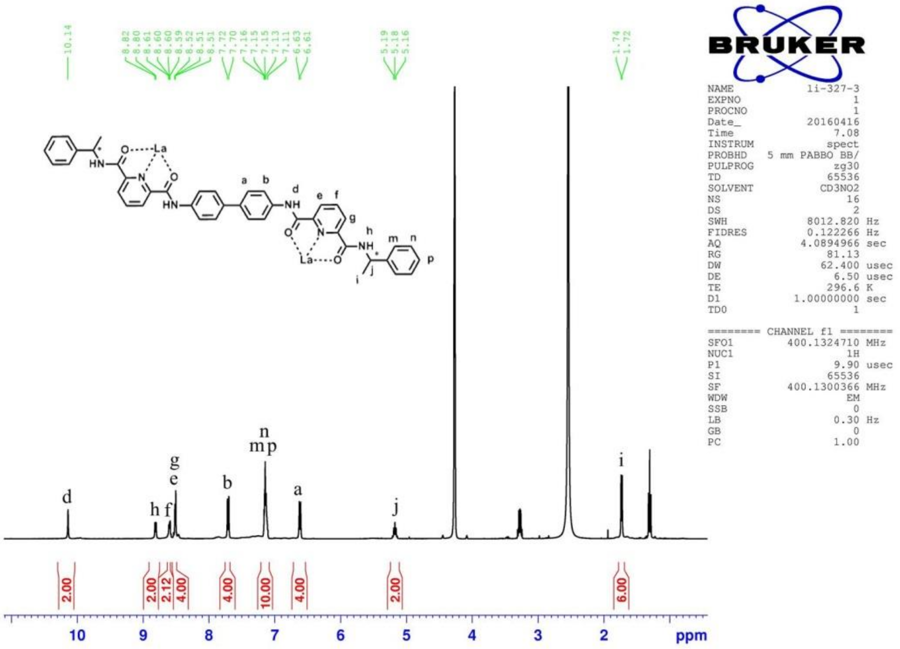

Supplementary Figure 74. ^{1}H NMR spectrum of $\mathbf{[La_2(L^{3S})_3](ClO_4)_6}$ (CD_3CN, 400 MHz, 298 K).

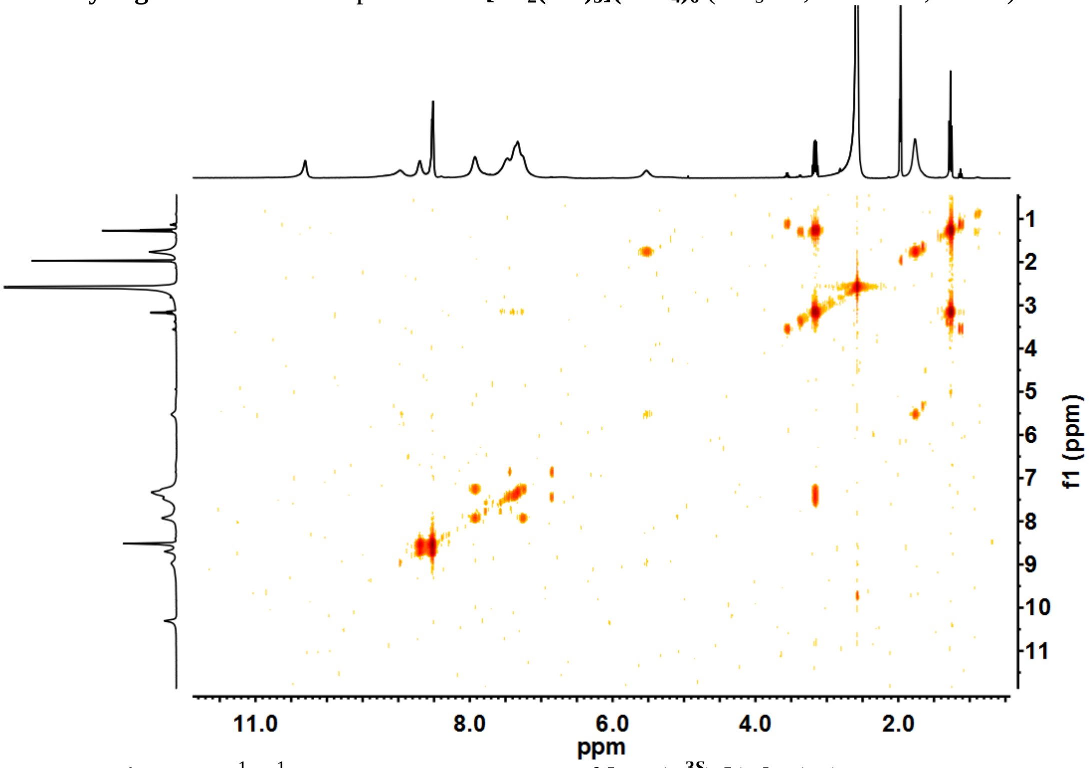

Supplementary Figure 75. ^{1}H-^{1}H COSY NMR spectrum of $\mathbf{[La_2(L^{3S})_3](ClO_4)_6}$ (CD_3CN, 400 MHz, 298 K).

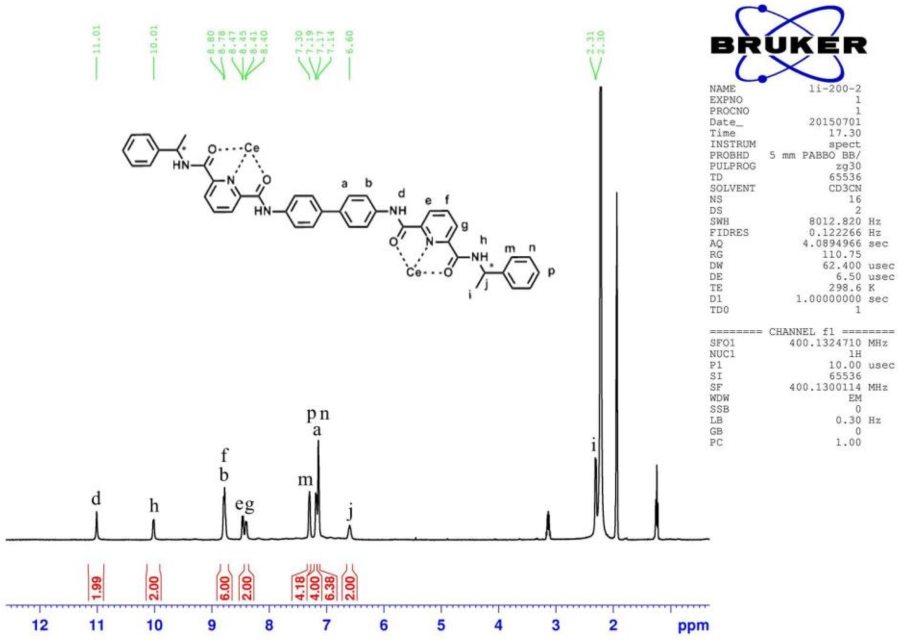

Supplementary Figure 76. ^{1}H NMR spectrum of **[$Ce_2(L^{3S})_3](CF_3SO_3)_6$** ($CD_3CN$, 400 MHz, 298 K).

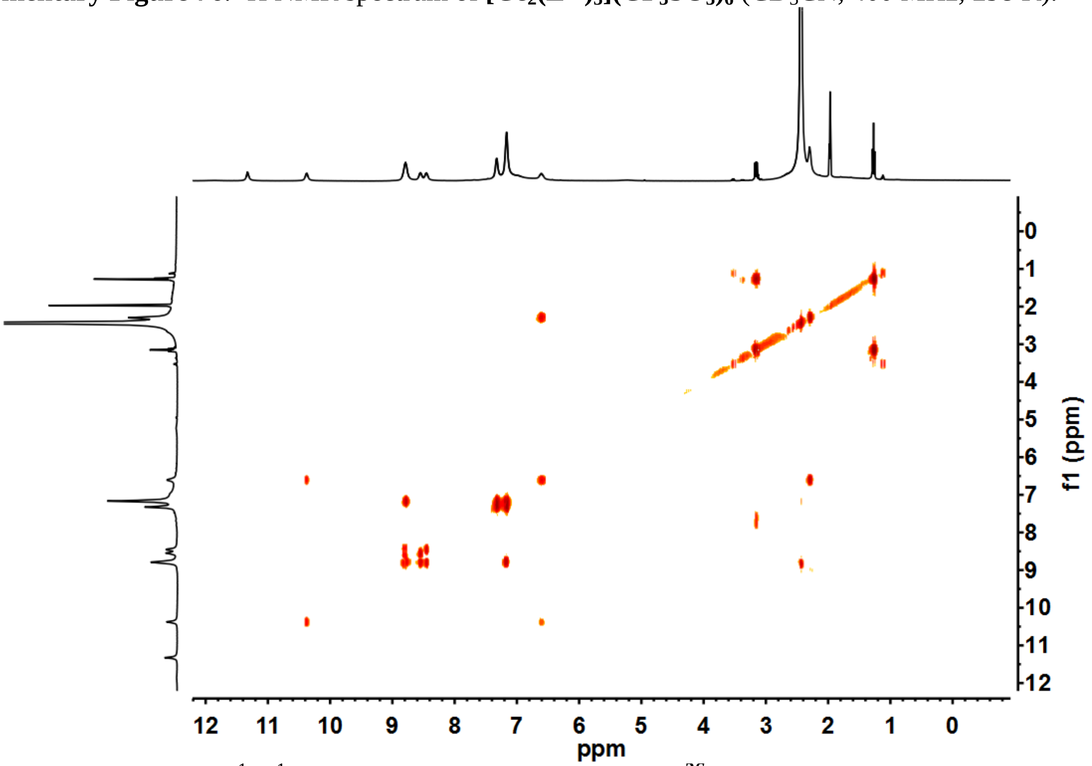

Supplementary Figure 77. ^{1}H-^{1}H COSY NMR spectrum of **[$Ce_2(L^{3S})_3](CF_3SO_3)_6$** ($CD_3CN$, 400 MHz, 298 K).

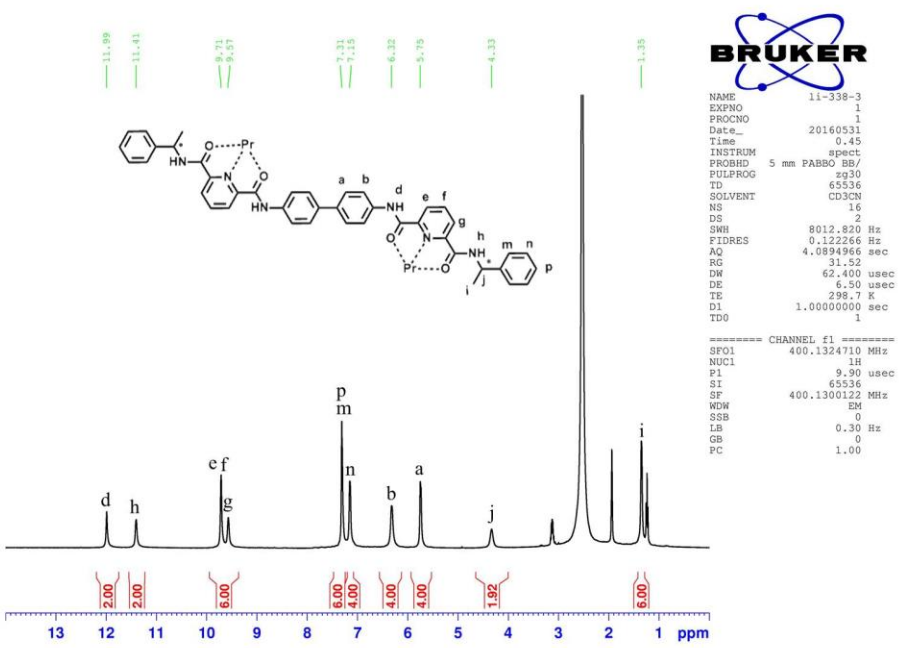

Supplementary Figure 78. ^{1}H NMR spectrum of $\mathbf{[Pr_2(L^{3S})_3](ClO_4)_6}$ (CD_3CN, 400 MHz, 298 K).

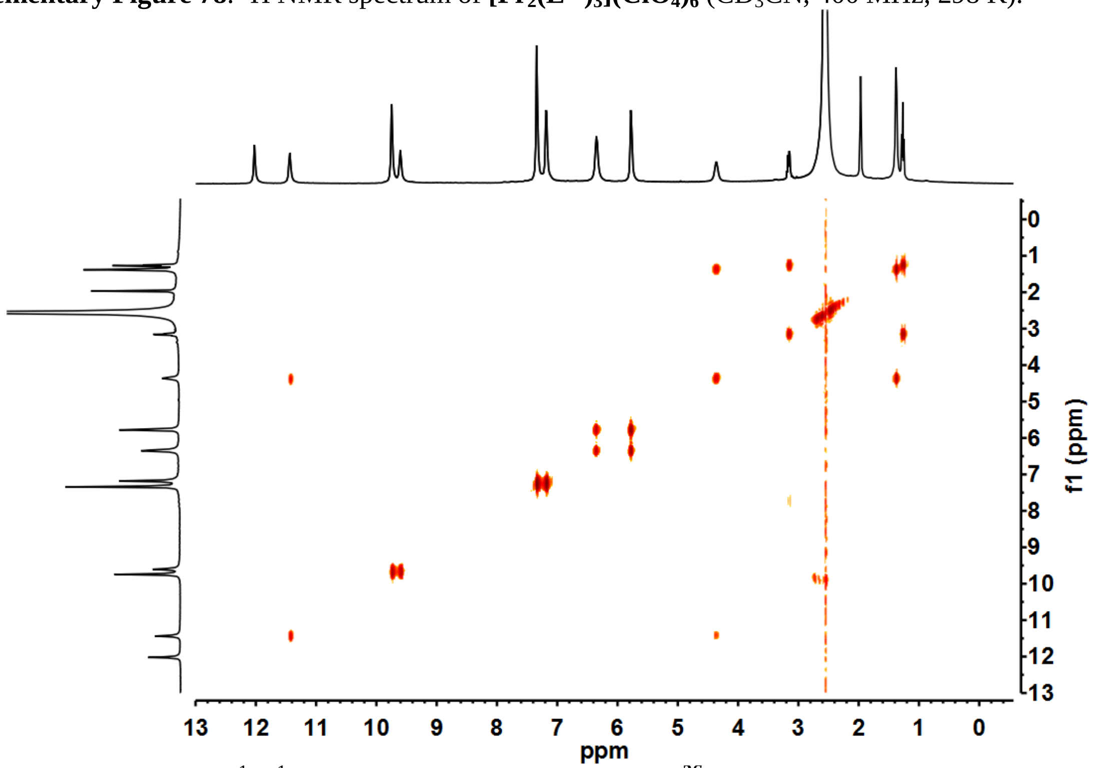

Supplementary Figure 79. ^{1}H-^{1}H COSY NMR spectrum of $\mathbf{[Pr_2(L^{3S})_3](ClO_4)_6}$ (CD_3CN, 400 MHz, 298 K).

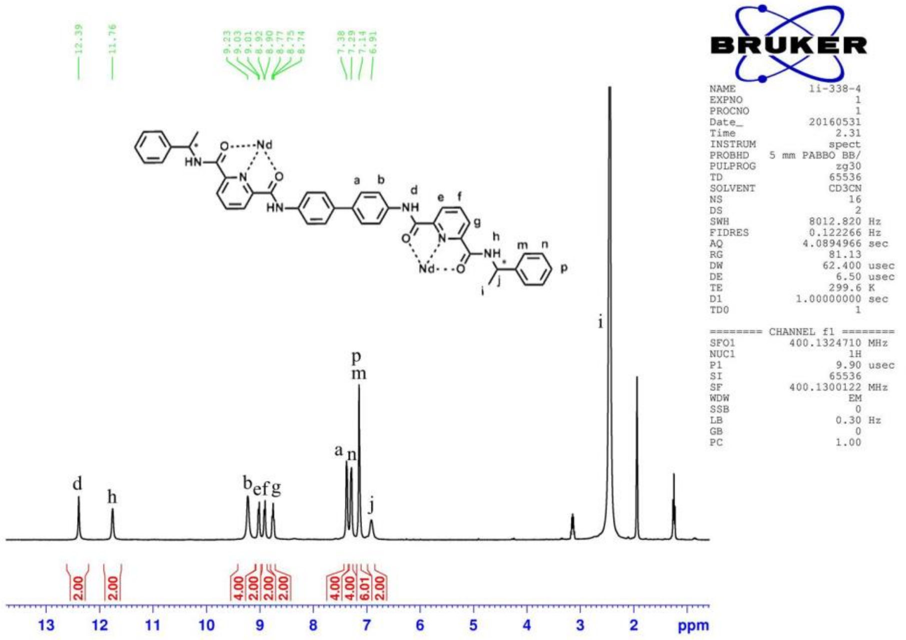

Supplementary Figure 80. ^{1}H NMR spectrum of **$[Nd_2(L^{3S})_3](ClO_4)_6$** ($CD_3CN$, 400 MHz, 298 K).

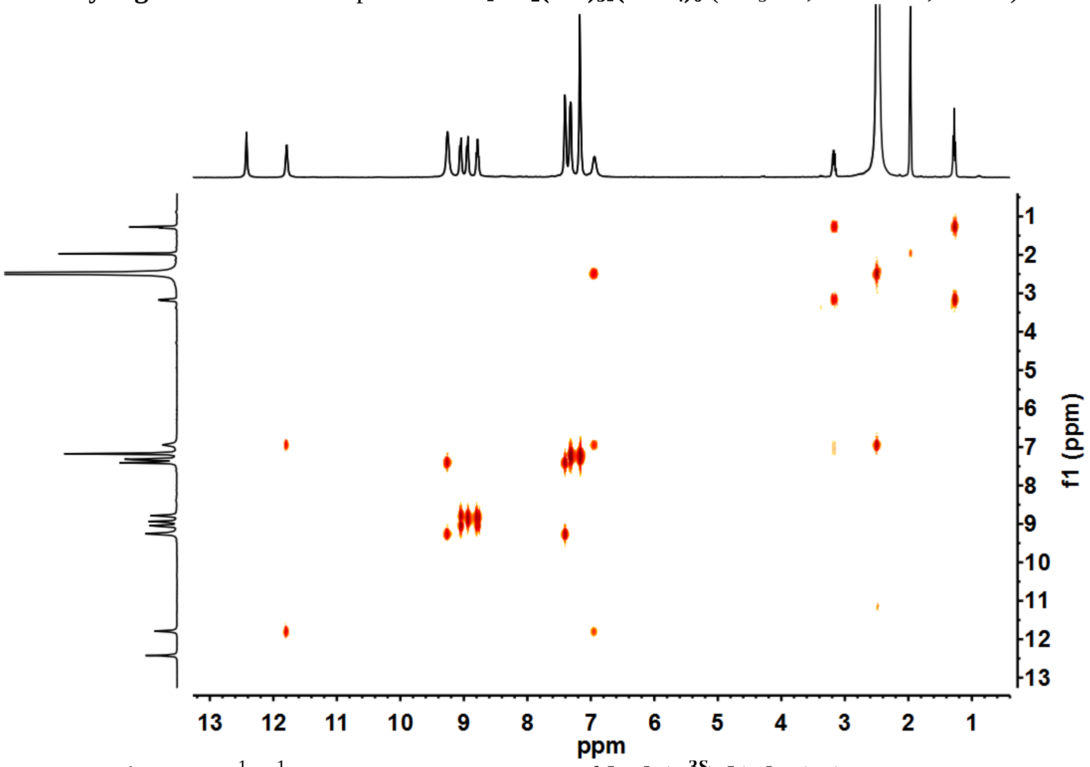

Supplementary Figure 81. ^{1}H-^{1}H COSY NMR spectrum of **$[Nd_2(L^{3S})_3](ClO_4)_6$** ($CD_3CN$, 400 MHz, 298 K).

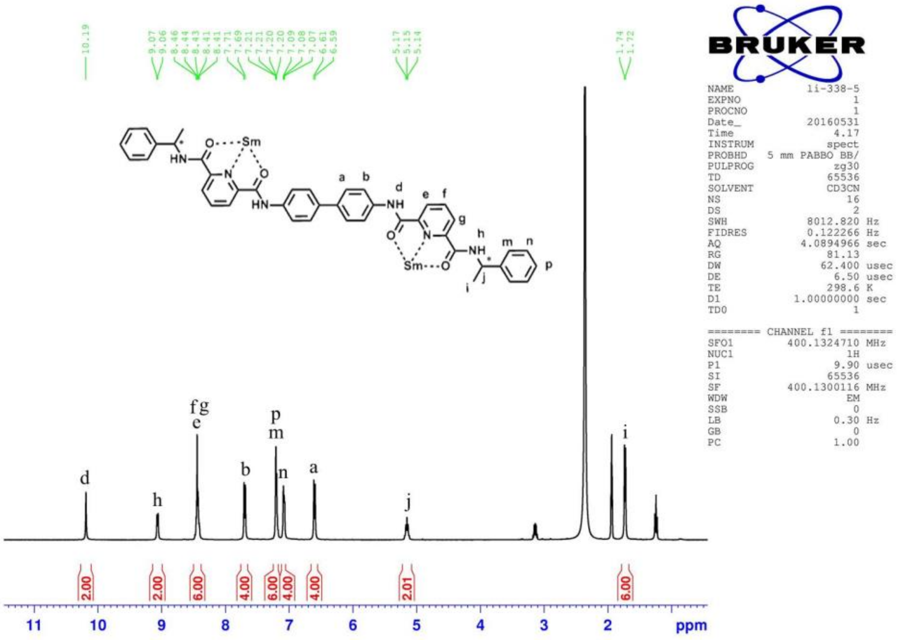

Supplementary Figure 82. ^{1}H NMR spectrum of **$[Sm_2(L^{3S})_3](ClO_4)_6$** ($CD_3CN$, 400 MHz, 298 K).

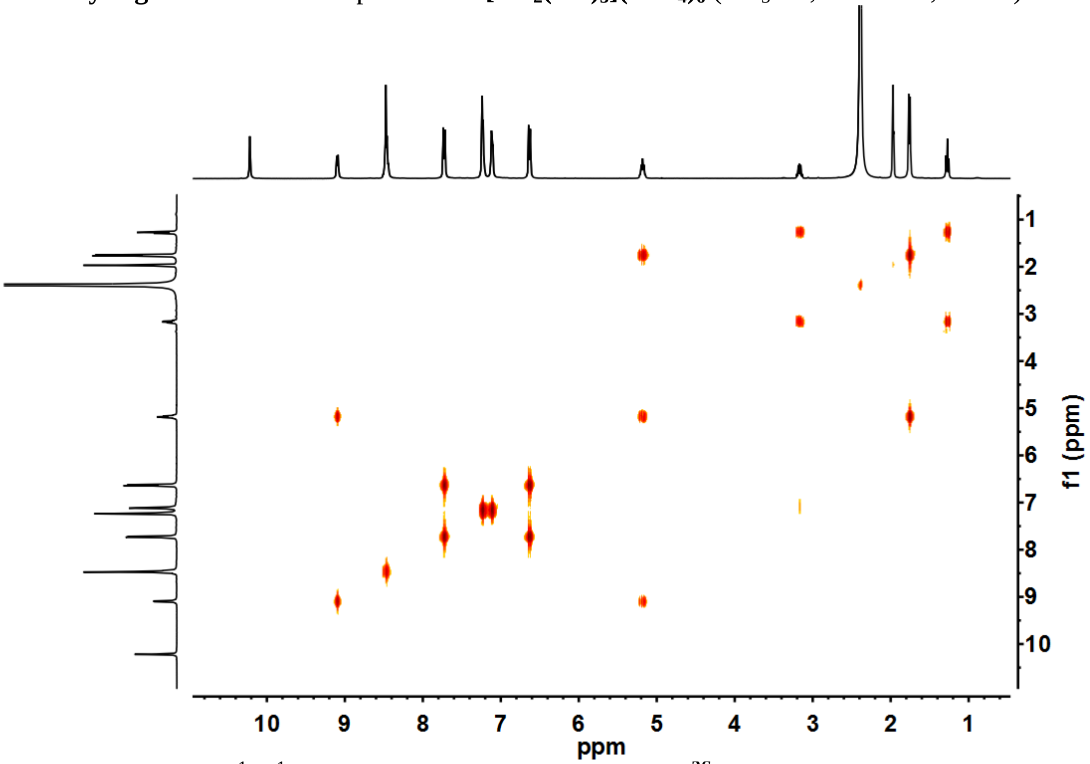

Supplementary Figure 83. ^{1}H-^{1}H COSY NMR spectrum of **$[Sm_2(L^{3S})_3](ClO_4)_6$** ($CD_3CN$, 400 MHz, 298 K).

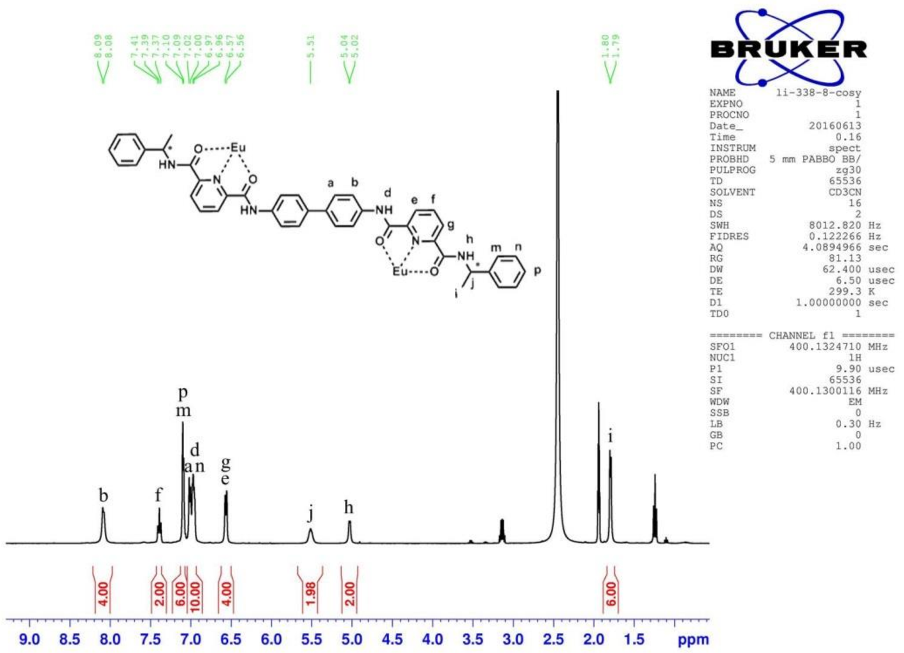

Supplementary Figure 84. ^{1}H NMR spectrum of **$[Eu_2(L^{3S})_3](ClO_4)_6$** ($CD_3CN$, 400 MHz, 298 K).

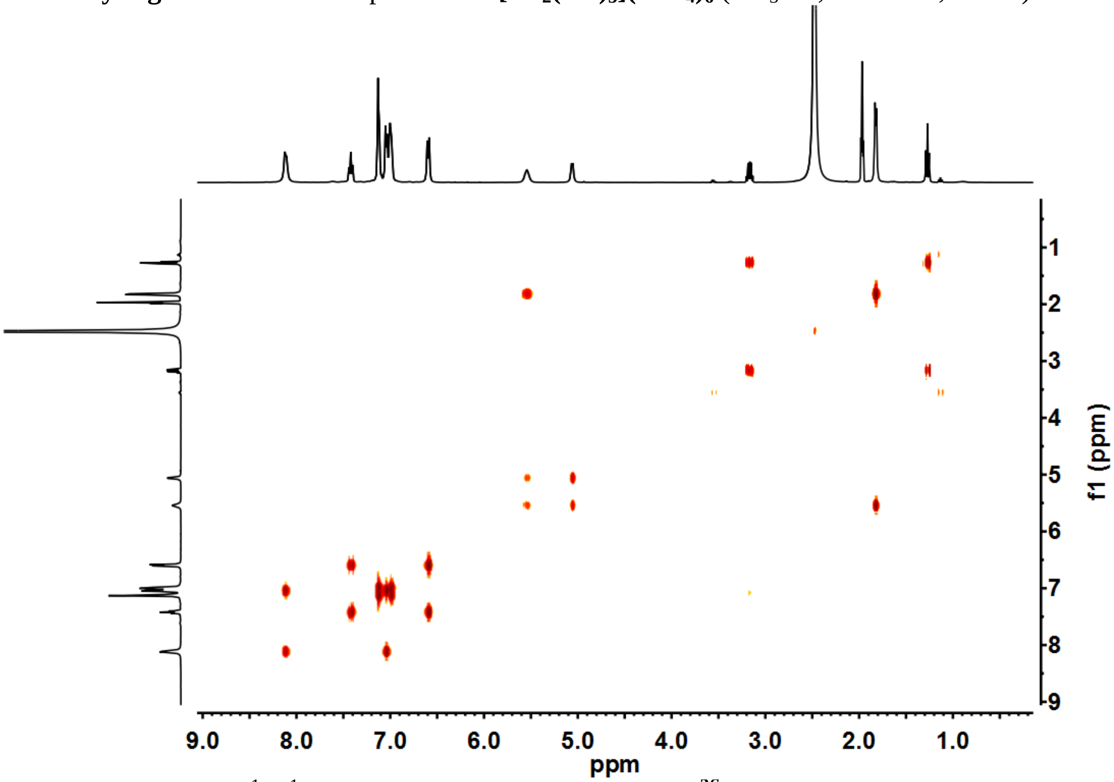

Supplementary Figure 85. ^{1}H-^{1}H COSY NMR spectrum of **$[Eu_2(L^{3S})_3](ClO_4)_6$** ($CD_3CN$, 400 MHz, 298 K).

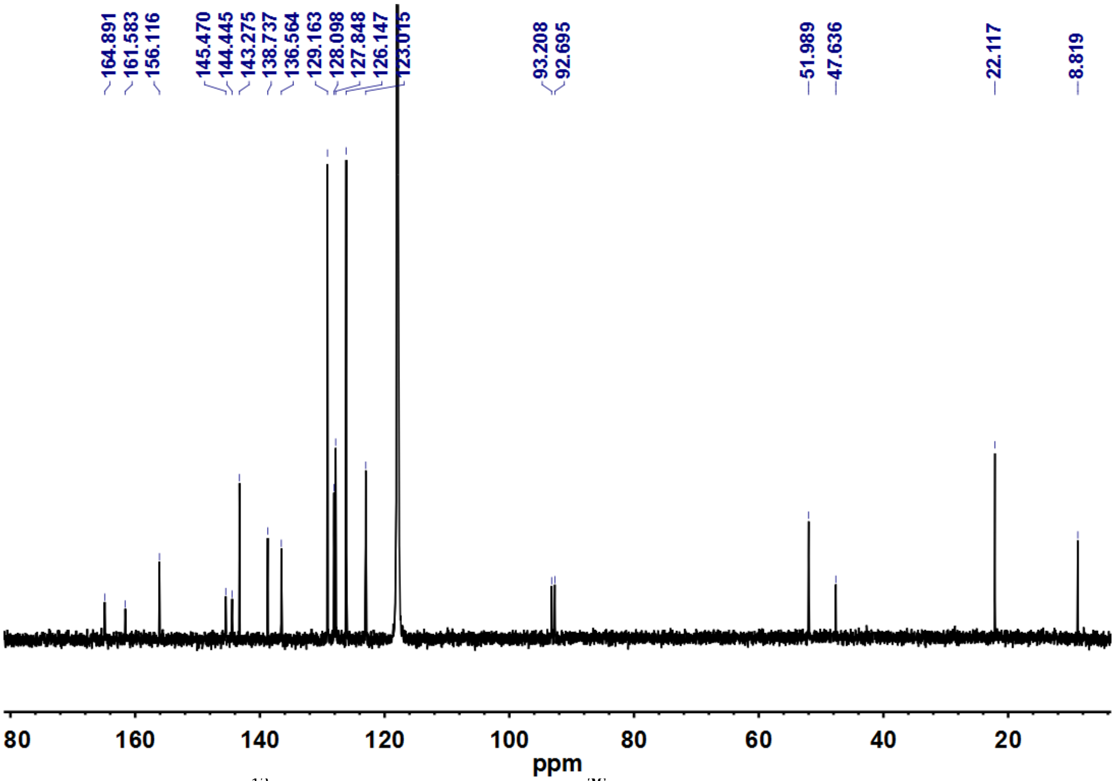

Supplementary Figure 86. ^{13}C NMR spectrum of **$[Eu_2(L^{3S})_3](ClO_4)_6$** ($CD_3CN$, 101 MHz, 298 K).

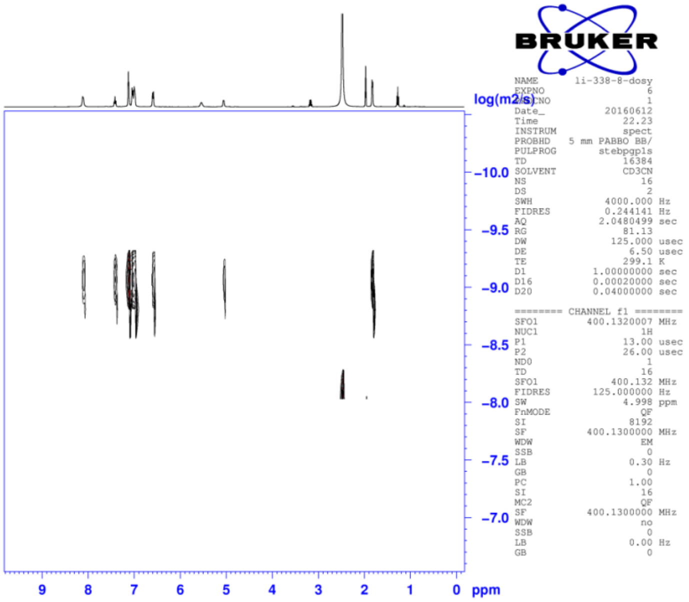

Diffusion Constant = 7.534E-10m^2/S   d = 1.6 nm

Supplementary Figure 87. **^{1}H** DOSY spectrum of **$[Eu_2(L^{3S})_3](ClO_4)_6$** ($CD_3CN$, 400 MHz, 298 K).

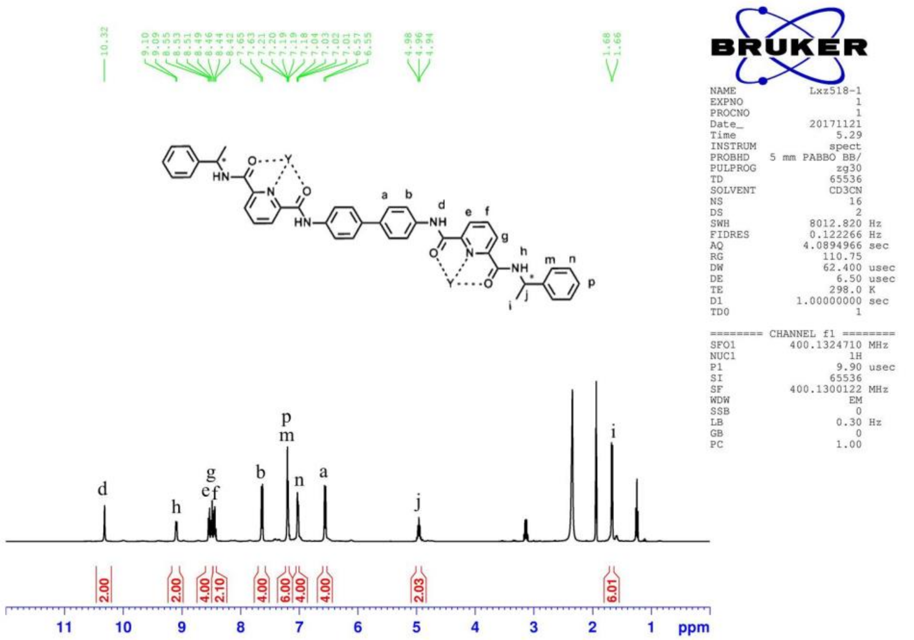

Supplementary Figure 88. ^{1}H NMR spectrum of **$[Y_2(L^{3S})_3](CF_3SO_3)_6$** ($CD_3CN$, 400 MHz, 298 K).

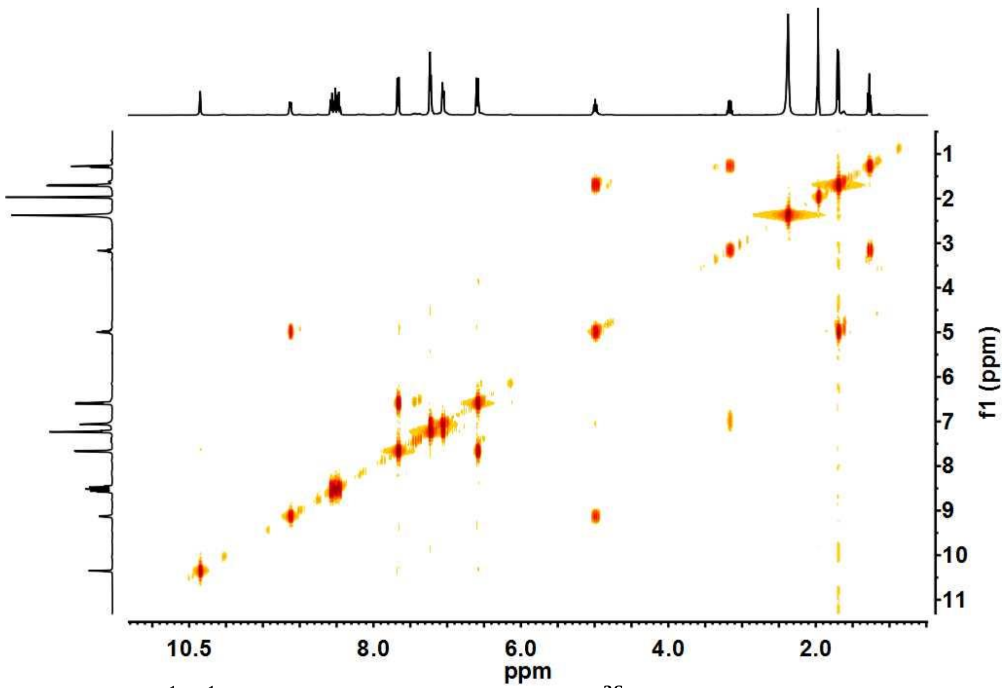

Supplementary Figure 89. ^{1}H-^{1}H COSY NMR spectrum of **$[Y_2(L^{3S})_3](CF_3SO_3)_6$** ($CD_3CN$, 400 MHz, 298 K).

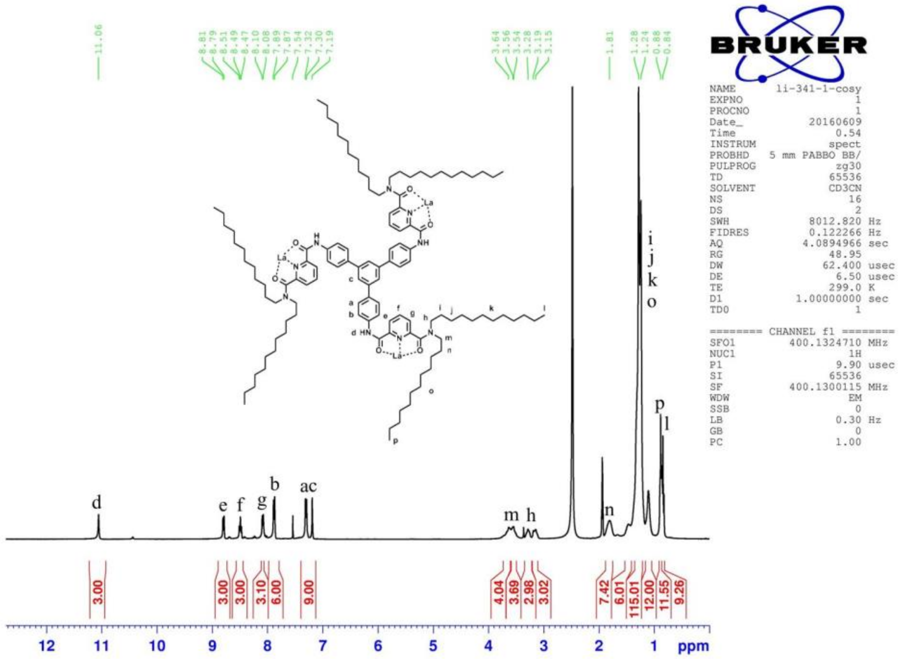

Supplementary Figure 90. ^{1}H NMR spectrum of **$[La_4(L^6)_4](CF_3SO_3)_{12}$** ($CD_3CN/CDCl_3$, 400 MHz, 298 K).

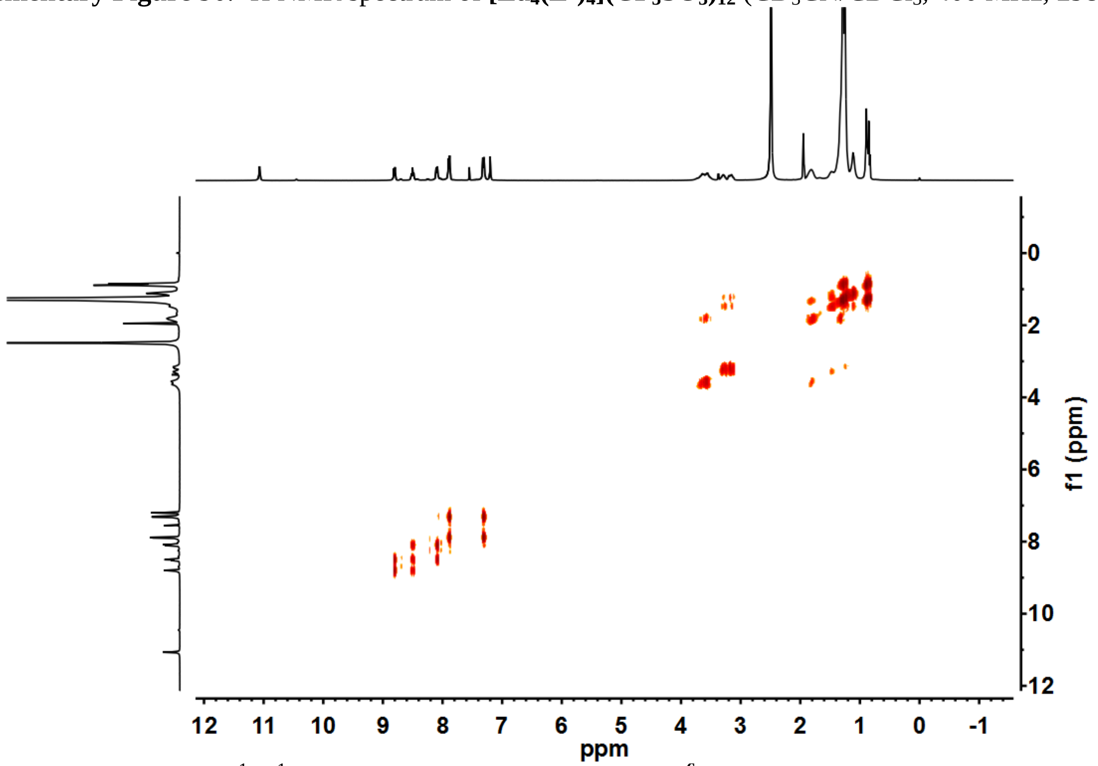

Supplementary Figure 91. ^{1}H-^{1}H COSY NMR spectrum of **$[La_4(L^6)_4](CF_3SO_3)_{12}$** ($CD_3CN/CDCl_3$, 400 MHz, 298 K).

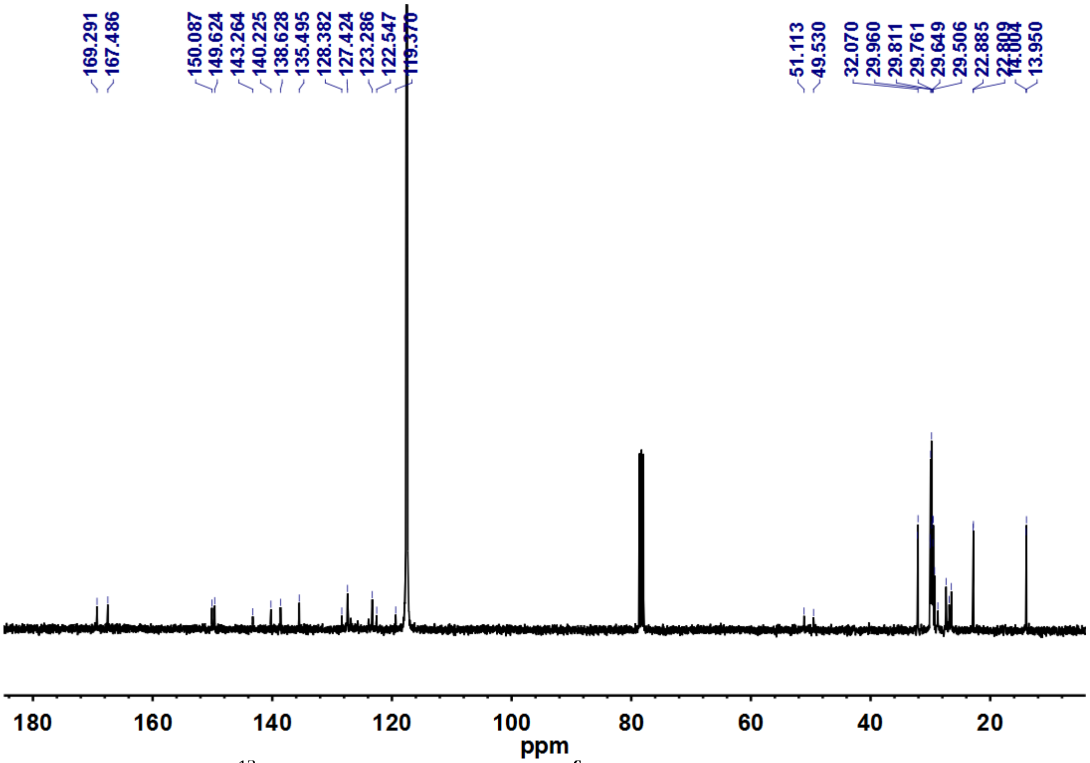

Supplementary Figure 92. ^{13}C NMR spectrum of **$[La_4(L^6)_4](CF_3SO_3)_{12}$** ($CD_3CN/CDCl_3$, 101 MHz, 298 K).

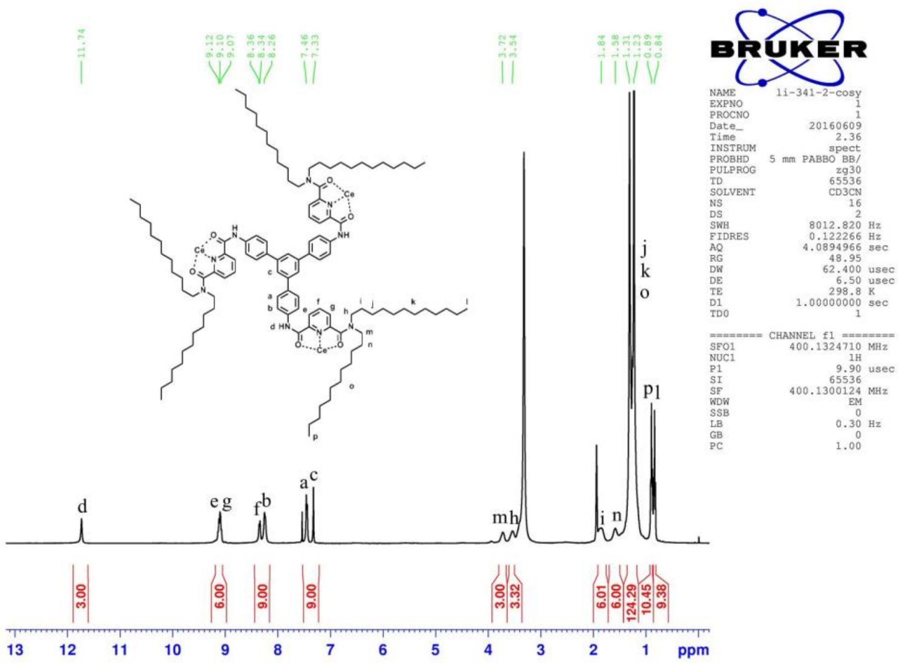

Supplementary Figure 93. ^{1}H NMR spectrum of **$[Ce_4(L^6)_4](CF_3SO_3)_{12}$** ($CD_3CN/CDCl_3$, 400 MHz, 298 K).

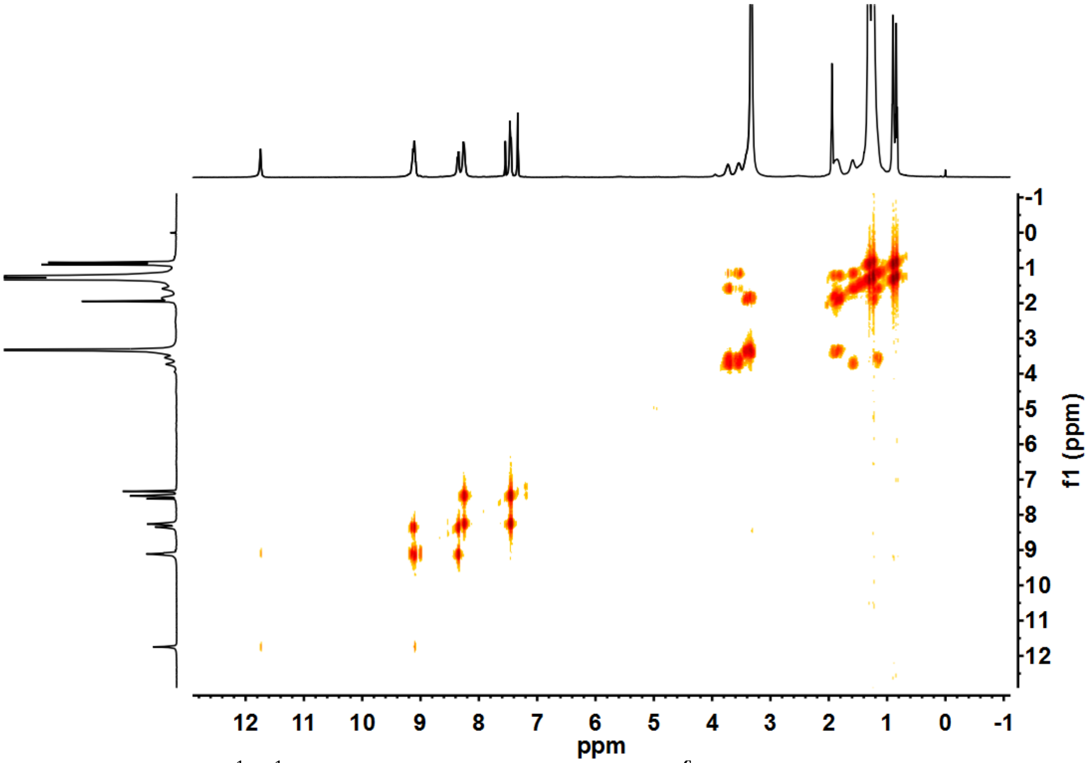

Supplementary Figure 94. ^{1}H-^{1}H COSY NMR spectrum of **[Ce$_4$(L^6)$_4$](CF$_3$SO$_3$)$_{12}$** (CD$_3$CN/CDCl$_3$, 400 MHz, 298 K).

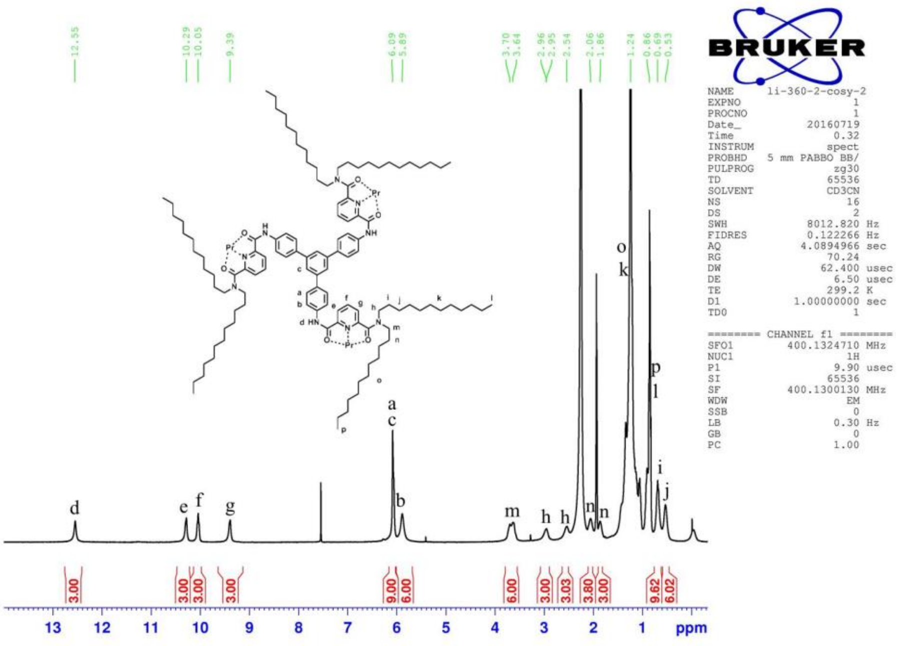

Supplementary Figure 95. ^{1}H NMR spectrum of **[Pr$_4$(L^6)$_4$](CF$_3$SO$_3$)$_{12}$** (CD$_3$CN/CDCl$_3$, 400 MHz, 298 K).

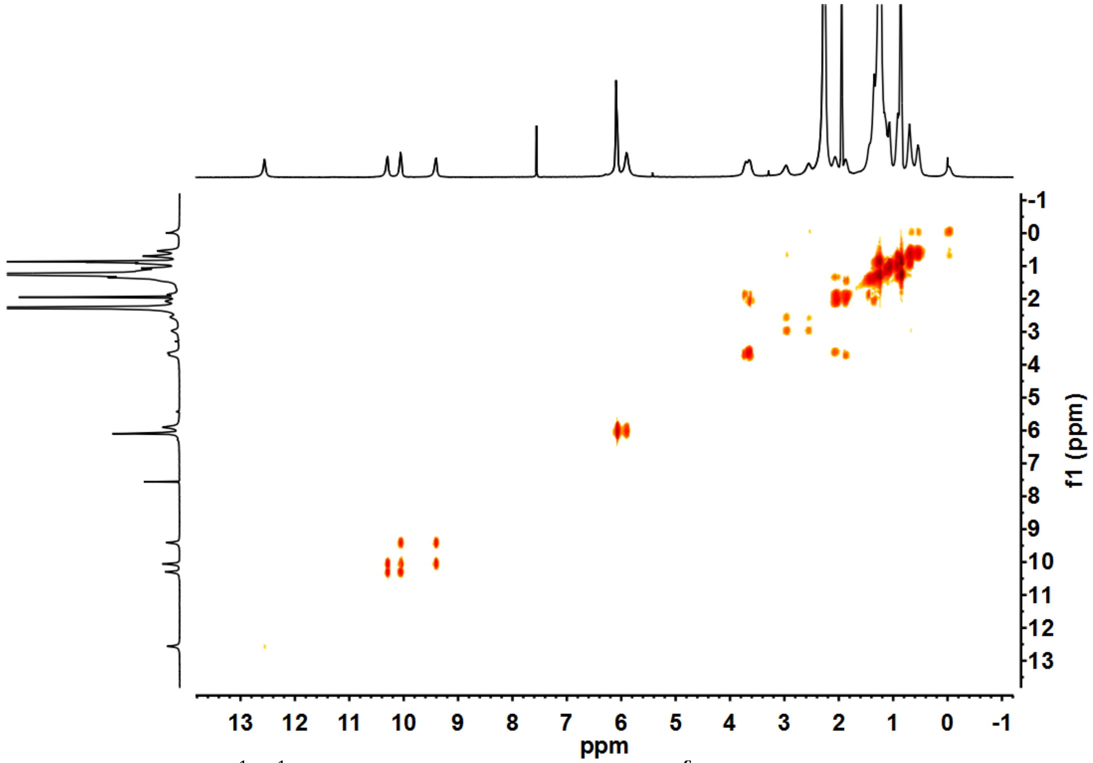

Supplementary Figure 96. ¹H-¹H COSY NMR spectrum of **[$Pr_4(L^6)_4$](CF_3SO_3)$_{12}$** ($CD_3CN/CDCl_3$, 400 MHz, 298 K).

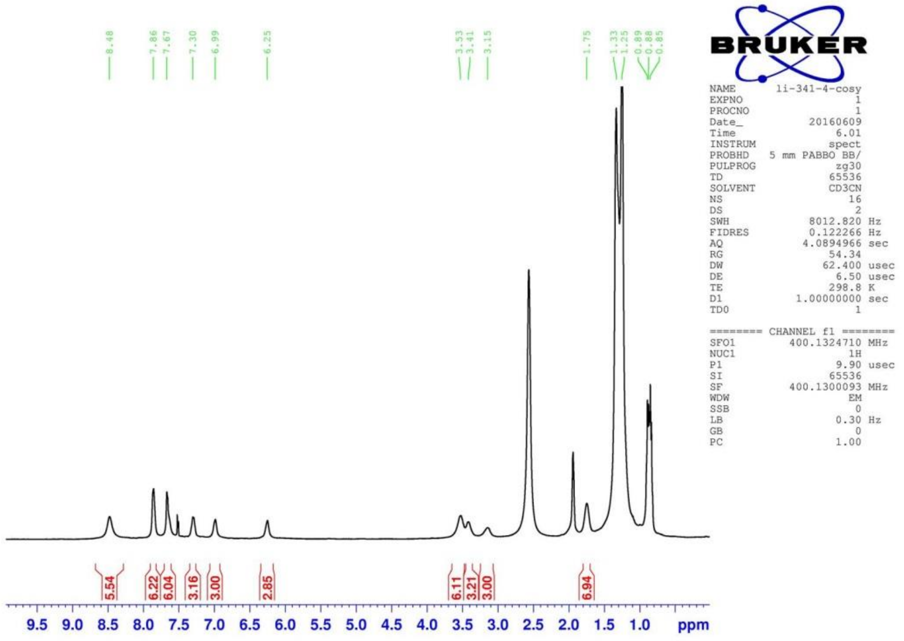

Supplementary Figure 97. ¹H NMR spectrum of **[$Eu_4(L^6)_4$](CF_3SO_3)$_{12}$** ($CD_3CN/CDCl_3$, 400 MHz, 298 K).

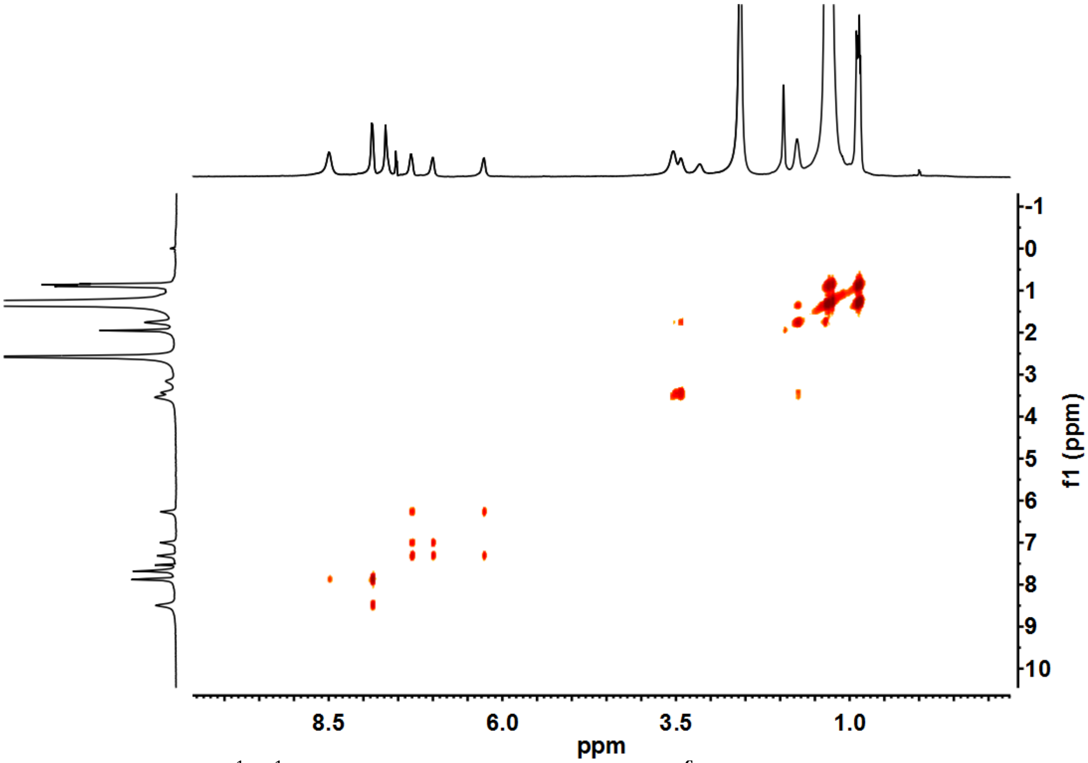

Supplementary Figure 98. ^{1}H-^{1}H COSY NMR spectrum of **[Eu$_4$(L^6)$_4$](CF$_3$SO$_3$)$_{12}$** (CD$_3$CN/CDCl$_3$, 400 MHz, 298 K).

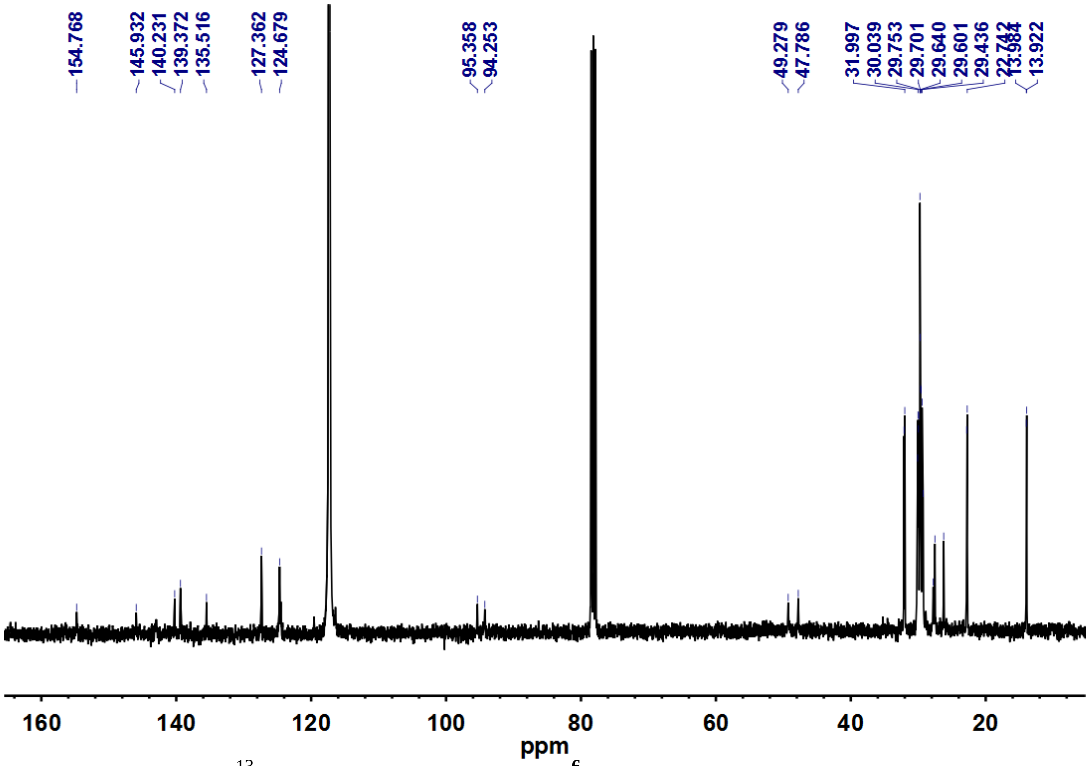

Supplementary Figure 99. ^{13}C NMR spectrum of **[Eu$_4$(L^6)$_4$](CF$_3$SO$_3$)$_{12}$** (CD$_3$CN/CDCl$_3$, 101 MHz, 298 K).

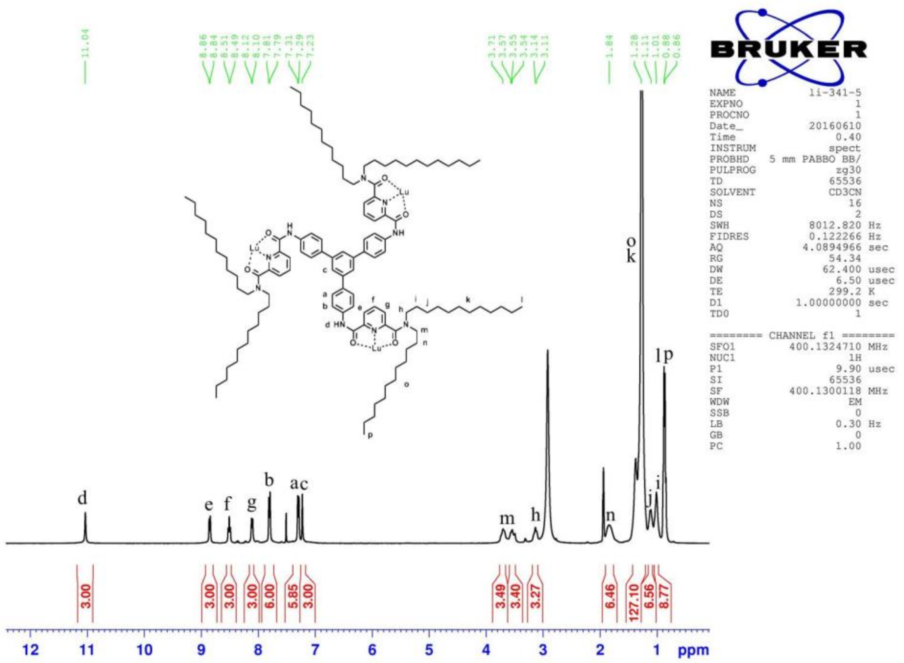

Supplementary Figure 100. ^{1}H NMR spectrum of **$[Lu_4(L^6)_4](CF_3SO_3)_{12}$** ($CD_3CN/CDCl_3$, 400 MHz, 298 K).

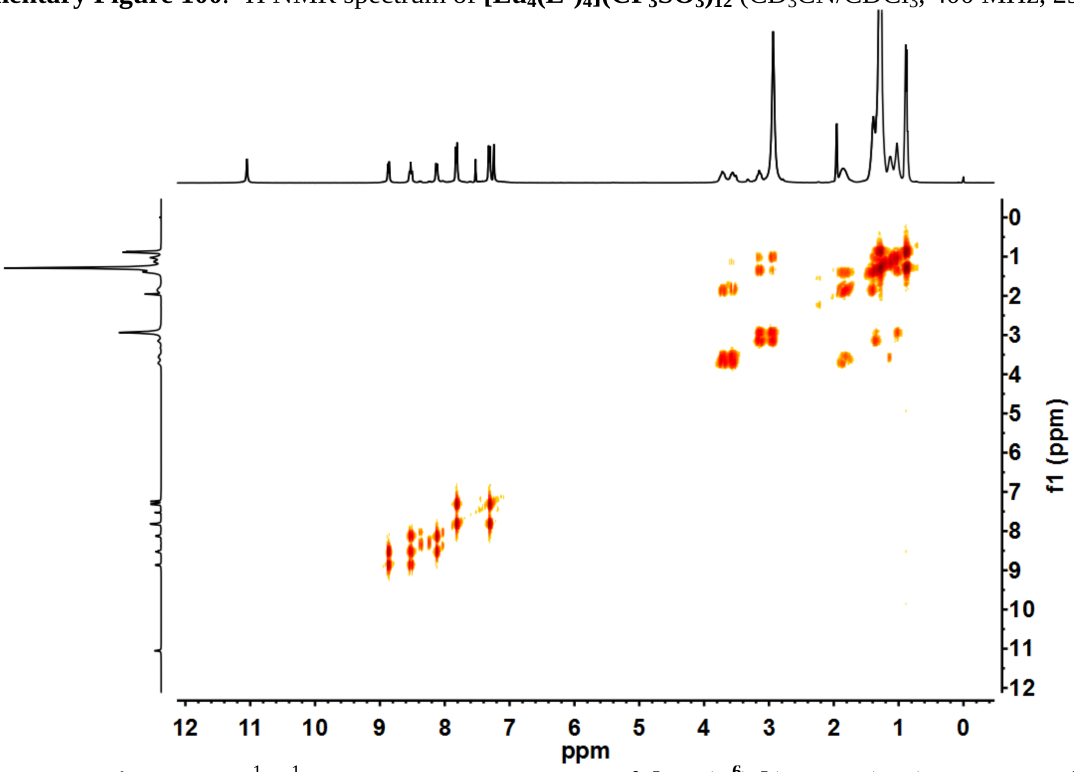

Supplementary Figure 101. ^{1}H-^{1}H COSY NMR spectrum of **$[Lu_4(L^6)_4](CF_3SO_3)_{12}$** ($CD_3CN/CDCl_3$, 400 MHz, 298 K).

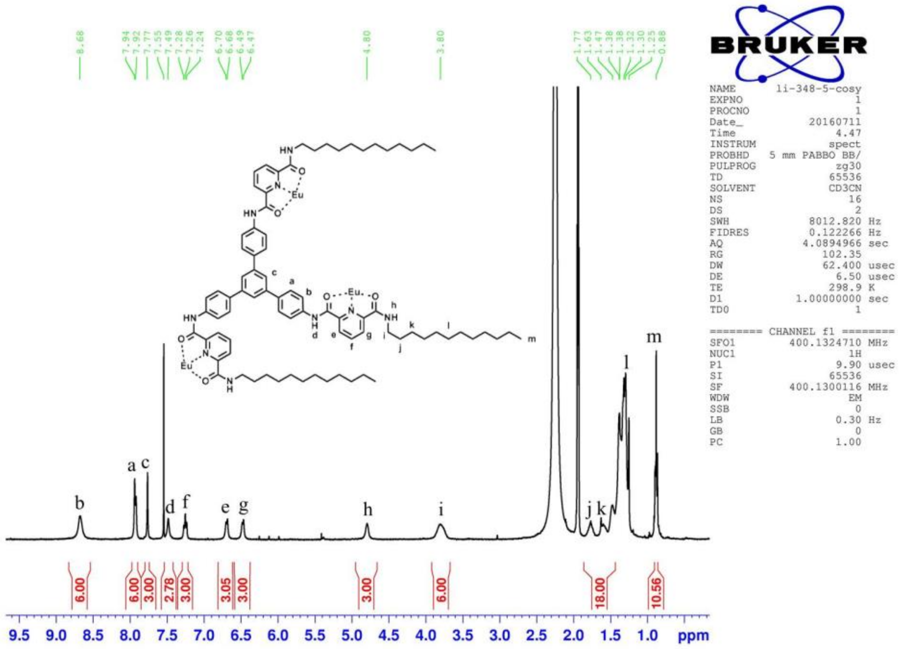

Supplementary Figure 102. ^{1}H NMR spectrum of **$[Eu_4(L^4)_4](CF_3SO_3)_{12}$** ($CD_3CN/CDCl_3$, 400 MHz, 298 K).

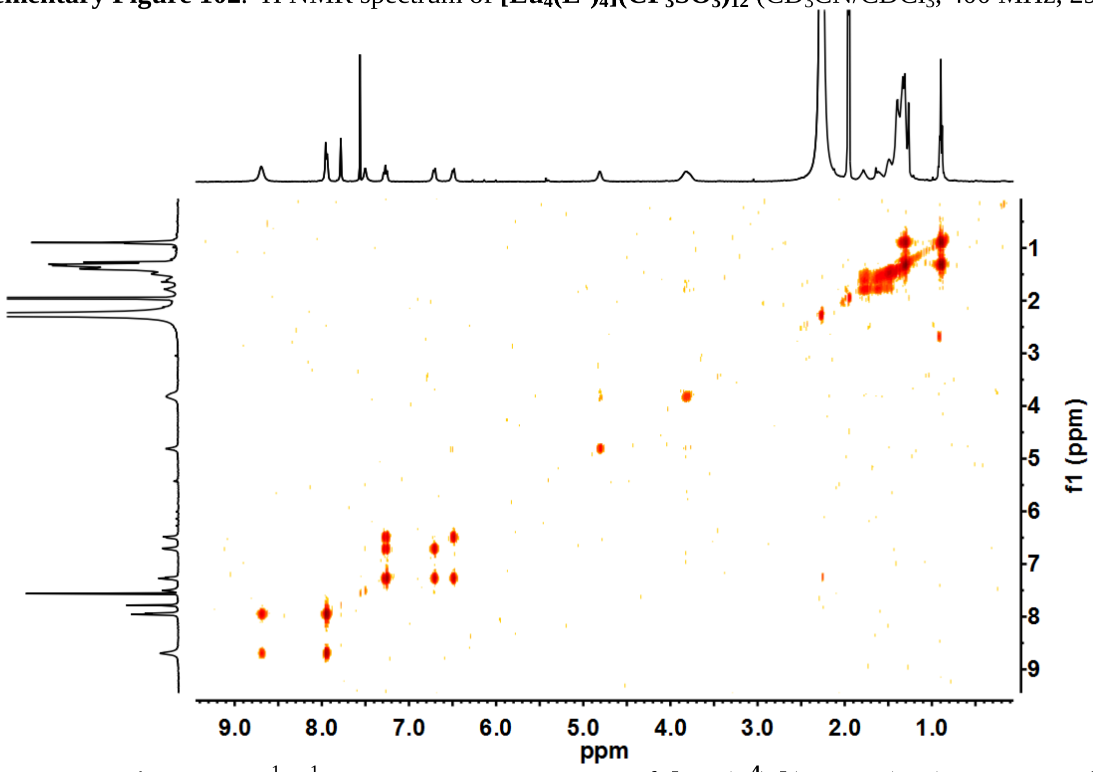

Supplementary Figure 103. ^{1}H-^{1}H COSY NMR spectrum of **$[Eu_4(L^4)_4](CF_3SO_3)_{12}$** ($CD_3CN/CDCl_3$, 400 MHz, 298 K).

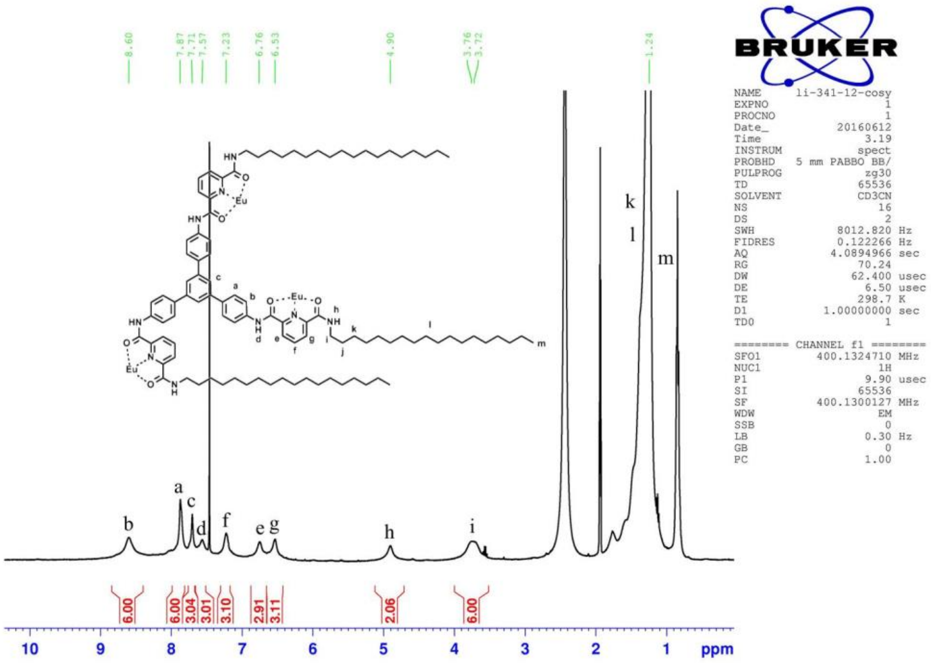

Supplementary Figure 104. ^{1}H NMR spectrum of **[$Eu_4(L^5)_4$](CF_3SO_3)$_{12}$** ($CD_3CN/CDCl_3$, 400 MHz, 298 K).

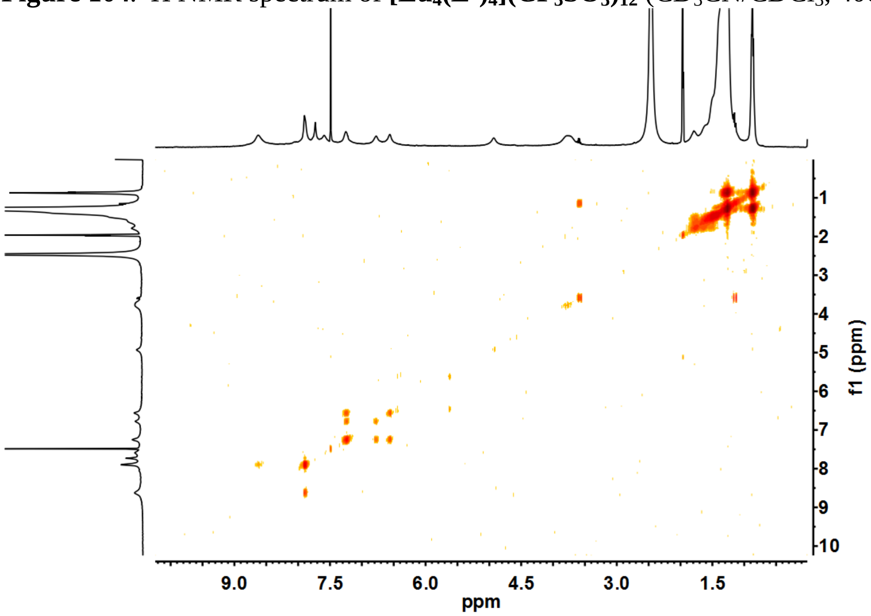

Supplementary Figure 105. ^{1}H-^{1}H COSY NMR spectrum of **[$Eu_4(L^5)_4$](CF_3SO_3)$_{12}$** ($CD_3CN/CDCl_3$, 400 MHz, 298 K)

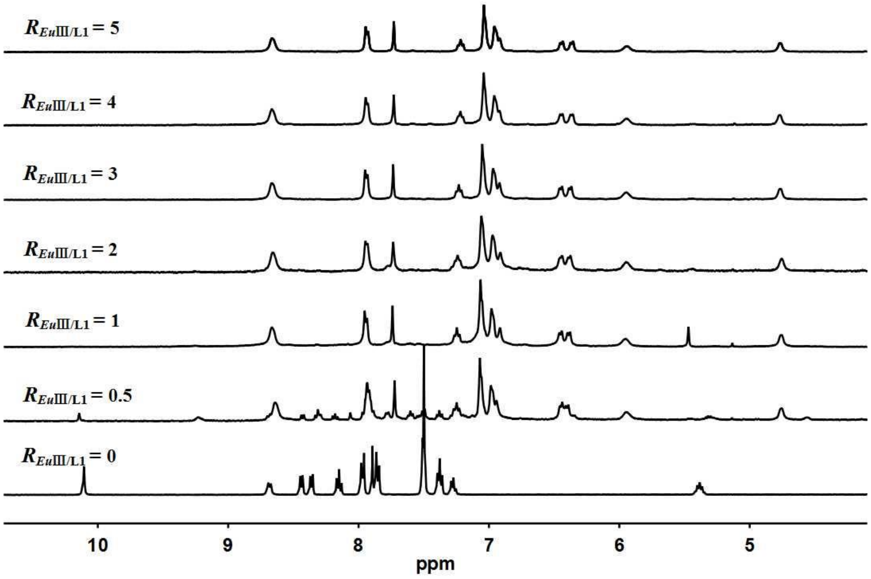

Supplementary Figure 106. ^{1}H NMR spectra for the titration of $\mathbf{L^1}$ (0.002M) with $Eu(ClO_4)_3$ (400 MHz, CD_3CN, 298 K).

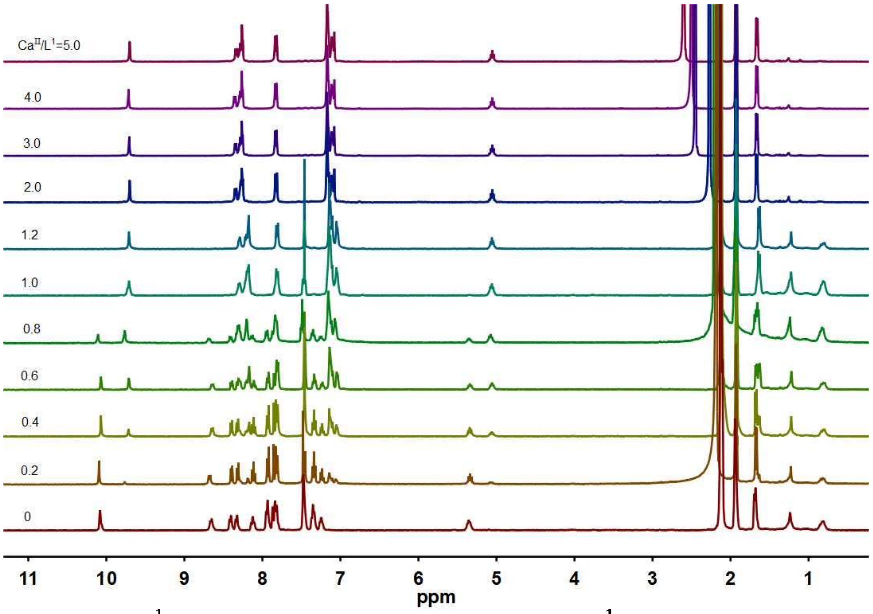

Supplementary Figure 107. ^{1}H NMR spectra for the titration of $\mathbf{L^1}$ (0.002M) with $Ca(ClO_4)_2$ (400 MHz, $CD_3CN/CDCl_3$ for Ca^{II}/L^1=0-1.2 and CD_3CN for Ca^{II}/L^1=2.0-5.0, 298 K).

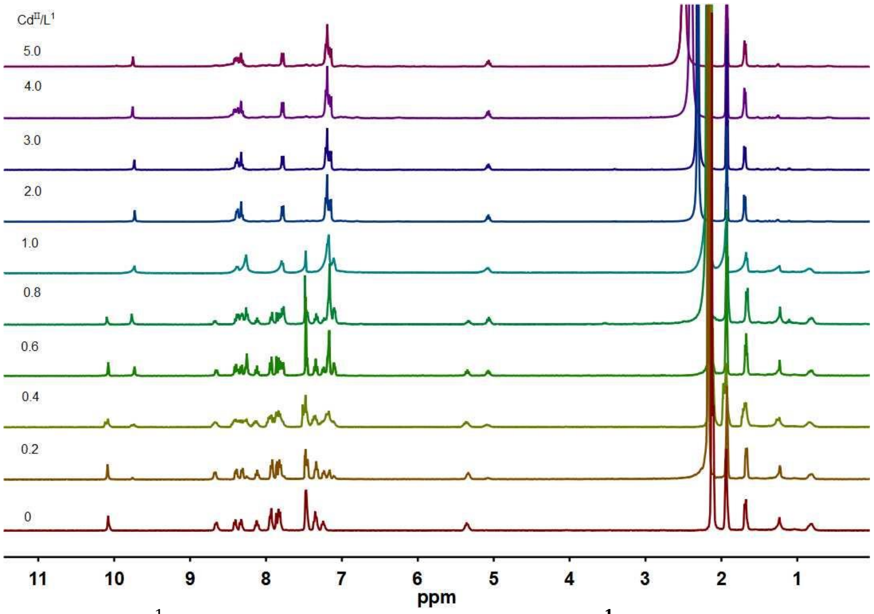

Supplementary Figure 108. ^{1}H NMR spectra for the titration of $\mathbf{L^1}$ (0.002M) with $Cd(ClO_4)_2$ (400 MHz, $CD_3CN/CDCl_3$ for Cd^{II}/L^1=0-1.0 and CD_3CN for Cd^{II}/L^1=2.0-5.0, 298 K).

These titration experiments of L^1 with metal ions (M = Eu^{III}, Ca^{II}, Cd^{II}) confirmed the exclusive formation of $M_4L^1_4$ metal-organic complexes when $R_{M/L1}$= 0 – 1 and the high structrual stability of these tetrahedral complexes in the presence of excess metal ions.

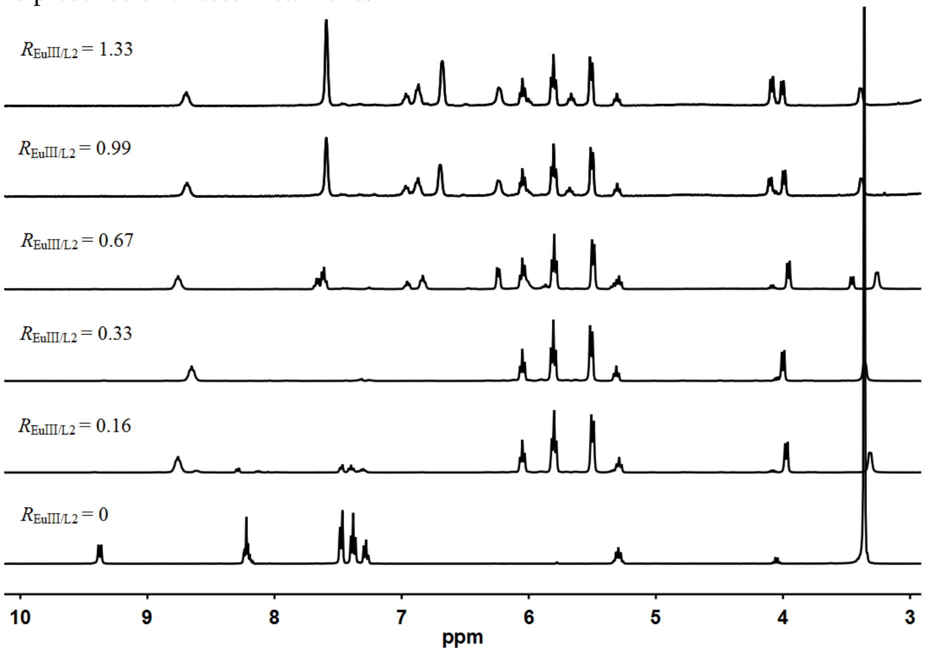

Supplementary Figure 109. ^{1}H NMR spectra for the titration of $\mathbf{L^2}$ (0.005M) with $Eu(ClO_4)_3$ (CD_3CN, 400 MHz, 298 K).

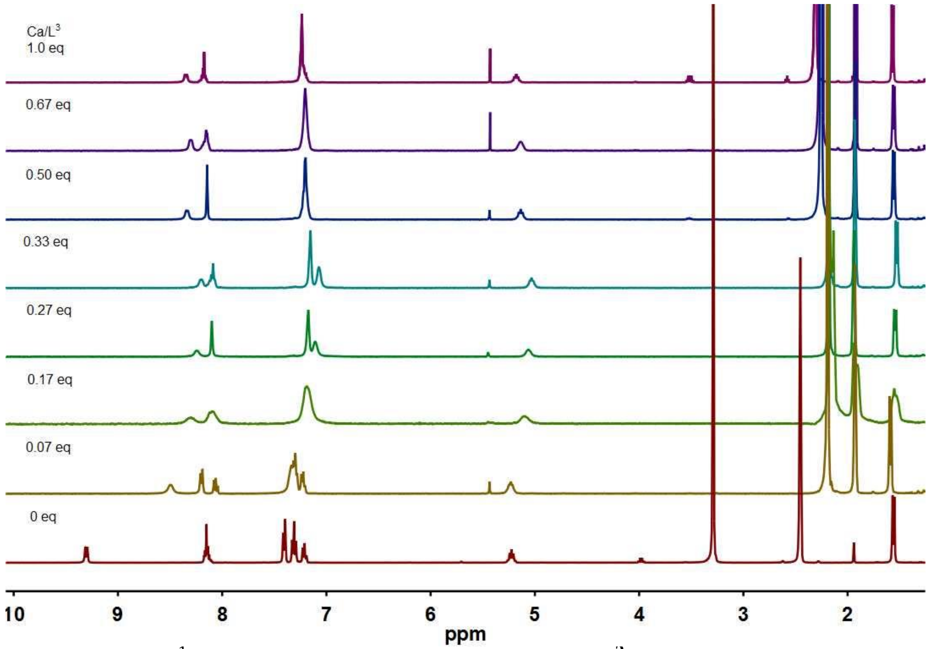

Supplementary Figure 110. ¹H NMR spectra for the titration of $\mathbf{L^2}$ (0.005M) with $Ca(ClO_4)_2$ (CD_3CN, 400 MHz, 298 K).

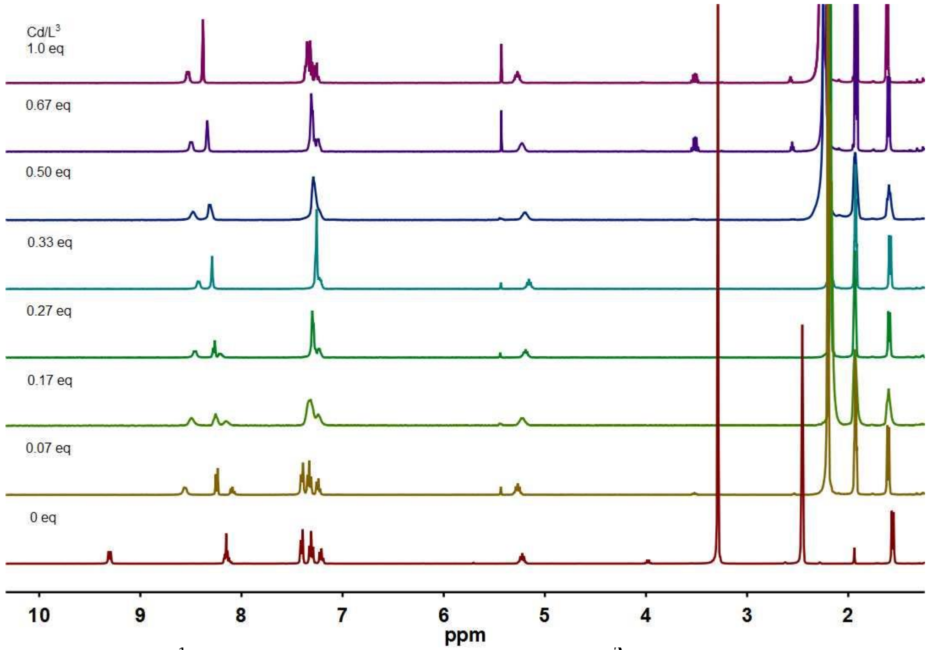

Supplementary Figure 111. ¹H NMR spectra for the titration of $\mathbf{L^2}$ (0.005M) with $Cd(ClO_4)_2$ (CD_3CN, 400 MHz, 298 K).

The titration experiments of L^2 with metal ions (M = Eu^{III}, Ca^{II}, Cd^{II}) indicated the formation of more than one kind of metal-organic assembly species with the ratio $R_{M/L1}$ ranging from 0 to 1.0, suggesting a rather low stability of the monometallic complexes, which hindered its application of in metal ion separation.

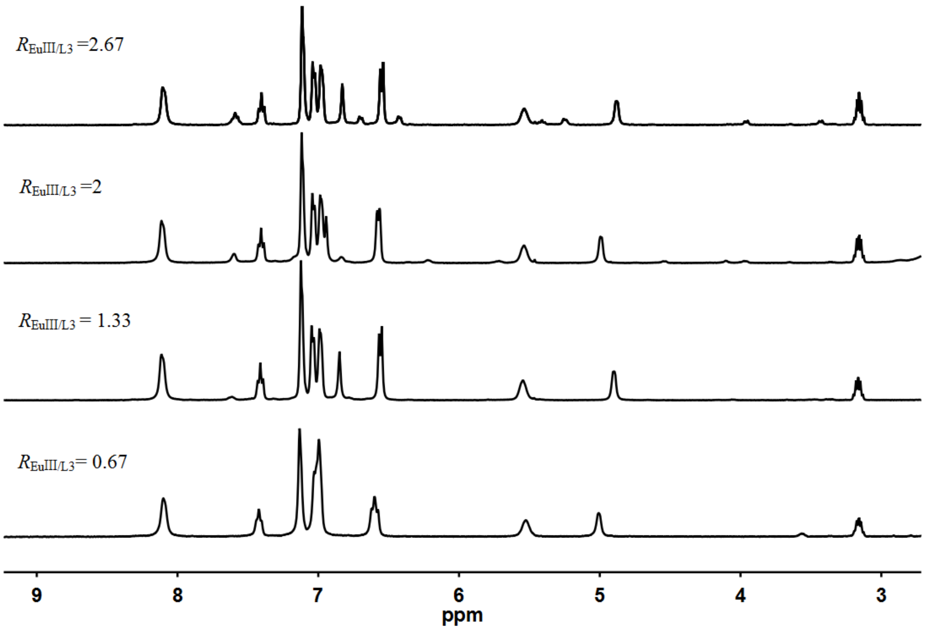

Supplementary Figure 112. ^{1}H NMR spectra for the titration of $\mathbf{L^3}$ (0.005M) with $Eu(ClO_4)_3$ (CD_3CN, 400 MHz, 298 K).

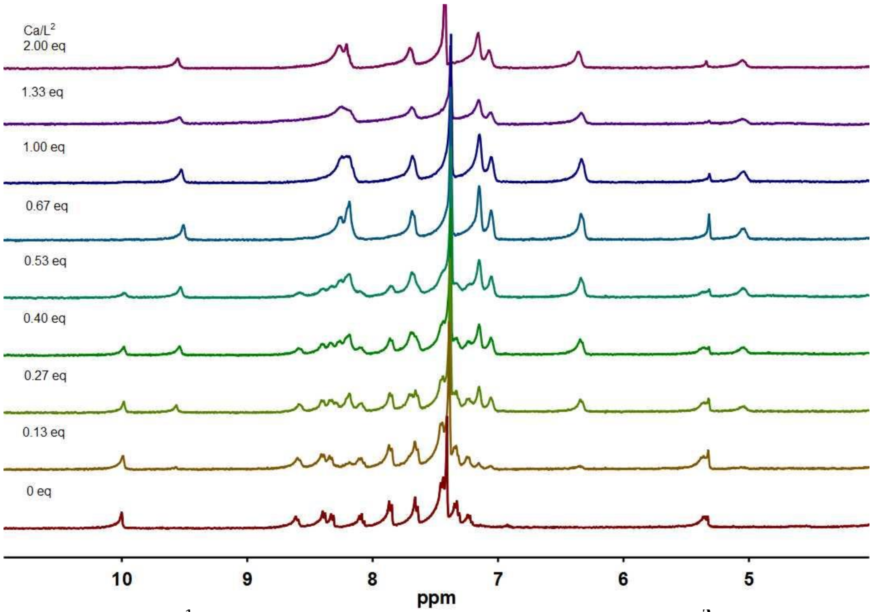

Supplementary Figure 113. ^{1}H NMR spectra for the titration of $\mathbf{L^3}$ (0.0015M) with $Ca(ClO_4)_2$ ($CD_3CN/CDCl_3$, (v:v=1:3) 400 MHz, 298 K).

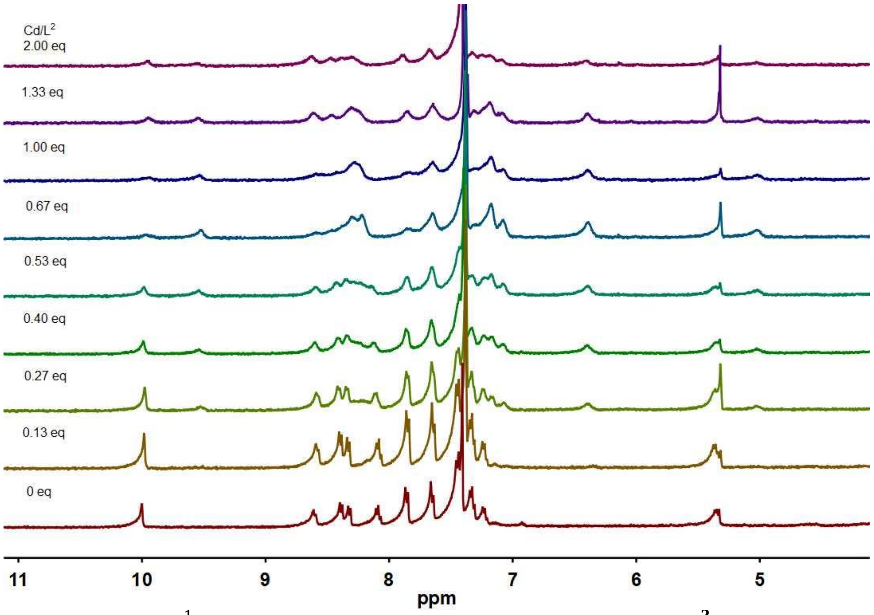

Supplementary Figure 114. ^{1}H NMR spectra for the titration of $\mathbf{L^3}$ (0.0015M) with $Cd(ClO_4)_2$ ($CD_3CN/CDCl_3$, (*v*:*v*=1:3)400 MHz, 298 K).

No successful self-assembly complexes were obtained in the titration experiments of L^3 with $Cd(ClO_4)_2$. And it is worth noting that, both Ca^{II} and Cd^{II} cannot assemble with L^3 in CD_3CN as no clear reaction solution can be obtained and no ^{1}H NMR signals can be acquired. The titration experiments can only be taken in mixture solution of $CD_3CN/CDCl_3$ (*v*:*v*=1:3) at a rather low concentration of the ligands, because of the low solubility of the coordination complexes.

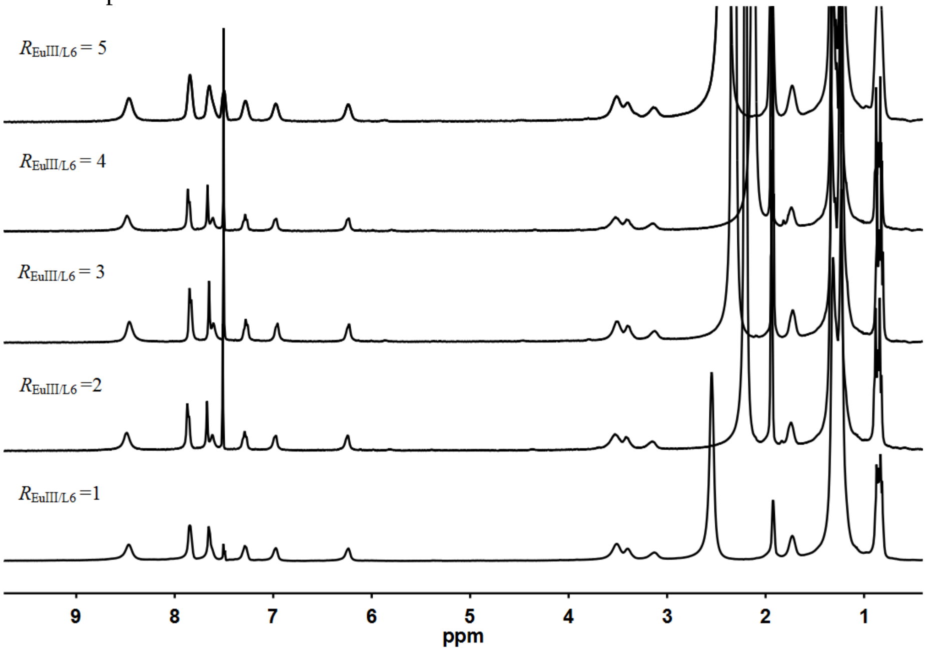

Supplementary Figure 115. ^{1}H NMR spectra for the titration of $\mathbf{L^6}$ (0.002M)with $Eu(CF_3SO_3)_3$ (400 MHz, $CD_3CN/CDCl_3$, 298 K)

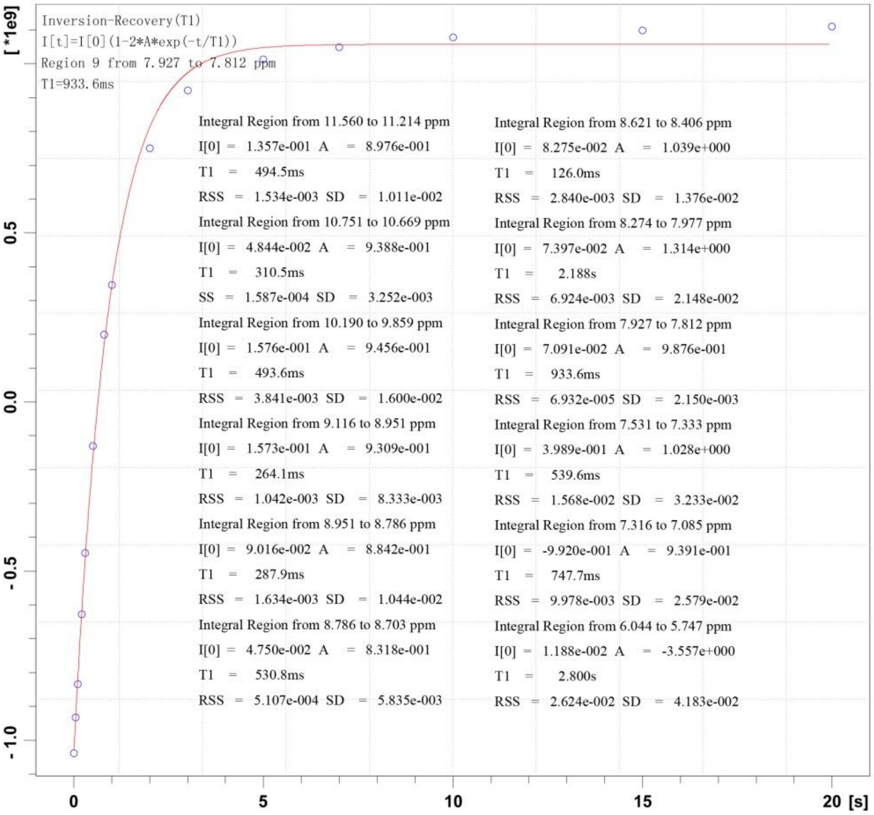

Supplementary Figure 116. Fitting curve of T1 relaxation time of La^{III}/Ce^{III} mixed-metal self-assembled complexes with L^1 with insets showing the T1 values corresponding to different peaks.

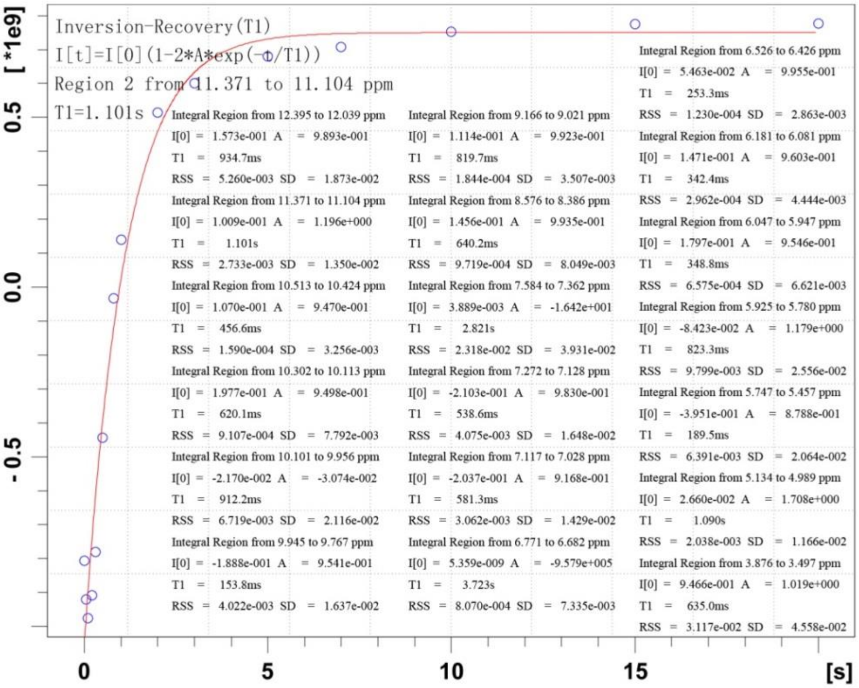

Supplementary Figure 117. Fitting curve of T1 relaxation time of La^{III}/Pr^{III} mixed-metal self-assembled complexes with L^1 with insets showing the T1 values corresponding to different peaks.

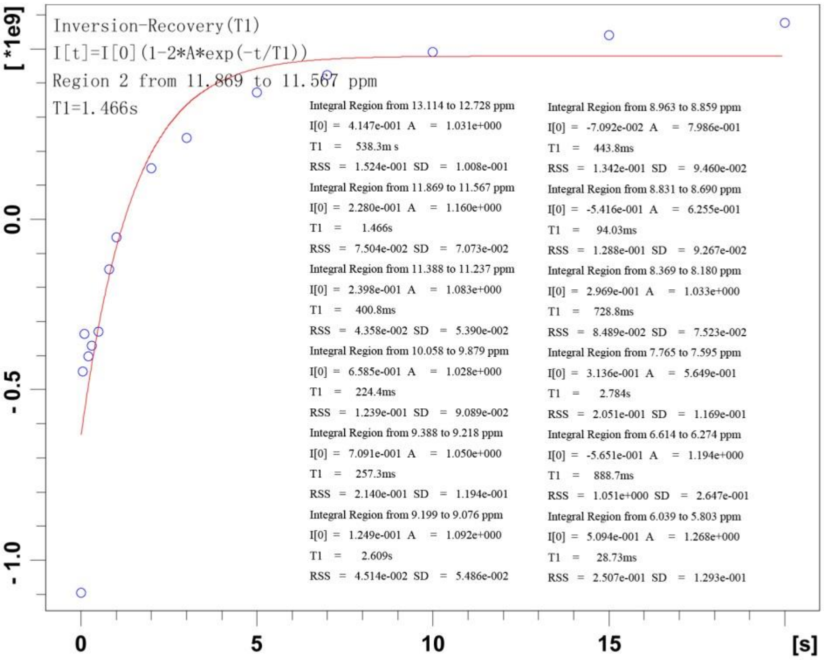

Supplementary Figure 118. Fitting curve of T1 relaxation time of Ce^{III}/Nd^{III} mixed-metal self-assembled complexes with L^1 with insets showing the T1 values corresponding to different peaks.

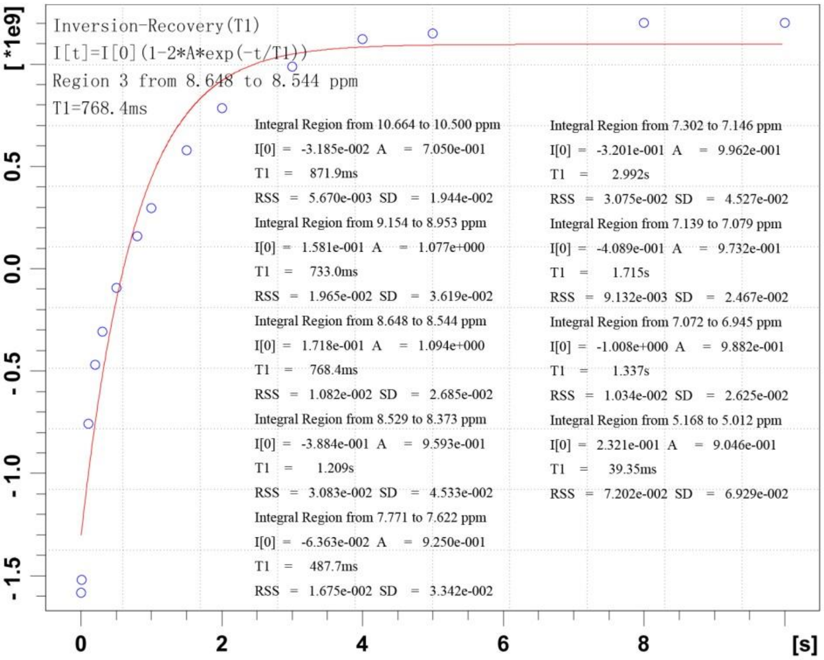

Supplementary Figure 119. Fitting curve of T1 relaxation time of Ce^{III}/Sm^{III} mixed-metal self-assembled complexes with L^1 with insets showing the T1 values corresponding to different peaks.

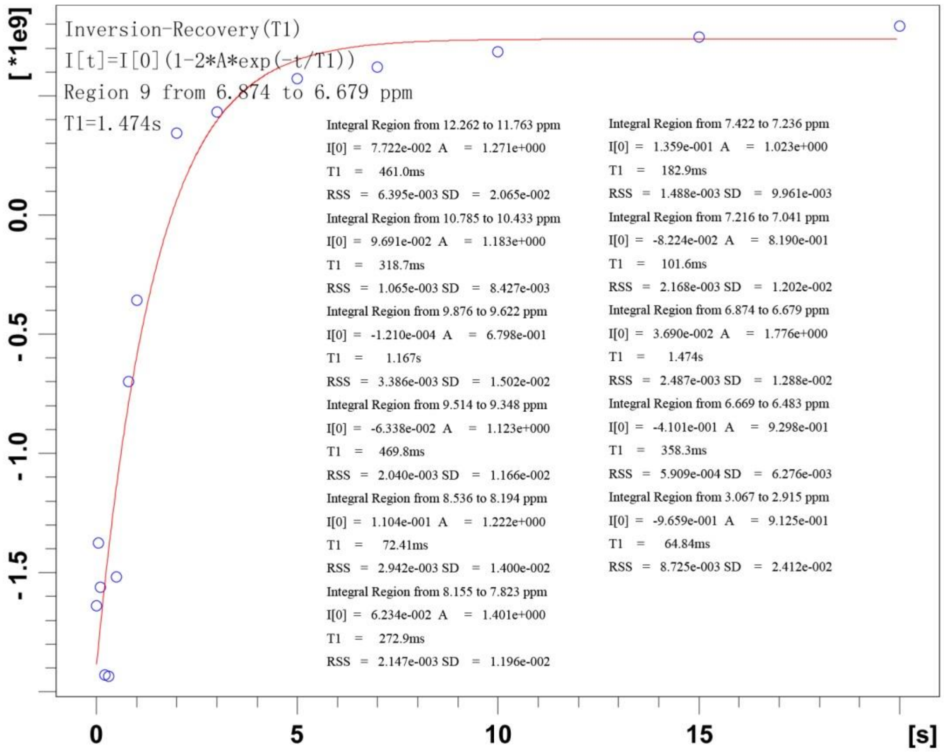

Supplementary Figure 120. Fitting curve of T1 relaxation time of Ce^{III}/Yb^{III} mixed-metal self-assembled complexes with L^1 with insets showing the T1 values corresponding to different peaks.

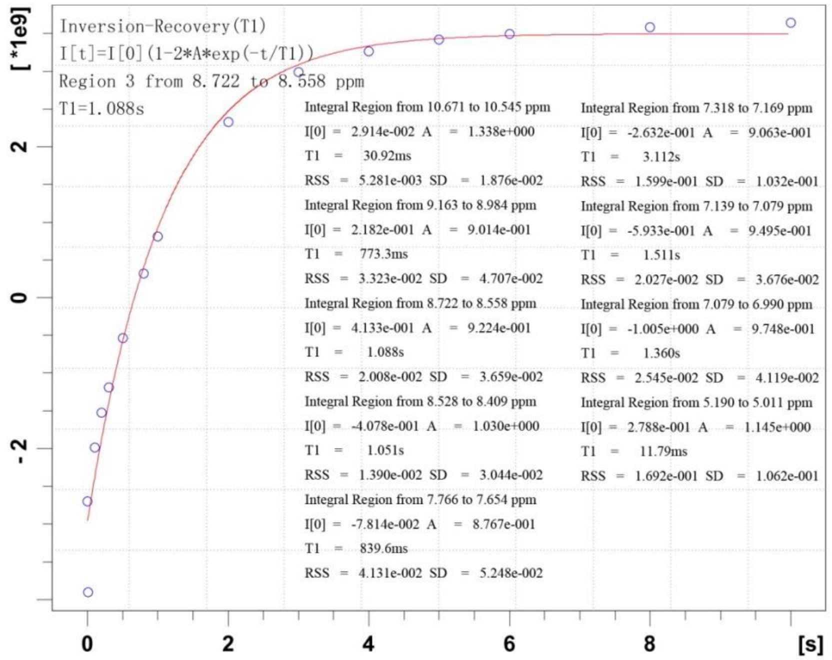

Supplementary Figure 121. Fitting curve of T1 relaxation time of Pr^{III}/Sm^{III} mixed-metal self-assembled complexes with L^1 with insets showing the T1 values corresponding to different peaks.

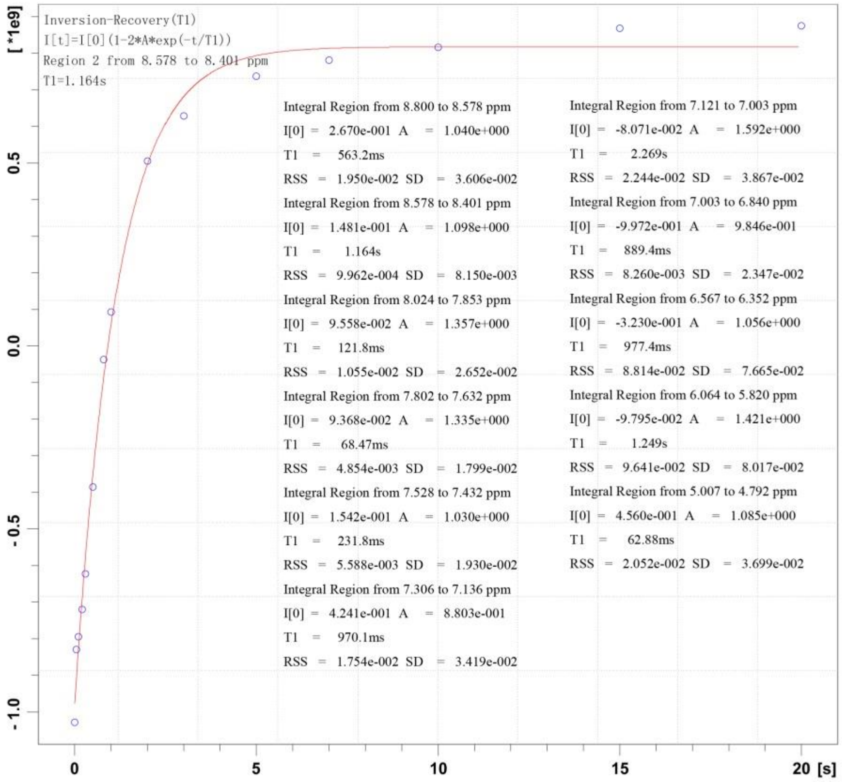

Supplementary Figure 122. Fitting curve of T1 relaxation time of Pr^{III}/Eu^{III} mixed-metal self-assembled complexes with L^1 with insets showing the T1 values corresponding to different peaks.

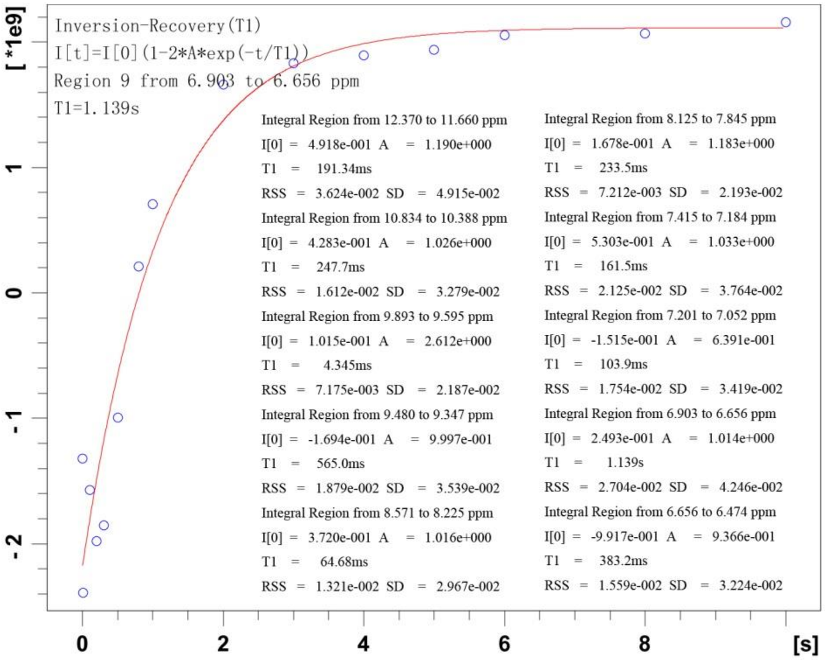

Supplementary Figure 123. Fitting curve of T1 relaxation time of Pr^{III}/Yb^{III} mixed-metal self-assembled complexes with L^1 with insets showing the T1 values corresponding to different peaks.

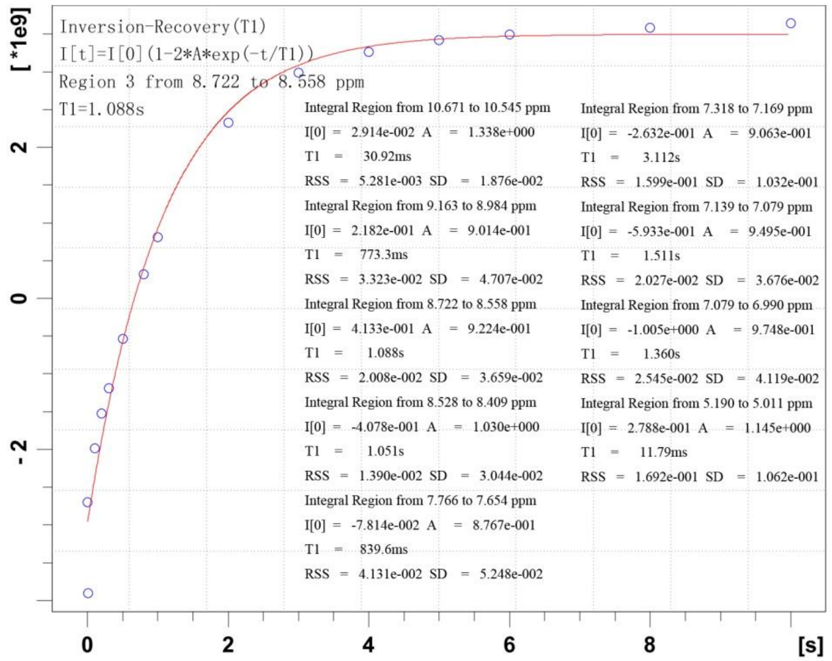

Supplementary Figure 124. Fitting curve of T1 relaxation time of Nd^{III}/Sm^{III} mixed-metal self-assembled complexes with L^1 with insets showing the T1 values corresponding to different peaks.

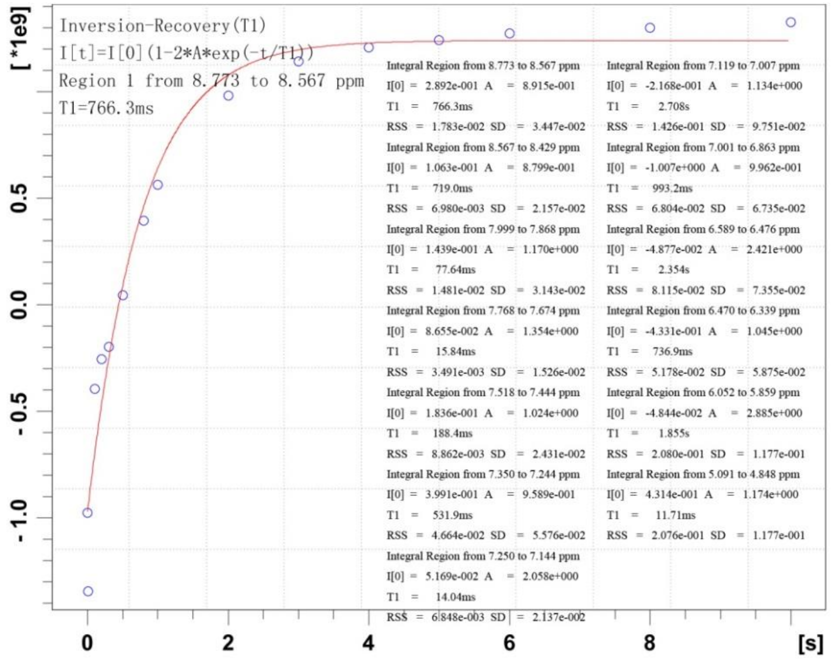

Supplementary Figure 125. Fitting curve of T1 relaxation time of Nd^{III}/Eu^{III} mixed-metal self-assembled complexes with L^1 with insets showing the T1 values corresponding to different peaks.

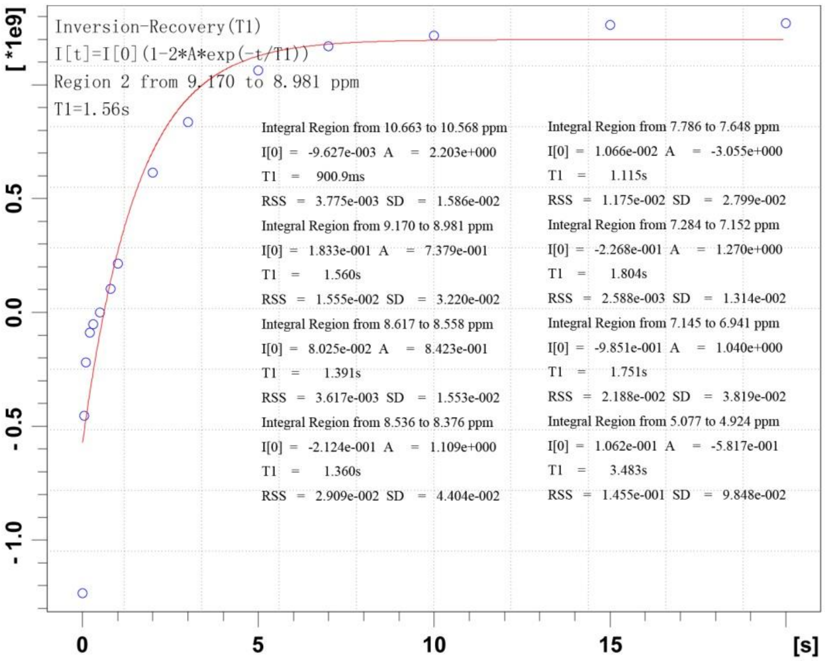

Supplementary Figure 126. Fitting curve of T1 relaxation time of Nd^{III}/Y^{III} mixed-metal self-assembled complexes with L^1 with insets showing the T1 values corresponding to different peaks.

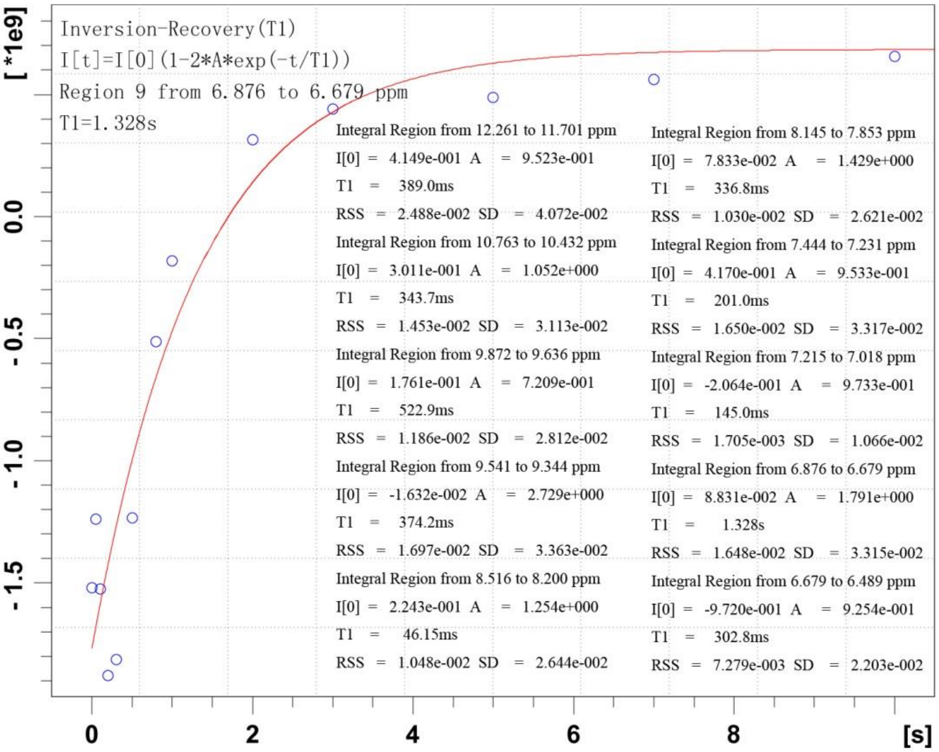

Supplementary Figure 127. Fitting curve of T1 relaxation time of Nd^{III}/Yb^{III} mixed-metal self-assembled complexes with L^1 with insets showing the T1 values corresponding to different peaks.

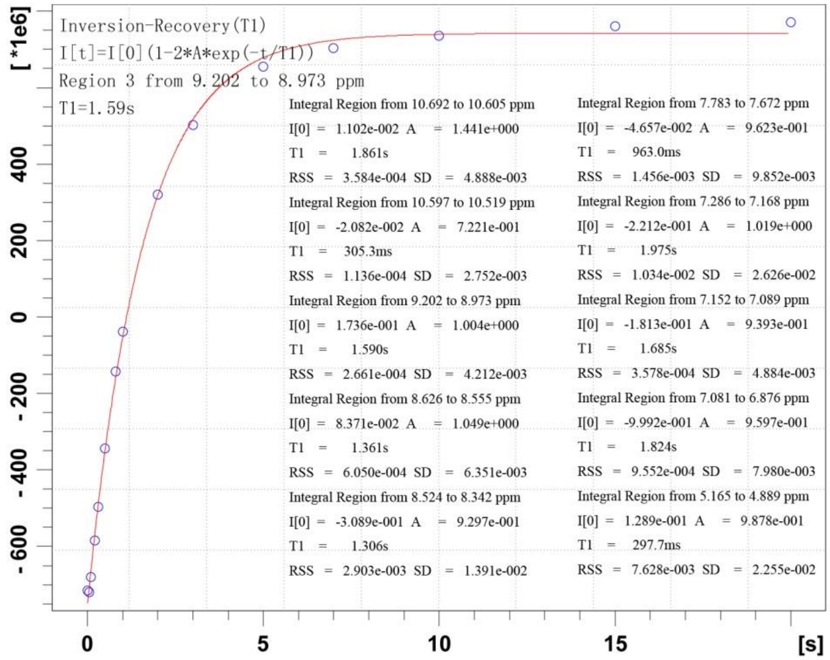

Supplementary Figure 128. Fitting curve of T1 relaxation time of Sm^{III}/Y^{III} mixed-metal self-assembled complexes with L^1 with insets showing the T1 values corresponding to different peaks.

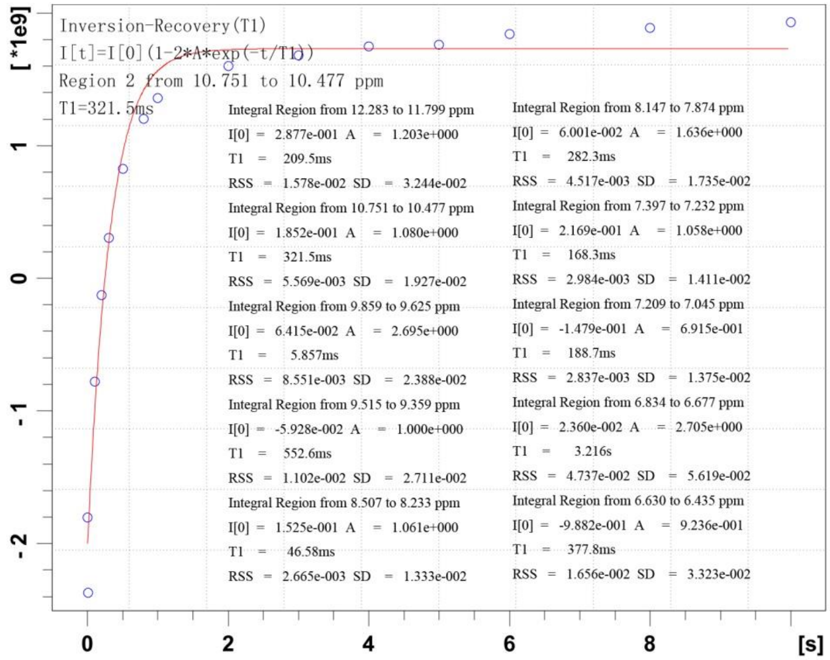

Supplementary Figure 129. Fitting curve of T1 relaxation time of Sm^{III}/Yb^{III} mixed-metal self-assembled complexes with L^1 with insets showing the T1 values corresponding to different peaks.

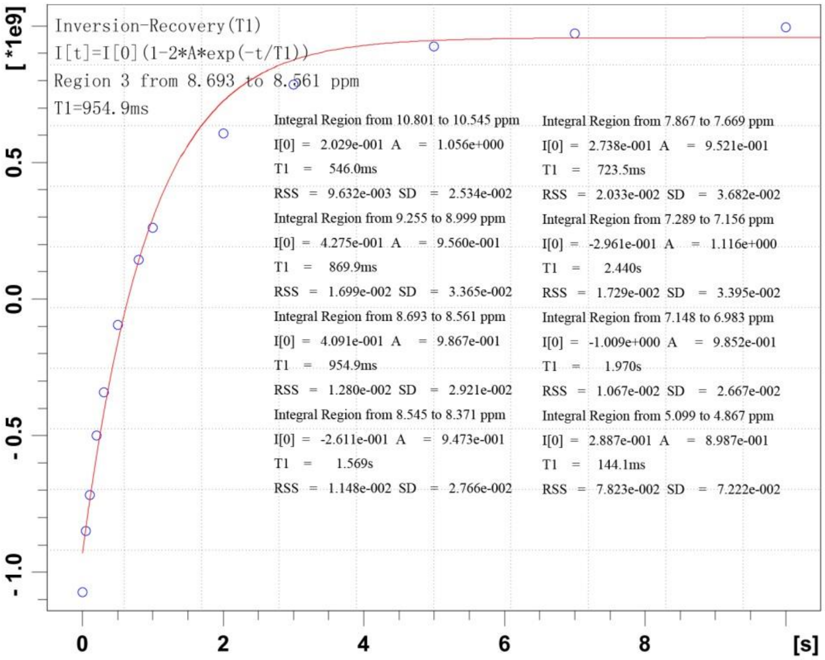

Supplementary Figure 130. Fitting curve of T1 relaxation time of Eu^{III}/Y^{III} mixed-metal self-assembled complexes with L^1 with insets showing the T1 values corresponding to different peaks.

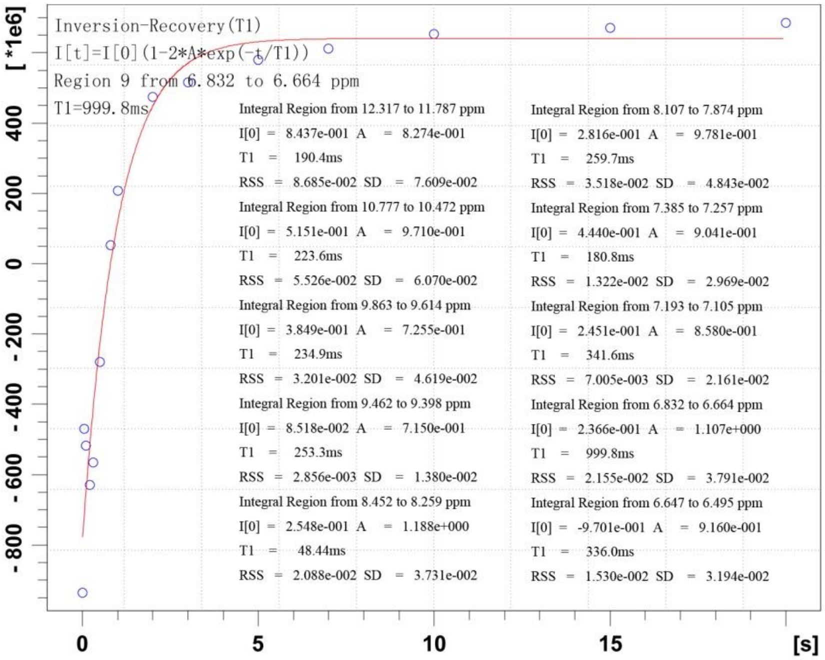

Supplementary Figure 131. Fitting curve of T1 relaxation time of Eu^{III}/Yb^{III} mixed-metal self-assembled complexes with L^1 with insets showing the T1 values corresponding to different peaks.

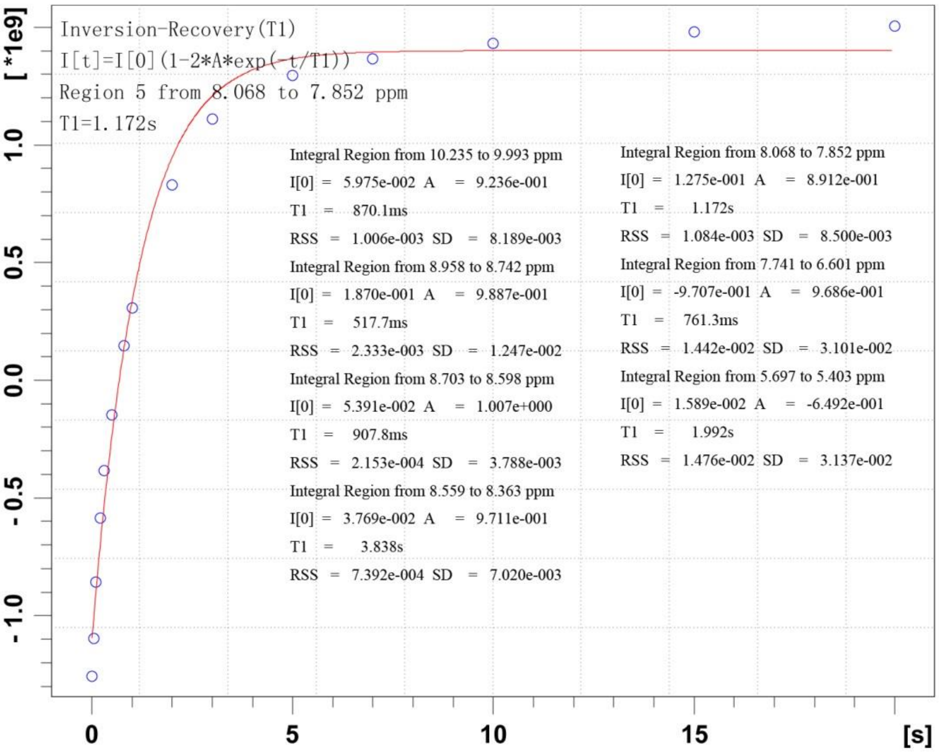

Supplementary Figure 132. Fitting curve of T1 relaxation time of La^{III}/Ce^{III} mixed-metal self-assembled complexes with L^3 with insets showing the T1 values corresponding to different peaks.

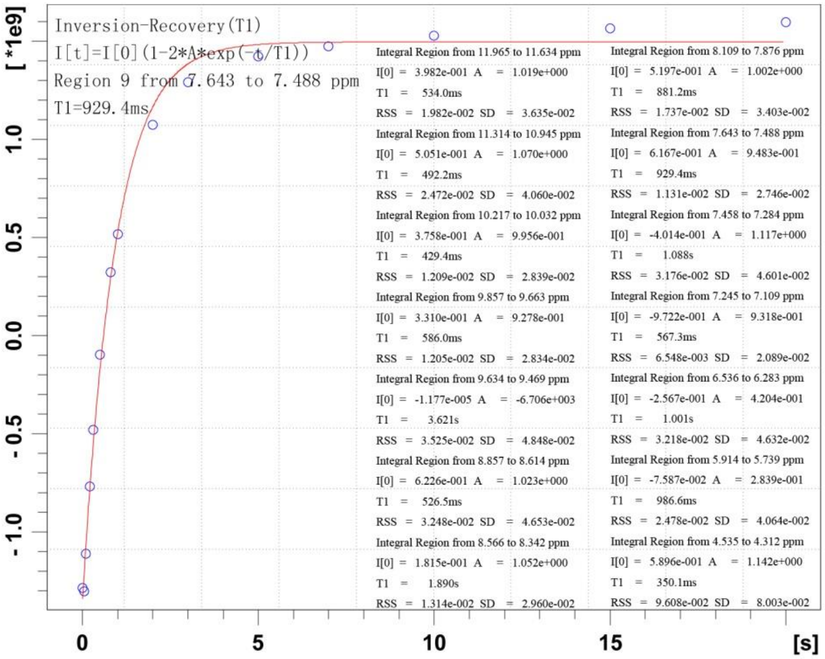

Supplementary Figure 133. Fitting curve of T1 relaxation time of La^{III}/Pr^{III} mixed-metal self-assembled complexes with L^3 with insets showing the T1 values corresponding to different peaks.

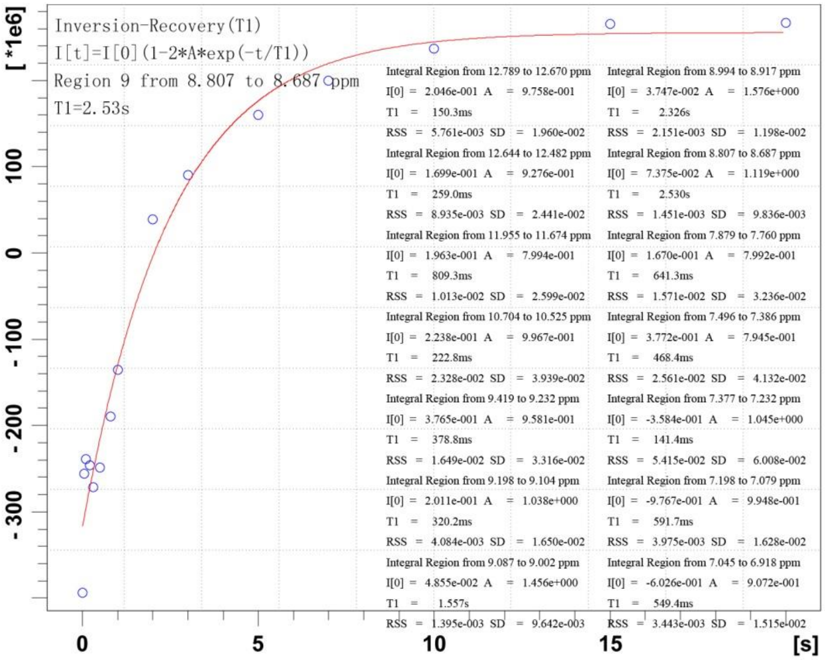

Supplementary Figure 134. Fitting curve of T1 relaxation time of Ce^{III}/Nd^{III} mixed-metal self-assembled complexes with L^3 with insets showing the T1 values corresponding to different peaks.

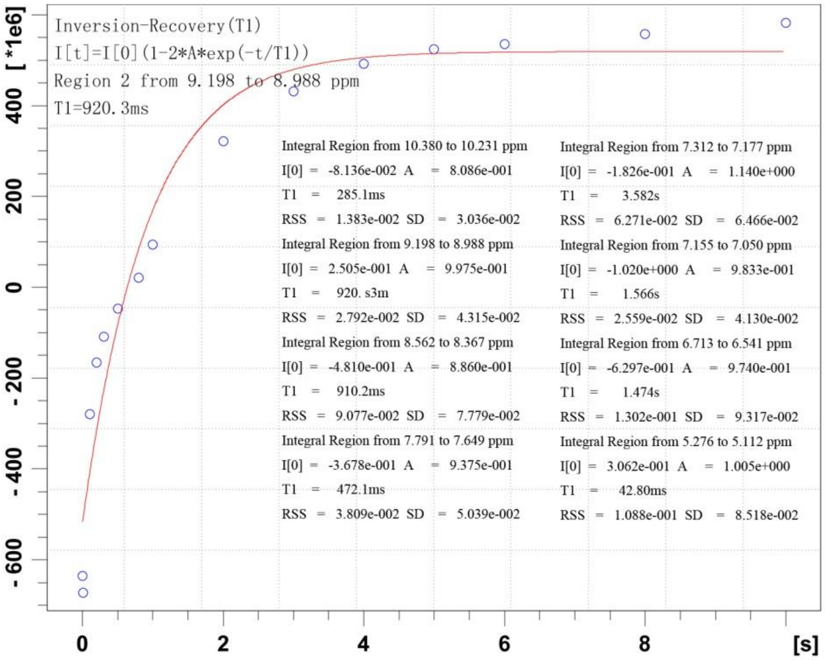

Supplementary Figure 135. Fitting curve of T1 relaxation time of Ce^{III}/Sm^{III} mixed-metal self-assembled complexes with L^3 with insets showing the T1 values corresponding to different peaks.

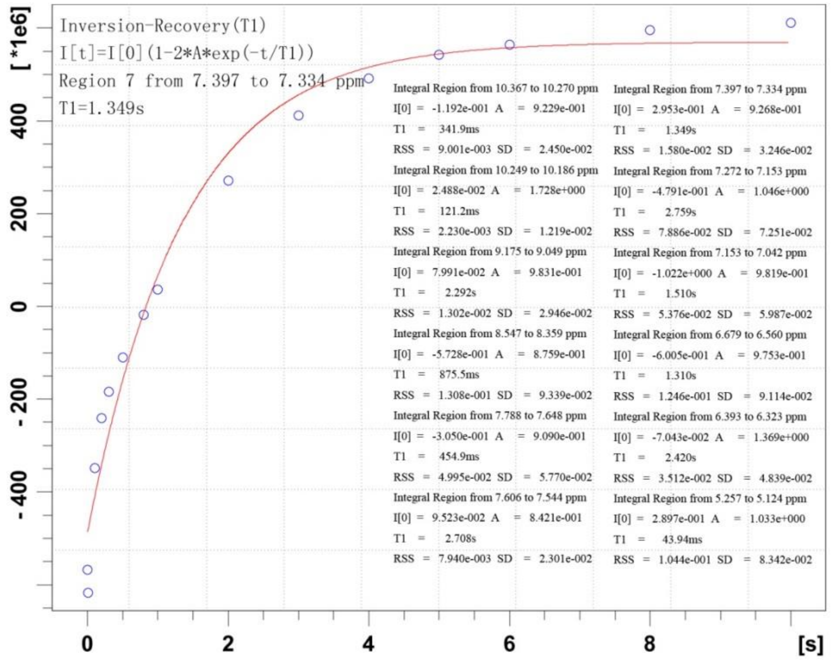

Supplementary Figure 136. Fitting curve of T1 relaxation time of Pr^{III}/Sm^{III} mixed-metal self-assembled complexes with L^3 with insets showing the T1 values corresponding to different peaks.

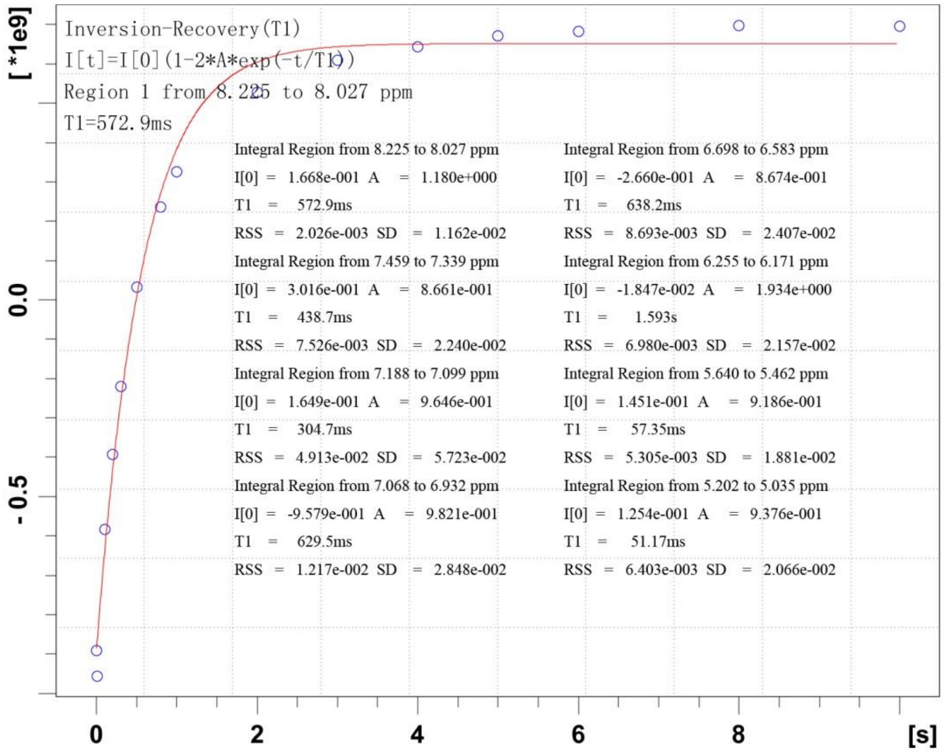

Supplementary Figure 137. Fitting curve of T1 relaxation time of Pr^{III}/Eu^{III} mixed-metal self-assembled complexes with L^3 with insets showing the T1 values corresponding to different peaks.

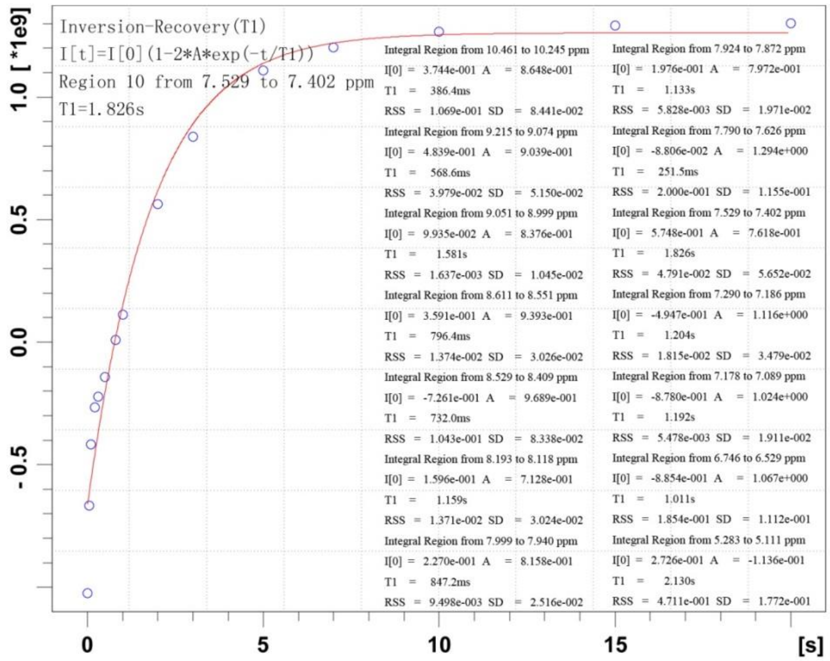

Supplementary Figure 138. Fitting curve of T1 relaxation time of Nd^{III}/Sm^{III} mixed-metal self-assembled complexes with L^3 with insets showing the T1 values corresponding to different peaks.

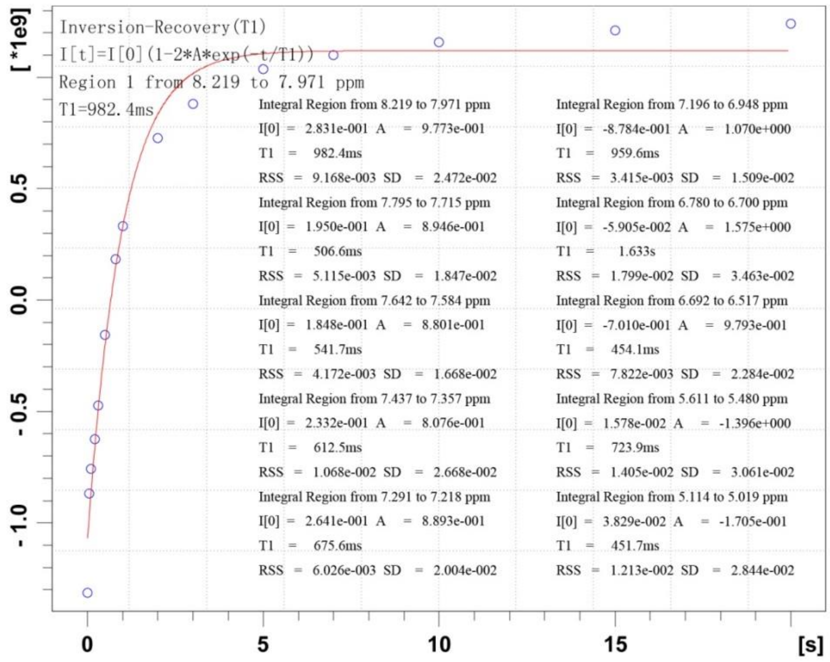

Supplementary Figure 139. Fitting curve of T1 relaxation time of Nd^{III}/Eu^{III} mixed-metal self-assembled complexes with L^3 with insets showing the T1 values corresponding to different peaks.

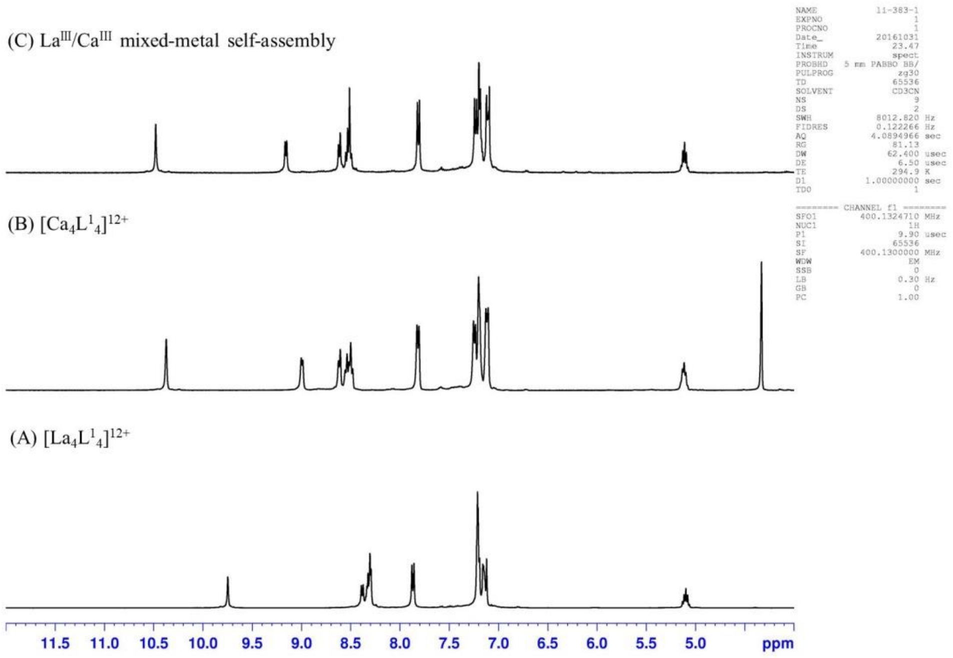

Supplementary Figure 140. ^{1}H NMR spectra (400 MHz, CD_3CN, 298K) for $[Ca_4L^1_4]^{8+}$ (A), $[La_4L^1_4]^{12+}$ (B) and La^{III}/Ca^{II} mixed-metal self-assembled complexes (ClO_4^- salt) (C).
Absolute metal ion self-recognition assembly (at 40 ℃ for 1h) of L^1 (0.01 M) with La^{III} was obtained according to the ^{1}H NMR characterization and no spectra change in (C) was observed even after 2 weeks.

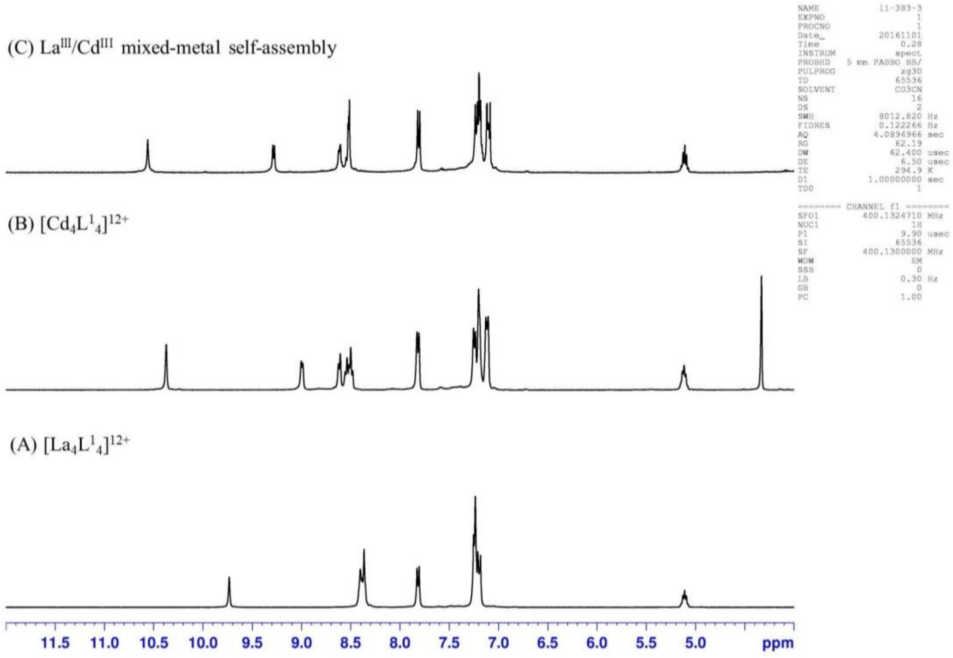

Supplementary Figure 141. ^{1}H NMR spectra (400 MHz, CD_3CN, 298K) for $[Cd_4L^1_4]^{8+}$ (A), $[La_4L^1_4]^{12+}$ (B) and La^{III}/Cd^{II} mixed-metal self-assembled complexes with L^1 (ClO_4^- salt) (C).
Absolute metal ion self-recognition assembly (at 40 ℃ for 1h) of L^1 (0.01 M) with La^{III} was obtained according to the ^{1}H NMR characterization and no spectra change in (C) was observed even after 2 weeks.

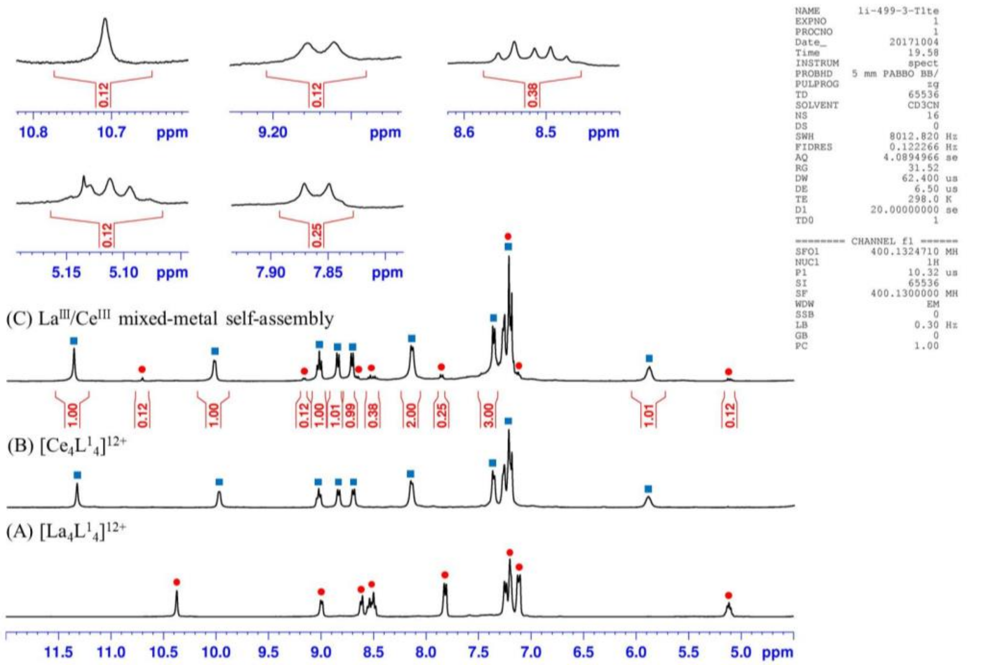

Supplementary Figure 142. ^{1}H NMR spectra (400 MHz, CD_3CN, 298K) for $[La_4L^1_4]^{8+}$ (A), $[Ce_4L^1_4]^{12+}$ (B) and La^{III}/Ce^{III} mixed-metal self-assembled complexes with L^1 (ClO_4^- salt) (C) with spectra zoomed in over some of the peak ranges selected for the integrals.

Non-absolute metal ion self-recognition assembly (at 40 ℃ for 1h) of L^1 (0.01 M) with La^{III}/Ce^{III} mixtures (0.01 M of each), with 89.3 percent Ce^{III} and 10.7 percent La^{III} in the assembled complexes, was observed according to the ^{1}H NMR characterization and no spectra change in (C) was observed even after 2 weeks.

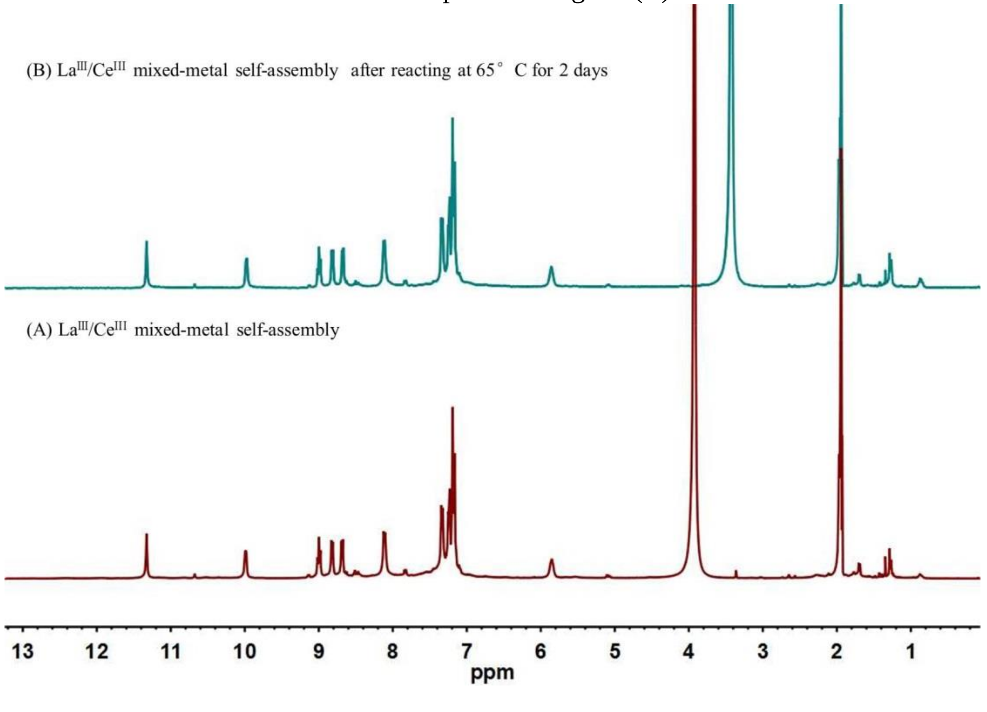

Supplementary Figure 143. ^{1}H NMR spectra (400 MHz, CD_3CN, 298K) of La^{III}/Ce^{III} mixed-metal self-assembled complexes with L^1 (ClO_4^- salt)

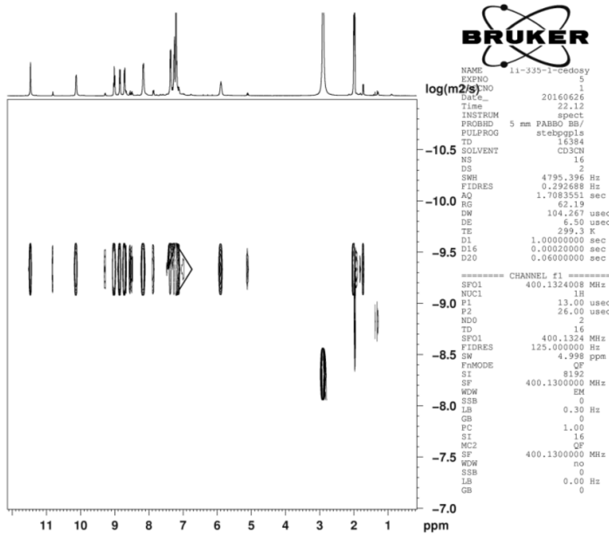

Diffusion Constant = 4.121E-10m²/S   d = 3.0 nm

Supplementary Figure 144. **^{1}**H DOSY spectra (CD_3CN, 400 MHz, 298 K) of La^{III}/Ce^{III} mixed-metal self-assembled complexes with L^1 (ClO_4^- salt).

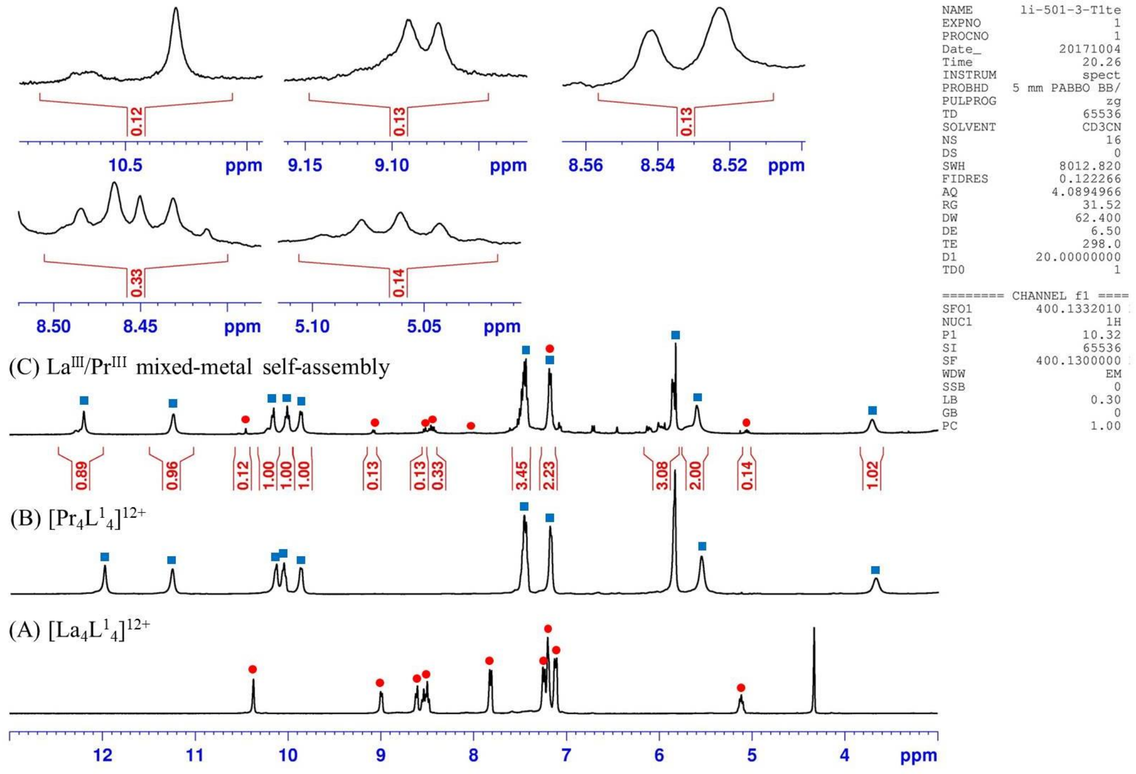

Supplementary Figure 145. ^{1}H NMR spectra (400 MHz, CD_3CN, 298K) for $[La_4L^1_4]^{8+}$ (A), $[Pr_4L^1_4]^{12+}$ (B) and La^{III}/Pr^{III} mixed-metal self-assembled complexes with L^1 (ClO_4^- salt) (C) with spectra zoomed in over some of the peak ranges selected for the integrals.

Non-absolute metal ion self-recognition assembly (at 40 ℃ for 1h) of L^1 (0.01 M) with La^{III}/Pr^{III} mixtures (0.01 M of each), with 88.50 percent Pr^{III} and 11.50 percent La^{III} in the assembled complexes, was observed according to the ^{1}H NMR characterization and no spectra change in (C) was observed even after 2 weeks.

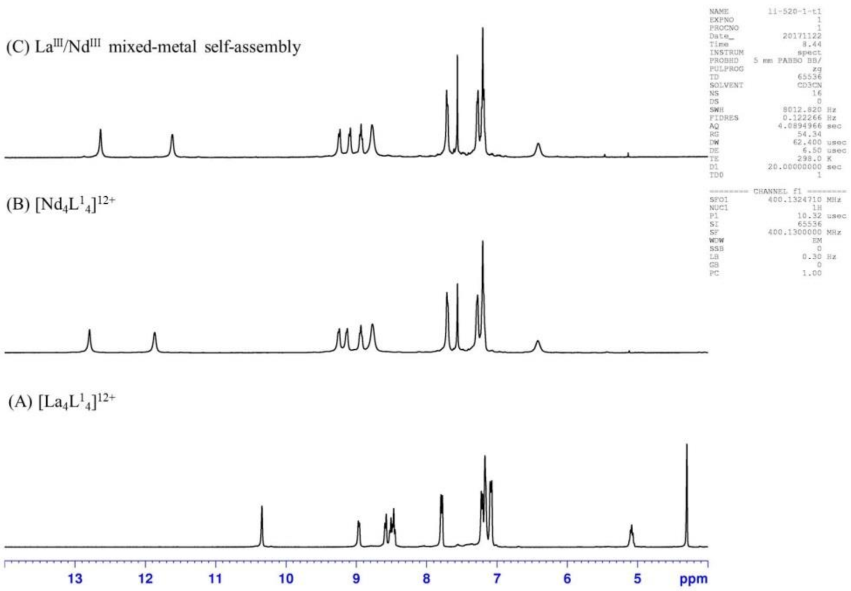

Supplementary Figure 146. ^{1}H NMR spectra (400 MHz, CD_3CN, 298K) for $[La_4L^1{}_4]^{8+}$ (A), $[Nd_4L^1{}_4]^{12+}$ (B) and La^{III}/Nd^{III} mixed-metal self-assembled complexes with L^1 (ClO_4^- salt) (C).
No spectra change in (C) was observed even after 2 weeks.

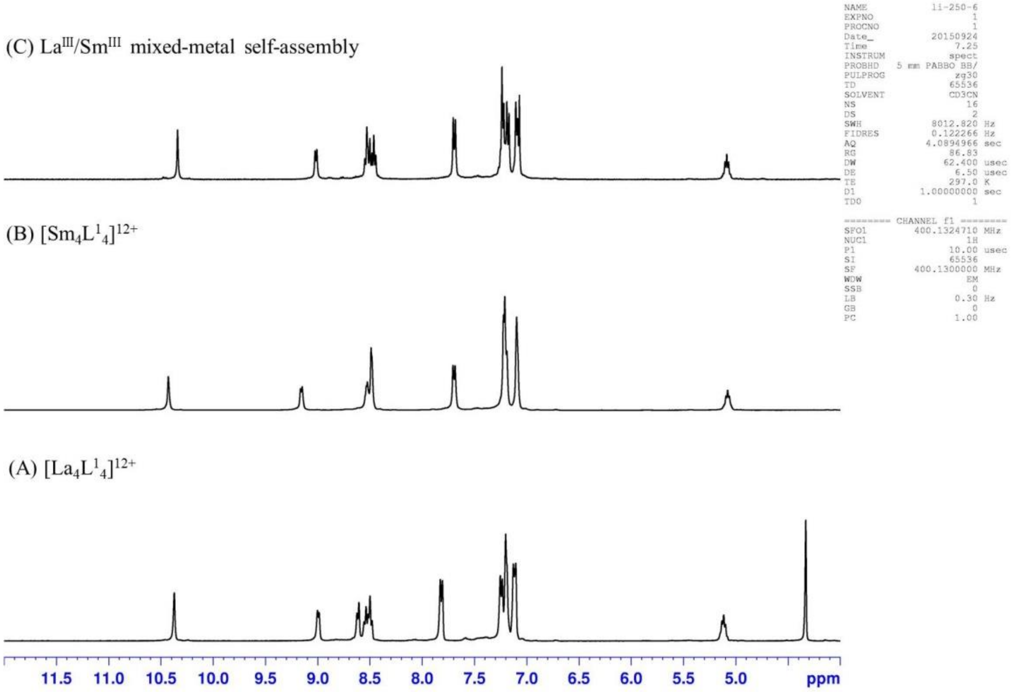

Supplementary Figure 147. ^{1}H NMR spectra (400 MHz, CD_3CN, 298K) for $[La_4L^1{}_4]^{12+}$ (A), $[Sm_4L^1{}_4]^{12+}$ (B) and La^{III}/Sm^{III} mixed-metal self-assembled complexes with L^1 (ClO_4^- salt) (C).
Complete metal ion self-recognition assembly (at 40 ℃ for 1h) of L^1 (0.01 M) with Sm^{III} was obtained according to the ^{1}H NMR characterization and no spectra change in (C) was observed even after 2 weeks.

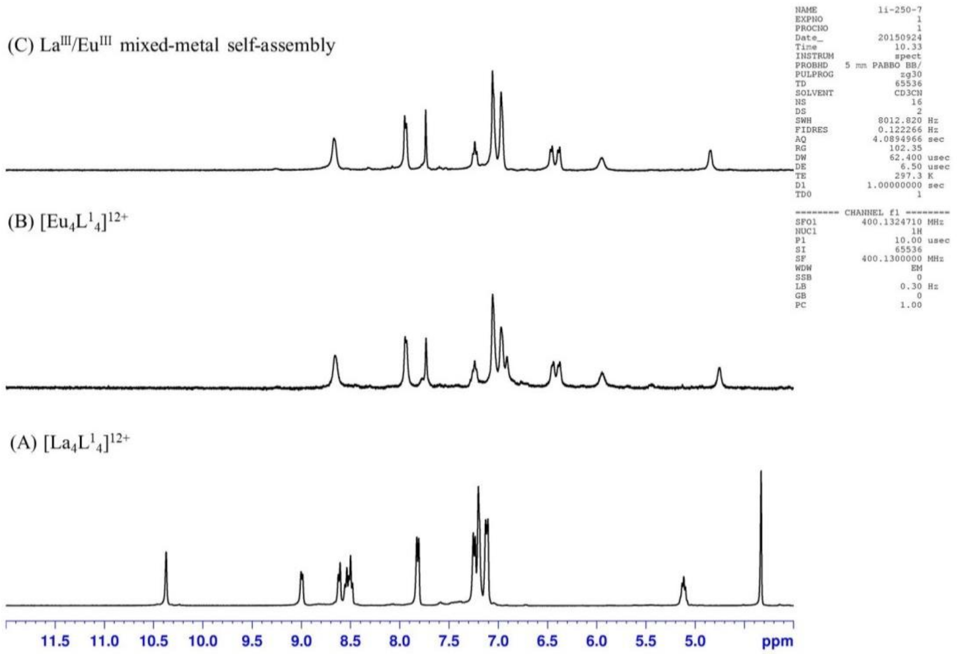

Supplementary Figure 148. ^{1}H NMR spectra (400 MHz, CD_3CN, 298K) for $[La_4L^1_4]^{12+}$ (A), $[Eu_4L^1_4]^{12+}$ (B) and La^{III}/Eu^{III} mixed-metal self-assembled complexes with L^1 (ClO_4^- salt) (C).
Complete metal ion self-recognition assembly (at 40 ℃ for 1h) of L^1 (0.01 M) with Eu^{III} was obtained according to the ^{1}H NMR characterization and no spectra change in (C) was observed even after 2 weeks.

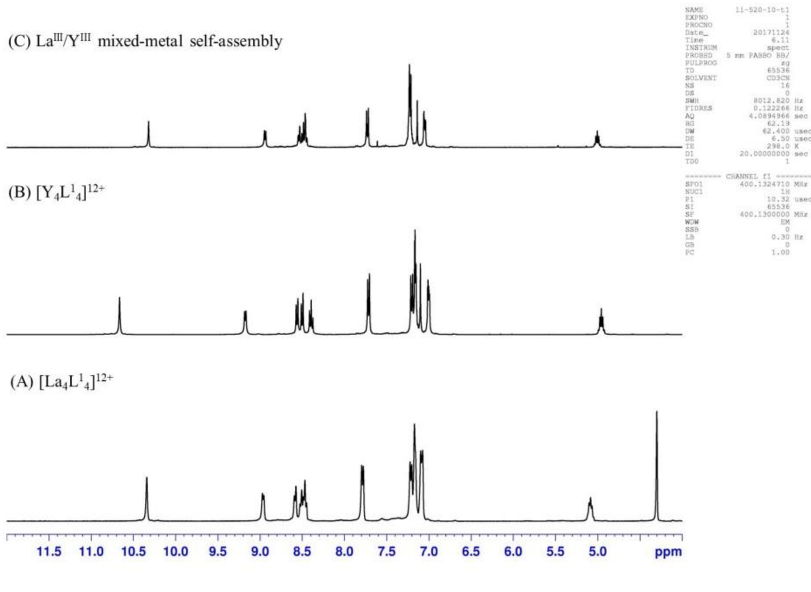

Supplementary Figure 149. ^{1}H NMR spectra (400 MHz, CD_3CN, 298K) for $[La_4L^1_4]^{12+}$ (A), $[Y_4L^1_4]^{12+}$ (B) and La^{III}/Y^{III} mixed-metal self-assembled complexes with L^1 (ClO_4^- salt) (C).
Complete metal ion self-recognition assembly (at 40 ℃ for 1h) of L^1 (0.01 M) with Y^{III} was obtained according to the ^{1}H NMR characterization and no spectra change in (C) was observed even after 2 weeks.

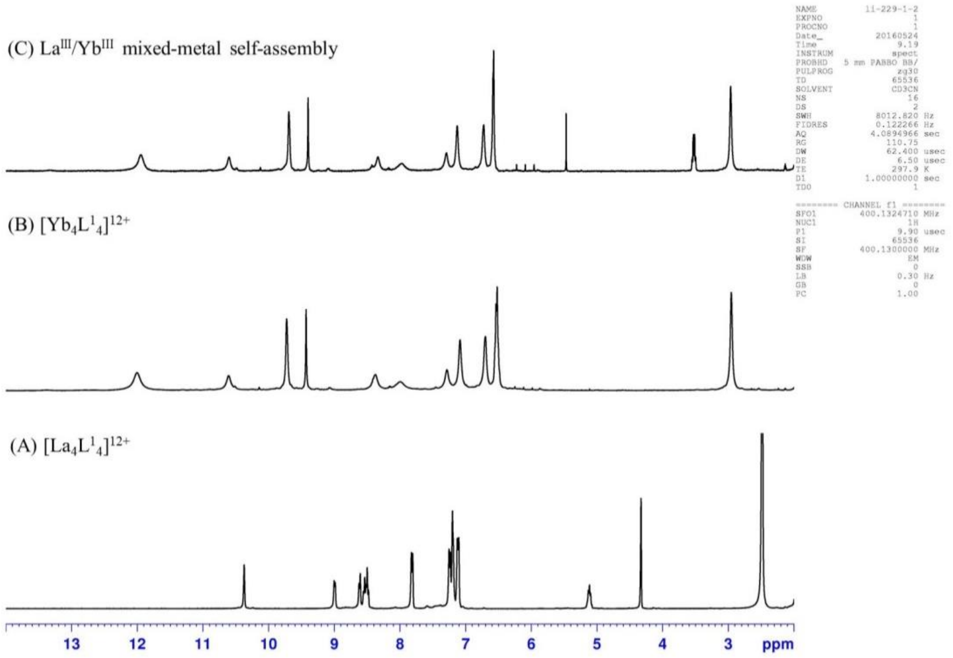

Supplementary Figure 150. ^{1}H NMR spectra (400 MHz, CD_3CN, 298K) for $[La_4L^1_4]^{12+}$ (A), $[Yb_4L^1_4]^{12+}$ (B) and La^{III}/Yb^{III} mixed-metal self-assembled complexes with L^1 ($CF_3SO_3^-$ salt) (C).
Complete metal ion self-recognition assembly (at 40 ℃ for 1h) of L^1 (0.01 M) with Yb^{III} was obtained according to the ^{1}H NMR characterization and no spectra change in (C) was observed even after 2 weeks.

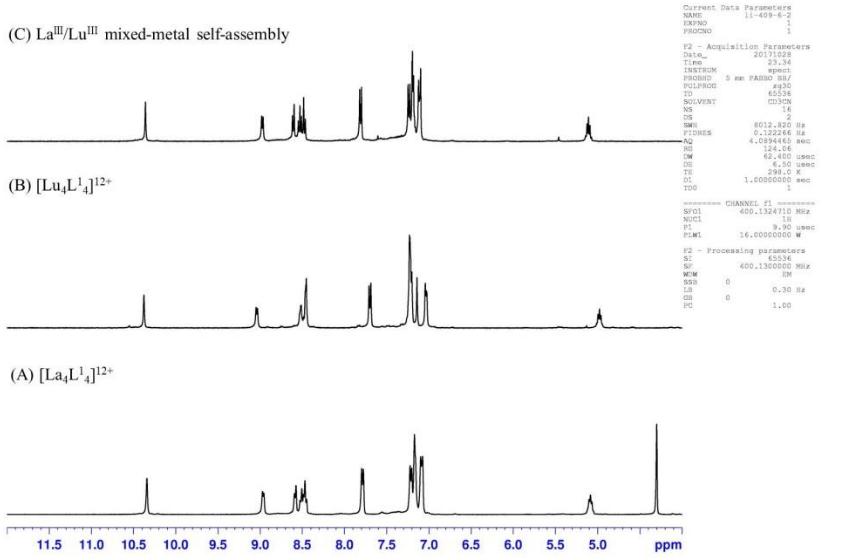

Supplementary Figure 151. ^{1}H NMR spectra (400 MHz, CD_3CN, 298K) for $[La_4L^1_4]^{12+}$ (A), $[Lu_4L^1_4]^{12+}$ (B) and La^{III}/Lu^{III} mixed-metal self-assembled complexes with L^1 (ClO_4^- salt) (C).
Complete metal ion self-recognition assembly (at 40 ℃ for 1h) of L^1 (0.01 M) with Lu^{III} was obtained according to the ^{1}H NMR characterization and no spectra change in (C) was observed even after 2 weeks.

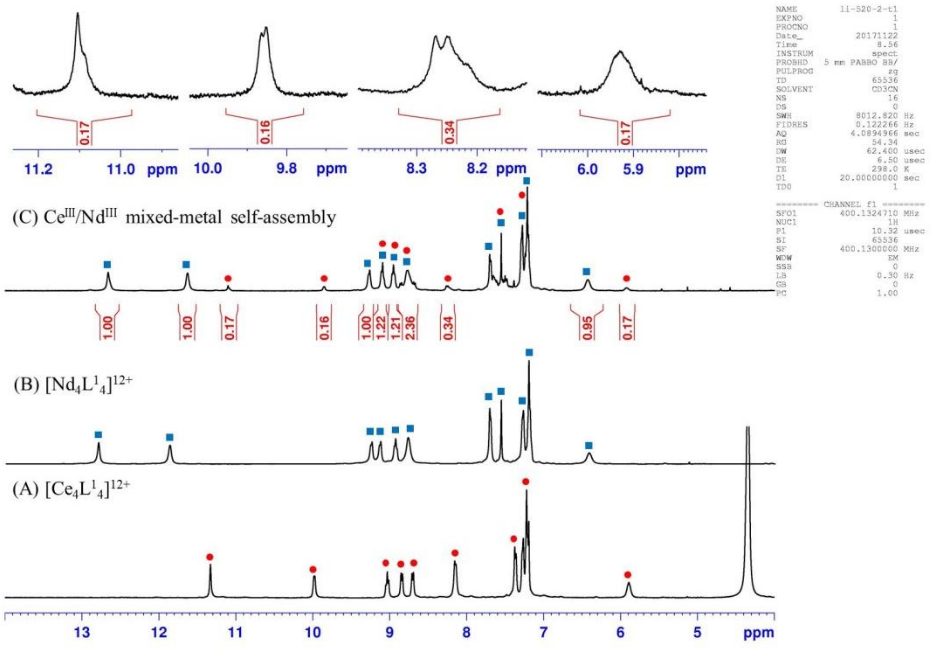

Supplementary Figure 152. ^{1}H NMR spectra (400 MHz, CD_3CN, 298K) for $[Ce_4L^1{}_4]^{12+}$ (A), $[Nd_4L^1{}_4]^{12+}$ (B) and Ce^{III}/Nd^{III} mixed-metal self-assembled complexes with L^1 (ClO_4^- salt) (C) with spectra zoomed in over some of the peak ranges selected for the integrals.

Non-absolute metal ion self-recognition assembly (at 40 ℃ for 1h) of L^1 (0.01 M) with Ce^{III}/Nd^{III} mixtures (0.01 M of each), with 85.47 percent Nd^{III} and 14.53 percent Ce^{III} in the assembled complexes, was observed according to the ^{1}H NMR characterization and no spectra change in (C) was observed even after 2 weeks.

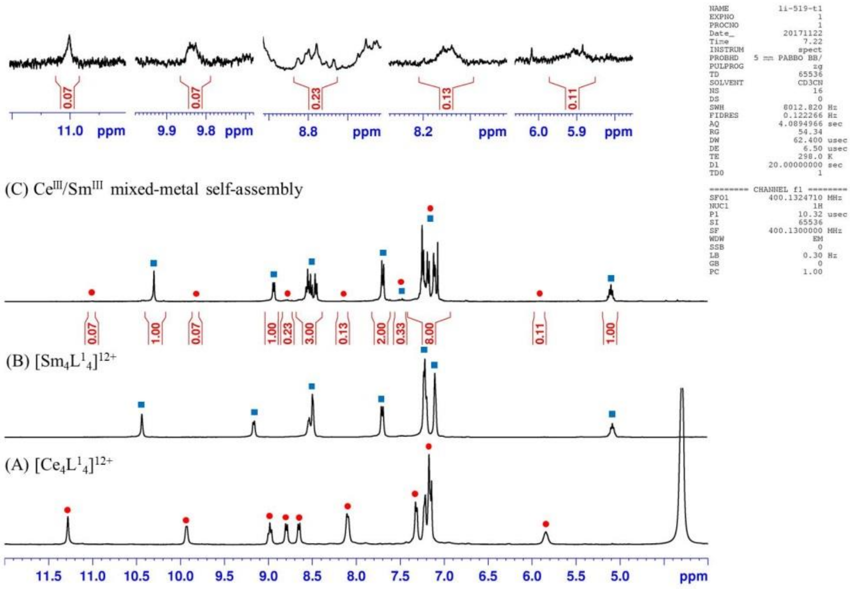

Supplementary Figure 153. ^{1}H NMR spectra (400 MHz, CD_3CN, 298K) for $[Ce_4L^1{}_4]^{12+}$ (A), $[Sm_4L^1{}_4]^{12+}$ (B) and Ce^{III}/Sm^{III} mixed-metal self-assembled complexes with L^1 (ClO_4^- salt) (C) with spectra zoomed in over some of the peak ranges selected for the integrals.

Non-absolute metal ion self-recognition assembly (at 40 ℃ for 1h) of L^1 (0.01 M) with Ce^{III}/Sm^{III} mixtures (0.01 M of each), with 93.46 percent Sm^{III} and 6.54 percent Ce^{III} in the assembled complexes, was observed according to the ^{1}H NMR characterization and no spectra change in (C) was observed even after 2 weeks.

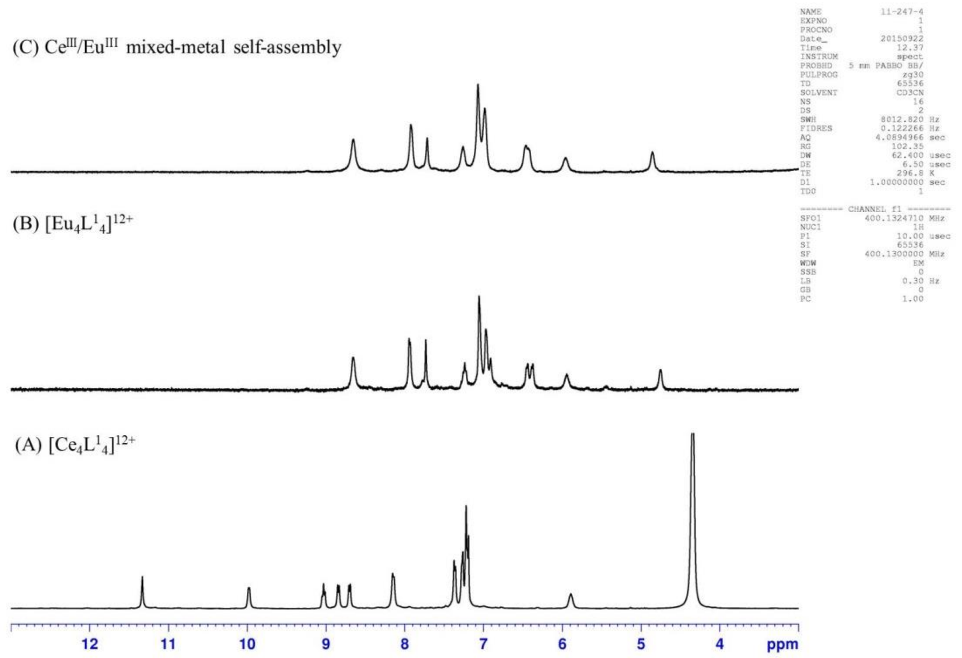

Supplementary Figure 154. ^{1}H NMR spectra (400 MHz, CD_3CN, 298K) for $[Ce_4L^1_4]^{12+}$ (A), $[Eu_4L^1_4]^{12+}$ (B) and Ce^{III}/Eu^{III} mixed-metal self-assembled complexes with L^1 (ClO_4^- salt) (C).
Complete metal ion self-recognition assembly (at 40 ℃ for 1h) of L^1 (0.01 M) with Eu^{III} was obtained according to the ^{1}H NMR characterization and no spectra change in (C) was observed even after 2 weeks.

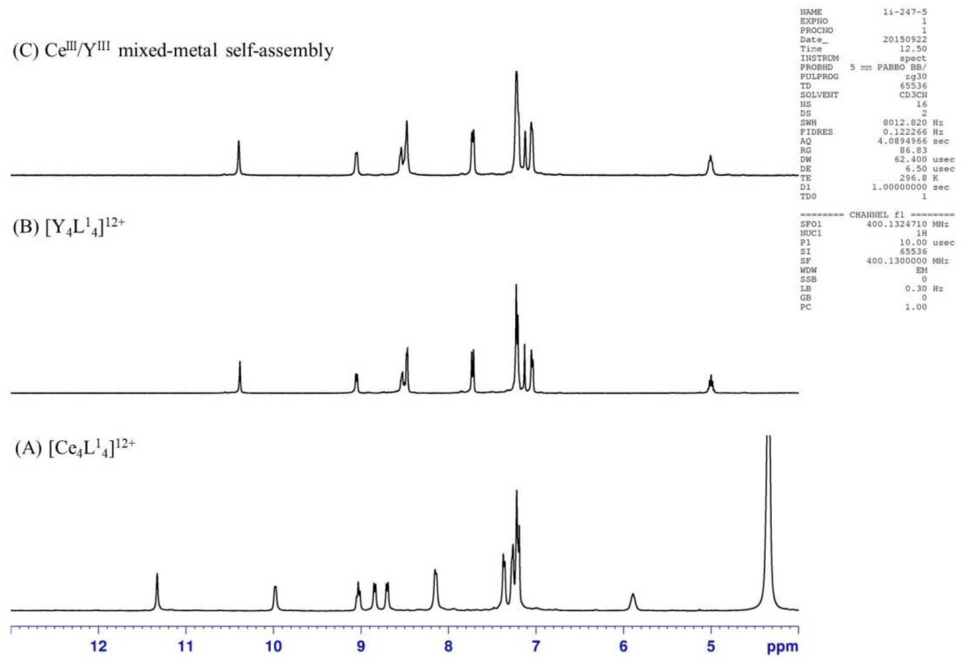

Supplementary Figure 155. ^{1}H NMR spectra (400 MHz, CD_3CN, 298K) for $[Ce_4L^1_4]^{12+}$ (A), $[Y_4L^1_4]^{12+}$ (B) and Ce^{III}/Y^{III} mixed-metal self-assembled complexes with L^1 (ClO_4^- salt) (C).
Complete metal ion self-recognition assembly (at 40 ℃ for 1h) of L^1 (0.01 M) with Y^{III} was obtained according to the ^{1}H NMR characterization and no spectra change in (C) was observed even after 2 weeks.

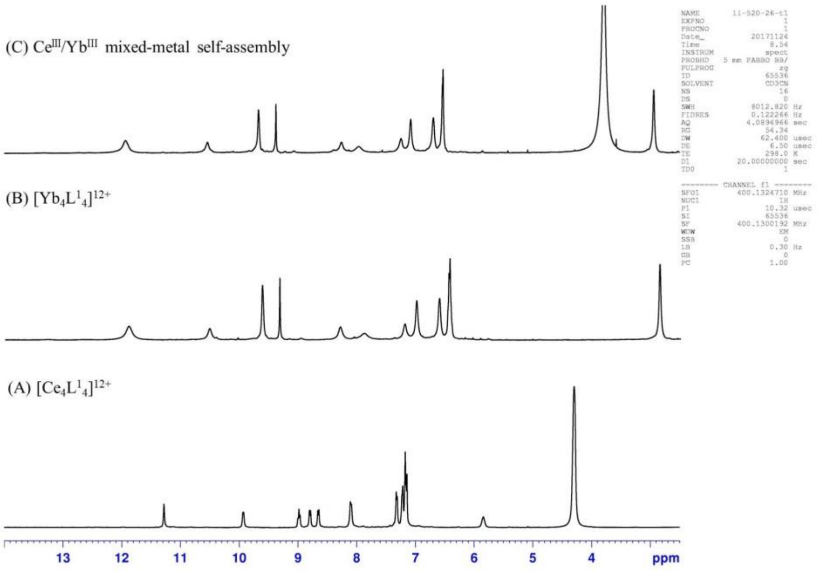

Supplementary Figure 156. ^{1}H NMR spectra (400 MHz, CD_3CN, 298K) for $[Ce_4L^1_4]^{12+}$ (A), $[Yb_4L^1_4]^{12+}$ (B) and Ce^{III}/Yb^{III} mixed-metal self-assembled complexes with L^1 ($CF_3SO_3^-$ salt) (C).

ESI-TOF-MS measurement indicates non-absolute metal ion self-recognition assembly of L^1 with Ce^{III}/Yb^{III} mixtures. However, the severe line-broadening of the ^{1}H NMR spectrum caused by paramagnetism of Yb^{III} hinders precise quantitation of different lanthanide ions in the assembled complexes through integration of the NMR spectra.

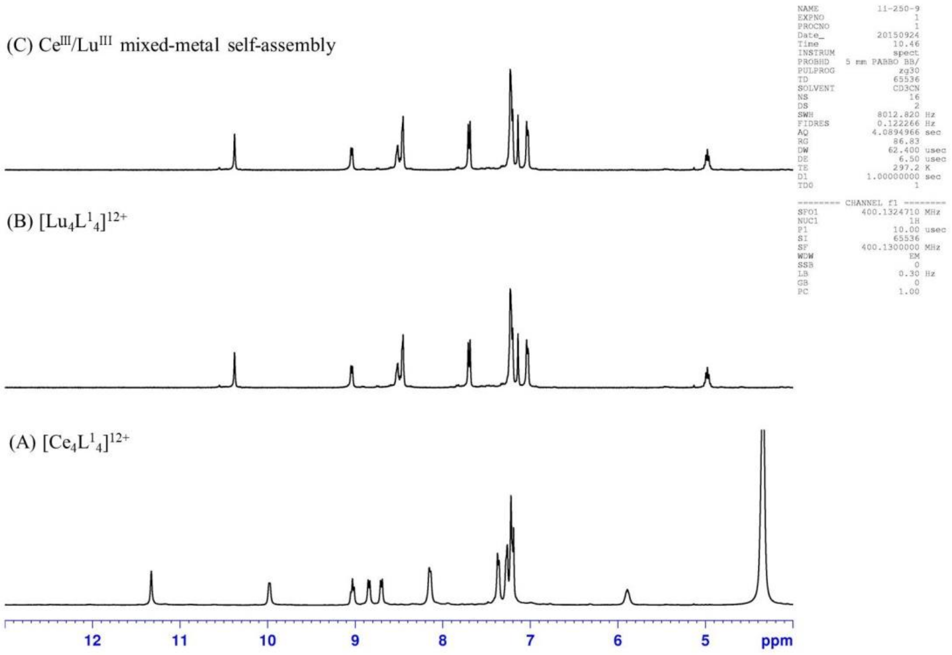

Supplementary Figure 157. ^{1}H NMR spectra (400 MHz, CD_3CN, 298K) for $[Ce_4L^1_4]^{12+}$ (A), $[Lu_4L^1_4]^{12+}$ (B) and Ce^{III}/Lu^{III} mixed-metal self-assembled complexes with L^1 ($CF_3SO_3^-$ salt) (C).

Complete metal ion self-recognition assembly (at 40 ℃ for 1h) of L^1 (0.01 M) with Lu^{III} was obtained according to the ^{1}H NMR characterization and no spectra change in (C) was observed even after 2 weeks.

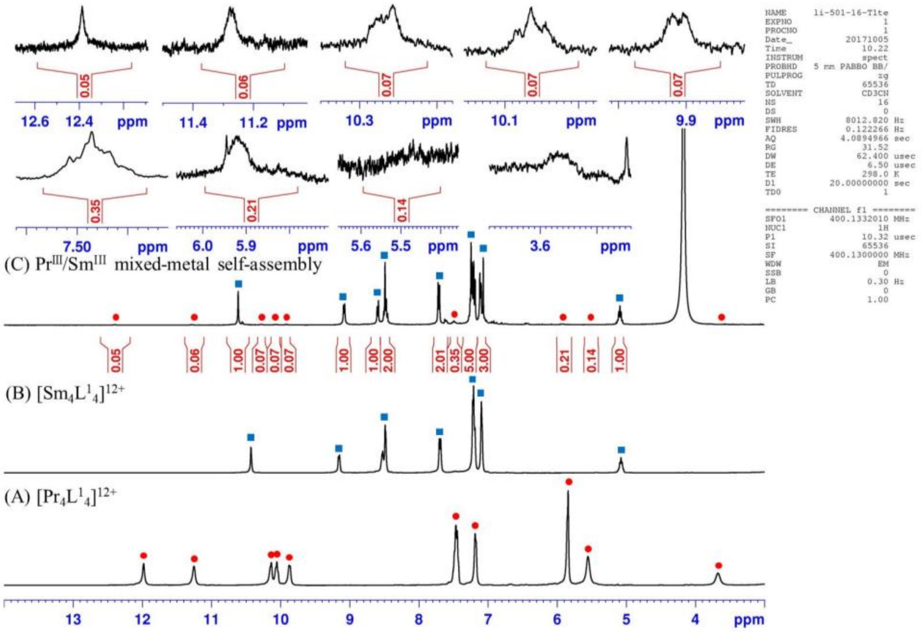

Supplementary Figure 158. ^{1}H NMR spectra (400 MHz, CD_3CN, 298K) for $[Pr_4L^1_4]^{12+}$ (A), $[Sm_4L^1_4]^{12+}$ (B) and Pr^{III}/Sm^{III} mixed-metal self-assembled complexes with L^1 ($CF_3SO_3^-$ salt) (C) with spectra zoomed in over some of the peak ranges selected for the integrals.

Non-absolute metal ion self-recognition assembly (at 40 ℃ for 1h) of L^1 (0.01 M) with Pr^{III}/Sm^{III} mixtures (0.01 M of each), with 93.46 percent Sm^{III} and 6.54 percent Pr^{III} in the assembled complexes, was observed according to the ^{1}H NMR characterization and no spectra change in (C) was observed even after 2 weeks.

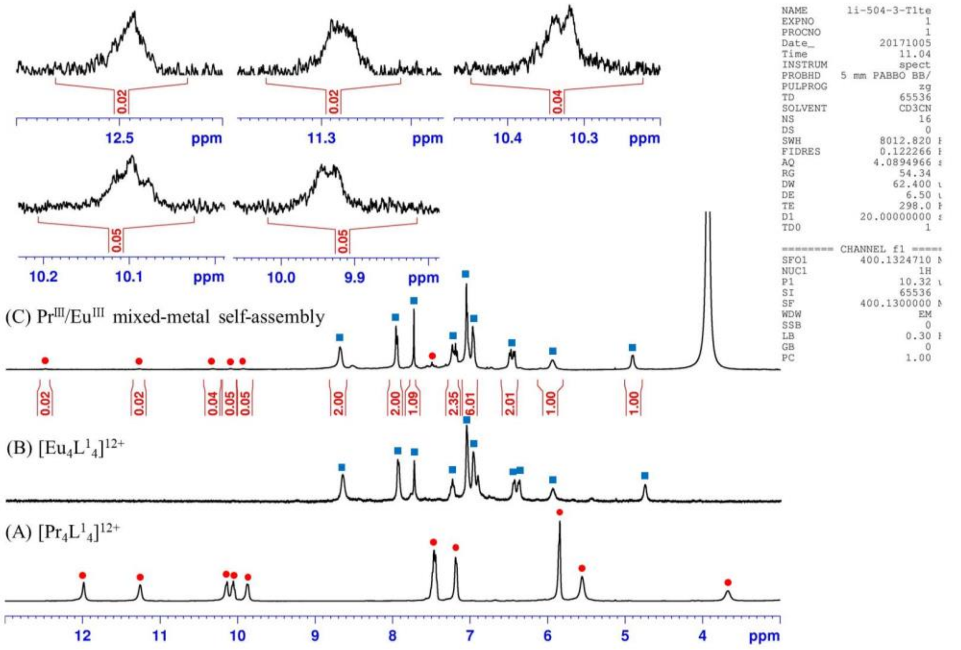

Supplementary Figure 159. ^{1}H NMR spectra (400 MHz, CD_3CN, 298K) for $[Pr_4L^1_4]^{12+}$ (A), $[Eu_4L^1_4]^{12+}$ (B) and Pr^{III}/Eu^{III} mixed-metal self-assembled complexes with L^1 ($CF_3SO_3^-$ salt) (C) with spectra zoomed in over some of the peak ranges selected for the integrals.

Non-absolute metal ion self-recognition assembly (at 40 ℃ for 1h) of L^1 (0.01 M) with Pr^{III}/Eu^{III} mixtures (0.01 M of each), with 95.24 percent Eu^{III} and 4.76 percent Pr^{III} in the assembled complexes, was observed according to the ^{1}H NMR characterization and no spectra change in (C) was observed even after 2 weeks.

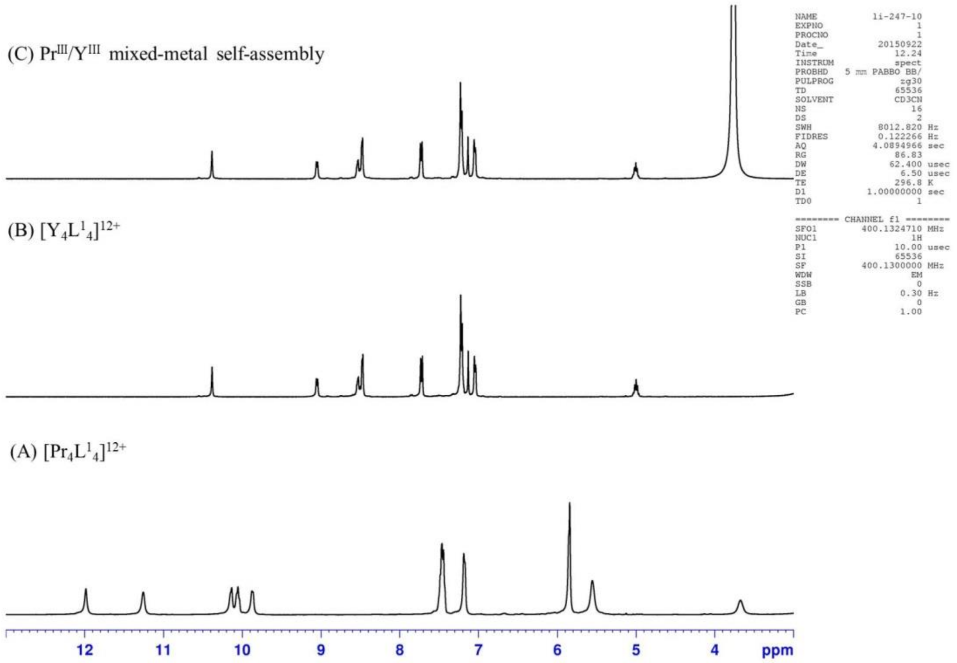

Supplementary Figure 160. ^{1}H NMR spectra (400 MHz, CD_3CN, 298K) for $[Pr_4L^1_4]^{12+}$ (A), $[Y_4L^1_4]^{12+}$ (B) and Pr^{III}/Y^{III} mixed-metal self-assembled complexes with L^1 (ClO_4^- salt) (C).

Complete metal ion self-recognition assembly (at 40 ℃ for 1h) of L^1 (0.01 M) with Y^{III} was obtained according to the ^{1}H NMR characterization and no spectra change in (C) was observed even after 2 weeks.

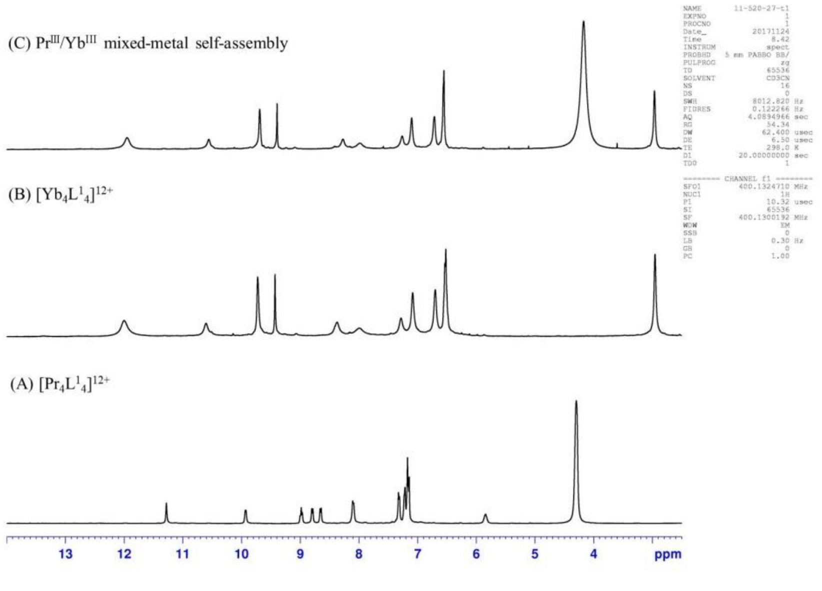

Supplementary Figure 161. ^{1}H NMR spectra (400 MHz, CD_3CN, 298K) for $[Pr_4L^1_4]^{12+}$ (A), $[Yb_4L^1_4]^{12+}$ (B) and Pr^{III}/Yb^{III} mixed-metal self-assembled complexes with L^1 ($CF_3SO_3^-$ salt) (C).

ESI-TOF-MS measurement indicates non-absolute metal ion self-recognition assembly of L^1 with Ce^{III}/Yb^{III} mixtures. However, the severe line-broadening of the ^{1}H NMR spectrum caused by paramagnetism of Yb^{III} hinders precise quantitation of different lanthanide ions in the assembled complexes through integration of the NMR spectra.

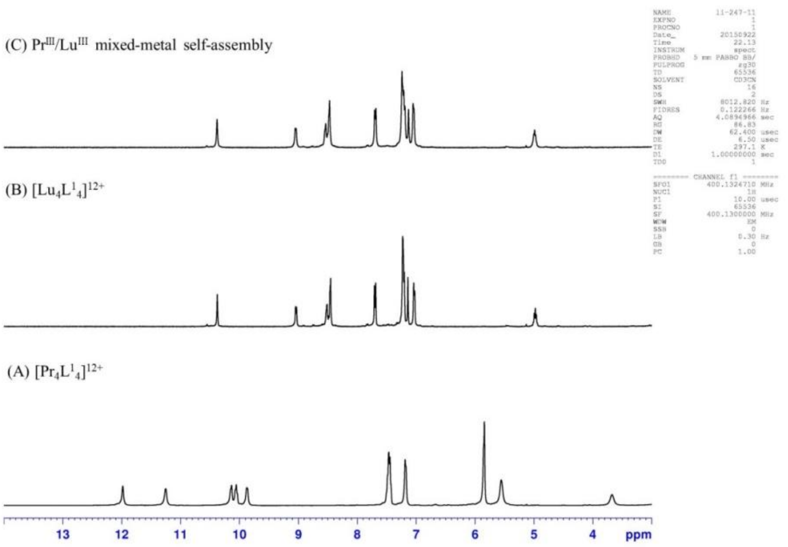

Supplementary Figure 162. ^{1}H NMR spectra (400 MHz, CD_3CN, 298K) for $[Pr_4L^1{}_4]^{12+}$ (A), $[Lu_4L^1{}_4]^{12+}$ (B) and Pr^{III}/Lu^{III} mixed-metal self-assembled complexes with L^1 (ClO_4^- salt) (C).
Complete metal ion self-recognition assembly (at 40 ℃ for 1h) of L^1 (0.01 M) with Lu^{III} was obtained according to the ^{1}H NMR characterization and no spectra change in (C) was observed even after 2 weeks.

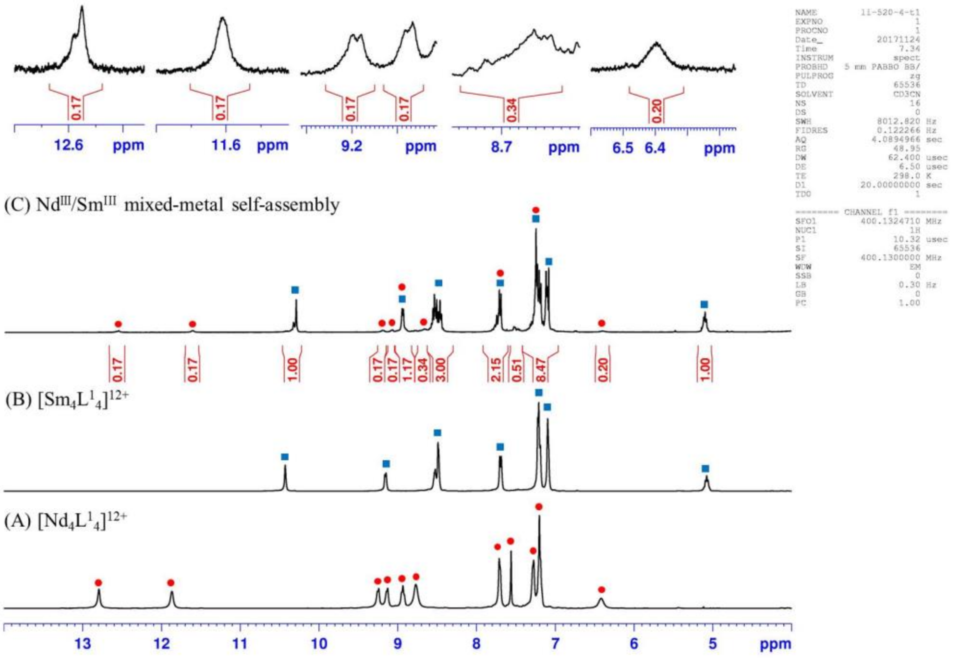

Supplementary Figure 163. ^{1}H NMR spectra (400 MHz, CD_3CN, 298K) for $[Nd_4L^1{}_4]^{12+}$ (A), $[Sm_4L^1{}_4]^{12+}$ (B) and Nd^{III}/Sm^{III} mixed-metal self-assembled complexes with L^1 ($CF_3SO_3^-$ salt) (C) with spectra zoomed in over some of the peak ranges selected for the integrals.
Non-absolute metal ion self-recognition assembly (at 40 ℃ for 1h) of L^1 (0.01 M) with Nd^{III}/Sm^{III} mixtures (0.01 M of each), with 85.47 percent Sm^{III} and 14.35 percent Nd^{III} in the assembled complexes, was observed according to the ^{1}H NMR characterization and no spectra change in (C) was observed even after 2 weeks.

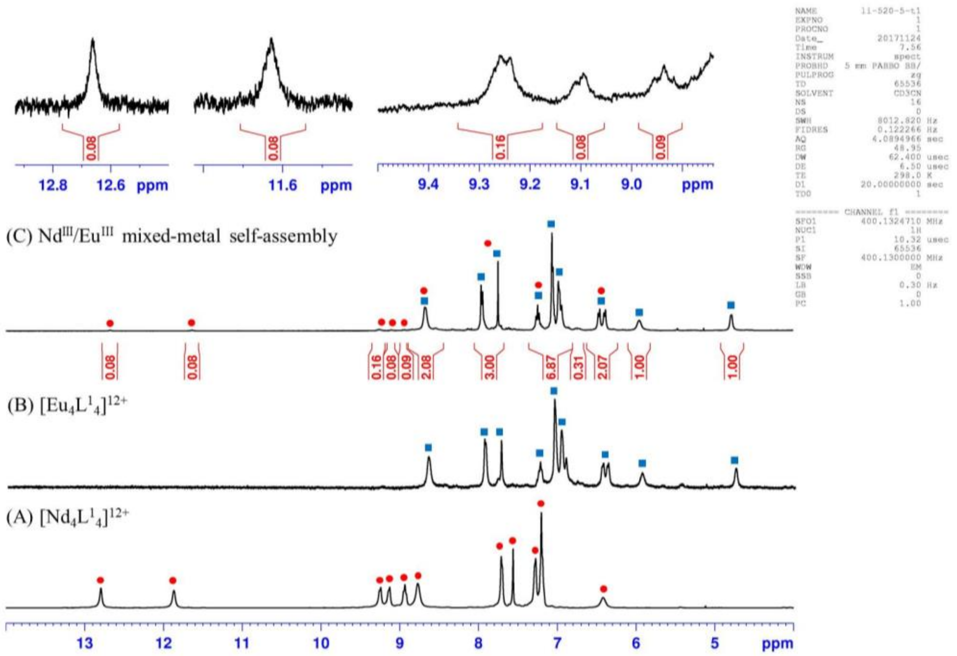

Supplementary Figure 164. ^{1}H NMR spectra (400 MHz, CD_3CN, 298K) for $[Nd_4L^1{}_4]^{12+}$ (A), $[Eu_4L^1{}_4]^{12+}$ (B) and Nd^{III}/Eu^{III} mixed-metal self-assembled complexes with L^1 ($CF_3SO_3^-$ salt) (C) with spectra zoomed in over some of the peak ranges selected for the integrals.

Non-absolute metal ion self-recognition assembly (at 40 ℃ for 1h) of L^1 (0.01 M) with Nd^{III}/Eu^{III} mixtures (0.01 M of each), with 92.59 percent Eu^{III} and 7.40 percent Nd^{III} in the assembled complexes, was observed according to the ^{1}H NMR characterization and no spectra change in (C) was observed even after 2 weeks.

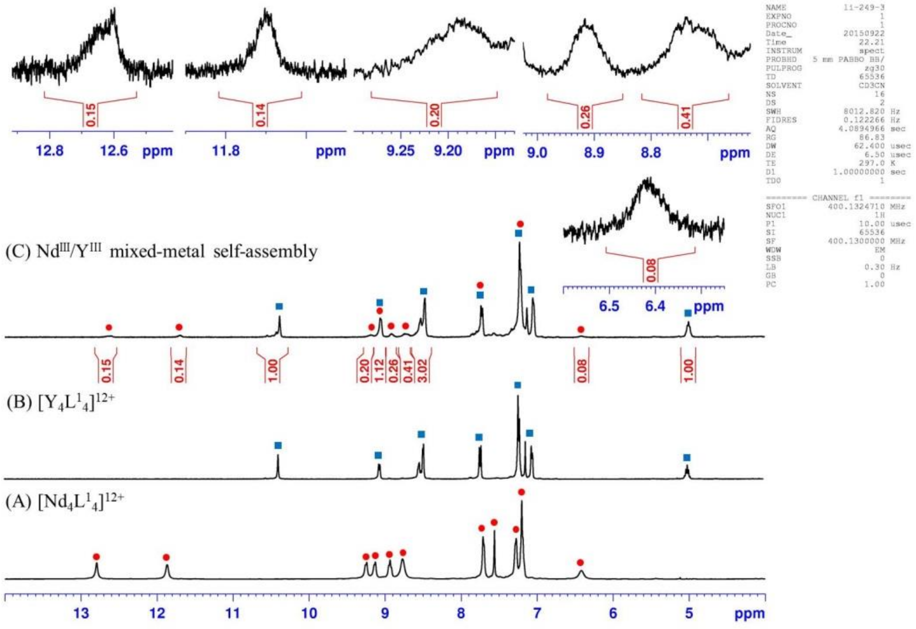

Supplementary Figure 165. ^{1}H NMR spectra (400 MHz, CD_3CN, 298K) $[Nd_4L^1{}_4]^{12+}$ (A), $[Y_4L^1{}_4]^{12+}$ (B) and Nd^{III}/Y^{III} mixed-metal self-assembled complexes with L^1 ($CF_3SO_3^-$ salt) (C) with spectra zoomed in over some of the peak ranges selected for the integrals.

Non-absolute metal ion self-recognition assembly (at 40 ℃ for 1h) of L^1 (0.01 M) with Nd^{III}/Y^{III} mixtures (0.01 M of each), with 83.33 percent Y^{III} and 16.67 percent Nd^{III} in the assembled complexes, was observed according to the ^{1}H NMR characterization and no spectra change in (C) was observed even after 2 weeks.

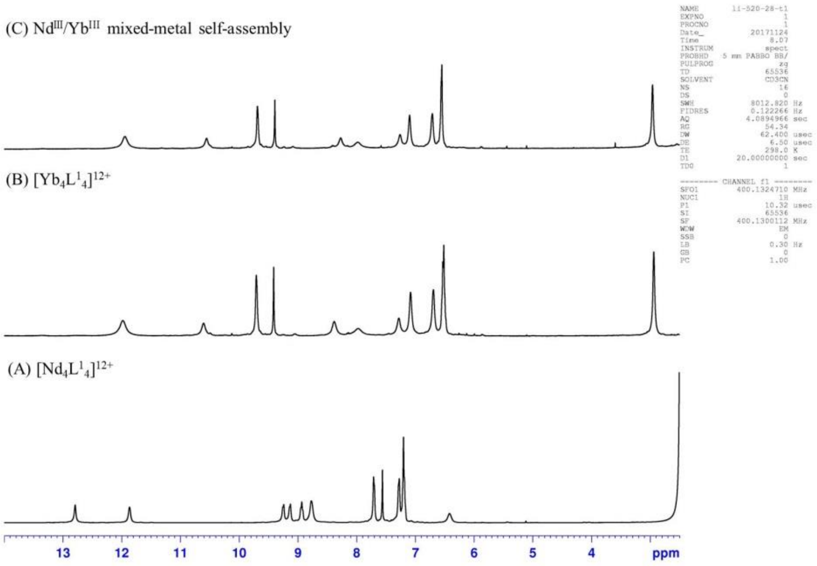

Supplementary Figure 166. ^{1}H NMR spectra (400 MHz, CD_3CN, 298K) for $[Nd_4L^1{}_4]^{12+}$ (A), $[Yb_4L^1{}_4]^{12+}$ (B) and Nd^{III}/Yb^{III} mixed-metal self-assembled complexes with L^1 ($CF_3SO_3^-$ salt) (C).

ESI-TOF-MS measurement indicates non-absolute metal ion self-recognition assembly of L^1 with Ce^{III}/Yb^{III} mixtures. However, the severe line-broadening of the ^{1}H NMR spectrum caused by paramagnetism of Yb^{III} hinders precise quantitation of different lanthanide ions in the assembled complexes through integration of the NMR spectra.

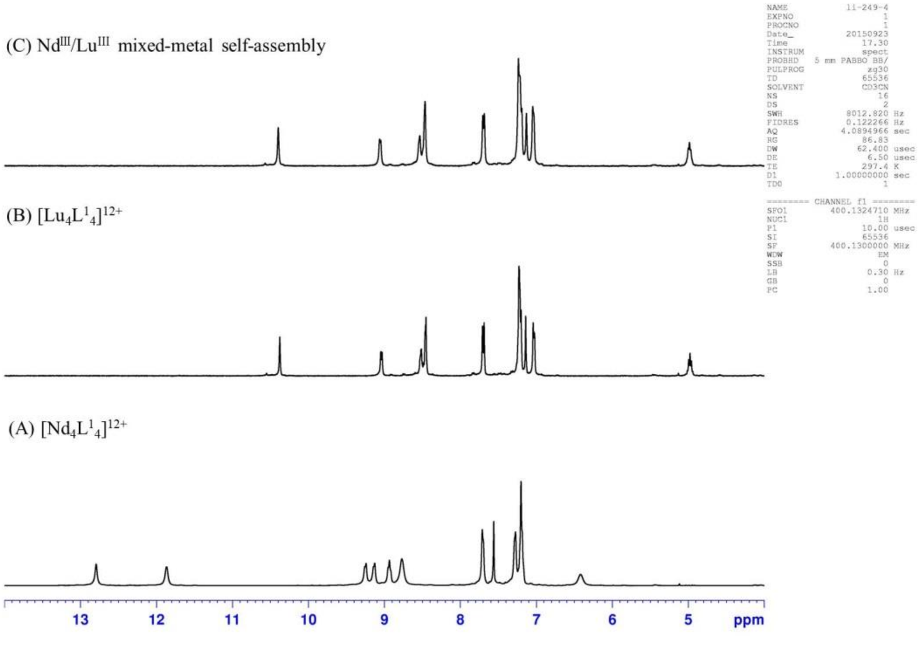

Supplementary Figure 167. ^{1}H NMR spectra (400 MHz, CD_3CN, 298K) for $[Nd_4L^1{}_4]^{12+}$ (A), $[Lu_4L^1{}_4]^{12+}$ (B) and Nd^{III}/Lu^{III} mixed-metal self-assembled complexes with L^1 (ClO_4^- salt) (C).

Complete metal ion self-recognition assembly (at 40 ℃ for 1h) of L^1 (0.01 M) with Lu^{III} was obtained according to the ^{1}H NMR characterization and no spectra change in (C) was observed even after 2 weeks.

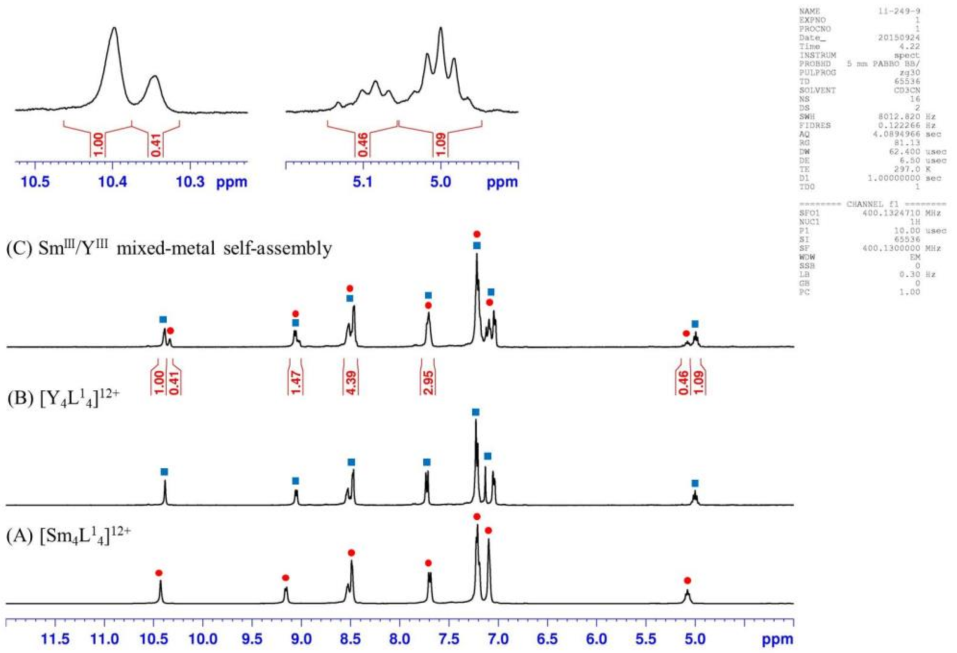

Supplementary Figure 168. ^{1}H NMR spectra (400 MHz, CD_3CN, 298K) for $[Sm_4L^1_4]^{12+}$ (A), $[Y_4L^1_4]^{12+}$ (B) and Sm^{III}/Y^{III} mixed-metal self-assembled complexes with L^1 ($CF_3SO_3^-$ salt) (C) with spectra zoomed in over some of the peak ranges selected for the integrals.

Non-absolute metal ion self-recognition assembly (at 40 ℃ for 1h) of L^1 (0.01 M) with Sm^{III}/Y^{III} mixtures (0.01 M of each), with 68.49 percent Y^{III} and 31.51 percent Sm^{III} in the assembled complexes, was observed according to the ^{1}H NMR characterization and no spectra change in (C) was observed even after 2 weeks.

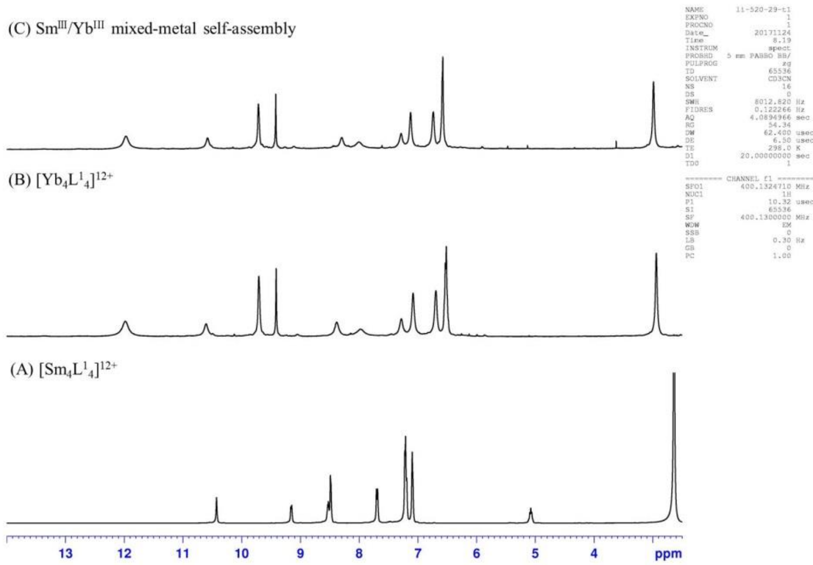

Supplementary Figure 169. ^{1}H NMR spectra (400 MHz, CD_3CN, 298K) for $[Sm_4L^1_4]^{12+}$ (A), $[Yb_4L^1_4]^{12+}$ (B) and Sm^{III}/Yb^{III} mixed-metal self- assembled complexes with L^1 ($CF_3SO_3^-$ salt) (C).

ESI-TOF-MS measurement indicates non-absolute metal ion self-recognition assembly of L^1 with Ce^{III}/Yb^{III} mixtures. However, the severe line-broadening of the ^{1}H NMR spectrum caused by paramagnetism of Yb^{III} hinders precise quantitation of different lanthanide ions in the assembled complexes through integration of the NMR spectra.

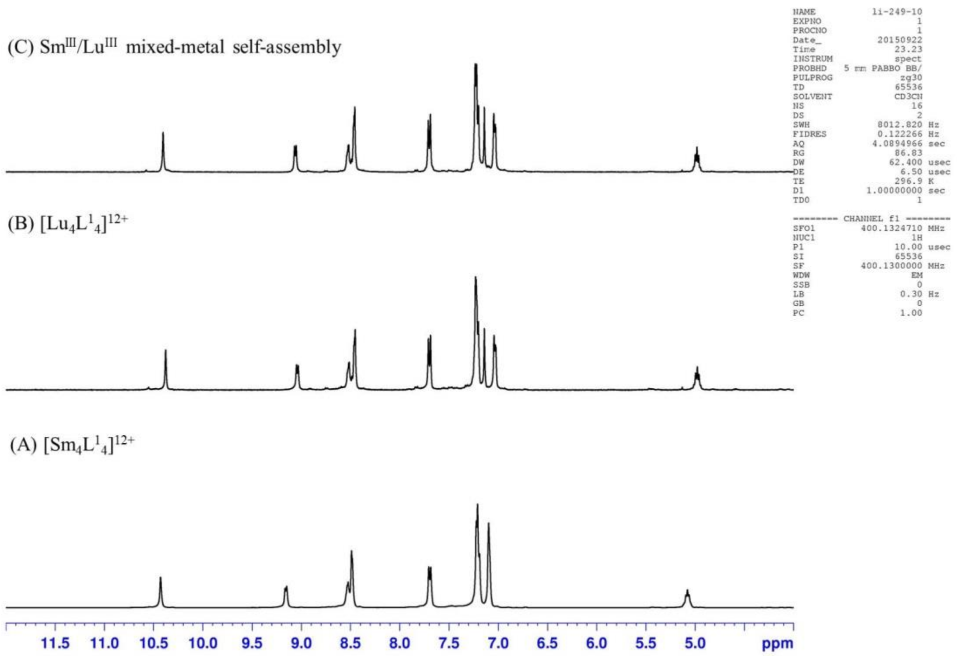

Supplementary Figure 170. ^{1}H NMR spectra (400 MHz, CD_3CN, 298K) for $[Sm_4L^1{}_4]^{12+}$ (A), $[Lu_4L^1{}_4]^{12+}$ (B) and Sm^{III}/Lu^{III} mixed-metal self- assembled complexes with L^1 (ClO_4^- salt) (C).

Complete metal ion self-recognition assembly (at 40 ℃ for 1h) of L^1 (0.01 M) with Lu^{III} was obtained according to the ^{1}H NMR characterization and no spectra change in (C) was observed even after 2 weeks.

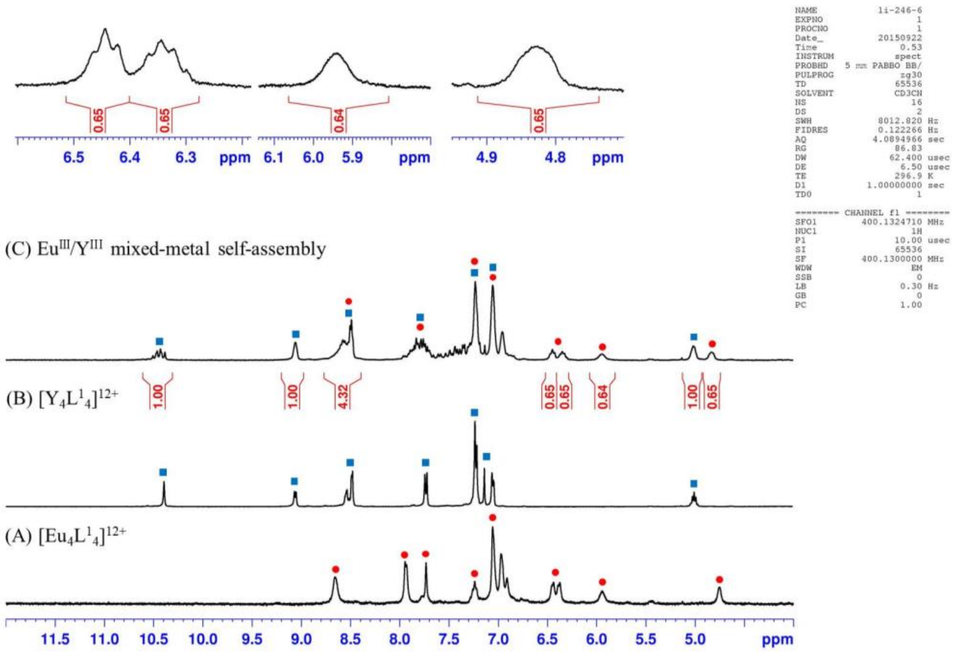

Supplementary Figure 171. ^{1}H NMR spectra (400 MHz, CD_3CN, 298K) for $[Eu_4L^1{}_4]^{12+}$ (A), $[Y_4L^1{}_4]^{12+}$ (B) and Eu^{III}/Y^{III} mixed-metal self-assembled complexes with L^1 ($CF_3SO_3^-$ salt) (C) with spectra zoomed in over some of the peak ranges selected for the integrals.

Non-absolute metal ion self-recognition assembly (at 40 ℃ for 1h) of L^1 (0.01 M) with Eu^{III}/Y^{III} mixtures (0.01 M of each), with 60.61 percent Y^{III} and 39.39 percent Eu^{III} in the assembled complexes, was observed according to the ^{1}H NMR characterization and no spectra change in (C) was observed even after 2 weeks.

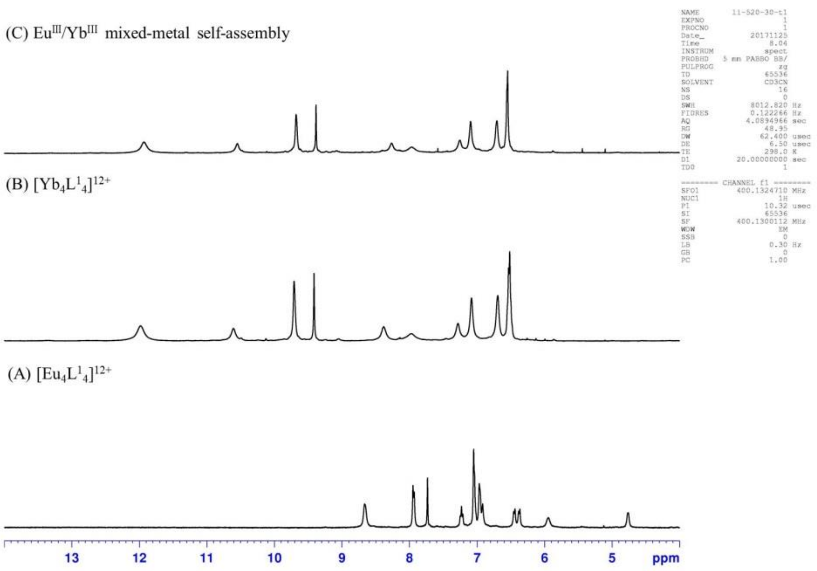

Supplementary Figure 172. ^{1}H NMR spectra (400 MHz, CD_3CN, 298K) for $[Eu_4L^1_4]^{12+}$ (A), $[Yb_4L^1_4]^{12+}$ (B) and Eu^{III}/Yb^{III} mixed-metal self-assembled complexes with L^1 ($CF_3SO_3^-$ salt) (C).

ESI-TOF-MS measurement indicates non-absolute metal ion self-recognition assembly of L^1 with Ce^{III}/Yb^{III} mixtures. However, the severe line-broadening of the ^{1}H NMR spectrum caused by paramagnetism of Yb^{III} hinders precise quantitation of different lanthanide ions in the assembled complexes through integration of the NMR spectra.

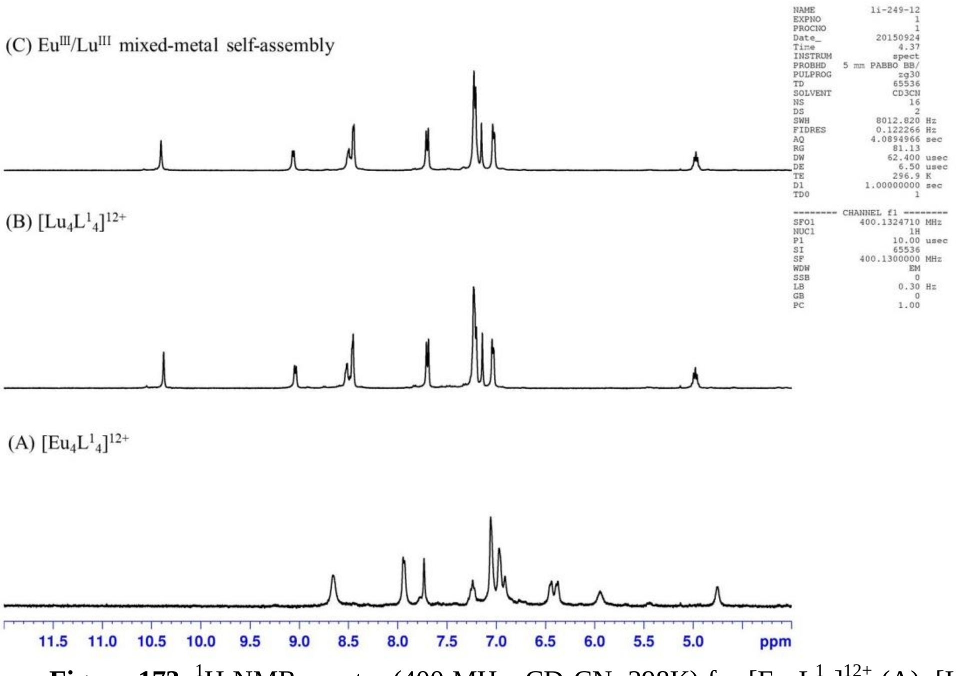

Supplementary Figure 173. ^{1}H NMR spectra (400 MHz, CD_3CN, 298K) for $[Eu_4L^1_4]^{12+}$ (A), $[Lu_4L^1_4]^{12+}$ (B) and Eu^{III}/Lu^{III} mixed-metal self-assembled complexes with L^1 (ClO_4^- salt) (C).

Complete metal ion self-recognition assembly (at 40 ℃ for 1h) of L^1 (0.01 M) with Lu^{III} was obtained according to the ^{1}H NMR characterization and no spectra change in (C) was observed even after 2 weeks.

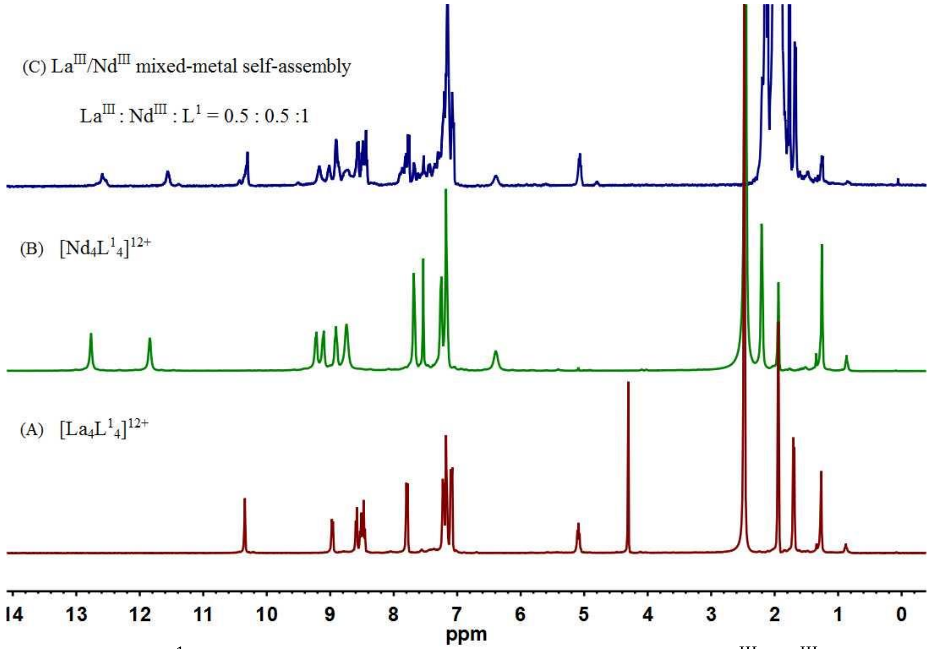

Supplementary Figure 174. ^{1}H NMR spectra (400 MHz, CD_3CN, 298K) of La^{III}/Nd^{III} mixed-metal self-assembled complexes with L^1 (ClO_4^- salt) with stoichiometric ratio as La^{III} : Nd^{III} : L^1 = 0.5 : 0.5 :1.

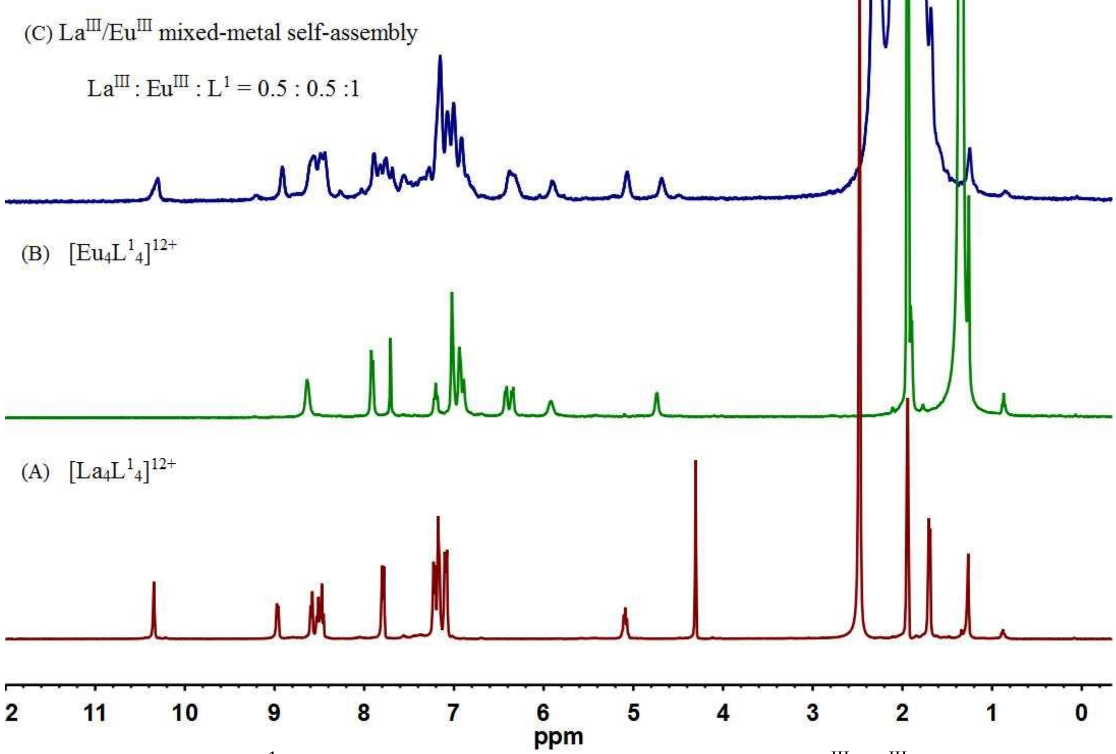

Supplementary Figure 175. ^{1}H NMR spectra (400 MHz, CD_3CN, 298K) of La^{III}/Eu^{III} mixed-metal self-assembled complexes with L^1 ($CF_3SO_3^-$ salt) with the metal and ligand ratio as La^{III} : Eu^{III} : L^1 = 0.5 : 0.5 :1.

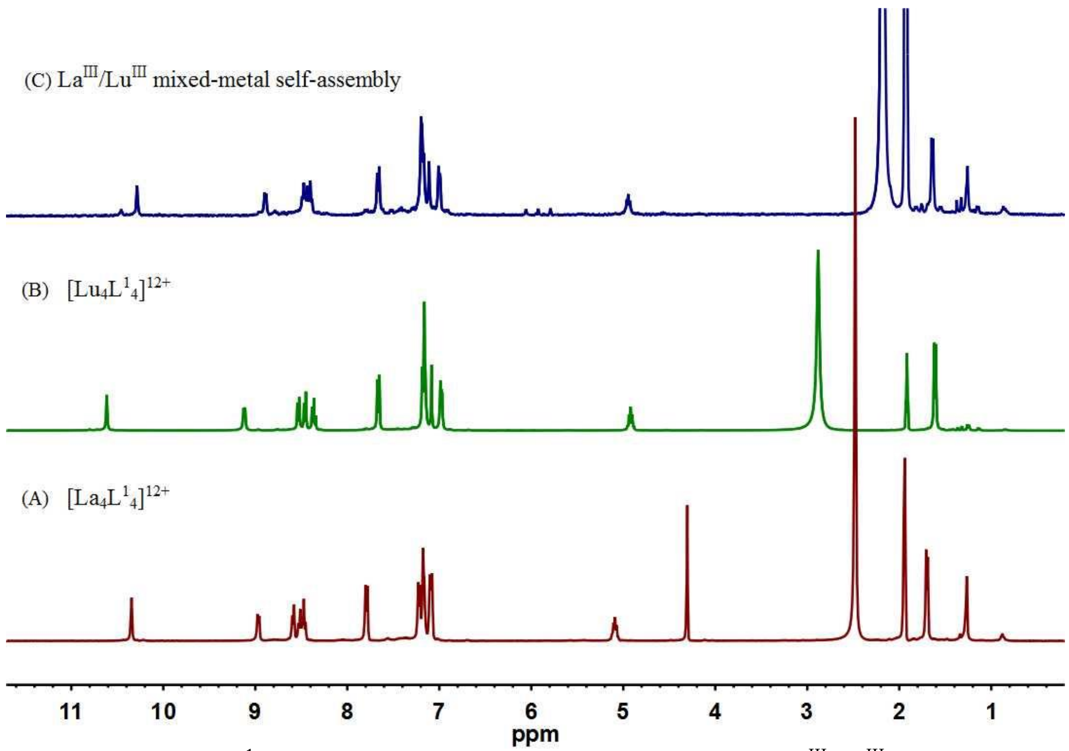

Supplementary Figure 176. ^{1}H NMR spectra (400 MHz, CD_3CN, 298K) of La^{III}/Lu^{III} mixed-metal self-assembled complexes with L^1 ($CF_3SO_3^-$ salt) with the metal and ligand ratio as La^{III} : Lu^{III} : L^1 = 0.5 : 0.5 :1.

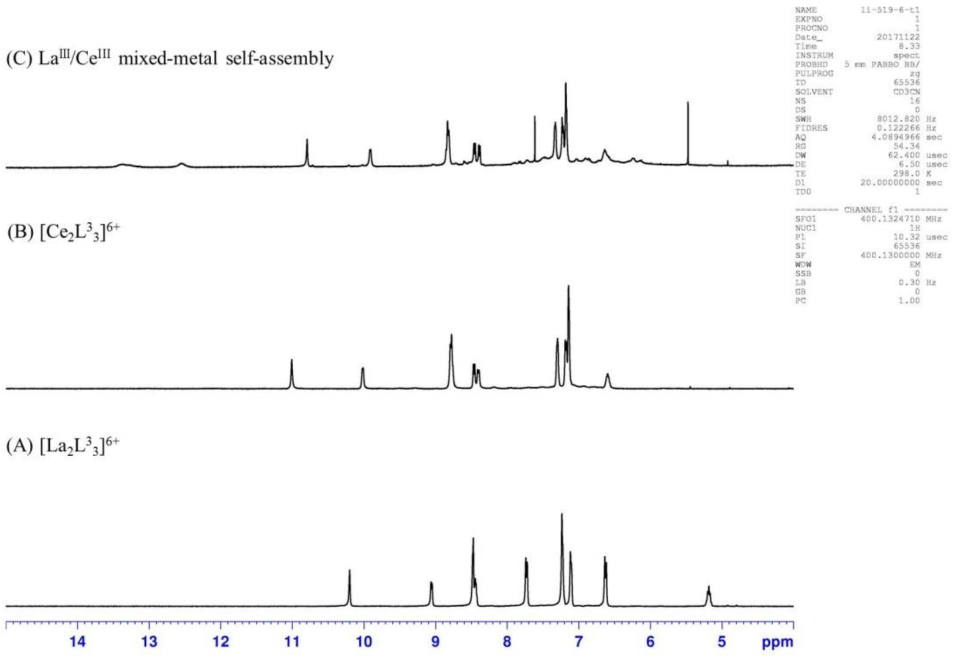

Supplementary Figure 177. ^{1}H NMR spectra (400 MHz, CD_3CN, 298K) for $[La_2L^3_3]^{6+}$ (A), $[Ce_2L^3_3]^{6+}$ (B) and La^{III}/Ce^{III} mixed-metal self-assembled complexes with L^3 (ClO_4^- salt) (C).

The unattributable peaks in the range of 12-13.5ppm in the above spectrum (C) make it difficult to determine the proportion of two kinds of metal ions through integration of the ^{1}H NMR spectrum.

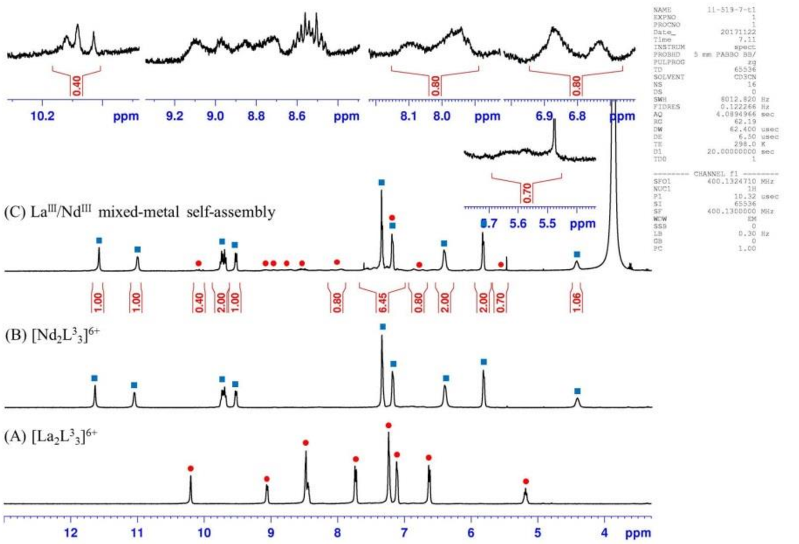

Supplementary Figure 178. ^{1}H NMR spectra (400 MHz, CD_3CN, 298K) for $[La_2L^3{}_3]^{6+}$ (A), $[Pr_2L^3{}_3]^{6+}$ (B) and La^{III}/Pr^{III} mixed-metal self-assembled complexes with L^3 (ClO_4^- salt) (C) with spectra zoomed in over some of the peak ranges selected for the integrals.

Non-absolute metal ion self-recognition assembly (at 40 ℃ for 1h) of L^3 (0.0075 M) with La^{III}/Pr^{III} mixtures (0.005 M of each), with 71.43 percent Pr^{III} and 28.57 percent La^{III} in the assembled complexes, was observed according to the ^{1}H NMR characterization and no spectra change in (C) was observed even after 2 weeks.

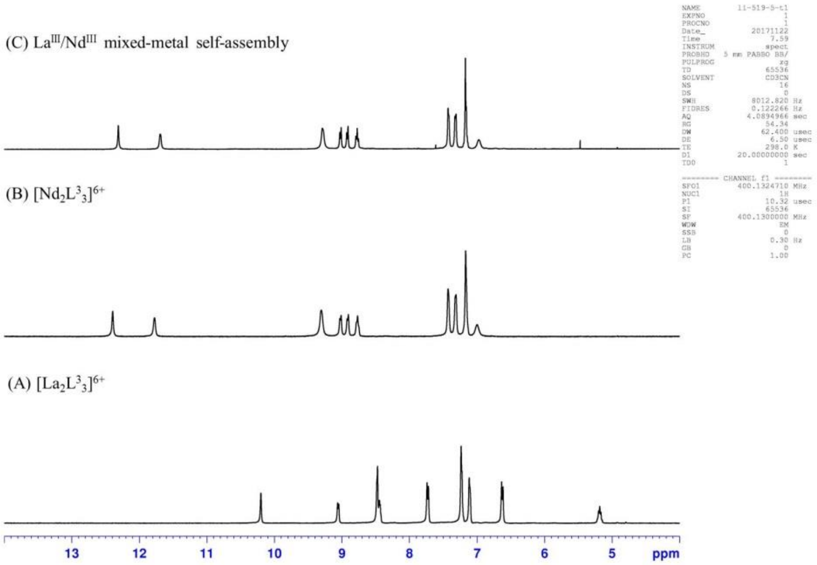

Supplementary Figure 179. ^{1}H NMR spectra (400 MHz, CD_3CN, 298K) for $[La_2L^3{}_3]^{6+}$ (A), $[Nd_2L^3{}_3]^{6+}$ (B) and La^{III}/Nd^{III} mixed-metal self-assembled complexes with L^3 (ClO_4^- salt) (C) with spectra zoomed in over some of the peak ranges selected for the integrals.

No spectra change in (C) was observed even after 2 weeks.

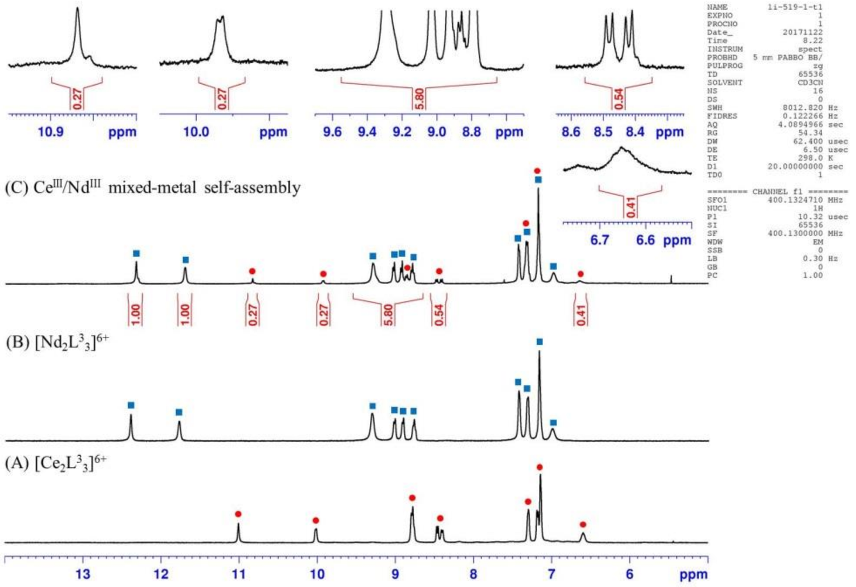

Supplementary Figure 180. ^{1}H NMR spectra (400 MHz, CD_3CN, 298K) for $[Ce_2L^3{}_3]^{6+}$ (A), $[Nd_2L^3{}_3]^{6+}$ (B) and Ce^{III}/Nd^{III} mixed-metal self-assembled complexes with L^3 (ClO_4^- salt) (C) with spectra zoomed in over some of the peak ranges selected for the integrals.

Non-absolute metal ion self-recognition assembly (at 40 ℃ for 1h) of L^3 (0.0075 M) with Ce^{III}/Nd^{III} mixtures (0.005 M of each), with 78.74 percent Nd^{III} and 21.26 percent Ce^{III} in the assembled complexes, was observed according to the ^{1}H NMR characterization and no spectra change in (C) was observed even after 2 weeks.

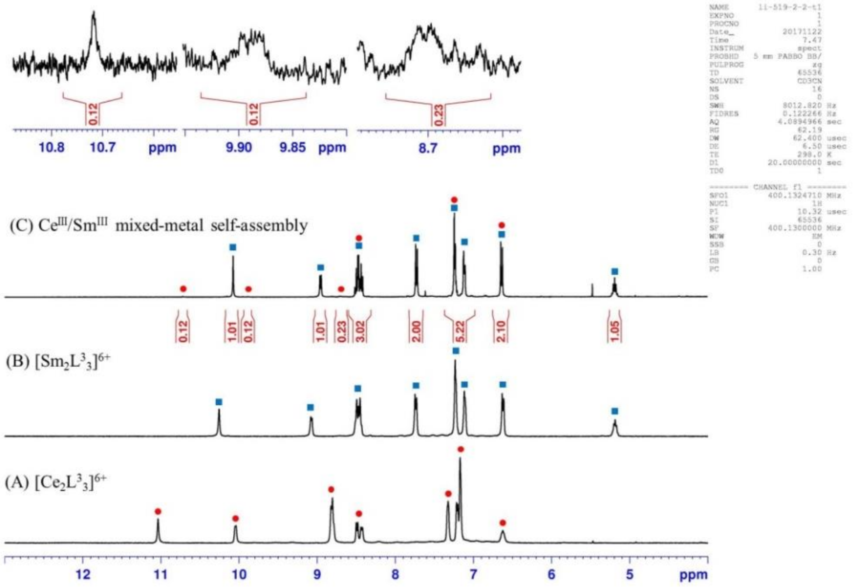

Supplementary Figure 181. ^{1}H NMR spectra (400 MHz, CD_3CN, 298K) for $[Ce_2L^3{}_3]^{6+}$ (A), $[Sm_2L^3{}_3]^{6+}$ (B) and Ce^{III}/Sm^{III} mixed-metal self-assembled complexes with L^3 ($CF_3SO_3^-$ salt) (C) with spectra zoomed in over some of the peak ranges selected for the integrals.

Non-absolute metal ion self-recognition assembly (at 40 ℃ for 1h) of L^3 (0.0075 M) with Ce^{III}/Sm^{III} mixtures (0.005 M of each), with 89.29 percent Sm^{III} and 10.71 percent Ce^{III} in the assembled complexes, was observed according to the ^{1}H NMR characterization and no spectra change in (C) was observed even after 2 weeks.

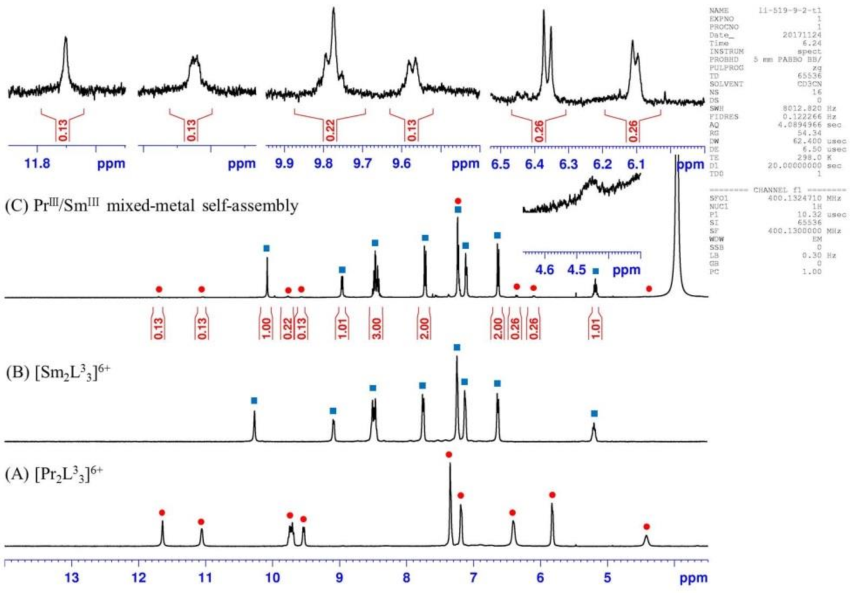

Supplementary Figure 182. ^{1}H NMR spectra (400 MHz, CD_3CN, 298K) for $[Pr_2L^3_3]^{6+}$ (A), $[Sm_2L^3_3]^{6+}$ (B) and Pr^{III}/Sm^{III} mixed-metal self-assembled complexes with L^3 ($CF_3SO_3^-$ salt) (C) with spectra zoomed in over some of the peak ranges selected for the integrals.

Non-absolute metal ion self-recognition assembly (at 40 ℃ for 1h) of L^3 (0.0075 M) with Pr^{III}/Sm^{III} mixtures (0.005 M of each), with 88.50 percent Sm^{III} and 11.50 percent Pr^{III} in the assembled complexes, was observed according to the ^{1}H NMR characterization and no spectra change in (C) was observed even after 2 weeks.

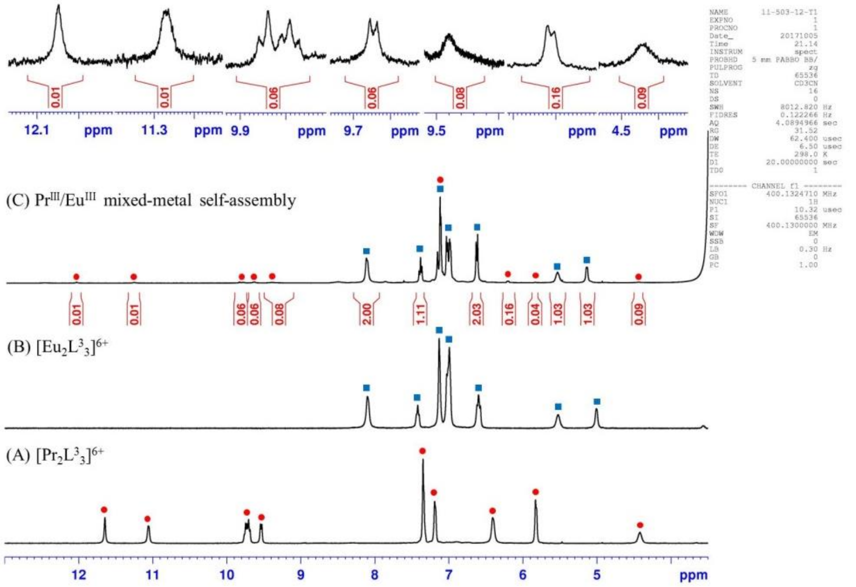

Supplementary Figure 183. ^{1}H NMR spectra (400 MHz, CD_3CN, 298K) for $[Pr_2L^3_3]^{6+}$ (A), $[Eu_2L^3_3]^{6+}$ (B) and Pr^{III}/Eu^{III} mixed-metal self-assembled complexes with L^3 ($CF_3SO_3^-$ salt) (C) with spectra zoomed in over some of the peak ranges selected for the integrals.

Non-absolute metal ion self-recognition assembly (at 40 ℃ for 1h) of L^3 (0.0075 M) with Pr^{III}/Eu^{III} mixtures (0.005 M of each), with 92.59 percent Eu^{III} and 7.41 percent Pr^{III} in the assembled complexes, was observed according to the ^{1}H NMR characterization and no spectra change in (C) was observed even after 2 weeks.

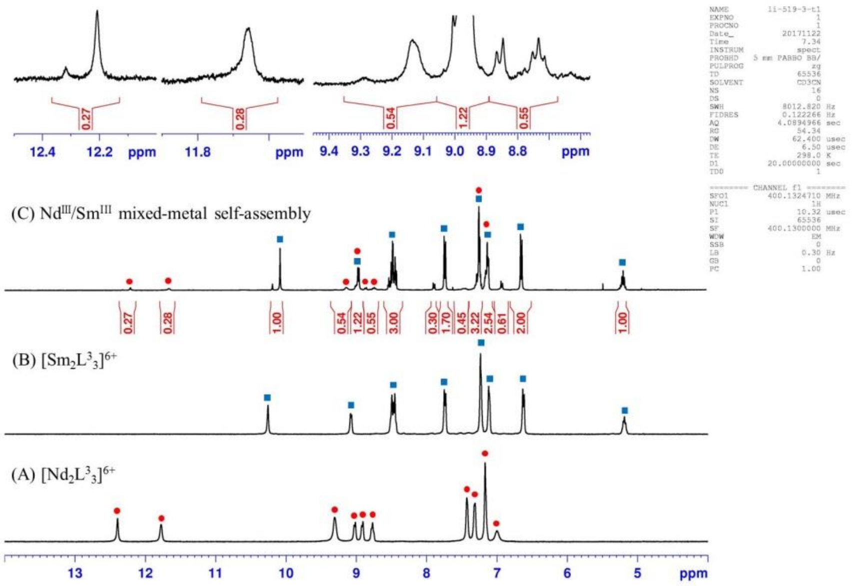

Supplementary Figure 184. ^{1}H NMR spectra (400 MHz, CD_3CN, 298K) for $[Nd_2L^3_3]^{6+}$ (A), $[Sm_2L^3_3]^{6+}$ (B) and Nd^{III}/Sm^{III} mixed-metal self-assembled complexes with L^3 (ClO_4^- salt) (C) with spectra zoomed in over some of the peak ranges selected for the integrals.
Non-absolute metal ion self-recognition assembly (at 40 ℃ for 1h) of L^3 (0.0075 M) with Nd^{III}/Sm^{III} mixtures (0.005 M of each), with 78.74 percent Sm^{III} and 21.26 percent Nd^{III} in the assembled complexes, was observed according to the ^{1}H NMR characterization and no spectra change in (C) was observed even after 2 weeks.

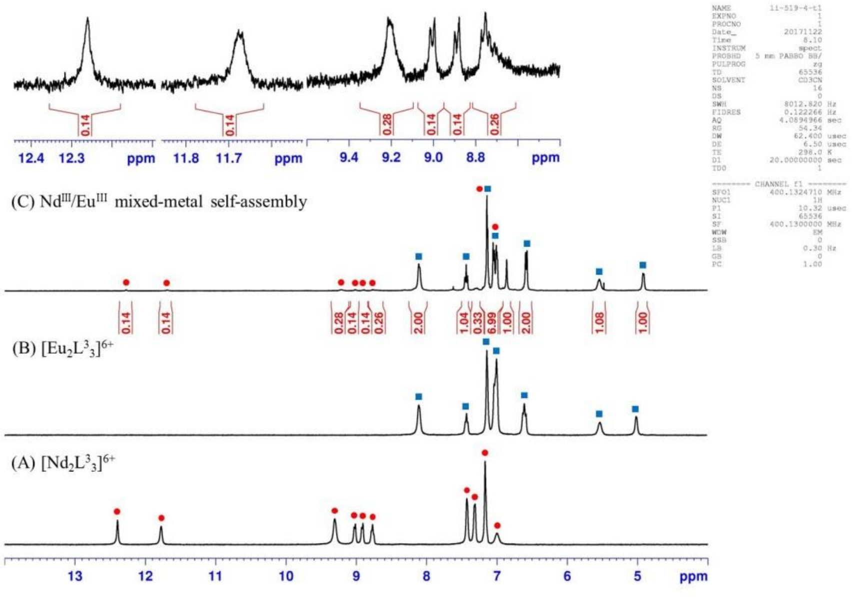

Supplementary Figure 185. ^{1}H NMR spectra (400 MHz, CD_3CN, 298K) for $[Nd_2L^3_3]^{6+}$ (A), $[Eu_2L^3_3]^{6+}$ (B) and Nd^{III}/Eu^{III} mixed-metal self-assembled complexes with L^3 (ClO_4^- salt) (C) with spectra zoomed in over some of the peak ranges selected for the integrals.
Non-absolute metal ion self-recognition assembly (at 40 ℃ for 1h) of L^3 (0.0075 M) with Nd^{III}/Eu^{III} mixtures (0.005 M of each), with 87.72 percent Eu^{III} and 12.28 percent Nd^{III} in the assembled complexes, was observed according to the ^{1}H NMR characterization and no spectra change in (C) was observed even after 2 weeks.

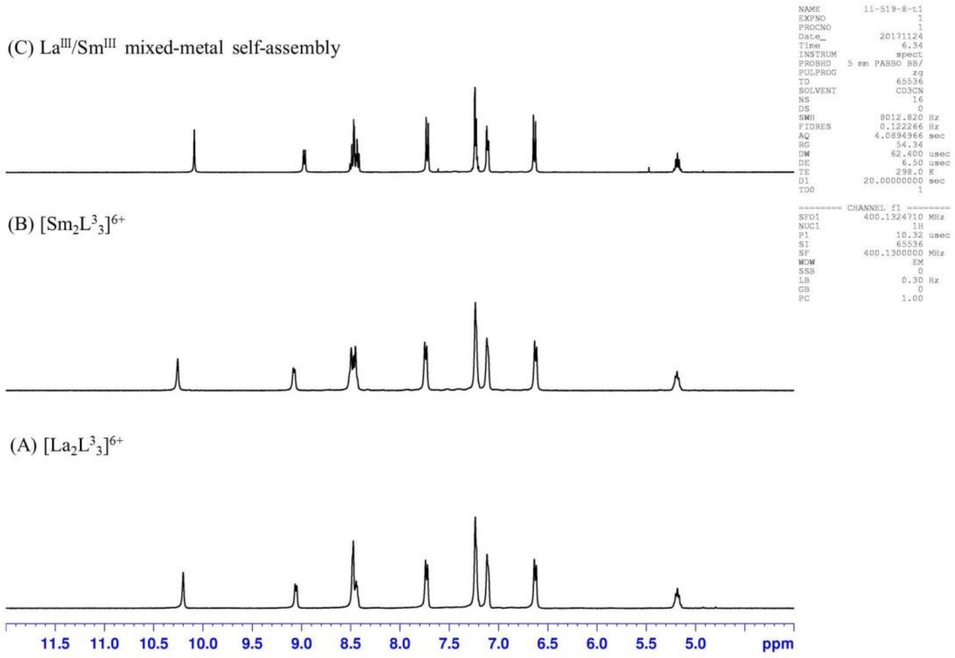

Supplementary Figure 186. ^{1}H NMR spectra (400 MHz, CD_3CN, 298K) for $[La_2L^3{}_3]^{6+}$ (A), $[Sm_2L^3{}_3]^{6+}$ (B) and La^{III}/Sm^{III} mixed-metal self-assembled complexes with L^3 ($CF_3SO_3^-$ salt) (C).

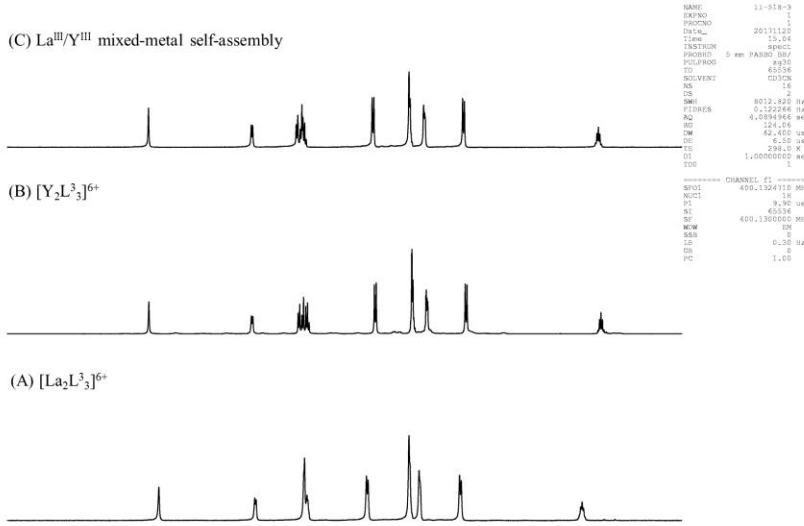

Supplementary Figure 187. ^{1}H NMR spectra (400 MHz, CD_3CN, 298K) for $[La_2L^3{}_3]^{6+}$ (A), $[Y_2L^3{}_3]^{6+}$ (B) and La^{III}/Y^{III} mixed-metal self-assembled complexes with L^3 ($CF_3SO_3^-$ salt) (C).

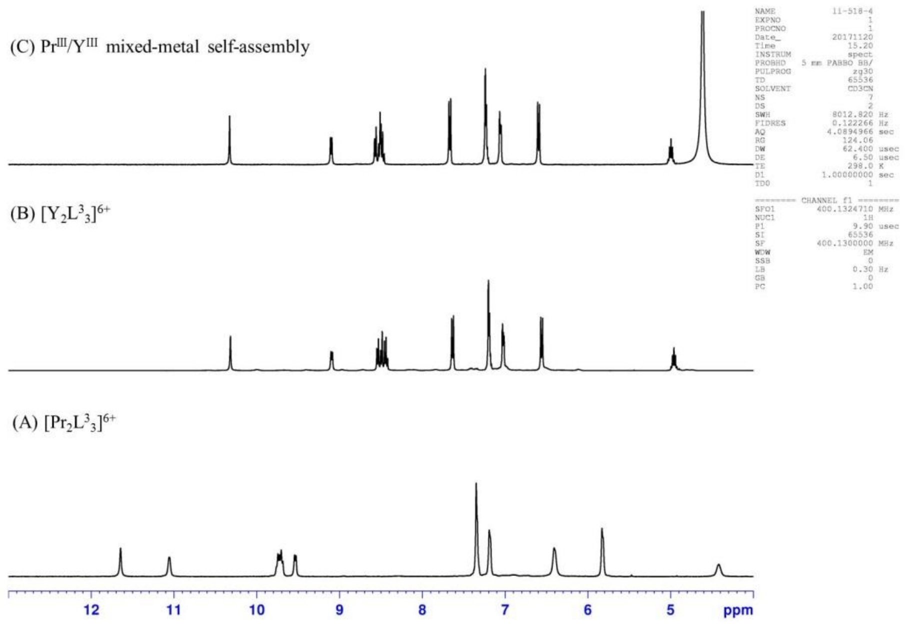

Supplementary Figure 188. ^{1}H NMR spectra (400 MHz, CD_3CN, 298K) for $[Pr_2L^3_3]^{6+}$ (A), $[Y_2L^3_3]^{6+}$ (B) and Pr^{III}/Y^{III} mixed-metal self-assembled complexes with L^3 ($CF_3SO_3^-$ salt) (C).

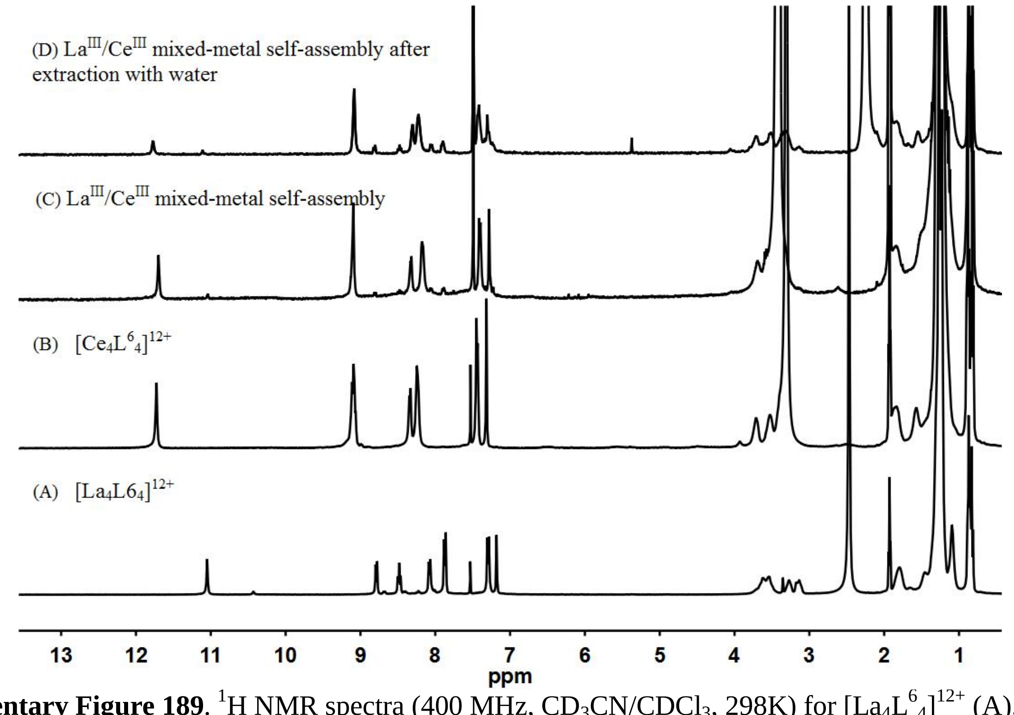

Supplementary Figure 189. ^{1}H NMR spectra (400 MHz, $CD_3CN/CDCl_3$, 298K) for $[La_4L^6_4]^{12+}$ (A), $[Ce_4L^6_4]^{12+}$ (B), La^{III}/Ce^{III} mixed-metal self-assembled (C) and La^{III}/Ce^{III} mixed-metal self-assembled complexes after extraction with water (D)($CF_3SO_3^-$ salt).

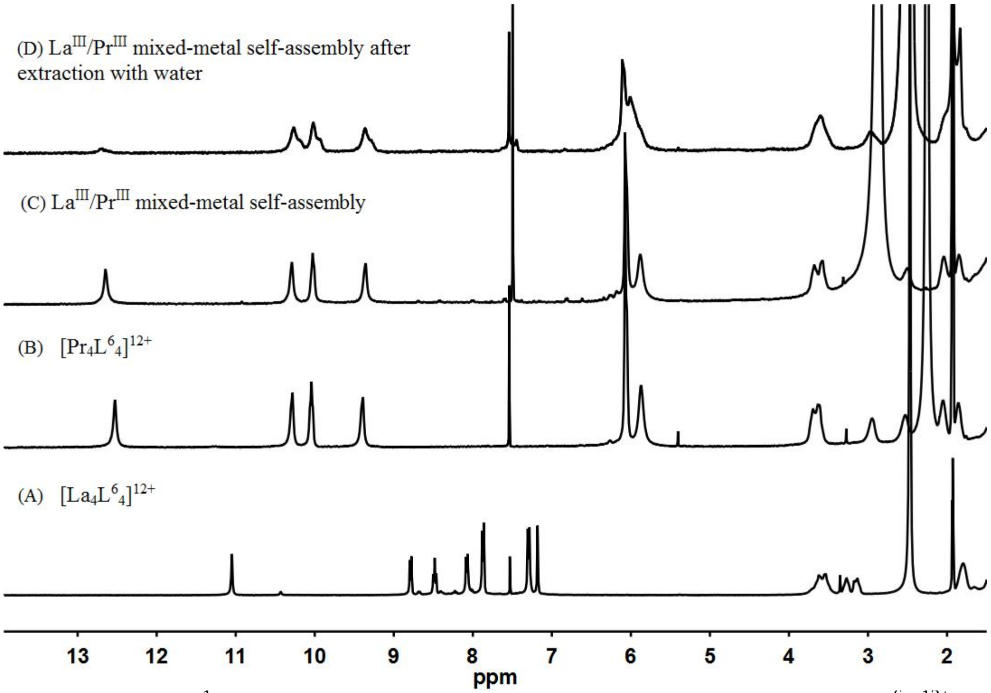

Supplementary Figure 190. ^{1}H NMR spectra (400 MHz, $CD_3CN/CDCl_3$, 298K) for $[La_4L^6{}_4]^{12+}$ (A), $[Pr_4L^6{}_4]^{12+}$ (B), La^{III}/Pr^{III} mixed-metal self-assembled (C) and La^{III}/Pr^{III} mixed-metal self-assembled complexes after extraction with water (D)($CF_3SO_3^-$ salt).

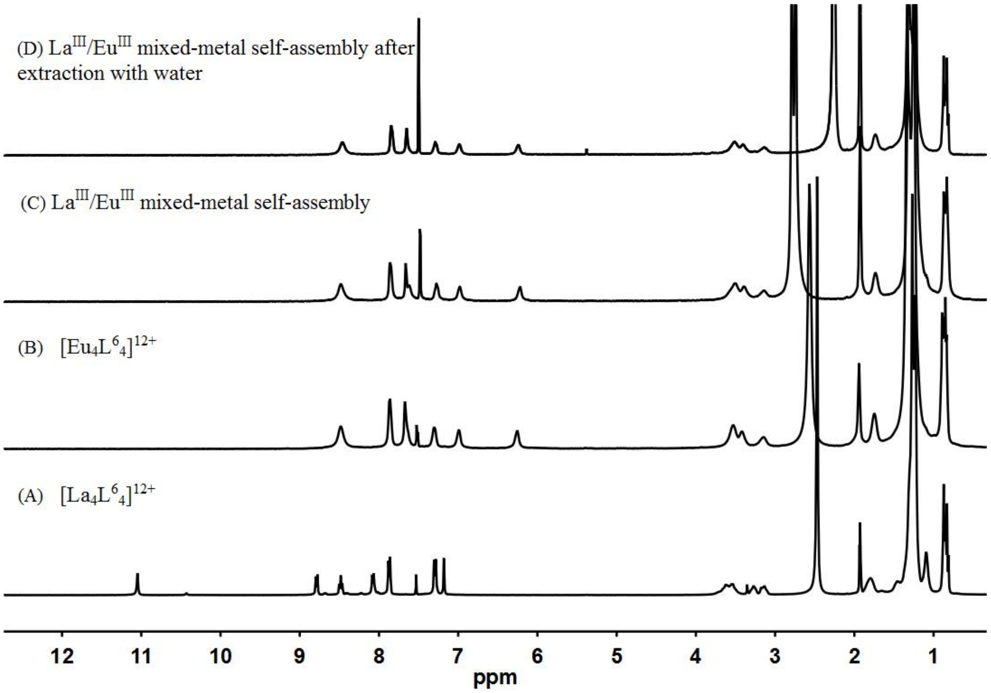

Supplementary Figure 191. ^{1}H NMR spectra (400 MHz, $CD_3CN/CDCl_3$, 298K) for $[La_4L^6{}_4]^{12+}$ (A), $[Eu_4L^6{}_4]^{12+}$ (B), La^{III}/Eu^{III} mixed-metal self-assembled (C) and La^{III}/Eu^{III} mixed-metal self-assembled complexes after extraction with water (D)($CF_3SO_3^-$ salt).

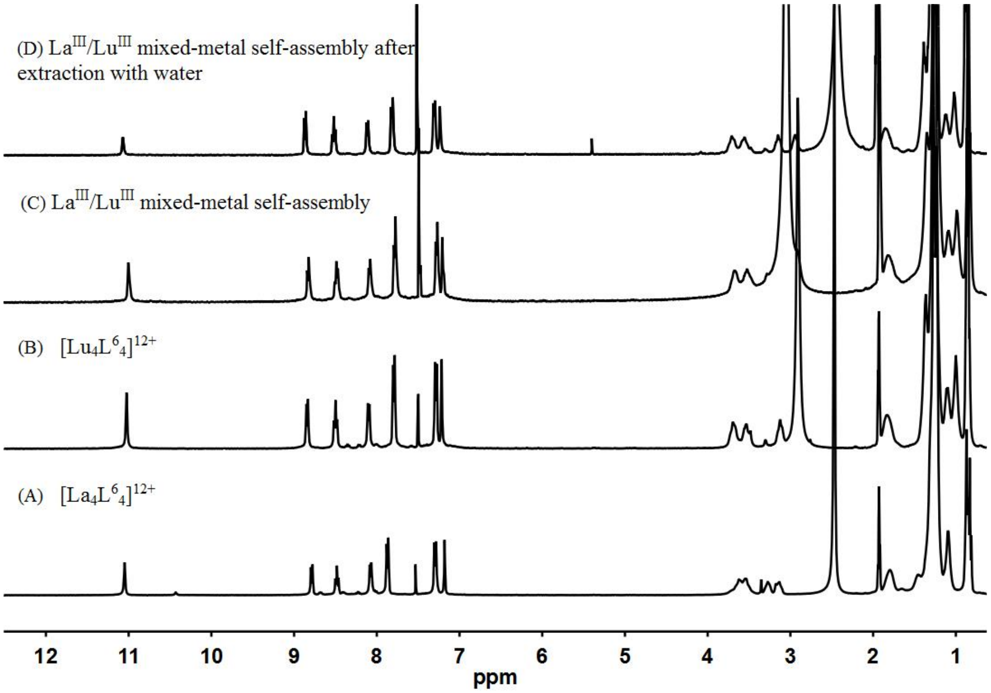

Supplementary Figure 192. ^{1}H NMR spectra (400 MHz, $CD_3CN/CDCl_3$, 298K) for $[La_4L^6{}_4]^{12+}$ (A), $[Lu_4L^6{}_4]^{12+}$ (B), La^{III}/Lu^{III} mixed-metal self-assembled (C) and La^{III}/Lu^{III} mixed-metal self-assembled complexes after extraction with water (D)($CF_3SO_3^-$ salt).

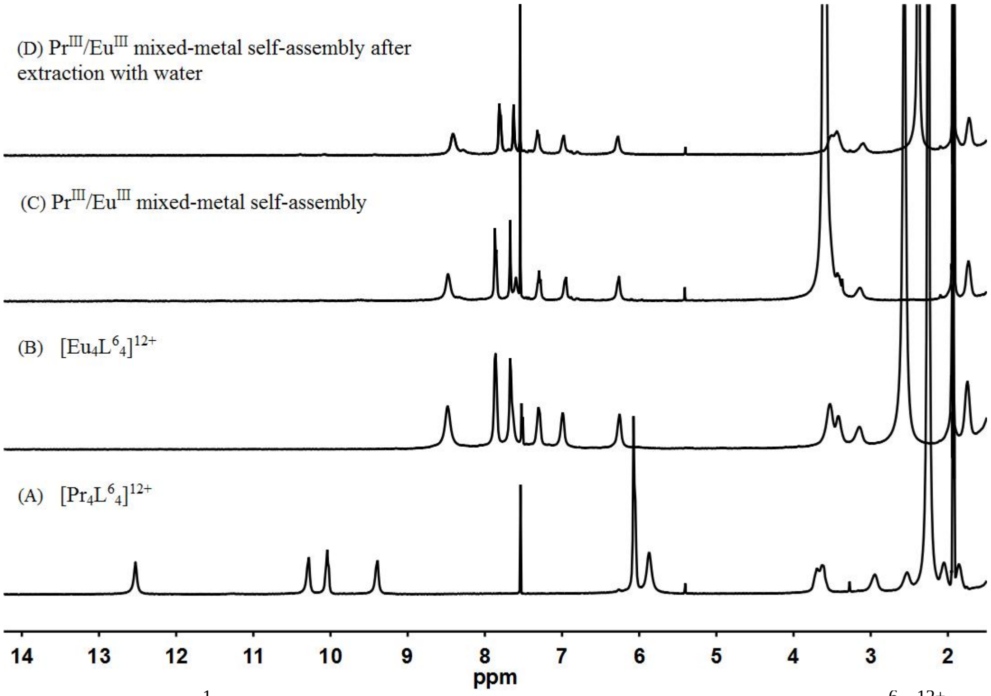

Supplementary Figure 193. ^{1}H NMR spectra (400 MHz, $CD_3CN/CDCl_3$, 298K) for $[Pr_4L^6{}_4]^{12+}$ (A), $[Eu_4L^6{}_4]^{12+}$ (B), Pr^{III}/Eu^{III} mixed-metal self-assembled (C) and Pr^{III}/Eu^{III} mixed-metal self-assembled complexes after extraction with water (D) ($CF_3SO_3^-$ salt).

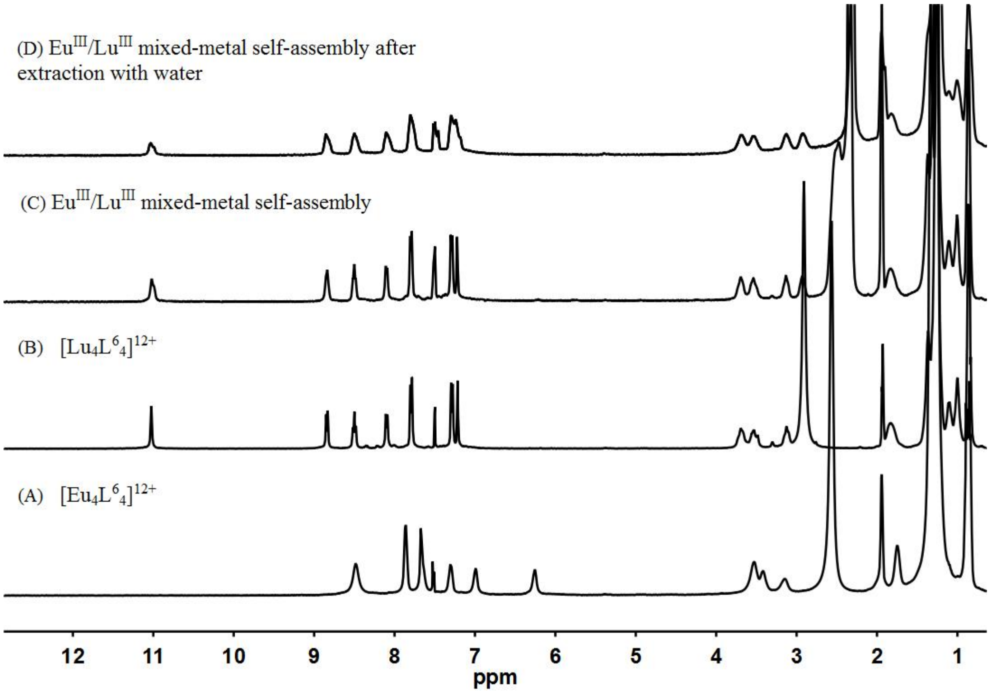

Supplementary Figure 194. ^{1}H NMR spectra (400 MHz, $CD_3CN/CDCl_3$, 298K) for $[Eu_4L^6{}_4]^{12+}$ (A), $[Lu_4L^6{}_4]^{12+}$ (B), Eu^{III}/Lu^{III} mixed-metal self-assembled (C)and Eu^{III}/Lu^{III} mixed-metal self-assembled complexes after extraction with water (D) ($CF_3SO_3^-$ salt).

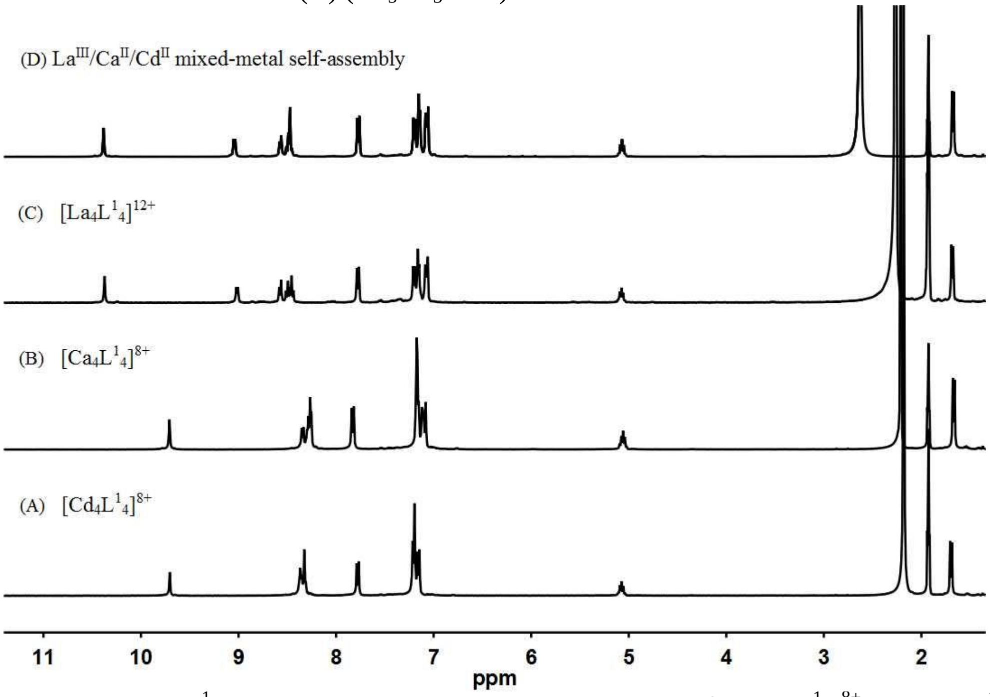

Supplementary Figure 195. ^{1}H NMR spectra (400 MHz, CD_3CN, 298K) for $[Cd_4L^1{}_4]^{8+}$ (A), $[Ca_4L^1{}_4]^{8+}$ (B), $[La_4L^1{}_4]^{12+}$ (C) and $Cd^{II}/Ca^{II}/La^{III}$ mixed-metal self-assembled complexes with L^1 (ClO_4^-salt) (D).

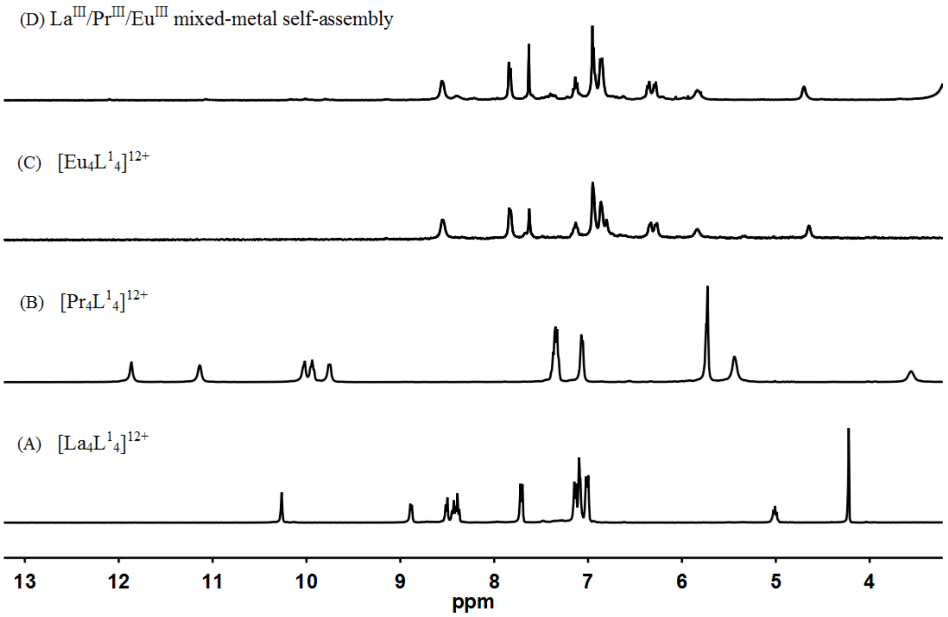

Supplementary Figure 196. ^{1}H NMR spectra (400 MHz, CD_3CN, 298K) for $[La_4L^1_4]^{12+}$ (A), $[Pr_4L^1_4]^{12+}$ (B), $[Eu_4L^1_4]^{12+}$ (C) and La^{III}/Pr^{III}/Eu^{III} mixed-metal self-assembled complexes with L^1 (ClO_4^- salt) (D).

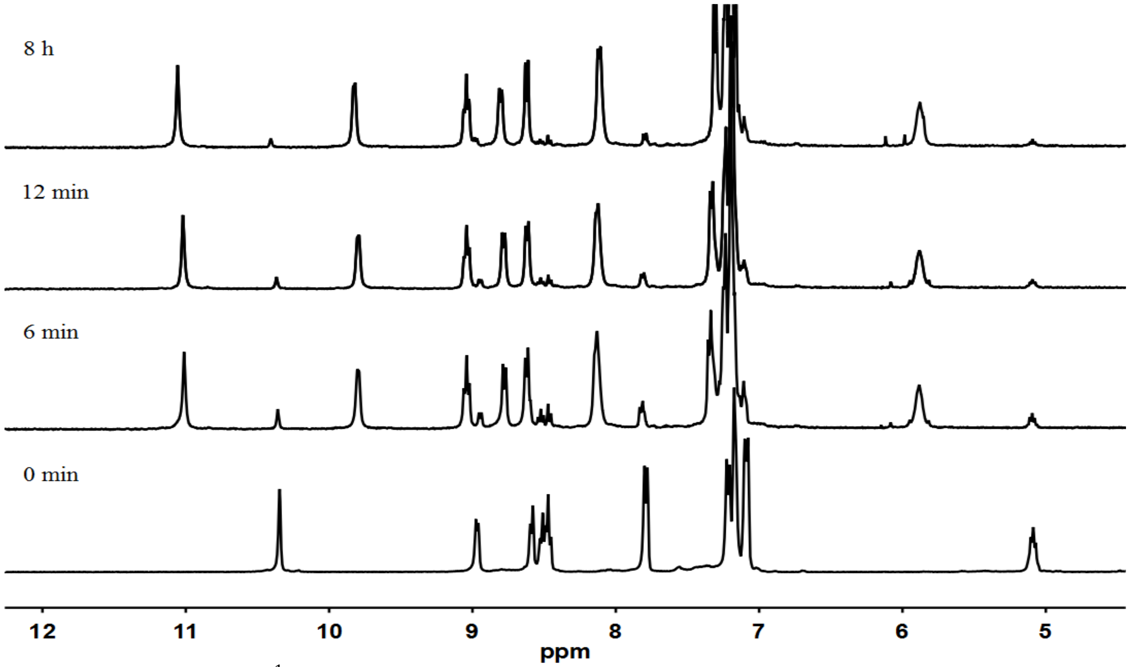

Supplementary Figure 197. ^{1}H NMR spectra (CD_3CN, 400 MHz, 298 K) for the post-synthetic metal-ion metathesis experiments of Ce^{III} toward $[La_4L^1_4]^{12+}$ (ClO_4^- salt).

The post-synthetic metal-ion metathesis experiments of Ce^{III} toward $[La_4L^1_4]^{12+}$ was performed in the similar method as above and the reaction system reached equilibrium in just 12 min.

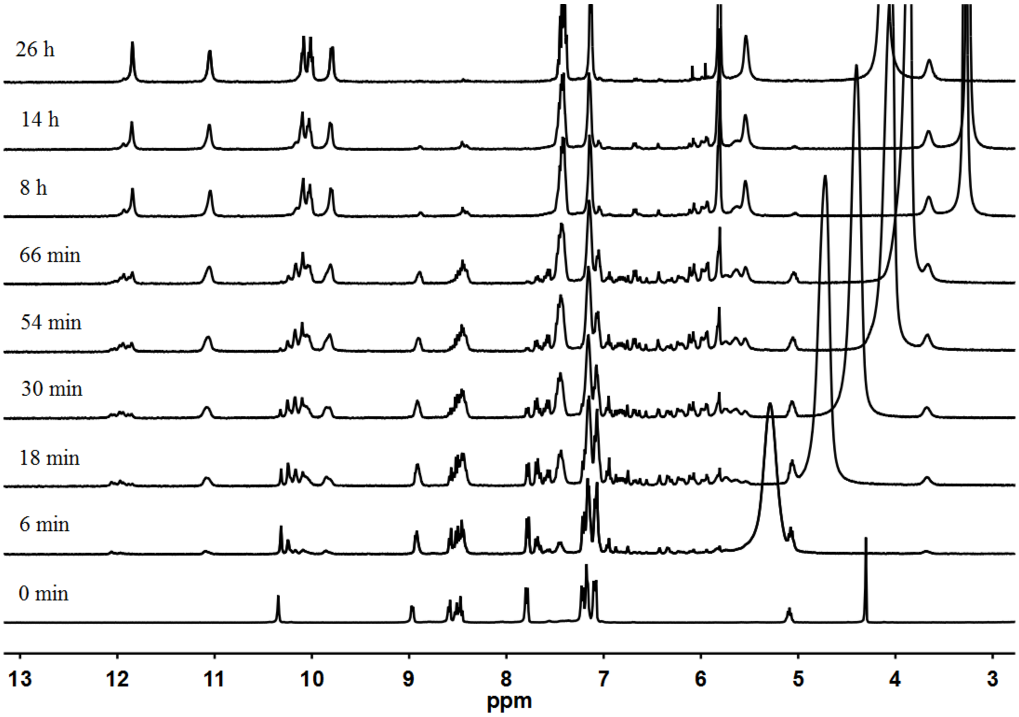

Supplementary Figure 198. ^{1}H NMR spectra (CD_3CN, 400 MHz, 298 K) for the post-synthetic metal-ion metathesis experiments of Pr^{III} toward $[La_4L^1{}_4]^{12+}$ (ClO_4^- salt).

After addition of $Pr(ClO_4)_3$•$6H_2O$ (6.0 μmol) to a solution of preformed $La_4L^1{}_4$ (1.5 μmol) in CD_3CN, with the total volume of CD_3CN as 0.6mL, the substitution process was monitored by ^{1}H NMR spectroscopy at room temperature, and progressively substitution of La(III) by Pr(III) was clearly observed, as new ^{1}H NMR signals corresponding to Pr(III) coordination environment appeared. During the time of 6min to 14h, the highly complicated ^{1}H NMR spectra suggested a mixture of $[(La_nPr_{4-n})L^1{}_4]^{12+}$ (n = 1 to 4) and at the equilibrium state, exactly the same ^{1}H NMR spectrum was observed as that seen in the one-pot self-assembly process. It is worth mentioning that the substitution rate for each metal combination depends on the difference in the ionic radii, and a larger difference results in faster substitution and vice versa. For example, it takes only 30 minutes for Y(III) to fully substitute all four La(III) vertices in the $La_4L^1{}_4$ cage, whereas it takes more than 20 hours for substitution with Pr(III).

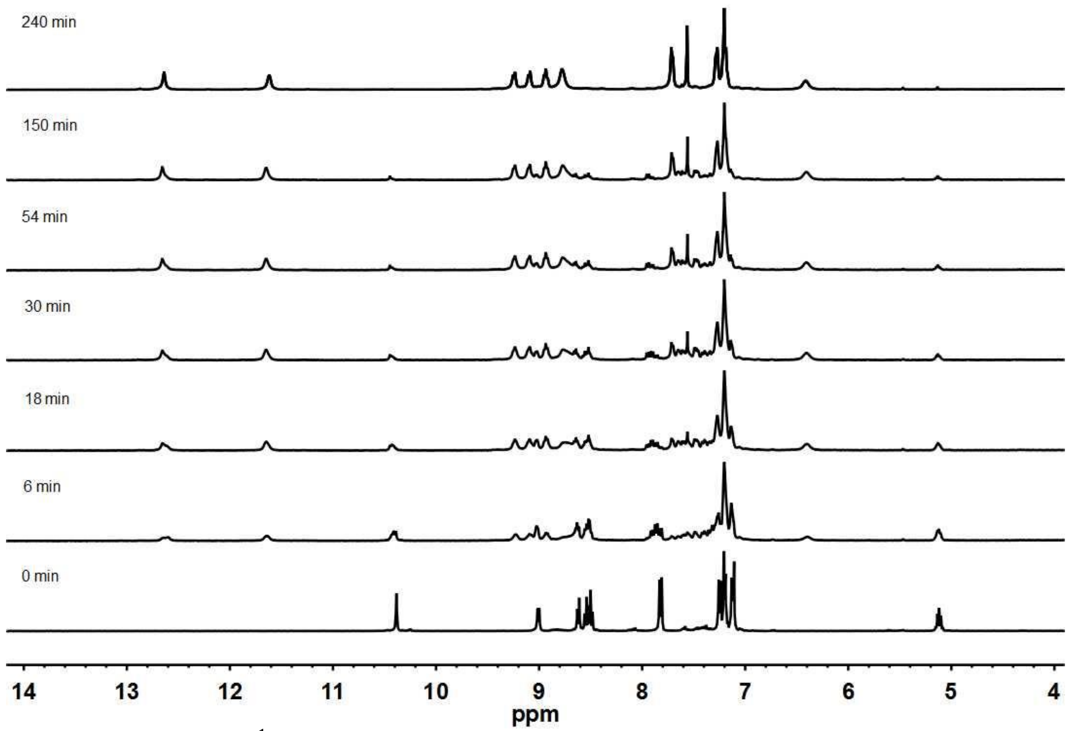

Supplementary Figure 199. ^{1}H NMR spectra (CD_3CN, 400 MHz, 298 K) for the post-synthetic metal-ion metathesis experiments of Nd^{III} toward $[La_4L^1{}_4]^{12+}$ (ClO_4^- salt).

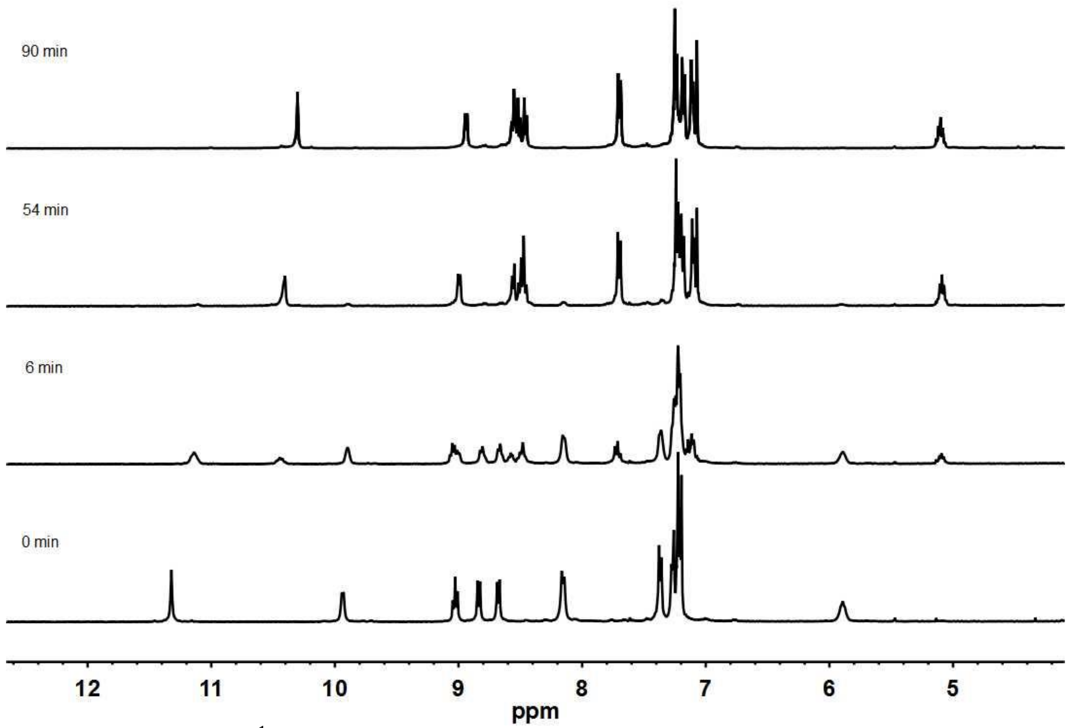

Supplementary Figure 200. ^{1}H NMR spectra (CD_3CN, 400 MHz, 298 K) for the post-synthetic metal-ion metathesis experiments of Sm^{III} toward $[Ce_4L^1{}_4]^{12+}$ (ClO_4^- salt).

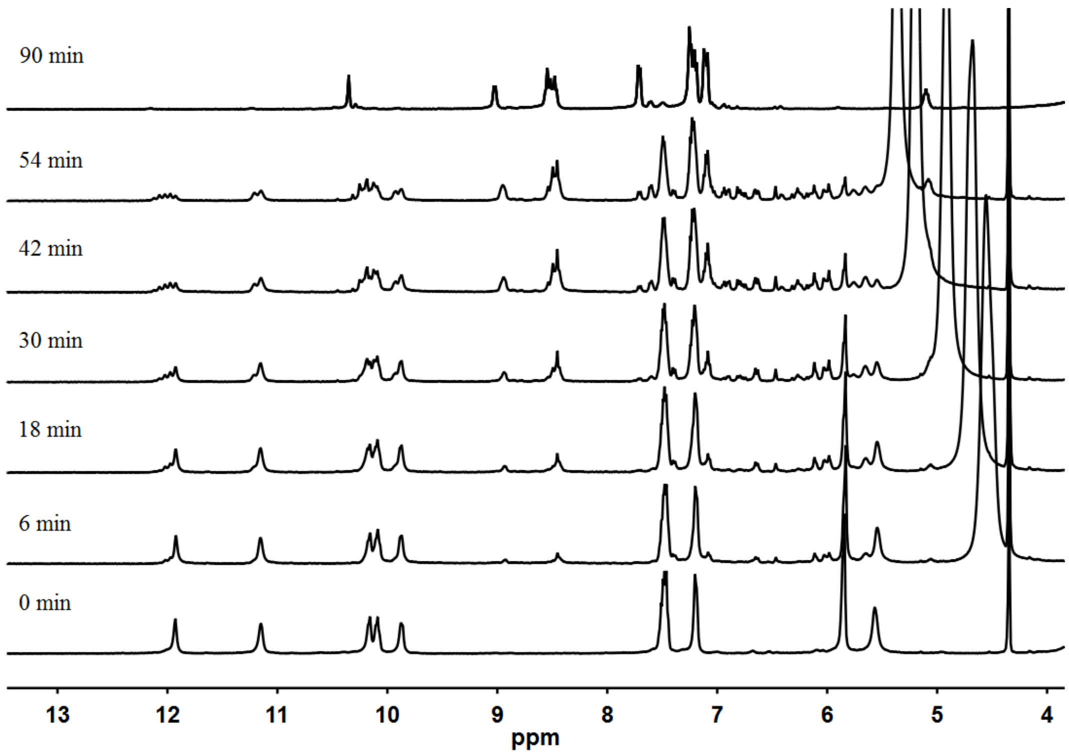

Supplementary Figure 201. ^{1}H NMR spectra (CD_3CN, 400 MHz, 298 K) for the post-synthetic metal-ion metathesis experiments of Sm^{III} toward $[Pr_4L^1{}_4]^{12+}$ (ClO_4^- counter-anion).

After addition of $Sm(ClO_4)_3$•$6H_2O$ (6.0 μmol) to a solution of preformed $Pr_4L^1{}_4$ (1.5 μmol) in CD_3CN at room temperature, progressively substitution of Pr(III) by Sm(III) was clearly observed according to the ^{1}H NMR spectroscopy. During the substitution process, a mixture of $[(Pr_nSm_{4-n})L^1{}_4]^{12+}$ (n = 1 to 4) was speculated to come into formation and at the final equilibrium state (in 90 min), exactly the same ^{1}H NMR spectrum was observed as that seen in the one-pot self-assembly process.

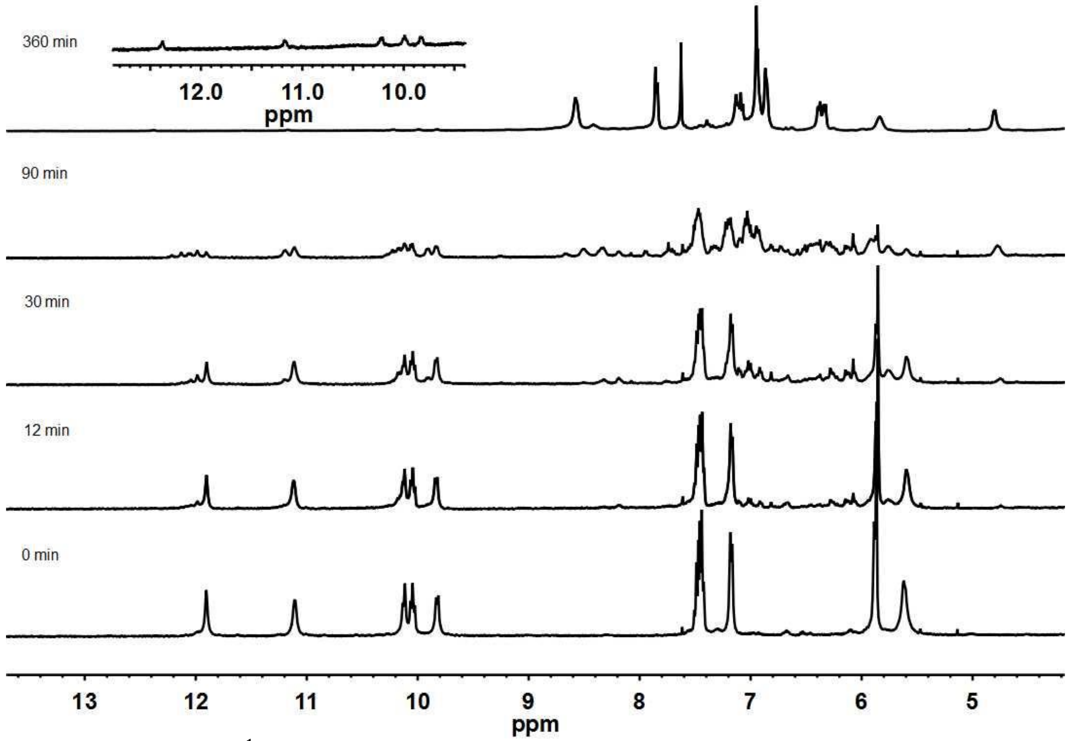

Supplementary Figure 202. ^{1}H NMR spectra (CD_3CN, 400 MHz, 298 K) for the post-synthetic metal-ion metathesis experiments of Eu^{III} toward $[Pr_4L^1{}_4]^{12+}$ (ClO_4^- counter-anion).

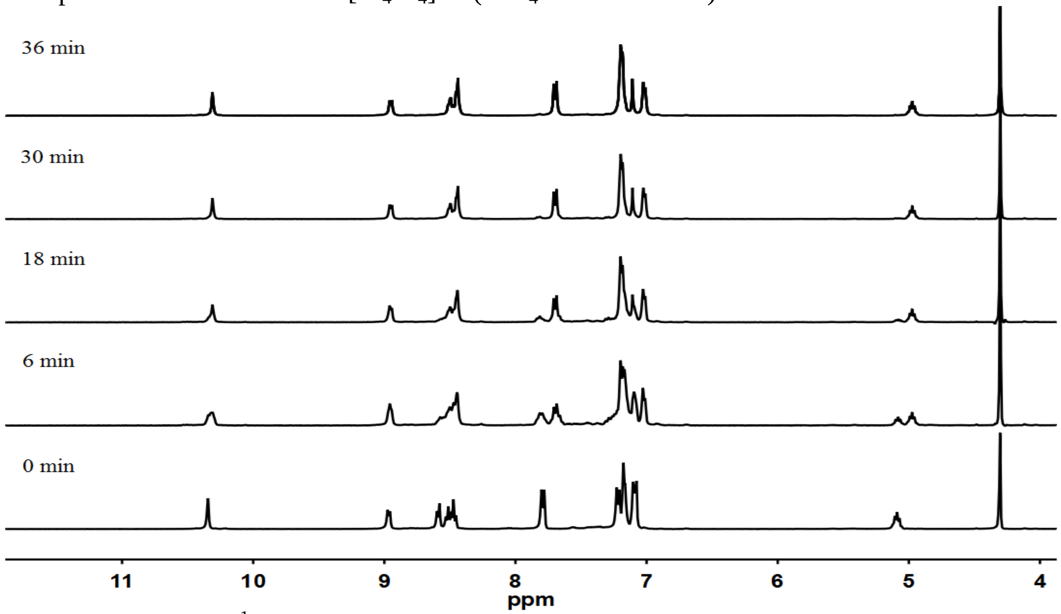

Supplementary Figure 203. ^{1}H NMR spectra (CD_3CN, 400 MHz, 298 K) for the post-synthetic metal-ion metathesis experiments of Y^{III} toward $[La_4L^1{}_4]^{12+}$ (ClO_4^-salt).

After addition of $Y(ClO_4)_3$•$6H_2O$ (6.0 μmol) to a solution of preformed $La_4L^1{}_4$ (1.5 μmol) in CD_3CN at room temperature, substitution of Pr(III) by Sm(III) was observed according to the ^{1}H NMR spectroscopy. The substitution process reached equilibrium in 30 min, resulting in exactly the same ^{1}H NMR spectrum was observed as that seen in the one-pot mix-metal self-assembly process.

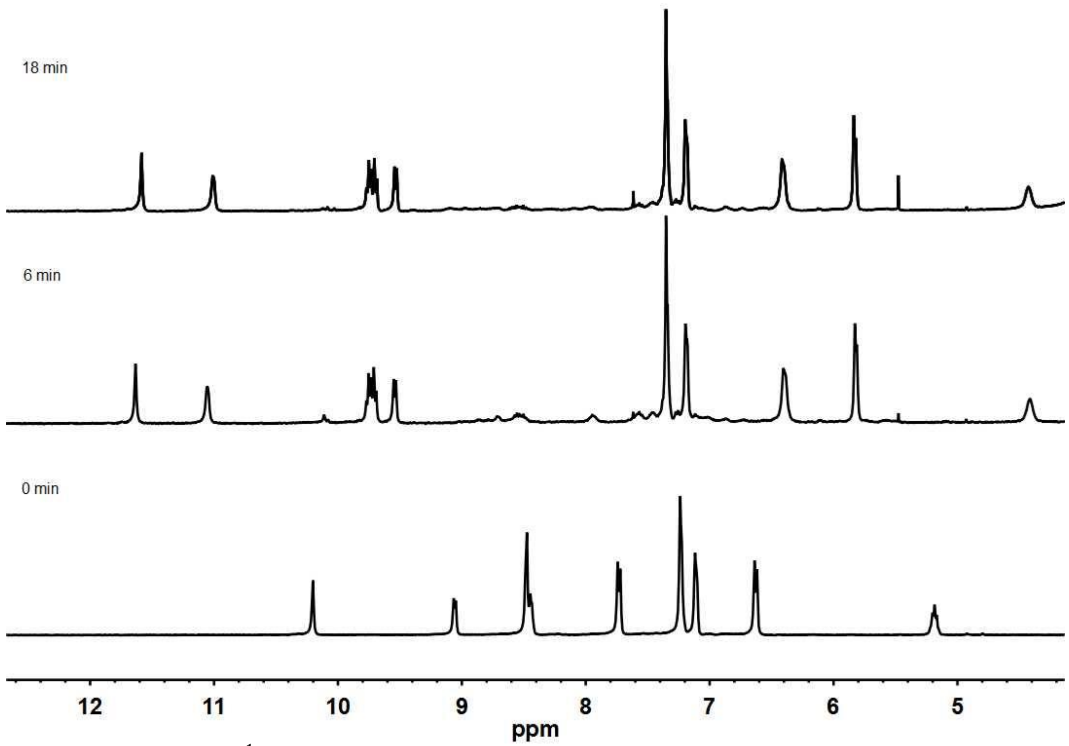

Supplementary Figure 204. ^{1}H NMR spectra (CD_3CN, 400 MHz, 298 K) for the post-synthetic metal-ion metathesis experiments of Pr^{III} toward $[La_2L^3_3]^{6+}$ (ClO_4^- salt).

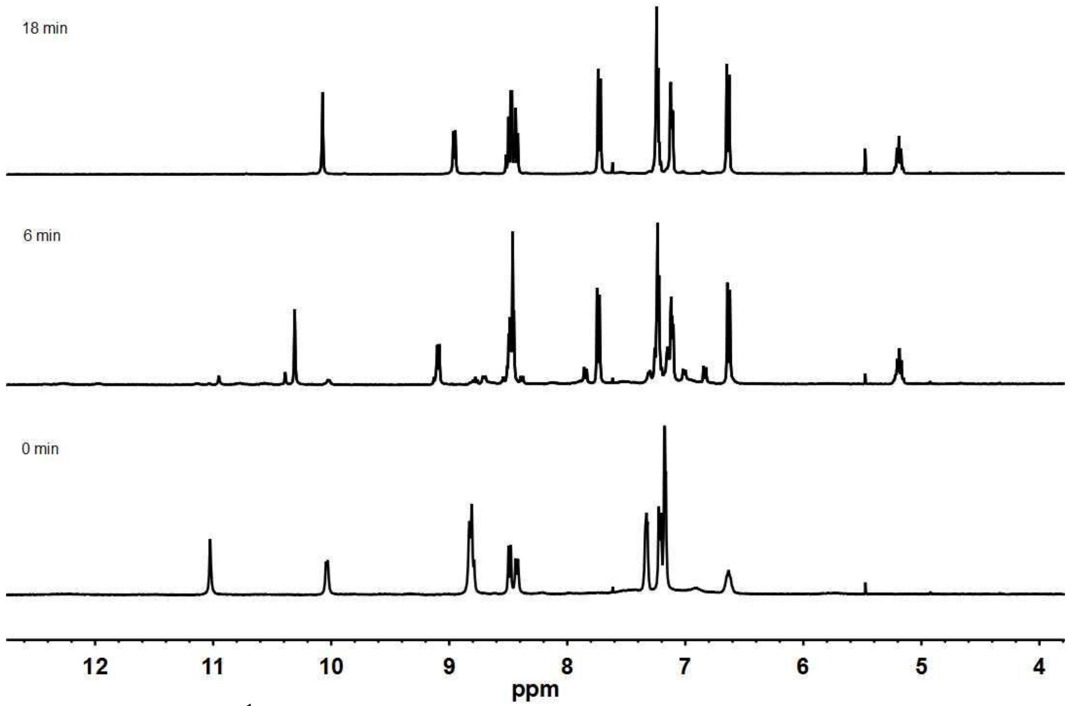

Supplementary Figure 205. ^{1}H NMR spectra (CD_3CN, 400 MHz, 298 K) for the post-synthetic metal-ion metathesis experiments of Sm^{III} toward $[Ce_2L^3_3]^{6+}$ (ClO_4^- salt).

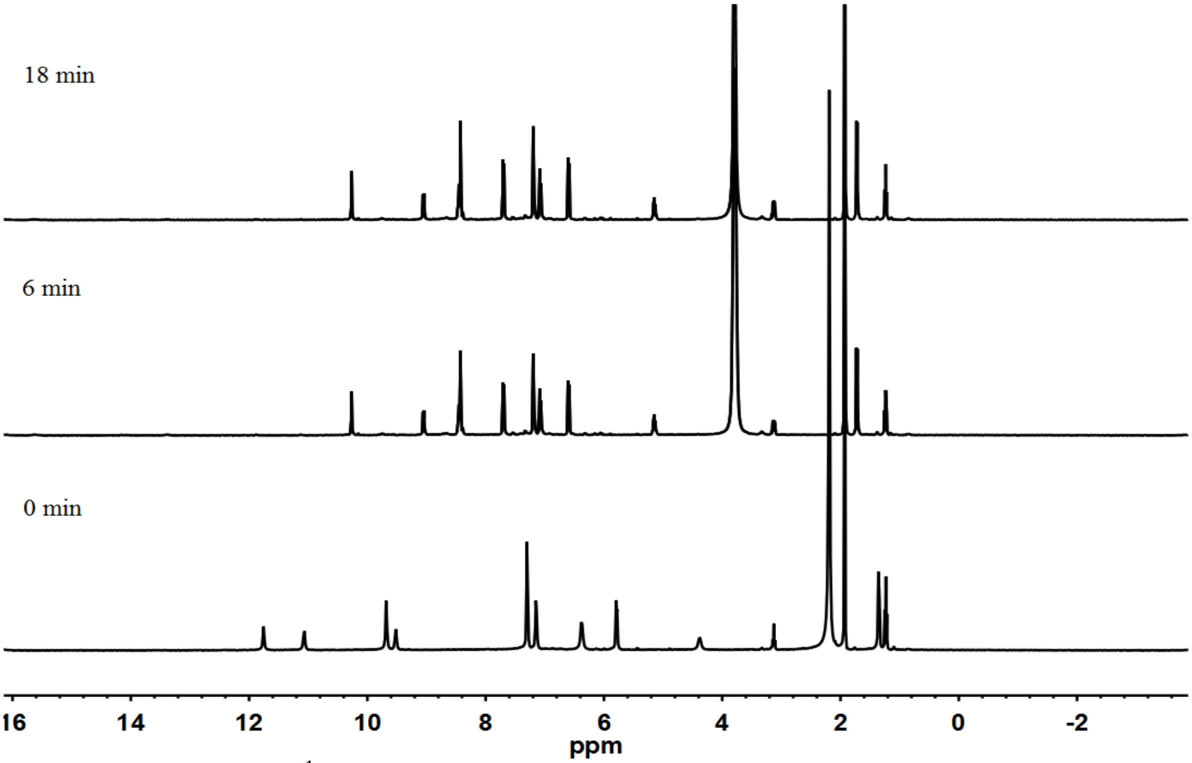

Supplementary Figure 206. ^{1}H NMR spectra (CD_3CN, 400 MHz, 298 K) for the post-synthetic metal-ion metathesis experiments of Sm^{III} toward $[Pr_2L^3_3]^{6+}$ (ClO_4^- salt).

The post-synthetic metal-ion metathesis experiments of Sm^{III} toward $[Pr_2L^3_3]^{6+}$ was performed in the similar method as above and the reaction system reached equilibrium in just 6 min.

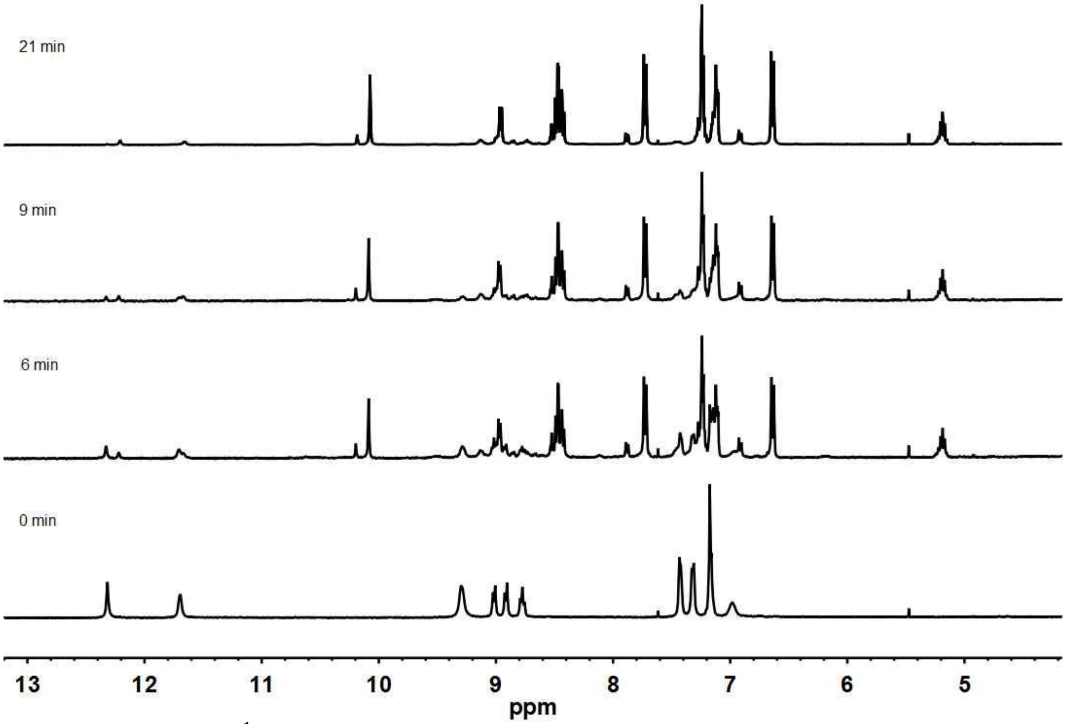

Supplementary Figure 207. ^{1}H NMR spectra (CD_3CN, 400 MHz, 298 K) for the post-synthetic metal-ion metathesis experiments of Sm^{III} toward $[Nd_2L^3_3]^{6+}$ (ClO_4^- salt).

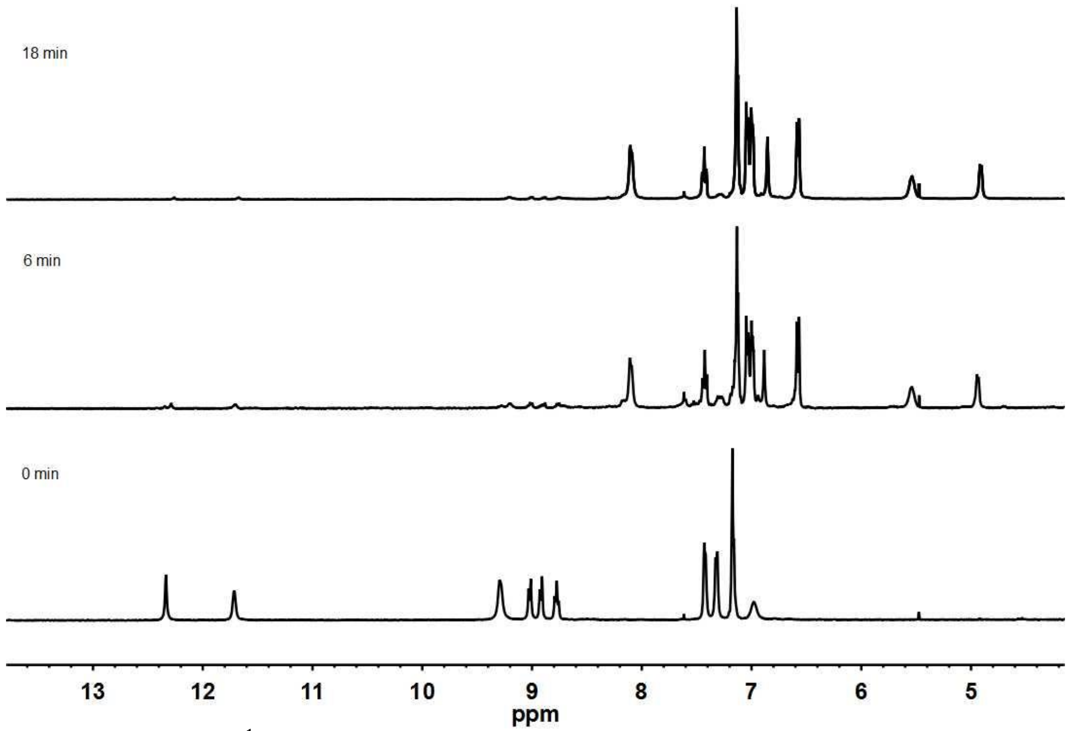

Supplementary Figure 208. ^{1}H NMR spectra (CD_3CN, 400 MHz, 298 K) for the post-synthetic metal-ion metathesis experiments of Eu^{III} toward $[Nd_2L^3_3]^{6+}$ (ClO_4^- salt).

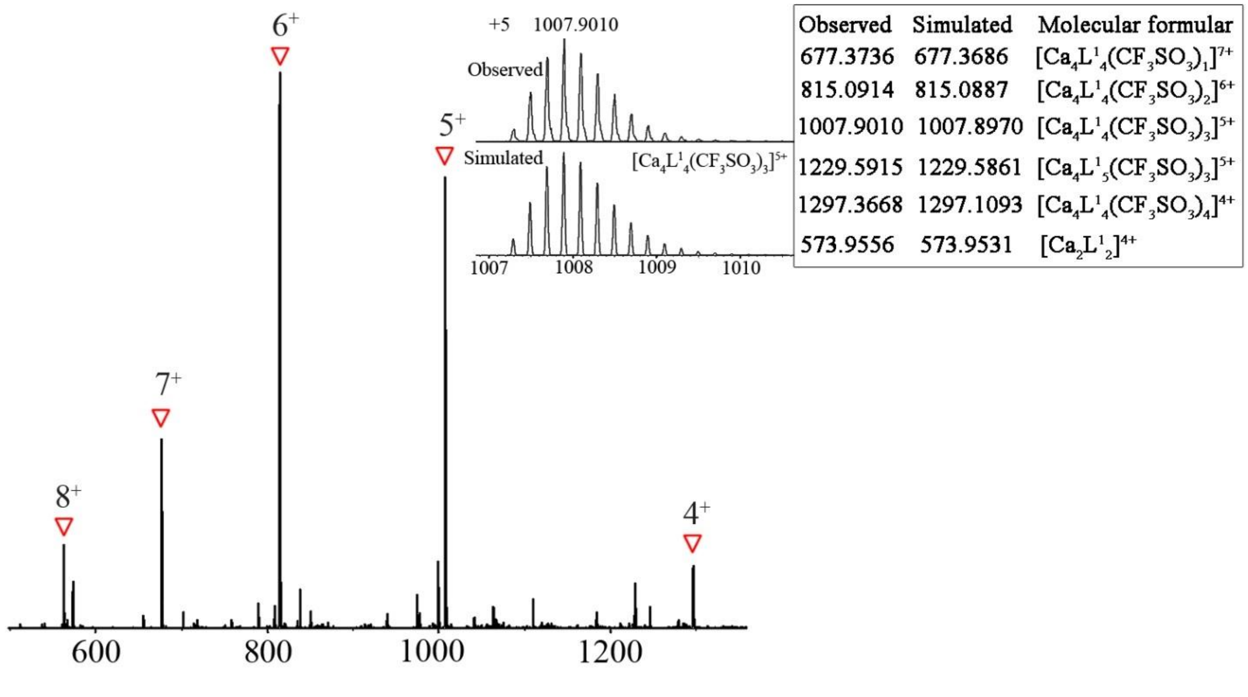

Supplementary Figure 209. ESI-TOF-MS spectrum for $\mathbf{[Ca_4L^{1S}_4](CF_3SO_3)_8}$ with insets showing the observed and simulated isotope patterns of the +5 peaks.

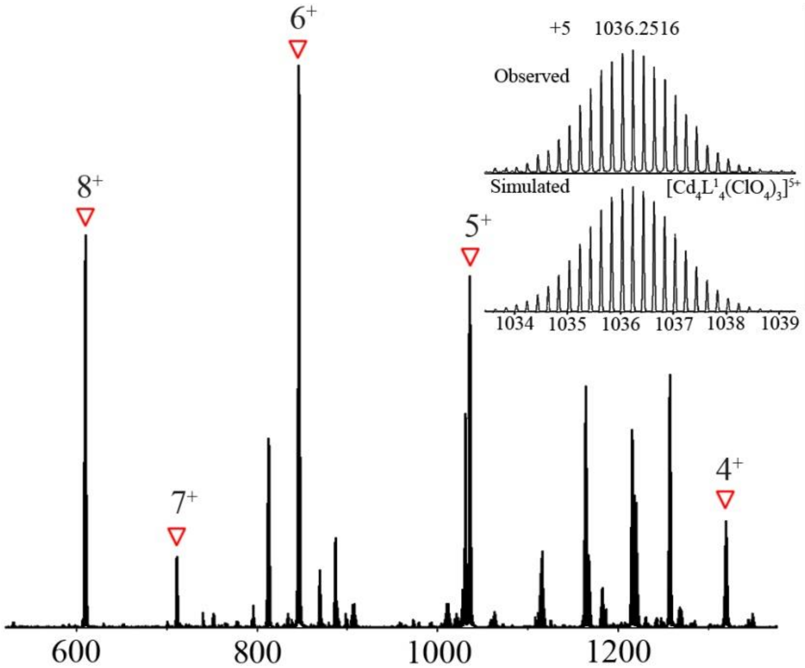

Observed	Simulated	Molecular formular
610.6775	610.2990	$[Cd_4L^1_4]^{8+}$
711.6234	711.7629	$[Cd_4L^1_4(ClO_4)_1]^{7+}$
870.1173	870.1123	$[Cd_4L^1_5(ClO_4)_1]^{7+}$
846.8852	846.8814	$[Cd_4L^1_4(ClO_4)_2]^{6+}$
1031.6258	1031.6224	$[Cd_4L^1_5(ClO_4)_2]^{6+}$
1036.2516	1036.2475	$[Cd_4L^1_4(ClO_4)_3]^{5+}$
1320.3012	1320.0464	$[Cd_4L^1_4(ClO_4)_4]^{4+}$

Supplementary Figure 210. ESI-TOF-MS spectrum for **$[Cd_4L^{1S}_4](ClO_4)_8$** with insets showing the observed and simulated isotope patterns of the +5 peaks.

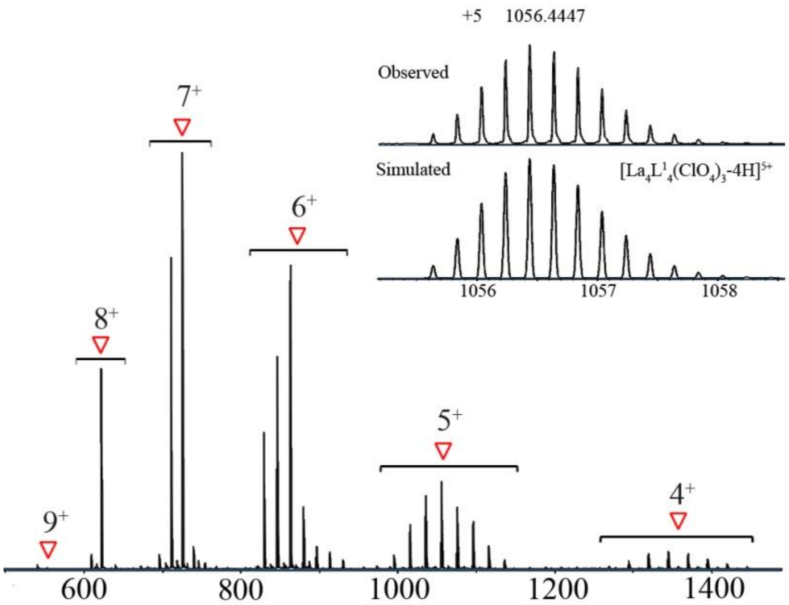

Observed	Simulated	Molecular formular
553.9323	553.9310	$[La_4L^1_4\text{-}3H]^{9+}$
623.0479	623.0465	$[La_4L^1_4\text{-}4H]^{8+}$
711.9106	711.9092	$[La_4L^1_4\text{-}5H]^{7+}$
726.1896	726.1885	$[La_4L^1_4(ClO_4)_1\text{-}4H]^{7+}$
830.3948	830.3929	$[La_4L^1_4\text{-}6H]^{6+}$
847.0533	847.0520	$[La_4L^1_4(ClO_4)_1\text{-}5H]^{6+}$
863.8795	863.8781	$[La_4L^1_4(ClO_4)_2\text{-}4H]^{6+}$
880.5388	880.5373	$[La_4L^1_4(ClO_4)_3\text{-}3H]^{6+}$
1016.2624	1016.2610	$[La_4L^1_4(ClO_4)_1\text{-}6H]^{5+}$
1036.4537	1036.4523	$[La_4L^1_4(ClO_4)_2\text{-}5H]^{5+}$
1056.4447	1056.4434	$[La_4L^1_4(ClO_4)_3\text{-}4H]^{5+}$
1076.6361	1076.6346	$[La_4L^1_4(ClO_4)_4\text{-}3H]^{5+}$
1096.8274	1096.6257	$[La_4L^1_4(ClO_4)_5\text{-}2H]^{5+}$
1320.3030	1320.3024	$[La_4L^1_4(ClO_4)_3\text{-}5H]^{4+}$
1345.5422	1345.5414	$[La_4L^1_4(ClO_4)_4\text{-}4H]^{4+}$
1370.5309	1370.5303	$[La_4L^1_4(ClO_4)_5\text{-}3H]^{4+}$

Supplementary Figure 211. ESI-TOF-MS spectrum for **$[La_4L^{1S}_4](ClO_4)_{12}$** with insets showing the observed and simulated isotope patterns of the +5 peaks.

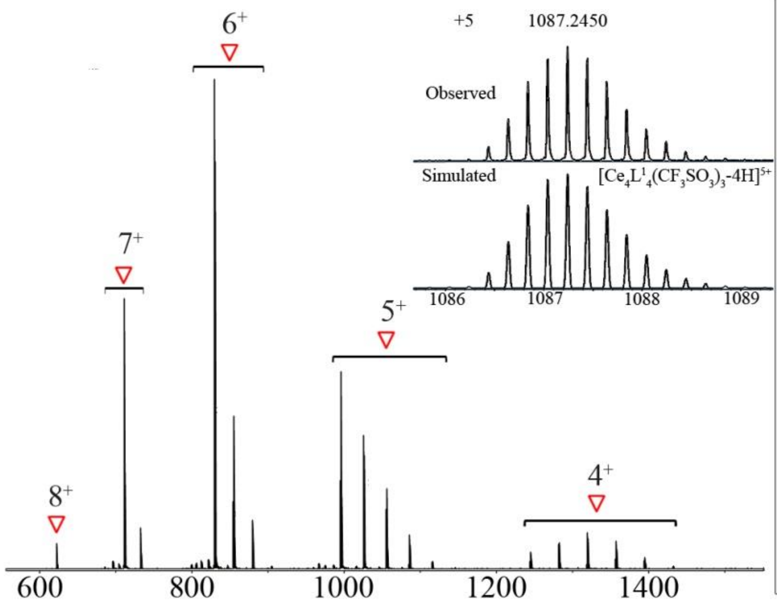

Observed	Simulated	Molecular formular
623.5462	623.5460	$[Ce_4L^1_4-4H]^{8+}$
712.4802	712.4800	$[Ce_4L^1_4-5H]^{7+}$
734.0465	734.0461	$[Ce_4L^1_4(CF_3SO_3)_1-4H]^{7+}$
831.2261	831.0588	$[Ce_4L^1_4-6H]^{6+}$
856.2190	856.2192	$[Ce_4L^1_4(CF_3SO_3)_1-5H]^{6+}$
881.2125	881.2125	$[Ce_4L^1_4(CF_3SO_3)_2-4H]^{6+}$
997.2694	997.0692	$[Ce_4L^1_4-7H]^{5+}$
1027.2611	1207.2616	$[Ce_4L^1_4(CF_3SO_3)_1-6H]^{5+}$
1057.2529	1057.2535	$[Ce_4L^1_4(CF_3SO_3)_2-5H]^{5+}$
1087.2450	1087.2450	$[Ce_4L^1_4(CF_3SO_3)_3-4H]^{5+}$
1246.0836	1246.0846	$[Ce_4L^1_4-8H]^{4+}$
1283.8243	1283.8252	$[Ce_4L^1_4(CF_3SO_3)_1-7H]^{4+}$
1321.3137	1321.3150	$[Ce_4L^1_4(CF_3SO_3)_2-6H]^{4+}$
1358.8039	1358.8049	$[Ce_4L^1_4(CF_3SO_3)_3-5H]^{4+}$
1396.2933	1396.2948	$[Ce_4L^1_4(CF_3SO_3)_4-4H]^{4+}$

Supplementary Figure 212. ESI-TOF-MS spectrum for $\mathbf{[Ce_4L^{1S}{}_4](CF_3SO_3)_{12}}$ with insets showing the observed and simulated isotope patterns of the +5 peaks.

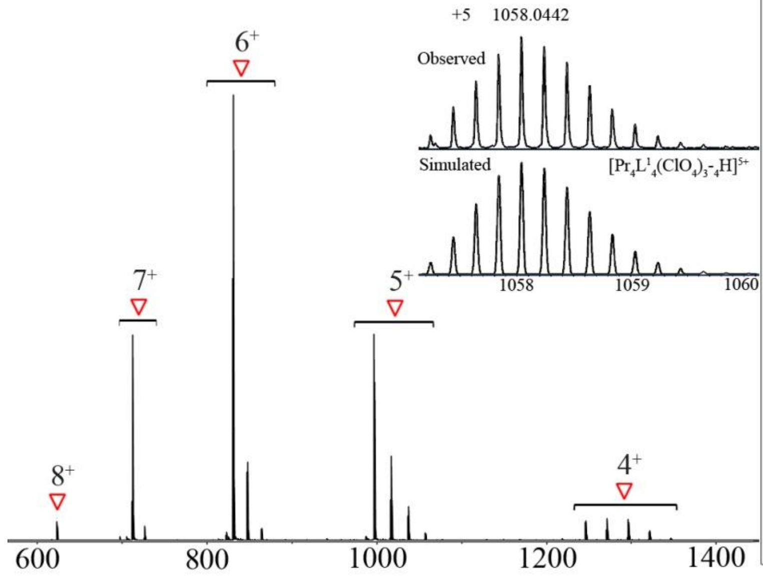

Observed	Simulated	Molecular formular
624.0475	624.0471	$[Pr_4L^1_4-4H]^{8+}$
713.0533	713.0528	$[Pr_4L^1_4-5H]^{7+}$
727.3324	727.3321	$[Pr_4L^1_4(ClO_4)_1-4H]^{7+}$
831.7277	831.7271	$[Pr_4L^1_4-6H]^{6+}$
848.3859	848.3862	$[Pr_4L^1_4(ClO_4)_1-5H]^{6+}$
865.2125	865.2123	$[Pr_4L^1_4(ClO_4)_2-4H]^{6+}$
997.8714	997.8710	$[Pr_4L^1_4-7H]^{5+}$
1017.8618	1017.8620	$[Pr_4L^1_4(ClO_4)_1-6H]^{5+}$
1038.0532	1038.0534	$[Pr_4L^1_4(ClO_4)_2-5H]^{5+}$
1058.0442	1058.0444	$[Pr_4L^1_4(ClO_4)_3-4H]^{5+}$
1247.0863	1247.0870	$[Pr_4L^1_4-8H]^{4+}$
1272.0751	1272.0757	$[Pr_4L^1_4(ClO_4)_1-7H]^{4+}$
1297.3142	1297.3149	$[Pr_4L^1_4(ClO_4)_2-6H]^{4+}$
1322.3031	1322.3037	$[Pr_4L^1_4(ClO_4)_3-5H]^{4+}$
1347.5416	1347.5427	$[Pr_4L^1_4(ClO_4)_4-4H]^{4+}$

Supplementary Figure 213. ESI-TOF-MS spectrum for $\mathbf{[Pr_4L^{1S}{}_4](ClO_4)_{12}}$ with insets showing the observed and simulated isotope patterns of the +5 peaks.

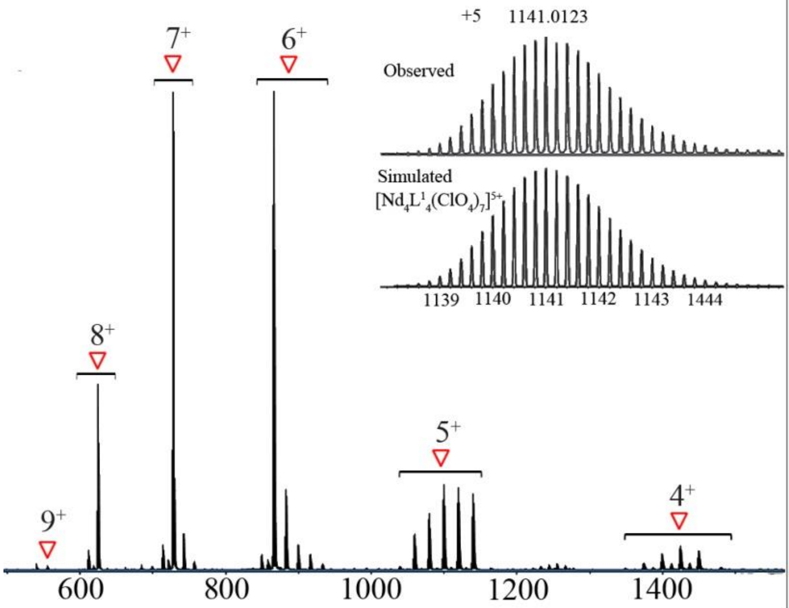

Observed	Simulated	Molecular formular
556.1555	556.1551	$[Nd_4L^1{}_4\text{-}3H]^{9+}$
625.5495	625.5486	$[Nd_4L^1{}_4\text{-}4H]^{8+}$
729.1922	729.1912	$[Nd_4L^1{}_4(ClO_4)_1\text{-}4H]^{7+}$
743.4713	743.4704	$[Nd_4L^1{}_4(ClO_4)_2\text{-}3H]^{7+}$
867.2156	867.2143	$[Nd_4L^1{}_4(ClO_4)_2\text{-}4H]^{6+}$
883.8743	884.0404	$[Nd_4L^1{}_4(ClO_4)_3\text{-}3H]^{6+}$
900.7002	900.6995	$[Nd_4L^1{}_4(ClO_4)_4\text{-}2H]^{6+}$
1060.6482	1060.6470	$[Nd_4L^1{}_4(ClO_4)_3\text{-}4H]^{5+}$
1080.8392	1080.6380	$[Nd_4L^1{}_4(ClO_4)_4\text{-}3H]^{5+}$
1101.0303	1100.8293	$[Nd_4L^1{}_4(ClO_4)_5\text{-}2H]^{5+}$
1121.0210	1121.0205	$[Nd_4L^1{}_4(ClO_4)_6\text{-}1H]^{5+}$
1141.0123	1141.0116	$[Nd_4L^1{}_4(ClO_4)_7]^{5+}$
1375.7841	1375.7848	$[Nd_4L^1{}_4(ClO_4)_5\text{-}3H]^{4+}$
1400.7736	1401.0238	$[Nd_4L^1{}_4(ClO_4)_6\text{-}2H]^{4+}$
1426.0121	1426.0126	$[Nd_4L^1{}_4(ClO_4)_7\text{-}1H]^{4+}$
1451.2514	1451.2517	$[Nd_4L^1{}_4(ClO_4)_8]^{4+}$

Supplementary Figure 214. ESI-TOF-MS spectrum for $\mathbf{[Nd_4L^{1S}{}_4](ClO_4)_{12}}$ with insets showing the observed and simulated isotope patterns of the +5 peaks.

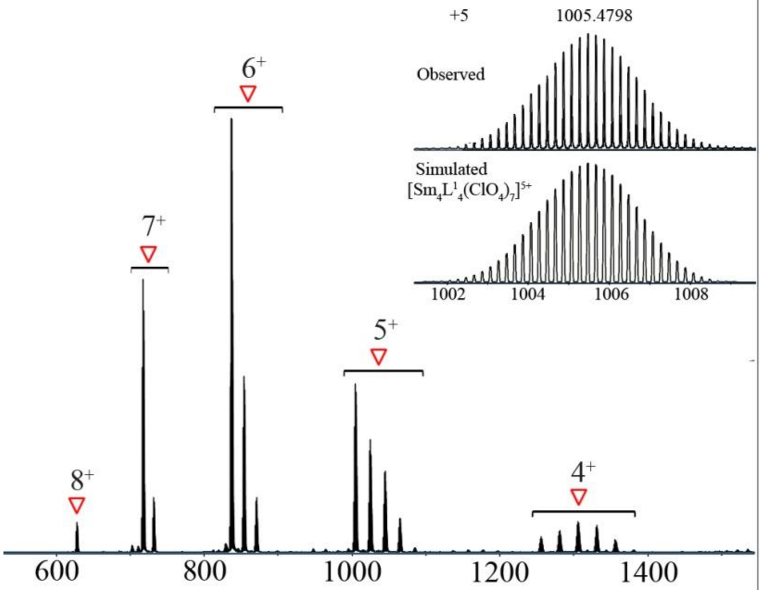

Observed	Simulated	Molecular formular
628.8033	628.8025	$[Sm_4L^1{}_4\text{-}4H]^{8+}$
718.4881	718.4875	$[Sm_4L^1{}_4\text{-}5H]^{7+}$
732.7674	732.9098	$[Sm_4L^1{}_4(ClO_4)_1\text{-}4H]^{7+}$
838.0682	838.0675	$[Sm_4L^1{}_4\text{-}6H]^{6+}$
854.8937	854.8936	$[Sm_4L^1{}_4(ClO_4)_1\text{-}5H]^{6+}$
871.5531	871.5528	$[Sm_4L^1{}_4(ClO_4)_2\text{-}4H]^{6+}$
1005.4798	1005.4796	$[Sm_4L^1{}_4\text{-}7H]^{5+}$
1025.4707	1025.6708	$[Sm_4L^1{}_4(ClO_4)_1\text{-}6H]^{5+}$
1045.6619	1045.6619	$[Sm_4L^1{}_4(ClO_4)_2\text{-}5H]^{5+}$
1065.6530	1065.6529	$[Sm_4L^1{}_4(ClO_4)_3\text{-}4H]^{5+}$
1086.0447	1085.8441	$[Sm_4L^1{}_4(ClO_4)_4\text{-}3H]^{5+}$
1256.5971	1256.5977	$[Sm_4L^1{}_4\text{-}8H]^{4+}$
1282.0862	1281.8367	$[Sm_4L^1{}_4(ClO_4)_1\text{-}7H]^{4+}$
1306.8249	1306.8255	$[Sm_4L^1{}_4(ClO_4)_2\text{-}6H]^{4+}$
1332.3146	1332.0646	$[Sm_4L^1{}_4(ClO_4)_3\text{-}5H]^{4+}$
1356.8021	1357.0534	$[Sm_4L^1{}_4(ClO_4)_4\text{-}4H]^{4+}$

Supplementary Figure 215. ESI-TOF-MS spectrum for $\mathbf{[Sm_4L^{1S}{}_4](ClO_4)_{12}}$ with insets showing the observed and simulated isotope patterns of the +5 peaks.

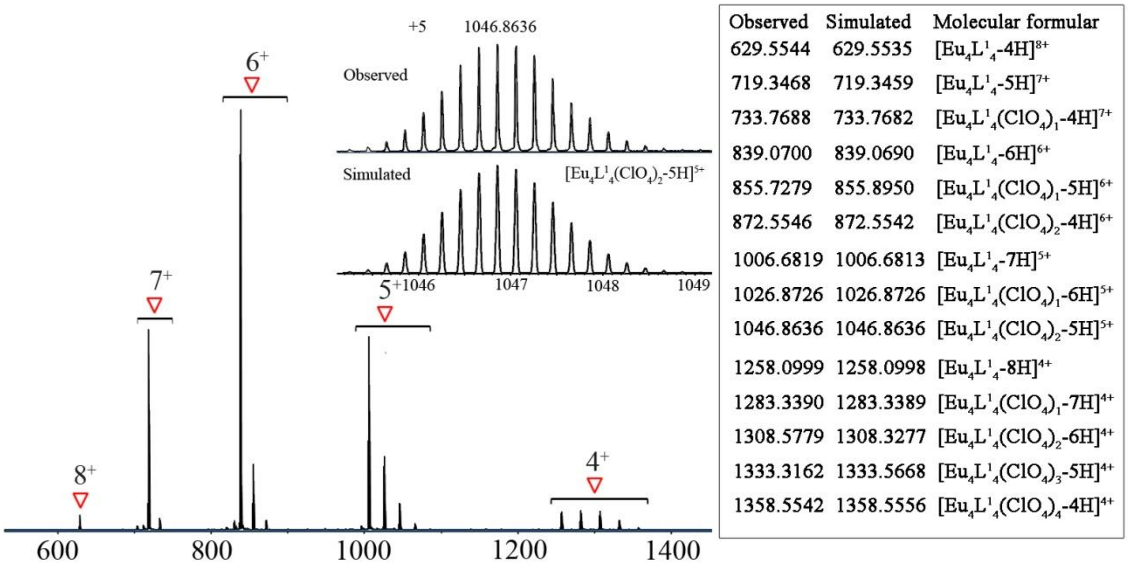

Supplementary Figure 216. ESI-TOF-MS spectrum for **$[Eu_4L^{1S}{}_4](ClO_4)_{12}$** with insets showing the observed and simulated isotope patterns of the +5 peaks.

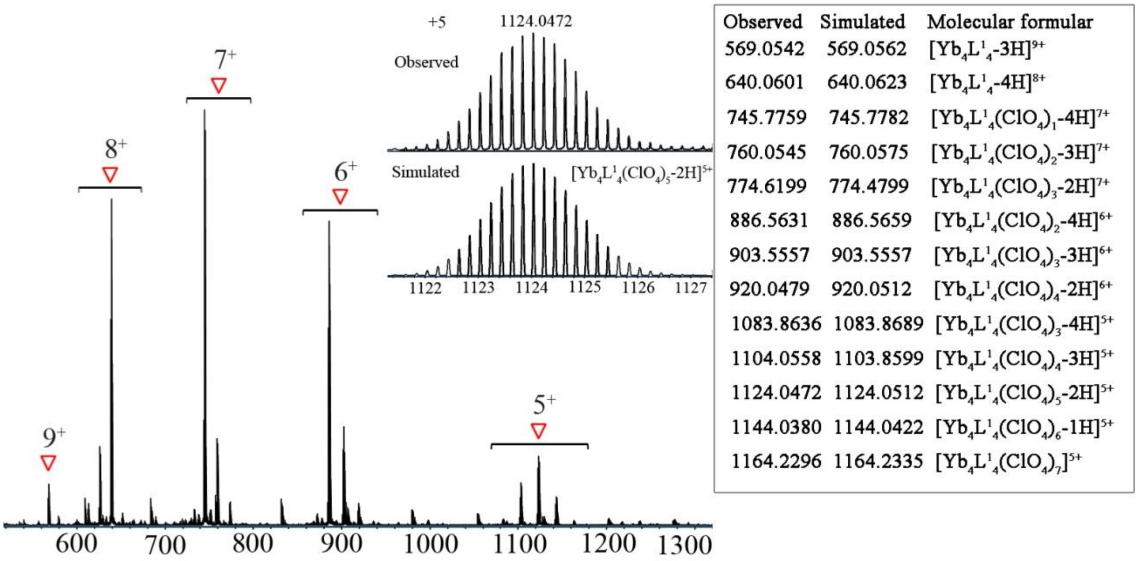

Supplementary Figure 217. ESI-TOF-MS spectrum for **$[Yb_4L^{1S}{}_4](ClO_4)_{12}$** with insets showing the observed and simulated isotope patterns of the +5 peaks.

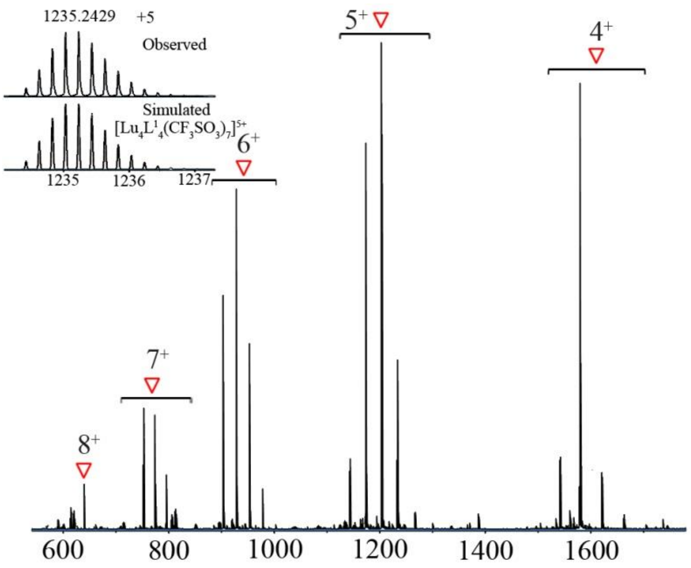

Observed	Simulated	Molecular formular
641.0651	641.0637	$[Lu_4L^1_4\text{-}4H]^{8+}$
753.9250	753.9231	$[Lu_4L^1_4(CF_3SO_3)_1\text{-}4H]^{7+}$
775.3477	775.3459	$[Lu_4L^1_4(CF_3SO_3)_2\text{-}3H]^{7+}$
796.7707	796.7687	$[Lu_4L^1_4(CF_3SO_3)_3\text{-}2H]^{7+}$
904.4046	904.4023	$[Lu_4L^1_4(CF_3SO_3)_2\text{-}4H]^{6+}$
929.3977	929.3956	$[Lu_4L^1_4(CF_3SO_3)_3\text{-}3H]^{6+}$
954.3909	954.3889	$[Lu_4L^1_4(CF_3SO_3)_4\text{-}2H]^{6+}$
979.3838	979.3821	$[Lu_4L^1_4(CF_3SO_3)_5\text{-}1H]^{6+}$
1145.0672	1145.0652	$[Lu_4L^1_4(CF_3SO_3)_4\text{-}3H]^{5+}$
1175.0590	1175.0571	$[Lu_4L^1_4(CF_3SO_3)_5\text{-}2H]^{5+}$
1205.2510	1205.0490	$[Lu_4L^1_4(CF_3SO_3)_6\text{-}1H]^{5+}$
1235.2429	1235.0410	$[Lu_4L^1_4(CF_3SO_3)_7]^{5+}$
1543.8012	1543.5494	$[Lu_4L^1_4(CF_3SO_3)_7\text{-}1H]^{4+}$
1581.2908	1581.2898	$[Lu_4L^1_4(CF_3SO_3)_8]^{4+}$

Supplementary Figure 218. ESI-TOF-MS spectrum for $\mathbf{[Lu_4L^{1S}{}_4](CF_3SO_3)_{12}}$ with insets showing the observed and simulated isotope patterns of the +5 peaks.

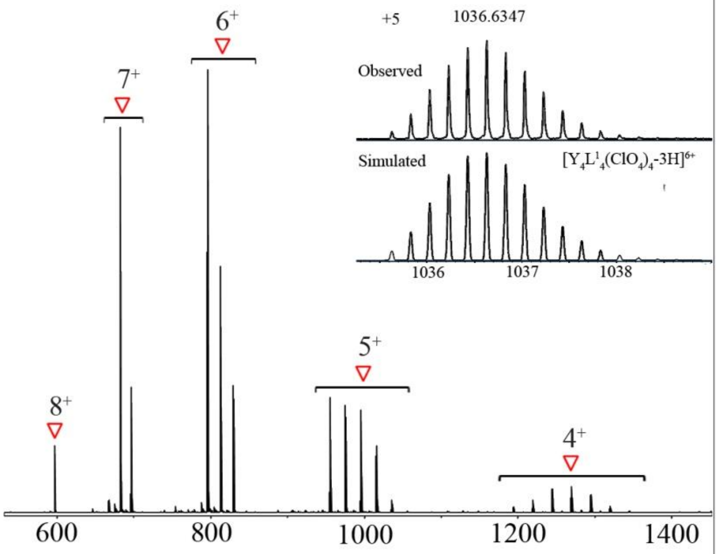

Observed	Simulated	Molecular formular
598.0473	598.0462	$[Y_4L^1_4\text{-}4H]^{8+}$
683.3384	683.3375	$[Y_4L^1_4\text{-}5H]^{7+}$
697.6174	697.6168	$[Y_4L^1_4(ClO_4)_1\text{-}4H]^{7+}$
797.0605	797.0592	$[Y_4L^1_4\text{-}6H]^{6+}$
813.7188	813.7183	$[Y_4L^1_4(ClO_4)_1\text{-}5H]^{6+}$
830.5451	830.5445	$[Y_4L^1_4(ClO_4)_2\text{-}4H]^{6+}$
956.2706	956.2696	$[Y_4L^1_4\text{-}7H]^{5+}$
976.2612	976.2606	$[Y_4L^1_4(ClO_4)_1\text{-}6H]^{5+}$
996.4526	996.4519	$[Y_4L^1_4(ClO_4)_2\text{-}5H]^{5+}$
1016.4435	1016.4430	$[Y_4L^1_4(ClO_4)_3\text{-}4H]^{5+}$
1036.6347	1036.6342	$[Y_4L^1_4(ClO_4)_4\text{-}3H]^{5+}$
1195.0861	1195.0852	$[Y_4L^1_4\text{-}8H]^{4+}$
1220.0742	1220.0739	$[Y_4L^1_4(ClO_4)_1\text{-}7H]^{4+}$
1245.3133	1245.3131	$[Y_4L^1_4(ClO_4)_2\text{-}6H]^{4+}$
1270.3021	1270.3019	$[Y_4L^1_4(ClO_4)_3\text{-}5H]^{4+}$
1295.5411	1295.5409	$[Y_4L^1_4(ClO_4)_4\text{-}4H]^{4+}$

Supplementary Figure 219. ESI-TOF-MS spectrum for $\mathbf{[Y_4L^{1S}{}_4](ClO_4)_{12}}$with insets showing the observed and simulated isotope patterns of the +5 peaks.

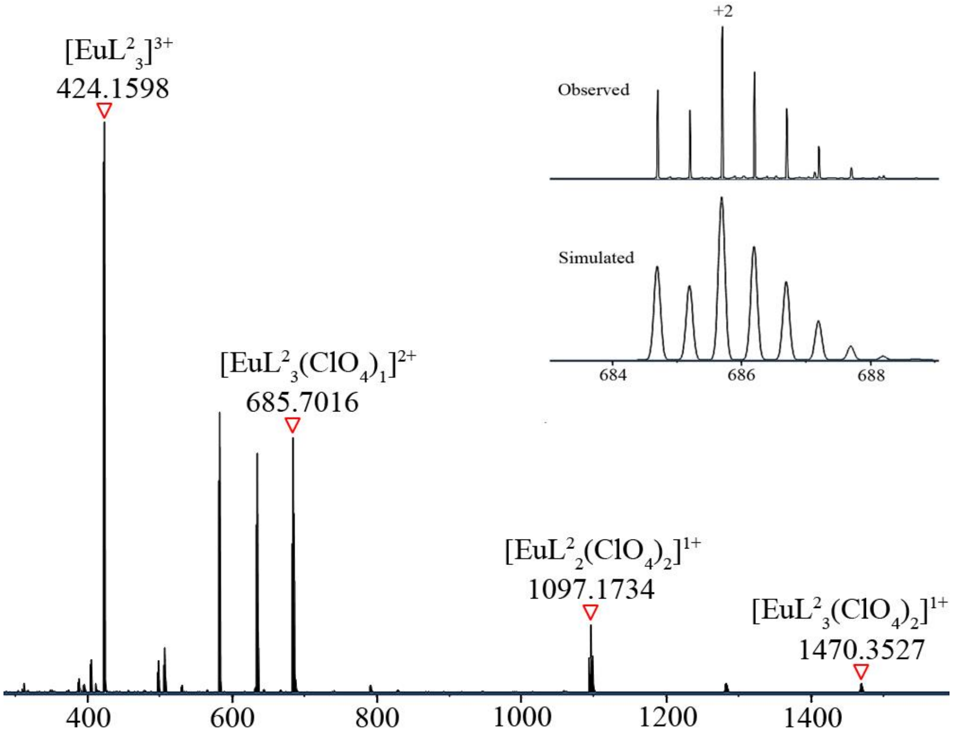

Supplementary Figure 220. ESI-TOF-MS spectrum for **$[Eu_1L^{2S}_3](ClO_4)_3$** with insets showing the observed and simulated isotope patterns of the +2 peaks.

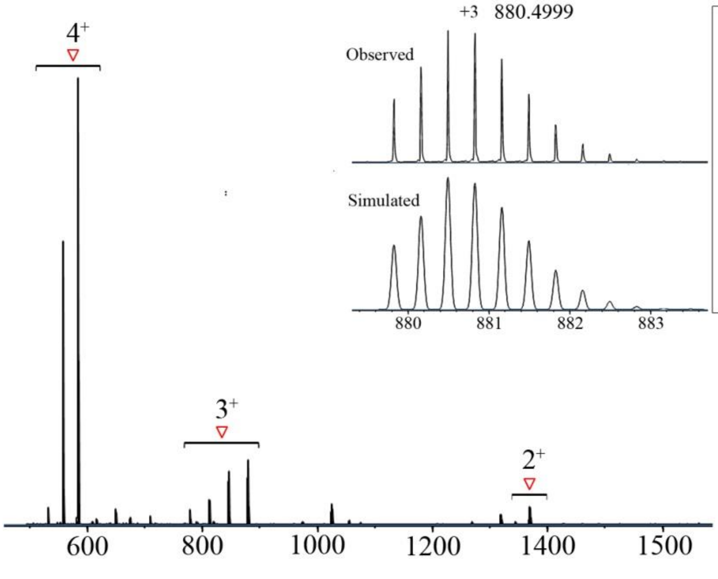

Observed	Simulated	Molecular formular
585.4098	585.4093	$[La_2L^3_3\text{-}2H]^{4+}$
780.2104	780.2100	$[La_2L^3_3\text{-}3H]^{3+}$
813.5294	813.5288	$[La_2L^3_3(ClO_4)_1\text{-}2H]^{3+}$
847.1815	847.1808	$[La_2L^3_3(ClO_4)_2\text{-}H]^{3+}$
880.4999	880.4993	$[La_2L^3_3(ClO_4)_3]^{3+}$
1270.2682	1270.2676	$[La_2L^3_3(ClO_4)_2\text{-}2H]^{2+}$
1320.2464	1320.2453	$[La_2L^3_3(ClO_4)_3\text{-}H]^{2+}$
1370.7248	1370.7237	$[La_2L^3_3(ClO_4)_4]^{2+}$

Supplementary Figure 221. ESI-TOF-MS spectrum for **$[La_2L^{3S}_3](ClO_4)_6$** with insets showing the observed and simulated isotope patterns of the +3 peaks.

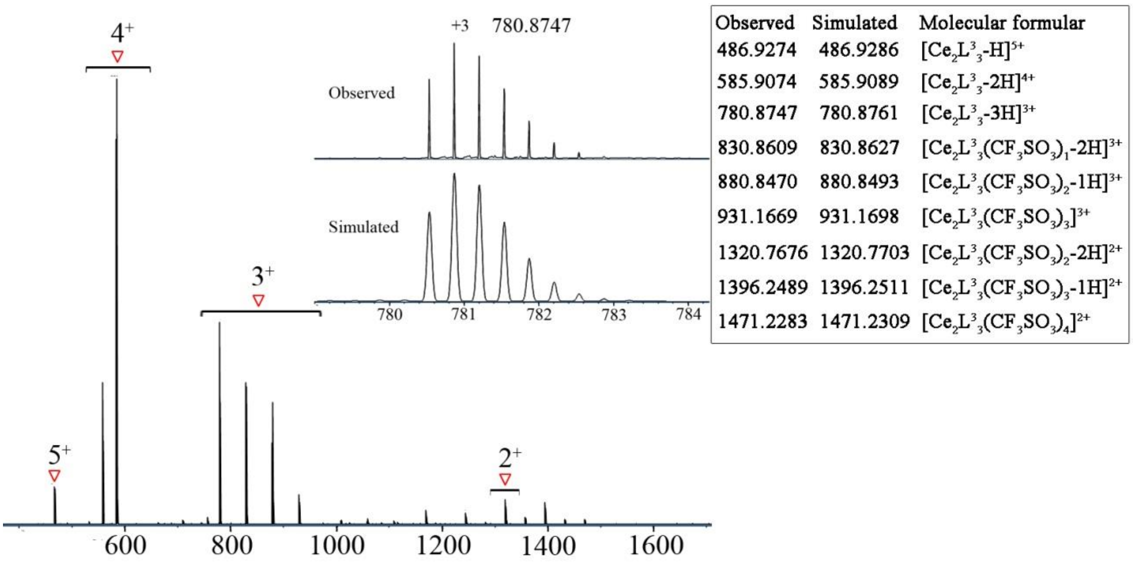

Observed	Simulated	Molecular formular
486.9274	486.9286	$[Ce_2L^3_3\text{-H}]^{5+}$
585.9074	585.9089	$[Ce_2L^3_3\text{-2H}]^{4+}$
780.8747	780.8761	$[Ce_2L^3_3\text{-3H}]^{3+}$
830.8609	830.8627	$[Ce_2L^3_3(CF_3SO_3)_1\text{-2H}]^{3+}$
880.8470	880.8493	$[Ce_2L^3_3(CF_3SO_3)_2\text{-1H}]^{3+}$
931.1669	931.1698	$[Ce_2L^3_3(CF_3SO_3)_3]^{3+}$
1320.7676	1320.7703	$[Ce_2L^3_3(CF_3SO_3)_2\text{-2H}]^{2+}$
1396.2489	1396.2511	$[Ce_2L^3_3(CF_3SO_3)_3\text{-1H}]^{2+}$
1471.2283	1471.2309	$[Ce_2L^3_3(CF_3SO_3)_4]^{2+}$

Supplementary Figure 222. ESI-TOF-MS spectrum for $\mathbf{[Ce_2L^{3S}_3](CF_3SO_3)_6}$ with insets showing the observed and simulated isotope patterns of the +3 peaks.

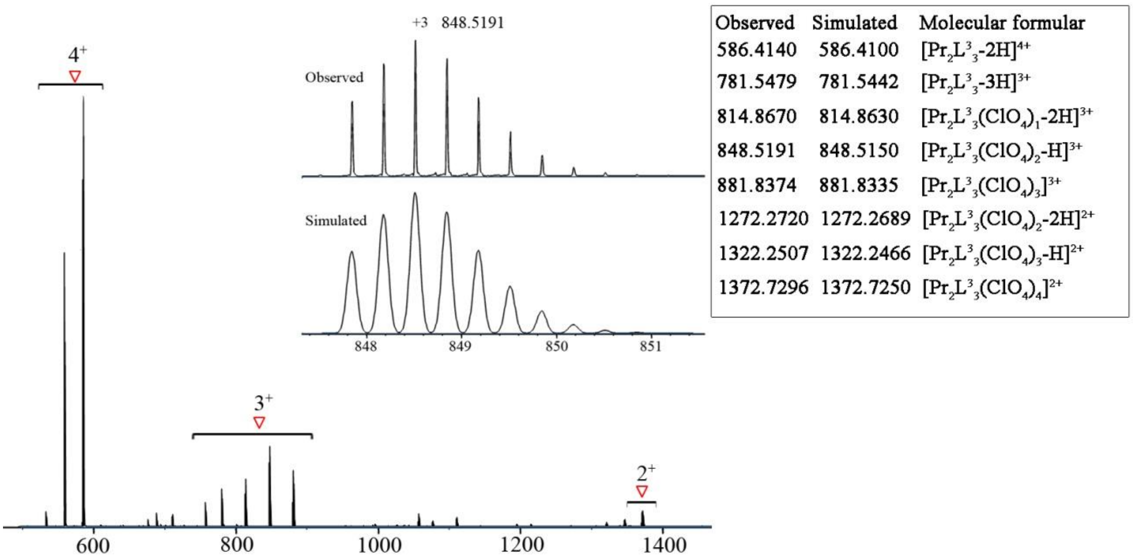

Observed	Simulated	Molecular formular
586.4140	586.4100	$[Pr_2L^3_3\text{-2H}]^{4+}$
781.5479	781.5442	$[Pr_2L^3_3\text{-3H}]^{3+}$
814.8670	814.8630	$[Pr_2L^3_3(ClO_4)_1\text{-2H}]^{3+}$
848.5191	848.5150	$[Pr_2L^3_3(ClO_4)_2\text{-H}]^{3+}$
881.8374	881.8335	$[Pr_2L^3_3(ClO_4)_3]^{3+}$
1272.2720	1272.2689	$[Pr_2L^3_3(ClO_4)_2\text{-2H}]^{2+}$
1322.2507	1322.2466	$[Pr_2L^3_3(ClO_4)_3\text{-H}]^{2+}$
1372.7296	1372.7250	$[Pr_2L^3_3(ClO_4)_4]^{2+}$

Supplementary Figure 223. ESI-TOF-MS spectrum for $\mathbf{[Pr_2L^{3S}_3](ClO_4)_6}$ with insets showing the observed and simulated isotope patterns of the +3 peaks.

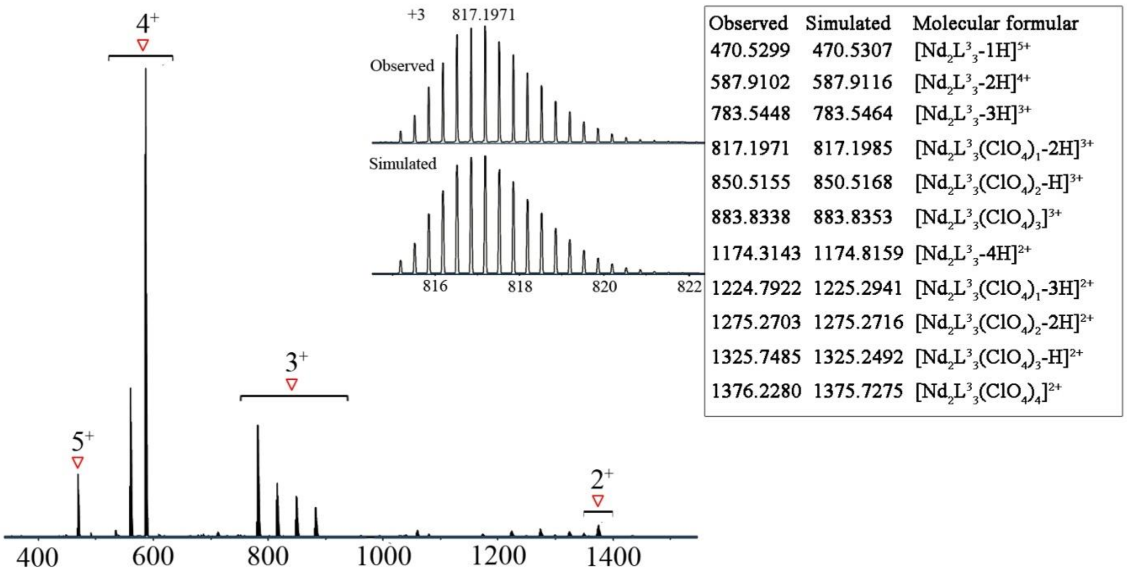

Observed	Simulated	Molecular formular
470.5299	470.5307	$[Nd_2L^3_3\text{-}1H]^{5+}$
587.9102	587.9116	$[Nd_2L^3_3\text{-}2H]^{4+}$
783.5448	783.5464	$[Nd_2L^3_3\text{-}3H]^{3+}$
817.1971	817.1985	$[Nd_2L^3_3(ClO_4)_1\text{-}2H]^{3+}$
850.5155	850.5168	$[Nd_2L^3_3(ClO_4)_2\text{-}H]^{3+}$
883.8338	883.8353	$[Nd_2L^3_3(ClO_4)_3]^{3+}$
1174.3143	1174.8159	$[Nd_2L^3_3\text{-}4H]^{2+}$
1224.7922	1225.2941	$[Nd_2L^3_3(ClO_4)_1\text{-}3H]^{2+}$
1275.2703	1275.2716	$[Nd_2L^3_3(ClO_4)_2\text{-}2H]^{2+}$
1325.7485	1325.2492	$[Nd_2L^3_3(ClO_4)_3\text{-}H]^{2+}$
1376.2280	1375.7275	$[Nd_2L^3_3(ClO_4)_4]^{2+}$

Supplementary Figure 224. ESI-TOF-MS spectrum for **$[Nd_2L^{3S}{}_3](ClO_4)_6$** with insets showing the observed and simulated isotope patterns of the +3 peaks.

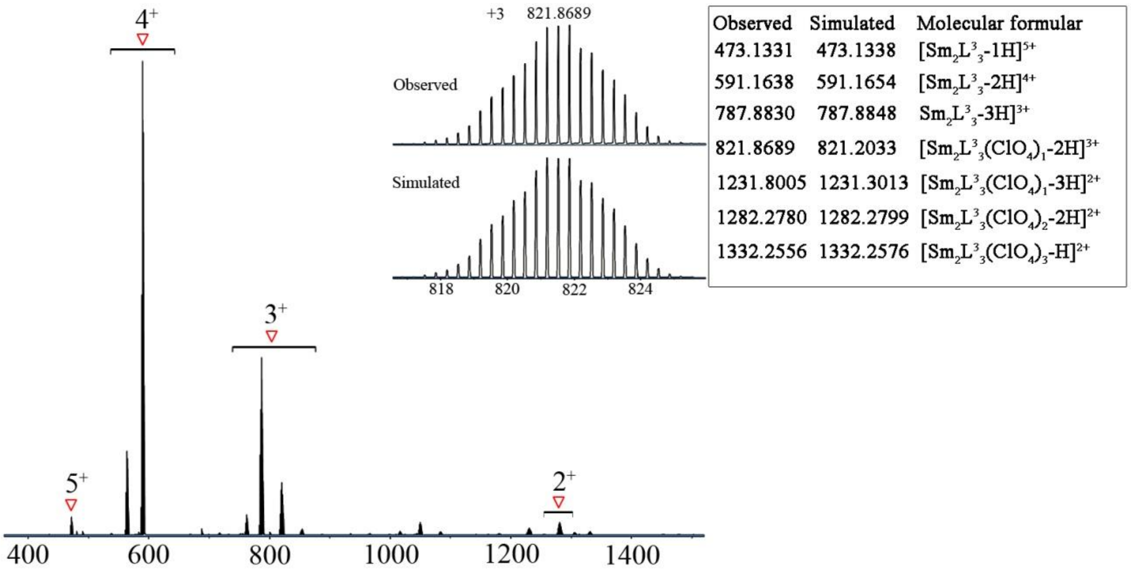

Observed	Simulated	Molecular formular
473.1331	473.1338	$[Sm_2L^3_3\text{-}1H]^{5+}$
591.1638	591.1654	$[Sm_2L^3_3\text{-}2H]^{4+}$
787.8830	787.8848	$Sm_2L^3_3\text{-}3H]^{3+}$
821.8689	821.2033	$[Sm_2L^3_3(ClO_4)_1\text{-}2H]^{3+}$
1231.8005	1231.3013	$[Sm_2L^3_3(ClO_4)_1\text{-}3H]^{2+}$
1282.2780	1282.2799	$[Sm_2L^3_3(ClO_4)_2\text{-}2H]^{2+}$
1332.2556	1332.2576	$[Sm_2L^3_3(ClO_4)_3\text{-}H]^{2+}$

Supplementary Figure 225. ESI-TOF-MS spectrum for **$[Sm_2L^{3S}{}_3](ClO_4)_6$** with insets showing the observed and simulated isotope patterns of the +3 peaks.

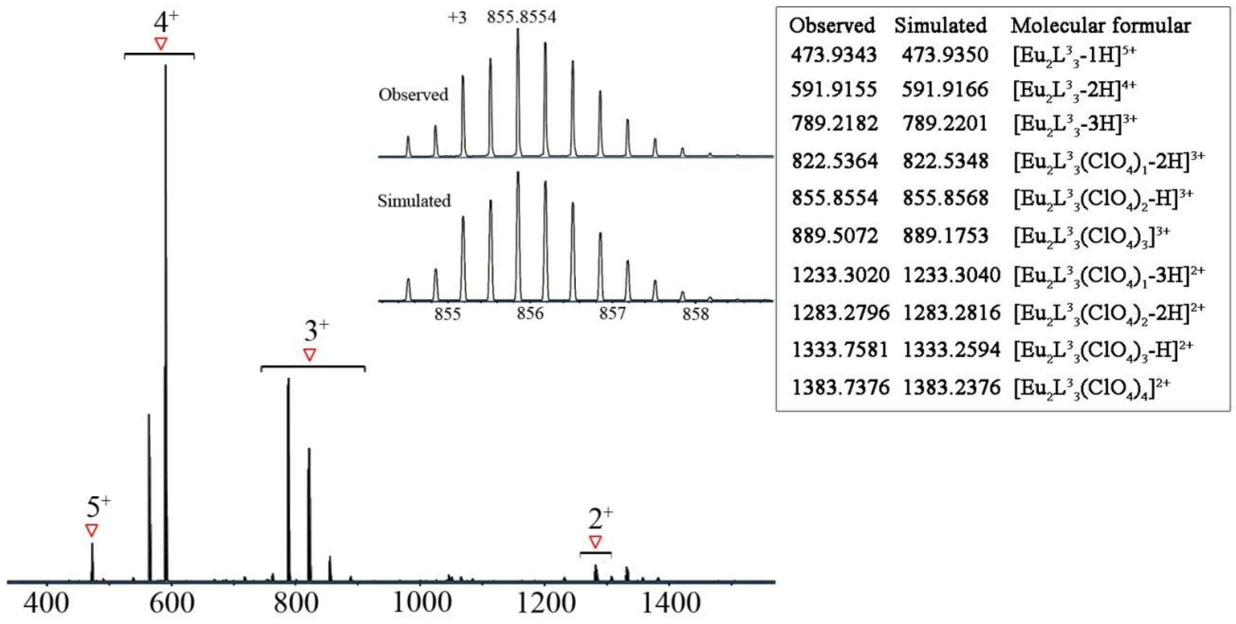

Supplementary Figure 226. ESI-TOF-MS spectrum for **$[Eu_2L^{3S}_3](ClO_4)_6$** with insets showing the observed and simulated isotope patterns of the +3 peaks.

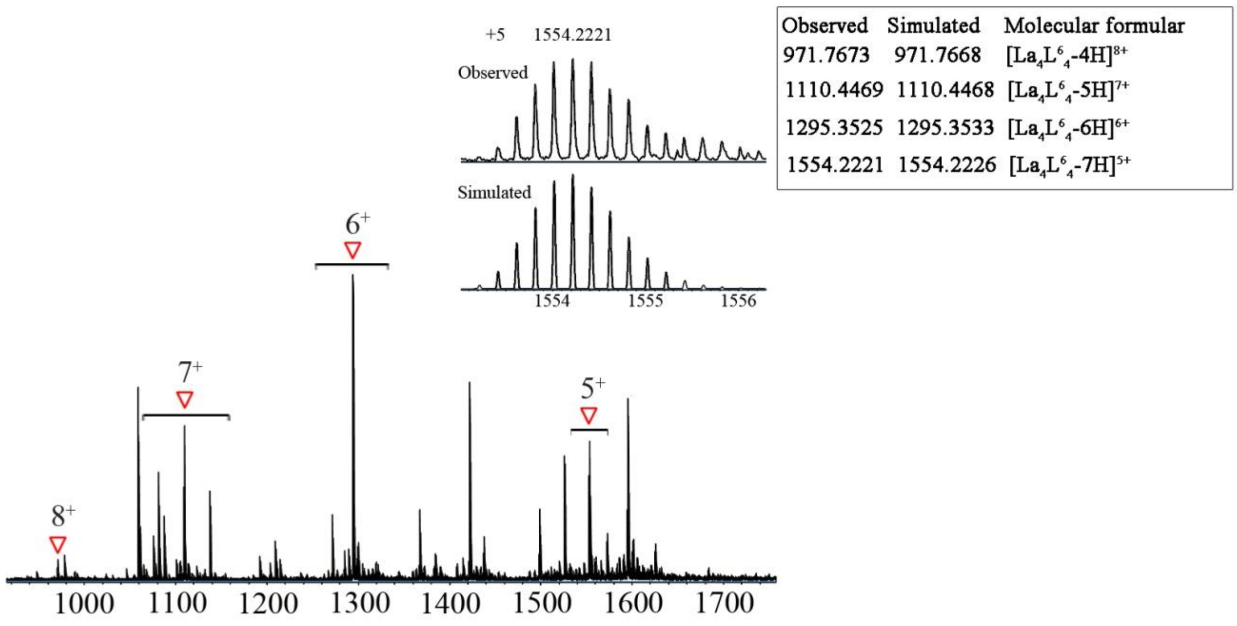

Supplementary Figure 227. ESI-TOF-MS spectrum for **$[La_4L^6_4](CF_3SO_3)_{12}$** with insets showing the observed and simulated isotope patterns of the +5 peaks.

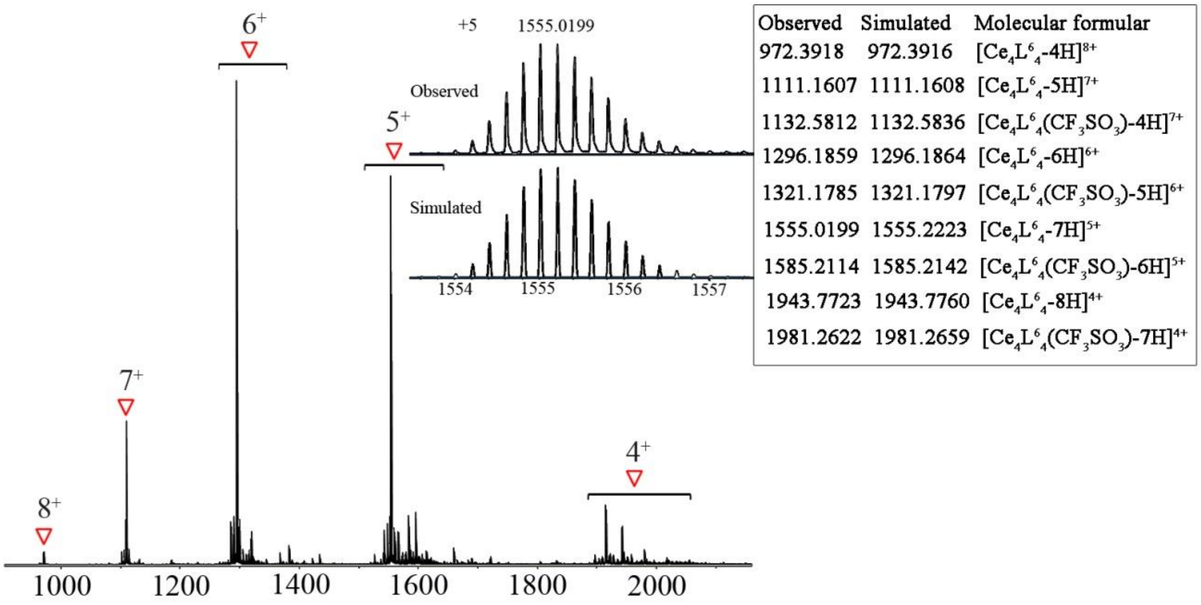

Supplementary Figure 228. ESI-TOF-MS spectrum for **$[Ce_4L^6_4](CF_3SO_3)_{12}$** with insets showing the observed and simulated isotope patterns of the +5 peaks.

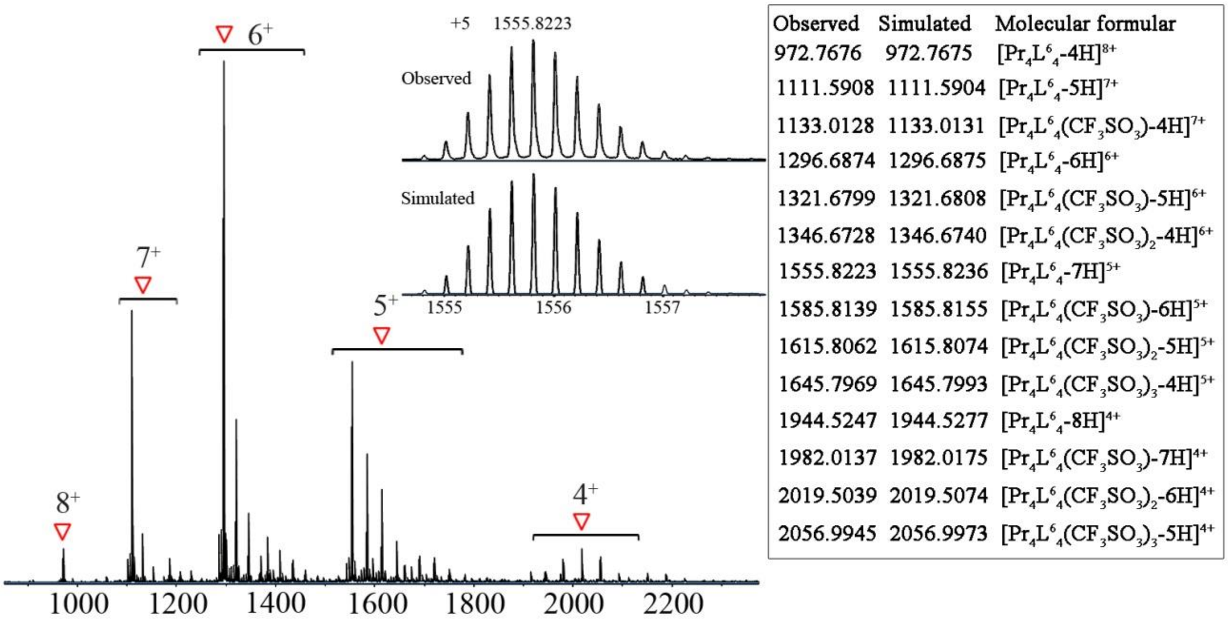

Supplementary Figure 229. ESI-TOF-MS spectrum for **$[Pr_4L^6_4](CF_3SO_3)_{12}$** with insets showing the observed and simulated isotope patterns of the +5 peaks.

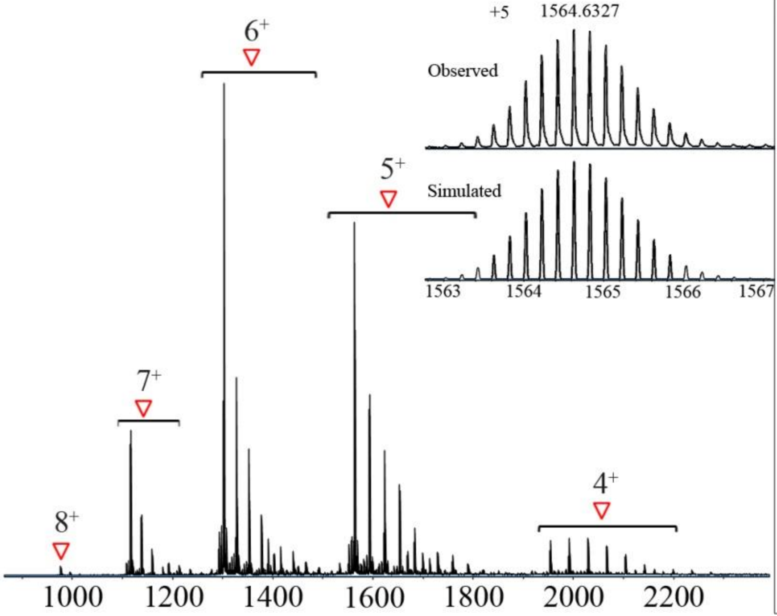

Observed	Simulated	Molecular formular
978.2744	978.2739	$[Eu_4L^6_4\text{-}4H]^{8+}$
1117.8844	1117.8834	$[Eu_4L^6_4\text{-}5H]^{7+}$
1139.3079	1139.3062	$[Eu_4L^6_4(CF_3SO_3)\text{-}4H]^{7+}$
1160.8738	1160.7290	$[Eu_4L^6_4(CF_3SO_3)_2\text{-}3H]^{7+}$
1304.1962	1304.0294	$[Eu_4L^6_4\text{-}6H]^{6+}$
1329.0232	1329.0227	$[Eu_4L^6_4(CF_3SO_3)\text{-}5H]^{6+}$
1354.0170	1354.0160	$[Eu_4L^6_4(CF_3SO_3)_2\text{-}4H]^{6+}$
1379.1776	1379.1762	$[Eu_4L^6_4(CF_3SO_3)_3\text{-}3H]^{6+}$
1564.6327	1564.6339	$[Eu_4L^6_4\text{-}7H]^{5+}$
1594.8247	1594.6258	$[Eu_4L^6_4(CF_3SO_3)\text{-}6H]^{5+}$
1624.6175	1624.6177	$[Eu_4L^6_4(CF_3SO_3)_2\text{-}5H]^{5+}$
1654.8103	1654.8100	$[Eu_4L^6_4(CF_3SO_3)_3\text{-}4H]^{5+}$
1955.5380	1955.5405	$[Eu_4L^6_4\text{-}8H]^{4+}$
1993.0290	1993.0304	$[Eu_4L^6_4(CF_3SO_3)\text{-}7H]^{4+}$
2030.7695	2030.5203	$[Eu_4L^6_4(CF_3SO_3)_2\text{-}6H]^{4+}$
2068.2592	2068.2607	$[Eu_4L^6_4(CF_3SO_3)_3\text{-}5H]^{4+}$

Supplementary Figure 230. ESI-TOF-MS spectrum for $\mathbf{[Eu_4L^6_4](CF_3SO_3)_{12}}$ with insets showing the observed and simulated isotope patterns of the +5 peaks.

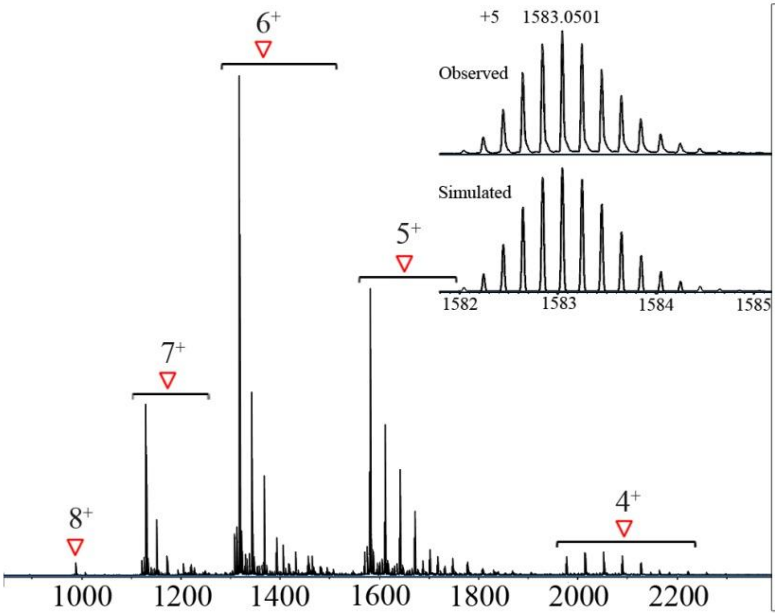

Observed	Simulated	Molecular formular
989.7853	989.7840	$[Lu_4L^6_4\text{-}4H]^{8+}$
1131.0398	1131.0378	$[Lu_4L^6_4\text{-}5H]^{7+}$
1152.4626	1152.4606	$[Lu_4L^6_4(CF_3SO_3)\text{-}4H]^{7+}$
1173.8856	1173.8834	$[Lu_4L^6_4(CF_3SO_3)_2\text{-}3H]^{7+}$
1319.3773	1319.3763	$[Lu_4L^6_4\text{-}6H]^{6+}$
1344.3709	1344.3695	$[Lu_4L^6_4(CF_3SO_3)\text{-}5H]^{6+}$
1369.3642	1369.3628	$[Lu_4L^6_4(CF_3SO_3)_2\text{-}4H]^{6+}$
1583.0501	1583.0501	$[Lu_4L^6_4\text{-}7H]^{5+}$
1613.0420	1613.0420	$[Lu_4L^6_4(CF_3SO_3)\text{-}6H]^{5+}$
1643.0345	1643.0339	$[Lu_4L^6_4(CF_3SO_3)_2\text{-}5H]^{5+}$
1673.0266	1673.0258	$[Lu_4L^6_4(CF_3SO_3)_3\text{-}4H]^{5+}$
1703.0183	1703.0177	$[Lu_4L^6_4(CF_3SO_3)_4\text{-}3H]^{5+}$
1978.5589	1978.5608	$[Lu_4L^6_4\text{-}8H]^{4+}$
2016.0494	2016.0506	$[Lu_4L^6_4(CF_3SO_3)\text{-}7H]^{4+}$
2053.7903	2053.5405	$[Lu_4L^6_4(CF_3SO_3)_2\text{-}6H]^{4+}$
2091.0300	2091.0304	$[Lu_4L^6_4(CF_3SO_3)_3\text{-}5H]^{4+}$

Supplementary Figure 231. ESI-TOF-MS spectrum for $\mathbf{[Lu_4L^6_4](CF_3SO_3)_{12}}$ with insets showing the observed and simulated isotope patterns of the +5 peaks.

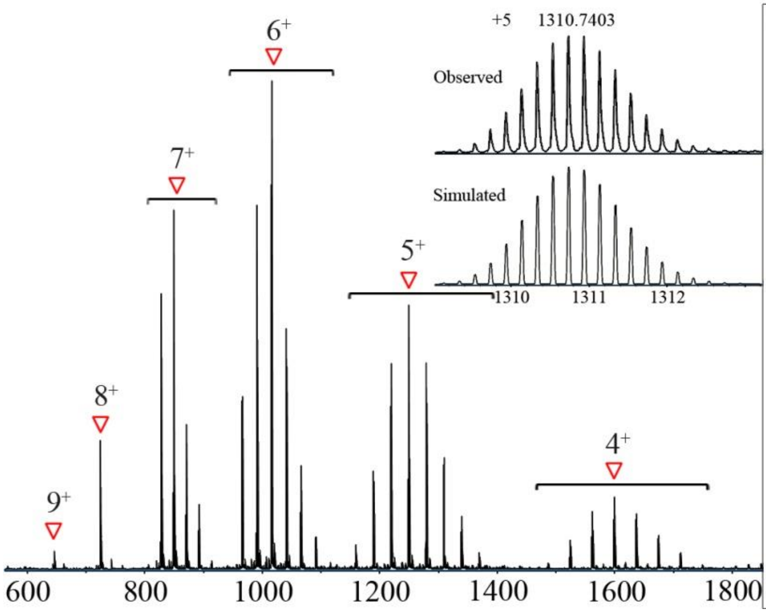

Observed	Simulated	Molecular formular
645.3273	645.3267	$[Eu_4L^4_4\text{-}3H]^{9+}$
725.8678	725.8667	$[Eu_4L^4_4\text{-}4H]^{8+}$
829.4190	829.4180	$[Eu_4L^4_4\text{-}5H]^{7+}$
850.8414	850.8408	$[Eu_4L^4_4(CF_3SO_3)\text{-}4H]^{7+}$
872.4070	872.2636	$[Eu_4L^4_4(CF_3SO_3)_2\text{-}3H]^{7+}$
893.6870	893.6864	$[Eu_4L^4_4(CF_3SO_3)_3\text{-}2H]^{7+}$
967.4875	967.4865	$[Eu_4L^4_4\text{-}6H]^{6+}$
992.4802	992.4797	$[Eu_4L^4_4(CF_3SO_3)\text{-}5H]^{6+}$
1017.4729	1017.4730	$[Eu_4L^4_4(CF_3SO_3)_2\text{-}4H]^{6+}$
1042.4658	1042.4663	$[Eu_4L^4_4(CF_3SO_3)_3\text{-}3H]^{6+}$
1067.4587	1067.4595	$[Eu_4L^4_4(CF_3SO_3)_4\text{-}2H]^{6+}$
1250.7570	1250.7580	$[Eu_4L^4_4(CF_3SO_3)_3\text{-}4H]^{5+}$
1280.7484	1280.7500	$[Eu_4L^4_4(CF_3SO_3)_4\text{-}3H]^{5+}$
1310.7403	1310.7419	$[Eu_4L^4_4(CF_3SO_3)_5\text{-}2H]^{5+}$
1600.6835	1600.6856	$[Eu_4L^4_4(CF_3SO_3)_4\text{-}4H]^{4+}$
1638.1722	1638.1756	$[Eu_4L^4_4(CF_3SO_3)_5\text{-}3H]^{4+}$

Supplementary Figure 232. ESI-TOF-MS spectrum for $\mathbf{[Eu_4L^4_4](CF_3SO_3)_{12}}$ with insets showing the observed and simulated isotope patterns of the +5 peaks.

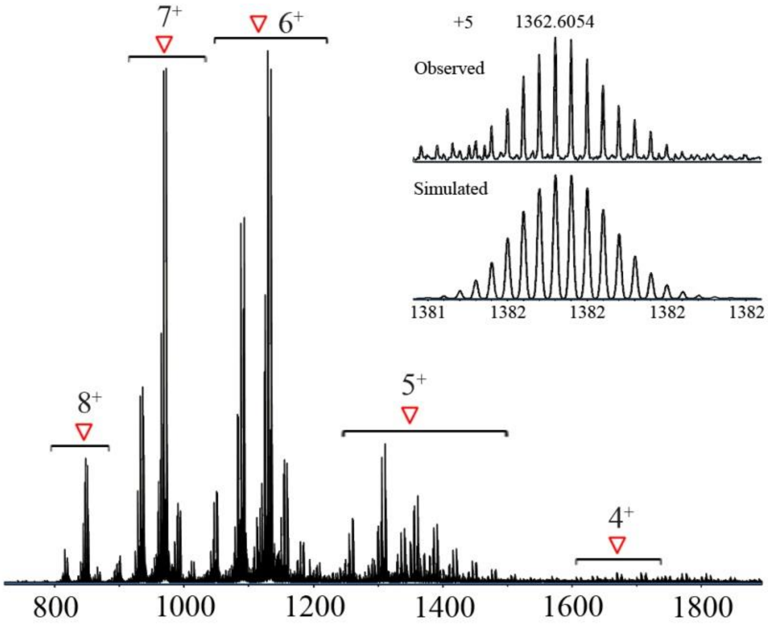

Observed	Simulated	Molecular formular
852.0065	852.0077	$[Eu_4L^5_4\text{-}4H]^{8+}$
973.5774	973.5791	$[Eu_4L^5_4\text{-}5H]^{7+}$
995.2864	995.1451	$[Eu_4L^5_4(CF_3SO_3)\text{-}4H]^{7+}$
1135.6725	1135.6745	$[Eu_4L^5_4\text{-}6H]^{6+}$
1160.6651	1160.8347	$[Eu_4L^5_4(CF_3SO_3)\text{-}5H]^{6+}$
1185.8255	1185.8280	$[Eu_4L^5_4(CF_3SO_3)_2\text{-}4H]^{6+}$
1362.6054	1362.6079	$[Eu_4L^5_4\text{-}7H]^{5+}$
1392.7972	1392.8002	$[Eu_4L^5_4(CF_3SO_3)\text{-}6H]^{5+}$
1422.5884	1422.7921	$[Eu_4L^5_4(CF_3SO_3)_2\text{-}5H]^{5+}$
1452.9824	1452.7840	$[Eu_4L^5_4(CF_3SO_3)_3\text{-}4H]^{5+}$
1703.0065	1703.0080	$[Eu_4L^5_4\text{-}8H]^{4+}$
1740.7442	1740.7484	$[Eu_4L^5_4(CF_3SO_3)\text{-}7H]^{4+}$
1778.2319	1778.2383	$[Eu_4L^5_4(CF_3SO_3)_2\text{-}6H]^{4+}$
1815.7209	1815.7282	$[Eu_4L^5_4(CF_3SO_3)_3\text{-}5H]^{4+}$

Supplementary Figure 233. ESI-TOF-MS spectrum for $\mathbf{[Eu_4L^5_4](CF_3SO_3)_{12}}$along with signals created by rupture of alkyl groups, with insets showing the observed and simulated isotope patterns of the +5 peaks.

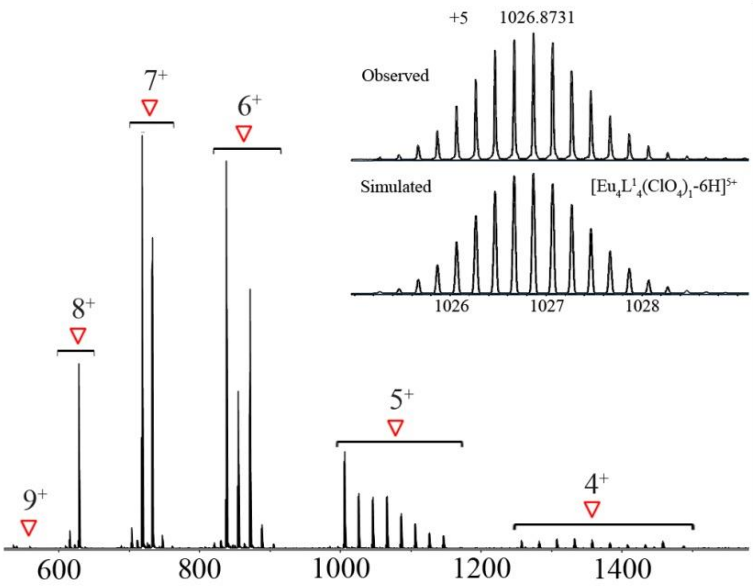

Observed	Simulated	Molecular formular
559.7162	559.7151	$[Eu_4L^1_4\text{-}3H]^{9+}$
629.5544	629.5535	$[Eu_4L^1_4\text{-}4H]^{8+}$
719.3468	719.3459	$[Eu_4L^1_4\text{-}5H]^{7+}$
733.7690	733.7682	$[Eu_4L^1_4(ClO_4)_1\text{-}4H]^{7+}$
839.0699	839.0690	$[Eu_4L^1_4\text{-}6H]^{6+}$
855.8955	855.8950	$[Eu_4L^1_4(ClO_4)_1\text{-}5H]^{6+}$
872.5551	872.5542	$[Eu_4L^1_4(ClO_4)_2\text{-}4H]^{6+}$
1006.6818	1006.6813	$[Eu_4L^1_4\text{-}7H]^{5+}$
1026.8731	1026.8726	$[Eu_4L^1_4(ClO_4)_1\text{-}6H]^{5+}$
1046.8639	1046.8636	$[Eu_4L^1_4(ClO_4)_2\text{-}5H]^{5+}$
1067.0555	1067.0549	$[Eu_4L^1_4(ClO_4)_3\text{-}4H]^{5+}$
1087.0466	1087.0459	$[Eu_4L^1_4(ClO_4)_4\text{-}3H]^{5+}$
1283.3385	1283.3389	$[Eu_4L^1_4(ClO_4)_1\text{-}7H]^{4+}$
1308.5775	1308.3277	$[Eu_4L^1_4(ClO_4)_2\text{-}6H]^{4+}$
1333.5661	1333.5668	$[Eu_4L^1_4(ClO_4)_3\text{-}5H]^{4+}$
1358.5551	1358.5556	$[Eu_4L^1_4(ClO_4)_4\text{-}4H]^{4+}$

Supplementary Figure 234. ESI-TOF-MS spectrum for titration of **L^1** (0.002M) with $Eu(ClO_4)_3$ Ⱨ$_2$O when $R_{EuIII/L1}$=5 with insets showing the observed and simulated isotope patterns of the +5 peaks.

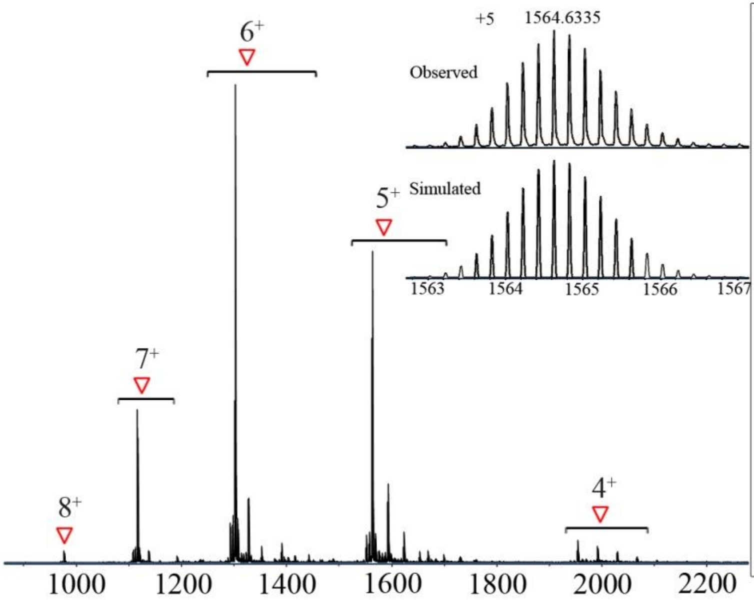

Observed	Simulated	Molecular formular
978.3998	978.2739	$[Eu_4L^6_4\text{-}4H]^{8+}$
1117.8843	1117.8834	$[Eu_4L^6_4\text{-}5H]^{7+}$
1139.3064	1139.3062	$[Eu_4L^6_4(CF_3SO_3)\text{-}4H]^{7+}$
1304.0299	1304.0294	$[Eu_4L^6_4\text{-}6H]^{6+}$
1329.1889	1329.0227	$[Eu_4L^6_4(CF_3SO_3)\text{-}5H]^{6+}$
1354.1821	1354.0160	$[Eu_4L^6_4(CF_3SO_3)_2\text{-}4H]^{6+}$
1564.6335	1564.6339	$[Eu_4L^6_4\text{-}7H]^{5+}$
1594.8244	1594.6258	$[Eu_4L^6_4(CF_3SO_3)\text{-}6H]^{5+}$
1624.6164	1624.6177	$[Eu_4L^6_4(CF_3SO_3)_2\text{-}5H]^{5+}$
1654.8076	1654.8100	$[Eu_4L^6_4(CF_3SO_3)_3\text{-}4H]^{5+}$
1955.5384	1955.5405	$[Eu_4L^6_4\text{-}8H]^{4+}$
1993.2777	1993.0304	$[Eu_4L^6_4(CF_3SO_3)\text{-}7H]^{4+}$
2030.7678	2030.5203	$[Eu_4L^6_4(CF_3SO_3)_2\text{-}6H]^{4+}$
2068.2587	2068.2607	$[Eu_4L^6_4(CF_3SO_3)_3\text{-}5H]^{4+}$
2106.0005	2105.7506	$[Eu_4L^6_4(CF_3SO_3)_4\text{-}4H]^{4+}$

Supplementary Figure 235. ESI-TOF-MS spectrum for titration of **L^6** with $Eu(ClO_4)_3$ Ⱨ$_2$O when $R_{EuIII/L6}$=5 with insets showing the observed and simulated isotope patterns of the +5 peaks.

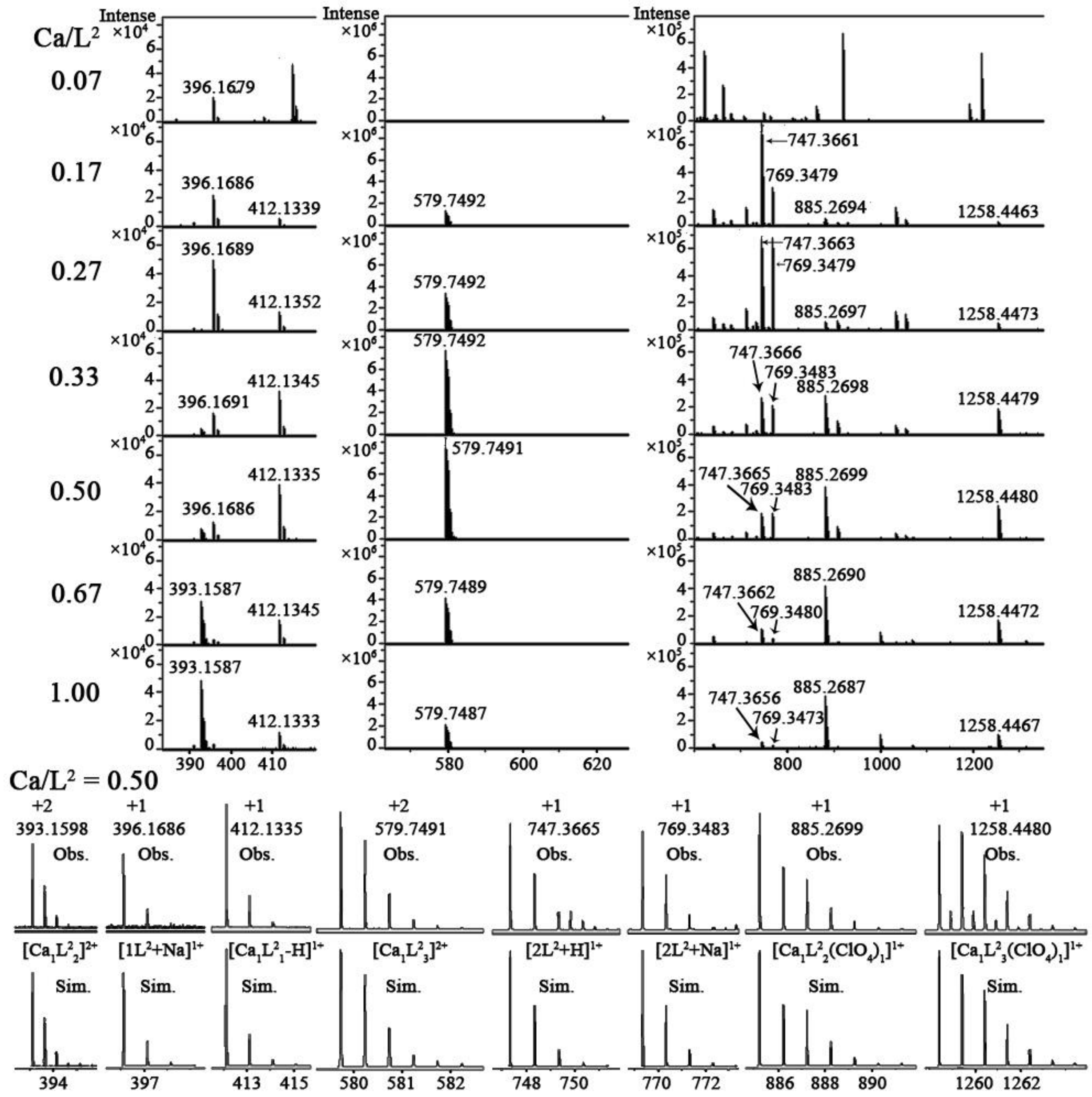

Supplementary Figure 236. ESI-TOF-MS spectra for titration of $\mathbf{L^2}$ with $Ca(ClO_4)_2 \cdot 4H_2O$ when $R_{Ca/L2}$= 0.07-1.00 with insets showing the observed and simulated isotope patterns of the observed peaks.

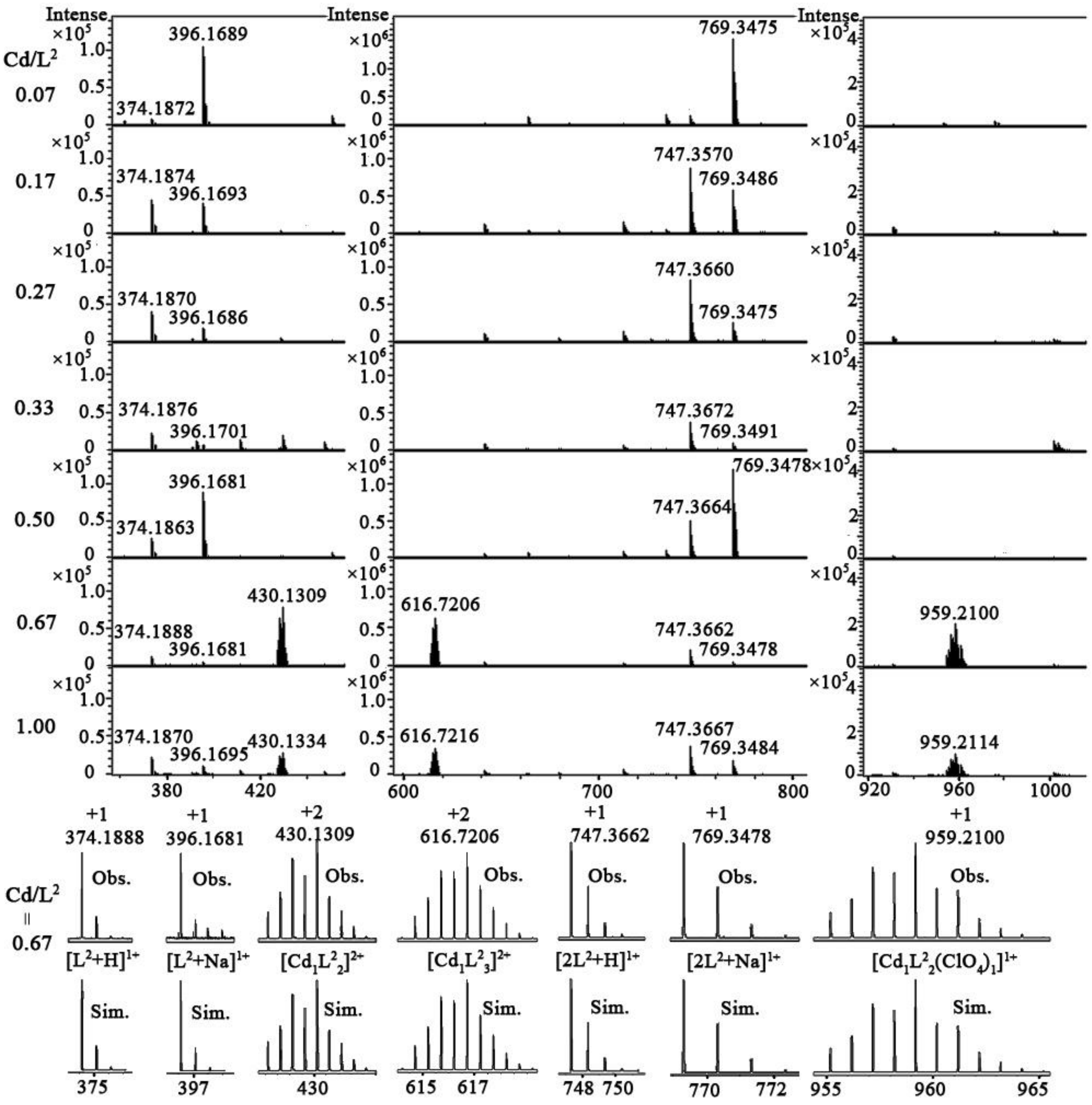

Supplementary Figure 237. ESI-TOF-MS spectra for titration of $\mathbf{L^2}$ with $Cd(ClO_4)_2 \cdot 6H_2O$ when $R_{Cd/L2}$= 0.07-1.00 with insets showing the observed and simulated isotope patterns of the observed peaks.

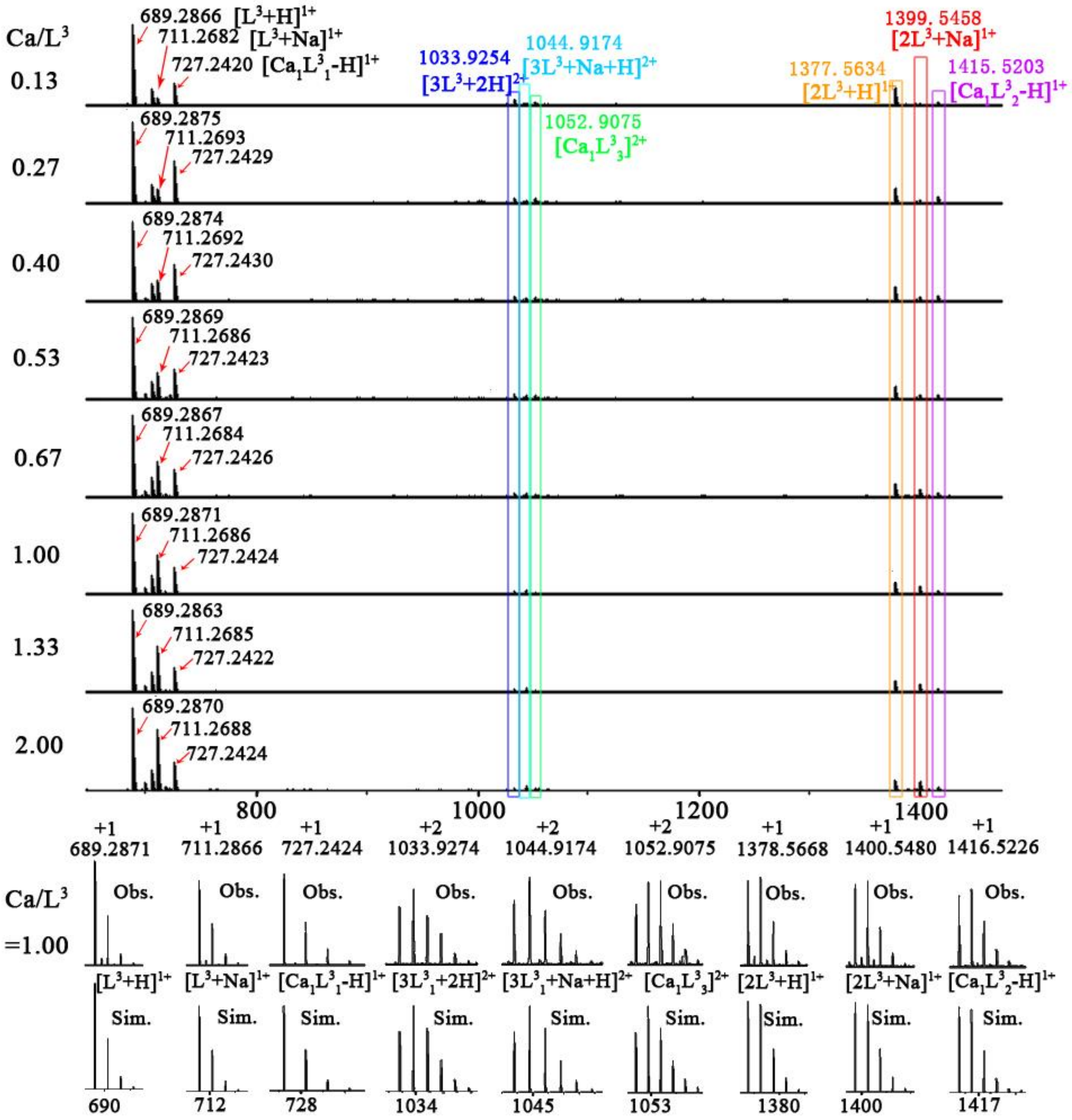

Supplementary Figure 238. ESI-TOF-MS spectra for titration of $\mathbf{L^3}$ with $Ca(ClO_4)_2 \cdot 4H_2O$ when $R_{Ca/L3}$= 0.13-2.00 with insets showing the observed and simulated isotope patterns of the observed peaks.

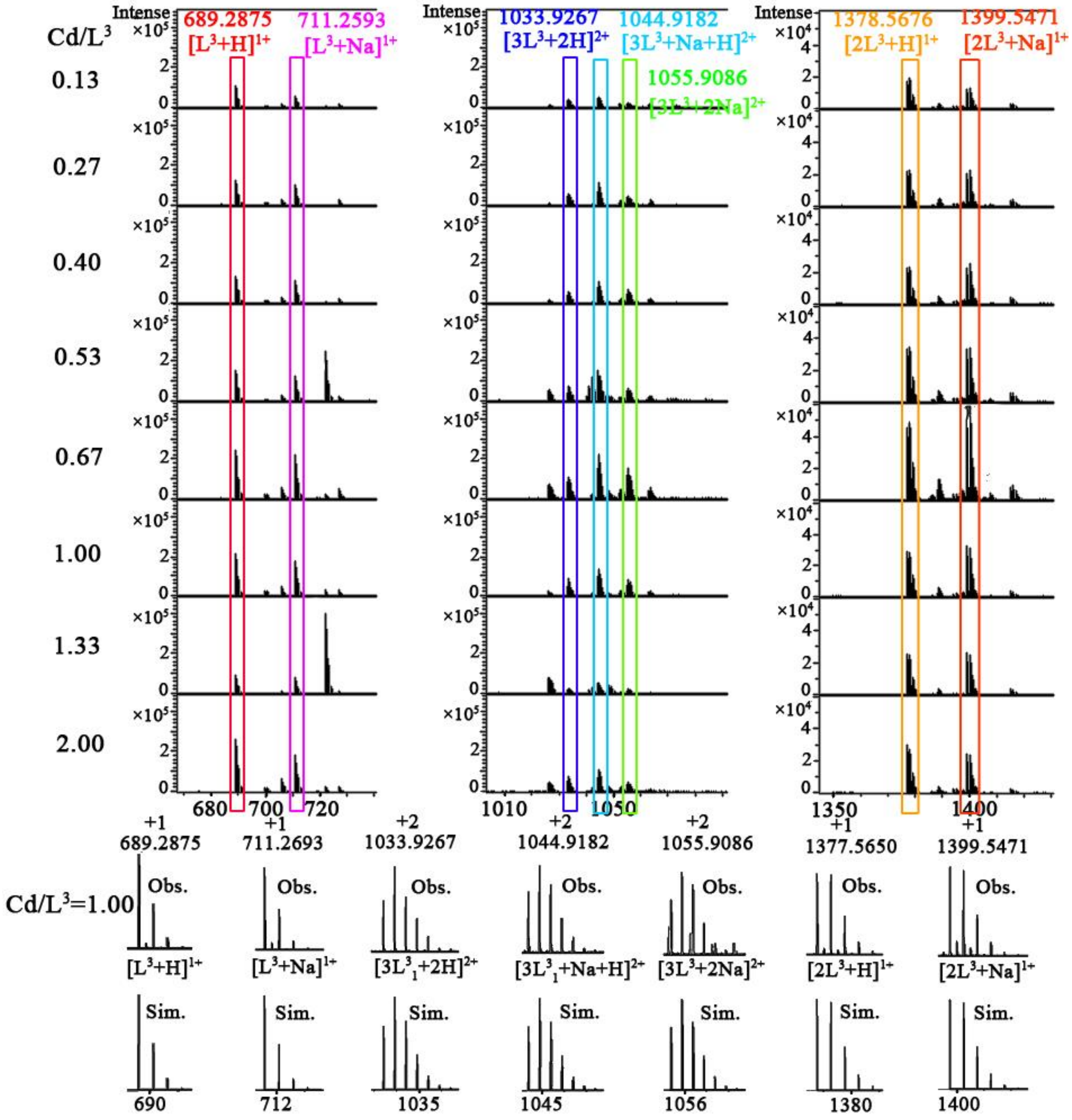

Supplementary Figure 239. ESI-TOF-MS spectra for titration of $\mathbf{L^3}$ with $Cd(ClO_4)_2$ $6H_2O$ when $R_{Cd/L3}$= 0.13-2.00 with insets showing the observed and simulated isotope patterns of the observed peaks.

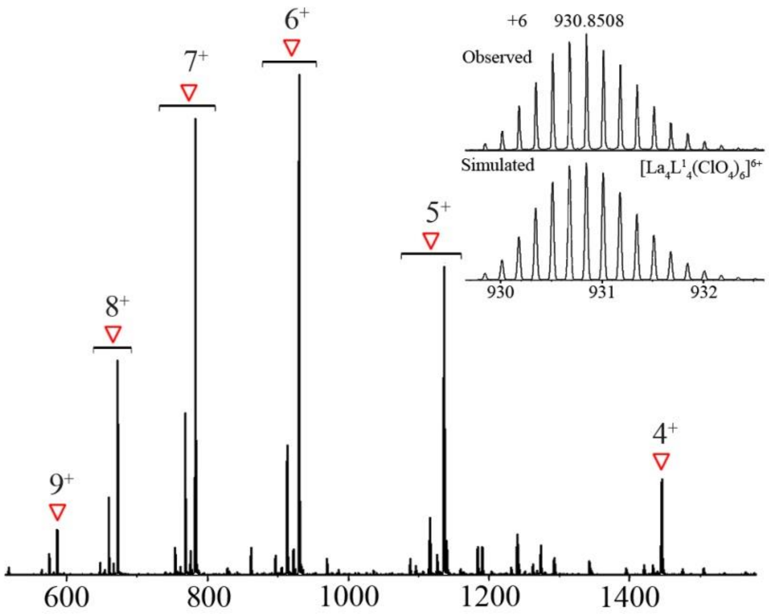

Observed	Simulated	Molecular formular
576.1453	576.2545	$[La_4L^1_4(ClO_4)_2\text{-}1H]^{9+}$
587.3623	587.3607	$[La_4L^1_4(ClO_4)_3]^{9+}$
648.0372	648.1604	$[La_4L^1_4(ClO_4)_2\text{-}2H]^{8+}$
660.6568	660.6548	$[La_4L^1_4(ClO_4)_3\text{-}1H]^{8+}$
673.1514	673.2744	$[La_4L^1_4(ClO_4)_4]^{8+}$
755.0342	754.8902	$[La_4L^1_4(ClO_4)_3\text{-}2H]^{7+}$
769.1713	769.3125	$[La_4L^1_4(ClO_4)_4\text{-}1H]^{7+}$
783.5939	783.5919	$[La_4L^1_4(ClO_4)_5]^{7+}$
897.5313	897.3634	$[La_4L^1_4(ClO_4)_4\text{-}2H]^{6+}$
914.0245	914.0226	$[La_4L^1_4(ClO_4)_5\text{-}1H]^{6+}$
930.8508	930.8486	$[La_4L^1_4(ClO_4)_6]^{6+}$
1116.8183	1116.8169	$[La_4L^1_4(ClO_4)_6\text{-}1H]^{5+}$
1136.8102	1136.8080	$[La_4L^1_4(ClO_4)_7]^{5+}$
1446.2492	1445.9971	$[La_4L^1_4(ClO_4)_8]^{4+}$

Supplementary Figure 240. ESI-TOF-MS spectrum for La^{III}-Ca^{II} mixed-metal self-assembly of $\mathbf{L^1}$(ClO_4^- counter-anion) with insets showing the observed and simulated isotope patterns of the +6 peaks.

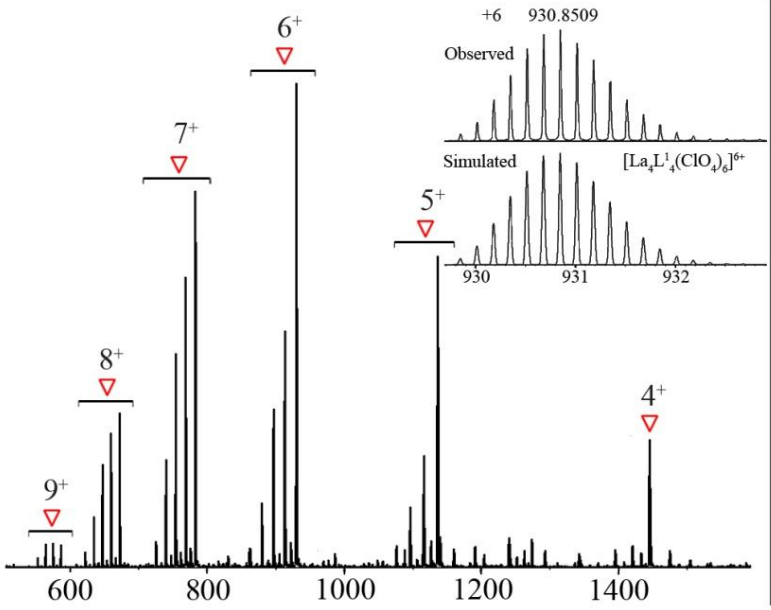

Observed	Simulated	Molecular formular
565.1504	565.0371	$[La_4L^1_4(ClO_4)_1\text{-}2H]^{9+}$
576.2561	576.2545	$[La_4L^1_4(ClO_4)_2\text{-}1H]^{9+}$
587.3632	587.3607	$[La_4L^1_4(ClO_4)_3]^{9+}$
635.5430	635.5408	$[La_4L^1_4(ClO_4)_1\text{-}3H]^{8+}$
648.1625	648.1604	$[La_4L^1_4(ClO_4)_2\text{-}2H]^{8+}$
660.6571	660.6548	$[La_4L^1_4(ClO_4)_3\text{-}1H]^{8+}$
673.1519	673.2744	$[La_4L^1_4(ClO_4)_4]^{8+}$
755.0355	754.8902	$[La_4L^1_4(ClO_4)_3\text{-}2H]^{7+}$
769.3146	769.3125	$[La_4L^1_4(ClO_4)_4\text{-}1H]^{7+}$
783.5939	783.5919	$[La_4L^1_4(ClO_4)_5]^{7+}$
897.3655	897.3634	$[La_4L^1_4(ClO_4)_4\text{-}2H]^{6+}$
914.0248	914.0226	$[La_4L^1_4(ClO_4)_5\text{-}1H]^{6+}$
930.8509	930.8486	$[La_4L^1_4(ClO_4)_6]^{6+}$
1116.8195	1116.8169	$[La_4L^1_4(ClO_4)_6\text{-}1H]^{5+}$
1136.8104	1136.808	$[La_4L^1_4(ClO_4)_7]^{5+}$
1446.2492	1446.2497	$[La_4L^1_4(ClO_4)_8]^{4+}$

Supplementary Figure 241. ESI-TOF-MS spectrum for La^{III}-Cd^{II} mixed-metal self-assembly of $\mathbf{L^1}$(ClO_4^- counter-anion) with insets showing the observed and simulated isotope patterns of the +6 peaks.

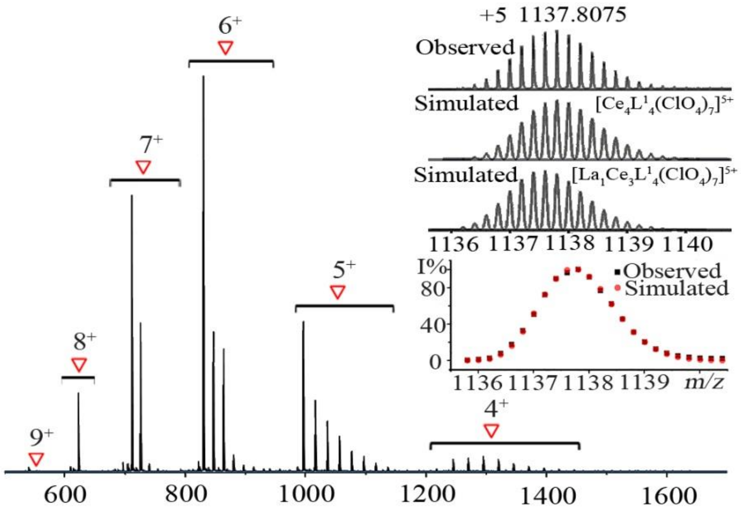

Observed	Simulated	Molecular formular
554.3754	554.3750	$[Ce_4L^1_4-3H]^{9+}$
623.5465	623.5460	$[Ce_4L^1_4-4H]^{8+}$
712.4803	712.4800	$[Ce_4L^1_4-5H]^{7+}$
726.9026	726.9025	$[Ce_4L^1_4(ClO_4)_1-4H]^{7+}$
831.0590	831.0588	$[Ce_4L^1_4-6H]^{6+}$
847.7177	847.8851	$[Ce_4L^1_4(ClO_4)_1-5H]^{6+}$
864.5440	864.7442	$[Ce_4L^1_4(ClO_4)_2-4H]^{6+}$
997.0692	997.0692	$[Ce_4L^1_4-7H]^{5+}$
1017.2602	1017.2606	$[Ce_4L^1_4(ClO_4)_1-6H]^{5+}$
1037.2512	1037.2516	$[Ce_4L^1_4(ClO_4)_2-5H]^{5+}$
1057.2424	1057.4430	$[Ce_4L^1_4(ClO_4)_3-4H]^{5+}$
1077.4339	1077.6342	$[Ce_4L^1_4(ClO_4)_4-3H]^{5+}$
1097.6250	1097.6253	$[Ce_4L^1_4(ClO_4)_5-2H]^{5+}$
1117.6167	1117.8165	$[Ce_4L^1_4(ClO_4)_6-1H]^{5+}$
1137.8075	1137.8076	$[Ce_4L^1_4(ClO_4)_7]^{5+}$
1296.3119	1296.3127	$[Ce_4L^1_4(ClO_4)_2-6H]^{4+}$

Supplementary Figure 242. ESI-TOF-MS spectrum for La^{III}-Ce^{III} mixed-metal self-assembly of $\mathbf{L^1}$(ClO_4^- counter-anion) with insets showing the observed and simulated isotope patterns of the +5 peaks.

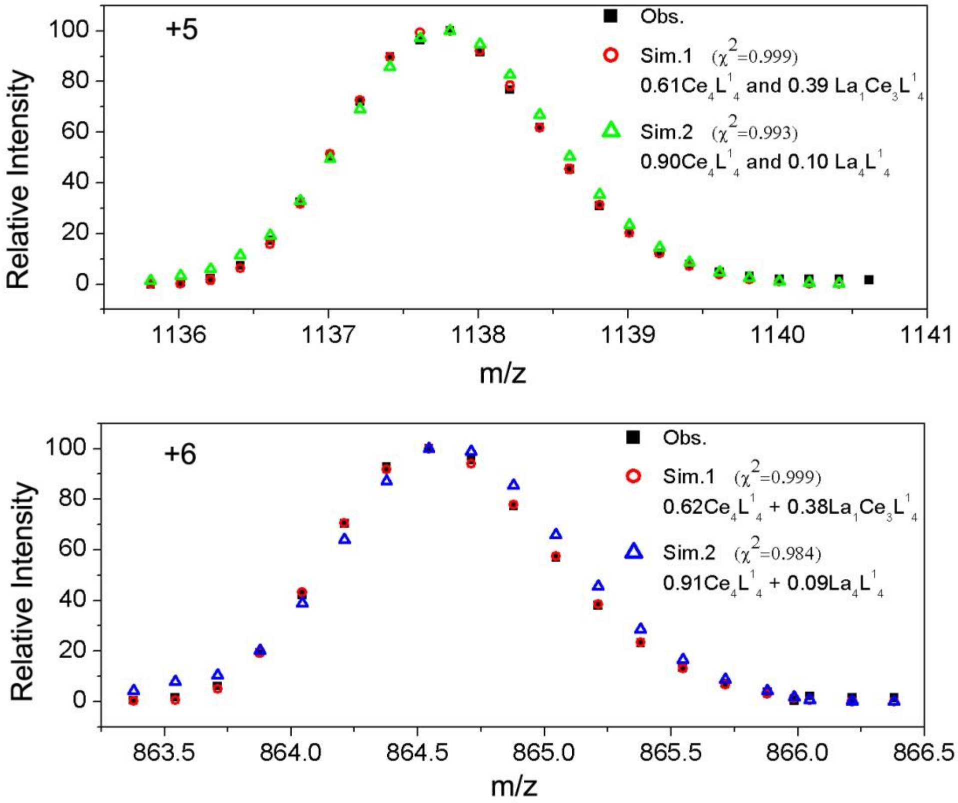

Supplementary Figure 243. Nonlinear curve fitting of simulated isotope patterns of La^{III}-Ce^{III} mixed-metal self-assembly complexes (ClO_4^- counter-anion).

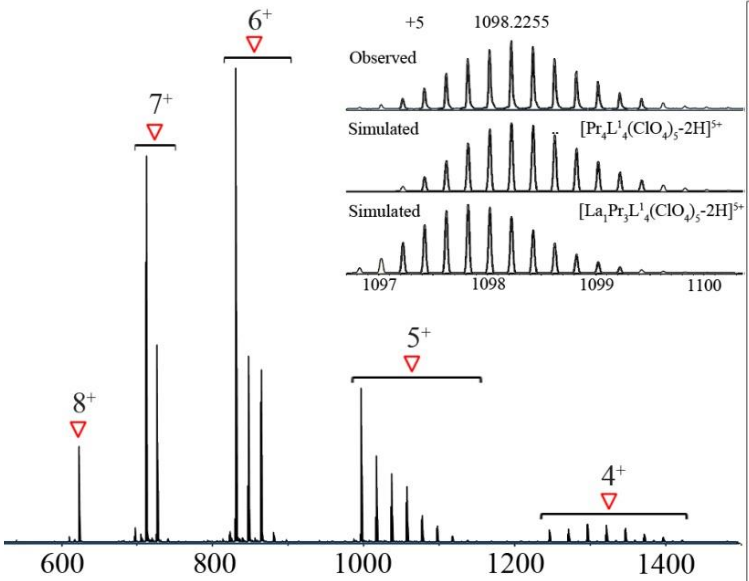

Observed	Simulated	Molecular formular
624.0470	624.0471	$[Pr_4L^1{}_4\text{-}4H]^{8+}$
712.9094	713.0528	$[Pr_4L^1{}_4\text{-}5H]^{7+}$
727.3314	727.3321	$[Pr_4L^1{}_4(ClO4)_1\text{-}4H]^{7+}$
831.5598	831.7271	$[Pr_4L^1{}_4\text{-}6H]^{6+}$
848.3855	848.3862	$[Pr_4L^1{}_4(ClO4)_1\text{-}5H]^{6+}$
865.0444	865.2123	$[Pr_4L^1{}_4(ClO4)_2\text{-}4H]^{6+}$
997.6700	997.8710	$[Pr_4L^1{}_4\text{-}7H]^{5+}$
1017.8613	1017.8620	$[Pr_4L^1{}_4(ClO4)_1\text{-}6H]^{5+}$
1037.8524	1038.0534	$[Pr_4L^1{}_4(ClO4)_2\text{-}5H]^{5+}$
1058.0437	1058.0437	$[Pr_4L^1{}_4(ClO4)_3\text{-}4H]^{5+}$
1078.0345	1078.2356	$[Pr_4L^1{}_4(ClO4)_4\text{-}3H]^{5+}$
1098.2255	1098.2267	$[Pr_4L^1{}_4(ClO4)_5\text{-}2H]^{5+}$
1297.3138	1297.3149	$[Pr_4L^1{}_4(ClO4)_2\text{-}6H]^{4+}$
1322.3025	1322.3037	$[Pr_4L^1{}_4(ClO4)_3\text{-}5H]^{4+}$
1347.5409	1347.5427	$[Pr_4L^1{}_4(ClO4)_4\text{-}4H]^{4+}$
1372.5304	1372.5316	$[Pr_4L^1{}_4(ClO4)_5\text{-}3H]^{4+}$

Supplementary Figure 244. ESI-TOF-MS spectrum for La^{III}-Pr^{III} mixed-metal self-assembly of **L^1** (ClO_4^- counter-anion) with insets showing the observed and simulated isotope patterns of the +5 peaks.

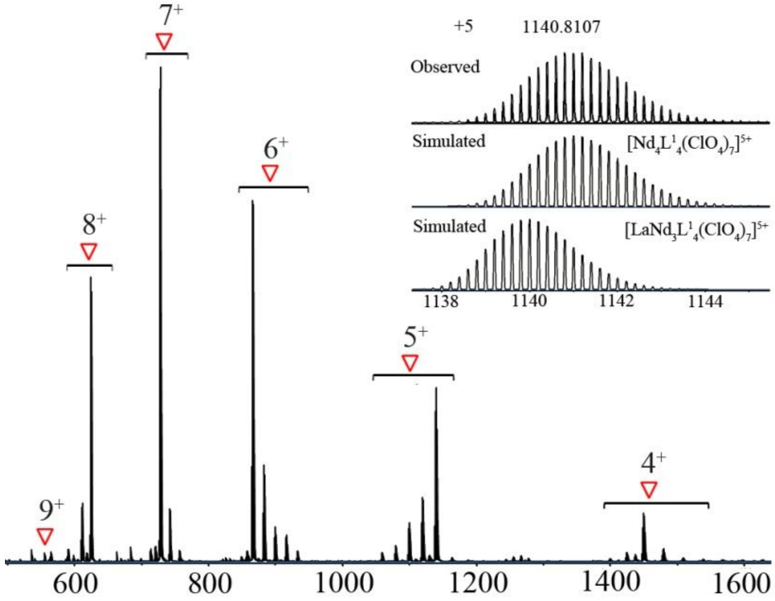

Observed	Simulated	Molecular formular
556.1551	556.1551	$[Nd_4L^1{}_4\text{-}3H]^{9+}$
625.4235	625.5486	$[Nd_4L^1{}_4\text{-}4H]^{8+}$
729.1914	729.1912	$[Nd_4L^1{}_4(ClO_4)_1\text{-}4H]^{7+}$
743.4704	743.4704	$[Nd_4L^1{}_4(ClO_4)_2\text{-}3H]^{7+}$
867.2144	867.2143	$[Nd_4L^1{}_4(ClO_4)_2\text{-}4H]^{6+}$
883.8733	884.0404	$[Nd_4L^1{}_4(ClO_4)_3\text{-}3H]^{6+}$
900.6993	900.6995	$[Nd_4L^1{}_4(ClO_4)_4\text{-}2H]^{6+}$
917.5252	917.5256	$[Nd_4L^1{}_4(ClO_4)_5\text{-}1H]^{6+}$
934.1845	934.1848	$[Nd_4L^1{}_4(ClO_4)_6]^{6+}$
1060.6462	1060.6470	$[Nd_4L^1{}_4(ClO_4)_3\text{-}4H]^{5+}$
1080.8375	1080.6380	$[Nd_4L^1{}_4(ClO_4)_4\text{-}3H]^{5+}$
1100.8287	1100.8293	$[Nd_4L^1{}_4(ClO_4)_5\text{-}2H]^{5+}$
1120.8197	1120.8203	$[Nd_4L^1{}_4(ClO_4)_6\text{-}1H]^{5+}$
1140.8107	1141.0116	$[Nd_4L^1{}_4(ClO_4)_7]^{5+}$
1451.2500	1451.2517	$[Nd_4L^1{}_4(ClO_4)_8]^{4+}$

Supplementary Figure 245. ESI-TOF-MS spectrum for La^{III}-Nd^{III} mixed-metal self-assembly of **L^1** (ClO_4^- counter-anion) with insets showing the observed and simulated isotope patterns of the +5 peaks.

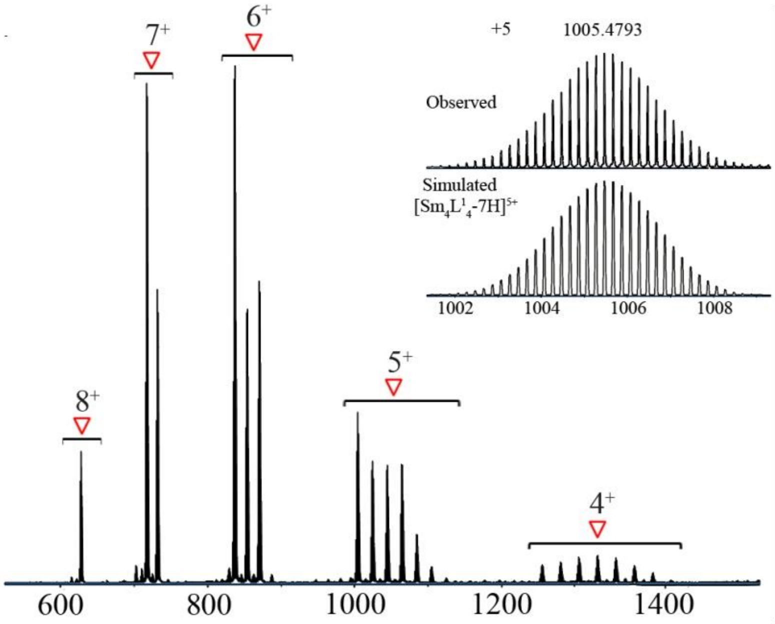

Observed	Simulated	Molecular formular
628.8028	628.8025	$[Sm_4L^1{}_4\text{-}4H]^{8+}$
718.4877	718.4875	$[Sm_4L^1{}_4\text{-}5H]^{7+}$
732.9098	732.9098	$[Sm_4L^1{}_4(ClO_4)_1\text{-}4H]^{7+}$
838.0676	838.0675	$[Sm_4L^1{}_4\text{-}6H]^{6+}$
854.8933	854.8936	$[Sm_4L^1{}_4(ClO_4)_1\text{-}5H]^{6+}$
871.7192	871.5528	$[Sm_4L^1{}_4(ClO_4)_2\text{-}4H]^{6+}$
1005.4793	1005.4796	$[Sm_4L^1{}_4\text{-}7H]^{5+}$
1025.6703	1025.6708	$[Sm_4L^1{}_4(ClO_4)_1\text{-}6H]^{5+}$
1045.8617	1045.6619	$[Sm_4L^1{}_4(ClO_4)_2\text{-}5H]^{5+}$
1065.4521	1065.4521	$[Sm_4L^1{}_4(ClO_4)_3\text{-}4H]^{5+}$
1086.0435	1085.8441	$[Sm_4L^1{}_4(ClO_4)_4\text{-}3H]^{5+}$
1281.5847	1281.8367	$[Sm_4L^1{}_4(ClO_4)_1\text{-}7H]^{4+}$
1306.5742	1306.8255	$[Sm_4L^1{}_4(ClO_4)_2\text{-}6H]^{4+}$
1332.0631	1332.0646	$[Sm_4L^1{}_4(ClO_4)_3\text{-}5H]^{4+}$
1356.8014	1357.0534	$[Sm_4L^1{}_4(ClO_4)_4\text{-}4H]^{4+}$
1382.2913	1382.2924	$[Sm_4L^1{}_4(ClO_4)_5\text{-}3H]^{4+}$

Supplementary Figure 246. ESI-TOF-MS spectrum for La^{III}-Sm^{III} mixed-metal self-assembly of **L^1** (ClO_4^- counter-anion) with insetsshowing the observed and simulated isotope patterns of the +5 peaks.

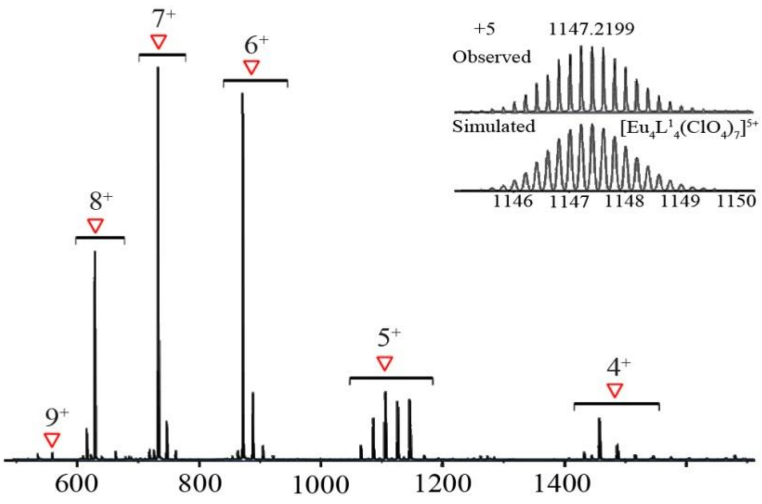

Observed	Simulated	Molecular formular
559.7151	559.7151	$[Eu_4L^1{}_4\text{-}3H]^{9+}$
629.5545	629.5535	$[Eu_4L^1{}_4\text{-}4H]^{8+}$
733.7692	733.7682	$[Eu_4L^1{}_4(ClO_4)_1\text{-}4H]^{7+}$
748.0483	748.0475	$[Eu_4L^1{}_4(ClO_4)_2\text{-}3H]^{7+}$
762.4707	762.4699	$[Eu_4L^1{}_4(ClO_4)_3\text{-}2H]^{7+}$
872.5554	872.5542	$[Eu_4L^1{}_4(ClO_4)_2\text{-}4H]^{6+}$
889.3811	889.3803	$[Eu_4L^1{}_4(ClO_4)_3\text{-}3H]^{6+}$
906.0404	906.0395	$[Eu_4L^1{}_4(ClO_4)_4\text{-}2H]^{6+}$
1067.0554	1067.0549	$[Eu_4L^1{}_4(ClO_4)_3\text{-}4H]^{5+}$
1087.0469	1087.0459	$[Eu_4L^1{}_4(ClO_4)_4\text{-}3H]^{5+}$
1107.2379	1107.2372	$[Eu_4L^1{}_4(ClO_4)_5\text{-}2H]^{5+}$
1127.2289	1127.2282	$[Eu_4L^1{}_4(ClO_4)_6\text{-}1H]^{5+}$
1147.2199	1147.4194	$[Eu_4L^1{}_4(ClO_4)_7]^{5+}$
1459.0109	1459.0113	$[Eu_4L^1{}_4(ClO_4)_8]^{4+}$

Supplementary Figure 247. ESI-TOF-MS spectrum for La^{III}-Eu^{III} mixed-metal self-assembly of **L^1** (ClO_4^- counter-anion) with insets showing the observed and simulated isotope patterns of the +5 peaks.

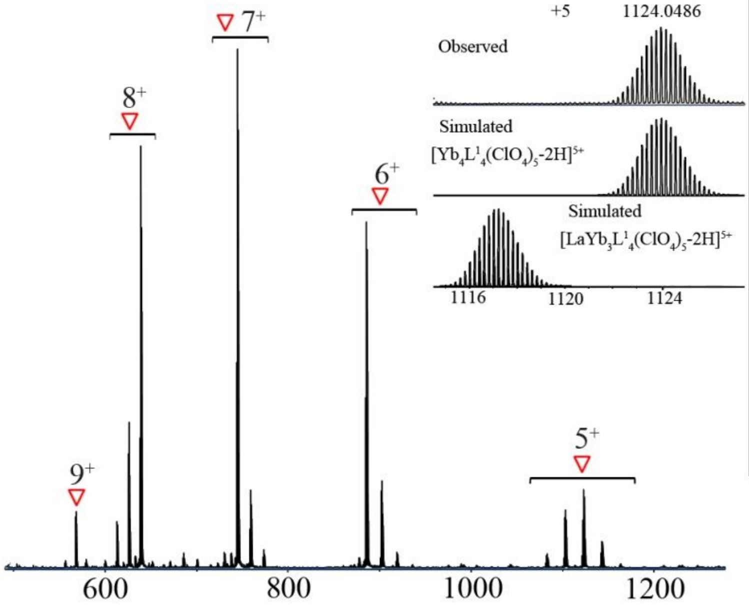

Observed	Simulated	Molecular formular
569.0554	569.0562	$[Yb_4L^1{}_4\text{-}3H]^{9+}$
640.0611	640.0623	$[Yb_4L^1{}_4\text{-}4H]^{8+}$
745.7765	745.7782	$[Yb_4L^1{}_4(ClO_4)_1\text{-}4H]^{7+}$
760.0554	760.0575	$[Yb_4L^1{}_4(ClO_4)_2\text{-}3H]^{7+}$
774.4776	774.4799	$[Yb_4L^1{}_4(ClO_4)_3\text{-}2H]^{7+}$
886.5641	886.5659	$[Yb_4L^1{}_4(ClO_4)_2\text{-}4H]^{6+}$
903.3897	903.5557	$[Yb_4L^1{}_4(ClO_4)_3\text{-}3H]^{6+}$
920.0490	920.0512	$[Yb_4L^1{}_4(ClO_4)_4\text{-}2H]^{6+}$
1083.6661	1083.8689	$[Yb_4L^1{}_4(ClO_4)_3\text{-}4H]^{5+}$
1104.0576	1103.8599	$[Yb_4L^1{}_4(ClO_4)_4\text{-}3H]^{5+}$
1124.0486	1124.0512	$[Yb_4L^1{}_4(ClO_4)_5\text{-}2H]^{5+}$
1144.0394	1144.0422	$[Yb_4L^1{}_4(ClO_4)_6\text{-}1H]^{5+}$

Supplementary Figure 248. ESI-TOF-MS spectrum for La^{III}-Yb^{III} mixed-metal self-assembly of $\mathbf{L^1}$ (ClO_4^- counter-anion) with insets showing the observed and simulated isotope patterns of the +5 peaks.

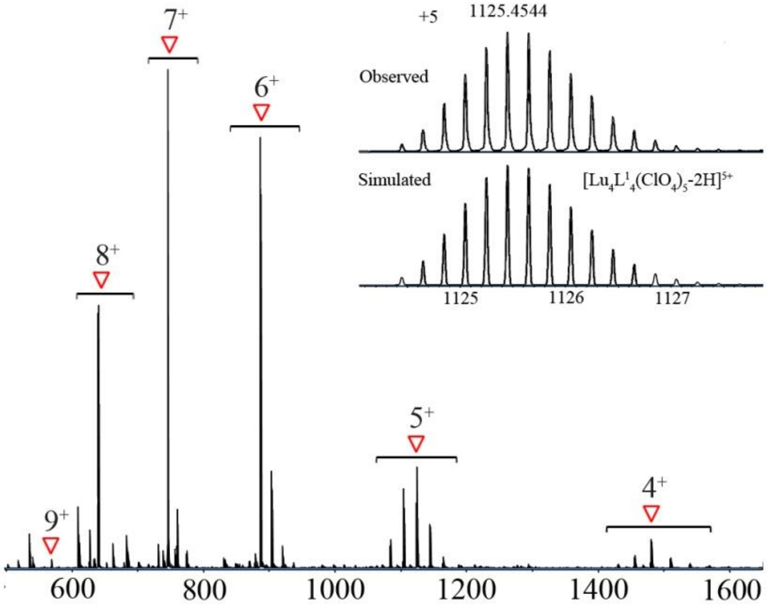

Observed	Simulated	Molecular formular
569.9479	569.9463	$[Lu_4L^1{}_4\text{-}3H]^{9+}$
641.0649	641.0637	$[Lu_4L^1{}_4\text{-}4H]^{8+}$
746.7809	746.7796	$[Lu_4L^1{}_4(ClO_4)_1\text{-}4H]^{7+}$
761.2040	761.2020	$[Lu_4L^1{}_4(ClO_4)_2\text{-}3H]^{7+}$
775.4828	775.4813	$[Lu_4L^1{}_4(ClO_4)_3\text{-}2H]^{7+}$
887.9026	887.9011	$[Lu_4L^1{}_4(ClO_4)_2\text{-}4H]^{6+}$
904.5616	904.5603	$[Lu_4L^1{}_4(ClO_4)_3\text{-}3H]^{6+}$
921.2209	921.3863	$[Lu_4L^1{}_4(ClO_4)_4\text{-}2H]^{6+}$
1085.4720	1085.2709	$[Lu_4L^1{}_4(ClO_4)_3\text{-}4H]^{5+}$
1105.4636	1105.4622	$[Lu_4L^1{}_4(ClO_4)_4\text{-}3H]^{5+}$
1125.4544	1125.4532	$[Lu_4L^1{}_4(ClO_4)_5\text{-}2H]^{5+}$
1145.6454	1145.6445	$[Lu_4L^1{}_4(ClO_4)_6\text{-}1H]^{5+}$
1456.7923	1456.7926	$[Lu_4L^1{}_4(ClO_4)_7\text{-}1H]^{4+}$
1482.0321	1482.0316	$[Lu_4L^1{}_4(ClO_4)_8]^{4+}$

Supplementary Figure 249. ESI-TOF-MS spectrum for La^{III}-Lu^{III} mixed-metal self-assembly of $\mathbf{L^1}$ (ClO_4^- counter-anion) with insets showing the observed and simulated isotope patterns of the +5 peaks.

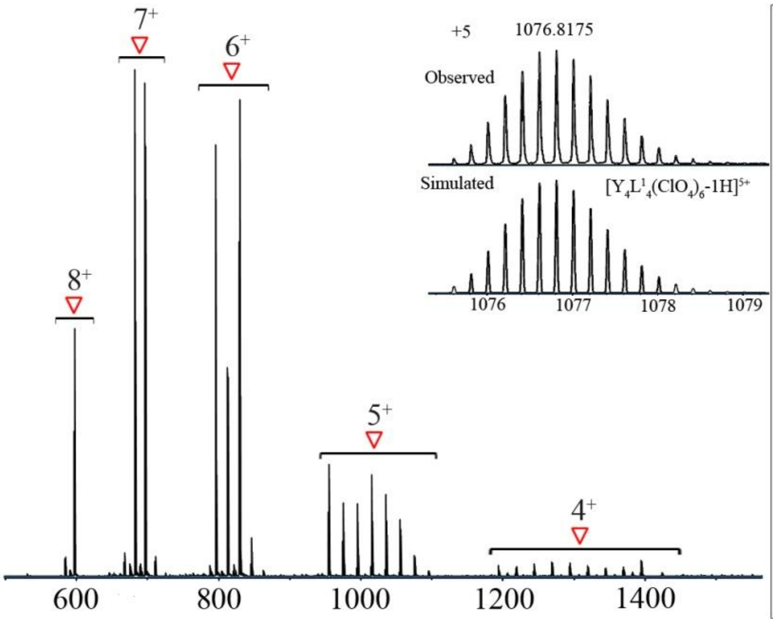

Observed	Simulated	Molecular formular
598.0470	598.0462	$[Y_4L^1_4\text{-}4H]^{8+}$
683.3384	683.3375	$[Y_4L^1_4\text{-}5H]^{7+}$
697.6175	697.6168	$[Y_4L^1_4(ClO_4)_1\text{-}4H]^{7+}$
797.0602	797.0592	$[Y_4L^1_4\text{-}6H]^{6+}$
813.7190	813.7183	$[Y_4L^1_4(ClO_4)_1\text{-}5H]^{6+}$
830.5454	830.5445	$[Y_4L^1_4(ClO_4)_2\text{-}4H]^{6+}$
956.2705	956.2696	$[Y_4L^1_4\text{-}7H]^{5+}$
976.2611	976.2606	$[Y_4L^1_4(ClO_4)_1\text{-}6H]^{5+}$
996.4527	996.4519	$[Y_4L^1_4(ClO_4)_2\text{-}5H]^{5+}$
1016.4438	1016.4430	$[Y_4L^1_4(ClO_4)_3\text{-}4H]^{5+}$
1036.6351	1036.6342	$[Y_4L^1_4(ClO_4)_4\text{-}3H]^{5+}$
1056.6264	1056.6253	$[Y_4L^1_4(ClO_4)_5\text{-}2H]^{5+}$
1076.8175	1076.8165	$[Y_4L^1_4(ClO_4)_6\text{-}1H]^{5+}$
1220.0745	1220.0739	$[Y_4L^1_4(ClO_4)_1\text{-}7H]^{4+}$
1245.3131	1245.3131	$[Y_4L^1_4(ClO_4)_2\text{-}6H]^{4+}$
1270.3020	1270.3019	$[Y_4L^1_4(ClO_4)_3\text{-}5H]^{4+}$

Supplementary Figure 250. ESI-TOF-MS spectrum for La^{III}-Y^{III} mixed-metal self-assembly of $\mathbf{L^1}$ (ClO_4^- counter-anion) with insets showing the observed and simulated isotope patterns of the +5 peaks.

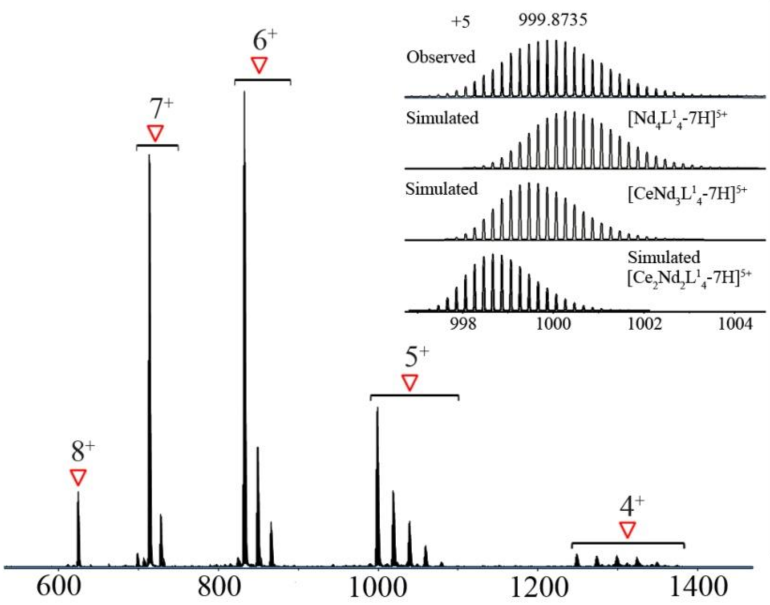

Observed	Simulated	Molecular formular
625.4240	625.5486	$[Nd_4L^1_4\text{-}4H]^{8+}$
714.4834	714.7688	$[Nd_4L^1_4\text{-}5H]^{7+}$
728.9056	729.1912	$[Nd_4L^1_4(ClO_4)_1\text{-}4H]^{7+}$
833.3960	833.7290	$[Nd_4L^1_4\text{-}6H]^{6+}$
850.2217	850.5551	$[Nd_4L^1_4(ClO_4)_1\text{-}5H]^{6+}$
867.0478	867.2143	$[Nd_4L^1_4(ClO_4)_2\text{-}4H]^{6+}$
999.8735	1000.2734	$[Nd_4L^1_4\text{-}7H]^{5+}$
1019.8644	1020.4647	$[Nd_4L^1_4(ClO_4)_1\text{-}6H]^{5+}$
1040.0554	1040.4557	$[Nd_4L^1_4(ClO_4)_2\text{-}5H]^{5+}$
1060.2469	1060.6470	$[Nd_4L^1_4(ClO_4)_3\text{-}4H]^{5+}$
1249.5890	1250.0899	$[Nd_4L^1_4\text{-}8H]^{4+}$
1274.8286	1275.3291	$[Nd_4L^1_4(ClO_4)_1\text{-}7H]^{4+}$
1299.5672	1300.3178	$[Nd_4L^1_4(ClO_4)_2\text{-}6H]^{4+}$
1324.8066	1325.5569	$[Nd_4L^1_4(ClO_4)_3\text{-}5H]^{4+}$

Supplementary Figure 251. ESI-TOF-MS spectrum for Ce^{III}-Nd^{III} mixed-metal self-assembly of $\mathbf{L^1}$ (ClO_4^- counter-anion) with insets showing the observed and simulated isotope patterns of the +5 peaks.

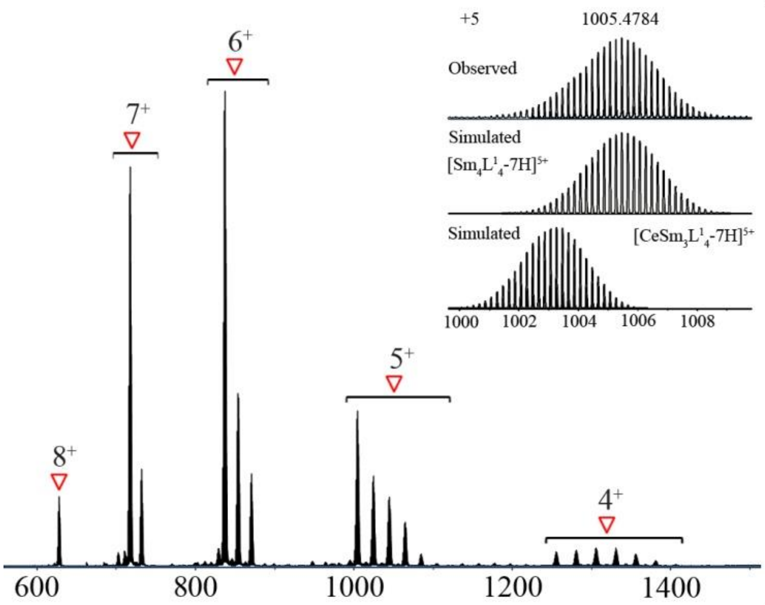

Observed	Simulated	Molecular formular
628.9272	628.8025	$[Sm_4L^1{}_4\text{-}4H]^{8+}$
718.4871	718.4875	$[Sm_4L^1{}_4\text{-}5H]^{7+}$
732.9094	732.9098	$[Sm_4L^1{}_4(ClO_4)_1\text{-}4H]^{7+}$
838.0669	838.0675	$[Sm_4L^1{}_4\text{-}6H]^{6+}$
854.7258	854.8936	$[Sm_4L^1{}_4(ClO_4)_1\text{-}5H]^{6+}$
871.7178	871.5528	$[Sm_4L^1{}_4(ClO_4)_2\text{-}4H]^{6+}$
1005.4784	1005.4796	$[Sm_4L^1{}_4\text{-}7H]^{5+}$
1025.6692	1025.6708	$[Sm_4L^1{}_4(ClO_4)_1\text{-}6H]^{5+}$
1045.8650	1045.6619	$[Sm_4L^1{}_4(ClO_4)_2\text{-}5H]^{5+}$
1065.6512	1065.4521	$[Sm_4L^1{}_4(ClO_4)_3\text{-}4H]^{5+}$
1085.6420	1085.8441	$[Sm_4L^1{}_4(ClO_4)_4\text{-}3H]^{5+}$
1256.5943	1256.5977	$[Sm_4L^1{}_4\text{-}8H]^{4+}$
1281.5834	1281.8367	$[Sm_4L^1{}_4(ClO_4)_1\text{-}7H]^{4+}$
1306.5722	1306.8255	$[Sm_4L^1{}_4(ClO_4)_2\text{-}6H]^{4+}$
1332.0616	1332.0646	$[Sm_4L^1{}_4(ClO_4)_3\text{-}5H]^{4+}$
1356.5494	1357.0534	$[Sm_4L^1{}_4(ClO_4)_4\text{-}4H]^{4+}$

Supplementary Figure 252. ESI-TOF-MS spectrum for Ce^{III}-Sm^{III} mixed-metal self-assembly of $\mathbf{L^1}$ (ClO_4^- counter-anion) with insets showing the observed and simulated isotope patterns of the +6 peaks.

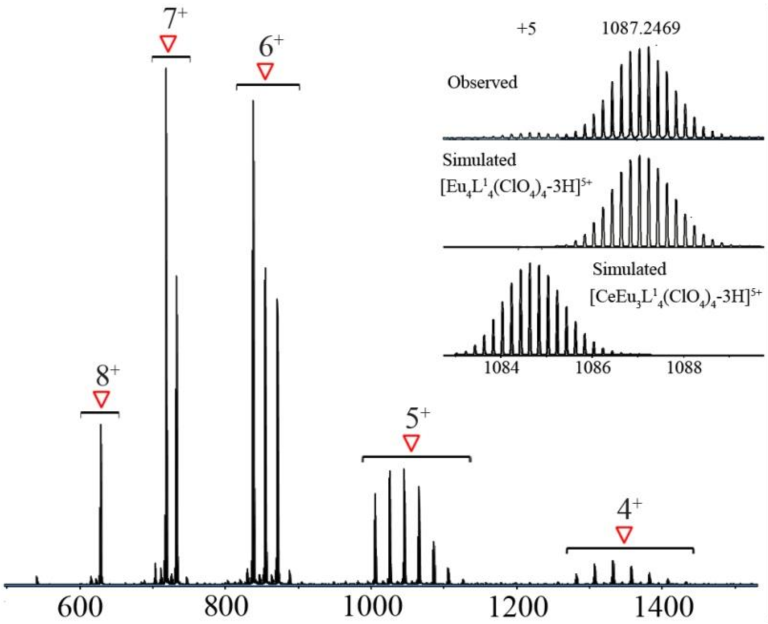

Observed	Simulated	Molecular formular
629.5545	629.5535	$[Eu_4L^1{}_4\text{-}4H]^{8+}$
719.3469	719.3459	$[Eu_4L^1{}_4\text{-}5H]^{7+}$
733.6259	733.7682	$[Eu_4L^1{}_4(ClO_4)1\text{-}4H]^{7+}$
839.0699	839.0690	$[Eu_4L^1{}_4\text{-}6H]^{6+}$
855.8959	855.8950	$[Eu_4L^1{}_4(ClO_4)_1\text{-}5H]^{6+}$
872.5549	872.5542	$[Eu_4L^1{}_4(ClO_4)_2\text{-}4H]^{6+}$
1006.6821	1006.6813	$[Eu_4L^1{}_4\text{-}7H]^{5+}$
1026.8732	1026.8726	$[Eu_4L^1{}_4(ClO_4)_1\text{-}6H]^{5+}$
1046.8642	1046.8636	$[Eu_4L^1{}_4(ClO_4)_2\text{-}5H]^{5+}$
1067.0555	1067.0549	$[Eu_4L^1{}_4(ClO_4)_3\text{-}4H]^{5+}$
1087.2469	1087.0459	$[Eu_4L^1{}_4(ClO_4)_4\text{-}3H]^{5+}$
1107.0379	1107.2372	$[Eu_4L^1{}_4(ClO_4)_5\text{-}2H]^{5+}$
1308.3278	1308.3277	$[Eu_4L^1{}_4(ClO_4)_2\text{-}6H]^{4+}$
1333.3162	1333.5668	$[Eu_4L^1{}_4(ClO_4)_3\text{-}5H]^{4+}$
1358.5554	1358.5556	$[Eu_4L^1{}_4(ClO_4)_4\text{-}4H]^{4+}$
1383.5443	1383.7946	$[Eu_4L^1{}_4(ClO_4)_5\text{-}3H]^{4+}$

Supplementary Figure 253. ESI-TOF-MS spectrum for Ce^{III}-Eu^{III} mixed-metal self-assembly of $\mathbf{L^1}$ (ClO_4^- counter-anion) with insets showing the observed and simulated isotope patterns of the +5 peaks.

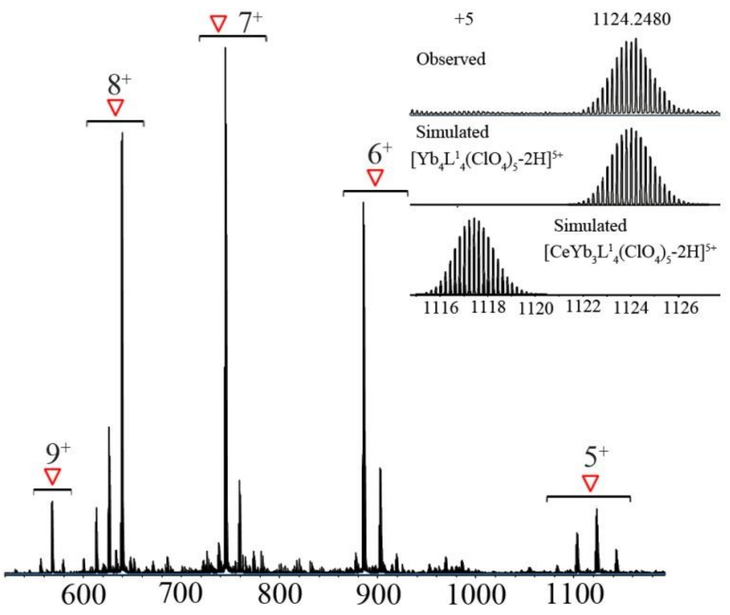

Observed	Simulated	Molecular formular
569.0550	569.0562	$[Yb_4L^1_4-3H]^{9+}$
640.1862	640.0623	$[Yb_4L^1_4-4H]^{8+}$
745.7766	745.7782	$[Yb_4L^1_4(ClO_4)_1-4H]^{7+}$
760.0550	760.0575	$[Yb_4L^1_4(ClO_4)_2-3H]^{7+}$
774.4773	774.4799	$[Yb_4L^1_4(ClO_4)_3-2H]^{7+}$
886.5641	886.5659	$[Yb_4L^1_4(ClO_4)_2-4H]^{6+}$
903.2226	903.5557	$[Yb_4L^1_4(ClO_4)_3-3H]^{6+}$
920.3814	920.0512	$[Yb_4L^1_4(ClO_4)_4-2H]^{6+}$
1104.0573	1103.8599	$[Yb_4L^1_4(ClO_4)_4-3H]^{5+}$
1124.2480	1124.0512	$[Yb_4L^1_4(ClO_4)_5-2H]^{5+}$
1144.0398	1144.0422	$[Yb_4L^1_4(ClO_4)_6-1H]^{5+}$

Supplementary Figure 254. ESI-TOF-MS spectrum for Ce^{III}-Yb^{III} mixed-metal self-assembly of $\mathbf{L^1}$ (ClO_4^- counter-anion) with insets showing the observed and simulated isotope patterns of the +5 peaks.

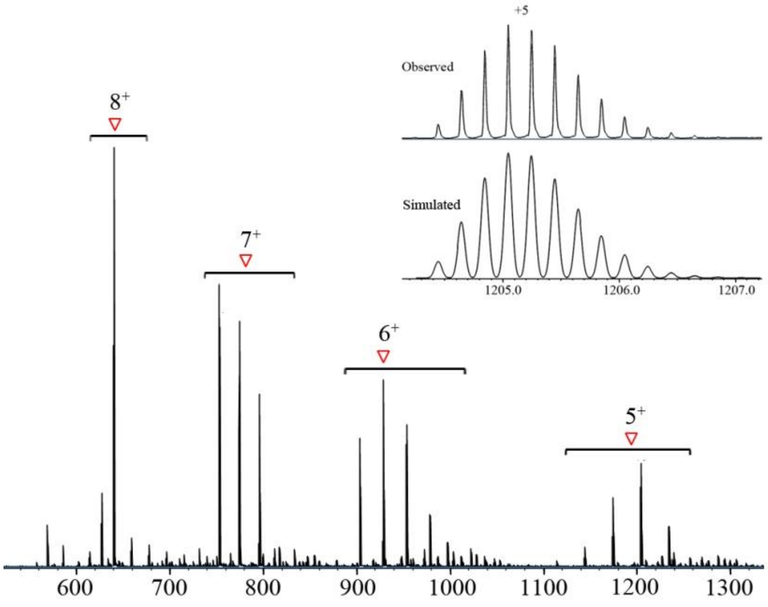

Observed	Simulated	Molecular formular
641.0643	641.0637	$[Lu_4L^1_4-4H]^{8+}$
753.9240	753.9231	$[Lu_4L^1_4(CF_3SO_3)_1-4H]^{7+}$
775.3468	775.3459	$[Lu_4L^1_4(CF_3SO_3)_2-3H]^{7+}$
796.7700	796.7687	$[Lu_4L^1_4(CF_3SO_3)_3-2H]^{7+}$
904.4039	904.4023	$[Lu_4L^1_4(CF_3SO_3)_2-4H]^{6+}$
929.3972	929.3956	$[Lu_4L^1_4(CF_3SO_3)_3-3H]^{6+}$
954.3904	954.3889	$[Lu_4L^1_4(CF_3SO_3)_4-2H]^{6+}$
979.3837	979.3821	$[Lu_4L^1_4(CF_3SO_3)_5-1H]^{6+}$
1145.0668	1145.0652	$[Lu_4L^1_4(CF_3SO_3)_4-3H]^{5+}$
1175.0592	1175.0571	$[Lu_4L^1_4(CF_3SO_3)_5-2H]^{5+}$
1205.0512	1205.0490	$[Lu_4L^1_4(CF_3SO_3)_6-1H]^{5+}$
1235.0431	1235.0410	$[Lu_4L^1_4(CF_3SO_3)_7]^{5+}$

Supplementary Figure 255. ESI-TOF-MS spectrum for Ce^{III}-Lu^{III} mixed-metal self-assembly of $\mathbf{L^1}$ ($CF_3SO_3^-$ counter-anion) with insets showing the observed and simulated isotope patterns of the +5 peaks.

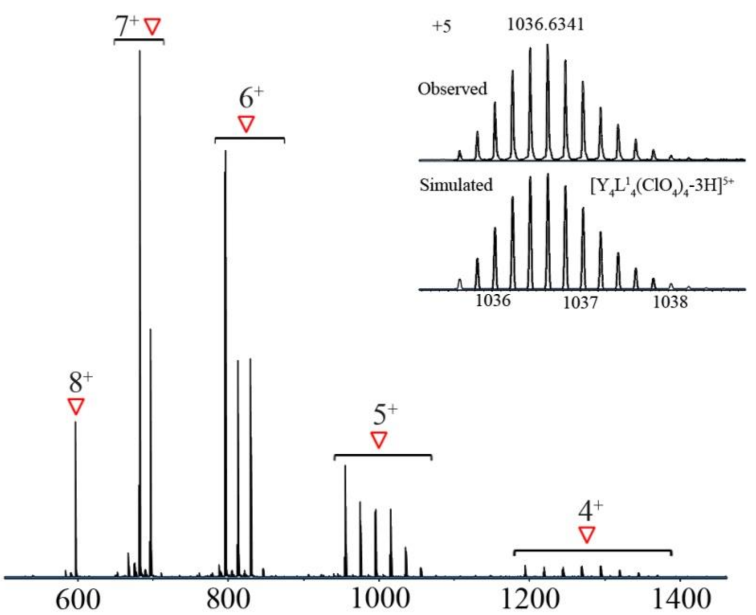

Observed	Simulated	Molecular formular
598.0464	598.0462	$[Y_4L^1_4\text{-}4H]^{8+}$
683.3378	683.3375	$[Y_4L^1_4\text{-}5H]^{7+}$
697.6165	697.6168	$[Y_4L^1_4(ClO_4)_1\text{-}4H]^{7+}$
797.0596	797.0592	$[Y_4L^1_4\text{-}6H]^{6+}$
813.7183	813.7183	$[Y_4L^1_4(ClO_4)_1\text{-}5H]^{6+}$
830.5443	830.5445	$[Y_4L^1_4(ClO_4)_2\text{-}4H]^{6+}$
956.2697	956.2696	$[Y_4L^1_4\text{-}7H]^{5+}$
976.2605	976.2606	$[Y_4L^1_4(ClO_4)_1\text{-}6H]^{5+}$
996.4518	996.4519	$[Y_4L^1_4(ClO_4)_2\text{-}5H]^{5+}$
1016.4428	1016.4430	$[Y_4L^1_4(ClO_4)_3\text{-}4H]^{5+}$
1036.6341	1036.6342	$[Y_4L^1_4(ClO_4)_4\text{-}3H]^{5+}$
1195.0856	1195.0852	$[Y_4L^1_4\text{-}8H]^{4+}$
1220.0734	1220.0739	$[Y_4L^1_4(ClO_4)_1\text{-}7H]^{4+}$
1245.0620	1245.3131	$[Y_4L^1_4(ClO_4)_2\text{-}6H]^{4+}$
1270.3013	1270.3019	$[Y_4L^1_4(ClO_4)_3\text{-}5H]^{4+}$
1295.5399	1295.5409	$[Y_4L^1_4(ClO_4)_4\text{-}4H]^{4+}$

Supplementary Figure 256. ESI-TOF-MS spectrum for Ce^{III}-Y^{III} mixed-metal self-assembly of $\mathbf{L^1}$ (ClO_4^- counter-anion) with insets showing the observed and simulated isotope patterns of the +6 peaks.

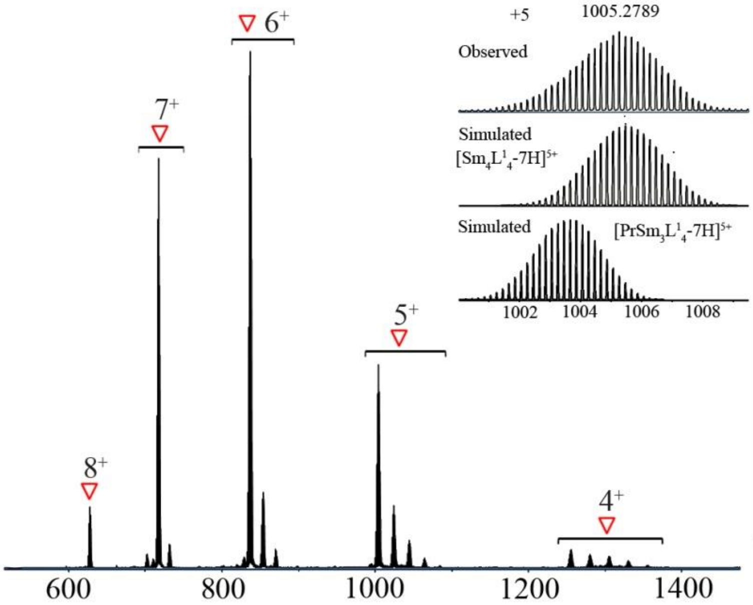

Observed	Simulated	Molecular formular
628.5525	628.8025	$[Sm_4L^1_4\text{-}4H]^{8+}$
718.3448	718.4875	$[Sm_4L^1_4\text{-}5H]^{7+}$
837.9008	838.0675	$[Sm_4L^1_4\text{-}6H]^{6+}$
854.8926	854.8936	$[Sm_4L^1_4(ClO_4)_1\text{-}5H]^{6+}$
871.3854	871.5528	$[Sm_4L^1_4(ClO_4)_2\text{-}4H]^{6+}$
1005.2789	1005.4796	$[Sm_4L^1_4\text{-}7H]^{5+}$
1025.2693	1025.6708	$[Sm_4L^1_4(ClO_4)_1\text{-}6H]^{5+}$
1045.4604	1045.6619	$[Sm_4L^1_4(ClO_4)_2\text{-}5H]^{5+}$
1065.6518	1065.4521	$[Sm_4L^1_4(ClO_4)_3\text{-}4H]^{5+}$
1256.5955	1256.5977	$[Sm_4L^1_4\text{-}8H]^{4+}$
1281.8358	1281.8367	$[Sm_4L^1_4(ClO_4)_1\text{-}7H]^{4+}$
1307.0550	1306.8255	$[Sm_4L^1_4(ClO_4)_2\text{-}6H]^{4+}$
1331.3131	1332.0646	$[Sm_4L^1_4(ClO_4)_3\text{-}5H]^{4+}$
1356.8013	1357.0534	$[Sm_4L^1_4(ClO_4)_4\text{-}4H]^{4+}$

Supplementary Figure 257. ESI-TOF-MS spectrum for Pr^{III}-Sm^{III} mixed-metal self-assembly of $\mathbf{L^1}$ (ClO_4^- counter-anion) with insets showing the observed and simulated isotope patterns of the +5 peaks.

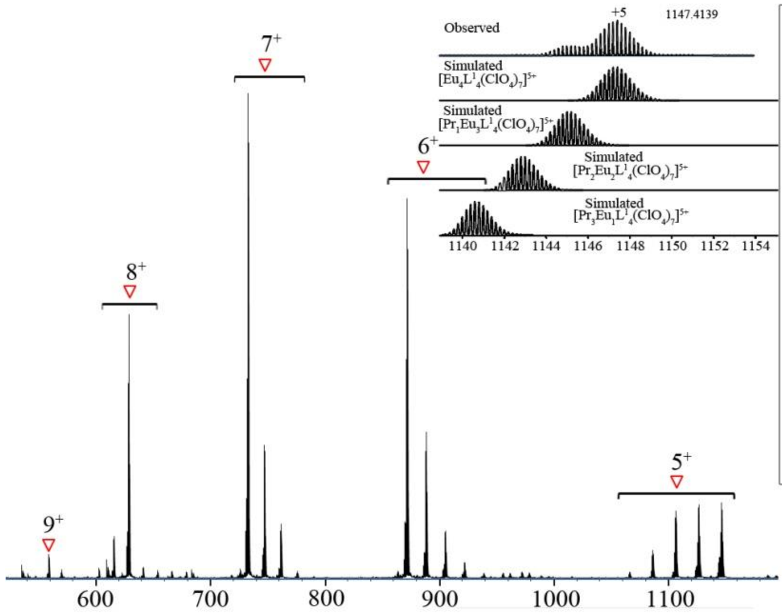

Observed	Simulated	Molecular formular
559.7126	559.7151	$[Eu_4L^1{}_4\text{-}3H]^{9+}$
629.5507	629.5535	$[Eu_4L^1{}_4\text{-}4H]^{8+}$
733.7649	733.7682	$[Eu_4L^1{}_4(ClO_4)_1\text{-}4H]^{7+}$
748.0438	748.0475	$[Eu_4L^1{}_4(ClO_4)_2\text{-}3H]^{7+}$
762.4658	762.4699	$[Eu_4L^1{}_4(ClO_4)_3\text{-}2H]^{7+}$
872.5504	872.5542	$[Eu_4L^1{}_4(ClO_4)_2\text{-}4H]^{6+}$
889.2093	889.3803	$[Eu_4L^1{}_4(ClO_4)_3\text{-}3H]^{6+}$
906.0351	906.0395	$[Eu_4L^1{}_4(ClO_4)_4\text{-}2H]^{6+}$
1087.0409	1087.0459	$[Eu_4L^1{}_4(ClO_4)_4\text{-}3H]^{5+}$
1107.2321	1107.2372	$[Eu_4L^1{}_4(ClO_4)_5\text{-}2H]^{5+}$
1127.2231	1127.2282	$[Eu_4L^1{}_4(ClO_4)_6\text{-}1H]^{5+}$
1147.4139	1147.4194	$[Eu_4L^1{}_4(ClO_4)_7]^{5+}$

Supplementary Figure 258. ESI-TOF-MS spectrum for Pr^{III}-Eu^{III} mixed-metal self-assembly of $\mathbf{L^1}$ (ClO_4^- counter-anion) with insets showing the observed and simulated isotope patterns of the +5 peaks.

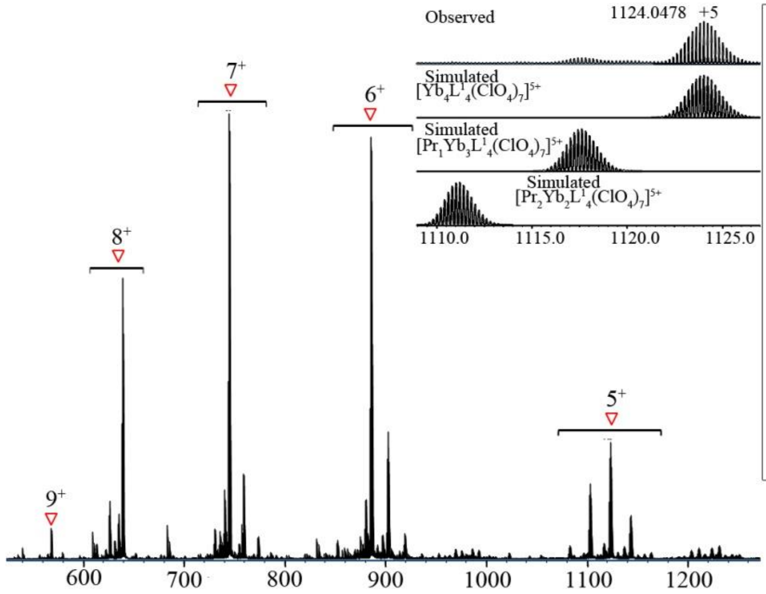

Observed	Simulated	Molecular formular
569.0559	569.0562	$[Yb_4L^1{}_4\text{-}3H]^{9+}$
640.1865	640.0623	$[Yb_4L^1{}_4\text{-}4H]^{8+}$
745.7768	745.7782	$[Yb_4L^1{}_4(ClO_4)_1\text{-}4H]^{7+}$
760.0558	760.0575	$[Yb_4L^1{}_4(ClO_4)_2\text{-}3H]^{7+}$
774.4782	774.4799	$[Yb_4L^1{}_4(ClO_4)_3\text{-}2H]^{7+}$
886.5645	886.5659	$[Yb_4L^1{}_4(ClO_4)_2\text{-}4H]^{6+}$
903.3899	903.3920	$[Yb_4L^1{}_4(ClO_4)_3\text{-}3H]^{6+}$
920.0489	920.0512	$[Yb_4L^1{}_4(ClO_4)_4\text{-}2H]^{6+}$
1103.8574	1103.8599	$[Yb_4L^1{}_4(ClO_4)_4\text{-}3H]^{5+}$
1124.0478	1124.0512	$[Yb_4L^1{}_4(ClO_4)_5\text{-}2H]^{5+}$
1144.0394	1144.0422	$[Yb_4L^1{}_4(ClO_4)_6\text{-}1H]^{5+}$
1164.4305	1164.2335	$[Yb_4L^1{}_4(ClO_4)_7]^{5+}$

Supplementary Figure 259. ESI-TOF-MS spectrum for Pr^{III}-Yb^{III} mixed-metal self-assembly of $\mathbf{L^1}$ (ClO_4^- counter-anion) with insets showing the observed and simulated isotope patterns of the +5 peaks.

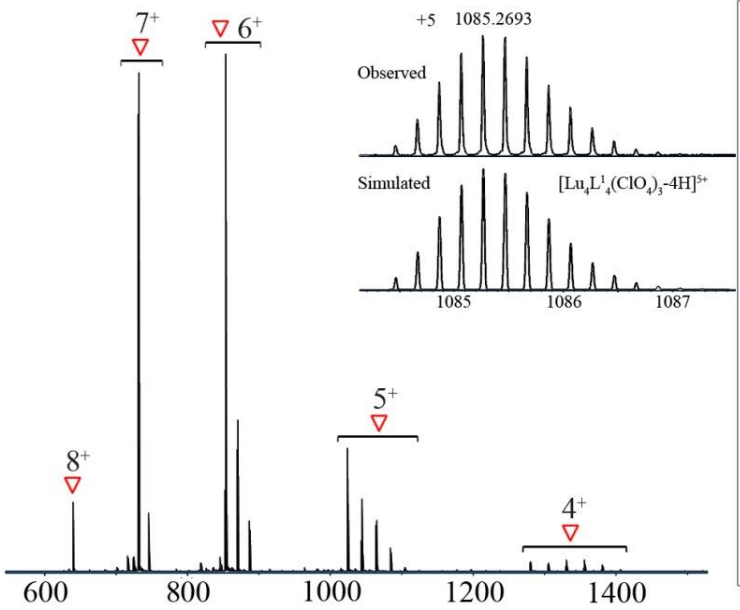

Observed	Simulated	Molecular formular
641.0633	641.0637	$[Lu_4L^1_4\text{-}4H]^{8+}$
732.4998	732.5003	$[Lu_4L^1_4\text{-}5H]^{7+}$
746.7784	746.7796	$[Lu_4L^1_4(ClO_4)_1\text{-}4H]^{7+}$
854.4152	854.4158	$[Lu_4L^1_4\text{-}6H]^{6+}$
871.0733	871.0750	$[Lu_4L^1_4(ClO_4)_1\text{-}5H]^{6+}$
887.8995	887.9011	$[Lu_4L^1_4(ClO_4)_2\text{-}4H]^{6+}$
1025.0962	1025.0975	$[Lu_4L^1_4\text{-}7H]^{5+}$
1045.0868	1045.0885	$[Lu_4L^1_4(ClO_4)_1\text{-}6H]^{5+}$
1065.2782	1065.2799	$[Lu_4L^1_4(ClO_4)_2\text{-}5H]^{5+}$
1085.2693	1085.2709	$[Lu_4L^1_4(ClO_4)_3\text{-}4H]^{5+}$
1281.1179	1281.1200	$[Lu_4L^1_4\text{-}8H]^{4+}$
1306.1059	1306.1088	$[Lu_4L^1_4(ClO_4)_1\text{-}7H]^{4+}$
1331.3450	1331.3480	$[Lu_4L^1_4(ClO_4)_2\text{-}6H]^{4+}$
1356.3336	1356.3368	$[Lu_4L^1_4(ClO_4)_3\text{-}5H]^{4+}$
1381.5722	1381.5759	$[Lu_4L^1_4(ClO_4)_4\text{-}4H]^{4+}$

Supplementary Figure 260. ESI-TOF-MS spectrum for Pr^{III}-Lu^{III} mixed-metal self-assembly of $\mathbf{L^1}$ (ClO_4^- counter-anion) with insets showing the observed and simulated isotope patterns of the +5 peaks.

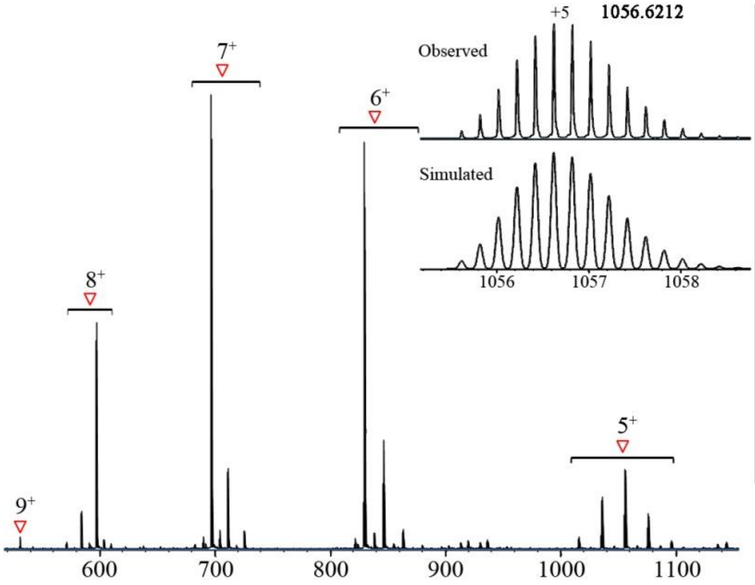

Observed	Simulated	Molecular formular
531.7069	531.7086	$[Y_4L^1_4\text{-}3H]^{9+}$
598.0439	598.0462	$[Y_4L^1_4\text{-}4H]^{8+}$
697.6146	697.6168	$[Y_4L^1_4(ClO_4)_1\text{-}4H]^{7+}$
712.0369	712.0392	$[Y_4L^1_4(ClO_4)_2\text{-}3H]^{7+}$
726.4588	726.3185	$[Y_4L^1_4(ClO_4)_3\text{-}2H]^{7+}$
830.5415	830.5445	$[Y_4L^1_4(ClO_4)_2\text{-}4H]^{6+}$
847.2005	847.2037	$[Y_4L^1_4(ClO_4)_3\text{-}3H]^{6+}$
864.0265	864.0297	$[Y_4L^1_4(ClO_4)_4\text{-}2H]^{6+}$
1016.4389	1016.4430	$[Y_4L^1_4(ClO_4)_3\text{-}4H]^{5+}$
1036.6303	1036.6342	$[Y_4L^1_4(ClO_4)_4\text{-}3H]^{5+}$
1056.6212	1056.6253	$[Y_4L^1_4(ClO_4)_5\text{-}2H]^{5+}$
1076.8122	1076.8165	$[Y_4L^1_4(ClO_4)_6\text{-}1H]^{5+}$

Supplementary Figure 261. ESI-TOF-MS spectrum for Pr^{III}-Y^{III} mixed-metal self-assembly of $\mathbf{L^1}$ (ClO_4^- counter-anion) with insets showing the observed and simulated isotope patterns of the +5 peaks.

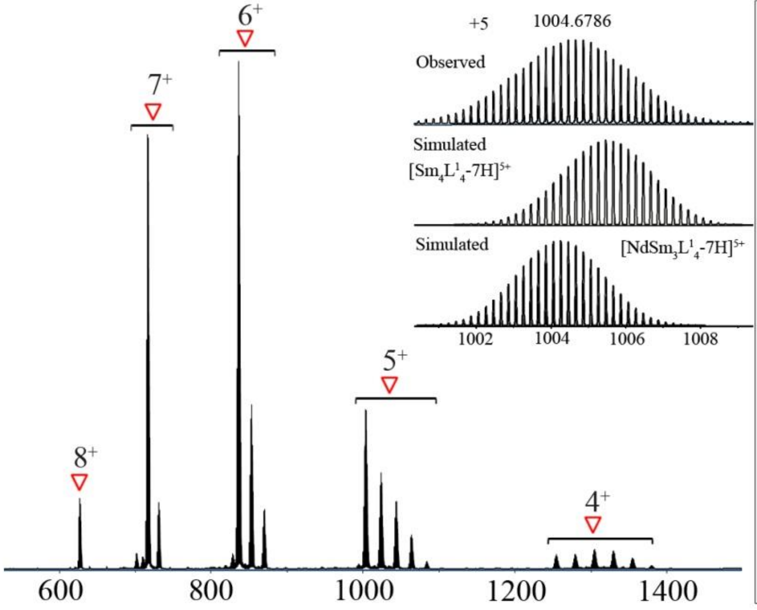

Observed	Simulated	Molecular formular
628.1770	628.8025	[Sm4L14-4H]8+
717.7727	718.4875	[Sm4L14-5H]7+
732.1954	732.9098	[Sm4L14(ClO4)1-4H]7+
837.2338	838.0675	[Sm4L14-6H]6+
853.8924	854.8936	[Sm4L14(ClO4)1-5H]6+
871.2189	871.5528	[Sm4L14(ClO4)2-4H]6+
1004.6786	1005.4796	[Sm4L14-7H]5+
1024.6697	1025.6708	[Sm4L14(ClO4)1-6H]5+
1045.0610	1045.6619	[Sm4L14(ClO4)2-5H]5+
1064.6512	1065.4521	[Sm4L14(ClO4)3-4H]5+
1255.5958	1256.5977	[Sm4L14-8H]4+
1280.0840	1281.8367	[Sm4L14(ClO4)1-7H]4+
1305.8241	1306.8255	[Sm4L14(ClO4)2-6H]4+
1331.0622	1332.0646	[Sm4L14(ClO4)3-5H]4+
1355.7998	1357.0534	[Sm4L14(ClO4)4-4H]4+
1381.0391	1382.2924	[Sm4L14(ClO4)5-3H]4+

Supplementary Figure 262. ESI-TOF-MS spectrum for Nd^{III}-Sm^{III} mixed-metal self-assembly of $\mathbf{L^1}$ (ClO_4^- counter-anion) with insets showing the observed and simulated isotope patterns of the +5 peaks.

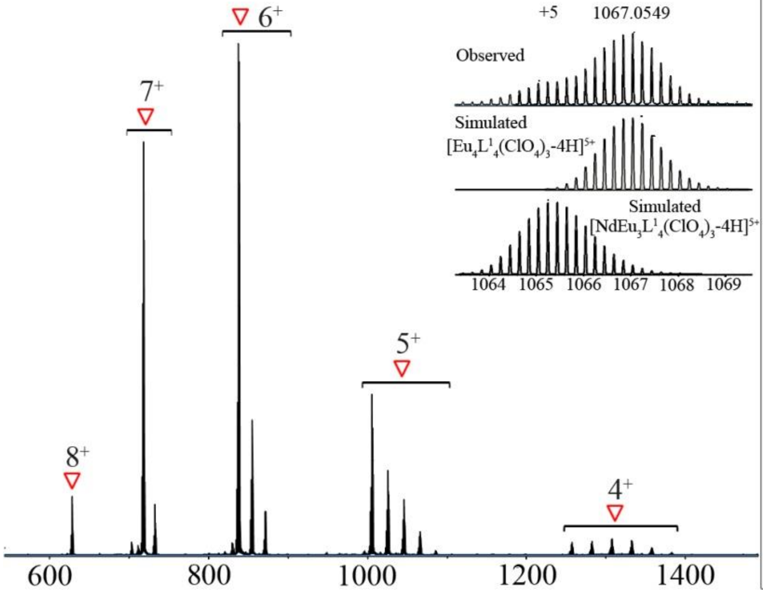

Observed	Simulated	Molecular formular
629.5544	629.5535	$[Eu_4L^1_4\text{-}4H]^{8+}$
719.3469	719.3459	$[Eu_4L^1_4\text{-}5H]^{7+}$
733.7688	733.7682	$[Eu_4L^1_4(ClO_4)_1\text{-}4H]^{7+}$
839.0698	839.0690	$[Eu_4L^1_4\text{-}6H]^{6+}$
855.8951	855.8950	$[Eu_4L^1_4(ClO_4)_1\text{-}5H]^{6+}$
872.5548	872.5542	$[Eu_4L^1_4(ClO_4)_2\text{-}4H]^{6+}$
1006.6818	1006.6813	$[Eu_4L^1_4\text{-}7H]^{5+}$
1026.8729	1026.8726	$[Eu_4L^1_4(ClO_4)_1\text{-}6H]^{5+}$
1046.8638	1046.8636	$[Eu_4L^1_4(ClO_4)_2\text{-}5H]^{5+}$
1067.0549	1067.0549	$[Eu_4L^1_4(ClO_4)_3\text{-}4H]^{5+}$
1257.8489	1258.0998	$[Eu_4L^1_4\text{-}8H]^{4+}$
1283.3380	1283.3389	$[Eu_4L^1_4(ClO_4)_1\text{-}7H]^{4+}$
1308.3273	1308.3277	$[Eu_4L^1_4(ClO_4)_2\text{-}6H]^{4+}$
1333.3161	1333.5668	$[Eu_4L^1_4(ClO_4)_3\text{-}5H]^{4+}$
1358.5550	1358.5556	$[Eu_4L^1_4(ClO_4)_4\text{-}4H]^{4+}$
1383.7933	1383.7946	$[Eu_4L^1_4(ClO_4)_5\text{-}3H]^{4+}$

Supplementary Figure 263. ESI-TOF-MS spectrum for Nd^{III}-Eu^{III} mixed-metal self-assembly of $\mathbf{L^1}$ (ClO_4^- counter-anion) with insets showing the observed and simulated isotope patterns of the +5 peaks.

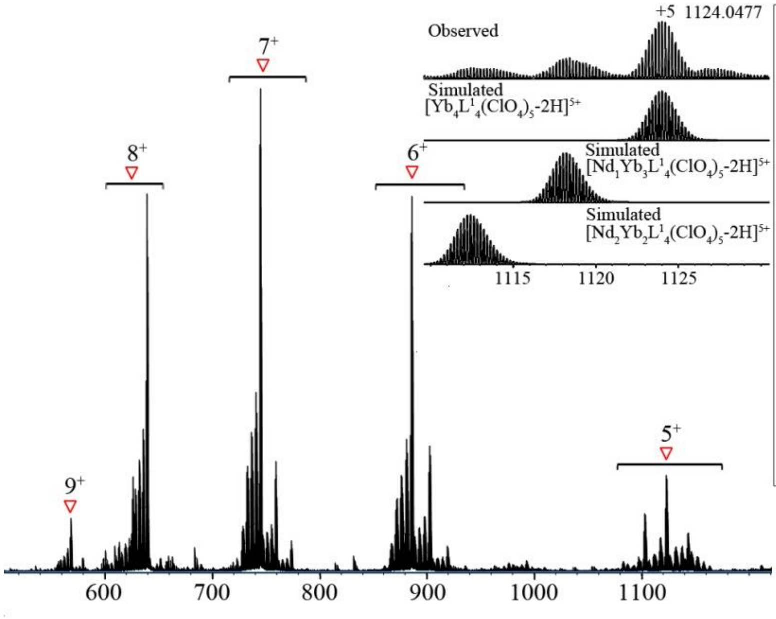

Observed	Simulated	Molecular formular
569.1659	569.0562	$[Yb_4L^1{}_4\text{-}3H]^{9+}$
640.1856	640.0623	$[Yb_4L^1{}_4\text{-}4H]^{8+}$
745.7760	745.7782	$[Yb_4L^1{}_4(ClO_4)_1\text{-}4H]^{7+}$
760.1990	760.0575	$[Yb_4L^1{}_4(ClO_4)_2\text{-}3H]^{7+}$
774.4774	774.4799	$[Yb_4L^1{}_4(ClO_4)_3\text{-}2H]^{7+}$
886.5635	886.5659	$[Yb_4L^1{}_4(ClO_4)_2\text{-}4H]^{6+}$
903.5559	903.3920	$[Yb_4L^1{}_4(ClO_4)_3\text{-}3H]^{6+}$
919.8809	920.0512	$[Yb_4L^1{}_4(ClO_4)_4\text{-}2H]^{6+}$
1103.8555	1103.8599	$[Yb_4L^1{}_4(ClO_4)_4\text{-}3H]^{5+}$
1124.0477	1124.0512	$[Yb_4L^1{}_4(ClO_4)_5\text{-}2H]^{5+}$
1144.4381	1144.0422	$[Yb_4L^1{}_4(ClO_4)_6\text{-}1H]^{5+}$
1164.2317	1164.2335	$[Yb_4L^1{}_4(ClO_4)_7]^{5+}$

Supplementary Figure 264. ESI-TOF-MS spectrum for Nd^{III}-Yb^{III} mixed-metal self-assembly of $\mathbf{L^1}$ (ClO_4^- counter-anion) with insets showing the observed and simulated isotope patterns of the +5 peaks.

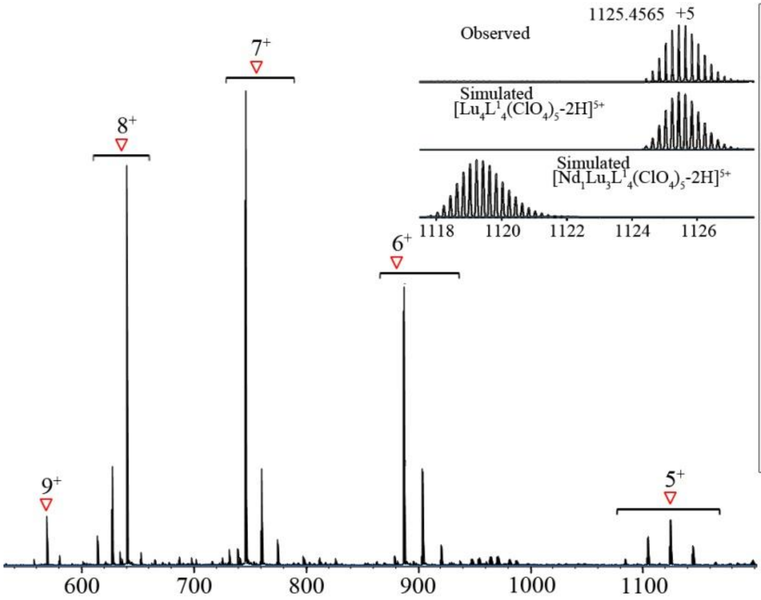

Observed	Simulated	Molecular formular
569.9501	569.9463	$[Lu_4L^1{}_4\text{-}3H]^{9+}$
641.0674	641.0637	$[Lu_4L^1{}_4\text{-}4H]^{8+}$
746.7830	746.7796	$[Lu_4L^1{}_4(ClO_4)_1\text{-}4H]^{7+}$
761.2052	761.2020	$[Lu_4L^1{}_4(ClO_4)_2\text{-}3H]^{7+}$
775.4845	775.4813	$[Lu_4L^1{}_4(ClO_4)_3\text{-}2H]^{7+}$
887.9047	887.9011	$[Lu_4L^1{}_4(ClO_4)_2\text{-}4H]^{6+}$
904.5638	904.5603	$[Lu_4L^1{}_4(ClO_4)_3\text{-}3H]^{6+}$
921.3896	921.3863	$[Lu_4L^1{}_4(ClO_4)_4\text{-}2H]^{6+}$
1105.4653	1105.4622	$[Lu_4L^1{}_4(ClO_4)_4\text{-}3H]^{5+}$
1125.4565	1125.4532	$[Lu_4L^1{}_4(ClO_4)_5\text{-}2H]^{5+}$
1145.6474	1145.6445	$[Lu_4L^1{}_4(ClO_4)_6\text{-}1H]^{5+}$
1165.6380	1165.6356	$[Lu_4L^1{}_4(ClO_4)_7]^{5+}$

Supplementary Figure 265. ESI-TOF-MS spectrum for Nd^{III}-Lu^{III} mixed-metal self-assembly of $\mathbf{L^1}$ (ClO_4^- counter-anion) with insets showing the observed and simulated isotope patterns of the +5 peaks.

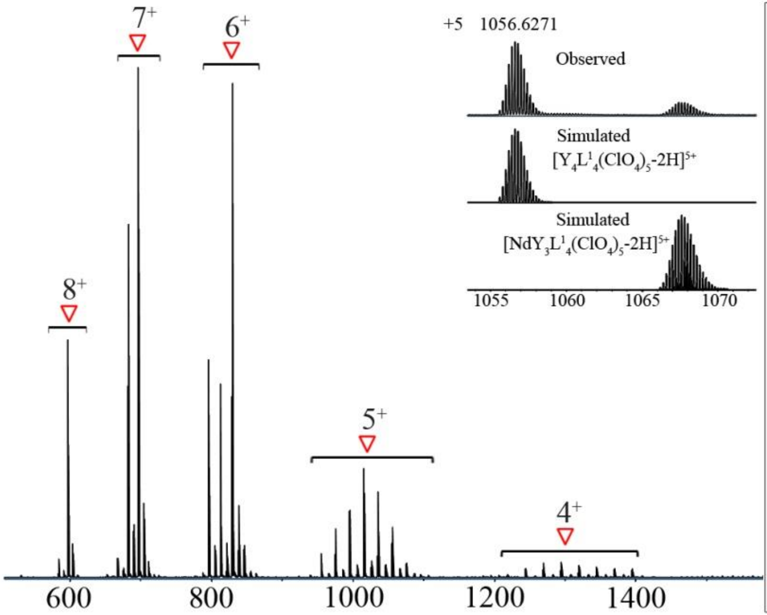

Observed	Simulated	Molecular formular
598.0480	598.0462	$[Y_4L^1_4\text{-}4H]^{8+}$
683.3392	683.3375	$[Y_4L^1_4\text{-}5H]^{7+}$
697.6183	697.6168	$[Y_4L^1_4(ClO_4)_1\text{-}4H]^{7+}$
797.0609	797.0592	$[Y_4L^1_4\text{-}6H]^{6+}$
813.7198	813.7183	$[Y_4L^1_4(ClO_4)_1\text{-}5H]^{6+}$
830.5461	830.5445	$[Y_4L^1_4(ClO_4)_2\text{-}4H]^{6+}$
976.2622	976.2606	$[Y_4L^1_4(ClO_4)_1\text{-}6H]^{5+}$
996.4534	996.4519	$[Y_4L^1_4(ClO_4)_2\text{-}5H]^{5+}$
1016.4447	1016.4430	$[Y_4L^1_4(ClO_4)_3\text{-}4H]^{5+}$
1036.6361	1036.6342	$[Y_4L^1_4(ClO_4)_4\text{-}3H]^{5+}$
1056.6271	1056.6253	$[Y_4L^1_4(ClO_4)_5\text{-}2H]^{5+}$
1076.8174	1076.8165	$[Y_4L^1_4(ClO_4)_6\text{-}1H]^{5+}$
1245.3134	1245.3131	$[Y_4L^1_4(ClO_4)_2\text{-}6H]^{4+}$
1270.3027	1270.3019	$[Y_4L^1_4(ClO_4)_3\text{-}5H]^{4+}$
1295.5419	1295.5409	$[Y_4L^1_4(ClO_4)_4\text{-}4H]^{4+}$
1320.5313	1320.5298	$[Y_4L^1_4(ClO_4)_5\text{-}3H]^{4+}$

Supplementary Figure 266. ESI-TOF-MS spectrum for Nd^{III}-Y^{III} mixed-metal self-assembly of $\mathbf{L^1}$ (ClO_4^- counter-anion) with insets showing the observed and simulated isotope patterns of the +5 peaks.

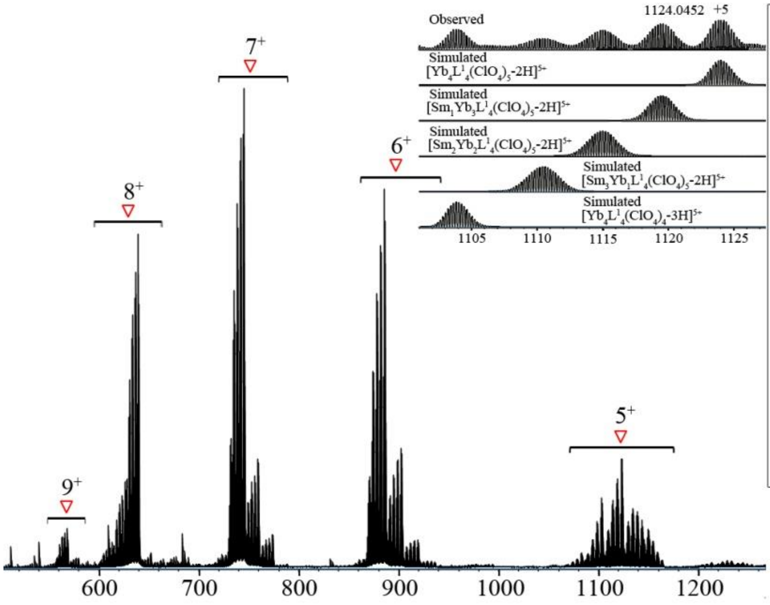

Observed	Simulated	Molecular formular
569.0535	569.0562	$[Yb_4L^1_4\text{-}3H]^{9+}$
640.0593	640.0623	$[Yb_4L^1_4\text{-}4H]^{8+}$
745.7745	745.7782	$[Yb_4L^1_4(ClO_4)_1\text{-}4H]^{7+}$
760.0507	760.0575	$[Yb_4L^1_4(ClO_4)_2\text{-}3H]^{7+}$
774.4756	774.4799	$[Yb_4L^1_4(ClO_4)_3\text{-}2H]^{7+}$
886.5618	886.5659	$[Yb_4L^1_4(ClO_4)_2\text{-}4H]^{6+}$
903.3872	903.3920	$[Yb_4L^1_4(ClO_4)_3\text{-}3H]^{6+}$
920.0462	920.0512	$[Yb_4L^1_4(ClO_4)_4\text{-}2H]^{6+}$
1103.8538	1103.8599	$[Yb_4L^1_4(ClO_4)_4\text{-}3H]^{5+}$
1124.0452	1124.0512	$[Yb_4L^1_4(ClO_4)_5\text{-}2H]^{5+}$
1144.0346	1144.0422	$[Yb_4L^1_4(ClO_4)_6\text{-}1H]^{5+}$
1164.4276	1164.2335	$[Yb_4L^1_4(ClO_4)_7]^{5+}$

Supplementary Figure 267. ESI-TOF-MS spectrum for Sm^{III}-Yb^{III} mixed-metal self-assembly of $\mathbf{L^1}$ (ClO_4^- counter-anion) with insets showing the observed and simulated isotope patterns of the +5 peaks.

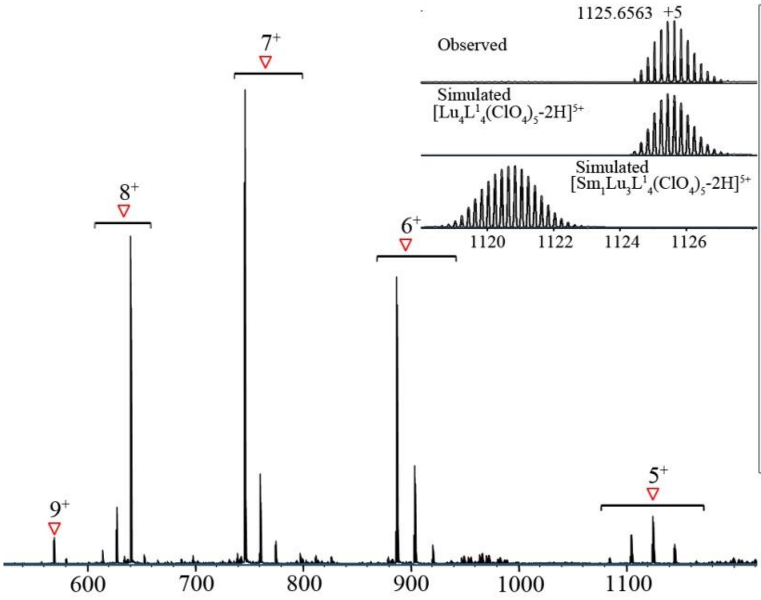

Observed	Simulated	Molecular formular
569.9499	569.9463	$[Lu_4L^1_4\text{-}3H]^{9+}$
641.0669	641.0637	$[Lu_4L^1_4\text{-}4H]^{8+}$
746.7827	746.7796	$[Lu_4L^1_4(ClO_4)_1\text{-}4H]^{7+}$
761.2048	761.2020	$[Lu_4L^1_4(ClO_4)_2\text{-}3H]^{7+}$
775.4842	775.4813	$[Lu_4L^1_4(ClO_4)_3\text{-}2H]^{7+}$
887.9043	887.9011	$[Lu_4L^1_4(ClO_4)_2\text{-}4H]^{6+}$
904.5635	904.5603	$[Lu_4L^1_4(ClO_4)_3\text{-}3H]^{6+}$
921.3894	921.3863	$[Lu_4L^1_4(ClO_4)_4\text{-}2H]^{6+}$
1105.4651	1105.4622	$[Lu_4L^1_4(ClO_4)_4\text{-}3H]^{5+}$
1125.6563	1125.4532	$[Lu_4L^1_4(ClO_4)_5\text{-}2H]^{5+}$
1145.6472	1145.6445	$[Lu_4L^1_4(ClO_4)_6\text{-}1H]^{5+}$
1165.6388	1165.6356	$[Lu_4L^1_4(ClO_4)_7]^{5+}$

Supplementary Figure 268. ESI-TOF-MS spectrum for Sm^{III}-Lu^{III} mixed-metal self-assembly of $\mathbf{L^1}$ (ClO_4^- counter-anion) with insets showing the observed and simulated isotope patterns of the +5 peaks.

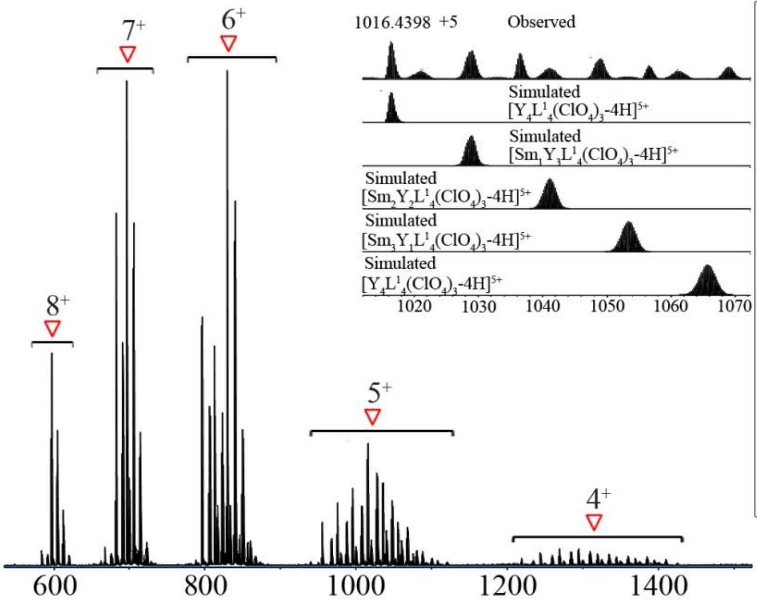

Observed	Simulated	Molecular formular
531.7063	531.7086	$[Y_4L^1_4\text{-}3H]^{9+}$
598.0438	598.0462	$[Y_4L^1_4\text{-}4H]^{8+}$
697.6145	697.6168	$[Y_4L^1_4(ClO_4)_1\text{-}4H]^{7+}$
712.0365	712.0392	$[Y_4L^1_4(ClO_4)_2\text{-}3H]^{7+}$
726.3157	726.3185	$[Y_4L^1_4(ClO_4)_3\text{-}2H]^{7+}$
830.5413	830.5445	$[Y_4L^1_4(ClO_4)_2\text{-}4H]^{6+}$
847.2001	847.2037	$[Y_4L^1_4(ClO_4)_3\text{-}3H]^{6+}$
864.0259	864.0297	$[Y_4L^1_4(ClO_4)_4\text{-}2H]^{6+}$
1016.4398	1016.4430	$[Y_4L^1_4(ClO_4)_3\text{-}4H]^{5+}$
1036.6294	1036.6342	$[Y_4L^1_4(ClO_4)_4\text{-}3H]^{5+}$
1056.6205	1056.6253	$[Y_4L^1_4(ClO_4)_5\text{-}2H]^{5+}$
1076.8113	1076.8165	$[Y_4L^1_4(ClO_4)_6\text{-}1H]^{5+}$
1096.8021	1096.8076	$[Y_4L^1_4(ClO_4)_7]^{5+}$

Supplementary Figure 269. ESI-TOF-MS spectrum for Sm^{III}-Y^{III} mixed-metal self-assembly of $\mathbf{L^1}$ (ClO_4^- counter-anion) with insets showing the observed and simulated isotope patterns of the +5 peaks.

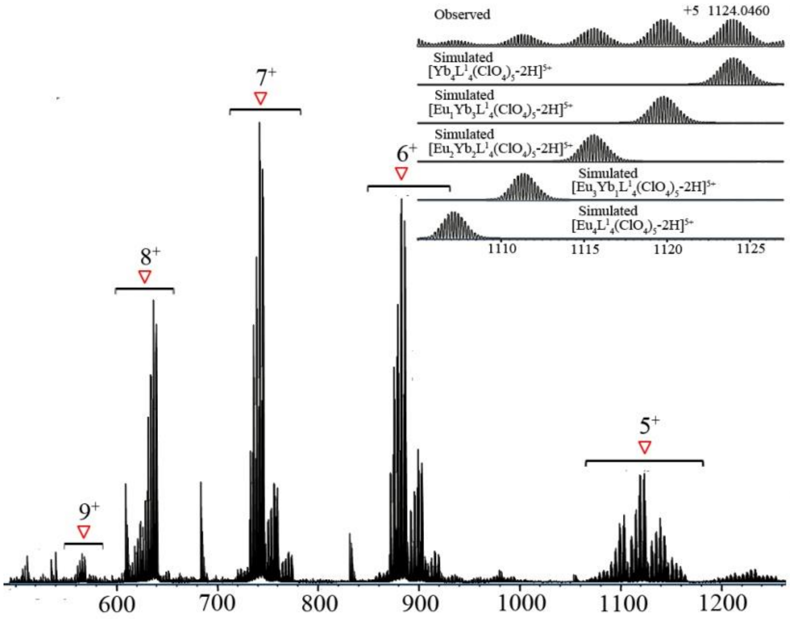

Observed	Simulated	Molecular formular
569.0533	569.0562	$[Yb_4L^1_4\text{-}3H]^{9+}$
640.0593	640.0623	$[Yb_4L^1_4\text{-}4H]^{8+}$
745.7749	745.7782	$[Yb_4L^1_4(ClO_4)_1\text{-}4H]^{7+}$
760.0537	760.0575	$[Yb_4L^1_4(ClO_4)_2\text{-}3H]^{7+}$
774.4759	774.4799	$[Yb_4L^1_4(ClO_4)_3\text{-}2H]^{7+}$
886.5621	886.5659	$[Yb_4L^1_4(ClO_4)_2\text{-}4H]^{6+}$
903.3878	903.3920	$[Yb_4L^1_4(ClO_4)_3\text{-}3H]^{6+}$
920.0468	920.0512	$[Yb_4L^1_4(ClO_4)_4\text{-}2H]^{6+}$
1103.8544	1103.8599	$[Yb_4L^1_4(ClO_4)_4\text{-}3H]^{5+}$
1124.0460	1124.0512	$[Yb_4L^1_4(ClO_4)_5\text{-}2H]^{5+}$
1144.0368	1144.0422	$[Yb_4L^1_4(ClO_4)_6\text{-}1H]^{5+}$
1164.0275	1164.2335	$[Yb_4L^1_4(ClO_4)_7]^{5+}$

Supplementary Figure 270. ESI-TOF-MS spectrum for Eu^{III}-Yb^{III} mixed-metal self-assembly of $\mathbf{L^1}$ (ClO_4^- counter-anion) with insets showing the observed and simulated isotope patterns of the +5 peaks.

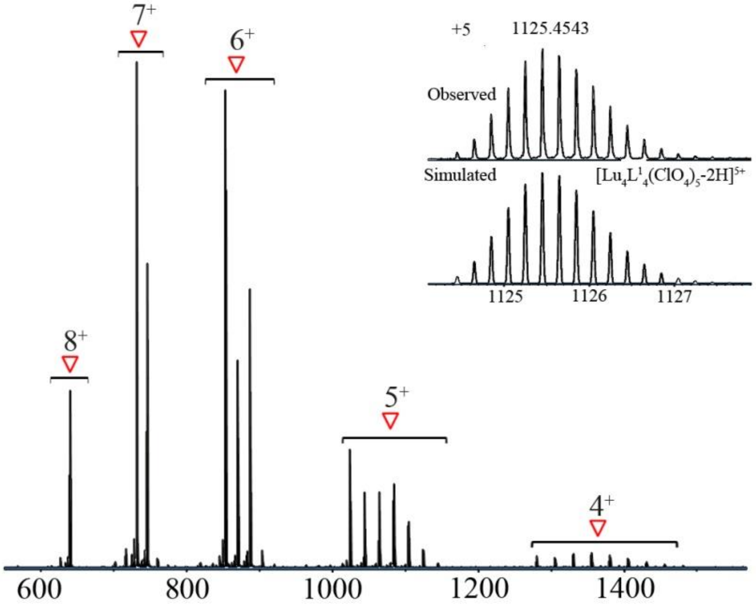

Observed	Simulated	Molecular formular
641.0649	641.0637	$[Lu_4L^1_4\text{-}4H]^{8+}$
732.5015	732.5003	$[Lu_4L^1_4\text{-}5H]^{7+}$
746.7805	746.7796	$[Lu_4L^1_4(ClO_4)_1\text{-}4H]^{7+}$
854.4171	854.4158	$[Lu_4L^1_4\text{-}6H]^{6+}$
871.0758	871.0750	$[Lu_4L^1_4(ClO_4)_1\text{-}5H]^{6+}$
887.9019	887.9011	$[Lu_4L^1_4(ClO_4)_2\text{-}4H]^{6+}$
1045.0892	1045.0885	$[Lu_4L^1_4(ClO_4)_1\text{-}6H]^{5+}$
1065.2807	1065.2799	$[Lu_4L^1_4(ClO_4)_2\text{-}5H]^{5+}$
1085.2720	1085.2709	$[Lu_4L^1_4(ClO_4)_3\text{-}4H]^{5+}$
1105.4631	1105.4622	$[Lu_4L^1_4(ClO_4)_4\text{-}3H]^{5+}$
1125.4543	1125.4532	$[Lu_4L^1_4(ClO_4)_5\text{-}2H]^{5+}$
1306.1092	1306.1088	$[Lu_4L^1_4(ClO_4)_1\text{-}7H]^{4+}$
1331.3481	1331.3480	$[Lu_4L^1_4(ClO_4)_2\text{-}6H]^{4+}$
1356.3372	1356.3368	$[Lu_4L^1_4(ClO_4)_3\text{-}5H]^{4+}$
1381.5761	1381.5759	$[Lu_4L^1_4(ClO_4)_4\text{-}4H]^{4+}$
1406.5651	1406.5647	$[Lu_4L^1_4(ClO_4)_5\text{-}3H]^{4+}$

Supplementary Figure 271. ESI-TOF-MS spectrum for Eu^{III}-Lu^{III} mixed-metal self-assembly of $\mathbf{L^1}$ (ClO_4^- counter-anion) with insets showing the observed and simulated isotope patterns of the +5 peaks.

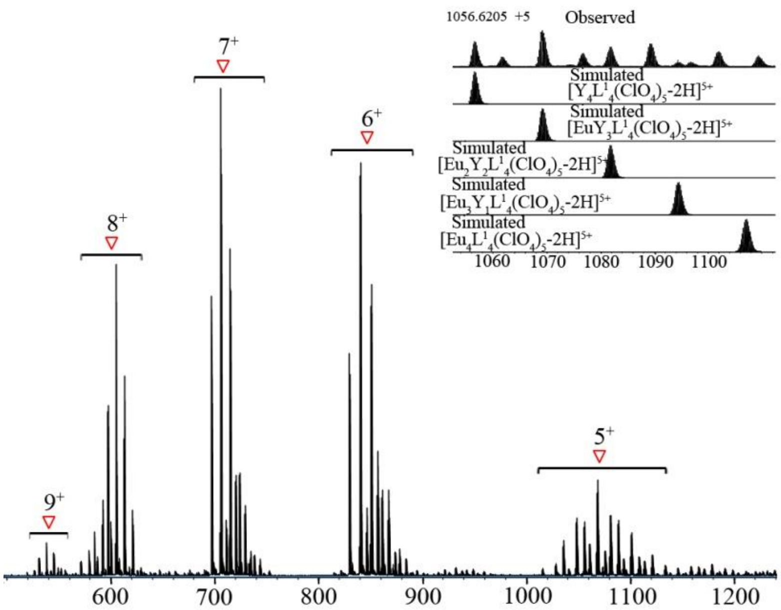

Observed	Simulated	Molecular formular
531.7063	531.7086	$[Y_4L^1_4\text{-}3H]^{9+}$
598.0438	598.0462	$[Y_4L^1_4\text{-}4H]^{8+}$
697.6145	697.6168	$[Y_4L^1_4(ClO_4)_1\text{-}4H]^{7+}$
712.0365	712.0392	$[Y_4L^1_4(ClO_4)_2\text{-}3H]^{7+}$
726.3157	726.3185	$[Y_4L^1_4(ClO_4)_3\text{-}2H]^{7+}$
830.5413	830.5445	$[Y_4L^1_4(ClO_4)_2\text{-}4H]^{6+}$
847.2001	847.2037	$[Y_4L^1_4(ClO_4)_3\text{-}3H]^{6+}$
864.0259	864.0297	$[Y_4L^1_4(ClO_4)_4\text{-}2H]^{6+}$
1016.4381	1016.4430	$[Y_4L^1_4(ClO_4)_3\text{-}4H]^{5+}$
1036.6294	1036.6342	$[Y_4L^1_4(ClO_4)_4\text{-}3H]^{5+}$
1056.6205	1056.6253	$[Y_4L^1_4(ClO_4)_5\text{-}2H]^{5+}$
1076.8113	1076.8165	$[Y_4L^1_4(ClO_4)_6\text{-}1H]^{5+}$
1096.8021	1096.8076	$[Y_4L^1_4(ClO_4)_7]^{5+}$

Supplementary Figure 272. ESI-TOF-MS spectrum for Eu^{III}-Y^{III} mixed-metal self-assembly of $\mathbf{L^1}$(ClO_4^- counter-anion) with insets showing the observed and simulated isotope patterns of the +5 peaks

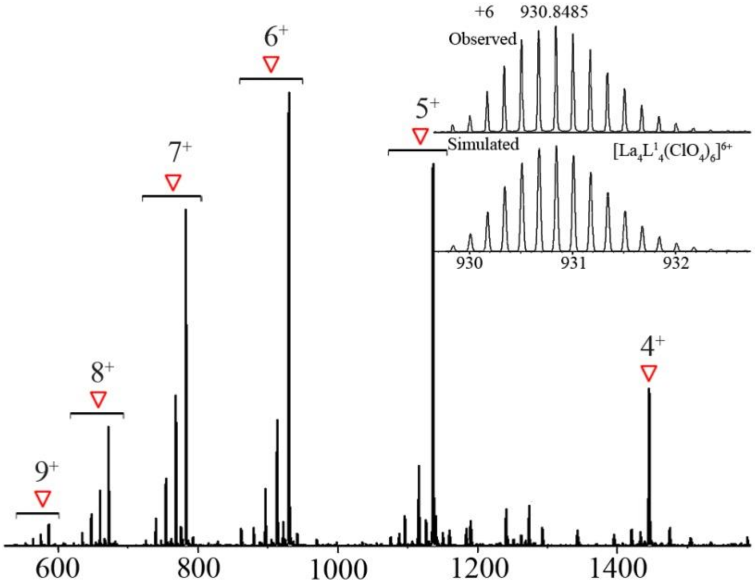

Observed	Simulated	Molecular formular
576.2553	576.2545	$[La_4L^1_4(ClO_4)_2\text{-}1H]^{9+}$
587.4718	587.3607	$[La_4L^1_4(ClO_4)_3]^{9+}$
648.1618	648.1604	$[La_4L^1_4(ClO_4)_2\text{-}2H]^{8+}$
660.7805	660.6548	$[La_4L^1_4(ClO_4)_3\text{-}1H]^{8+}$
673.2749	673.2744	$[La_4L^1_4(ClO_4)_4]^{8+}$
740.6122	740.6109	$[La_4L^1_4(ClO_4)_2\text{-}3H]^{7+}$
755.0340	754.8902	$[La_4L^1_4(ClO_4)_3\text{-}2H]^{7+}$
769.3128	769.3125	$[La_4L^1_4(ClO_4)_4\text{-}1H]^{7+}$
783.5919	783.5919	$[La_4L^1_4(ClO_4)_5]^{7+}$
897.1972	897.3634	$[La_4L^1_4(ClO_4)_4\text{-}2H]^{6+}$
914.1895	914.0226	$[La_4L^1_4(ClO_4)_5\text{-}1H]^{6+}$
930.8485	930.8486	$[La_4L^1_4(ClO_4)_6]^{6+}$
1116.8167	1116.8169	$[La_4L^1_4(ClO_4)_6\text{-}1H]^{5+}$
1137.0078	1136.8080	$[La_4L^1_4(ClO_4)_7]^{5+}$
1446.2492	1446.2497	$[La_4L^1_4(ClO_4)_8]^{4+}$

Supplementary Figure 273. ESI-TOF-MS spectrum for La^{III}/Ca^{II}/Cd^{II} mixed-metal self-assembly of $\mathbf{L^1}$ (ClO_4^- counter-anion) with insets showing the observed and simulated isotope patterns of the +6 peaks.

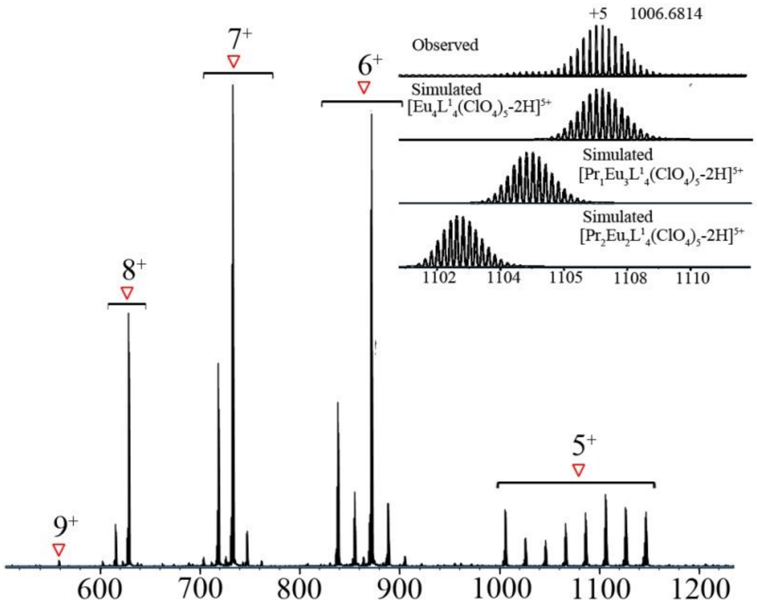

Observed	Simulated	Molecular formular
599.7167	559.7151	$[Eu_4L^1_4\text{-}3H]^{9+}$
629.6799	629.5535	$[Eu_4L^1_4\text{-}4H]^{8+}$
719.3463	719.3459	$[Eu_4L^1_4\text{-}5H]^{7+}$
733.7692	733.7682	$[Eu_4L^1_4(ClO_4)_1\text{-}4H]^{7+}$
839.2365	839.0690	$[Eu_4L^1_4\text{-}6H]^{6+}$
855.8951	855.8950	$[Eu_4L^1_4(ClO_4)_1\text{-}5H]^{6+}$
872.5553	872.5542	$[Eu_4L^1_4(ClO_4)_2\text{-}4H]^{6+}$
889.3815	889.3803	$[Eu_4L^1_4(ClO_4)_3\text{-}3H]^{6+}$
1006.6814	1006.6813	$[Eu_4L^1_4\text{-}7H]^{5+}$
1026.8726	1026.8726	$[Eu_4L^1_4(ClO_4)_1\text{-}6H]^{5+}$
1047.0640	1046.8636	$[Eu_4L^1_4(ClO_4)_2\text{-}5H]^{5+}$
1067.0554	1067.0549	$[Eu_4L^1_4(ClO_4)_3\text{-}4H]^{5+}$
1087.0470	1087.0459	$[Eu_4L^1_4(ClO_4)_4\text{-}3H]^{5+}$
1107.0383	1107.2372	$[Eu_4L^1_4(ClO_4)_5\text{-}2H]^{5+}$
1127.2295	1127.2282	$[Eu_4L^1_4(ClO_4)_6\text{-}1H]^{5+}$
1147.4207	1147.4194	$[Eu_4L^1_4(ClO_4)_7]^{5+}$

Supplementary Figure 274. ESI-TOF-MS spectrum for $La^{III}/Pr^{III}/Eu^{III}$ mixed-metal self-assembly of $\mathbf{L^1}$ (ClO_4^- counter-anion) with insets showing the observed and simulated isotope patterns of the +5 peaks.

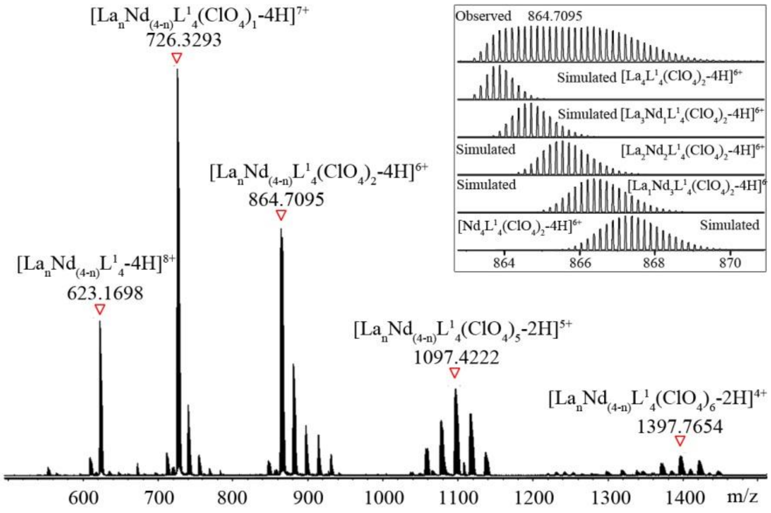

Supplementary Figure 275. ESI-Q-TOF mass spectrum of La^{III}/Nd^{III} mixed-metal self-assembled complexes with L^1 (ClO_4^- salt) with the metal and ligand ratio as $La^{III} : Nd^{III} : L^1 = 0.5 : 0.5 :1$.

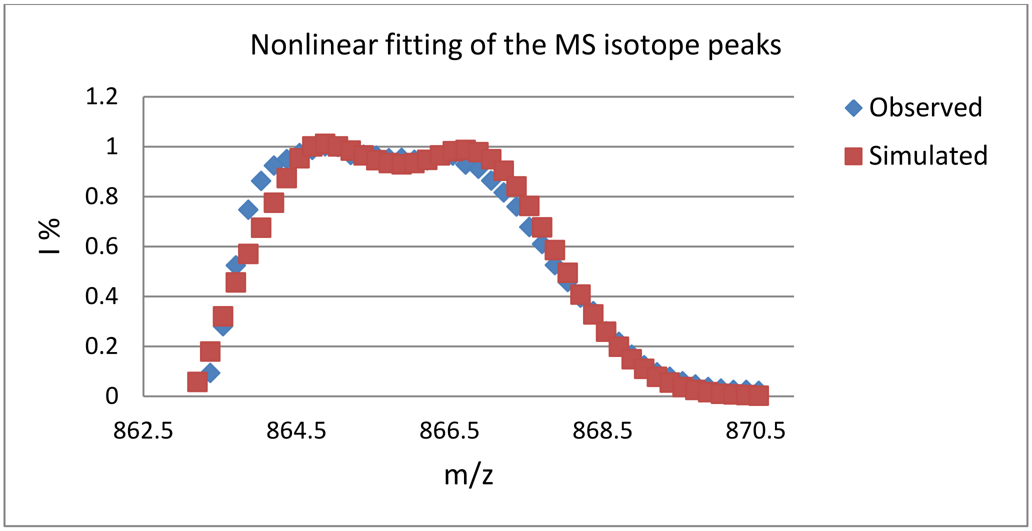

Supplementary Figure 276. Nonlinear curve fitting of simulated isotope patterns of La^{III}/Nd^{III} mixed-metal self-assembled complexes with L^1 ($CF_3SO_3^-$ salt) with the metal and ligand ratio as $La^{III} : Nd^{III} : L^1 = 0.5 : 0.5 : 1$.

^{1}H NMR spectra and ESI-TOF-MS indicated the biased formation of two homometallic cages, along with some statistical mixture of heterometallic complexes. For lanthanide pair of La^{III}/Nd^{III}, nonlinear curve fitting of the isotope patterns using the following models, a : $[La_4(L^1)_4]^{12+}$, b: $[La_3Nd_1(L^1)_4]^{12+}$, c: $[La_2Nd_2(L^1)_4]^{12+}$, d: $[La_1Nd_3(L^1)_4]^{12+}$, e: $[Nd_4(L^1)_4]^{12+}$ (4a+3b+2c+d=b+2c+3d+4e) has been carried out. It turns out that the observed isotope patterns approximately agrees with a composition of $[La_4(L^1)_4]^{12+}$: $[La_3Nd_1(L^1)_4]^{12+}$: $[La_2Nd_2(L^1)_4]^{12+}$: $[La_1Nd_3(L^1)_4]^{12+}$: $[Nd_4(L^1)_4]^{12+}$ = 1.08 : 1.94 : 1.28 : 1 : 1.55

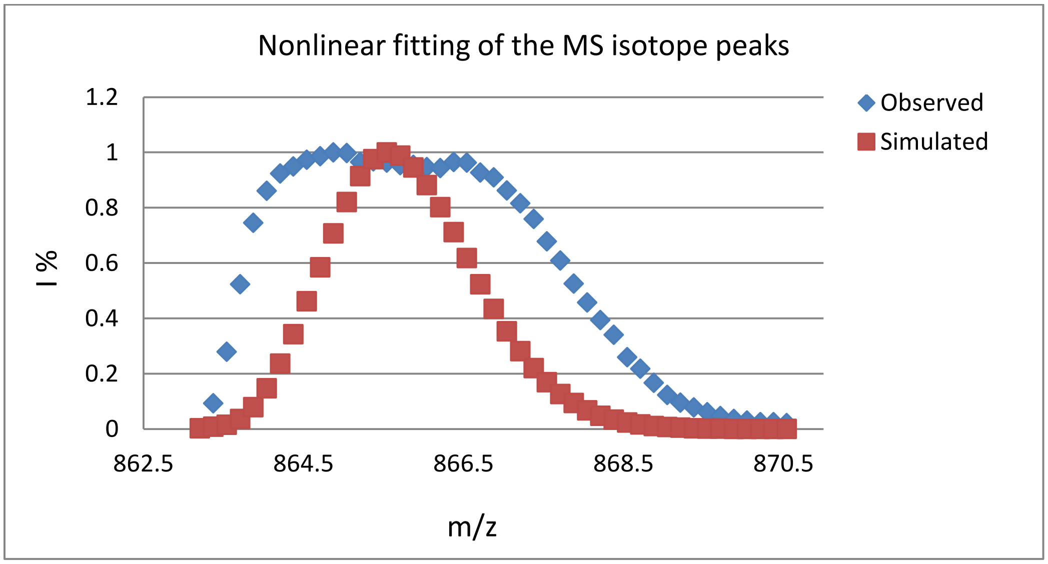

Supplementary Figure 277. Simulated isotope patterns of pure statistically-distributed mixtures of La^{III}/Nd^{III} mixed-metal self-assembled complexes with L^1 ($CF_3SO_3^-$ salt) with the metal and ligand ratio as $La^{III} : Nd^{III} : L^1 = 0.5 : 0.5 : 1$.

In theory, pure statistically-distributed mixtures of $[Lna_nLnb_{4-n}(L^1)_4]^{12+}$ (n = 0-4) species would favour the formation of the $[Lna_2Lnb_2(L^1)_4]^{12+}$ complex (statistical distribution ratio: $[Lna_4(L^1)_4]^{12+}$: $[Lna_3Lnb_1(L^1)_4]^{12+}$: $[Lna_2Lnb_2(L^1)_4]^{12+}$: $[Lna_1Lnb_3(L^1)_4]^{12+}$: $[Lnb_4(L^1)_4]^{12+}$ = $C_4^4 : C_4^1 C_4^3 : C_4^2 C_4^2 : C_4^3 C_4^1 : C_4^4$ = 1 : 16 : 36 : 16 : 1.

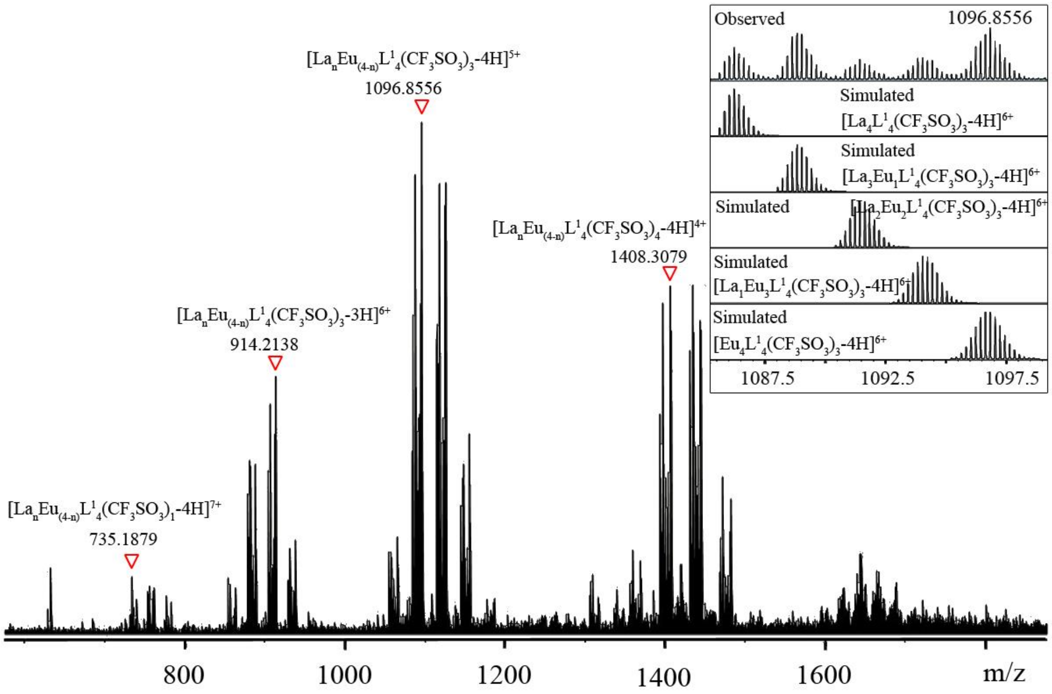

Supplementary Figure 278. Simulated isotope patterns of pure statistically-distributed mixtures of La^{III}/Eu^{III} mixed-metal self-assembly complexes with L^1 ($CF_3SO_3^-$ salt) with the metal and ligand ratio as $La^{III} : Eu^{III} : L^1 = 0.5 : 0.5 : 1$.

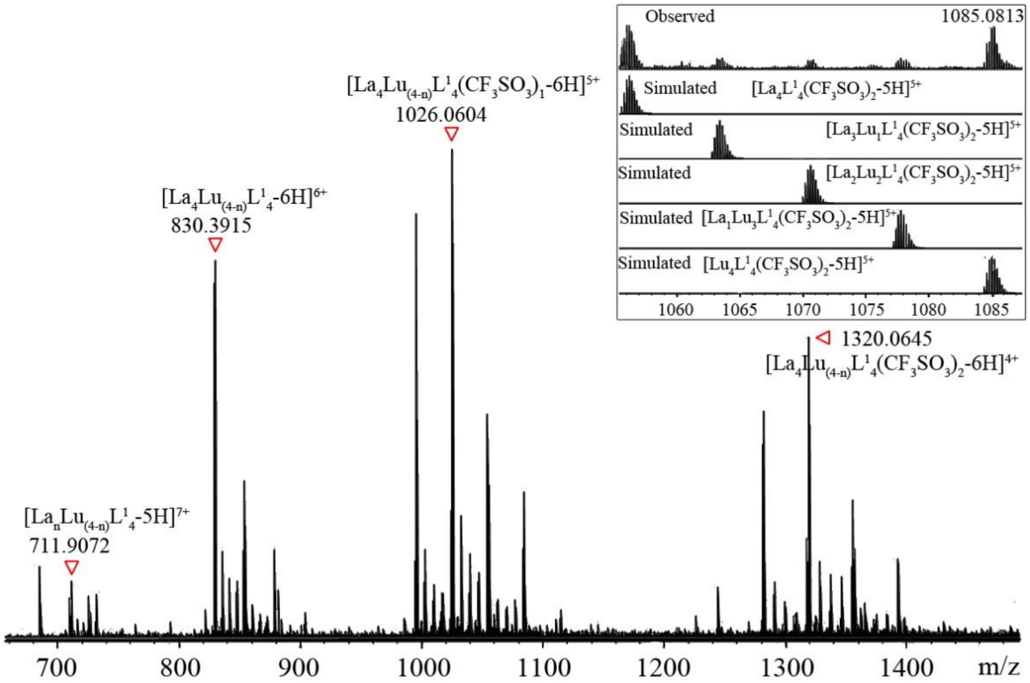

Supplementary Figure 279. Simulated isotope patterns of pure statistically-distributed mixtures of La^{III}/Lu^{III} mixed-metal self-assembly complexes with L^1 ($CF_3SO_3^-$ salt) with the metal and ligand ratio as $La^{III} : Lu^{III} : L^1 = 0.5 : 0.5 : 1$.

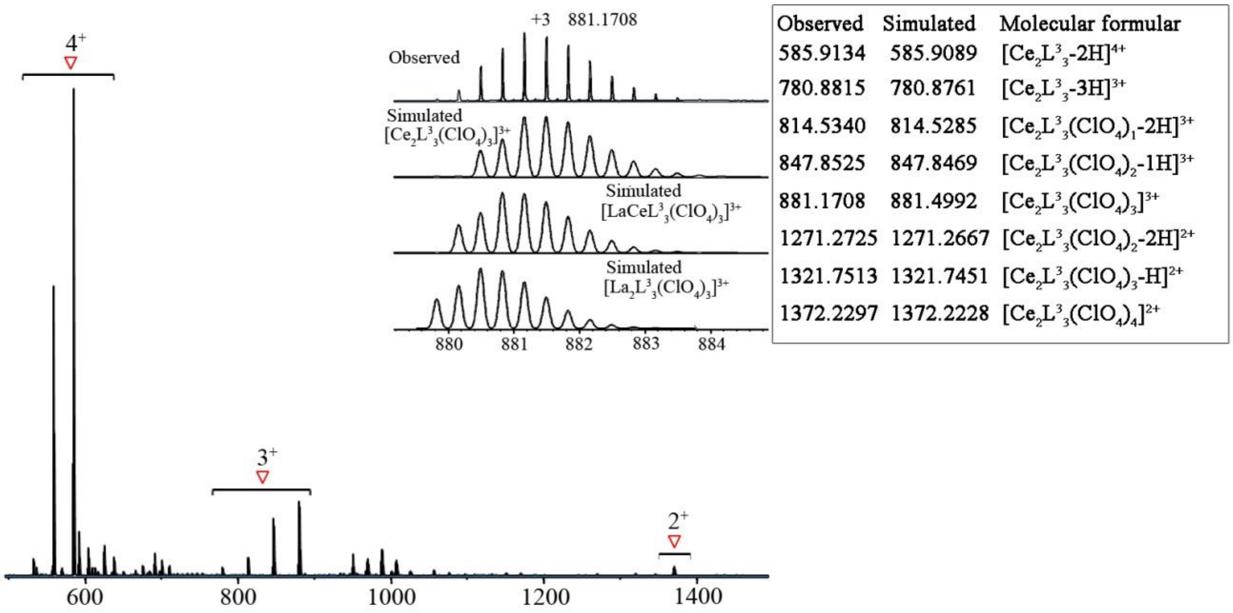

Observed	Simulated	Molecular formular
585.9134	585.9089	$[Ce_2L^3_3\text{-}2H]^{4+}$
780.8815	780.8761	$[Ce_2L^3_3\text{-}3H]^{3+}$
814.5340	814.5285	$[Ce_2L^3_3(ClO_4)_1\text{-}2H]^{3+}$
847.8525	847.8469	$[Ce_2L^3_3(ClO_4)_2\text{-}1H]^{3+}$
881.1708	881.4992	$[Ce_2L^3_3(ClO_4)_3]^{3+}$
1271.2725	1271.2667	$[Ce_2L^3_3(ClO_4)_2\text{-}2H]^{2+}$
1321.7513	1321.7451	$[Ce_2L^3_3(ClO_4)_3\text{-}H]^{2+}$
1372.2297	1372.2228	$[Ce_2L^3_3(ClO_4)_4]^{2+}$

Supplementary Figure 280. ESI-TOF-MS spectrum for La^{III}-Ce^{III} mixed-metal self-assembly of $\mathbf{L^3}$(ClO_4^- counter-anion) with insets showing the observed and simulated isotope patterns of the +3 peaks.

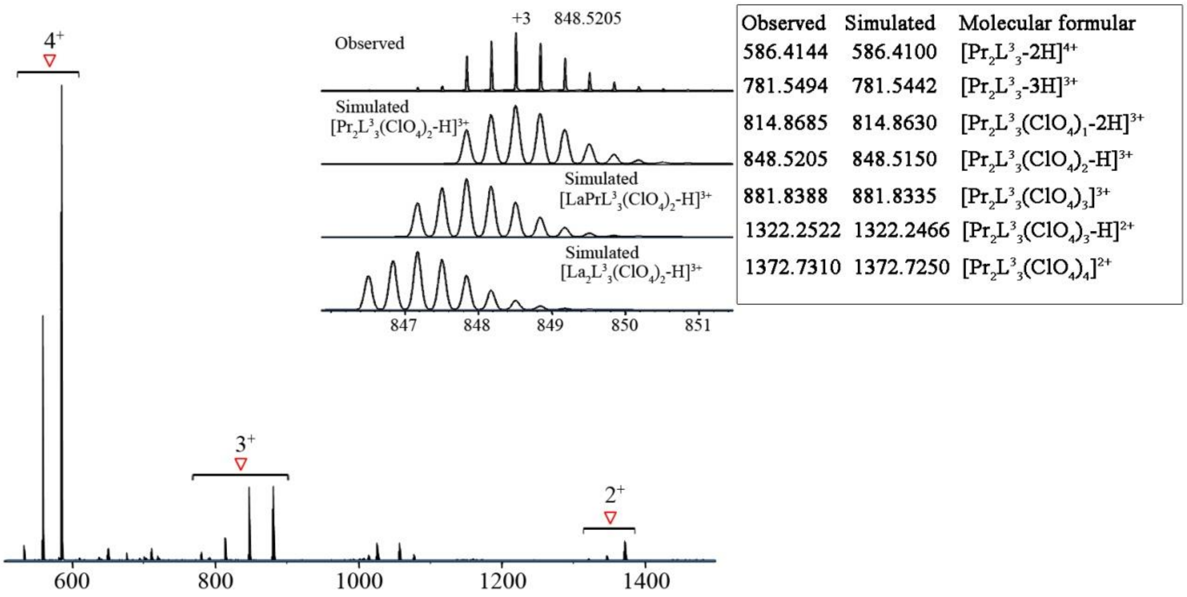

Observed	Simulated	Molecular formular
586.4144	586.4100	$[Pr_2L^3_3\text{-}2H]^{4+}$
781.5494	781.5442	$[Pr_2L^3_3\text{-}3H]^{3+}$
814.8685	814.8630	$[Pr_2L^3_3(ClO_4)_1\text{-}2H]^{3+}$
848.5205	848.5150	$[Pr_2L^3_3(ClO_4)_2\text{-}H]^{3+}$
881.8388	881.8335	$[Pr_2L^3_3(ClO_4)_3]^{3+}$
1322.2522	1322.2466	$[Pr_2L^3_3(ClO_4)_3\text{-}H]^{2+}$
1372.7310	1372.7250	$[Pr_2L^3_3(ClO_4)_4]^{2+}$

Supplementary Figure 281. ESI-TOF-MS spectrum for La^{III}-Pr^{III} mixed-metal self-assembly of $\mathbf{L^3}$(ClO_4^- counter-anion) with insets showing the observed and simulated isotope patterns of the +3 peaks.

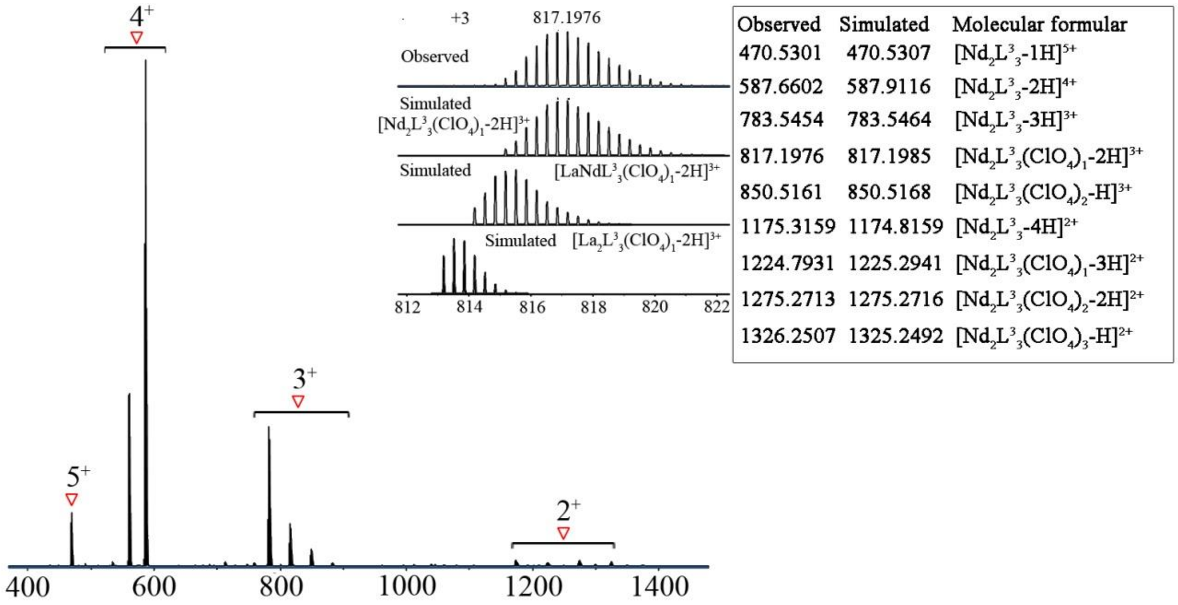

Supplementary Figure 282. ESI-TOF-MS spectrum for La^{III}-Nd^{III} mixed-metal self-assembly of $\mathbf{L^3}$(ClO_4^- counter-anion) with insets showing the observed and simulated isotope patterns of the +3 peaks.

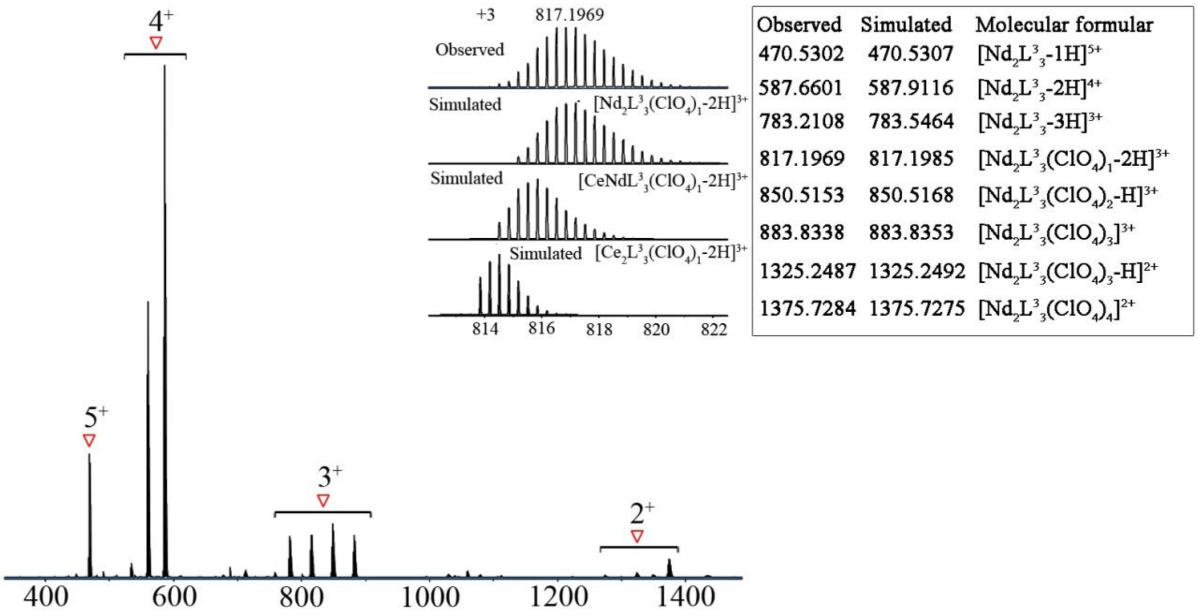

Supplementary Figure 283. ESI-TOF-MS spectrum for Ce^{III}-Nd^{III} mixed-metal self-assembly of $\mathbf{L^3}$(ClO_4^- counter-anion) with insets showing the observed and simulated isotope patterns of the +3 peaks.

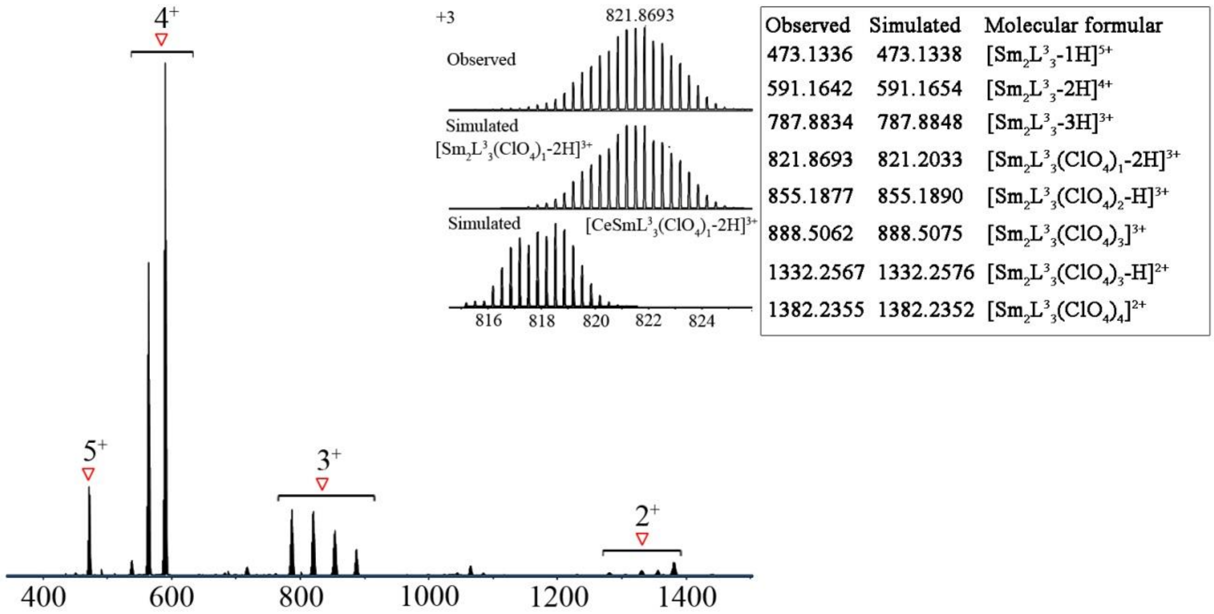

Observed	Simulated	Molecular formular
473.1336	473.1338	$[Sm_2L^3_3\text{-}1H]^{5+}$
591.1642	591.1654	$[Sm_2L^3_3\text{-}2H]^{4+}$
787.8834	787.8848	$[Sm_2L^3_3\text{-}3H]^{3+}$
821.8693	821.2033	$[Sm_2L^3_3(ClO_4)_1\text{-}2H]^{3+}$
855.1877	855.1890	$[Sm_2L^3_3(ClO_4)_2\text{-}H]^{3+}$
888.5062	888.5075	$[Sm_2L^3_3(ClO_4)_3]^{3+}$
1332.2567	1332.2576	$[Sm_2L^3_3(ClO_4)_3\text{-}H]^{2+}$
1382.2355	1382.2352	$[Sm_2L^3_3(ClO_4)_4]^{2+}$

Supplementary Figure 284. ESI-TOF-MS spectrum for Ce^{III}-Sm^{III} mixed-metal self-assembly of $\mathbf{L^3}$(ClO_4^- counter-anion) with insets showing the observed and simulated isotope patterns of the +3 peaks.

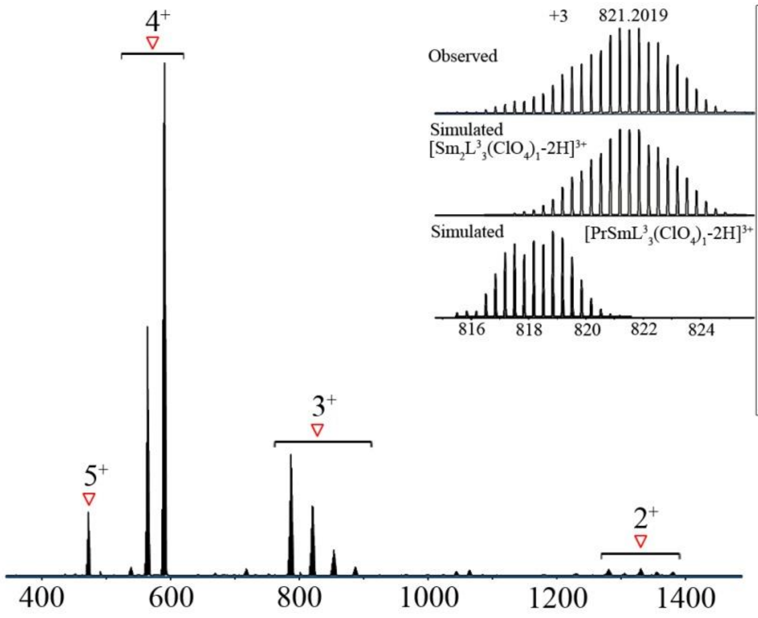

Observed	Simulated	Molecular formular
473.1330	473.1338	$[Sm_2L^3_3\text{-}1H]^{5+}$
591.1639	591.1654	$[Sm_2L^3_3\text{-}2H]^{4+}$
787.8836	787.8848	$[Sm_2L^3_3\text{-}3H]^{3+}$
821.2019	821.2033	$[Sm_2L^3_3(ClO_4)_1\text{-}2H]^{3+}$
855.1877	855.1890	$[Sm_2L^3_3(ClO_4)_2\text{-}H]^{3+}$
888.5062	888.5075	$[Sm_2L^3_3(ClO_4)_3]^{3+}$
1232.3003	1231.3013	$[Sm_2L^3_3(ClO_4)_1\text{-}3H]^{2+}$
1281.7770	1282.2799	$[Sm_2L^3_3(ClO_4)_2\text{-}2H]^{2+}$
1332.2557	1332.2576	$[Sm_2L^3_3(ClO_4)_3\text{-}H]^{2+}$
1382.2349	1382.2352	$[Sm_2L^3_3(ClO_4)_4]^{2+}$

Supplementary Figure 285. ESI-TOF-MS spectrum for Pr^{III}-Sm^{III} mixed-metal self-assembly of $\mathbf{L^3}$(ClO_4^- counter-anion) with insets showing the observed and simulated isotope patterns of the +3 peaks.

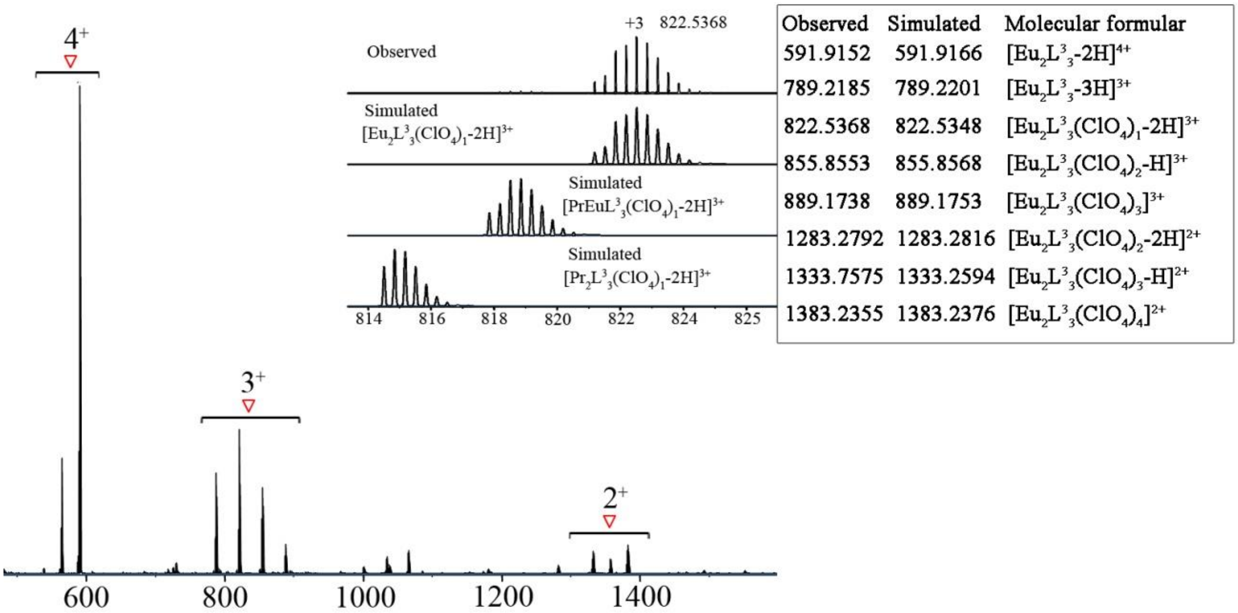

Observed	Simulated	Molecular formular
591.9152	591.9166	$[Eu_2L^3_3\text{-}2H]^{4+}$
789.2185	789.2201	$[Eu_2L^3_3\text{-}3H]^{3+}$
822.5368	822.5348	$[Eu_2L^3_3(ClO_4)_1\text{-}2H]^{3+}$
855.8553	855.8568	$[Eu_2L^3_3(ClO_4)_2\text{-}H]^{3+}$
889.1738	889.1753	$[Eu_2L^3_3(ClO_4)_3]^{3+}$
1283.2792	1283.2816	$[Eu_2L^3_3(ClO_4)_2\text{-}2H]^{2+}$
1333.7575	1333.2594	$[Eu_2L^3_3(ClO_4)_3\text{-}H]^{2+}$
1383.2355	1383.2376	$[Eu_2L^3_3(ClO_4)_4]^{2+}$

Supplementary Figure 286. ESI-TOF-MS spectrum for Pr^{III}-Eu^{III} mixed-metal self-assembly of $\mathbf{L^3}$(ClO_4^- counter-anion) with insets showing the observed and simulated isotope patterns of the +3 peaks.

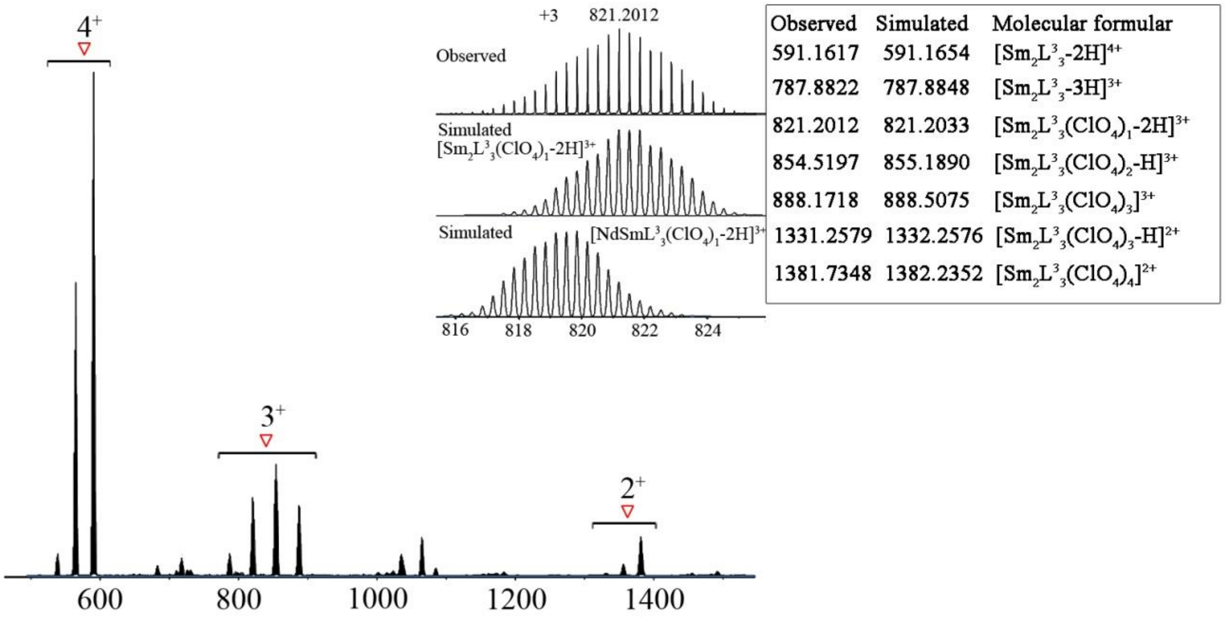

Observed	Simulated	Molecular formular
591.1617	591.1654	$[Sm_2L^3_3\text{-}2H]^{4+}$
787.8822	787.8848	$[Sm_2L^3_3\text{-}3H]^{3+}$
821.2012	821.2033	$[Sm_2L^3_3(ClO_4)_1\text{-}2H]^{3+}$
854.5197	855.1890	$[Sm_2L^3_3(ClO_4)_2\text{-}H]^{3+}$
888.1718	888.5075	$[Sm_2L^3_3(ClO_4)_3]^{3+}$
1331.2579	1332.2576	$[Sm_2L^3_3(ClO_4)_3\text{-}H]^{2+}$
1381.7348	1382.2352	$[Sm_2L^3_3(ClO_4)_4]^{2+}$

Supplementary Figure 287. ESI-TOF-MS spectrum for Nd^{III}-Sm^{III} mixed-metal self-assembly of $\mathbf{L^3}$(ClO_4^- counter-anion) with insets showing the observed and simulated isotope patterns of the +3 peaks.

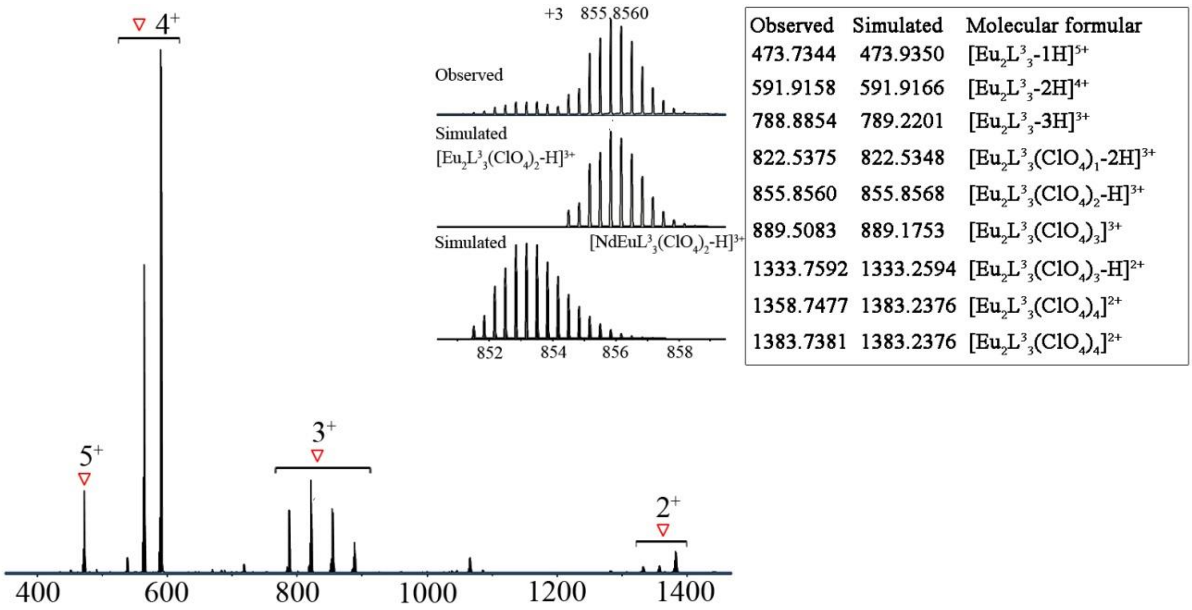

Observed	Simulated	Molecular formular
473.7344	473.9350	$[Eu_2L^3_3\text{-}1H]^{5+}$
591.9158	591.9166	$[Eu_2L^3_3\text{-}2H]^{4+}$
788.8854	789.2201	$[Eu_2L^3_3\text{-}3H]^{3+}$
822.5375	822.5348	$[Eu_2L^3_3(ClO_4)_1\text{-}2H]^{3+}$
855.8560	855.8568	$[Eu_2L^3_3(ClO_4)_2\text{-}H]^{3+}$
889.5083	889.1753	$[Eu_2L^3_3(ClO_4)_3]^{3+}$
1333.7592	1333.2594	$[Eu_2L^3_3(ClO_4)_3\text{-}H]^{2+}$
1358.7477	1383.2376	$[Eu_2L^3_3(ClO_4)_4]^{2+}$
1383.7381	1383.2376	$[Eu_2L^3_3(ClO_4)_4]^{2+}$

Supplementary Figure 288. ESI-TOF-MS spectrum for Nd^{III}-Eu^{III} mixed-metal self-assembly of $\mathbf{L^3}$(ClO_4^- counter-anion) with insets showing the observed and simulated isotope patterns of the +3 peaks.

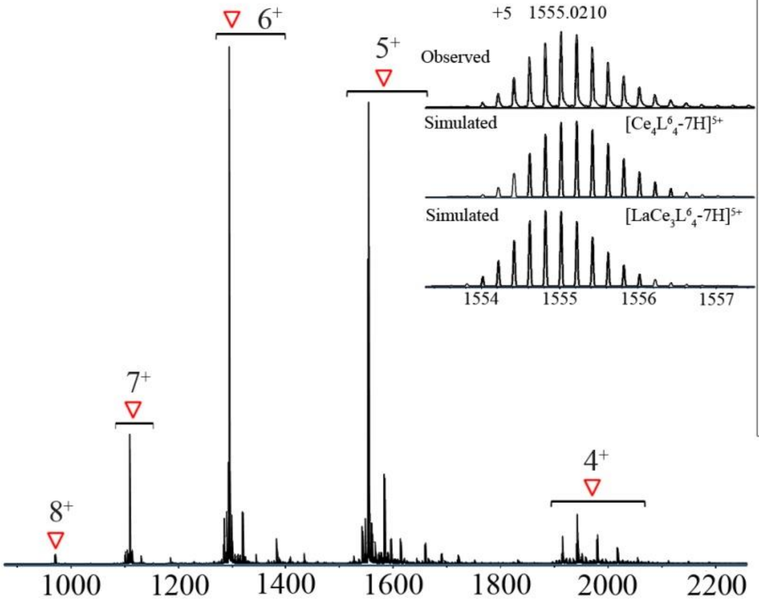

Observed	Simulated	Molecular formular
972.3913	972.3916	$[Ce_4L^6_4\text{-}4H]^{8+}$
1111.1614	1111.1608	$[Ce_4L^6_4\text{-}5H]^{7+}$
1132.4400	1132.5836	$[Ce_4L^6_4(CF_3SO_3)\text{-}4H]^{7+}$
1296.0195	1296.1864	$[Ce_4L^6_4\text{-}6H]^{6+}$
1321.1793	1321.1797	$[Ce_4L^6_4(CF_3SO_3)\text{-}5H]^{6+}$
1555.0210	1555.2223	$[Ce_4L^6_4\text{-}7H]^{5+}$
1585.0121	1585.2142	$[Ce_4L^6_4(CF_3SO_3)\text{-}6H]^{5+}$
1943.7746	1943.7760	$[Ce_4L^6_4\text{-}8H]^{4+}$
1981.2645	1981.2659	$[Ce_4L^6_4(CF_3SO_3)\text{-}7H]^{4+}$
2018.5043	2018.7558	$[Ce_4L^6_4(CF_3SO_3)_2\text{-}6H]^{4+}$
2055.9919	2056.2456	$[Ce_4L^6_4(CF_3SO_3)_3\text{-}5H]^{4+}$

Supplementary Figure 289. ESI-TOF-MS spectrum for La^{III}-Ce^{III} mixed-metal self-assemblyof $\mathbf{L^6}$ ($CF_3SO_3^-$ counter-anion) with insets showing the observed and simulated isotope patterns of the +5 peaks.

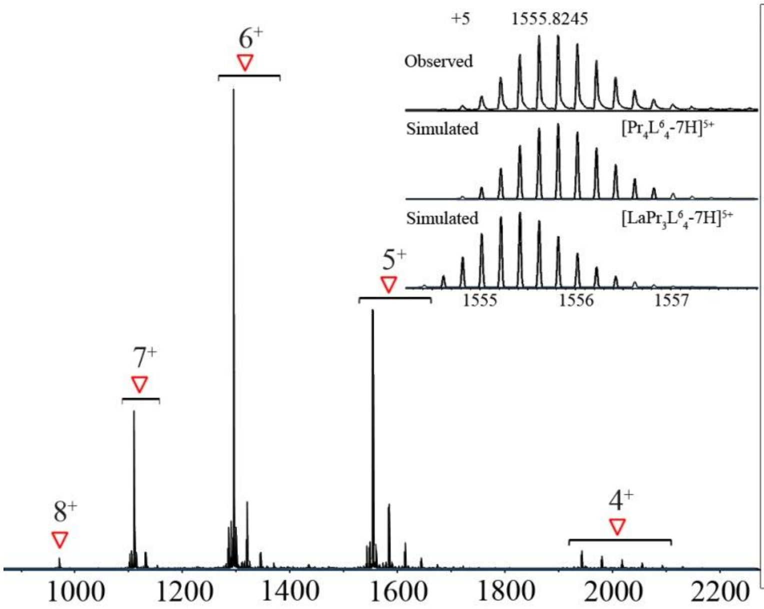

Observed	Simulated	Molecular formular
972.7700	972.7675	$[Pr_4L^6_4\text{-}4H]^{8+}$
1111.5930	1111.5904	$[Pr_4L^6_4\text{-}5H]^{7+}$
1133.0155	1133.0131	$[Pr_4L^6_4(CF_3SO_3)\text{-}4H]^{7+}$
1296.6893	1296.6875	$[Pr_4L^6_4\text{-}6H]^{6+}$
1321.6829	1321.6808	$[Pr_4L^6_4(CF_3SO_3)\text{-}5H]^{6+}$
1346.6763	1346.6740	$[Pr_4L^6_4(CF_3SO_3)_2\text{-}4H]^{6+}$
1555.8245	1555.8236	$[Pr_4L^6_4\text{-}7H]^{5+}$
1585.8162	1585.8155	$[Pr_4L^6_4(CF_3SO_3)\text{-}6H]^{5+}$
1615.8052	1615.8074	$[Pr_4L^6_4(CF_3SO_3)_2\text{-}5H]^{5+}$
1645.8005	1645.7993	$[Pr_4L^6_4(CF_3SO_3)_3\text{-}4H]^{5+}$
1944.5264	1944.5277	$[Pr_4L^6_4\text{-}8H]^{4+}$
1982.0168	1982.0175	$[Pr_4L^6_4(CF_3SO_3)\text{-}7H]^{4+}$
2019.5080	2019.5074	$[Pr_4L^6_4(CF_3SO_3)_2\text{-}6H]^{4+}$
2056.9980	2056.9973	$[Pr_4L^6_4(CF_3SO_3)_3\text{-}5H]^{4+}$
2094.4866	2094.4872	$[Pr_4L^6_4(CF_3SO_3)_4\text{-}4H]^{4+}$

Supplementary Figure 290. ESI-TOF-MS spectrum for La^{III}-Pr^{III} mixed-metal self-assemblyof $\mathbf{L^6}$ ($CF_3SO_3^-$ counter-anion) with insets showing the observed and simulated isotope patterns of the +5 peaks.

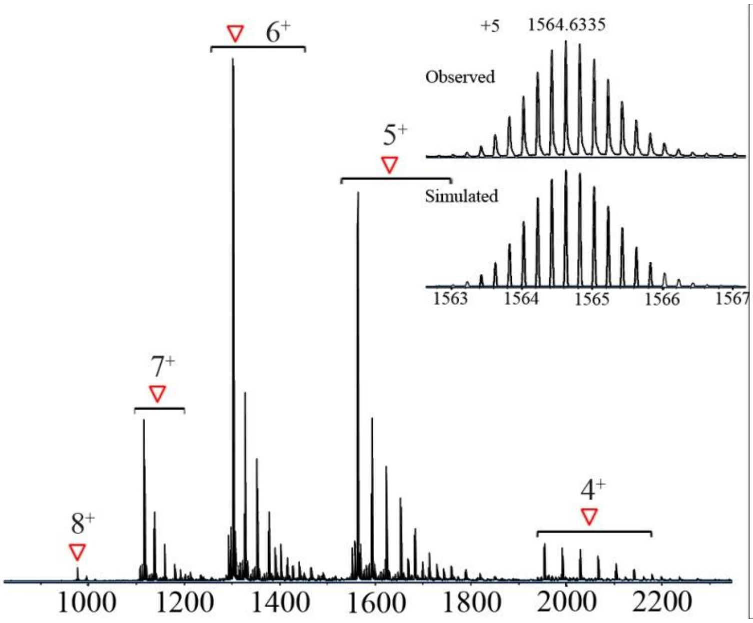

Observed	Simulated	Molecular formular
978.2751	978.2739	$[Eu_4L^6_4\text{-}4H]^{8+}$
1117.8849	1117.8834	$[Eu_4L^6_4\text{-}5H]^{7+}$
1139.3074	1139.3062	$[Eu_4L^6_4(CF_3SO_3)\text{-}4H]^{7+}$
1160.7305	1160.7290	$[Eu_4L^6_4(CF_3SO_3)_2\text{-}3H]^{7+}$
1304.0301	1304.0294	$[Eu_4L^6_4\text{-}6H]_{6+}$
1329.0234	1329.0227	$[Eu_4L^6_4(CF_3SO_3)\text{-}5H]^{6+}$
1354.0167	1354.0160	$[Eu_4L^6_4(CF_3SO_3)_2\text{-}4H]^{6+}$
1379.0103	1379.1762	$[Eu_4L^6_4(CF_3SO_3)_3\text{-}3H]^{6+}$
1564.6335	1564.6339	$[Eu_4L^6_4\text{-}7H]^{5+}$
1594.6247	1594.6258	$[Eu_4L^6_4(CF_3SO_3)\text{-}6H]^{5+}$
1624.6172	1624.6177	$[Eu_4L^6_4(CF_3SO_3)_2\text{-}5H]^{5+}$
1654.8101	1654.8100	$[Eu_4L^6_4(CF_3SO_3)_3\text{-}4H]^{5+}$
1995.5388	1955.5405	$[Eu_4L^6_4\text{-}8H]^{4+}$
1993.0286	1993.0304	$[Eu_4L^6_4(CF_3SO_3)\text{-}7H]^{4+}$
2030.7690	2030.5203	$[Eu_4L^6_4(CF_3SO_3)_2\text{-}6H]^{4+}$
2068.2592	2068.2607	$[Eu_4L^6_4(CF_3SO_3)_3\text{-}5H]^{4+}$

Supplementary Figure 291. ESI-TOF-MS spectrum for La^{III}-Eu^{III} mixed-metal self-assemblyof $\mathbf{L^6}$ ($CF_3SO_3^-$ counter-anion) with insets showing the observed and simulated isotope patterns of the +5 peaks.

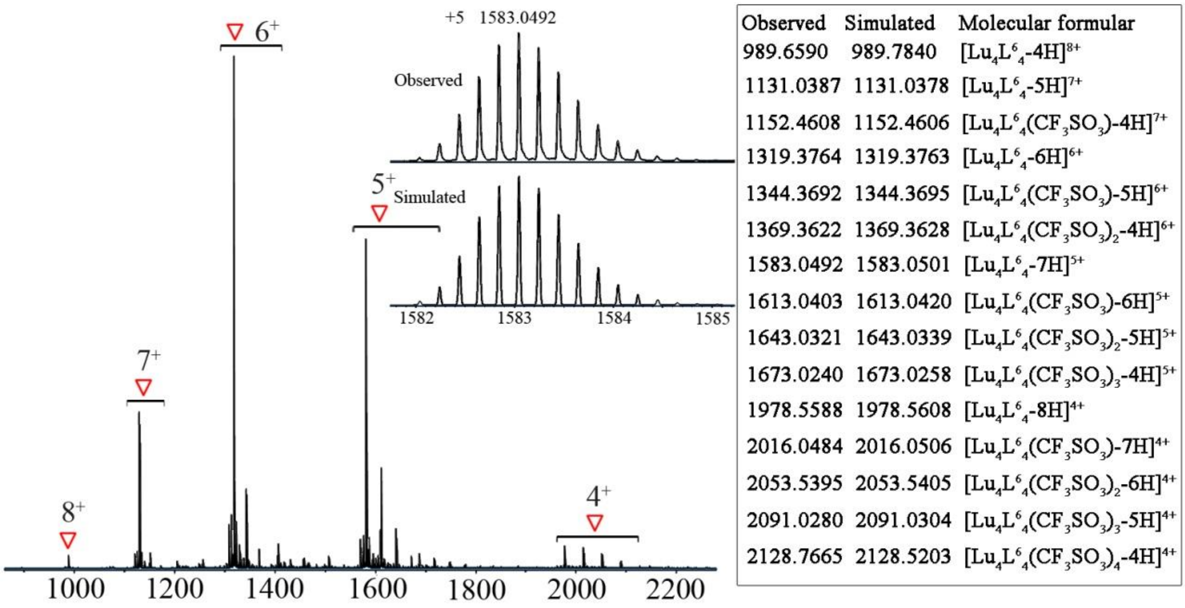

Observed	Simulated	Molecular formular
989.6590	989.7840	$[Lu_4L^6{}_4\text{-}4H]^{8+}$
1131.0387	1131.0378	$[Lu_4L^6{}_4\text{-}5H]^{7+}$
1152.4608	1152.4606	$[Lu_4L^6{}_4(CF_3SO_3)\text{-}4H]^{7+}$
1319.3764	1319.3763	$[Lu_4L^6{}_4\text{-}6H]^{6+}$
1344.3692	1344.3695	$[Lu_4L^6{}_4(CF_3SO_3)\text{-}5H]^{6+}$
1369.3622	1369.3628	$[Lu_4L^6{}_4(CF_3SO_3)_2\text{-}4H]^{6+}$
1583.0492	1583.0501	$[Lu_4L^6{}_4\text{-}7H]^{5+}$
1613.0403	1613.0420	$[Lu_4L^6{}_4(CF_3SO_3)\text{-}6H]^{5+}$
1643.0321	1643.0339	$[Lu_4L^6{}_4(CF_3SO_3)_2\text{-}5H]^{5+}$
1673.0240	1673.0258	$[Lu_4L^6{}_4(CF_3SO_3)_3\text{-}4H]^{5+}$
1978.5588	1978.5608	$[Lu_4L^6{}_4\text{-}8H]^{4+}$
2016.0484	2016.0506	$[Lu_4L^6{}_4(CF_3SO_3)\text{-}7H]^{4+}$
2053.5395	2053.5405	$[Lu_4L^6{}_4(CF_3SO_3)_2\text{-}6H]^{4+}$
2091.0280	2091.0304	$[Lu_4L^6{}_4(CF_3SO_3)_3\text{-}5H]^{4+}$
2128.7665	2128.5203	$[Lu_4L^6{}_4(CF_3SO_3)_4\text{-}4H]^{4+}$

Supplementary Figure 292. ESI-TOF-MS spectrum for La^{III}-Lu^{III} mixed-metal self-assemblyof $\mathbf{L^6}$ ($CF_3SO_3^-$ counter-anion) with insets showing the observed and simulated isotope patterns of the +5 peaks.

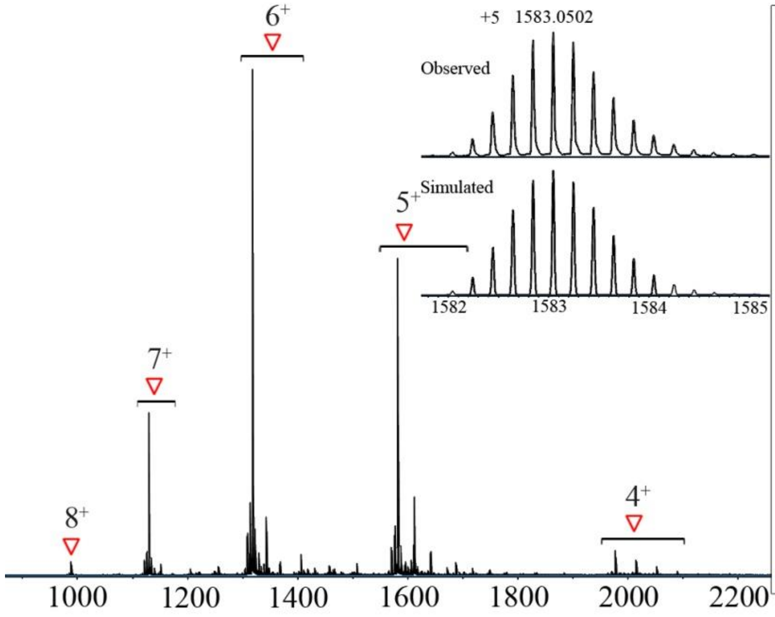

Observed	Simulated	Molecular formular
989.7848	989.7840	$[Lu_4L^6{}_4\text{-}4H]^{8+}$
1131.0391	1131.0378	$[Lu_4L^6{}_4\text{-}5H]^{7+}$
1152.4612	1152.4606	$[Lu_4L^6{}_4(CF_3SO_3)\text{-}4H]^{7+}$
1319.3771	1319.3763	$[Lu_4L^6{}_4\text{-}6H]^{6+}$
1344.3692	1344.3695	$[Lu_4L^6{}_4(CF_3SO_3)\text{-}5H]^{6+}$
1369.3625	1369.3628	$[Lu_4L^6{}_4(CF_3SO_3)_2\text{-}4H]^{6+}$
1583.0502	1583.0501	$[Lu_4L^6{}_4\text{-}7H]^{5+}$
1613.0406	1613.0420	$[Lu_4L^6{}_4(CF_3SO_3)\text{-}6H]^{5+}$
1643.0330	1643.0339	$[Lu_4L^6{}_4(CF_3SO_3)_2\text{-}5H]^{5+}$
1673.2247	1673.0258	$[Lu_4L^6{}_4(CF_3SO_3)_3\text{-}4H]^{5+}$
1978.5593	1978.5608	$[Lu_4L^6{}_4\text{-}8H]^{4+}$
2016.0476	2016.0506	$[Lu_4L^6{}_4(CF_3SO_3)\text{-}7H]^{4+}$
2053.5390	2053.5405	$[Lu_4L^6{}_4(CF_3SO_3)_2\text{-}6H]^{4+}$
2091.0266	2091.0304	$[Lu_4L^6{}_4(CF_3SO_3)_3\text{-}5H]^{4+}$
2128.7690	2128.5203	$[Lu_4L^6{}_4(CF_3SO_3)_4\text{-}4H]^{4+}$

Supplementary Figure 293. ESI-TOF-MS spectrum for Eu^{III}-Lu^{III} mixed-metal self-assemblyof $\mathbf{L^6}$ ($CF_3SO_3^-$ counter-anion) with insets showing the observed and simulated isotope patterns of the +5 peaks.

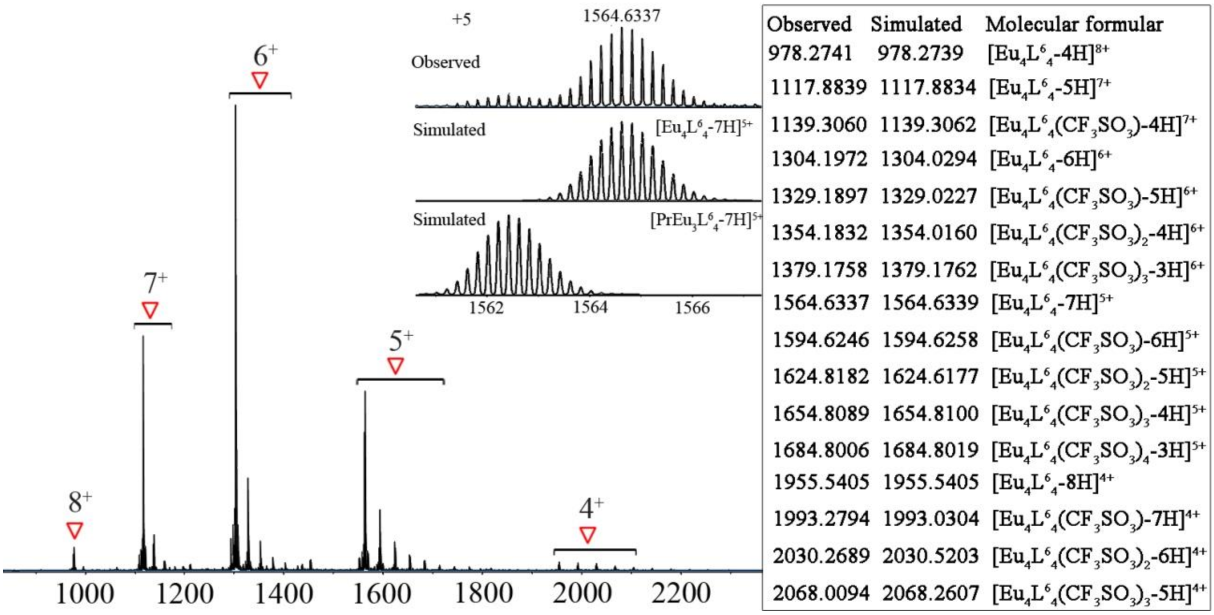

Observed	Simulated	Molecular formular
978.2741	978.2739	$[Eu_4L^6_4\text{-}4H]^{8+}$
1117.8839	1117.8834	$[Eu_4L^6_4\text{-}5H]^{7+}$
1139.3060	1139.3062	$[Eu_4L^6_4(CF_3SO_3)\text{-}4H]^{7+}$
1304.1972	1304.0294	$[Eu_4L^6_4\text{-}6H]^{6+}$
1329.1897	1329.0227	$[Eu_4L^6_4(CF_3SO_3)\text{-}5H]^{6+}$
1354.1832	1354.0160	$[Eu_4L^6_4(CF_3SO_3)_2\text{-}4H]^{6+}$
1379.1758	1379.1762	$[Eu_4L^6_4(CF_3SO_3)_3\text{-}3H]^{6+}$
1564.6337	1564.6339	$[Eu_4L^6_4\text{-}7H]^{5+}$
1594.6246	1594.6258	$[Eu_4L^6_4(CF_3SO_3)\text{-}6H]^{5+}$
1624.8182	1624.6177	$[Eu_4L^6_4(CF_3SO_3)_2\text{-}5H]^{5+}$
1654.8089	1654.8100	$[Eu_4L^6_4(CF_3SO_3)_3\text{-}4H]^{5+}$
1684.8006	1684.8019	$[Eu_4L^6_4(CF_3SO_3)_4\text{-}3H]^{5+}$
1955.5405	1955.5405	$[Eu_4L^6_4\text{-}8H]^{4+}$
1993.2794	1993.0304	$[Eu_4L^6_4(CF_3SO_3)\text{-}7H]^{4+}$
2030.2689	2030.5203	$[Eu_4L^6_4(CF_3SO_3)_2\text{-}6H]^{4+}$
2068.0094	2068.2607	$[Eu_4L^6_4(CF_3SO_3)_3\text{-}5H]^{4+}$

Supplementary Figure 294. ESI-TOF-MS spectrum for Pr^{III}-Eu^{III} mixed-metal self-assemblyof $\mathbf{L^6}$ ($CF_3SO_3^-$ counter-anion) with insets showing the observed and simulated isotope patterns of the +5 peaks.

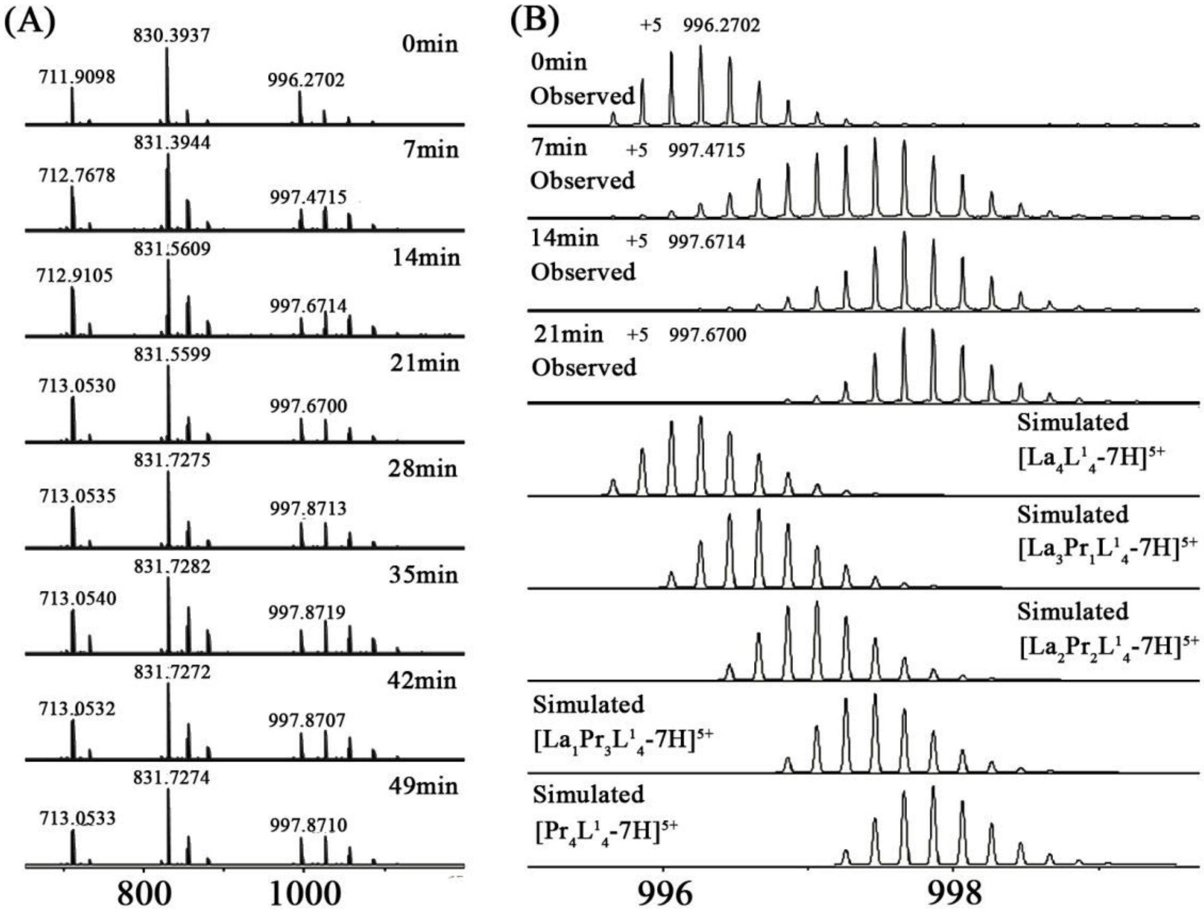

Supplementary Figure 295. ESI-TOF-MS spectra monitoring the post-synthetic metal-ion metathesis experiments of Pr^{III} toward $[La_4L^1{}_4]^{12+}$ (ClO_4^- counter-anion) at different time intervals (A) and the coresponding isotope patterns of the 5+ peaks (B).

By carefully comparing the observed and simulatedisotope patterns of ESI-TOF-MS, it can be seen that a mixture of $[Ln_nPr_{(4-n)}L^1{}_4]^{12+}$ (n=0-4) complexes were observed at 7min and La(III) was gradually substituted by Pr(III) over ca. 30min. The metathesis experiments reached equilibrium faster than that in the NMR experiment, which is possibly due to a low reaction concentration in this case.

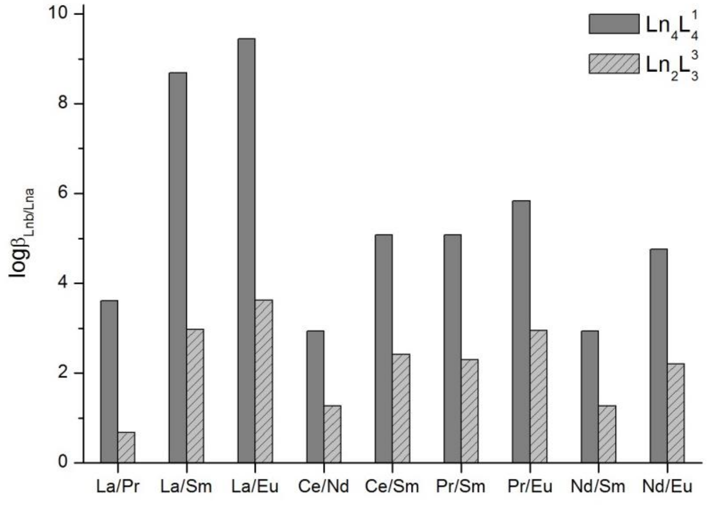

Supplementary Figure 296. Estimated relative stability constants $\beta_{Lnb/Lna}$ comparison of L^1 and L^3 based on post-synthetic metal-ion metathesis experiments**.**

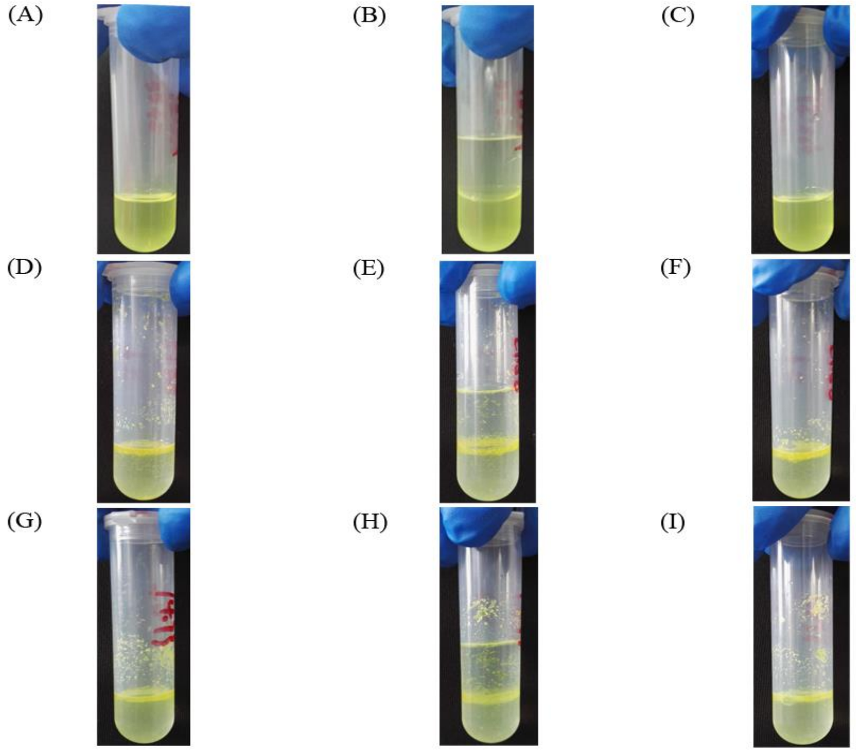

Supplementary Figure 297. Extraction performance of $\mathbf{L^6}$ self-assembled complexes in $CHCl_3$ (A), in $CHCl_3/H_2O$ for 20 min with slight stirring (B), in $CHCl_3$ after removal of H_2O (C); $\mathbf{L^4}$ self-assembled complexes in $CHCl_3$ (D), in $CHCl_3/H_2O$ for 20 min with slight stirring (E), in $CHCl_3$ after removal of H_2O (F) and $\mathbf{L^5}$ self-assembled complexes in $CHCl_3$ (G), in $CHCl_3/H_2O$ for 20 min with slight stirring (H), in $CHCl_3$ after removal of H_2O (I).

Supplementary Tables

Supplementary Table 1. Comparison of the ^{1}H NMR chemical shifts (δ_i) for L^1 and tetrahedral complexes $Ln_4(L^1)_4$ (CD_3CN, 298 K).

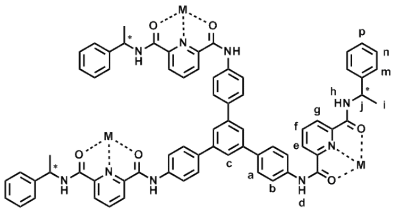

	a	*b*	*c*	*e*	*f*	*g*	*p*	*m*	*n*	*i*	*j*	*d*	*h*
**L^1**	7.93	7.84	7.87	8.42	8.12	8.34	7.24	7.48	7.35	1.70	5.36	10.08	8.67
**$[Ca_4(L^1)_4]^{12+}$**	7.80	7.16	7.20	8.35	8.35	8.35	7.20	7.20	7.20	1.71	5.08	9.74	8.39
**$[Cd_4(L^1)_4]^{12+}$**	7.78	7.15	7.20	8.35	8.35	8.35	7.20	7.20	7.20	1.71	5.09	9.71	8.35
**$[Y_4(L^1)_4]^{12+}$**	7.72	7.21	7.10	8.57	8.40	8.51	7.16	7.16	7.01	1.64	4.96	10.67	9.18
**$[La_4(L^1)_4]^{12+}$**	7.79	7.22	7.17	8.59	8.52	8.47	7.09	7.09	7.17	1.70	5.09	10.35	8.96
**$[Lu_4(L^1)_4]^{12+}$**	7.73	7.20	7.10	8.56	8.39	8.50	7.17	7.17	7.00	1.64	4.94	10.67	9.18
**$[Sm_4(L^1)_4]^{12+}$**	7.70	7.21	7.21	8.49	8.54	8.49	7.10	7.10	7.21	1.69	5.08	10.43	9.16
**$[Ce_4(L^1)_4]^{12+}$**	8.12	7.35	7.16	8.84	8.99	8.72	7.16	7.16	7.23	1.94	5.83	11.38	10.07
**$[Pr_4(L^1)_4]^{12+}$**	5.82	5.53	5.82	10.11	10.03	9.85	7.44	7.16	7.44	1.10	3.65	11.96	11.23
**$[Nd_4(L^1)_4]^{12+}$**	7.69	8.75	7.53	9.23	8.91	9.10	7.18	7.25	7.18	2.21	6.39	12.77	11.84
**$[Eu_4(L^1)_4]^{12+}$**	7.94	8.67	7.73	6.45	7.22	6.38	7.05	7.05	6.94	1.93	5.95	6.94	4.76
**$[Yb_4(L^1)_4]^{12+}$**	9.67	11.94	9.37	7.24	7.04	7.04	6.49	6.49	6.65	2.90	7.94	10.57	8.34

Supplementary Table 2. Comparison of metal ion selectivity with ligands of different coordination units.

	**Ln_2L_3 in Hooley's work[a]**	**Ln_2L_3 in this work**	**Ln_4L_4 in this work**
**La/Sm**	0.36/1	complete	complete
**La/Y**	0.03/1	complete	complete
**Pr/Sm**	0.33/1	0.13/1	0.07/1
**Pr/Y**	0.29/1	complete	complete

[a]Relative percentage of complexes after displacement titrations as reported in literature[S7].

Supplementary Table 3. Selectivity (defined by [Lnb(III)]/[Lna(III)] on the tetrahedral cage complex) observed in one-pot mixed-metal self-assembly using L^{1}.[a]

	La	Ce	Pr	Nd	Sm	Eu	Y	Yb[d]	Lu
$r_{Ln(III)}$/Å[b]	1.22	1.20	1.18	1.16	1.13	1.12	1.08	1.04	1.03
La		8.33	7.69	C	C	C	C	C	C[c]
Ce				5.88	14.29	C	C	-	C
Pr					14.29	20.00	C	-	C
Nd					5.88	12.5	5.00	-	C
Sm							2.17	-	C
Eu							1.54	-	C

[a][Lnb(III)]/[Lna(III)] was calculated from the integrals in the ^{1}H NMR spectra of mixed-metal self-assembly complexes, with a systematic error of 5%. ■ represents complete metal selectivity, ■ represents highly efficient metal selectivity and ■ represents no obvious metal selectivity.
[b]$r_{Ln(III)}$/Å is ionic radii for coordination number as 9[S8].
[c]No Lna(III) complexes were detected by NMR and ESI-MS.
[d] The ionic radii of Ln(III) ions shrinks gradually along the series as a result of lanthanide contraction. While our results on metal ion selectivity mainly follow the ionic radii sequence, other factors, such as electronegativity, could not be neglected. According to the Pauling's rule in electronegativity, lanthanide ions with smaller ionic radii tend to have bigger electronegativity. However, there are always exceptions. For example, Yb(III), which has an ionic radius just bigger than the smallest Lu(III), bears the minimum electronegativity among all the trivalent lanthanides. So there is a difference in metal ion selectivity between Yb(III) and Lu(III).
Moreover, ESI-TOF-MS measurements indicate non-absolute metal ion self-recognition assembly of L^{1} with Ln^{III}/Yb^{III} (Ln = Ce, Pr, Nd, Sm, Eu) mixtures. However, the severe line-broadening of the ^{1}H NMR spectrum caused by paramagnetism of Yb^{III} hinders precise quantitation of different lanthanide ions in the assembled complexes through integration of the NMR spectra.

Supplementary Table 4. Estimated relative stability constants of $[Ln^{b}{}_{4}L^{1}{}_{4}]^{12+}$ over $[Ln^{a}{}_{4}L^{1}{}_{4}]^{12+}$ based on post-synthetic metal-ion metathesis experiments (CD_3CN, 400 MHz, 298 K) (with ±5% exp. error).

**Metal combination**	**log($\beta_{Lnb/Lna}$)**	**Metal combination**	**log($\beta_{Lnb/Lna}$)**
**Ce/La**	3.81	Nd/Ce	2.94
**Pr/La**	3.61	Sm/Ce	5.08
**Nd/La**	6.75 ($\log\beta_{Nd/Ce}+\log\beta_{Ce/La}$)	Sm/Pr	5.08
**Sm/La**	8.89 ($\log\beta_{Sm/Ce}+\log\beta_{Ce/La}$)	Eu/Pr	5.83
	8.69 ($\log\beta_{Sm/Pr}+\log\beta_{Pr/La}$)	Sm/Nd	2.94
**Eu/La**	9.44 ($\log\beta_{Eu/Pr}+\log\beta_{Pr/La}$)	Eu/Nd	4.76

Supplementary Table 5. Estimated relative stability constants of $[Ln^{b}{}_{2}L^{3}{}_{3}]^{6+}$ over $[Ln^{a}{}_{2}L^{3}{}_{3}]^{6+}$ based on post-synthetic metal-ion metathesis experiments (CD_3CN, 400 MHz, 298 K) (with ±5% exp. error).

**Metal combination**	**log($\beta_{Lnb/Lna}$)**	**Metal combination**	**log($\beta_{Lnb/Lna}$)**
**Ce/La**	-	Nd/Ce	1.27
**Pr/La**	0.67	Sm/Ce	2.41
**Sm/La**	2.97 ($\log\beta_{Sm/Pr}+\log\beta_{Pr/La}$)	Sm/Pr	2.30
**Eu/La**	3.62 ($\log\beta_{Eu/Pr}+\log\beta_{Pr/La}$)	Eu/Pr	2.95
		Sm/Nd	1.27
		Eu/Nd	2.20

Supplementary Table 6. Extraction performance of $\mathbf{L^6}$ self-assembled complexes in $CHCl_3/H_2O$.[a]

Metal combination	Starting material/mg	Extract in water/mg	$S_{Lna/Lnb}$
La/Pr	1.18/1.21	0.37/0.219	2.1
	1.28/1.29	0.5972/0.3637	2.2
	1.32/1.24	0.5614/0.3367	1.9
La/Eu	1.00/1.07	0.49/0.11	8.4
	1.19/1.33	0.154/0.022	8.9
	1.25/1.34	0.1418/0.0207	8.1
La/Lu	1.17/1.40	0.57/0.015	87.7
	1.22/1.49	0.8543/0.039	86.7
	1.33/1.61	0.54/0.013	84.0
Pr/Eu	1.10/1.24	0.32/0.127	3.6
	1.29/1.37	0.0838/0.0264	3.6
	1.30/1.36	0.0715/0.0192	4.1
Eu/Lu	1.26/1.36	0.12/0.015	9.4
	1.34/1.54	0.5592/0.0976	10.6
	1.36/1.55	0.477/0.1018	7.7

[a]For both metal combinations of La^{III}/Lu^{III} and Eu^{III}/Lu^{III}, the concention of Lu^{III} was setted as 1 ppb, the lower detection limit of the instrucment, as its concentration after dilution preparation for ICP tests went beyond the lower detection limit (with ±5% exp. error).

Supplementary Table 7. Crystal data and refinement of complex $\mathbf{Cd_4L^1_4}$ (ClO_4^- salt).

Identification code	p6322_final
Empirical formula	C828 H684 Cd12 Cl18 N108 O163
Formula weight	16741.69
Temperature	293(2) K
Wavelength	0.750 Å
Crystal system	Hexagonal
Space group	$P6_322$
Unit cell dimensions	a = 48.446(4) Å α= 90°<br>b = 48.446(4) Å β= 90°.<br>c = 35.873(3) Å γ= 120°
Volume	72916(13) $Å^3$
Z	2
Density (calculated)	0.763 Mg/m^3
Absorption coefficient	0.286 mm^{-1}
F(000)	17188
Crystal size	0.20 x 0.20 x 0.30 mm^3
Theta range for data collection	1.024 to 18.225 °.
Index ranges	-34<=h<=34, -34<=k<=34, -24<=l<=25
Reflections collected	260315
Independent reflections	11880 [R(int) = 0.0639]
Completeness to theta = 18.225 °	80.0 %
Refinement method	Full-matrix least-squares on F^2
Data / restraints / parameters	11880 / 3404 / 1373
Goodness-of-fit on F^2	1.270
Final R indices [I>2sigma(I)]	R1 = 0.0991, wR2 = 0.2659
R indices (all data)	R1 = 0.1059, wR2 = 0.2770
Absolute structure parameter	0.029(17)
Extinction coefficient	n/a
Largest diff. peak and hole	0.464 and -0.241 e.$Å^{-3}$

Supplementary Table 8. Crystal data and refinement of complex $\mathbf{La_4L^1_4}$ (ClO_4^- salt).

Identification code	p213
Empirical formula	C276 H228 Cl12 La4 N36 O80
Formula weight	6309.97
Temperature	293(2) K
Wavelength	0.750 Å
Crystal system	Cubic
Space group	$P2_13$
Unit cell dimensions	a = 42.908(2) Å α= 90°
	b = 42.908(2) Å β= 90°
	c = 42.908(2) Å γ= 90°
Volume	78995(13) $Å^3$
Z	8
Density (calculated)	1.061 Mg/m^3
Absorption coefficient	0.651 mm^{-1}
F(000)	25664
Crystal size	0.18 x 0.18 x 0.18 mm^3
Theta range for data collection	0.708 to 22.024 °.
Index ranges	-42<=h<=42, -38<=k<=38, -42<=l<=42
Reflections collected	464086
Independent reflections	27430 [R(int) = 0.0838]
Completeness to theta = 22.024 °	99.7 %
Absorption correction	None
Refinement method	Full-matrix least-squares on F^2
Data / restraints / parameters	27430 / 3895 / 2392
Goodness-of-fit on F^2	1.090
Final R indices [I>2sigma(I)]	R1 = 0.0940, wR2 = 0.2387
R indices (all data)	R1 = 0.1126, wR2 = 0.2553
Absolute structure parameter	0.042(4)
Extinction coefficient	n/a
Largest diff. peak and hole	0.983 and -1.287 e.$Å^{-3}$

Supplementary Methods

Synthesis and physical properties of ligands $L^{1\text{-}6}$

Synthesis of $L^{1\text{-}3}$: Enantiomeric pure $L^{1\text{-}3}$ were synthesized according to reported procedures with spectroscopic data consistent with the literature. [S1, S2, S3]

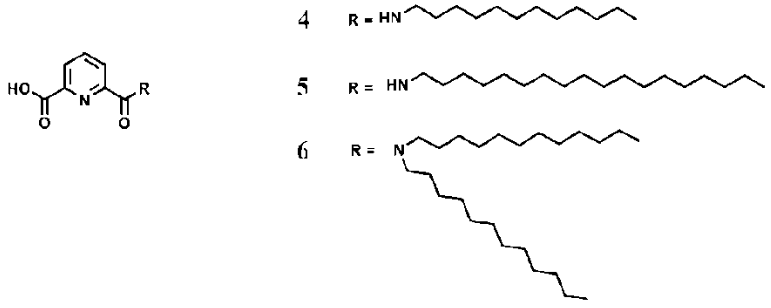

Supplementary Figure 298. Chemical structures of compounds **4-6**.

Synthesis of Compound 6: To a stirred solution of 2,6-pyridinedicarboxylic acid (5.00 g, 30.0 mmol, 2.5 equiv.) in anhydrous DMF/DCM (60 mL) at room temperature, HATU (4.56 g, 12.0 mmol, 1 equiv.) was added under nitrogen. After stirring for 30 min, di-N-dodecylamine (5.09 g, 14.4 mmol, 1.2 equiv.) was added, followed by the addition of DIPEA (5.50 mL, 31.6 mmol, 2.6 equiv.) over 5 min. The resulting solution was stirred at room temperature for 12 h and then diluted with water (100 mL). After extracted with DCM (5 × 30 mL), dried with $MgSO_4$ and concentrated in *vacuo*, the crude product was purified with flash column chromatography (DCM/MeOH = 50:0 to 50:1) to give 6 as a light yellow powder: (5.10 g, 10.1 mmol, 70% yield) ^1H NMR (400 MHz, $CDCl_3$) δ 8.29 (d, *J* = 8.3 Hz, 1H), 8.14–7.96 (m, 1H), 7.91–7.65 (m, 1H), 3.52 (s, 2H), 3.24 (s, 2H), 1.70 (s, 2H), 1.58 (s, 2H), 1.27 (s, 36H), 0.88 (s, 6H). ^{13}C NMR (101 MHz, $CDCl_3$) δ 167.24, 164.00, 154.06, 145.52, 139.32, 126.98, 124.33, 48.96, 45.85, 31.93, 29.63, 29.43, 29.36, 29.17, 29.08, 27.48, 27.12, 26.65, 22.69, 14.12. HR-MS (ESI) *m/z* calcd for $C_{31}H_{55}N_2O_3$ ($[M+H]^{1+}$): 503.4207; found 503.4209.

Compounds 4 and 5 were synthesized in a similar procedure as 6, by replacing di-N-dodecylamine with dodecanamine and octadecanamine, respectively.

4: light yellow powder, 69% yield. ^1H NMR (400 MHz, $CDCl_3$) δ 8.49 (d, *J* = 7.8 Hz, 1H), 8.37 (d, *J* = 7.7 Hz, 1H), 8.19 (s, 1H), 8.11 (t, *J* = 7.8 Hz, 1H), 3.49 (dd, *J* = 13.8, 6.8 Hz, 2H), 1.67–1.60 (m, 2H), 1.39–1.19 (m, 18H), 0.87 (t, *J* = 6.7 Hz, 3H). ^{13}C NMR (101 MHz, $CDCl_3$) δ 164.68, 163.17, 149.79, 145.40, 139.58, 126.87, 126.57, 39.95, 31.91, 29.64, 29.62, 29.54, 29.34, 27.04, 22.68, 14.11. HR-MS (ESI) *m/z* calcd for $C_{19}H_{31}N_2O_3$ ($[M+H]^{1+}$): 335.2329; found 335.2329.

5: light yellow powder, 70% yield. ^1H NMR (400 MHz, $CDCl_3$) δ 8.46 (d, *J* = 7.8 Hz, 1H), 8.34 (d, *J* = 7.6 Hz, 1H), 8.08 (t, *J* = 7.8 Hz, 1H), 8.02 (s, 1H), 3.48 (dd, *J* = 13.7, 6.8 Hz, 2H), 1.67–1.59 (m, 2H), 1.34–1.22 (m, 30H), 0.88 (t, *J* = 6.8 Hz, 3H). ^{13}C NMR (101 MHz, $CDCl_3$) δ 165.01, 163.07, 149.87, 146.05, 139.49, 126.76, 126.43, 39.93, 31.93, 29.71, 29.66, 29.62, 29.56, 29.37, 29.34, 27.05, 22.69, 14.12. HR-MS (ESI) *m/z* calcd for $C_{25}H_{43}N_2O_3$ ($[M+H]^{1+}$): 419.3268; found 419.3265.

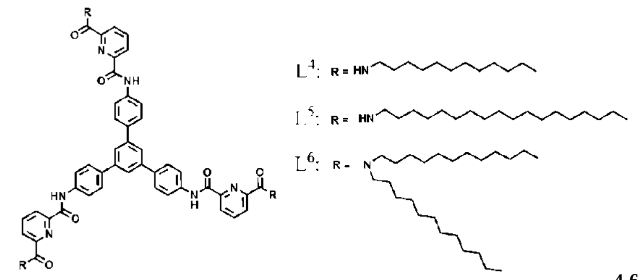

Supplementary Figure 299. Chemical structures of $\mathbf{L^{4\text{-}6}}$.

Synthesis of $L^{4\text{-}6}$: Compound 6 (1.86 g, 3.70 mmol, 1 equiv.) was dissolved in thionyl chloride ($SOCl_2$, 10 mL), followed by the addition of 2 drops of DMF at room temperature. After stirring the reaction mixture at

50 ℃ for 3h, excess $SOCl_2$ was removed in *vacuo* and the resulting residue was used without further purification. The acid chloride was dissolved in anhydrous DCM (50 mL) and treated with 1,3,5-tris(4-aminophenyl)benzene (325.1 mg, 0.92 mmol, 0.25 equiv.) and *N*-ethyldiisopropylamine (1 mL) at 0 ℃. The resulting solution was allowed to warm to room temperature and the reaction was monitored by TLC until all starting materials were consumed. After removing the solvent under reduced pressure, the product was purified with column chromatography (DCM:MeOH = 100:1) to give L^6 as pale yellow solid (1.30 g, 0.72 mmol) in 78% yield. ^1H NMR (400 MHz, $CDCl_3$) δ 9.89 (s, 3H), 8.38(d, *J* = 6.8 Hz, 3H), 8.03(t, *J* = 8.0 Hz, 3H), 7.90(d, *J* = 8.8 Hz, 6H), 7.79 (s, 3H), 7.76 (d, *J* = 8.0 Hz, 6H), 7.69(d, *J* = 8.4 Hz, 3H), 3.56(t, *J* = 8.0 Hz, 6H), 3.24(t, *J* = 8.0 Hz, 6H), 1.73(m, *J* = 7.2 Hz, 6H), 1.62(m, *J* = 4.4 Hz, 12H), 1.40 (s, 12H), 1.27 (s, 42H), 1.12(s, 48H), 0.87(m, *J* = 7.2 Hz, 9H), 0.82(m, *J* = 7.2 Hz, 9H). ^{13}C NMR (101 MHz, $CDCl_3$) δ 168.11, 161.44, 153.95, 148.65, 141.76, 138.84, 137.34, 137.07, 127.97, 125.59, 124.49, 122.85, 120.17, 48.88, 45.62, 31.93, 31.87, 29.68, 29.66, 29.57, 29.54, 29.48, 29.37, 29.29, 29.24, 29.13, 27.51, 27.16, 26.72, 22.69, 22.65, 14.12, 14.10. HR-MS (ESI) *m/z* calcd for $C_{117}H_{178}N_9O_6$ ($[M+H]^{1+}$): 1806.3927; found 1806.3930.

L^4 and L^5 were synthesized in the same procedure as above, by replacing starting material 6 with 4 and 5, respectively.

L^4: pale yellow solid, 75% yield. ^1H NMR (400 MHz, $CDCl_3$) δ 9.63 (s, 3H), 8.46 (dd, *J* = 16.6, 7.7 Hz, 6H), 8.11 (t, *J* = 7.5 Hz, 3H), 7.88 (d, *J* = 8.1 Hz, 6H), 7.85–7.75 (m, 9H), 7.75 (s, 3H), 3.57 (d, *J* = 6.5 Hz, 6H), 1.71 (d, *J* = 6.8 Hz, 12H), 1.45 (s, 6H), 1.25 (s, 42H), 0.84 (d, *J* = 6.6 Hz, 9H). ^{13}C NMR (101 MHz, $CDCl_3$) δ 163.29, 161.34, 149.24, 148.72, 141.72, 139.42, 137.53, 136.83, 128.02, 125.59, 125.25, 124.58, 120.54, 39.84, 31.90, 29.79, 29.67, 29.63, 29.39, 29.34, 27.15, 22.67, 14.11. HR-MS (ESI) *m/z* calcd for $C_{81}H_{106}N_9O_6$ ($[M+H]^{1+}$): 1300.8261; found 1300.8254.

L^5: pale yellow solid, 72% yield. ^1H NMR (400 MHz, $CDCl_3$) δ 9.64(s, 3H), 8.47 (d, *J* = 8.0 Hz, 3H), 8.43 (d, *J* = 8.0 Hz, 3H), 8.09 (t, *J* = 7.6 Hz, 3H), 7.87 (m, *J* = 8.4 Hz, 9H), 7.75(d, *J* = 8.4 Hz, 9H), 3.56 (m, *J* = 6.8 Hz, 6H), 1.71 (m, *J* = 7.2 Hz, 6H), 1.43 (m, *J* = 6.4 Hz, 6H), 1.22 (s, 84H), 0.87(t, *J* = 6.4 Hz, 9H). ^{13}C NMR (101 MHz, $CDCl_3$) δ 163.31, 161.35, 149.27, 148.74, 141.64, 139.38, 137.48, 136.83, 127.97, 125.61, 125.22, 124.58, 120.56, 100.00, 39.85, 31.91, 29.69, 29.65, 29.39, 29.35, 27.15, 22.68, 14.11, 10.78. HR-MS (ESI) *m/z* calcd for $C_{99}H_{142}N_9O_6$ ($[M+H]^{1+}$): 1554.1110; found 1554.1137.

Synthesis and physical properties of $[M_4L^1_4]^{8+}$ and $[Ln_4L^1_4]^{12+}$

$[Pr_4L^1_4](ClO_4)_{12}$: ^1H NMR (400 MHz, CD_3CN) δ 11.96(s, 3H), 11.23(s, 3H), 10.11(s, 3H), 10.03(t, *J* = 6.0 Hz, 3H), 9.85(d, *J* = 6.0 Hz, 3H), 7.44(t, *J* = 8.0 Hz, 9H), 7.16(d, *J* = 4.8 Hz, 6H), 5.82(s, 9H), 5.53(s, 6H), 3.65(s, 3H), 1.10(s, 9H). ^{13}C NMR (101 MHz, CD_3CN) δ 167.27, 152.02, 151.45, 142.42, 142.01, 141.24, 139.14, 137.14, 134.49, 129.43, 128.49, 126.59, 126.30, 122.50, 121.45, 53.14, 29.92, 20.43. ESI-TOF-MS calcd for $[M\text{-}8(ClO_4^-)\text{-}4(HClO_4)]^{8+}$ 624.0471, found 624.0475; calcd for $[M\text{-}7(ClO_4^-)\text{-}5(HClO_4)]^{7+}$ 713.0528, found 713.0533; calcd for $[M\text{-}6(ClO_4^-)\text{-}6(HClO_4)]^{6+}$ 831.7271, found 831.7277; calcd for $[M\text{-}5(ClO_4^-)\text{-}7(HClO_4)]^{5+}$ 997.8710, found 997.8714; calcd for $[M\text{-}4(ClO_4^-)\text{-}7(HClO_4)]^{4+}$ 1272.0757, found 1272.0751.

$[Nd_4L^1_4](ClO_4)_{12}$: ^1H NMR (400 MHz, CD_3CN) δ 12.77(s, 3H), 11.84(s, 3H), 9.23(d, *J* = 6.8 Hz, 3H), 9.10(d, *J* = 6.4 Hz, 3H), 8.91(d, *J* = 6.4 Hz, 3H), 8.75(s, 6H), 7.69(s, 6H), 7.53(s, 3H), 7.25(s, 6H), 7.18(s, 9H), 6.39(s, 3H), 2.21(s, 9H). ^{13}C NMR (101 MHz, CD_3CN) δ 168.22, 166.21, 149.04, 143.80, 143.52, 142.32, 140.79, 138.97, 138.82, 136.63, 129.19, 128.26, 128.19, 126.54, 124.14, 123.72, 54.60, 29.92, 21.56. ESI-TOF-MS calcd for $[M\text{-}9(ClO_4^-)\text{-}3(HClO_4)]^{9+}$ 556.1551, found 556.1555; calcd for $[M\text{-}8(ClO_4^-)\text{-}4(HClO_4)]^{8+}$ 625.5486, found 625.5495; calcd for $[M\text{-}7(ClO_4^-)\text{-}4(HClO_4)]^{7+}$ 729.1912, found 729.1922; calcd for $[M\text{-}6(ClO_4^-)\text{-}4(HClO_4)]^{6+}$ 867.2143, found 867.2156; calcd for $[M\text{-}5(ClO_4^-)\text{-}2(HClO_4)]^{5+}$ 1100.8293, found 1101.0303; calcd for $[M\text{-}4(ClO_4^-)\text{-}2(HClO_4)]^{4+}$ 1401.0238, found 1400.7736.

$[Sm_4L^1_4](ClO_4)_{12}$: ^1H NMR (400 MHz, CD_3CN) δ10.43(s, 3H), 9.16 (d, *J* = 6.4 Hz, 3H), 8.54(t, *J* = 4.0 Hz, 3H), 8.49 (s, 6H), 7.70 (d, *J* = 8.0 Hz, 6H), 7.21(t, *J* = 8.0 Hz, 15H), 7.10(s, 9H), 5.08 (m, *J* = 6.8 Hz, 3H), 1.69 (d, *J* = 6.8 Hz, 9H). ^{13}C NMR (101 MHz, CD_3CN) δ 170.17, 169.02, 150.06, 149.69, 144.29, 142.46, 140.20, 138.42, 135.51, 129.20, 128.18, 127.51, 126.28, 125.81, 123.72, 123.32, 52.85, 29.91, 21.33. ESI-TOF-MS calcd for $[M\text{-}8(ClO_4^-)\text{-}4(HClO_4)]^{8+}$ 628.8025, found 628.8033; calcd for $[M\text{-}7(ClO_4^-)\text{-}5(HClO_4)]^{7+}$ 718.4875, found 718.4881; calcd for $[M\text{-}6(ClO_4^-)\text{-}6(HClO_4)]^{6+}$ 838.0675, found 838.0682; calcd for $[M\text{-}5(ClO_4^-)\text{-}7(HClO_4)]^{5+}$ 1005.4796, found 1005.4798; calcd for $[M\text{-}4(ClO_4^-)\text{-}6(HClO_4)]^{4+}$ 1306.8255, found 1306.8249.

$[Y_4L^1_4](ClO_4)_{12}$: ^{1}H NMR (400 MHz, CD_3CN) δ 10.67(s, 3H), 9.18 (d, *J* = 6.8 Hz, 3H), 8.57(d, *J* = 8.0 Hz, 3H), 8.51(d, *J* = 8.0 Hz, 3H), 8.40(t, *J* = 8.0 Hz, 3H), 7.72 (d, *J* = 8.8 Hz, 6H), 7.21 (d, *J* = 8.8 Hz, 6H),7.16 (d, *J* = 6.8 Hz, 9H), 7.10(s, 3H), 7.01(t, *J* = 4.8 Hz, 6H), 4.96(m, *J* = 6.8 Hz, 3H), 1.64(d, *J* = 7.2 Hz, 9H). ^{13}C NMR (101 MHz, CD_3CN) δ 167.27, 166.50, 148.04, 147.68, 143.72, 142.41, 140.19, 138.45, 135.58, 129.17, 128.06, 127.52, 126.90, 126.51, 126.20, 123.70, 123.26, 122.92, 119.74, 52.80, 21.35. ESI-TOF-MS calcd for $[M\text{-}8(ClO_4^-)\text{-}4(HClO_4)]^{8+}$ 598.0462, found 598.0473; calcd for $[M\text{-}7(ClO_4^-)\text{-}5(HClO_4)]^{7+}$ 683.3375, found 683.3384; calcd for $[M\text{-}6(ClO_4^-)\text{-}6(HClO_4)]^{6+}$ 797.0592, found 797.0605; calcd for $[M\text{-}5(ClO_4^-)\text{-}7(HClO_4)]^{5+}$ 956.2696, found 956.2706; calcd for $[M\text{-}4(ClO_4^-)\text{-}5(HClO_4)]^{4+}$ 1270.3019, found 1270.3021.

$[Yb_4L^1_4](CF_3SO_3)_{12}$: ^{1}H NMR (400 MHz, CD_3CN) δ 11.94 (s, 6H), 10.57 (s, 3H), 9.67 (s, 6H), 9.37 (s, 3H), 8.34 (s, 3H), 7.94 (s, 3H), 7.24 (s, 3H), 7.04 (s, 6H), 6.65 (s, 6H), 6.49 (t, *J* = 7.6 Hz, 9H), 2.90 (s, 3H). ^{13}C NMR (101 MHz, CD_3CN) δ 153.79, 144.01, 142.36, 142.32, 141.14, 138.16, 130.01, 128.38, 127.23, 126.57, 126.02, 125.44, 123.27, 120.15, 119.14, 53.89, 22.83. ESI-TOF-MS calcd for $[M\text{-}9(ClO_4^-)\text{-}3(HClO_4)]^{9+}$ 569.0562, found 569.0542; calcd for $[M\text{-}8(ClO_4^-)\text{-}4(HClO_4)]^{8+}$ 640.0623, found 640.0601; calcd for $[M\text{-}7(ClO_4^-)\text{-}4(HClO_4)]^{7+}$ 745.7782, found 745.7759; calcd for $[M\text{-}6(ClO_4^-)\text{-}4(HClO_4)]^{6+}$ 886.5659, found 886.5631; calcd for $[M\text{-}5(ClO_4^-)\text{-}3(HClO_4)]^{5+}$ 1103.8599, found 1104.0558.

$[Lu_4L^1_4](CF_3SO_3)_{12}$: ^{1}H NMR (400 MHz, CD_3CN) δ 10.67(s, 3H), 9.18 (d, *J* = 6.4 Hz, 3H), 8.56 (d, *J* = 8.0 Hz, 3H), 8.50 (d, *J* = 8.0 Hz, 3H), 8.39(t, *J* = 8.0 Hz, 3H), 7.73(d, *J* = 8.8 Hz, 6H), 7.20(s, 6H), 7.17(d, *J* = 6.8 Hz, 9H), 7.10(s, 3H), 7.00(t, *J* = 4.6 Hz, 6H), 4.94(m, *J* = 7.2 Hz, 3H), 1.64(d, *J* = 7.2 Hz, 9H). ^{13}C NMR (101 MHz, CD_3CN) δ 167.46, 166.68, 147.66, 147.29, 143.59, 142.43, 140.14, 138.39, 135.54, 129.20, 128.06, 127.47, 126.91, 126.47, 126.15, 123.63, 123.14, 122.89, 119.71, 52.80, 21.41. ESI-TOF-MS calcd for $[M\text{-}8(CF_3SO_3^-)\text{-}4(HCF_3SO_3)]^{8+}$ 641.0637, found 641.0651; calcd for $[M\text{-}7(CF_3SO_3^-)\text{-}4(HCF_3SO_3)]^{7+}$ 753.9231, found 753.9250; calcd for $[M\text{-}6(CF_3SO_3^-)\text{-}3(HCF_3SO_3)]^{6+}$ 929.3956, found 929.3977; calcd for $[M\text{-}5(CF_3SO_3^-)\text{-}1(HCF_3SO_3)]^{5+}$ 1205.0490, found 1205.2510; calcd for $[M\text{-}4(CF_3SO_3^-)]^{4+}$ 1581.2898, found 1581.2908.

Synthesis and physical properties of $[Eu_1L^2_3]^{3+}$

A solution of $Eu(ClO_4)_3$•$6H_2O$ or $Eu(CF_3SO_3)_3$ (10.0 μmol, 1.0 equiv) in 1 mL CH_3CN was added to a solution of L^2 (30.0 μmol, 3.0 equiv) in 2 mL CH_3CN. The suspension turned into homogeneous yellow solution after stirring at 40 ℃ for 1 h. NMR and ESI-Q-TOF mass spectra confirmed the quantitative formation of $[EuL^2_3]^{3+}$ complexes, which is in accordance with the literature.[S2]

$[Eu_1L^2_3](CF_3SO_3)_3$: ^{1}H NMR (400 MHz, CD_3CN) δ 8.79–8.53 (m, 2H), 6.05 (t, *J* = 7.4 Hz, 2H), 5.81 (t, *J* = 7.6 Hz, 4H), 5.51 (d, *J* = 7.6 Hz, 4H), 5.32 (t, *J* = 7.8 Hz, 1H), 4.00 (d, *J* = 7.9 Hz, 2H), 3.36 (d, *J* = 5.8 Hz, 2H), 2.40 (d, *J* = 6.3 Hz, 6H). ESI-TOF-MS calcd for $[M\text{-}3(CF_3SO_3^-)]^{3+}$ 424.1526, found 424.1526; calcd for $[M\text{-}2(CF_3SO_3^-)]^{2+}$ 710.7051, found 710.7060; calcd for $[M\text{-}1(CF_3SO_3^-)]^{1+}$ 1719.1830, found 1719.1819; calcd for $[M\text{-}1(L^2)\text{-}1(CF_3SO_3^-)]^{1+}$ 1570.3626, found 1570.3622.

Synthesis and physical properties of $[Ln_2L^3_3]^{6+}$

To a yellow suspension of $\mathbf{L^3}$ (15.0 μmol, 1.5 equiv.) in 2 mL CH_3CN, a solution of $Ln(ClO_4)_3$•$6H_2O$ or $Ln(CF_3SO_3)_3$ (10.0 μmol, 1.0 equiv.) in 1 mL CH_3CN was added. The suspension turned into homogeneous yellow solution after stirring at 40 ℃ for 2 h. NMR and ESI-Q-TOF mass spectra confirmed the quantitative formation of $[Ln_2L^3_3]^{3+}$ complexes. Spectroscopic data for $[Eu_2L^3_3](OTf)_6$ are consistent with the literature.[S3]

$[La_2L^3_3](ClO_4)_6$: ^{1}H NMR (400 MHz, CD_3CN) δ 10.14(s, 2H), 8.81 (d, *J* = 6.8 Hz, 2H), 8.60 (t, *J* = 4.4 Hz, 2H), 8.51(d, *J* = 5.2 Hz, 4H), 7.71 (d, *J* = 8.8 Hz, 4H), 7.15(m, *J* = 5.6 Hz, 10H), 6.62 (d, *J* = 8.8 Hz, 4H), 5.18(m, *J* = 6.8 Hz, 2H), 1.73 (d, *J* = 6.8 Hz, 6H). ESI-TOF-MS calcd for $[M\text{-}4(ClO_4^-)\text{-}2(HClO_4)]^{4+}$ 585.4093, found 585.4098; calcd for $[M\text{-}3(ClO_4^-)]^{4+}$ 880.4993, found 880.4999; calcd for $[M\text{-}2(ClO_4^-)]^{2+}$ 1370.7237, found 1370.7248.

$[Ce_2L^3_3](CF_3SO_3)_6$: ^{1}H NMR (400 MHz, CD_3CN) δ 11.01(s, 2H), 10.01(s, 2H), 8.79 (t, *J* = 7.2 Hz, 6H), 8.47 (d, *J* = 7.2 Hz, 2H), 8.41(d, *J* = 7.2 Hz, 2H), 7.30(s, 4H), 7.19(d, *J* = 7.2 Hz, 4H), 7.15(s, 6H), 6.60(s, 2H), 2.31 (d, *J* = 6.0 Hz, 6H). ESI-TOF-MS calcd for $[M\text{-}5(CF_3SO_3^-)\text{-}1(HCF_3SO_3)]^{5+}$ 486.9286, found 486.9274; calcd for $[M\text{-}4(CF_3SO_3^-)\text{-}2(HCF_3SO_3)]^{4+}$ 585.9089, found 585.9074; calcd for $[M\text{-}3(CF_3SO_3^-)\text{-}3(HCF_3SO_3)]^{3+}$ 780.8761, found 780.8747; calcd for $[M\text{-}2(CF_3SO_3^-)]^{2+}$ 1471.2309, found 1471.2283.

$[Pr_2L^3_3](ClO_4)_6$: ^{1}H NMR (400 MHz, CD_3CN) δ 11.99(s, 2H), 11.41(s, 2H), 9.72(s, 4H), 9.57(s, 2H), 7.32(s, 6H), 7.16(s, 4H), 6.32(s, 4H), 5.75(s, 4H), 4.33(s, 2H), 1.35(s, 6H). ESI-TOF-MS calcd for [M-4(ClO_4^-

)-2($HClO_4$)]$^{4+}$ 586.4100, found 586.4140; calcd for [M-3(ClO_4^-)-1($HClO_4$)]$^{3+}$ 848.5150, found 848.5191; calcd for [M-2(ClO_4^-)]$^{2+}$ 1372.7250, found 1372.7296.

[$Nd_2L^3_3$](ClO_4)$_6$: ^{1}H NMR (400 MHz, CD_3CN) δ 12.40(s, 2H), 11.76(s, 2H), 9.23(s, 4H), 9.03(d, *J* = 7.6 Hz, 2H), 8.92(d, *J* = 7.6 Hz, 2H), 8.77 (t, *J* = 7.6 Hz, 2H), 7.38(s, 4H), 7.29(s, 4H), 7.15(s, 6H), 6.91(s, 2H). ESI-TOF-MS calcd for [M-5(ClO_4^-)-1($HClO_4$)]$^{5+}$ 470.5307, found 470.5299; calcd for [M-4(ClO_4^-)-2($HClO_4$)]$^{4+}$ 587.9116, found 587.9102; calcd for [M-3(ClO_4^-)-3($HClO_4$)]$^{3+}$ 783.5464, found 783.5448; calcd for [M-2(ClO_4^-)-1($HClO_4$)]$^{2+}$ 1325.2492, found 1325.7485.

[$Sm_2L^3_3$](ClO_4)$_6$: ^{1}H NMR (400 MHz, CD_3CN) δ 10.19(s, 2H), 9.07(d, *J* = 6.8 Hz, 2H), 8.44(t, *J* = 5.6 Hz, 6H), 7.71(d, *J* = 8.4 Hz, 4H), 7.21(d, *J* = 6.4 Hz, 6H), 7.08(t, *J* = 4.0 Hz, 4H), 6.61(d, *J* = 8.4 Hz, 4H), 5.15 (p, *J* = 6.8 Hz, 2H), 1.73 (d, *J* = 7.2 Hz, 6H). ESI-TOF-MS calcd for [M-5(ClO_4^-)-1($HClO_4$)]$^{5+}$ 473.1338, found 473.1331; calcd for [M-4(ClO_4^-)-2($HClO_4$)]$^{4+}$ 591.1654, found 591.1638; calcd for [M-3(ClO_4^-)-3($HClO_4$)]$^{3+}$ 787.8848, found 787.8830; calcd for [M-2(ClO_4^-)-2($HClO_4$)]$^{2+}$ 1282.2799, found 1282.2780.

[$Eu_2L^3_3$](ClO_4)$_6$: ^{1}H NMR (400 MHz, CD_3CN) δ 8.09(d, *J* = 5.6 Hz, 4H), 7.39(t, *J* = 7.6 Hz, 2H), 7.10(d, *J* = 4.0 Hz, 6H), 7.01 (d, *J* = 7.6 Hz, 4H), 6.96(m, *J* = 5.2 Hz, 6H), 6.57(t, *J* = 7.6 Hz, 4H), 5.51(s, 2H), 5.03 (d, *J* = 5.2 Hz, 2H), 1.79 (d, *J* = 6.0 Hz, 6H). ^{13}C NMR (101 MHz, CD_3CN) δ 164.89, 161.58, 156.12, 145.47, 144.45, 143.28, 138.74, 136.56, 129.16, 128.10, 127.85, 126.15, 123.02, 93.21, 92.69, 51.99, 47.64, 22.12, 8.82. ESI-TOF-MS calcd for [M-5(ClO_4^-)-1($HClO_4$)]$^{5+}$ 473.9350, found 473.9343; calcd for [M-4(ClO_4^-)-2($HClO_4$)]$^{4+}$ 591.9166, found 591.9155; calcd for [M-3(ClO_4^-)-3($HClO_4$)]$^{3+}$ 789.2201, found 789.2182; calcd for [M-2(ClO_4^-)-2($HClO_4$)]$^{2+}$ 1283.2816, found 1283.2796.

Synthesis and physical properties of [$Ln_4L^{4\text{-}6}_4$]$^{12+}$

[$Pr_4L^6_4$](CF_3SO_3)$_{12}$: ^{1}H NMR (400 MHz, CD_3CN) δ 12.56 (s, 3H), 10.30 (s, 3H), 10.06 (s, 3H), 9.40 (s, 3H), 6.10 (s, 9H), 5.90 (s, 6H), 3.68 (d, *J* = 24.7 Hz, 6H), 2.96 (s, 3H), 2.55 (s, 3H), 2.07 (s, 3H), 1.87 (s, 3H), 1.24 (d, *J* = 11.3 Hz, 96H), 0.91 (d, *J* = 5.8 Hz, 6H), 0.90–0.83 (m, 18H), 0.70 (s, 6H), 0.54 (s, 6H). ESI-TOF-MS calcd for [M-8($CF_3SO_3^-$)-4(HCF_3SO_3)]$^{8+}$ 972.7675, found 972.7676; calcd for [M-7($CF_3SO_3^-$)-5(HCF_3SO_3)]$^{7+}$ 1111.5904, found 1111.5908; calcd for [M-6($CF_3SO_3^-$)-6(HCF_3SO_3)]$^{6+}$ 1296.6875, found 1296.6874; calcd for [M-5($CF_3SO_3^-$)-7(HCF_3SO_3)]$^{5+}$ 1555.8236, found 1555.8223; calcd for [M-4($CF_3SO_3^-$)-6(HCF_3SO_3)]$^{4+}$ 2019.5074, found 2019.5039.

Single crystal X-ray diffraction studies.

X-ray data was collected at room temperature. The crystal was transferred and sealed inside a glass capillary with an atmosphere of the mother liquor without exposure to air. X-ray data collection at cryogenic conditions for these compounds resulted in the deterioration of crystallinity due to unknown reasons and gave worse quality of data. The reason for the deterioration of the crystals under cryogenic conditions possibly has something to do with the large cavities existing inside the big unit cells that are filled with amorphous organic solvents such as diethyl ether, THF and so on, which may still slowly diffuse/evaporate under liquid N_2 temperature. This is a quite different feature from biological samples which are always grown from water. So it is quite common to see X-ray data collected at room temperature for supramolecular systems. Our own experiences also suggest that to seal the crystals inside a glass capillary and collect the data quickly at room temperature may be a general protocol to follow for fragile crystals grown from volatile organic solvents.[S1, S4, S5]

Crystal data for **$Cd_4L^1_4$(ClO_4)$_8$**: Space group P$6_3$22, a=b=48.446(4) Å, c=35.873(3) Å, *V*=72916(13) Å^3, *Z* = 2, *T* = 293K. Anisotropic least-squares refinement for the framework atoms and isotropic refinement for the other atoms on 11880 independent merged reflections (R_{int} = 0.0639) converged at residual wR_2 = 0.2770 for all data; residual R_1 = 0.0991 for 10214 observed data [$I > 2\sigma(I)$], and goodness of fit (GOF) = 1.270.

Crystal data for **$La_4L^1_4$(ClO_4)$_{12}$**: Space group P$2_1$3, a = b = c =42.908(2) Å, *V* = 78995(13) Å^3, *Z* = 8, *T* = 293K. Anisotropic least-squares refinement for the framework atoms and isotropic refinement for the other atoms on 27430 independent merged reflections (R_{int} = 0.0838) converged at residual wR_2 = 0.2553 for all data; residual R_1 = 0.0940 for 21686 observed data [$I > 2\sigma(I)$], and goodness of fit (GOF) = 1.090.

Additional Comments: The crystals of these kinds of giant supramolecular assemblies diffract very weakly in nature. The diffractions for compound **$Cd_4L^1_4$(ClO_4)$_8$** and **$La_4L^1_4$(ClO_4)$_{12}$** are very limited even though we optimized the measurement based on synchrotron radiations. The final R factor was converged to very high

values, because the crystal was diffracting very weakly due to a large amount of amorphous solvents and anions, which occupy as much as 50.50% for $\mathbf{Cd_4L^1_4(ClO_4)_8}$ and 32.47% for $\mathbf{La_4L^1_4(ClO_4)_{12}}$ in their unit cells based on PLATON calculations[S6]. Especially for the structure of $\mathbf{Cd_4L^1_4(ClO_4)_8}$, where counter ions and solvent molecules were so highly disordered that they could not be reasonably located, the residual intensities were removed by PLATON/SQUEEZE routine[S6]. Still one A-alert and some B-alerts are found by the (IUCr) checkCIF routine, all of which are due to the poor diffraction nature of the crystals. Details on crystal data collection and refinement were summarized below:

A large number of restrains and constraints have to be applied to ensure the convergence of the refinement due to the poor data/parameter ratio. For the crystal structure of $Cd_4(L^1)_4$, two ligands, two Cd(II) ions, three and a half perchlorates and several hydrogen-bonded water molecules (no hydrogen was modeled in this case) are located in the asymmetrical unit. Organic ligands (“RESI 1” and “RESI 2”) and perchlorates (“RESI 3”) are separately labeled under the same scheme and are forced to adopt similar configurations as restrained by the “SAME” commands. For the Ligand structure, “AFIX 66” constraints have been applied to the six-membered aromatic rings including pyridines. Moreover, many geometrical restraints including “FLAT”, “DFIX”, “DANG”, “SADI” are applied to the ligands and the perchlorate ions based on the X-ray coordinates of a similar $Eu_4(L^1)_4$ structure from our previous work, which was determined to a much better resolution[S1]. More detailed information of these geometrical restraints can be found in the final .CIF files, where the full _shelx_res_files have now been incorporated.

Because of the restraints and constraints used, we have to emphasize that the main purpose of providing the X-ray structures is in general merely to confirm the connectivity of the target assemblies. For this reason, discussions based on the crystal structures are always kept to the minimum.

Supplementary References

1. Yan, L.-L., Tan, C.-H., Zhang, G.-L., Zhou, L.-P., Bünzli, J.-C., Sun, Q.-F. Stereocontrolled Self-Assembly and Self-Sorting of Luminescent Europium Tetrahedral Cages. *J. Am. Chem. Soc.* **137**, 8550-8555 (2015).
2. Hua, K. T., Xu, J., Quiroz, E. E., Lopez, S., Ingram, A. J., Johnson, V. A., Tisch, A. R., de Bettencourt-Dias, A., Straus, D. A., Muller, G. Structural and Photophysical Properties of Visible- and Near-IR-Emitting Tris Lanthanide(III) Complexes Formed with the Enantiomers of N,N'-Bis(1-phenylethyl)-2,6-pyridinedicarboxamide. *Inorg. Chem.* **51**, 647-660 (2012).
3. Yeung, C.-T.; Chan, W. T. K.; Yan, S.-C.; Yu, K.-L.; Yim, K.-H.; Wong, W.-T.; Law, G.-L. Lanthanide supramolecular helical diastereoselective breaking induced by point chirality: mixture or P-helix, M-helix *Chem. Commun.* **51**, 592-595 (2015).
4. Sun, Qing-Fu; Iwasa, Junji; Ogawa, Daichi; Ishido, Yoshitaka; Sato, Sota; Ozeki, Tomoji; Sei, Yoshihisa; Yamaguchi, Kentaro; Fujita, Makoto Self-Assembled $M_{24}L_{48}$ Polyhedra and Their Sharp Structural Switch upon Subtle Ligand Variation *Science*, **328**, 1144-1147 (2010).
5. Sun, Qing-Fu; Sato, Sota; Fujita, Makoto An M18L24 stellated cuboctahedron through post-stellation of an $M_{12}L_{24}$ core *Nature Chem.*, **4**, 330-333 (2012).
6. Spek, A. L., J. *Appl. Crystallogr*. **36**, 7-13 (2003).
7. Johnson, A. M., Young, M. C., Zhang, X., Julian, R. R., Hooley, R. J. Cooperative Thermodynamic Control of Selectivity in the Self-Assembly of Rare Earth Metal–Ligand Helices. *J. Am. Chem. Soc.* **135**, 17723-17726 (2013).
8. Bünzli, J.-C. G. Review: Lanthanide coordination chemistry: from old concepts to coordination polymers. *J. Coord. Chem.* **67**, 3706-3733 (2014).
