# Supplementary material for: A supramolecular lanthanide separation approach based on multivalent cooperative enhancement of metal ion selectivity
Source: Nat Commun. 2018 Feb 7;9:547. doi: 10.1038/s41467-018-02940-7 (PMC5803205; doi:10.1038/s41467-018-02940-7)
Supplement: Supplementary file 3 — Description of Additional Supplementary Files [file 41467_2018_2940_MOESM3_ESM.pdf]

Description of Additional Supplementary Files

File Name: Supplementary Data 1
Description: Crystal structure data 1

File Name: Supplementary Data 2
Description: Crystal structure data 2
